# Supplementary material for: LEGO® as a versatile platform for building reconfigurable low-cost lab equipment
Source: PLoS One. 2025 Aug 12;20(8):e0326938. doi: 10.1371/journal.pone.0326938 (PMC12342335; doi:10.1371/journal.pone.0326938)
Supplement: S1 File — (PDF) [file pone.0326938.s001.pdf]

Supplementary Information

LEGO® as a versatile platform for building reconfigurable low-cost lab equipment

Diane N. Jung[1], Kailey E. Shara[2], and Carson J. Bruns[1,2*]

[1]Paul M. Rady Department of Mechanical Engineering, University of Colorado Boulder, Boulder, CO, United States of America
[2]ATLAS Institute, University of Colorado Boulder, Boulder, CO, United States of America
[*]carson.bruns@colorado.edu

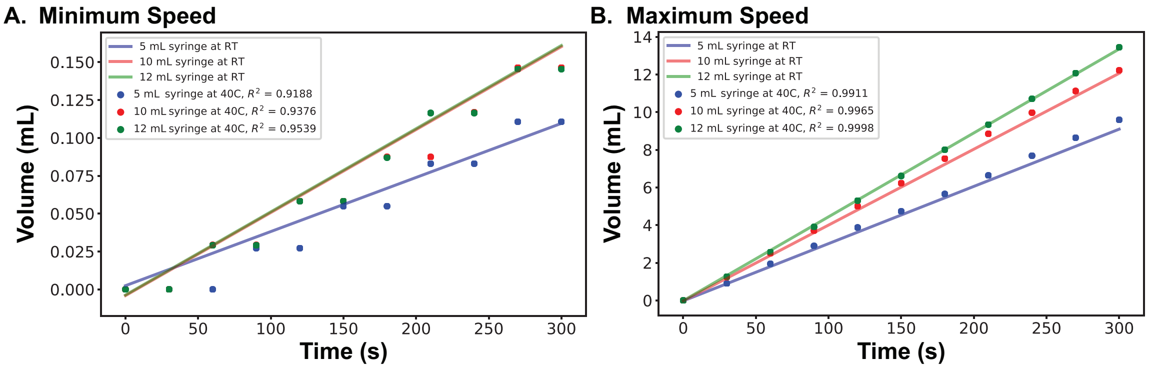

Figure S1: Dispense profiles of the syringe pump while operating at 45 °C in an oven. (**A**) Scatter plots of volume dispensed at the minimum speed setting overlaid on the slope of the same measurements obtained at RT (21 °C). (**B**) Scatter plots of volume dispensed at the maximum speed setting overlaid on the slope of the same measurements obtained at RT (21 °C).

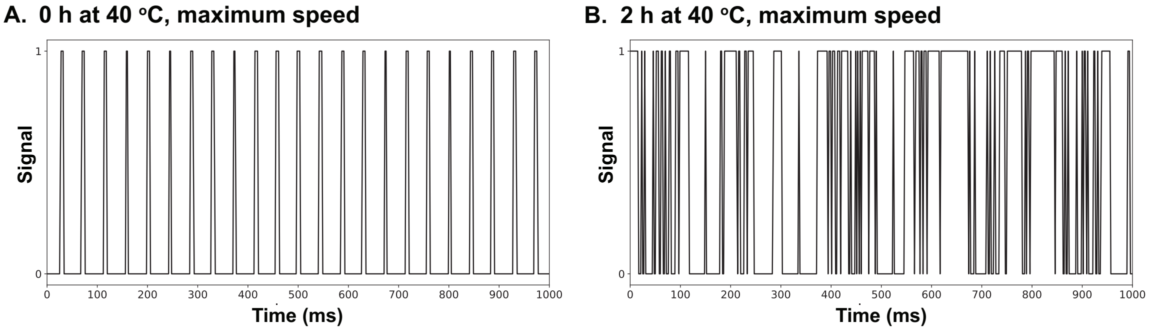

Figure S2: Representative photogate date detecting the rotating microcentrifuge arms (**A**) before and (**B**) after 2h of continuous maximum-speed operation at 45 °C in an oven.

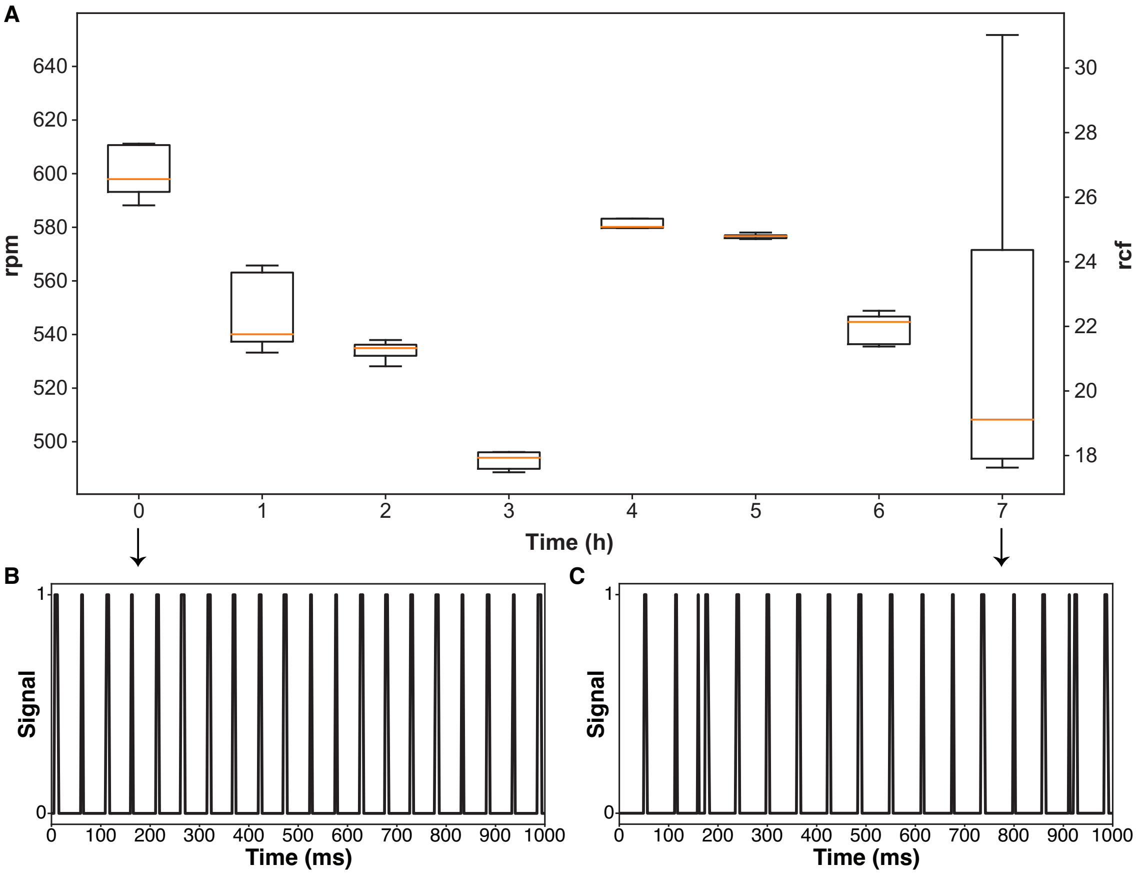

Figure S3: LEGO®-based microcentrifuge performance data over 7 h. (**A**) Box plots of revolutions per minute (rpm) and relative centrifugal force (rcf) measured for the LEGO®-based microcentrifuge over 7 h of continuous runtime in 1 h intervals. Each measurement represents the rpm calculations for each signal disturbance for 10-second recordings taken at one-minute intervals over a 5-min period, with a load of 2 microcentrifuge tubes filled with 1.5 mL of water. (**B**) One second of data output by a photogate used to detect revolutions of the rotor at maximum centrifugation speed at the beginning of the 7h trial. (**C**) One second of data output by a photogate used to detect revolutions of the rotor at maximum centrifugation speed after 7h runtime.

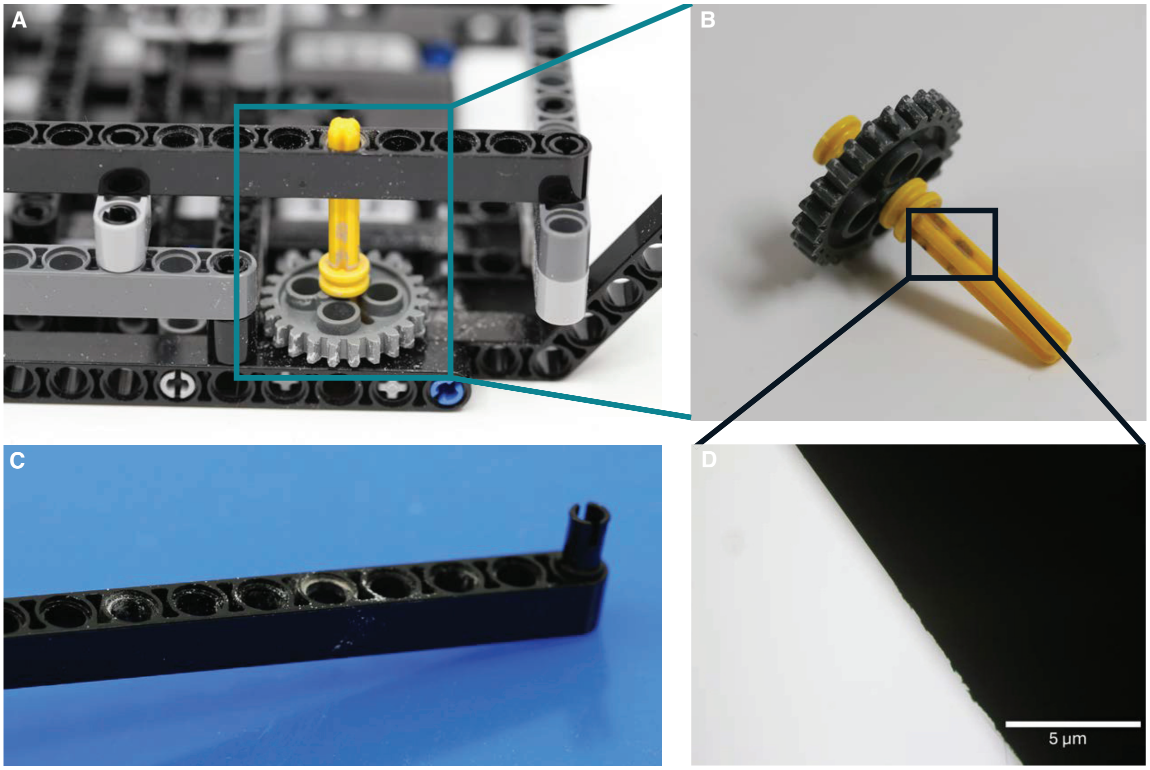

Figure S4: Failure analysis of the LEGO®-based microcentrifuge after 7 h of continuous maximum-speed operation at room temperature. (**A**) Photograph of the LEGO® Microcentrifuge after ∼ 7 h of continuous operation. White powder from worn plastic is observed around the LEGO® Technic™ gear and Technic™ liftarms. (**B**) Photograph of the LEGO® Technic™ axle and gear upon disassembly of the worn parts. (**C**) LEGO® Technic™ liftarm with white plastic residue on the holes nearest to the rotating axle of the device. (**D**) Magnified image of the LEGO® Technic™ axle, showing roughened edges at wear sites against the liftarm.

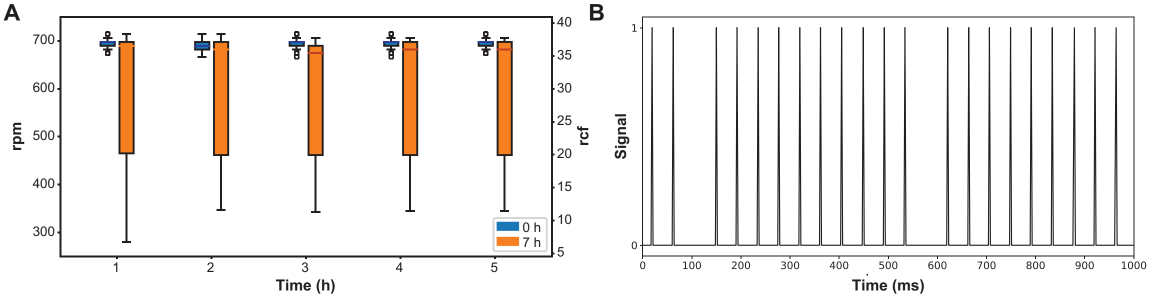

Figure S5: LEGO®-based microcentrifuge performance data with mineral oil lubricant to reduce wear to the coupling between the rotating axle and liftarm. (**A**) Box plots of revolutions per minute (rpm) and relative centrifugal force (rcf) measured for the LEGO®-based microcentrifuge after application of lubricant at time points of 0 h and 7h of continuous operation. Each measurement represents the rpm calculations for each signal disturbance for 10-second recordings taken at one-minute intervals over a 5-min period, with a load of 2 microcentrifuge tubes filled with 1.5 mL of water. (**B**) One second of data output by a photogate used to detect revolutions of the rotor at maximum centrifugation speed after 7 h of continuous operation at maximum speed with lubricant.

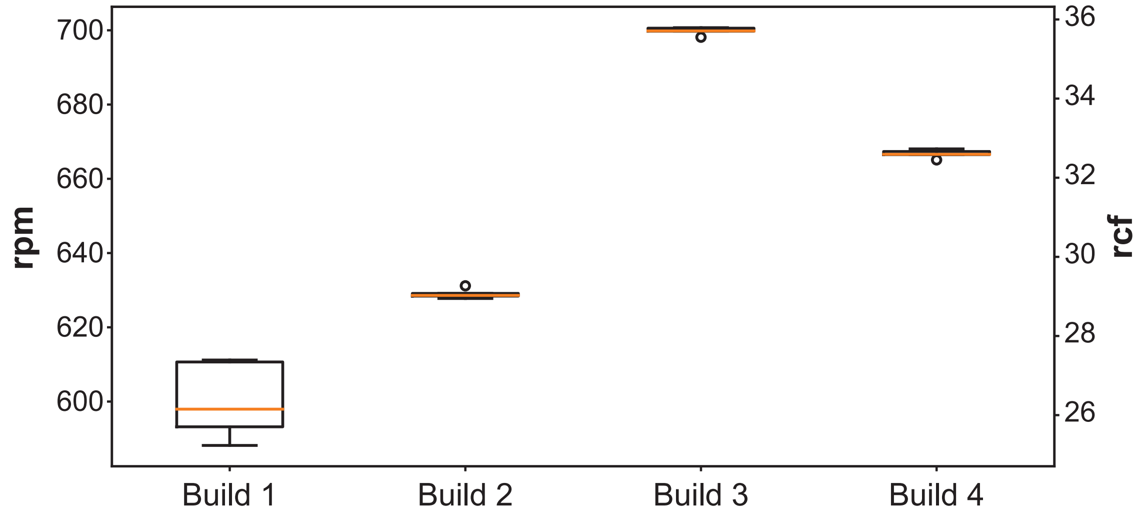

Figure S6: Box plots of the average revolutions per minute (rpm) and relative centrifugal force (rcf) for four different builds of the LEGO® microcentrifuge operating at the maximum motor speed setting of 9. Builds 1–2 had a load of 2 mL water; builds 3–4 had a load of 3 mL water.

Table S1: Table of LEGO® Technic parts prices used in the LEGO®-based lab equipment. Lowest available pricing for all parts were found on bricklink.com in June of 2024.

	Price (USD)	Orbital Shaker		Syringe Pump		Centrifuge	
Part #	June, 2024	#	price	#	price	#	price
32278	0.01	26	0.26	5	0.05	17	0.17
32525	0.0074	1	0.0074	2	0.0148	1	0.0074
32009	0.0058	8	0.0464	0	0	8	0.0464
40490	0.0075	7	0.0525	8	0.06	4	0.03
6629	0.0075	6	0.045	2	0.015	8	0.06
32524	0.0075	5	0.0375	12	0.09	6	0.045
32348	0.007	12	0.084	0	0	2	0.014
32526	0.0064	4	0.0256	28	0.1792	2	0.0128
32316	0.001	9	0.009	21	0.021	1	0.001
32140	0.0039	13	0.0507	12	0.0468	4	0.0156
60484	0.0075	8	0.06	5	0.0375	0	0
32523	0.0039	13	0.0507	7	0.0273	3	0.0117
60483	0.0032	0	0	1	0.0032	0	0
41677	0.0073	0	0	12	0.0876	0	0
6632	0.0073	8	0.0584	10	0.073	0	0
11478	0.03	0	0	4	0.12	0	0
41239	0.01	0	0	4	0.04	0	0
64179	0.32	7	2.24	5	1.6	1	0.32
14720	0.04	0	0	3	0.12	0	0
32449	0.0073	0	0	2	0.0146	0	0
43857	0.01	0	0	0	0	4	0.04
3706	0.0036	4	0.0144	1	0.0036	0	0
3705	0.0036	4	0.0144	0	0	0	0
55013	0.005	2	0.01	0	0	0	0
6587	0.0039	4	0.0156	2	0.0078	0	0
44294	0.0054	3	0.0162	4	0.0216	3	0.0162
18651	0.0039	6	0.0234	6	0.0234	5	0.0195
15100	0.01	1	0.01	3	0.03	0	0
32013	0.001	3	0.003	2	0.002	3	0.003
11214	0.0039	7	0.0273	9	0.0351	1	0.0039
32073	0.0036	15	0.054	0	0	13	0.0468
15462	0.0039	3	0.0117	0	0	1	0.0039
4519	0.0027	12	0.0324	6	0.0162	9	0.0243
43093	0.0011	5	0.0055	16	0.0176	8	0.0088
32184	0.0054	14	0.0756	10	0.054	11	0.0594
42003	0.0039	1	0.0039	3	0.0117	2	0.0078
22961	0.0074	2	0.0148	2	0.0148	2	0.0148
6536	0.0039	9	0.0351	7	0.0273	9	0.0351
32039	0.005	0	0	2	0.01	1	0.005
32905	0.05	0	0	8	0.4	0	0
3707	0.0053	0	0	3	0.0159	0	0

Continuation of Table S1							
	**Price (USD)**	**Orbital Shaker**		**Syringe Pump**		**Centrifuge**	
**Part #**	**June, 2024**	**#**	**price**	**#**	**price**	**#**	**price**
3737	0.0095	0	0	3	0.0285	0	0
50451	0.42	0	0	3	1.26	0	0
60485	0.0075	0	0	2	0.015	1	0.0075
32062	0.0011	0	0	8	0.0088	0	0
3704	0.0054	0	0	0	0	2	0.0108
32291	0.0089	0	0	0	0	2	0.0178
48989	0.0039	6	0.0234	0	0	9	0.0351
32054	0.0021	17	0.0357	11	0.0231	12	0.0252
87082	0.01	7	0.07	2	0.02	7	0.07
2780	0.0001	70	0.007	95	0.0095	49	0.0049
731c04	0.1	8	0.8	0	0	0	0
32556	0.0039	25	0.0975	0	0	26	0.1014
6558	0.005	56	0.28	99	0.495	0	0
32014	0.0043	0	0	8	0.0344	0	0
32034	0.005	0	0	2	0.01	0	0
60176	0.0008	0	0	0	0	0	0
32270	0.0086	1	0.0086	3	0.0258	1	0.0086
32269	0.009	1	0.009	5	0.045	0	0
4265c	0.0011	20	0.022	10	0.011	8	0.0088
3713	0.001	23	0.023	16	0.016	1	0.001
10928	0.02	0	0	3	0.06	0	0
3648	0.02	0	0	2	0.04	1	0.02
32498	0.37	0	0	0	0	1	0.37
3649	0.2	0	0	0	0	1	0.2
94925	0.01	0	0	2	0.02	0	0
95646c01	37.21	1	37.21	1	37.21	1	37.21
95658	12.95	1	12.95	0	0	1	12.95
95648	2.4	0	0	2	4.8	0	0
99455	19.2	0	0	1	19.2	0	0
	**TOTAL**	**458**	**54.9307**	**505**	**66.6231**	**252**	**52.0635**
