# Supplementary material for: LEGO® as a versatile platform for building reconfigurable low-cost lab equipment
Source: PLoS One. 2025 Aug 12;20(8):e0326938. doi: 10.1371/journal.pone.0326938 (PMC12342335; doi:10.1371/journal.pone.0326938)

# Assembly Instructions

Syringe Pump

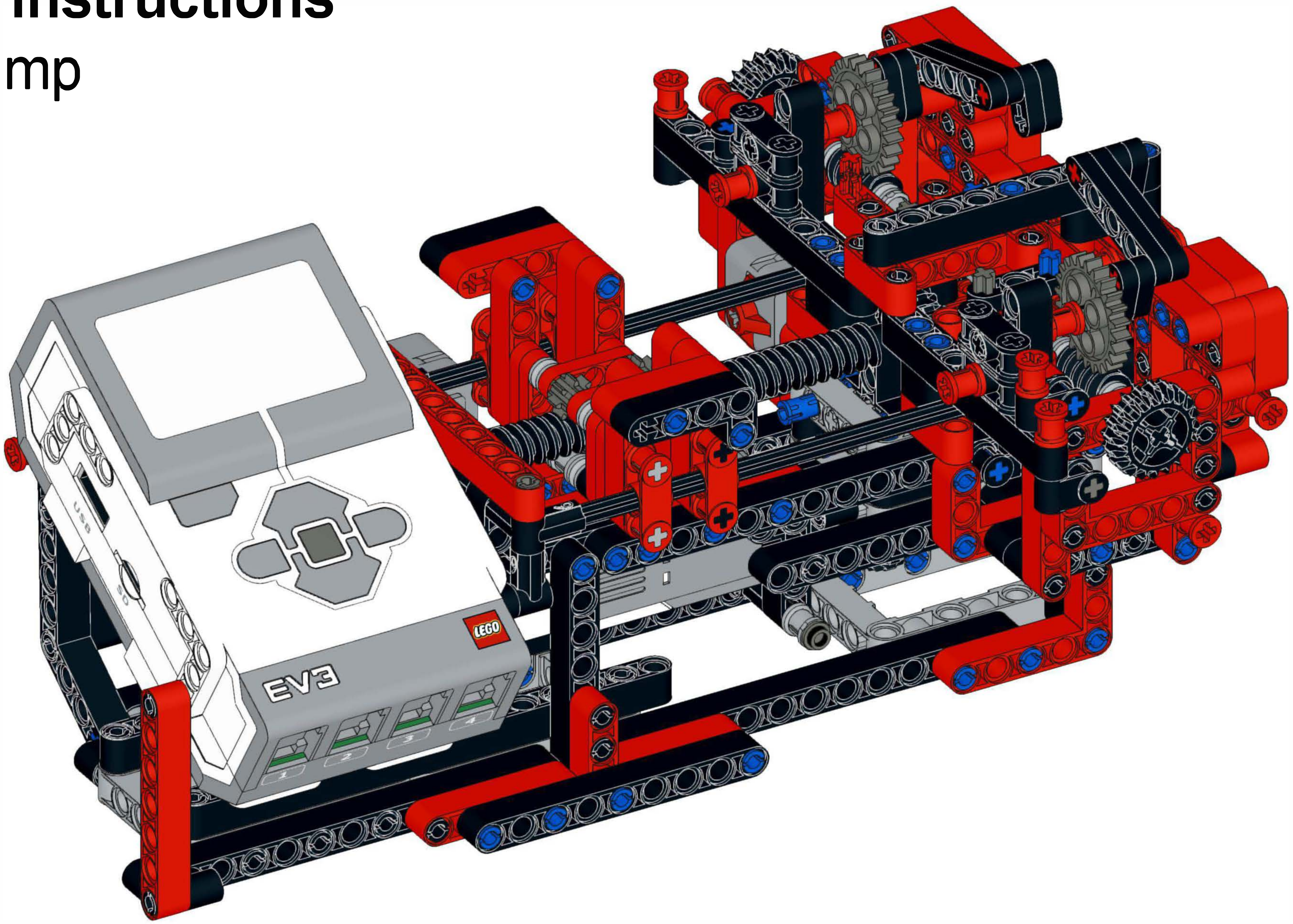

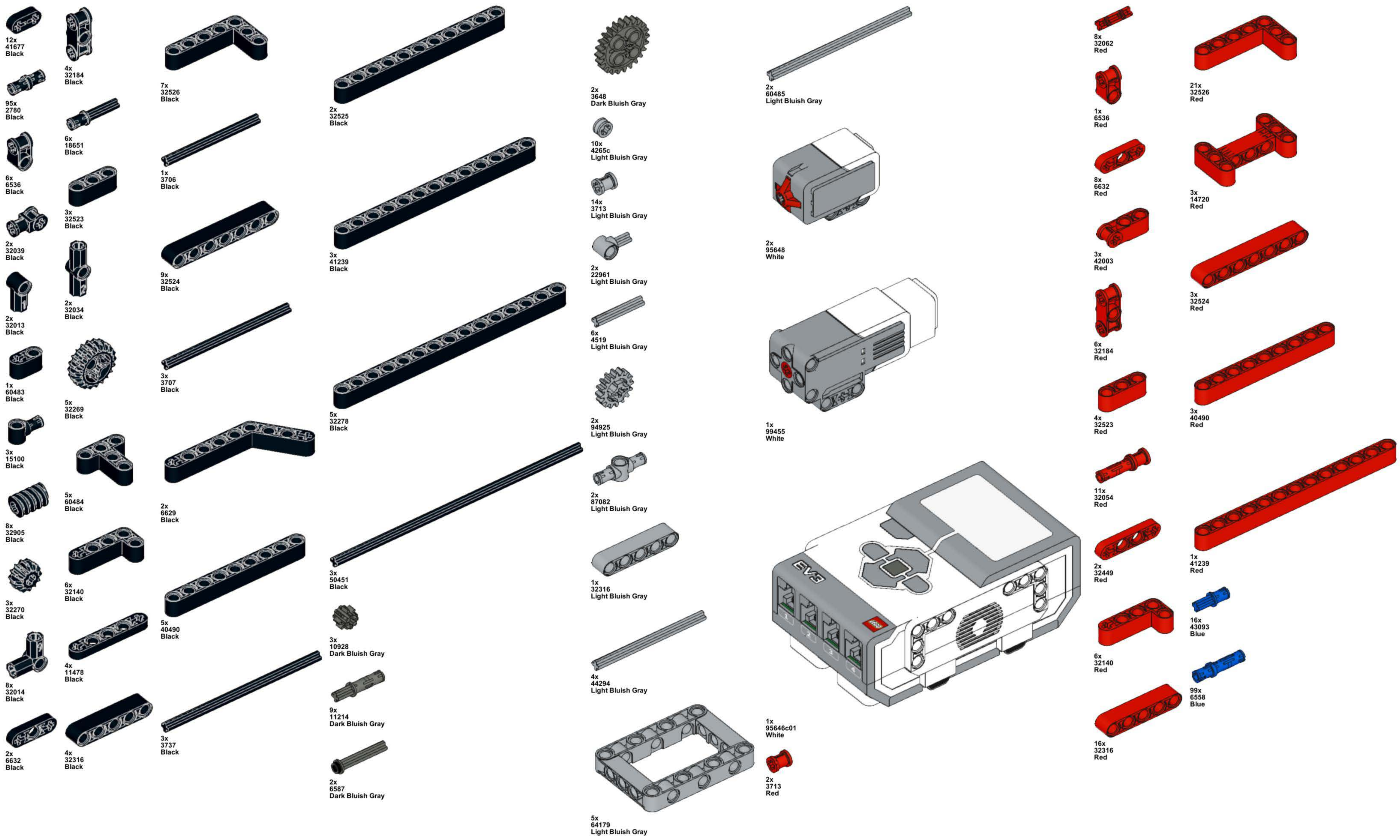
12x 41677 Black
95x 2780 Black
6x 6536 Black
2x 32039 Black
2x 32013 Black
1x 60483 Black
3x 15100 Black
8x 32905 Black
3x 32270 Black
8x 32014 Black
2x 6632 Black
4x 32184 Black
6x 18651 Black
3x 32523 Black
2x 32034 Black
5x 32269 Black
5x 60484 Black
6x 32140 Black
4x 11478 Black
4x 32316 Black
7x 32526 Black
1x 3706 Black
9x 32524 Black
3x 3707 Black
2x 6629 Black
5x 40490 Black
3x 3737 Black
2x 32525 Black
3x 41239 Black
5x 32278 Black
3x 50451 Black
3x 10928 Dark Bluish Gray
9x 11214 Dark Bluish Gray
2x 6587 Dark Bluish Gray
2x 3648 Dark Bluish Gray
10x 4265c Light Bluish Gray
14x 3713 Light Bluish Gray
2x 22961 Light Bluish Gray
6x 4519 Light Bluish Gray
2x 94925 Light Bluish Gray
2x 87082 Light Bluish Gray
1x 32316 Light Bluish Gray
4x 44294 Light Bluish Gray
5x 64179 Light Bluish Gray
2x 60485 Light Bluish Gray
2x 95648 White
1x 99455 White
1x 95646c01 White
2x 3713 Red
8x 32062 Red
1x 6536 Red
8x 6632 Red
3x 42003 Red
6x 32184 Red
4x 32523 Red
11x 32054 Red
2x 32449 Red
6x 32140 Red
16x 32316 Red
21x 32526 Red
3x 14720 Red
3x 32524 Red
3x 40490 Red
1x 41239 Red
16x 43093 Blue
99x 6558 Blue

## 1

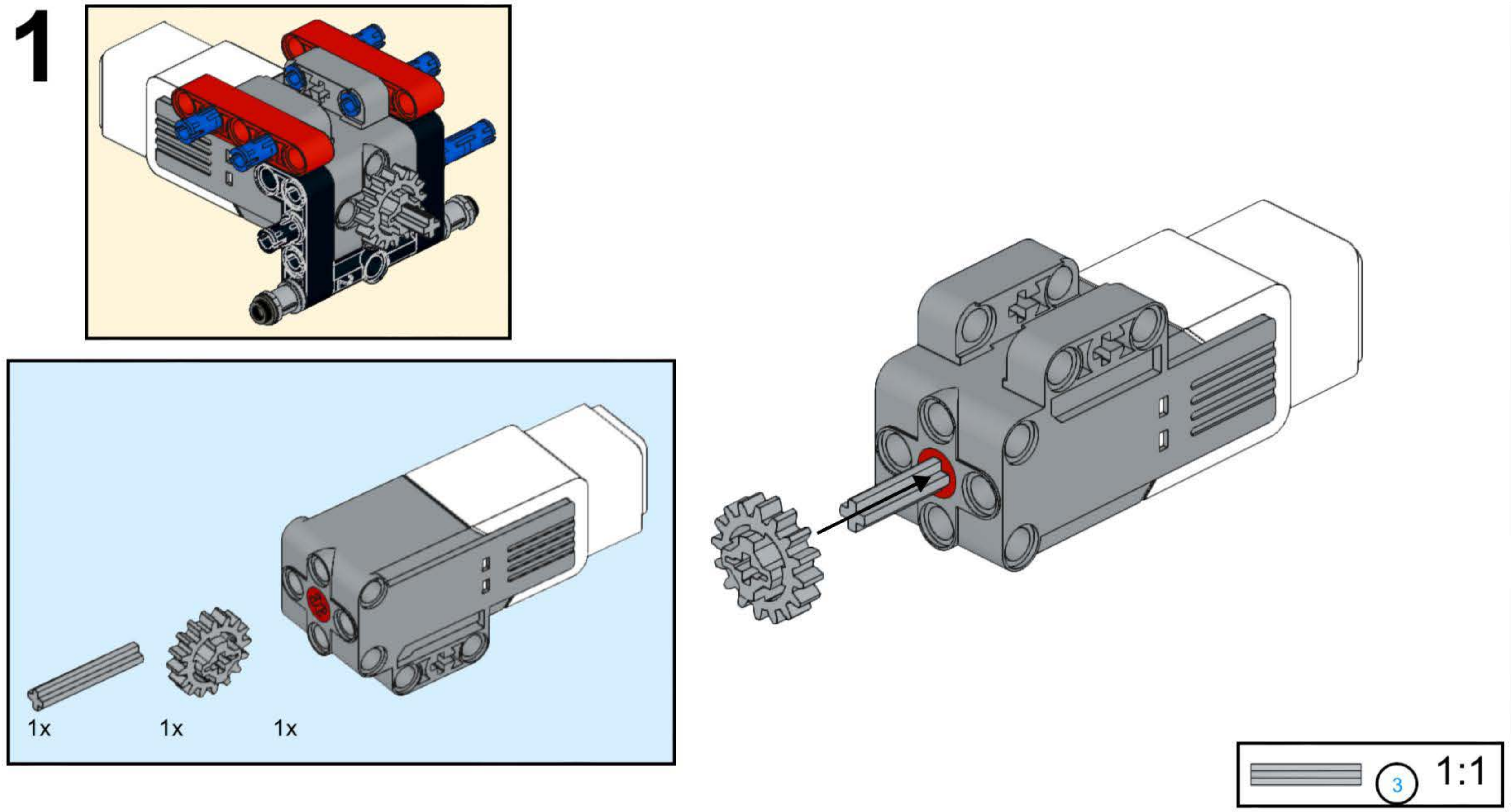


## 2

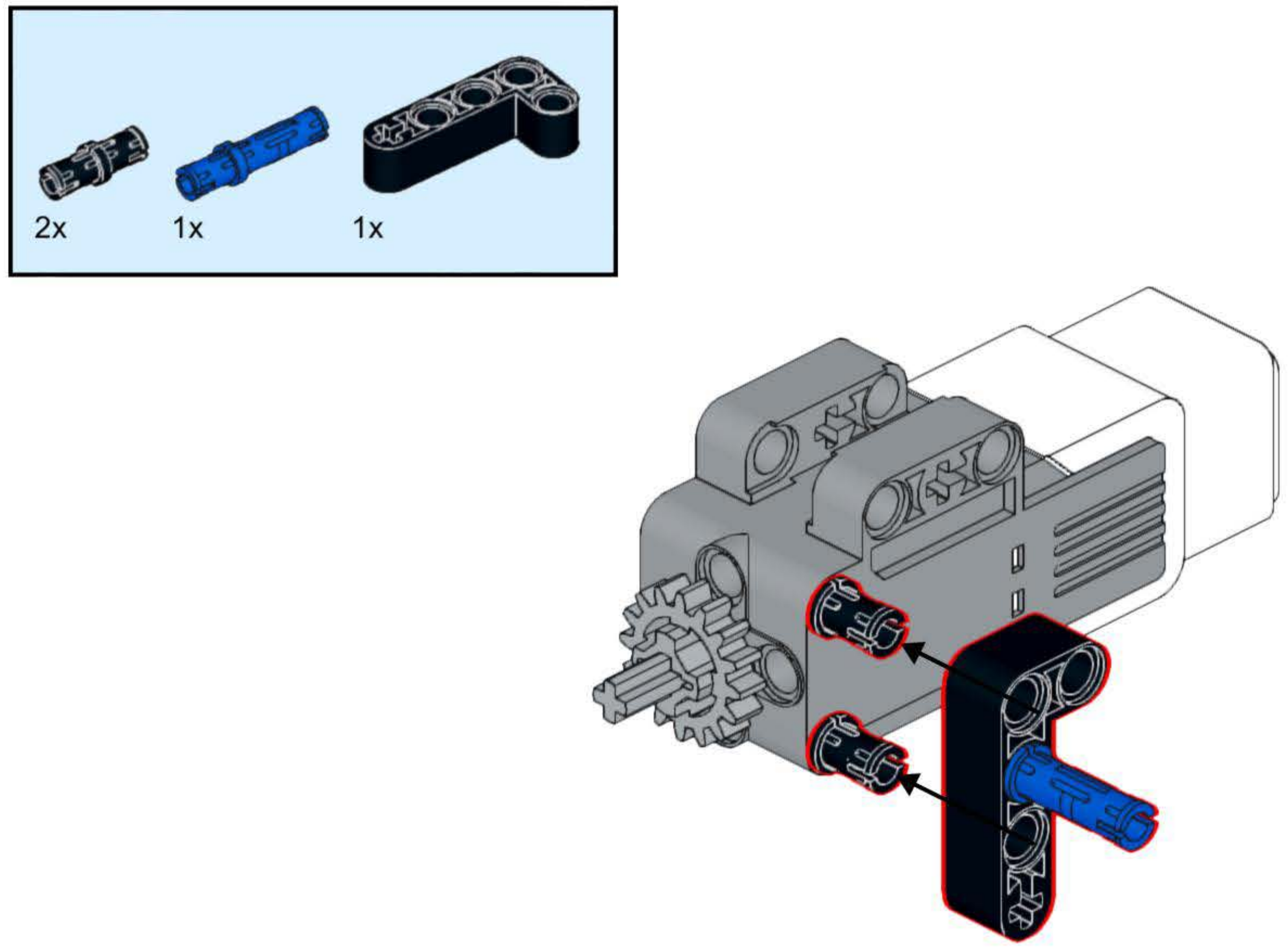


## 3

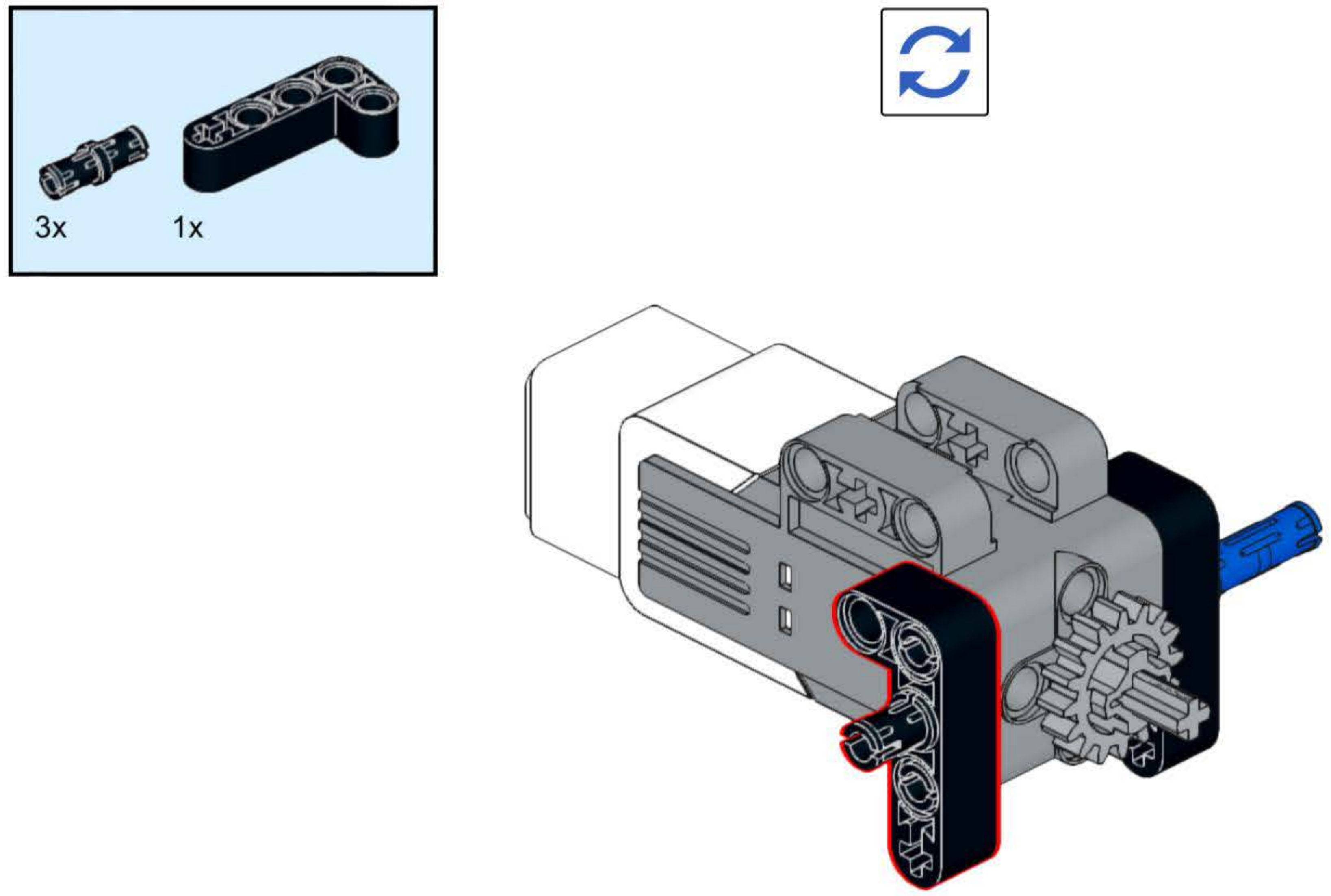


## 4

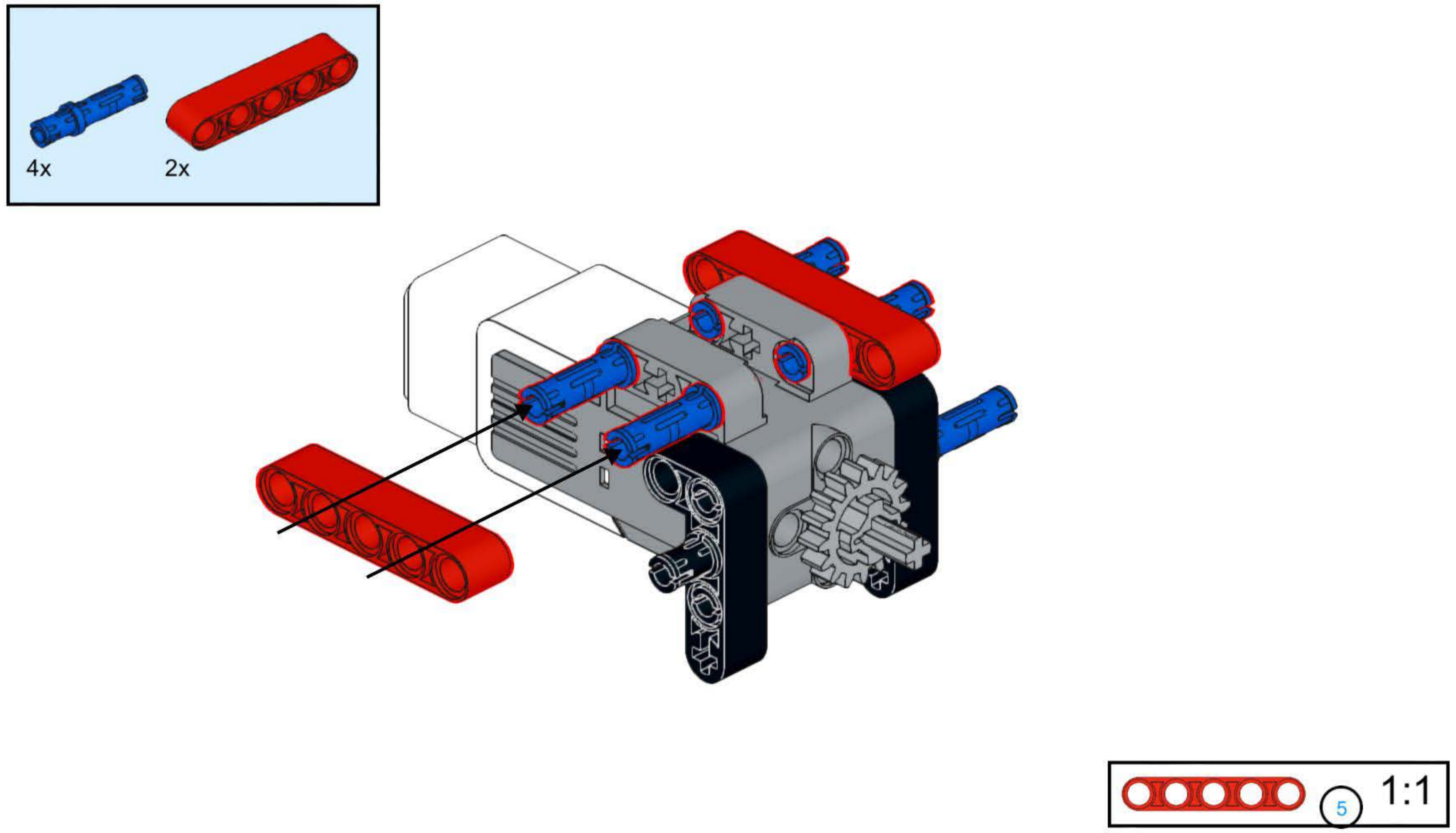

5

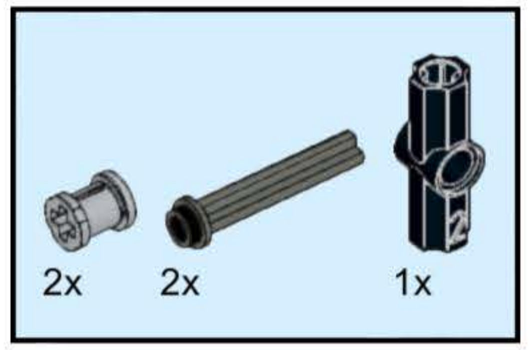


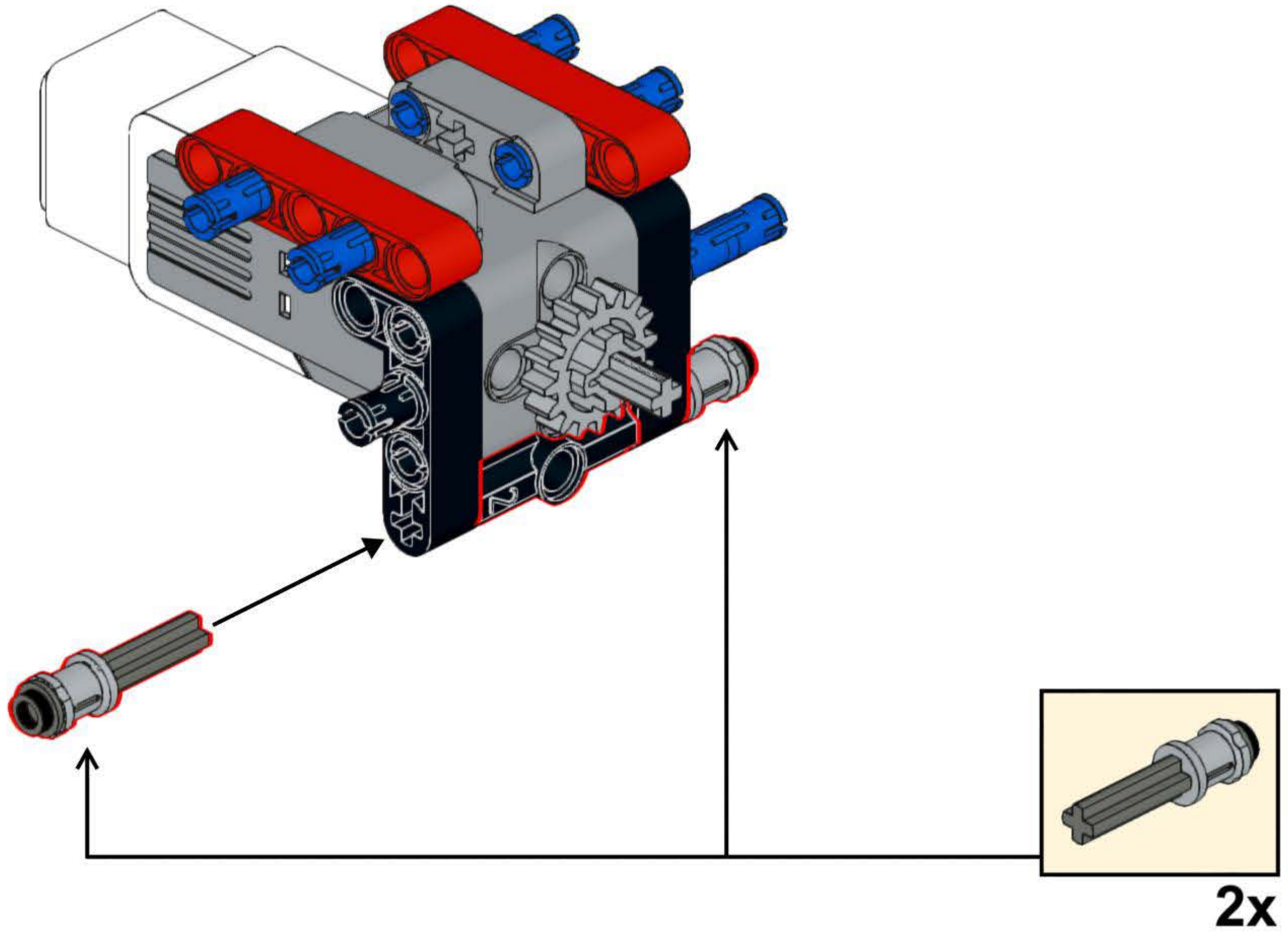


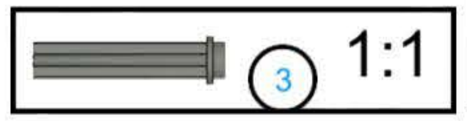


6

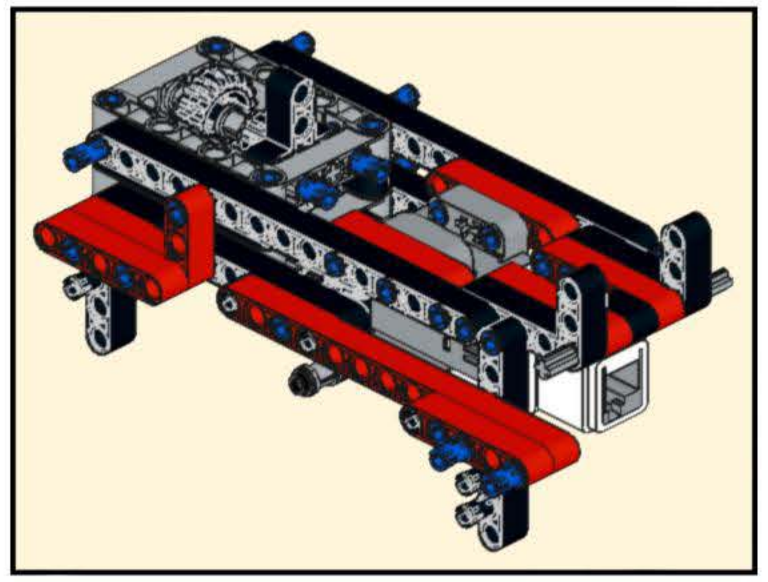

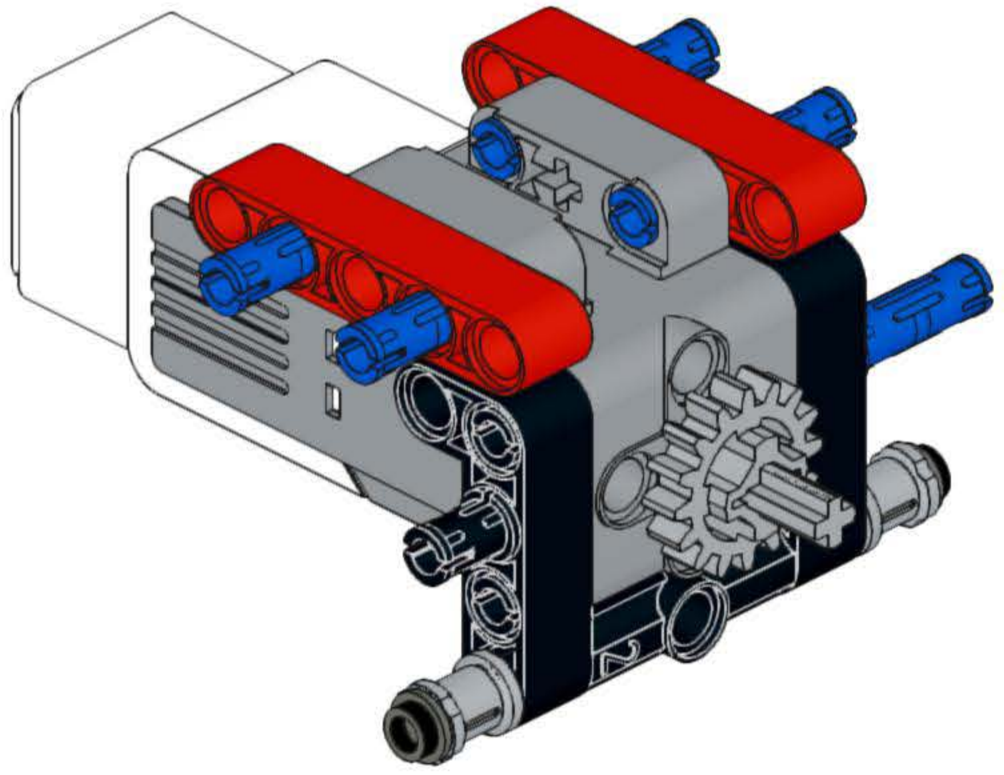

7

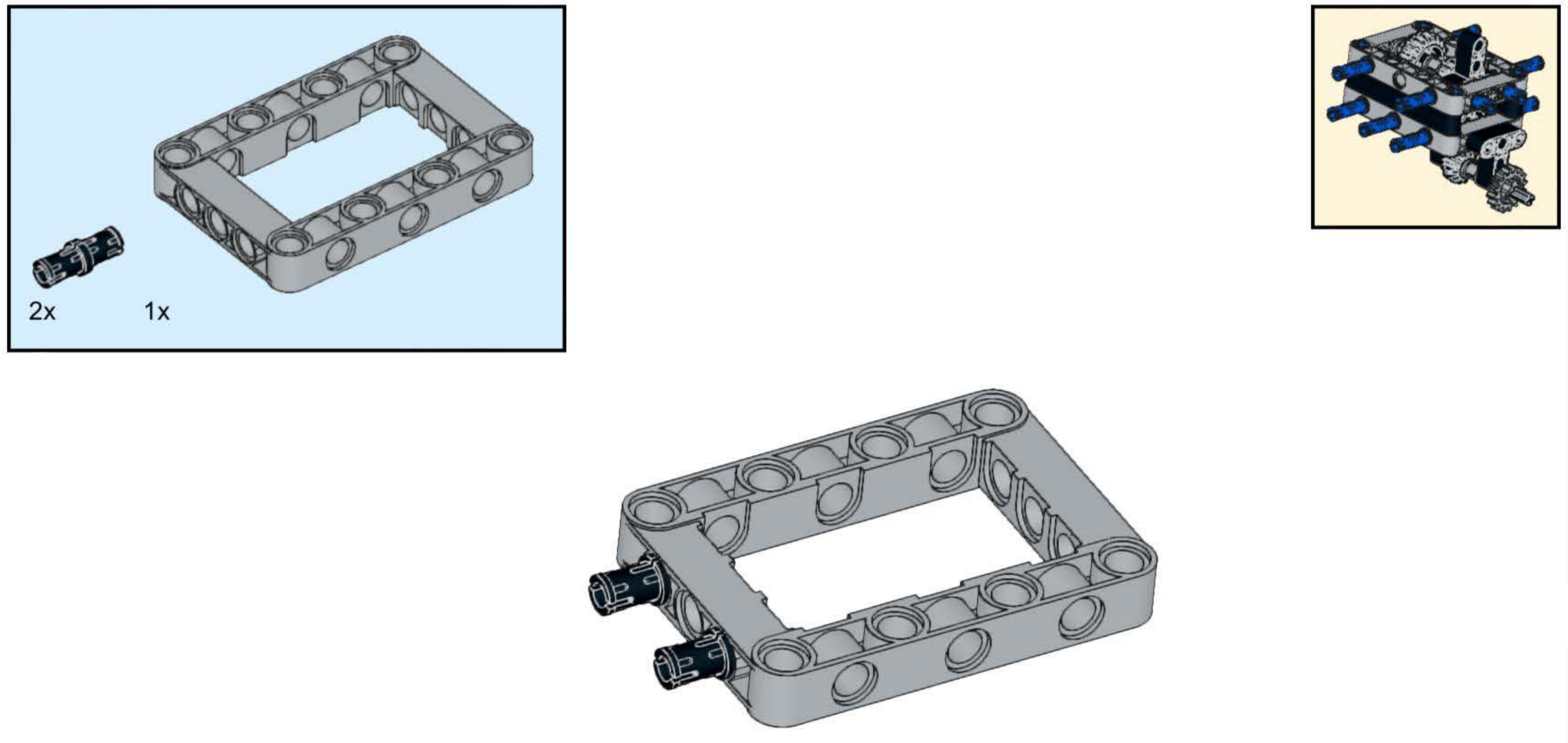


8

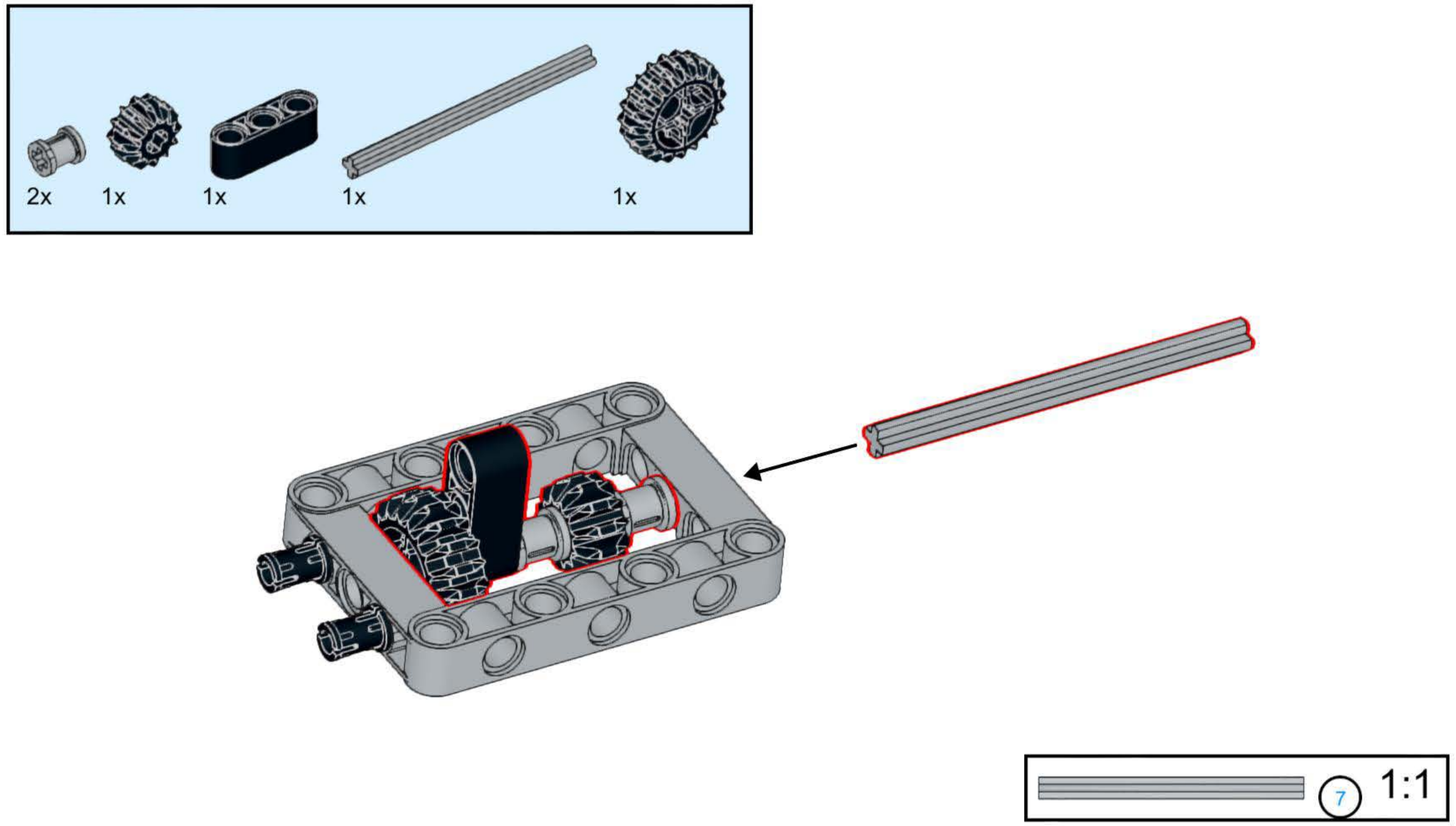


9

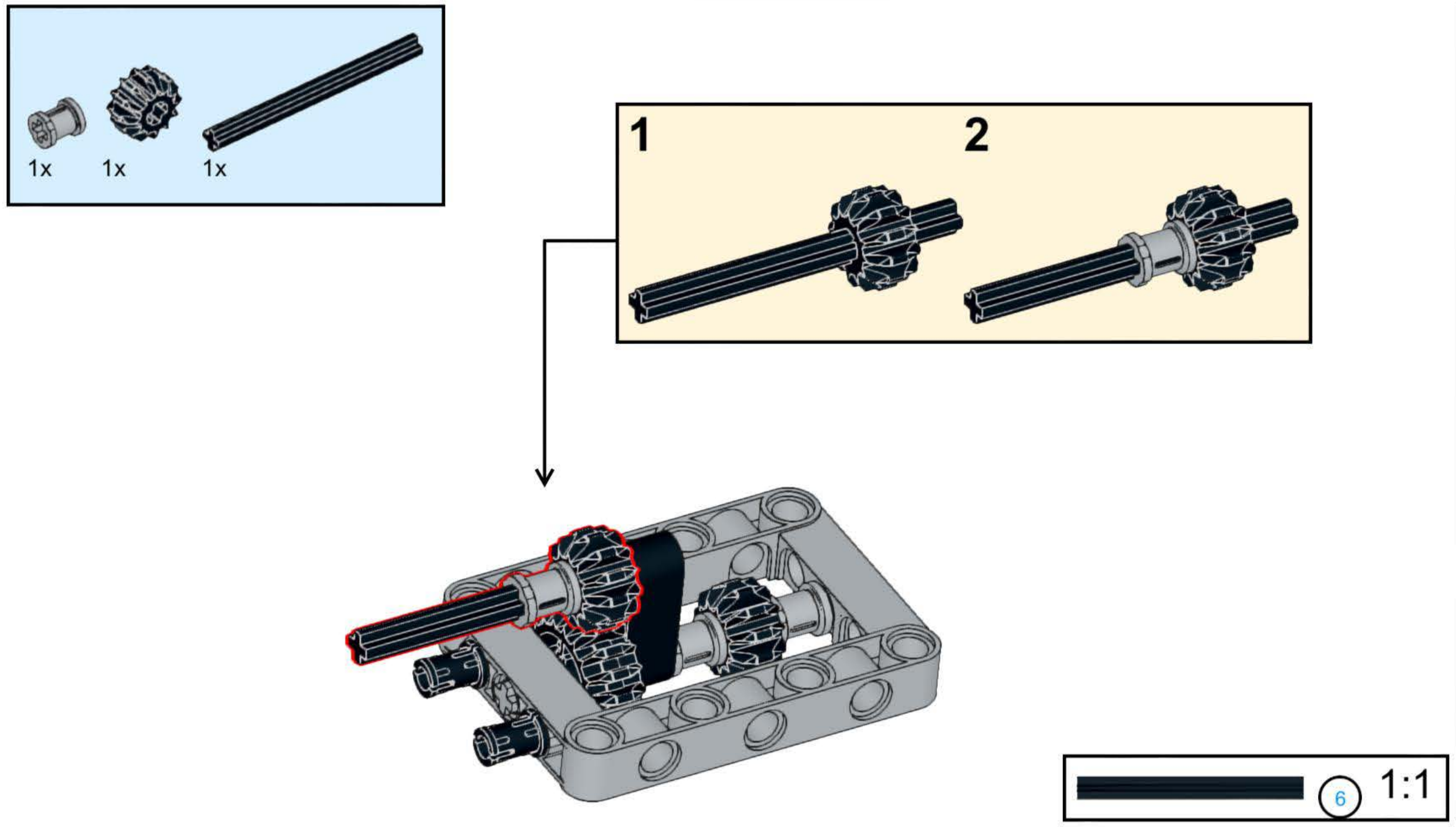


10

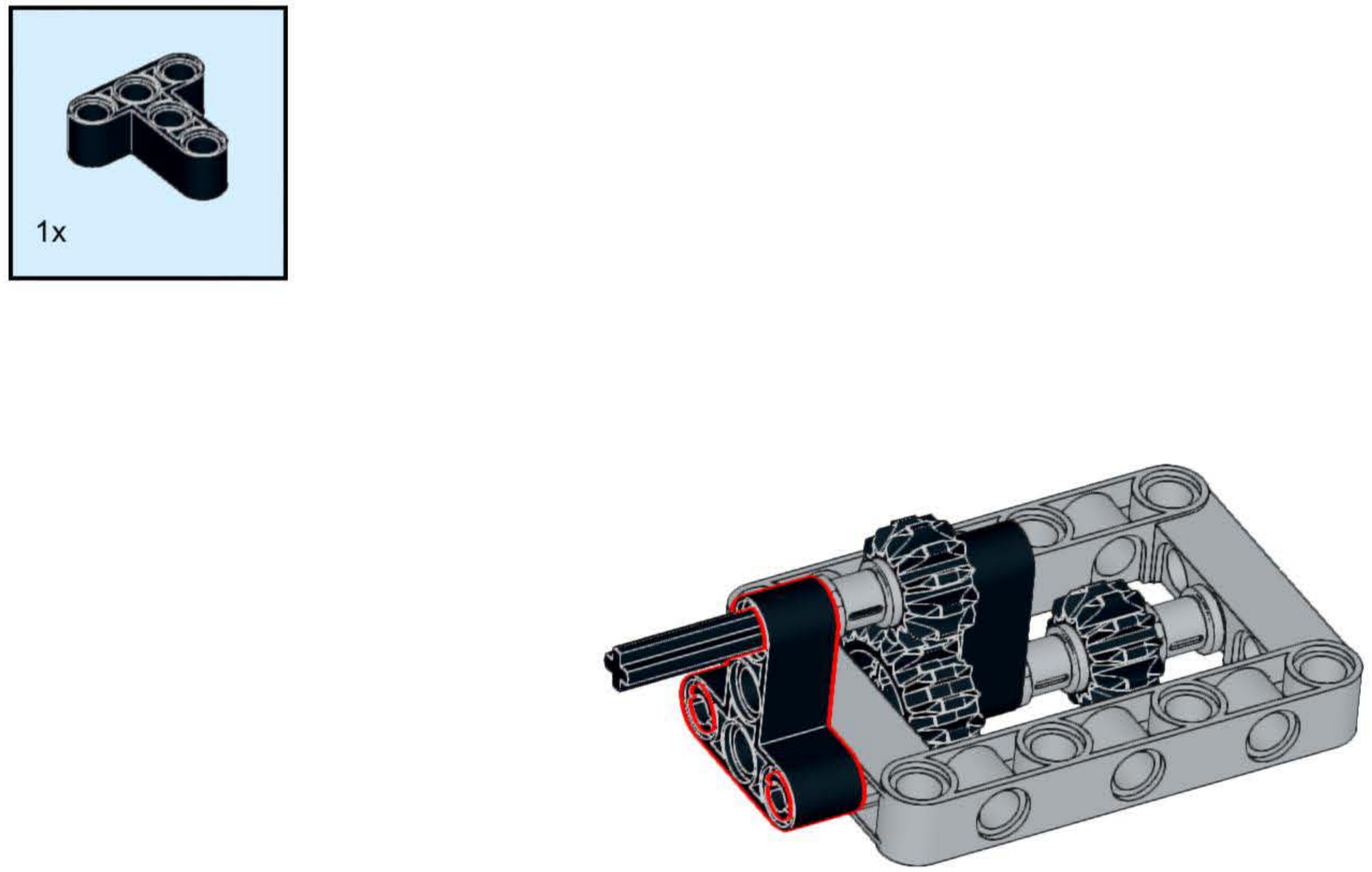

## 11

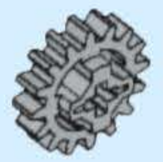

1x

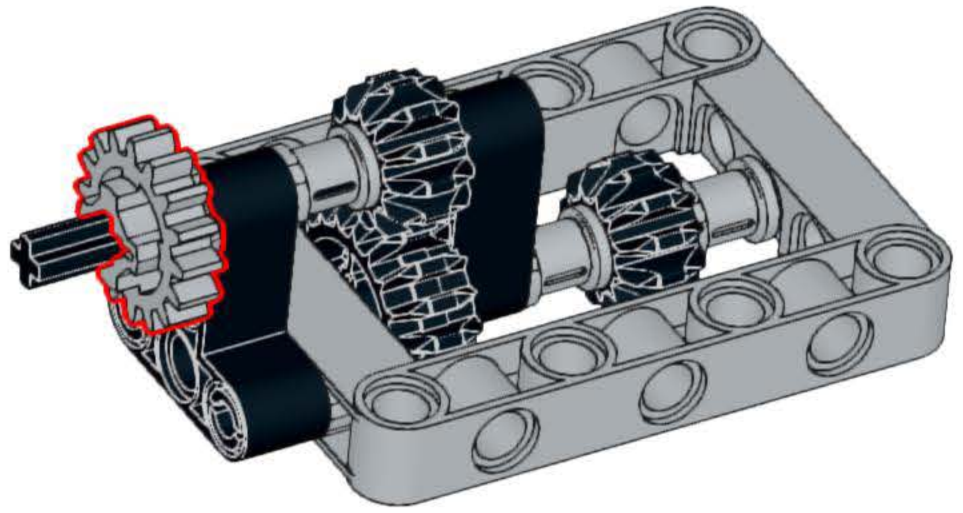

## 12

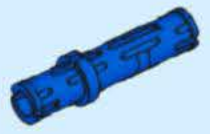

4x

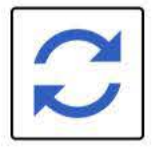

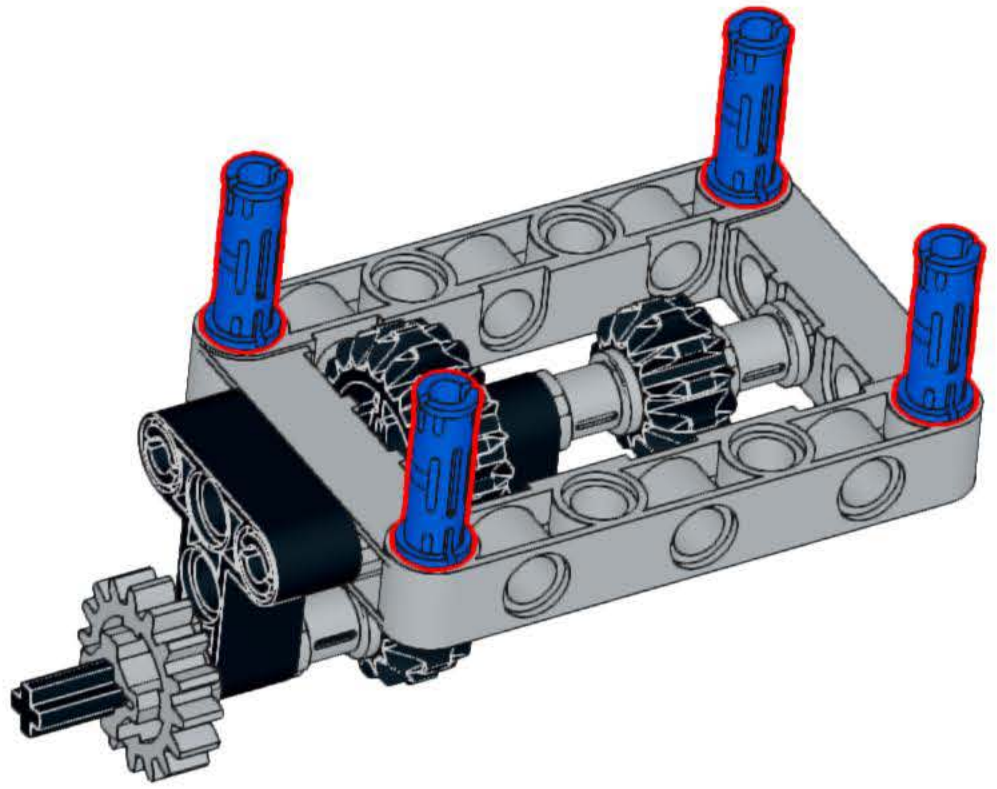

## 13

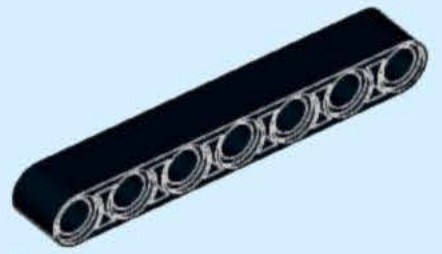

2x

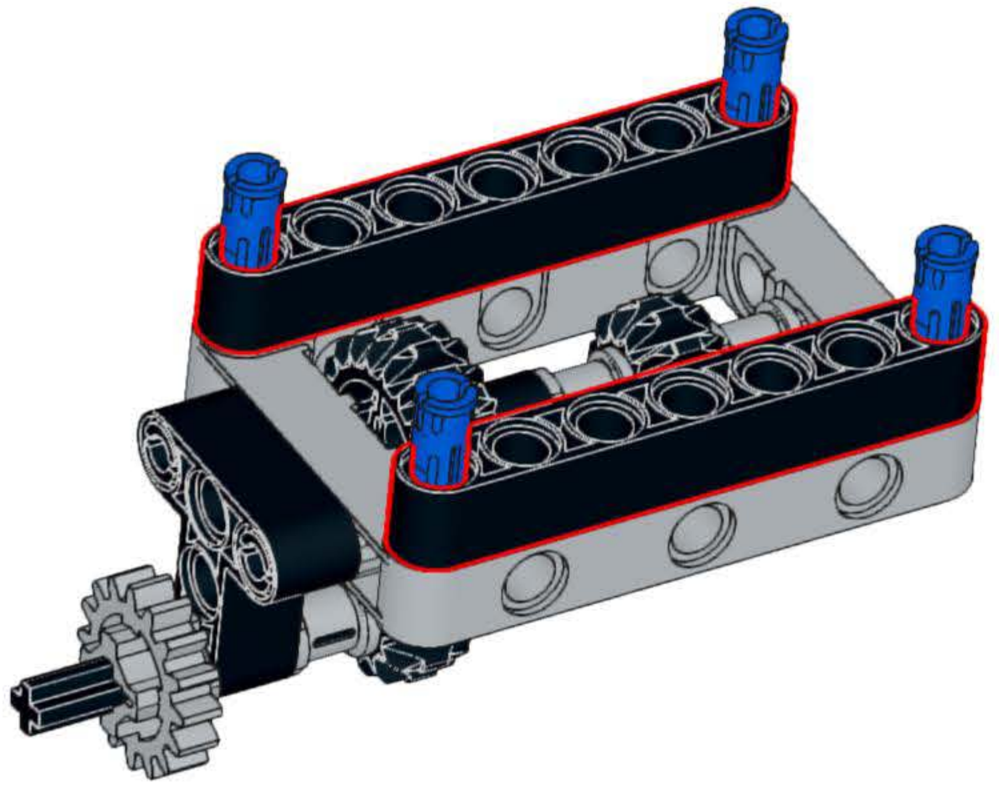

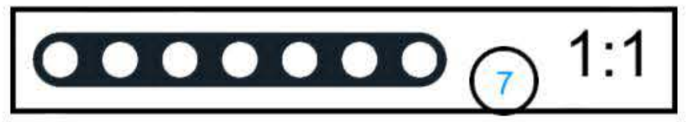

# 14

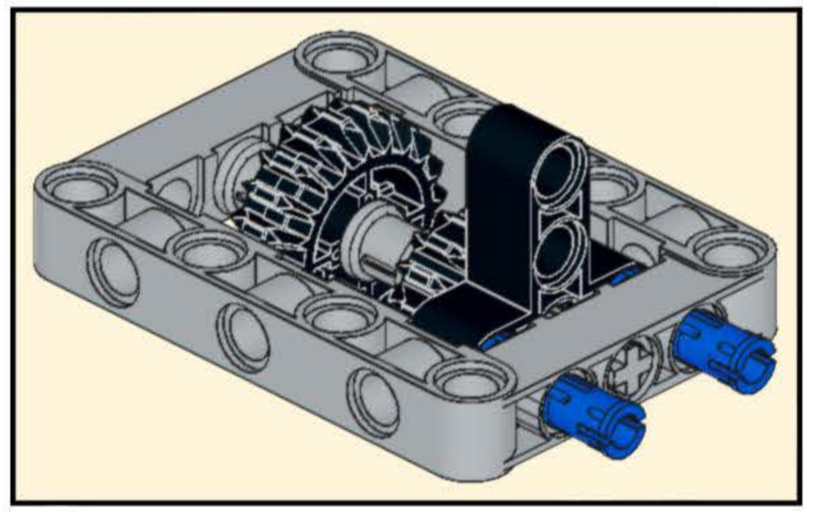

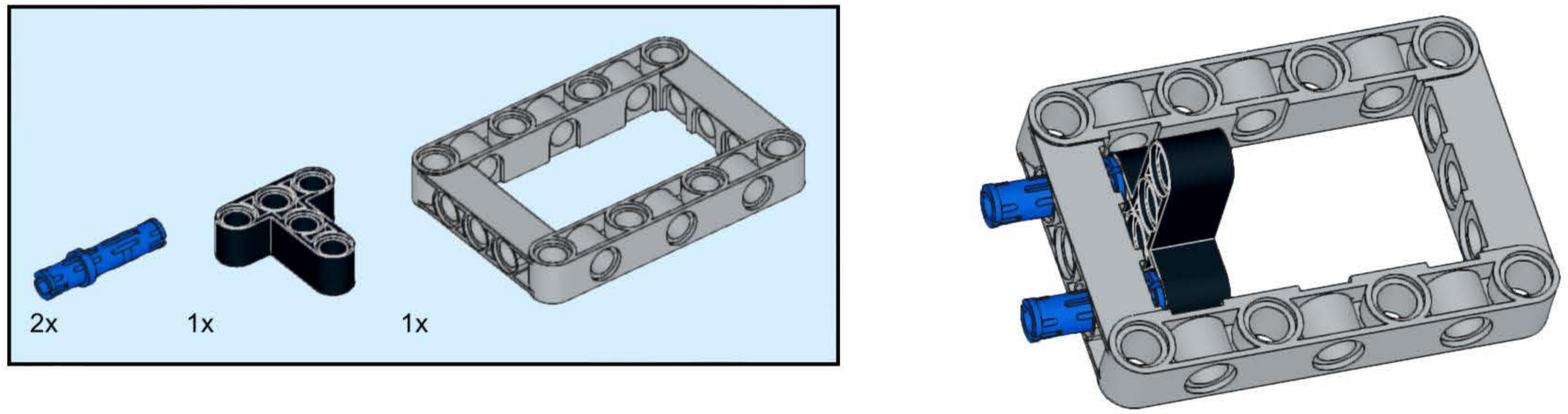


# 15

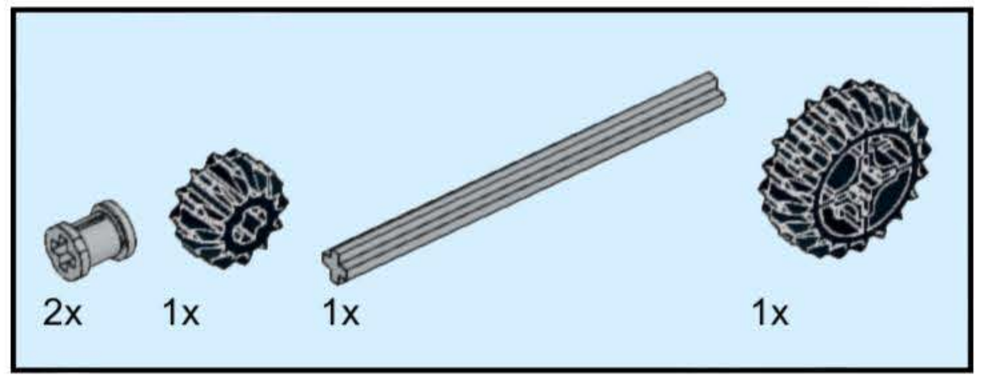


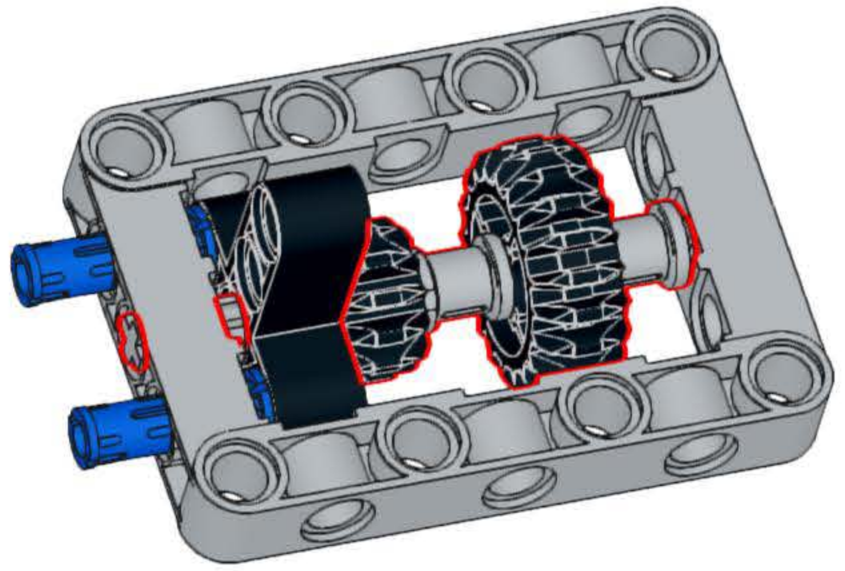

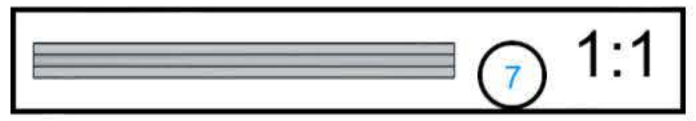


# 16

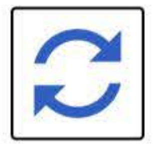

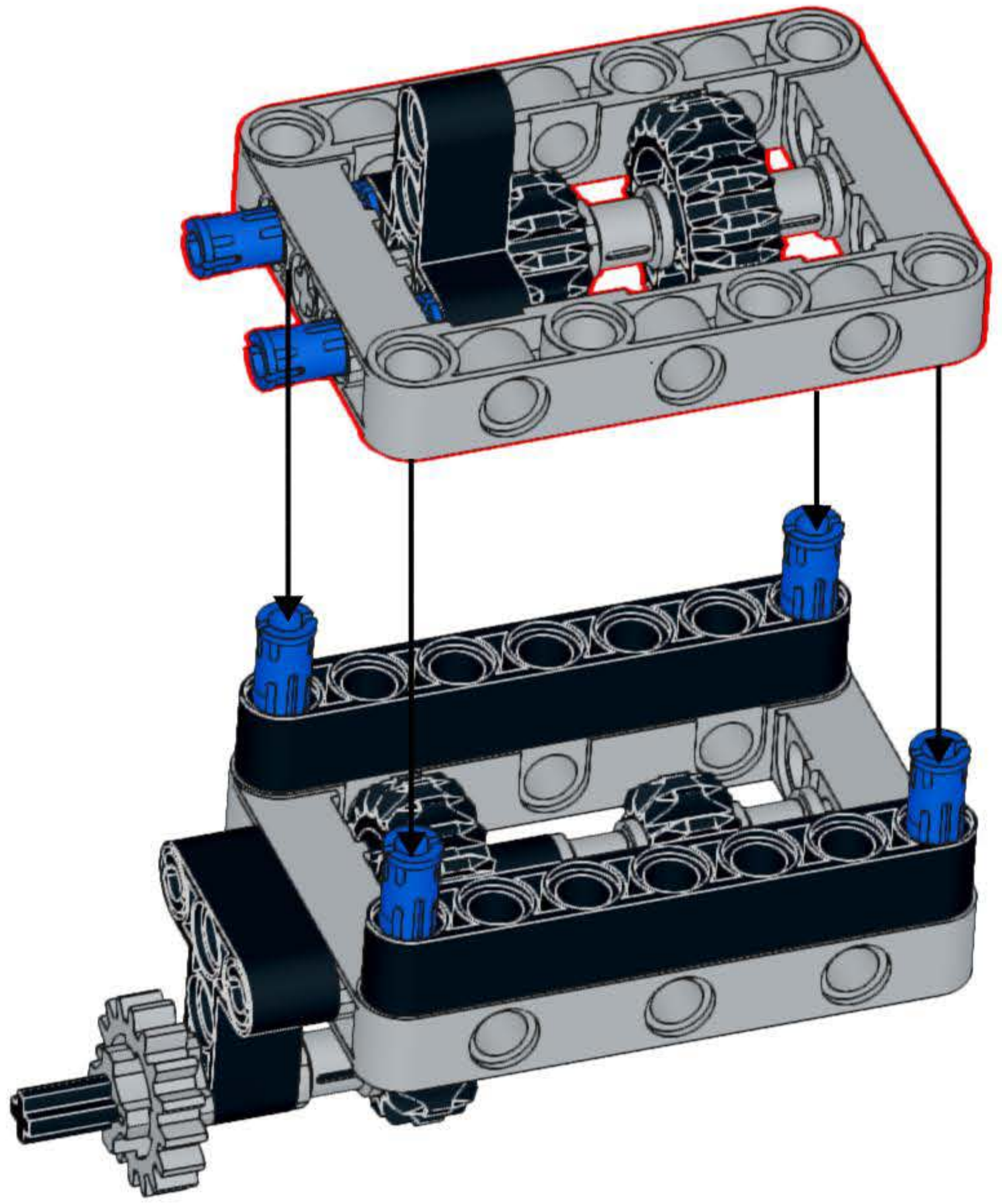

# 17

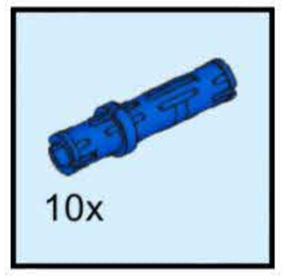


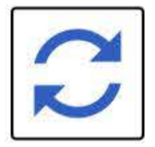

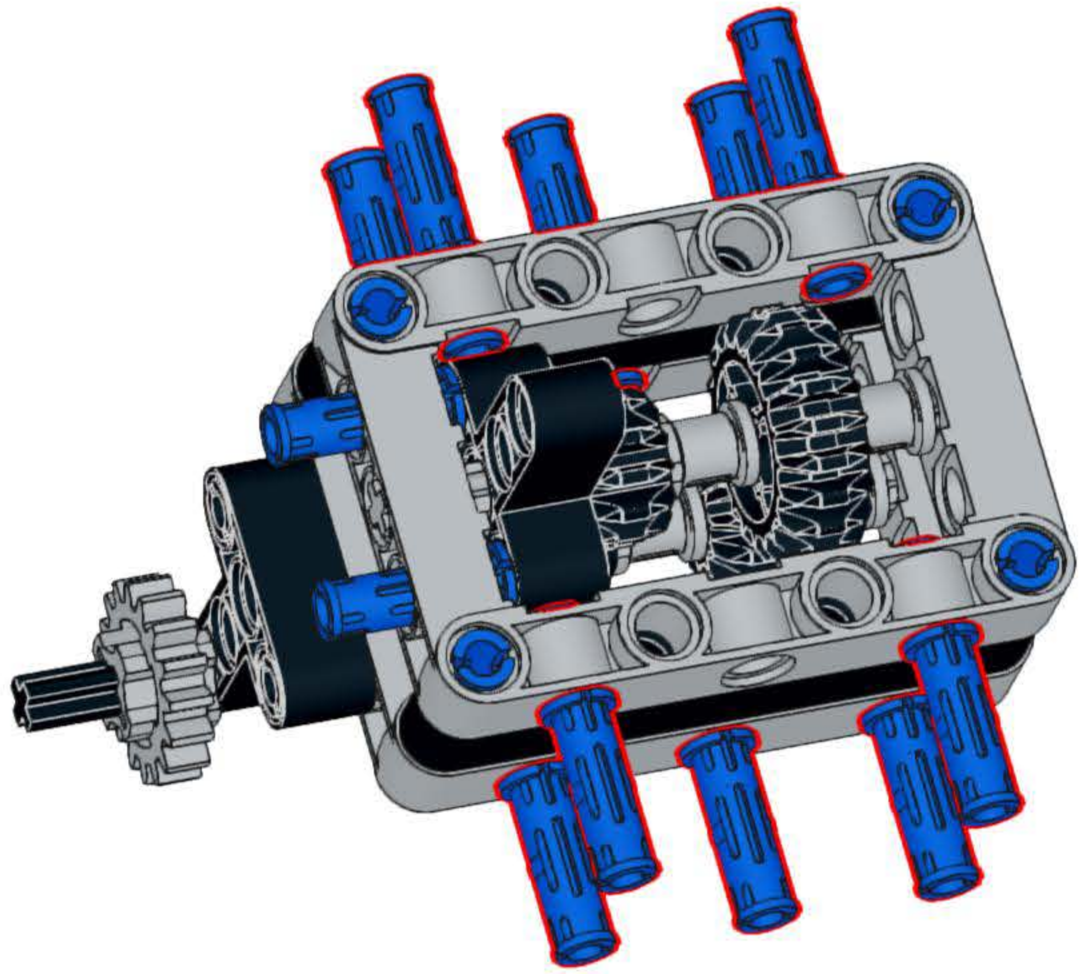

18

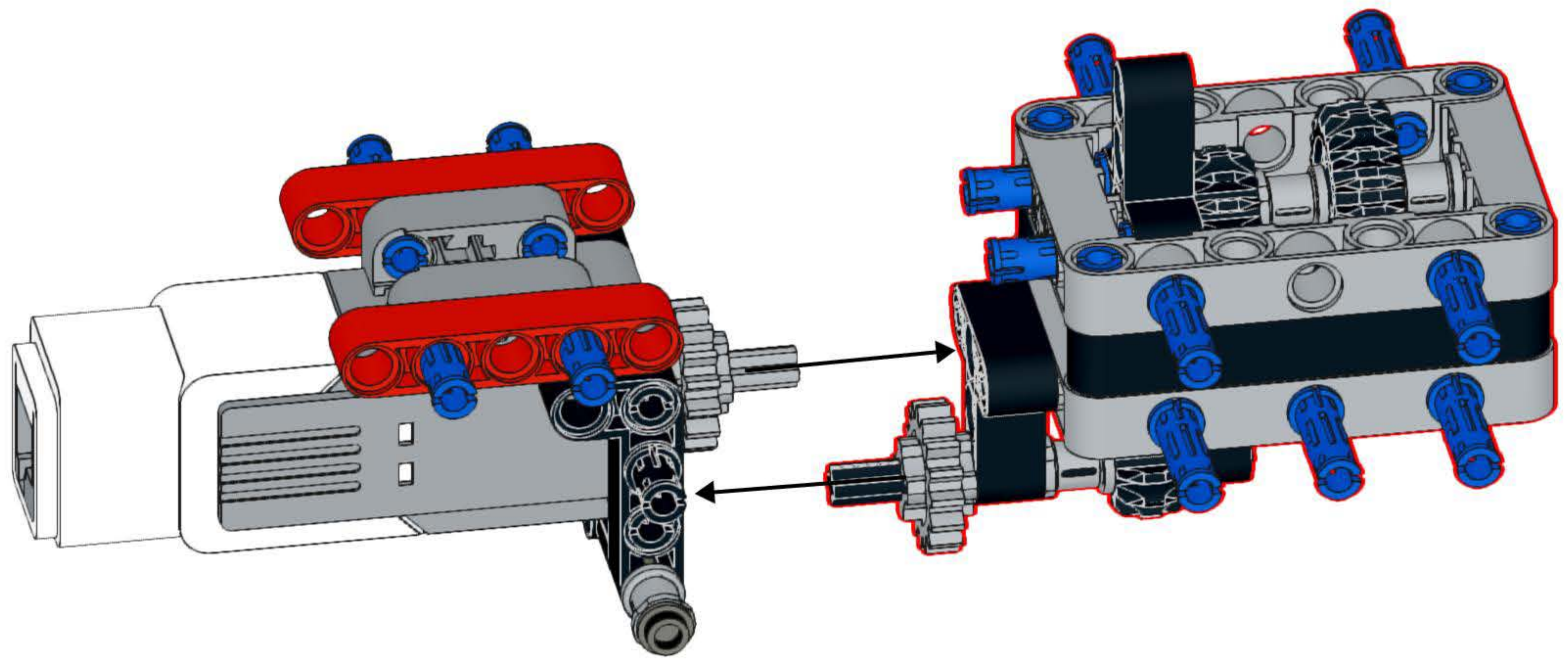

19

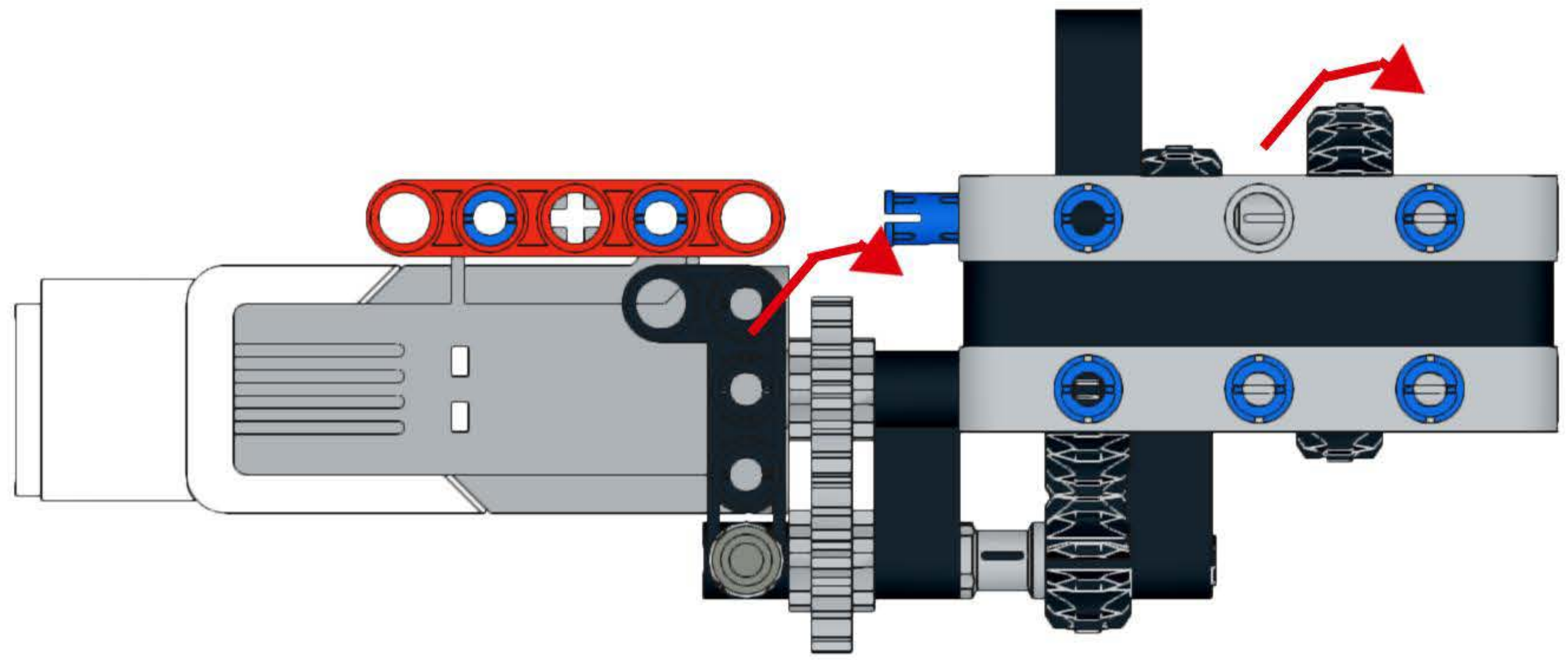

# 20

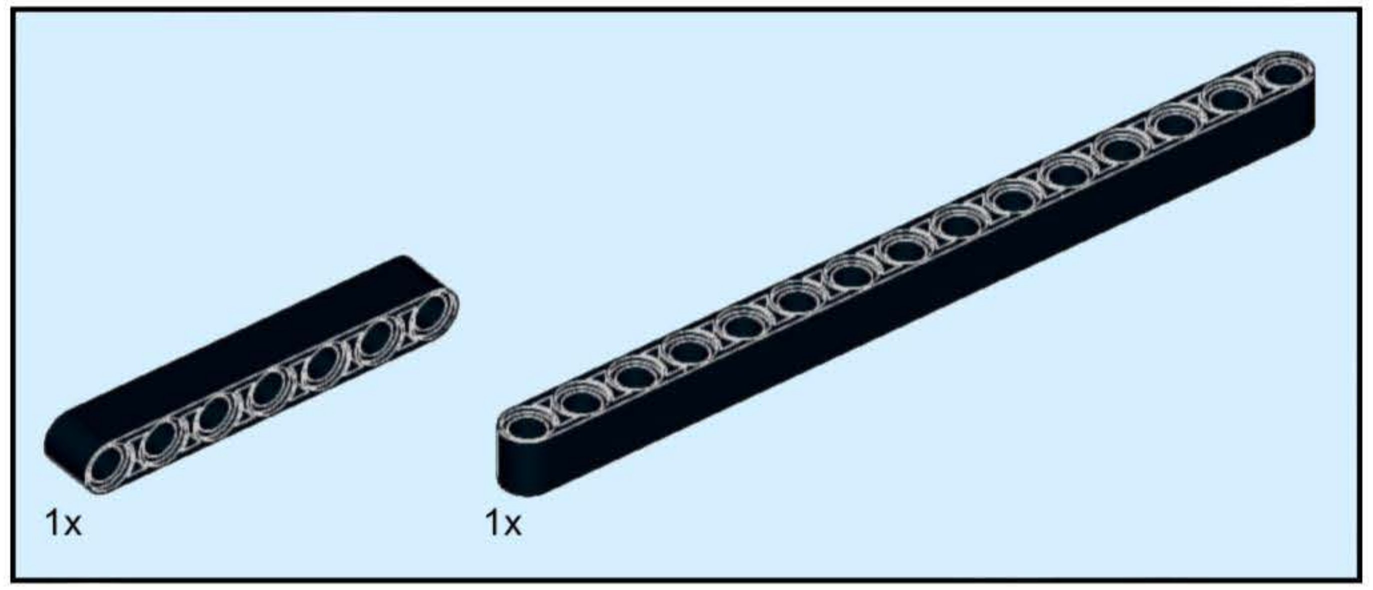


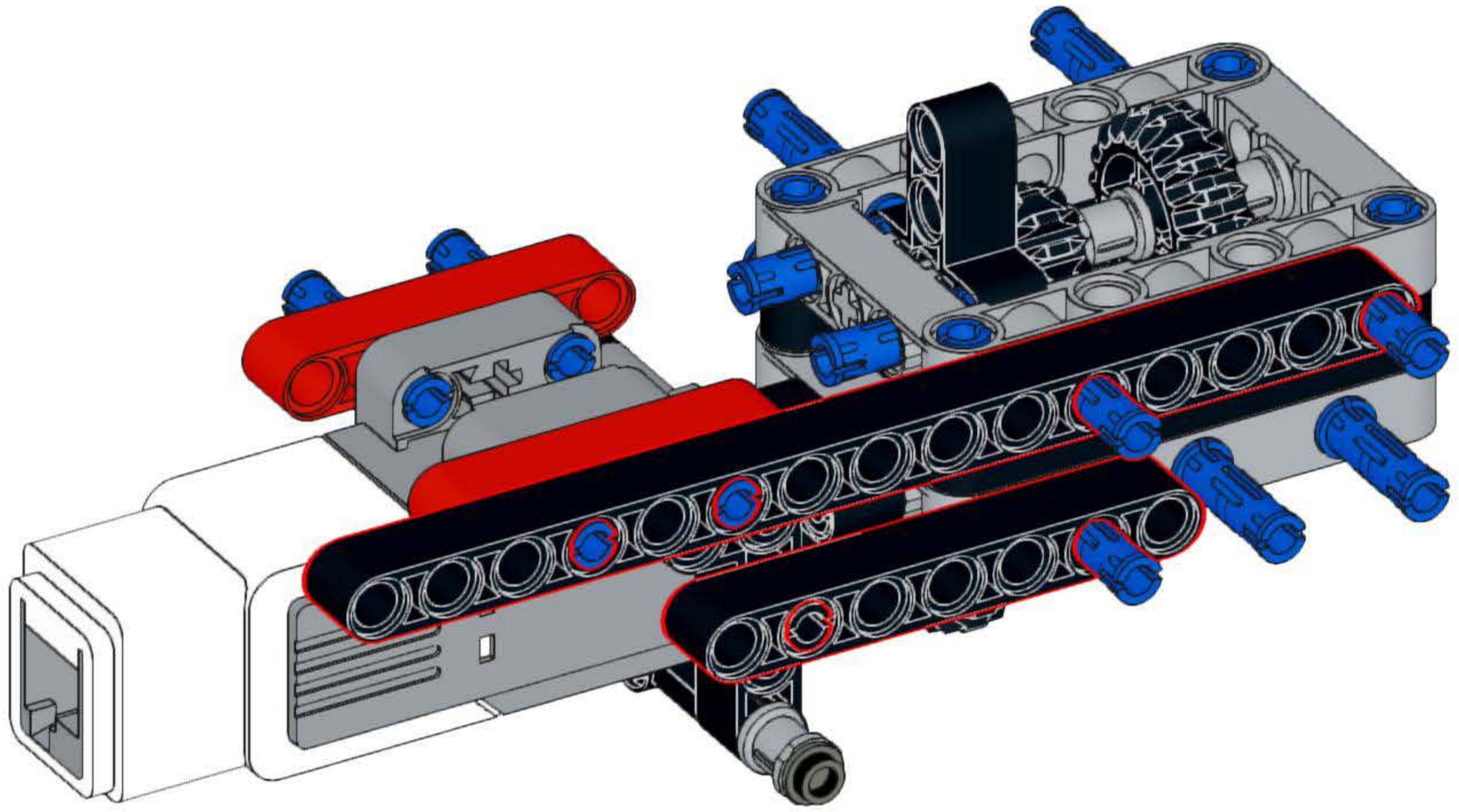

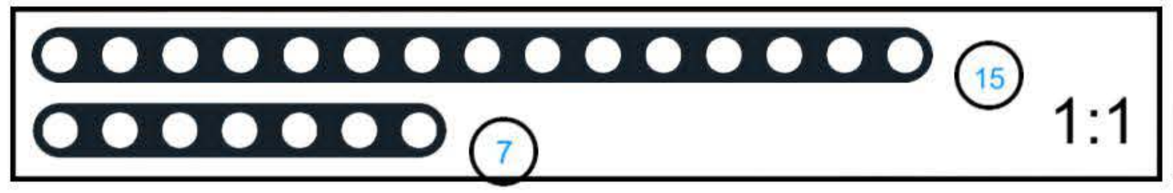


# 21

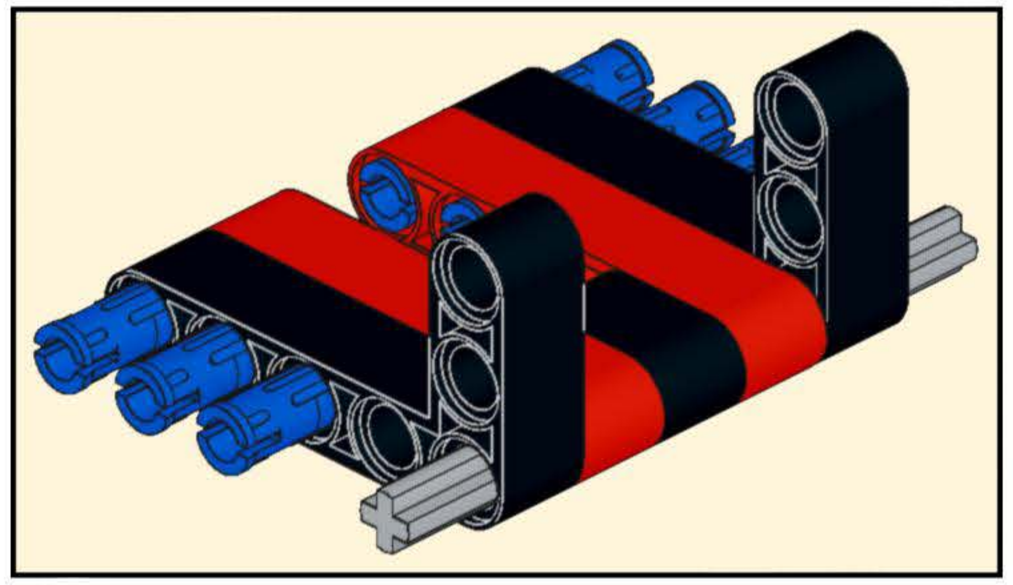

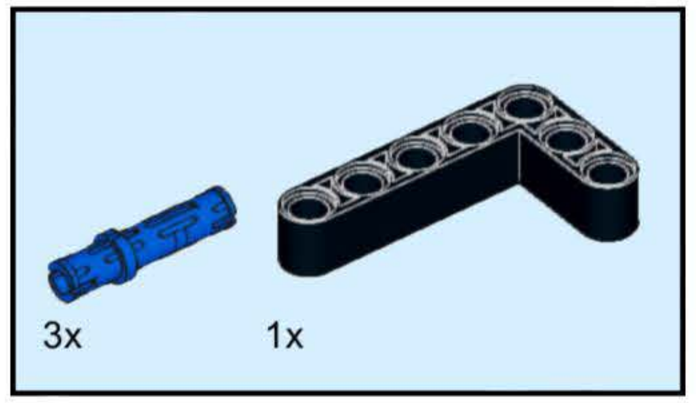


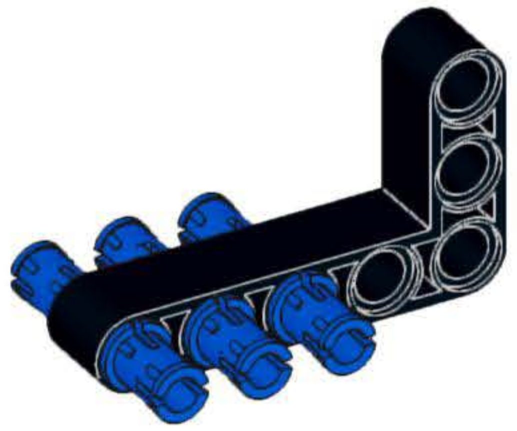

## 22

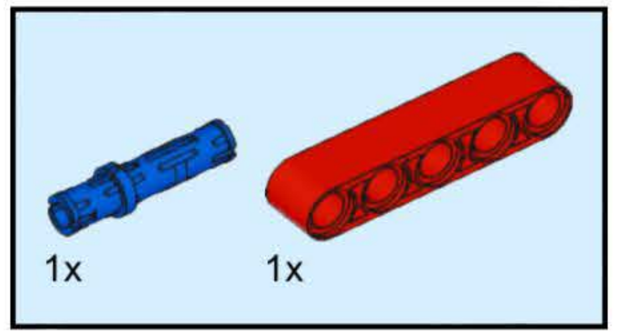


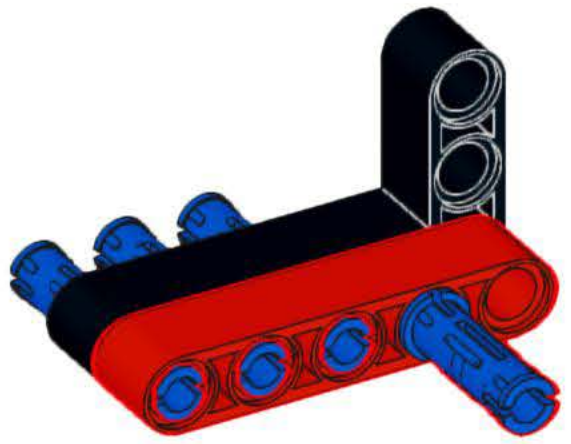

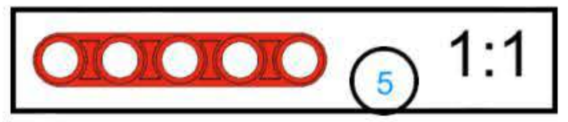


## 23

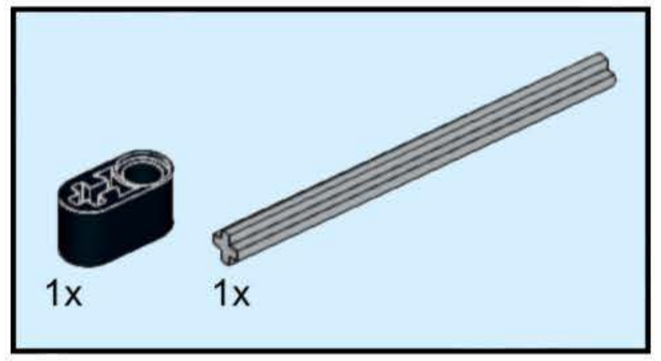


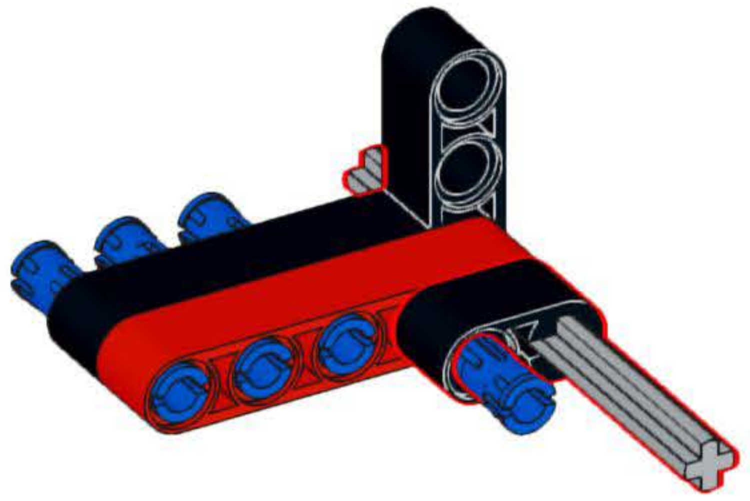

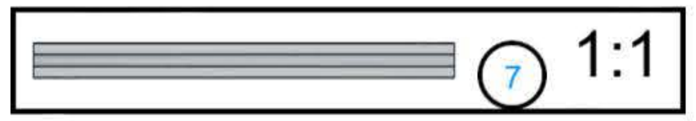


## 24

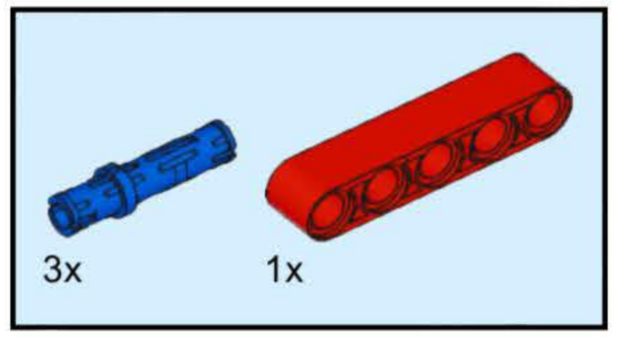


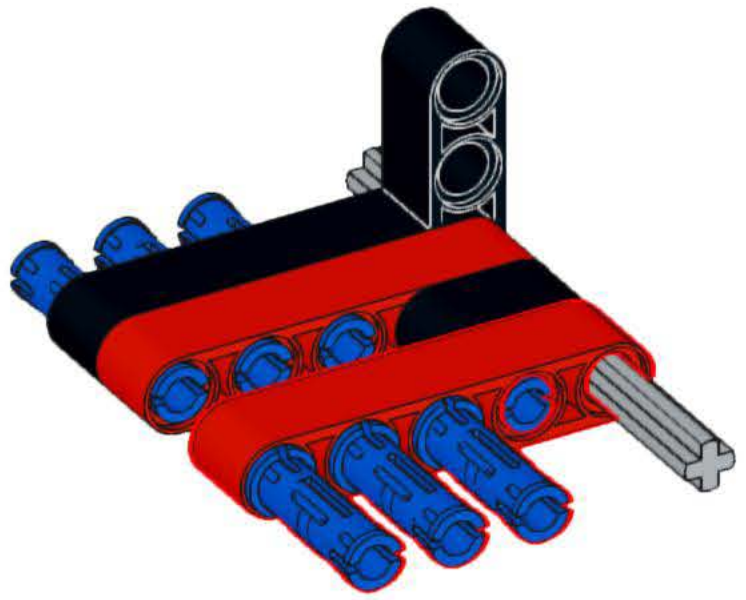

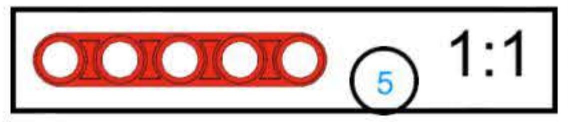


## 25

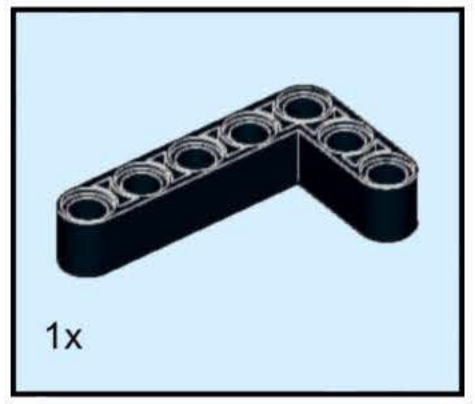


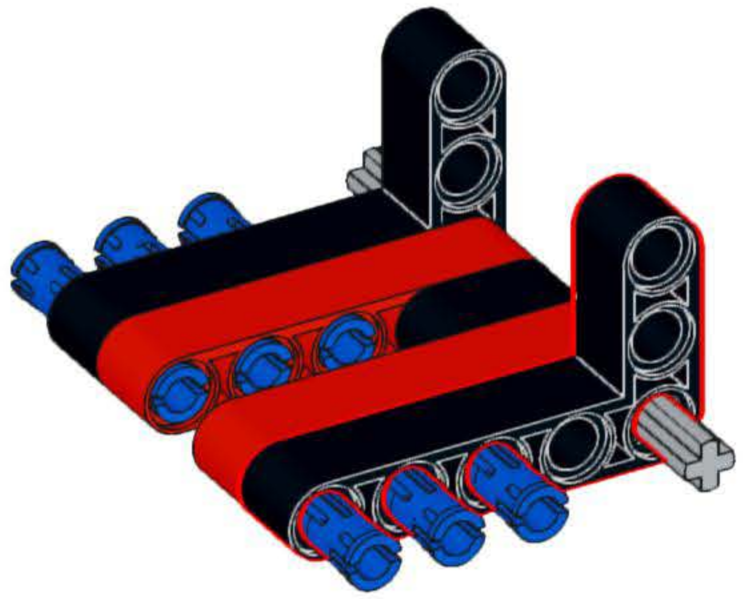

## 26

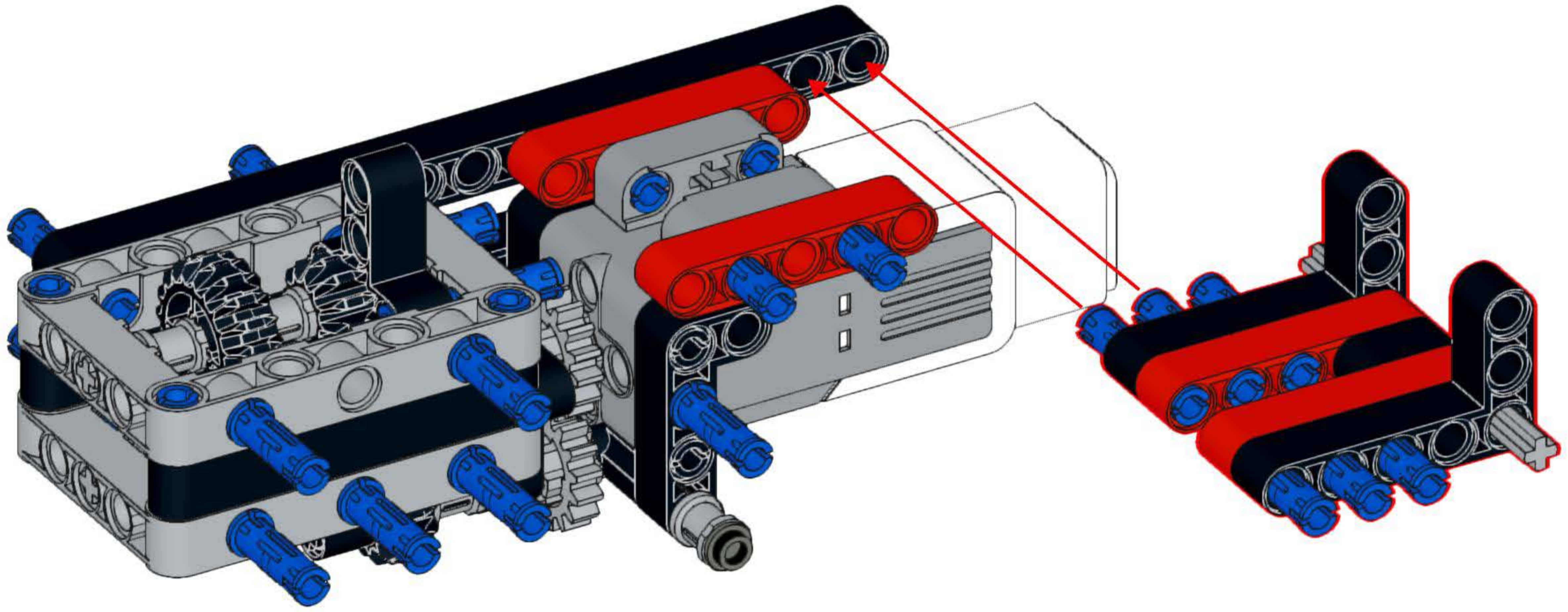

## 27

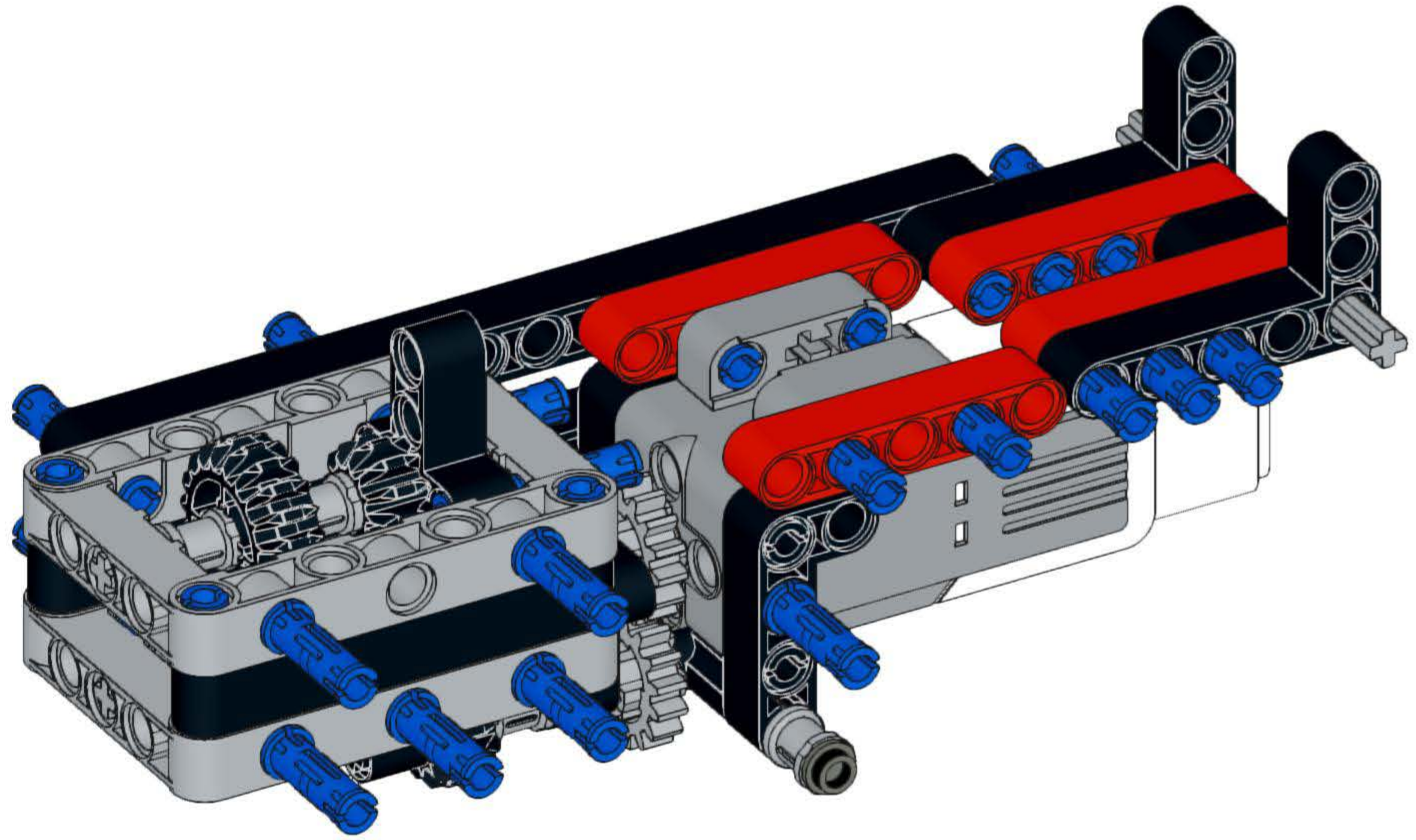

# 28

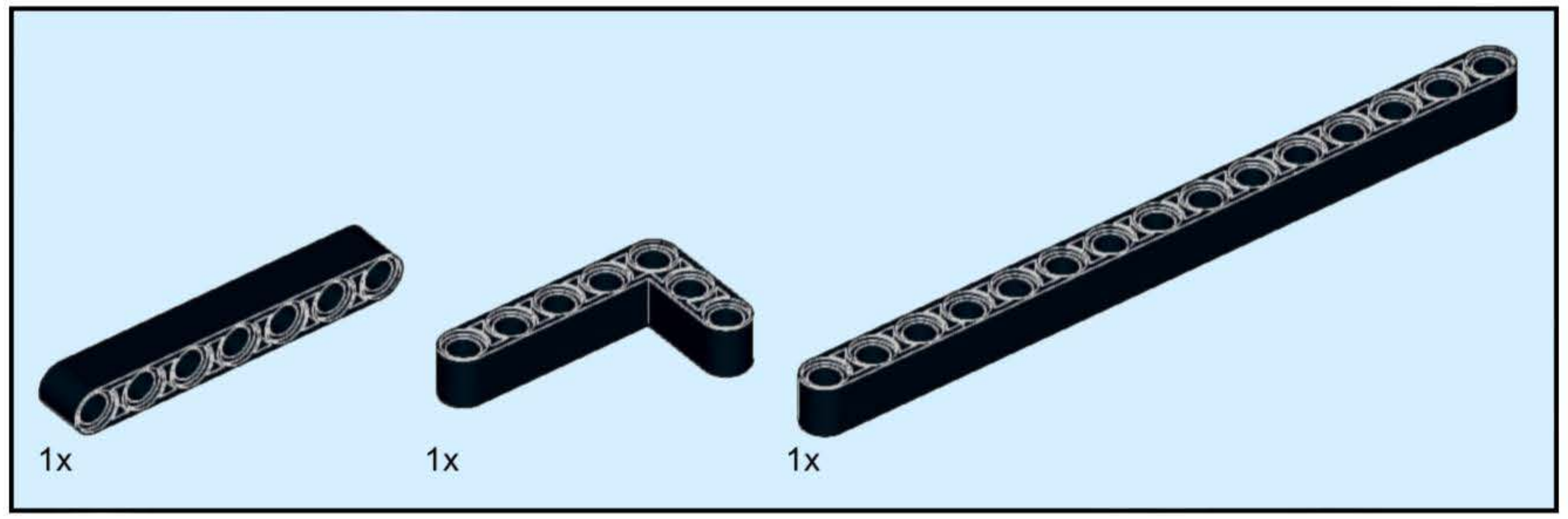


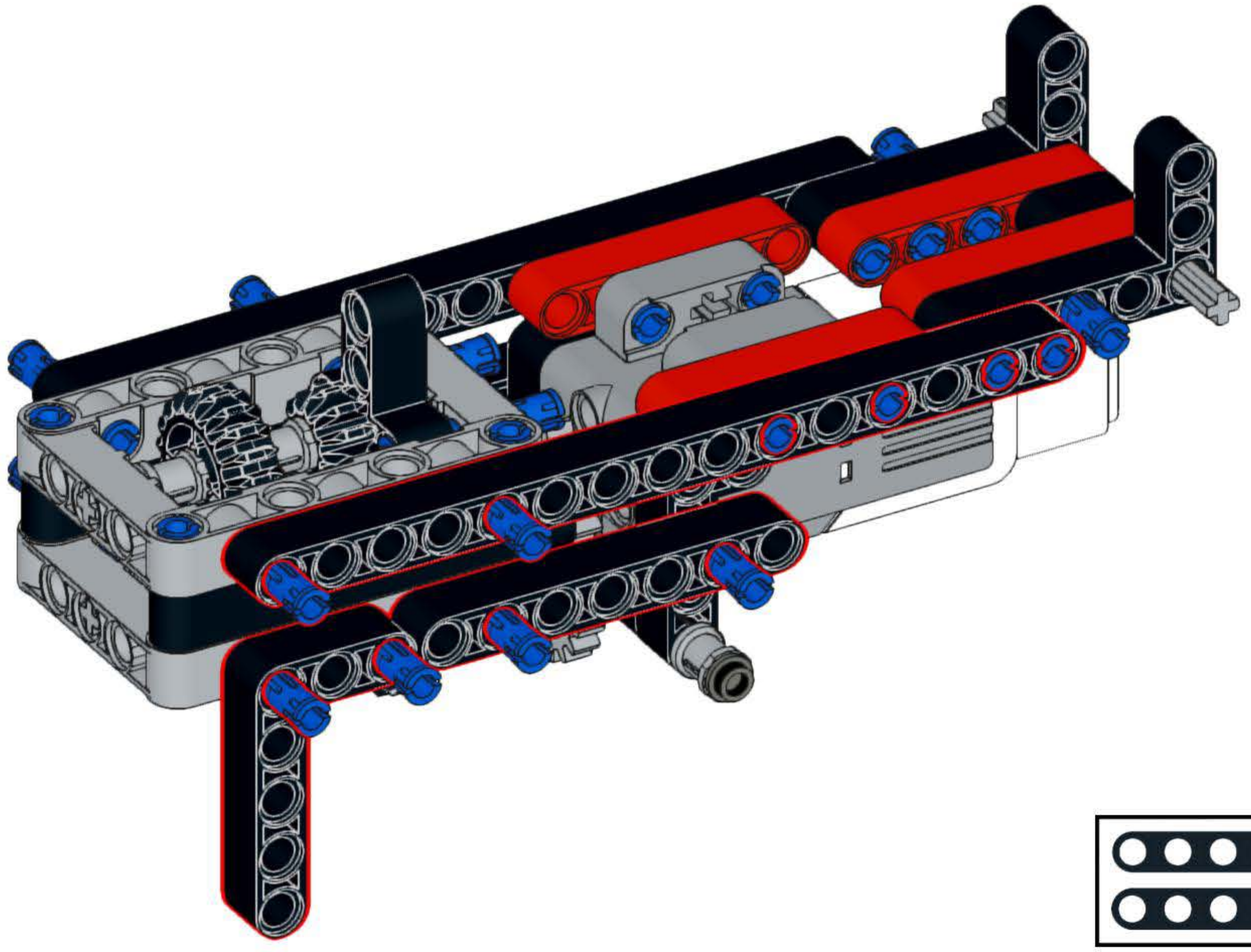

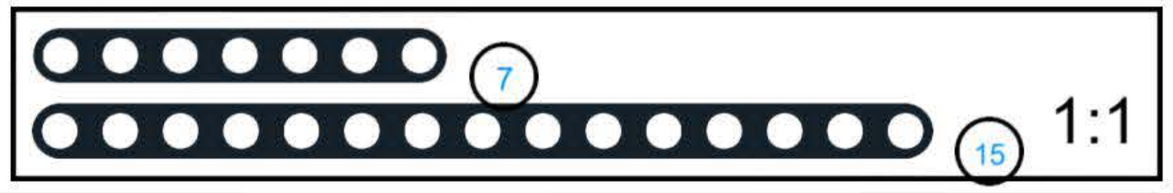


# 29

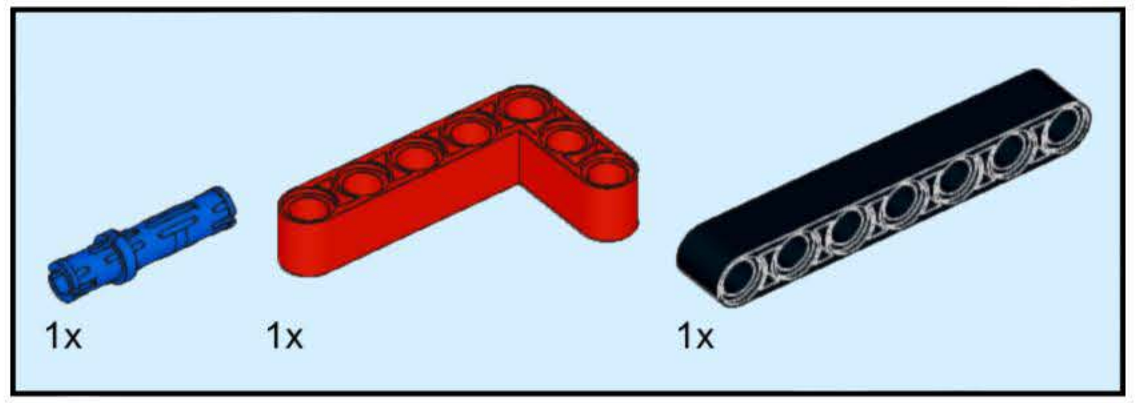


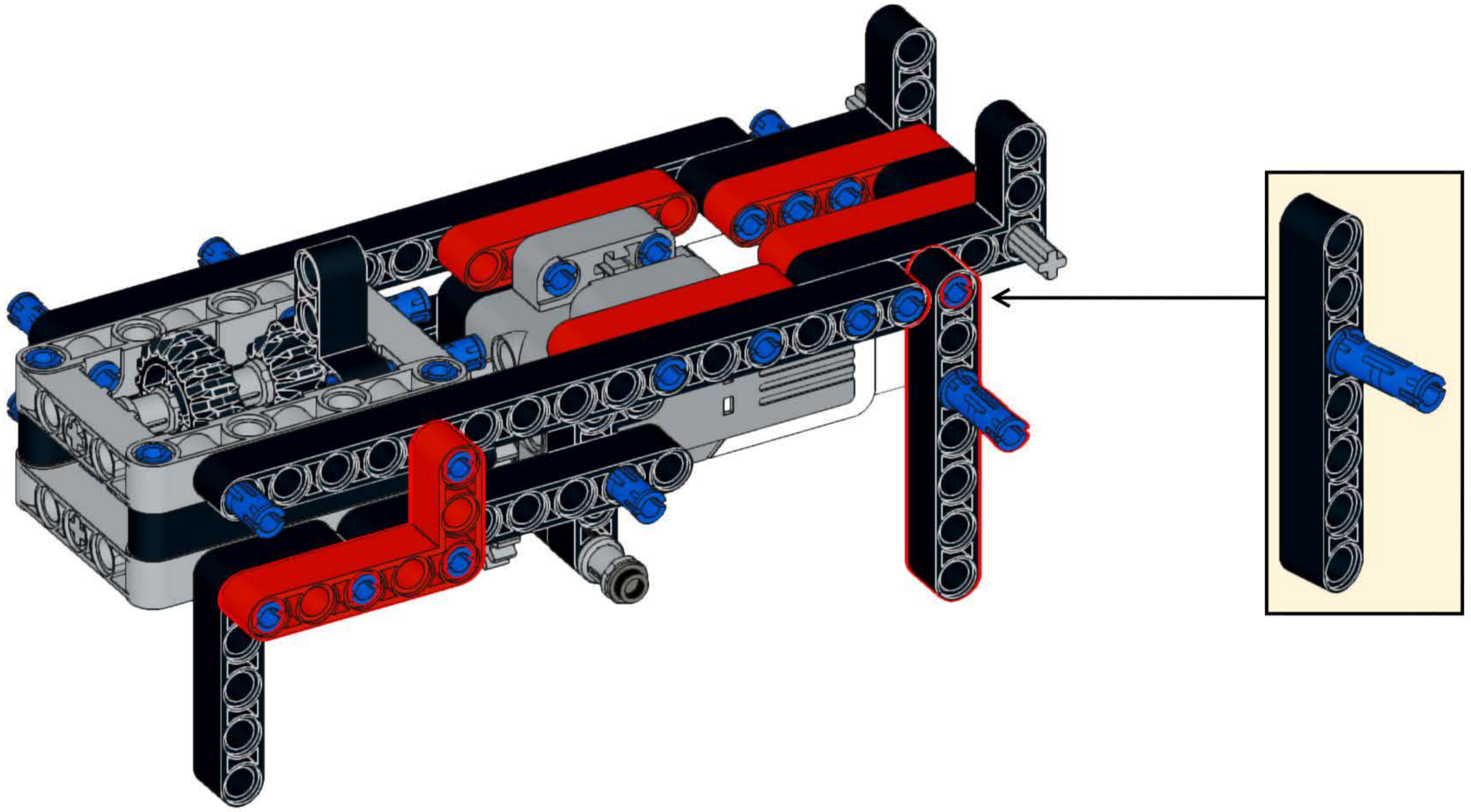

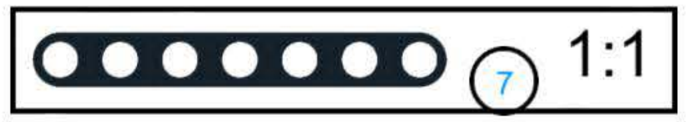

# 30

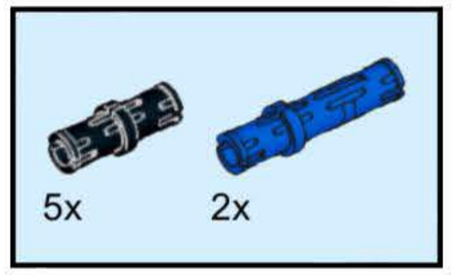


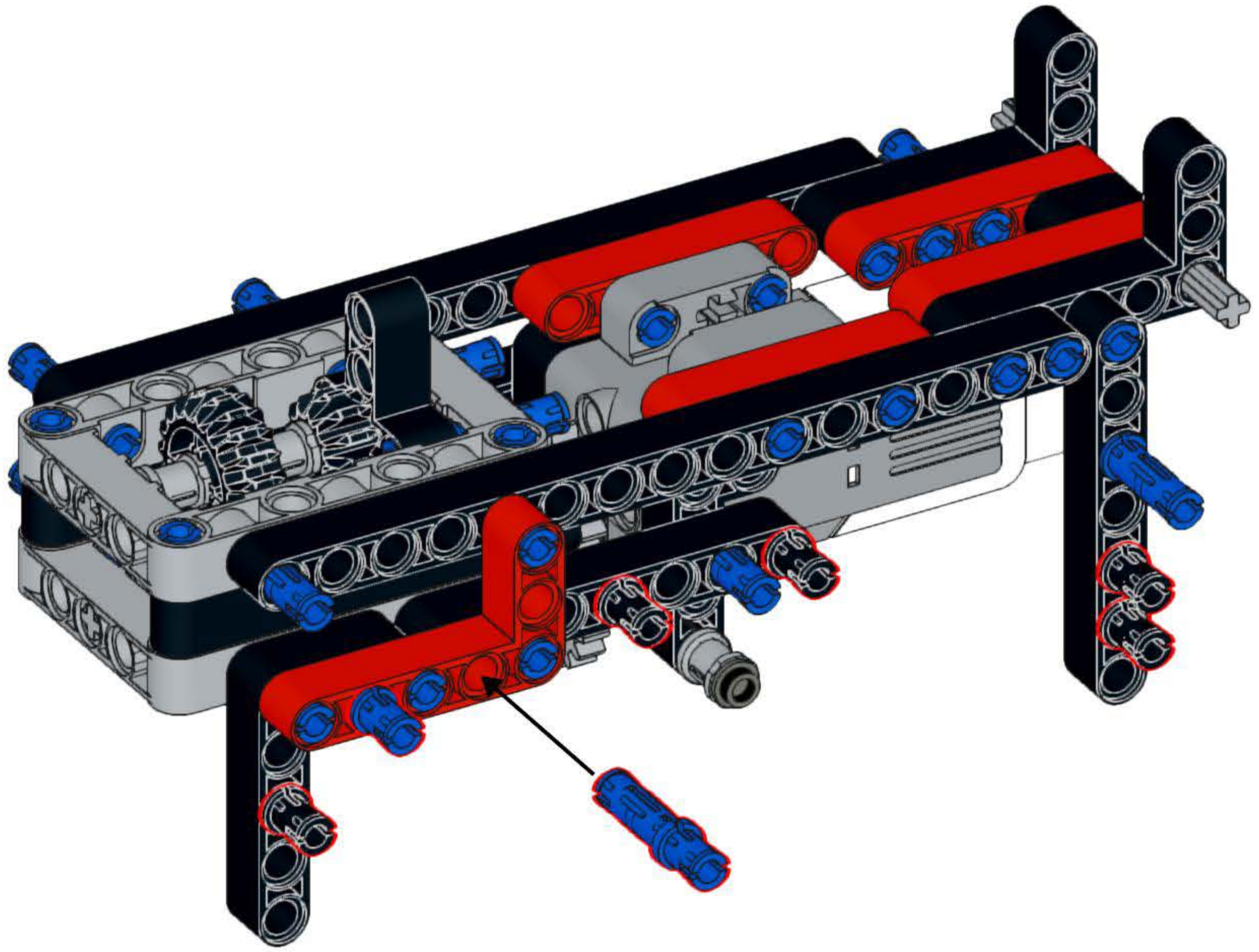

# 31

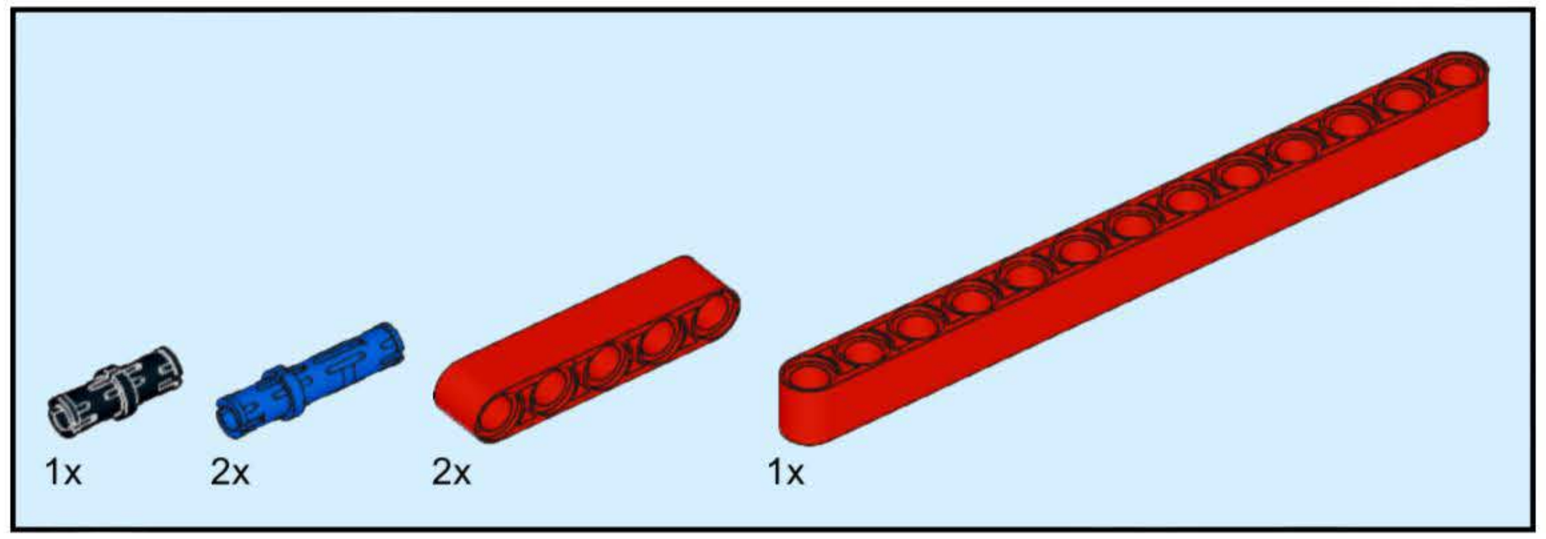


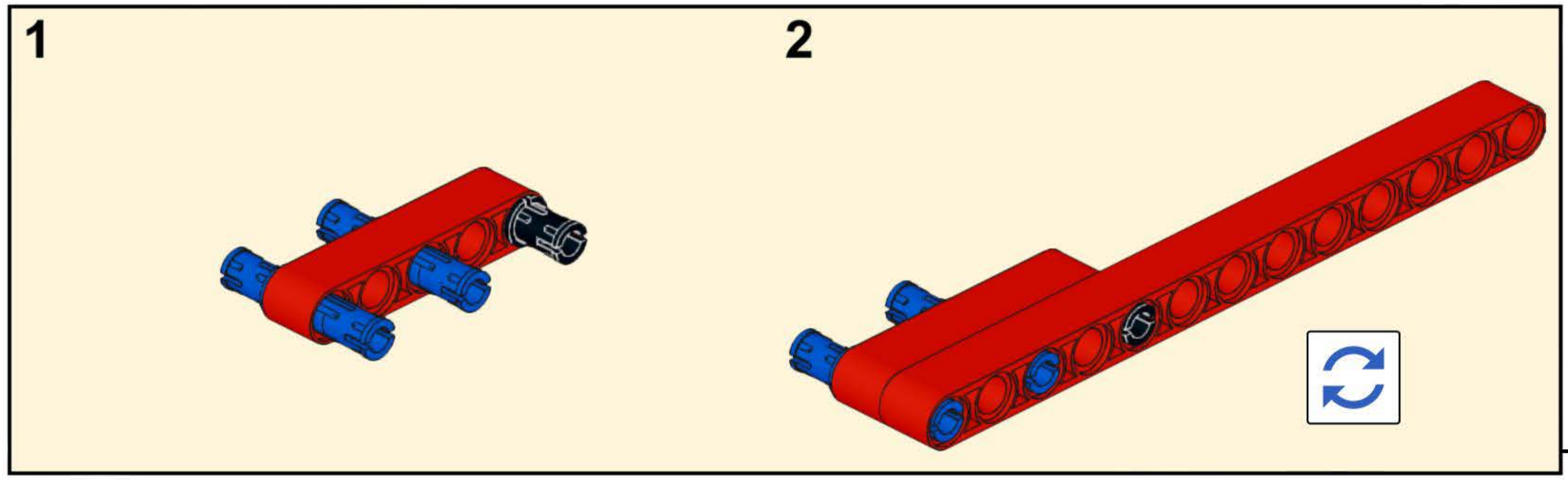


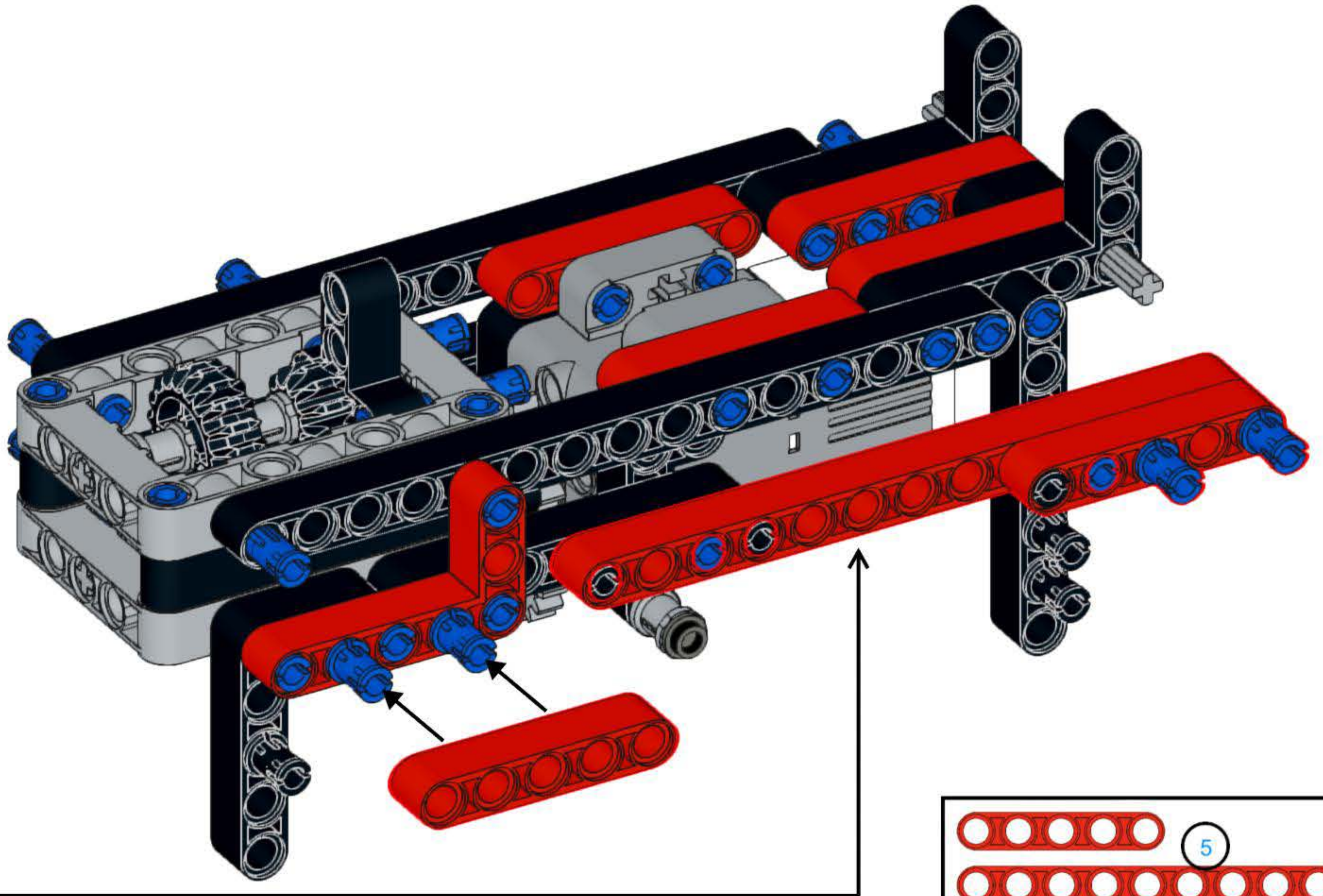

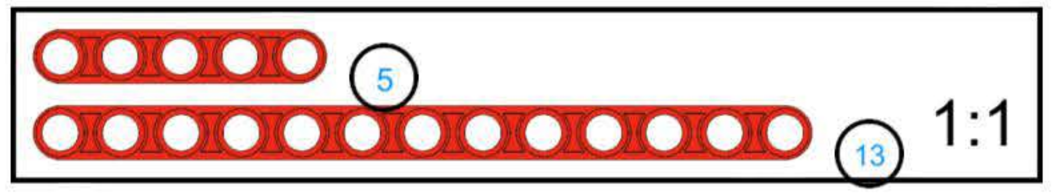

32

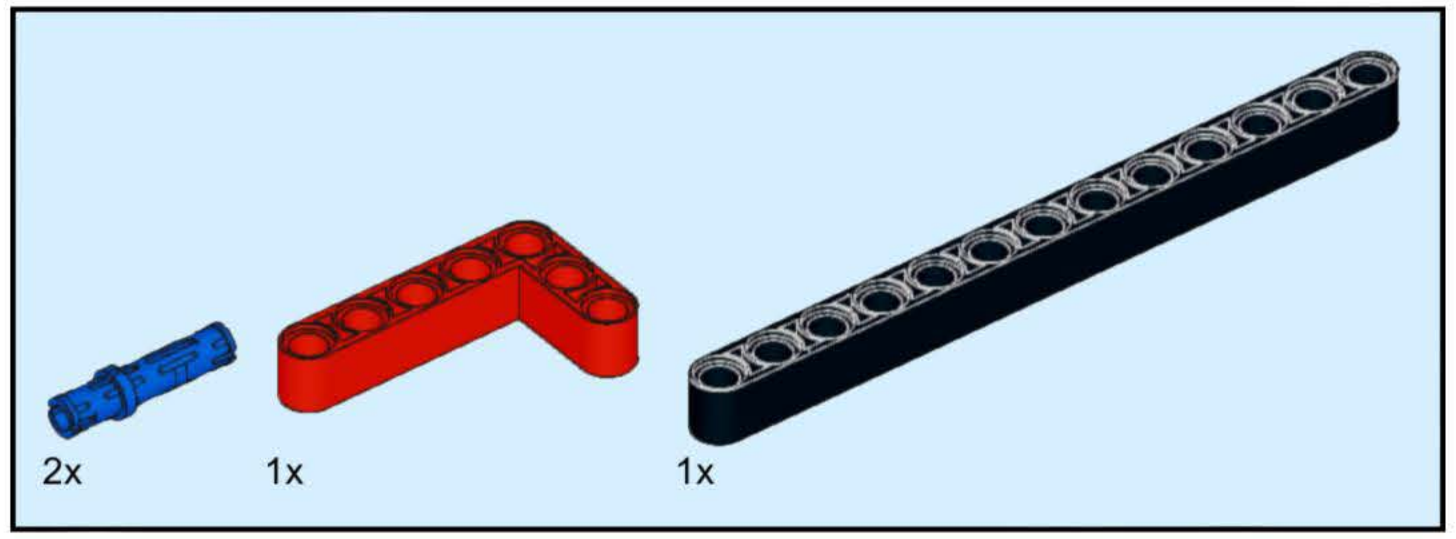


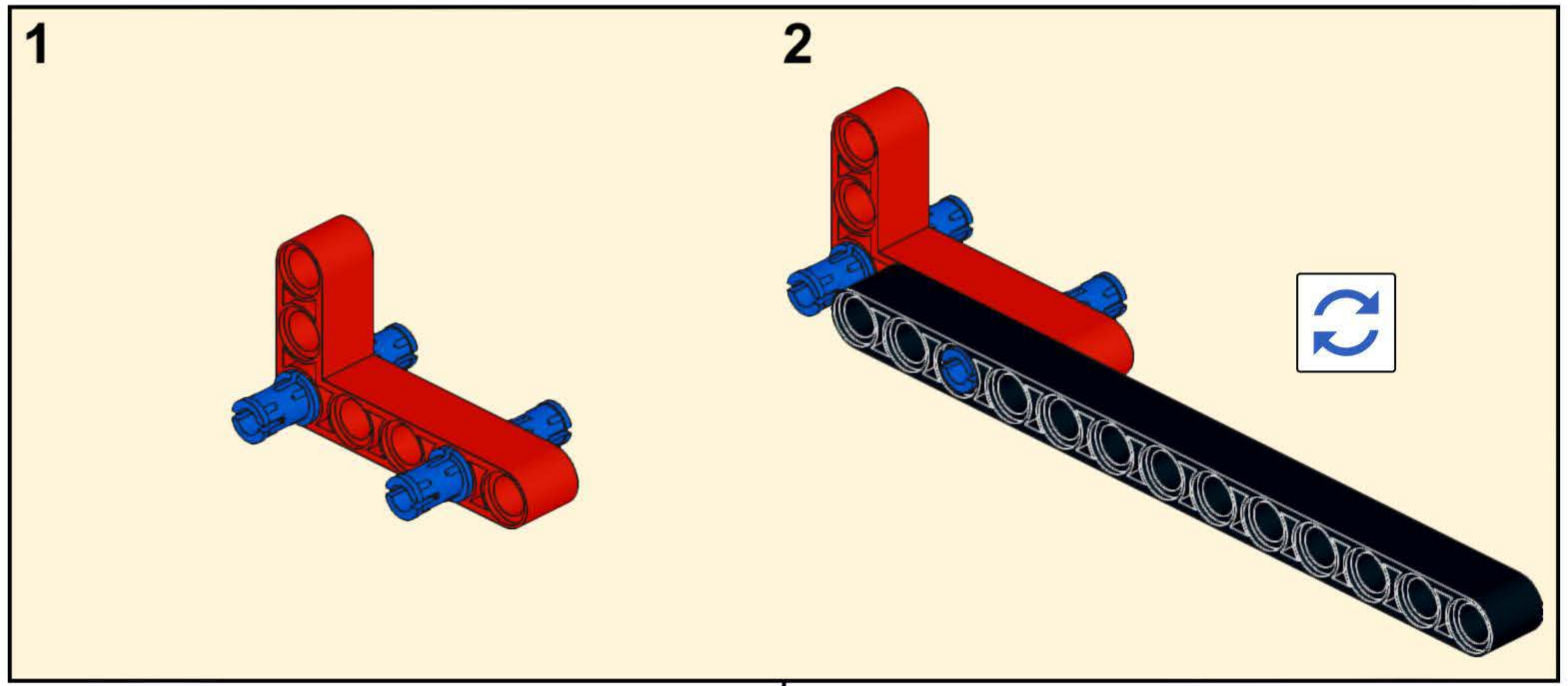


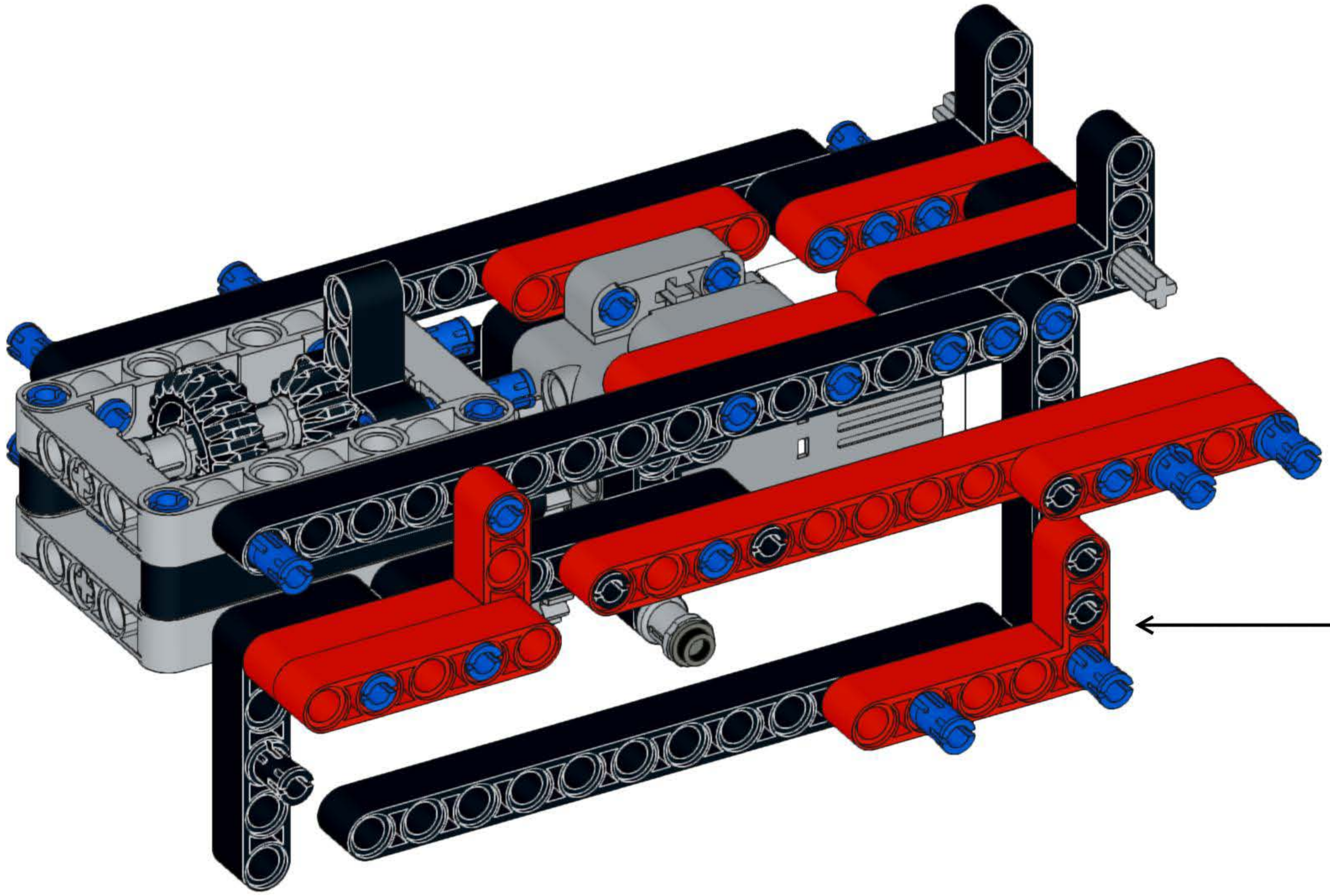

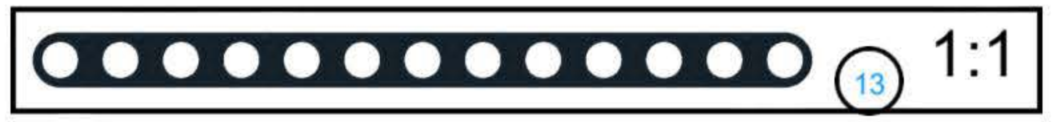

**33**

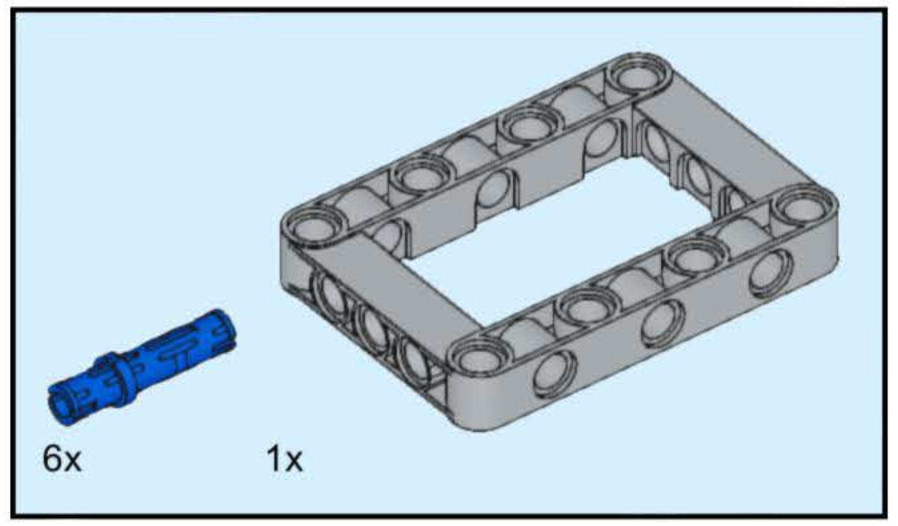


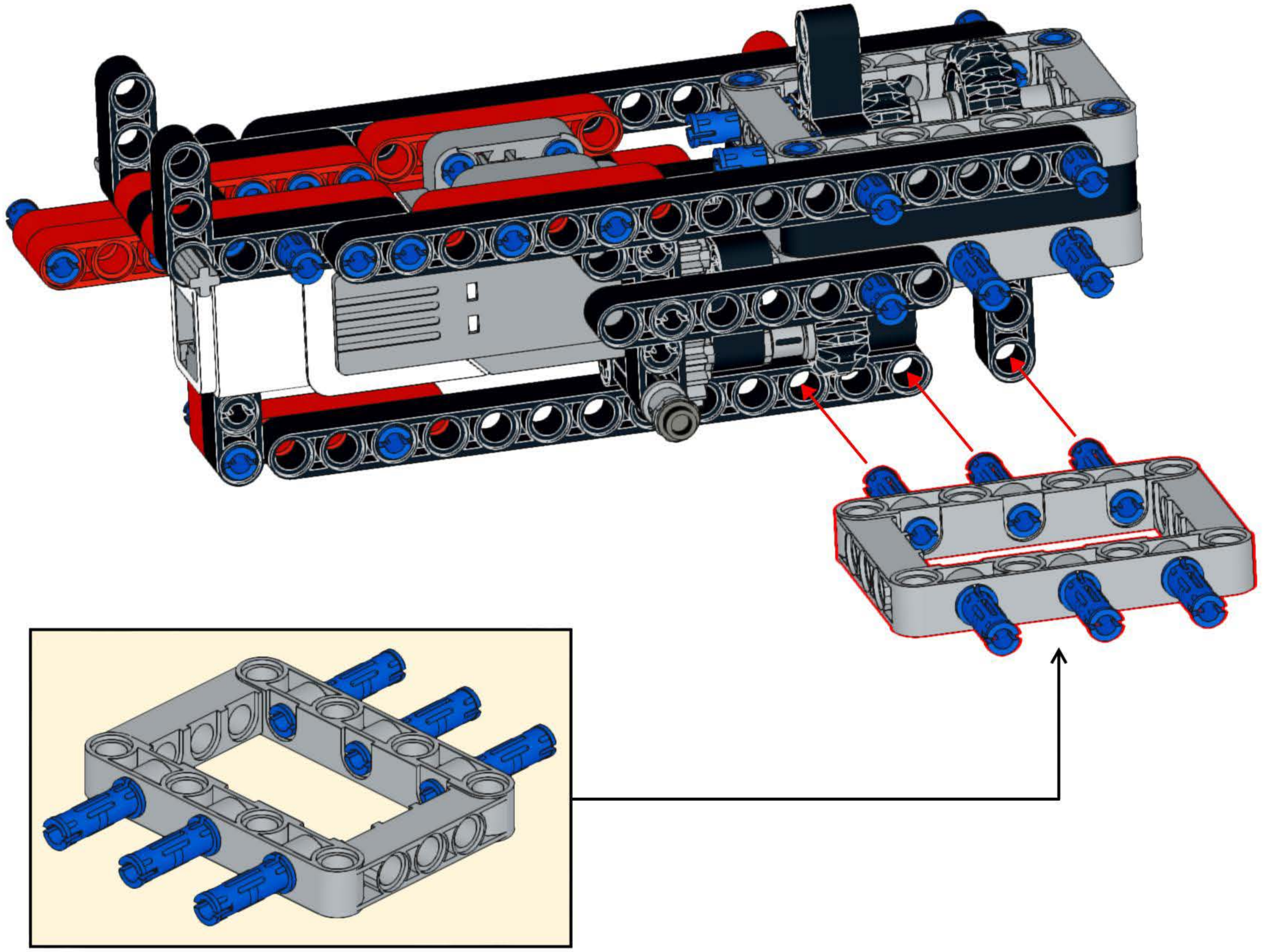

## 34

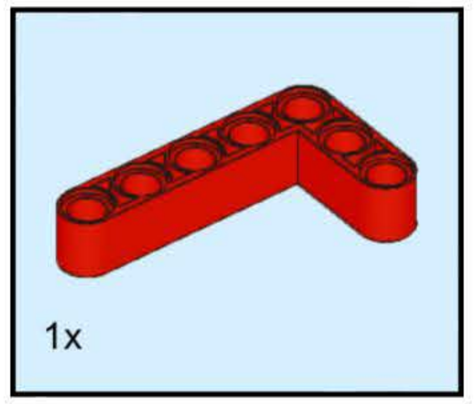


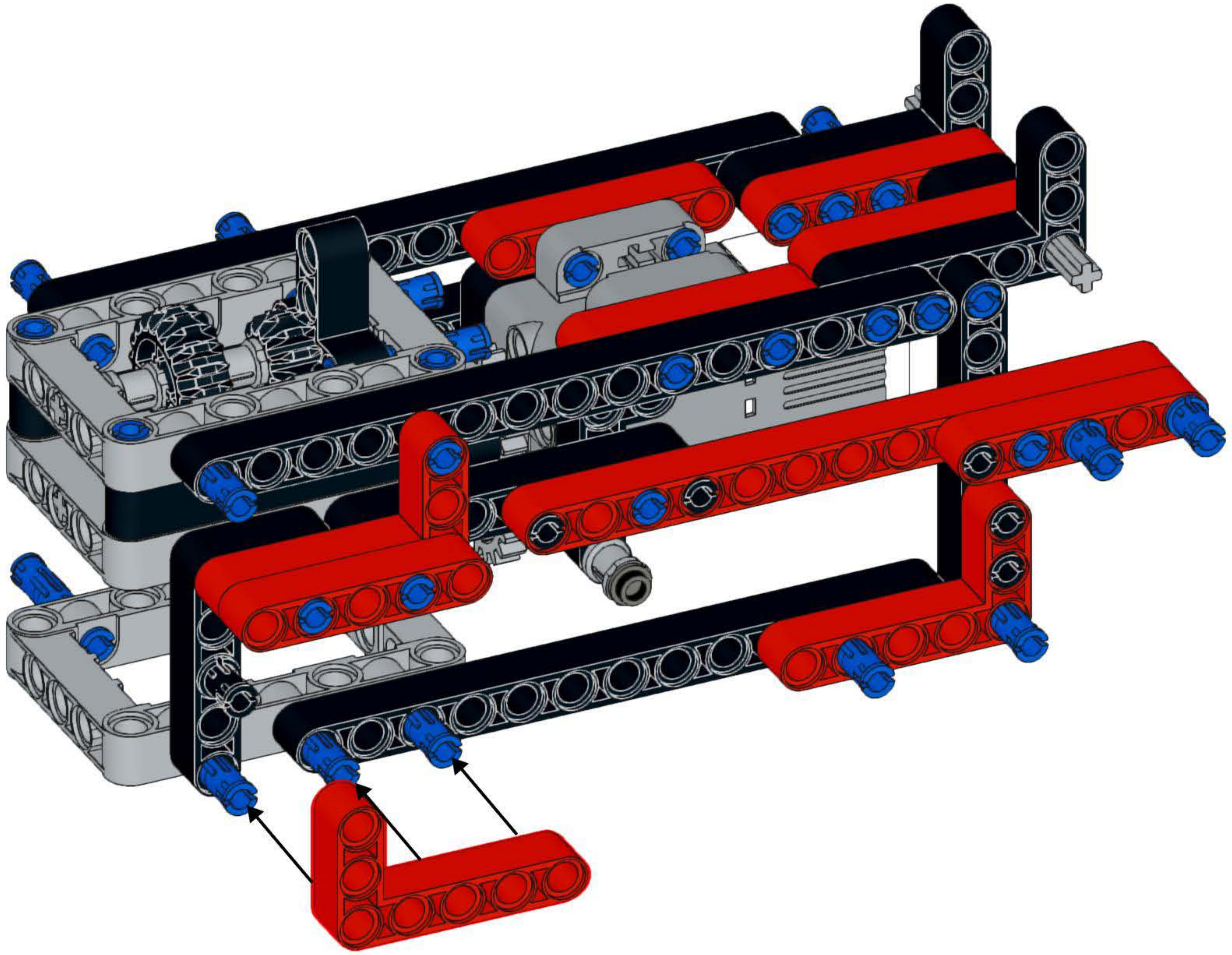

## 35

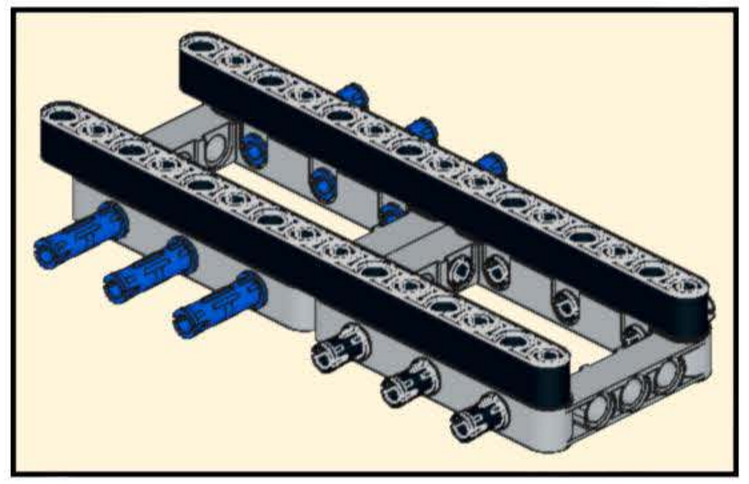

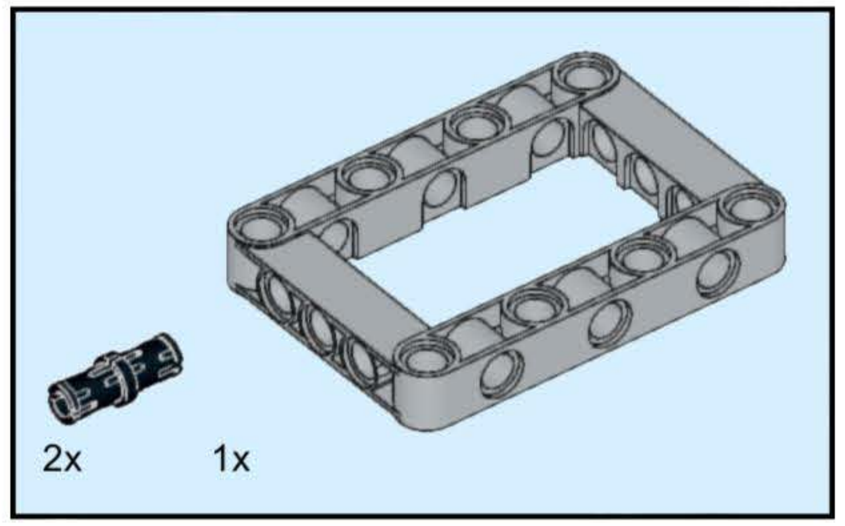


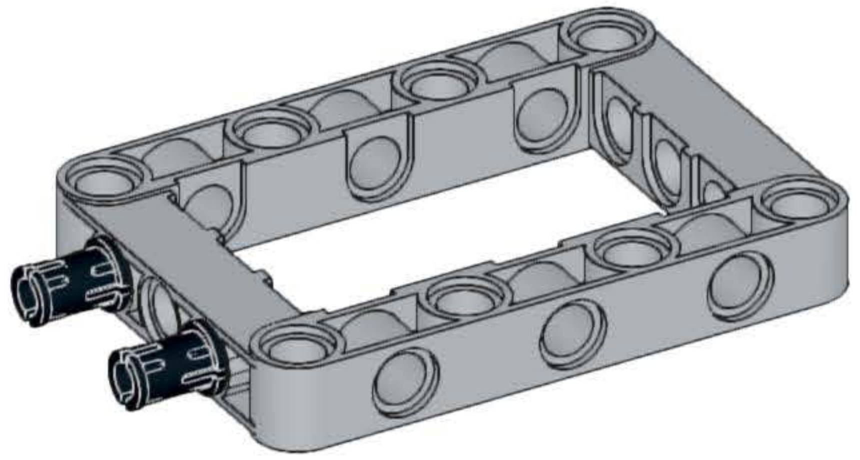

**36**

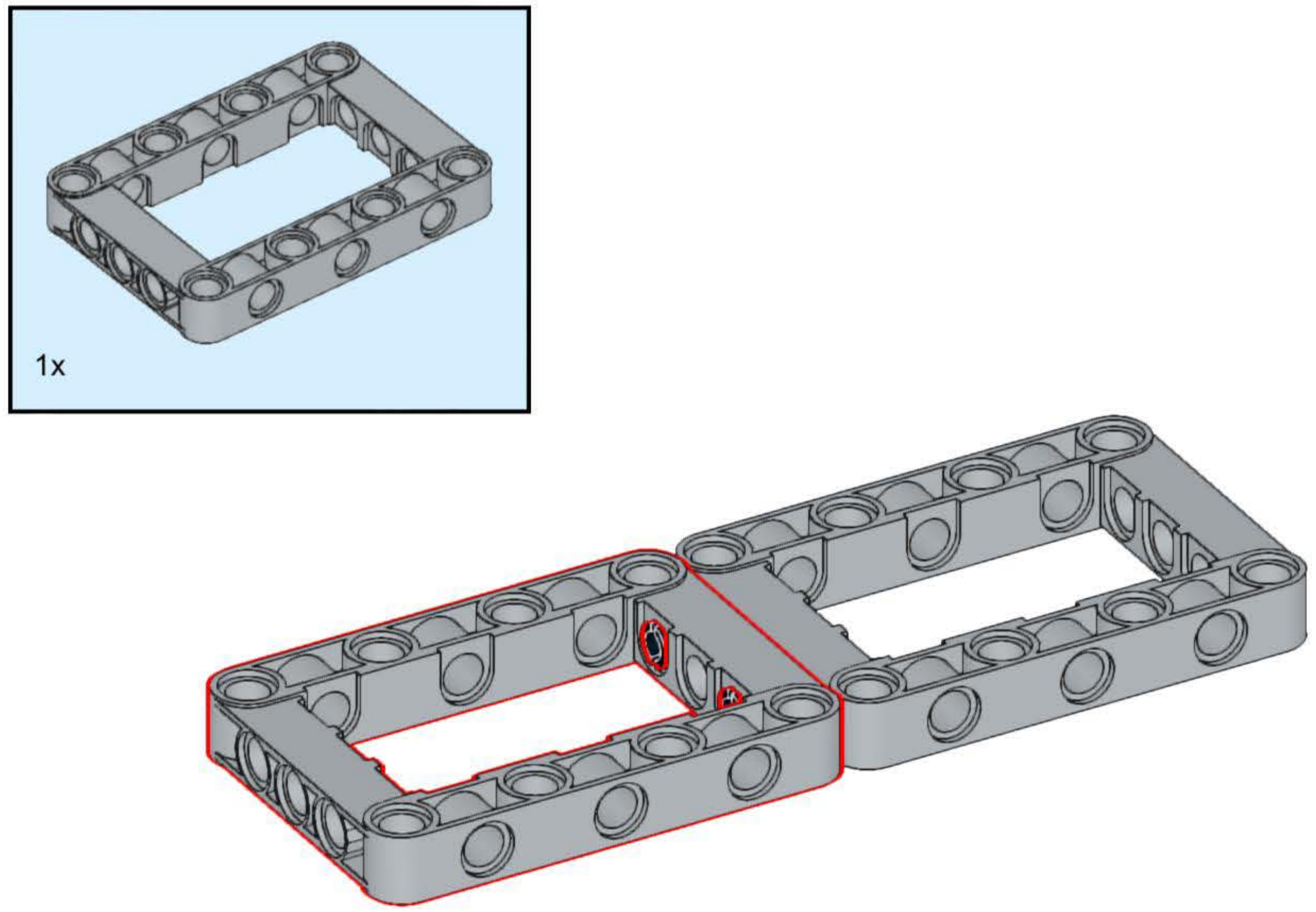


**37**

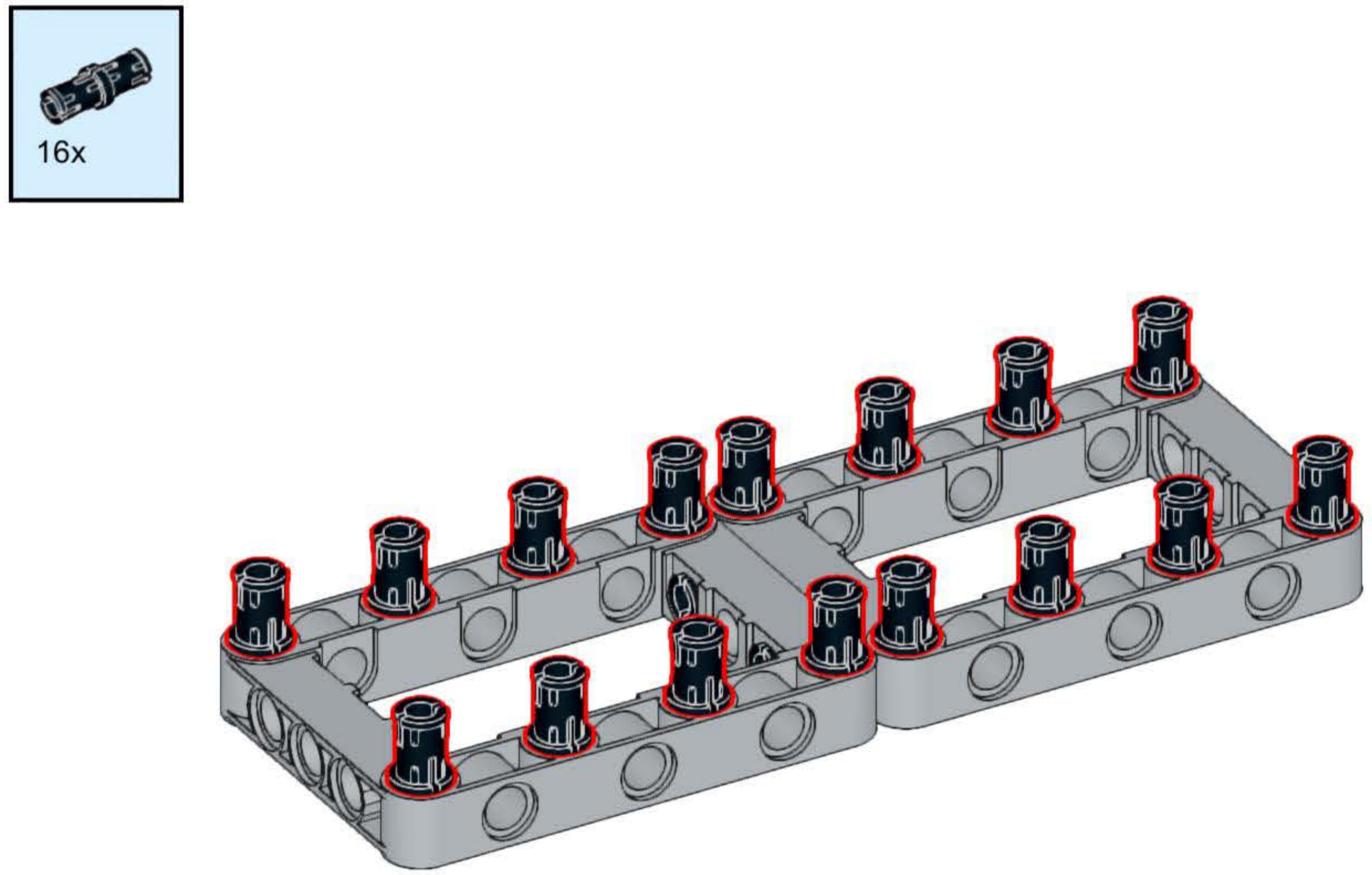


**38**

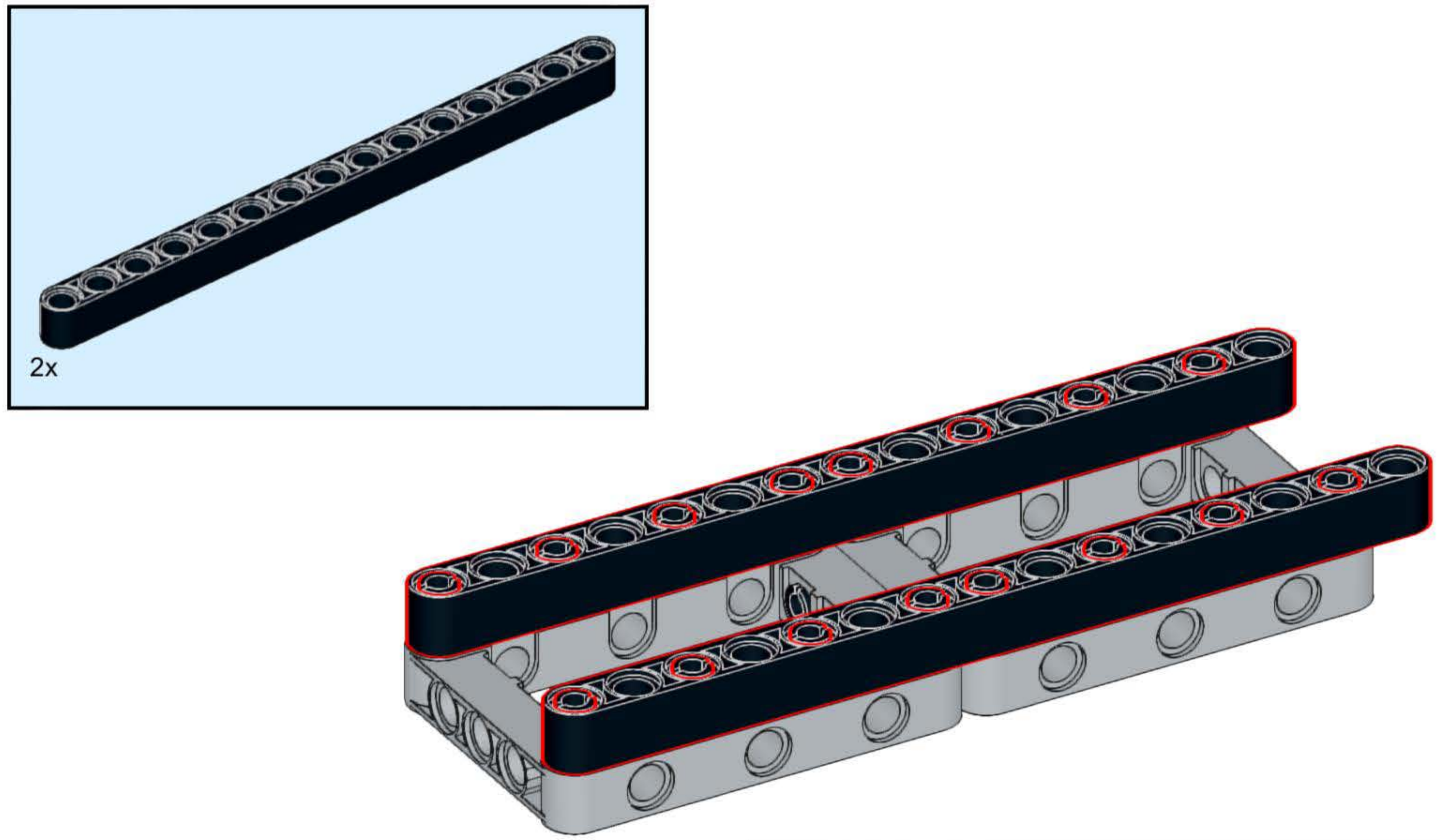


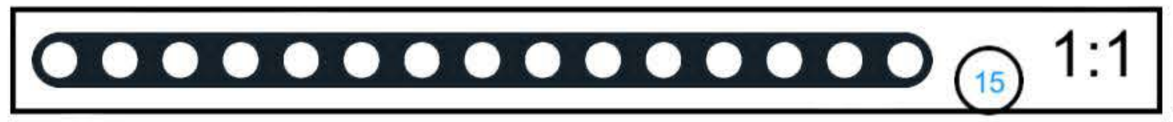


**39**

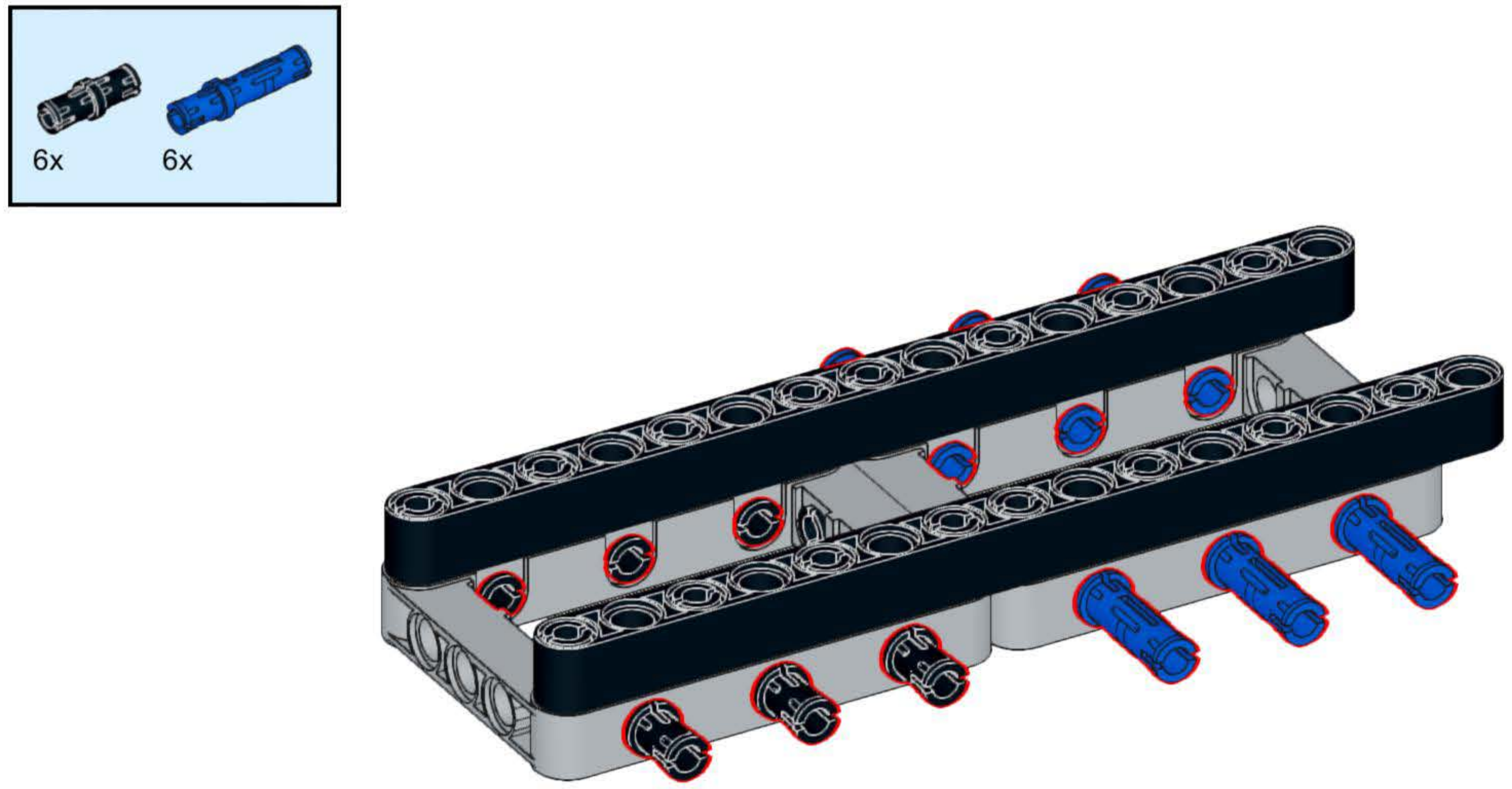

40

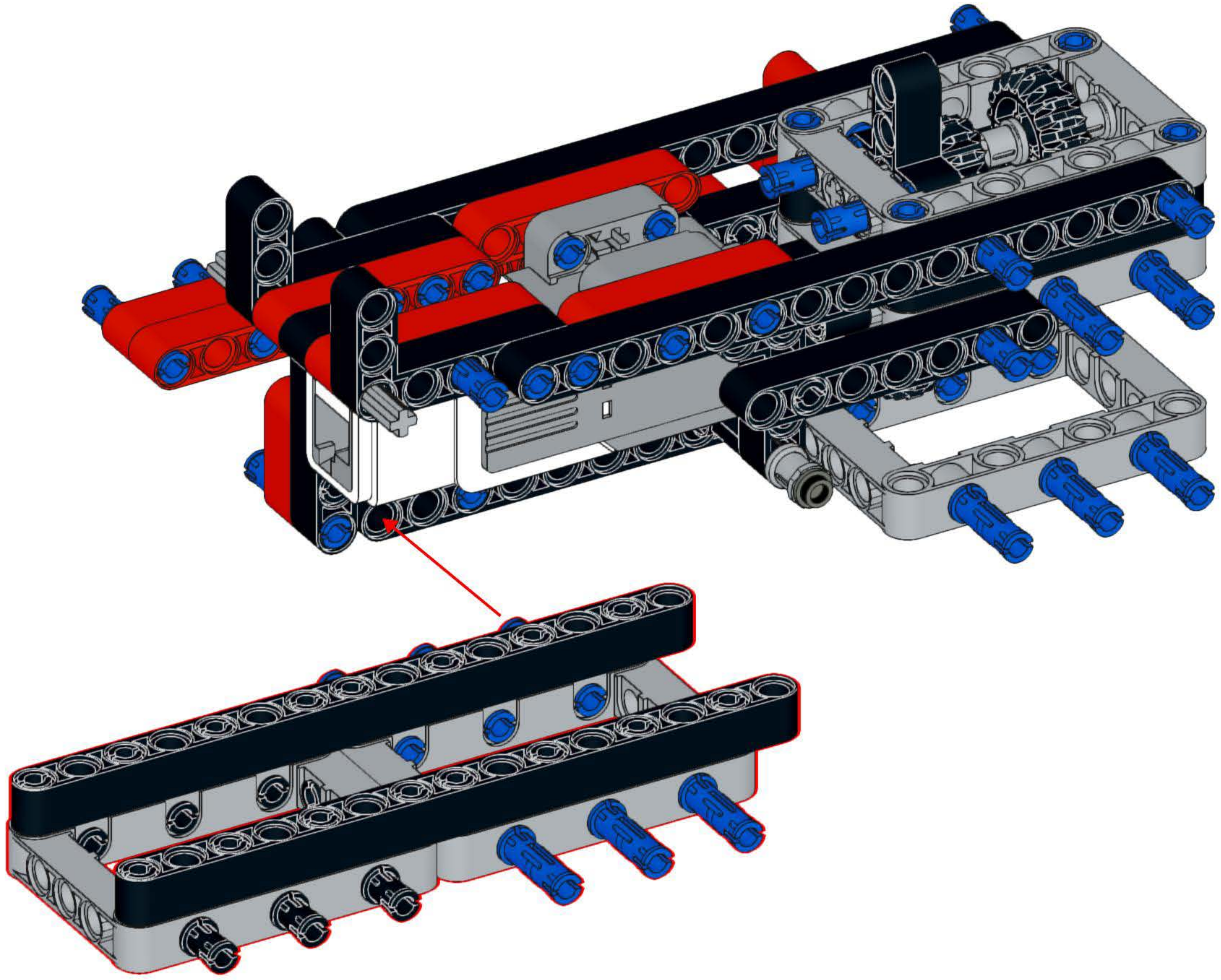

# 41

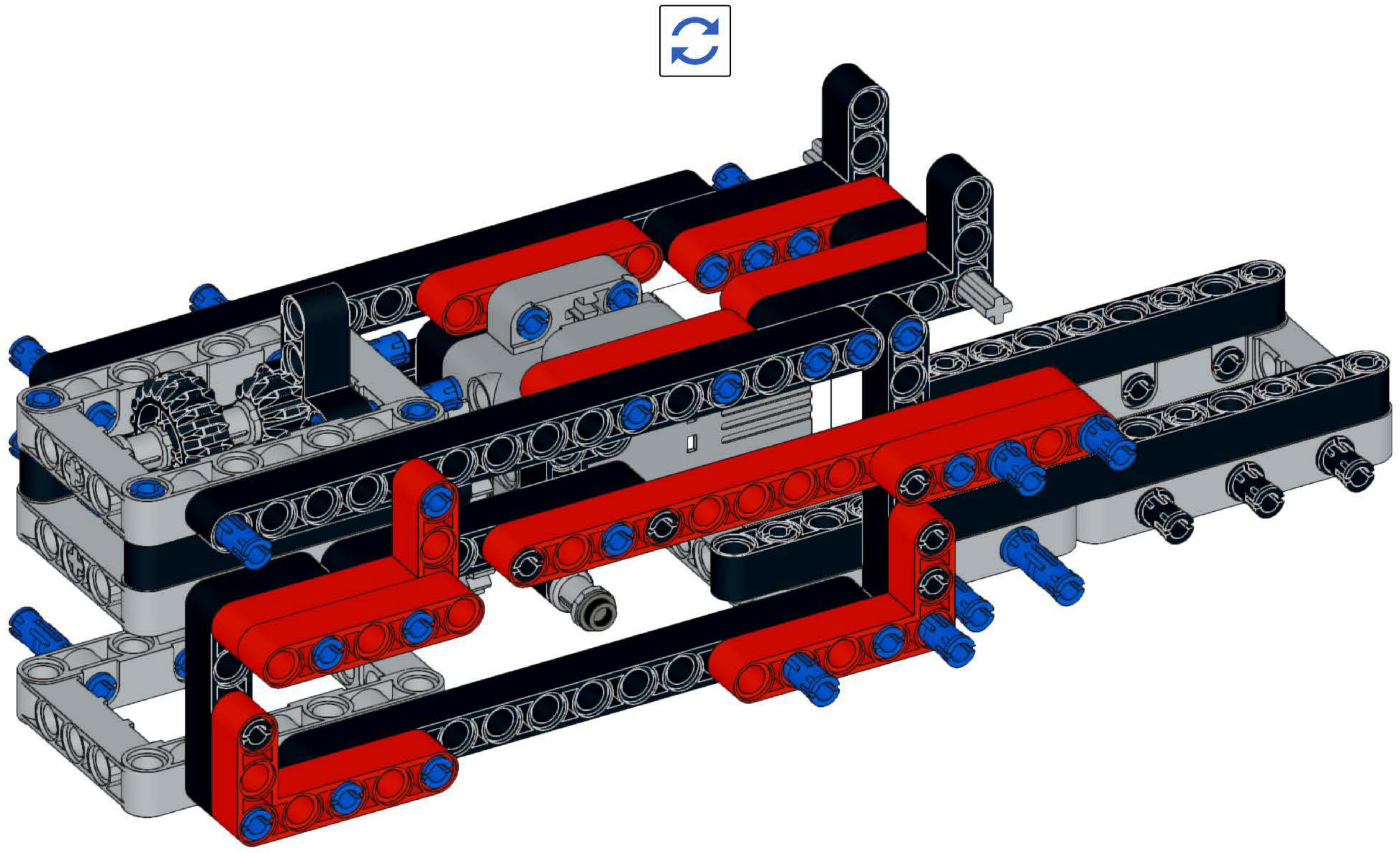

# 42

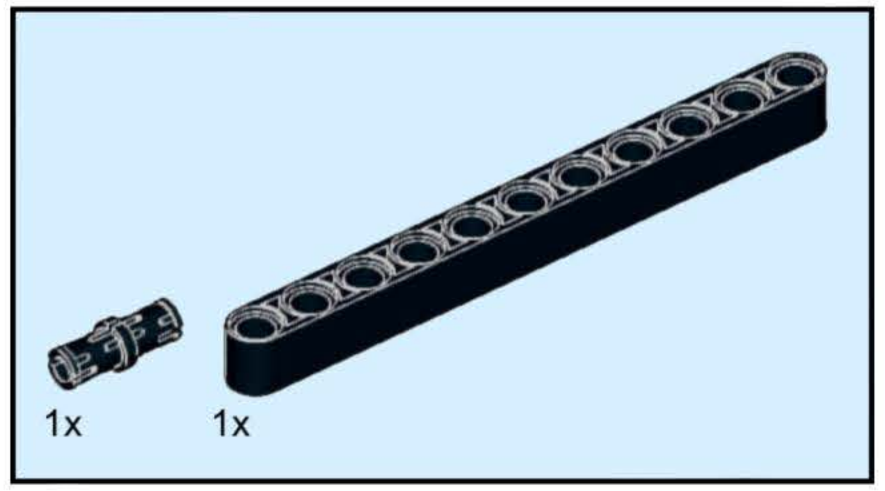


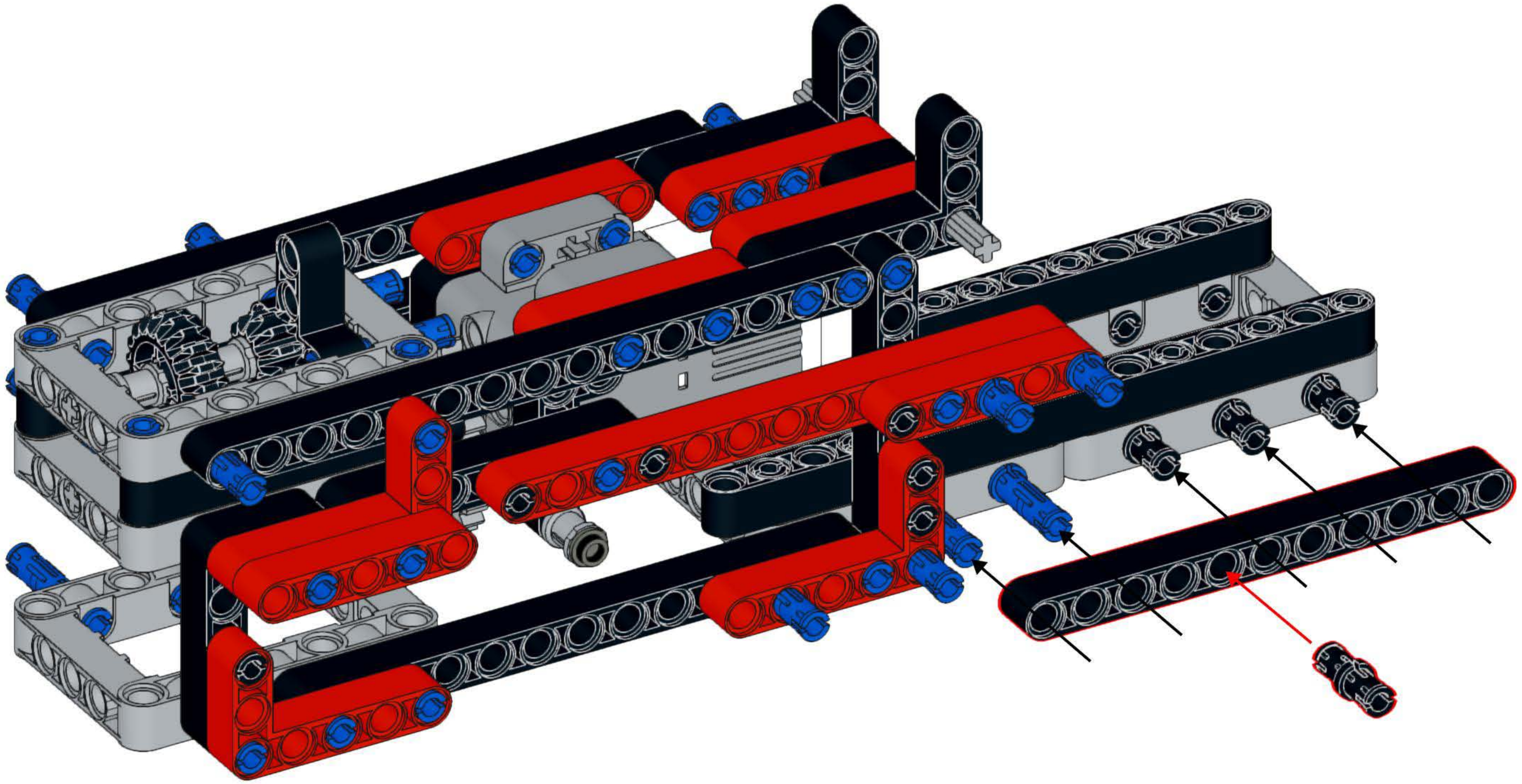

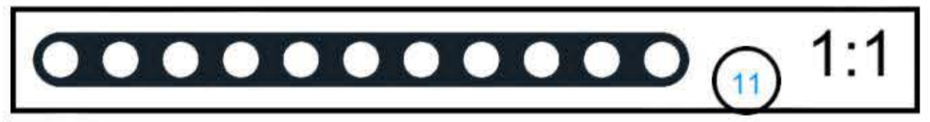

# 43

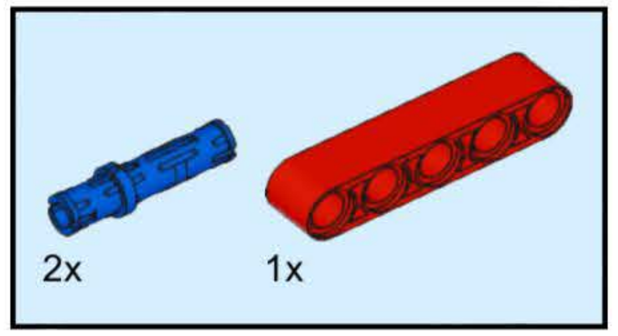


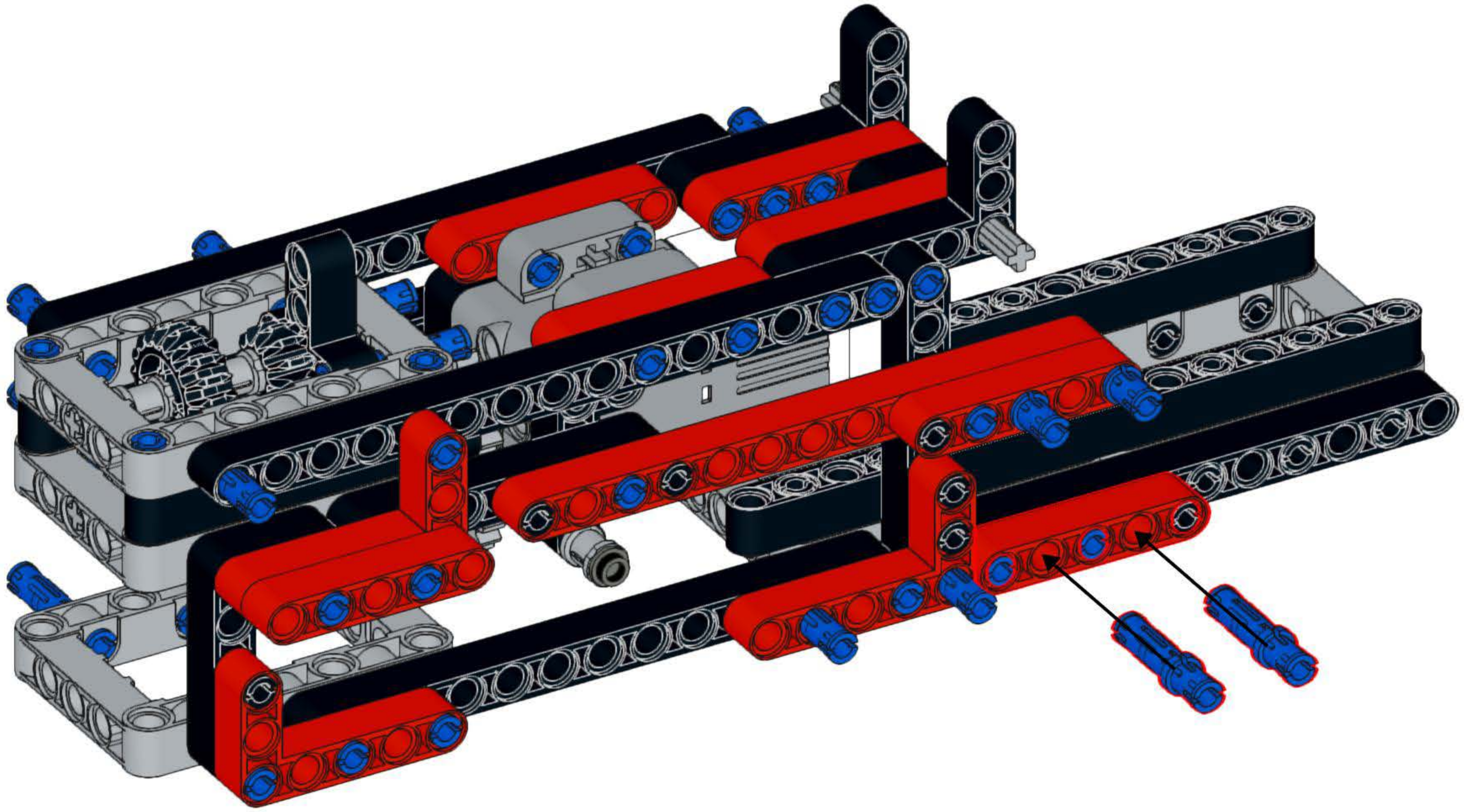

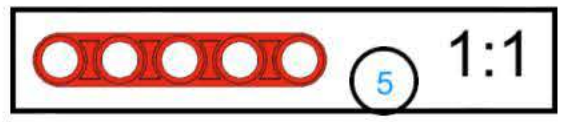


# 44

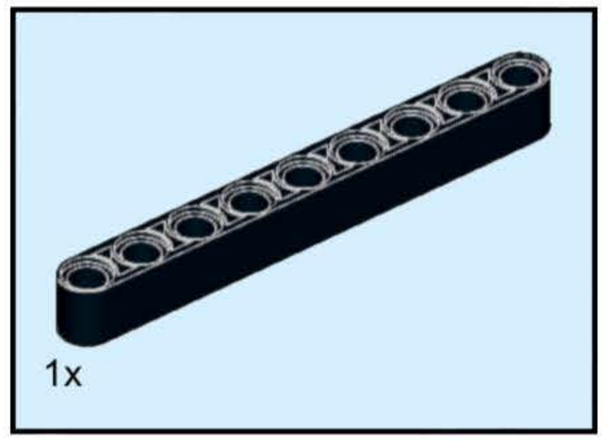


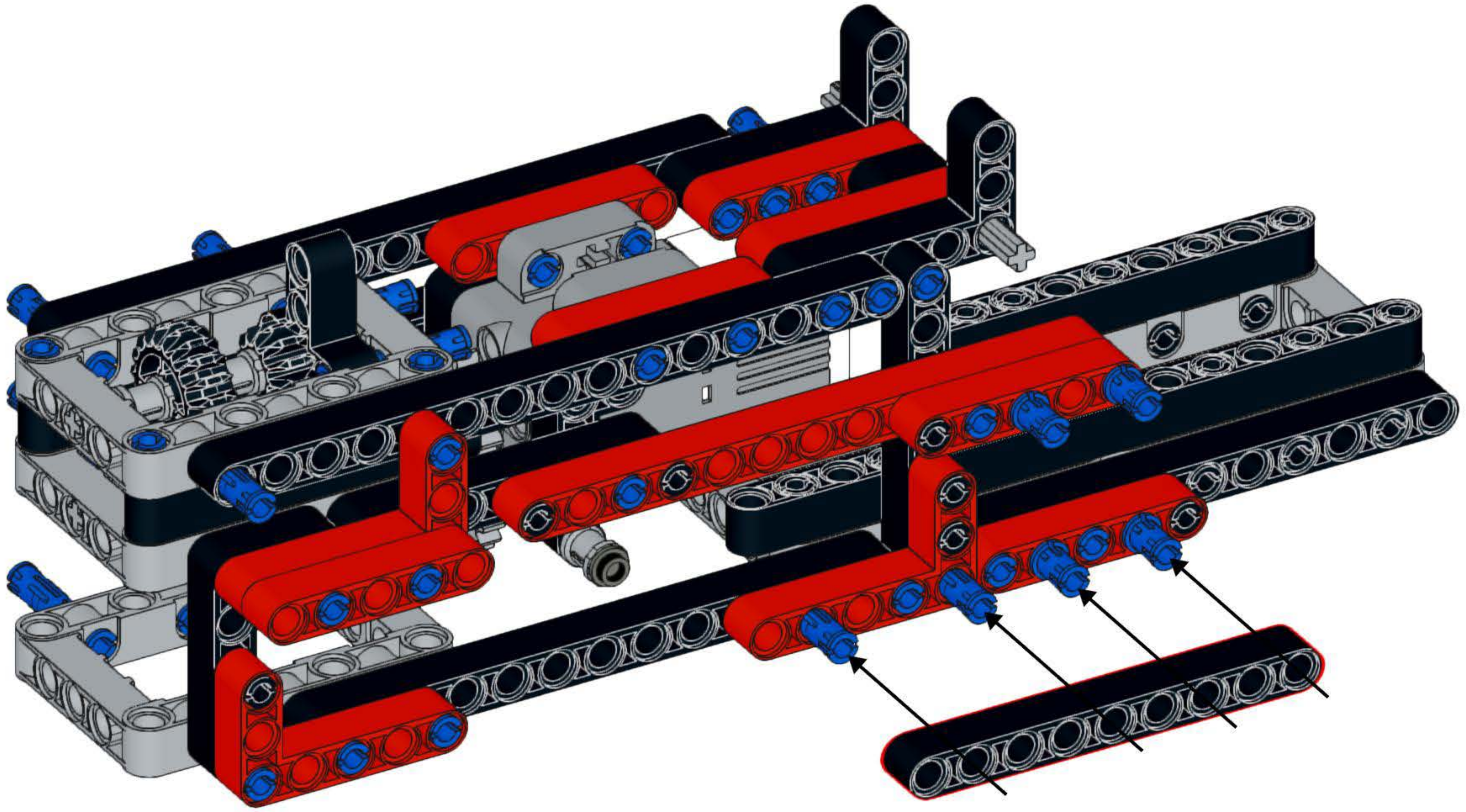

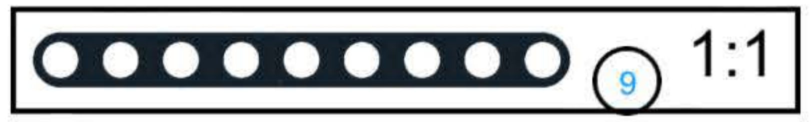

**45**

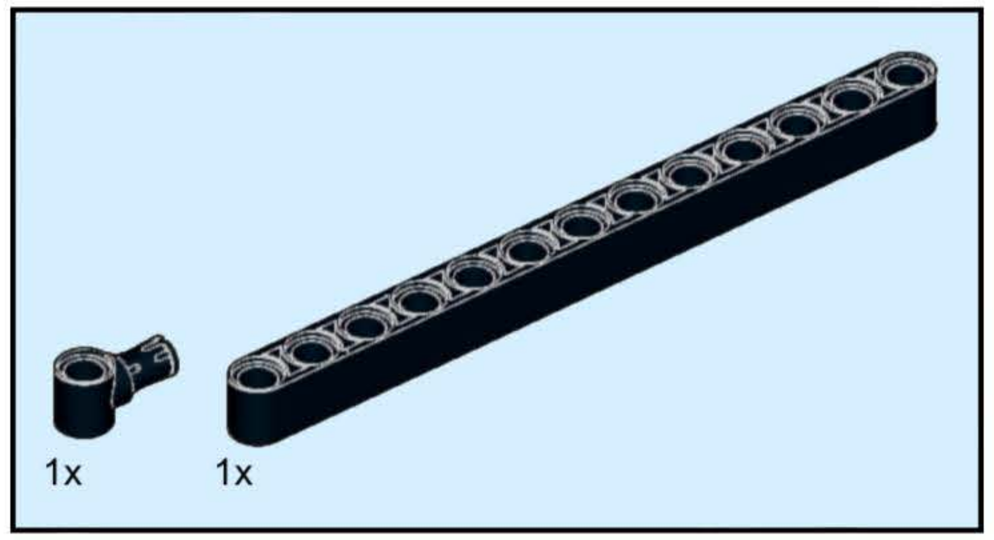


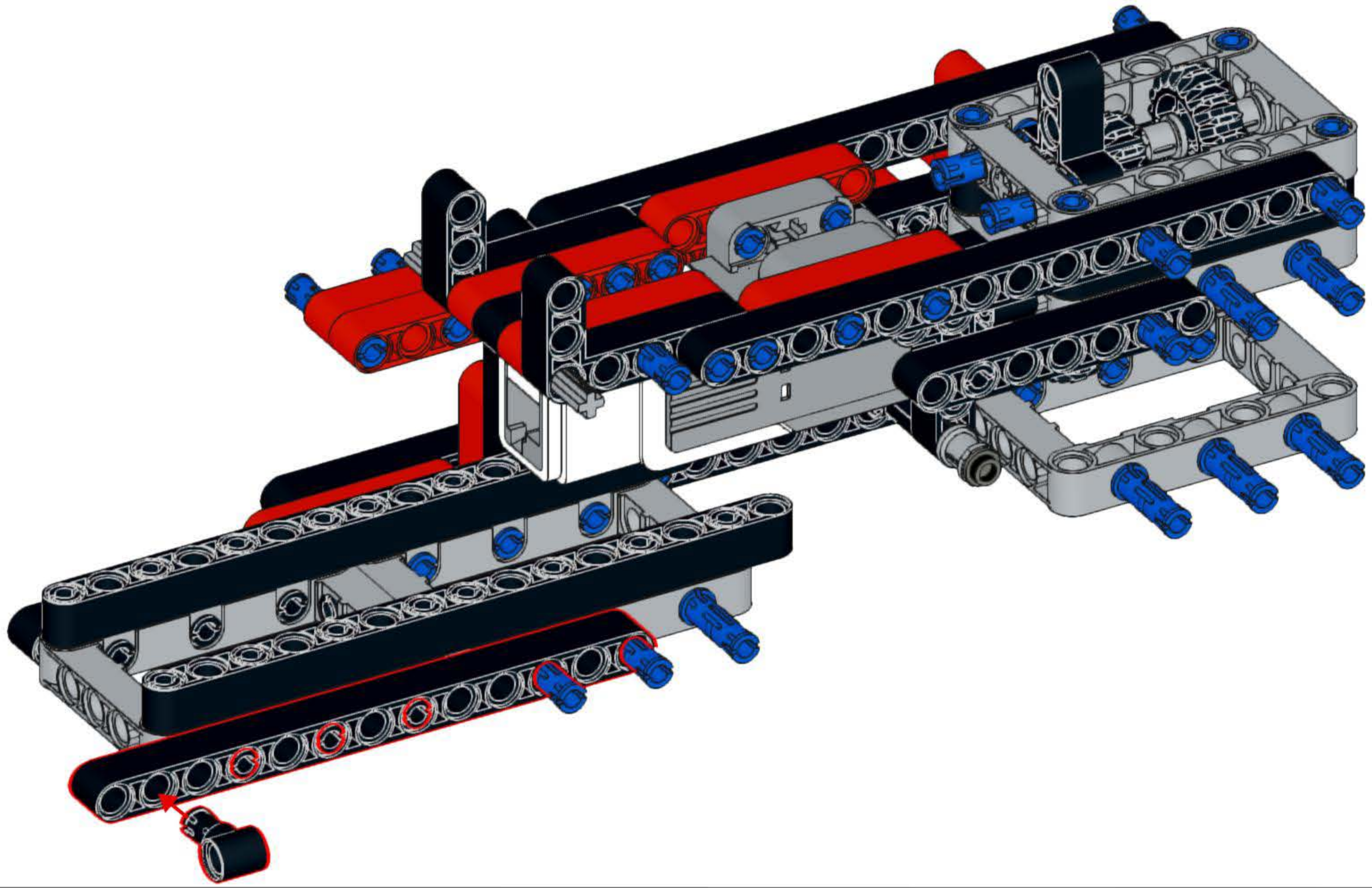

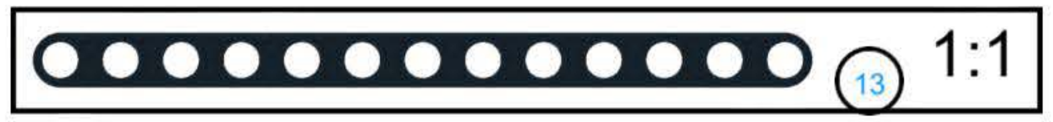


**46**

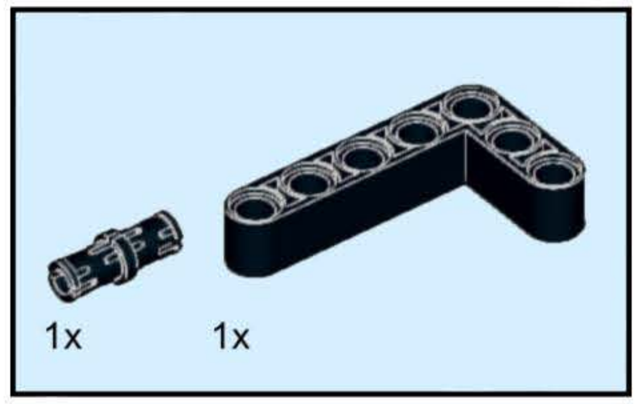


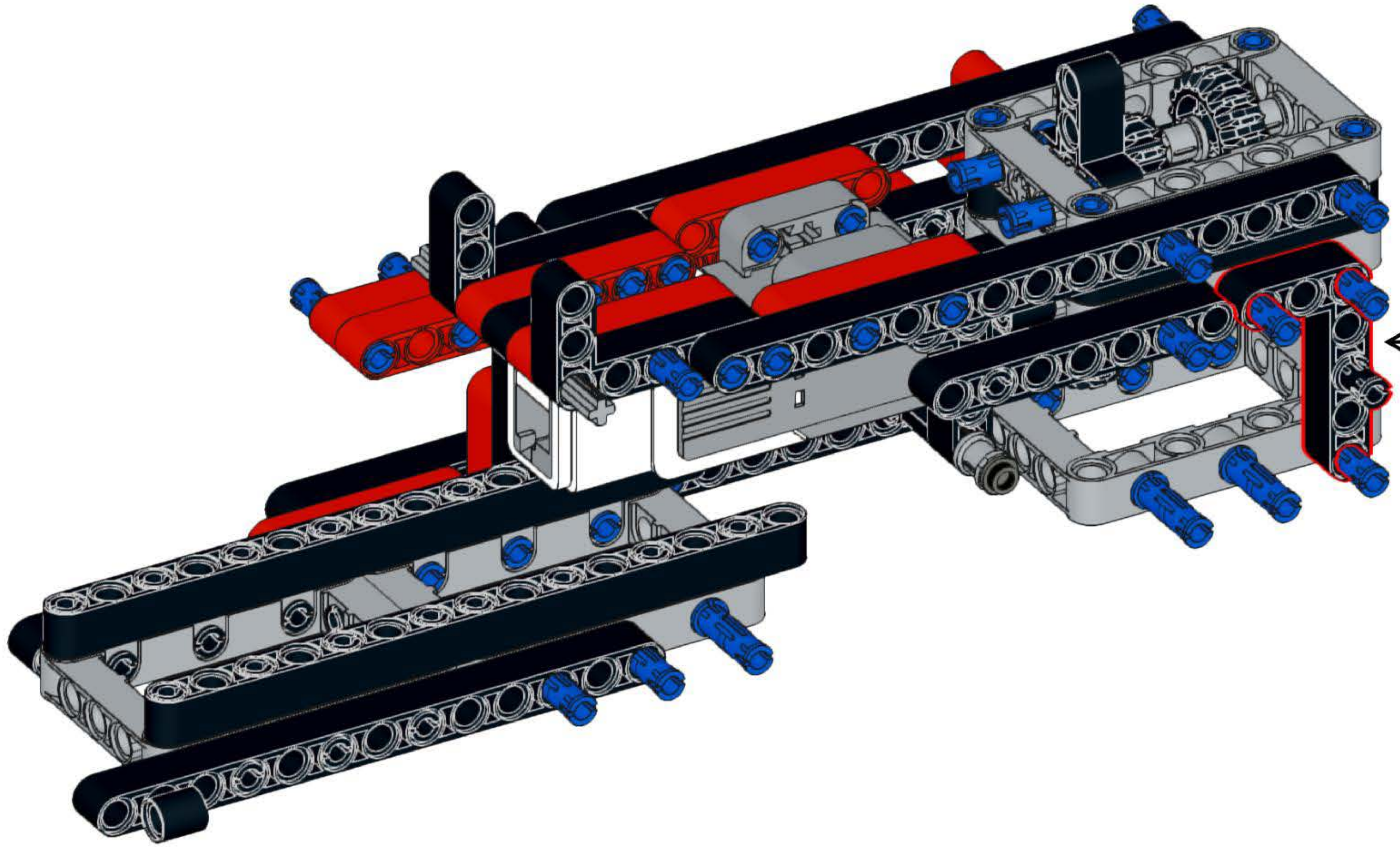

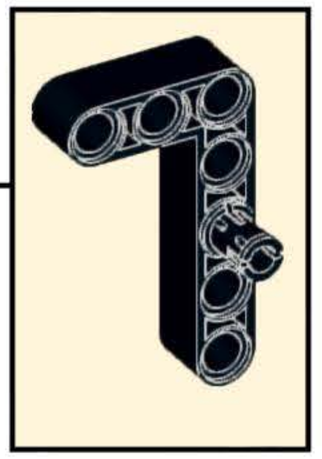

47

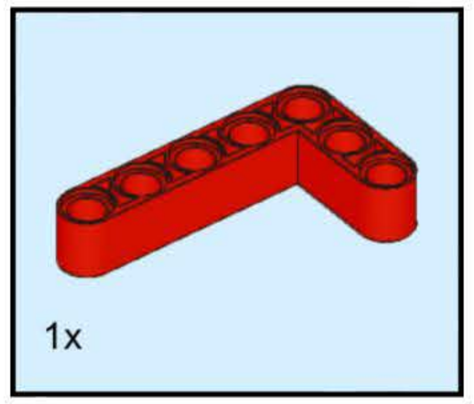


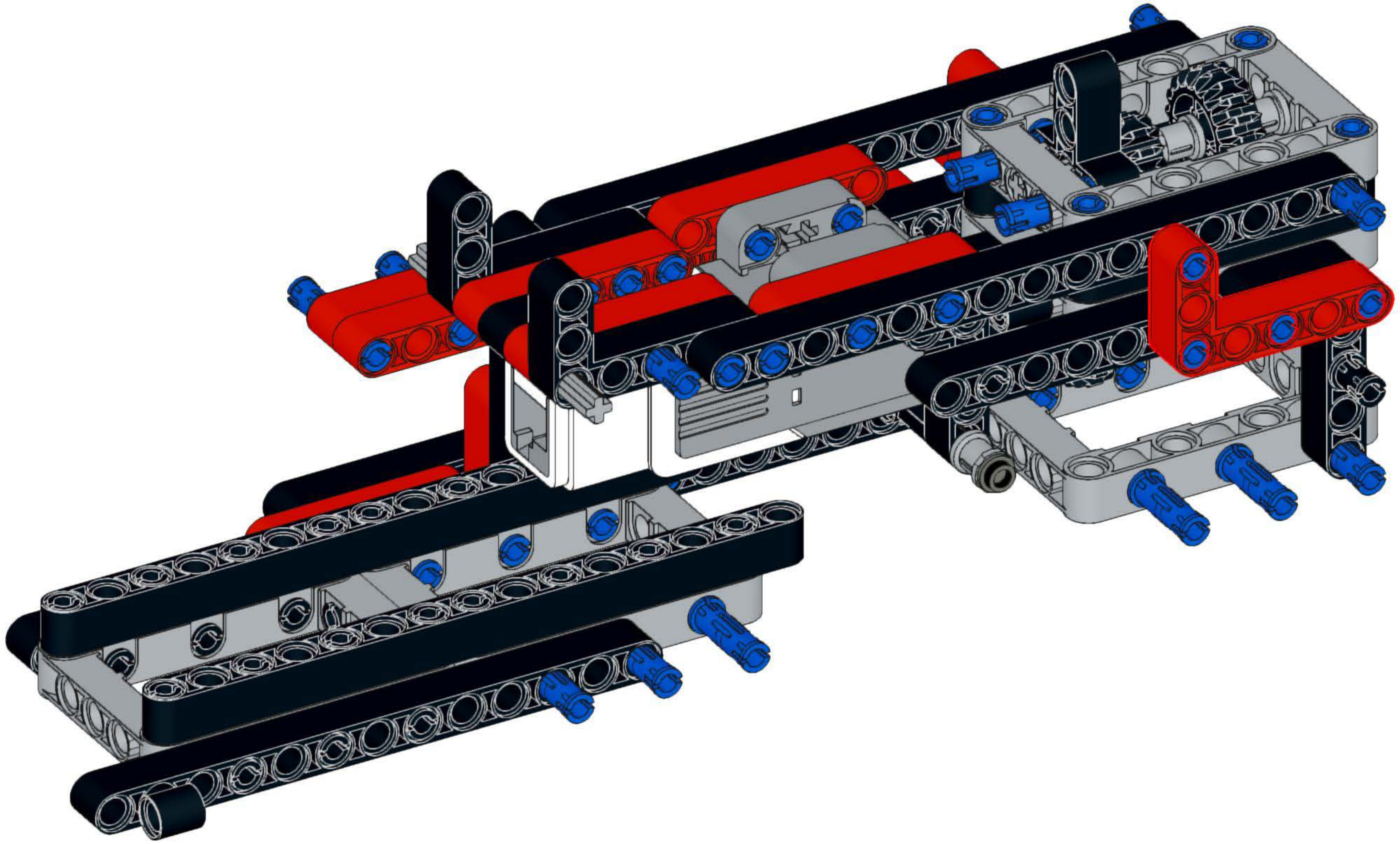

48

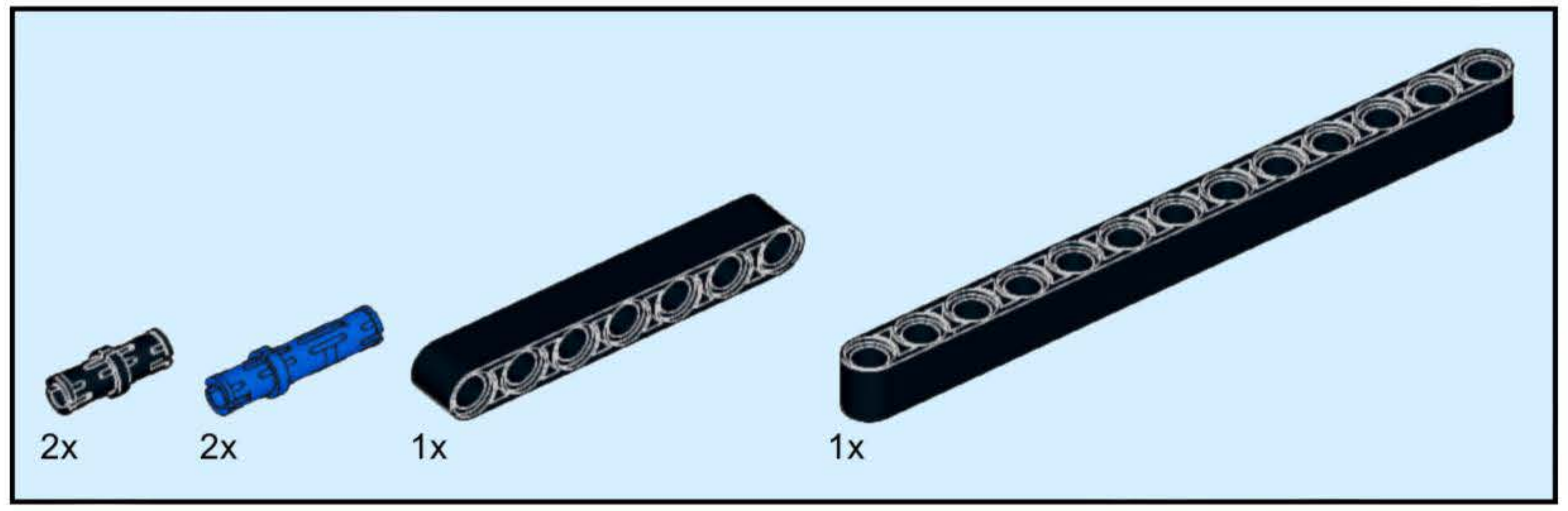


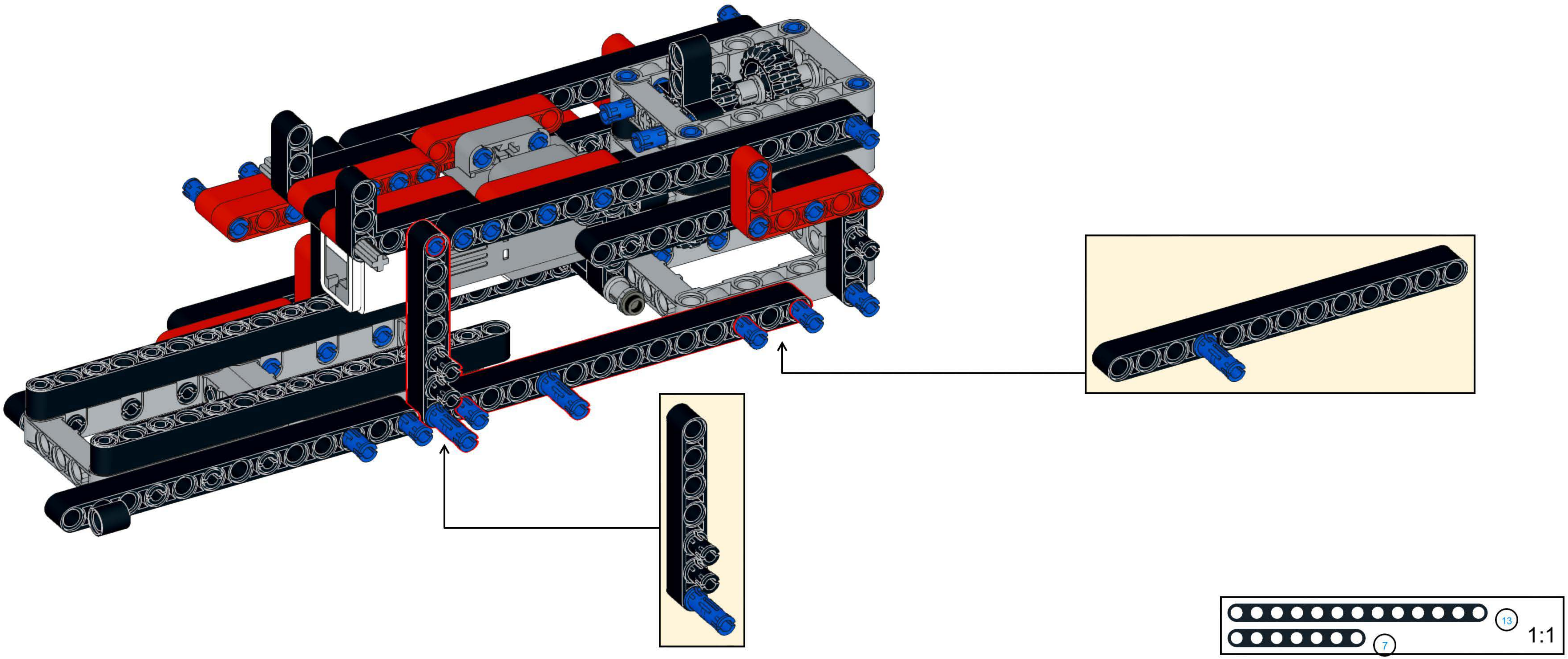

49

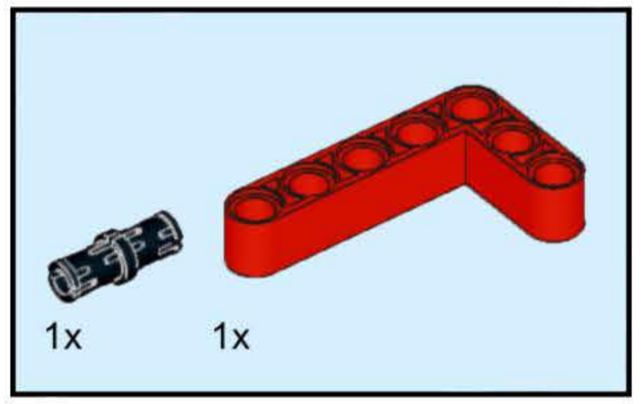


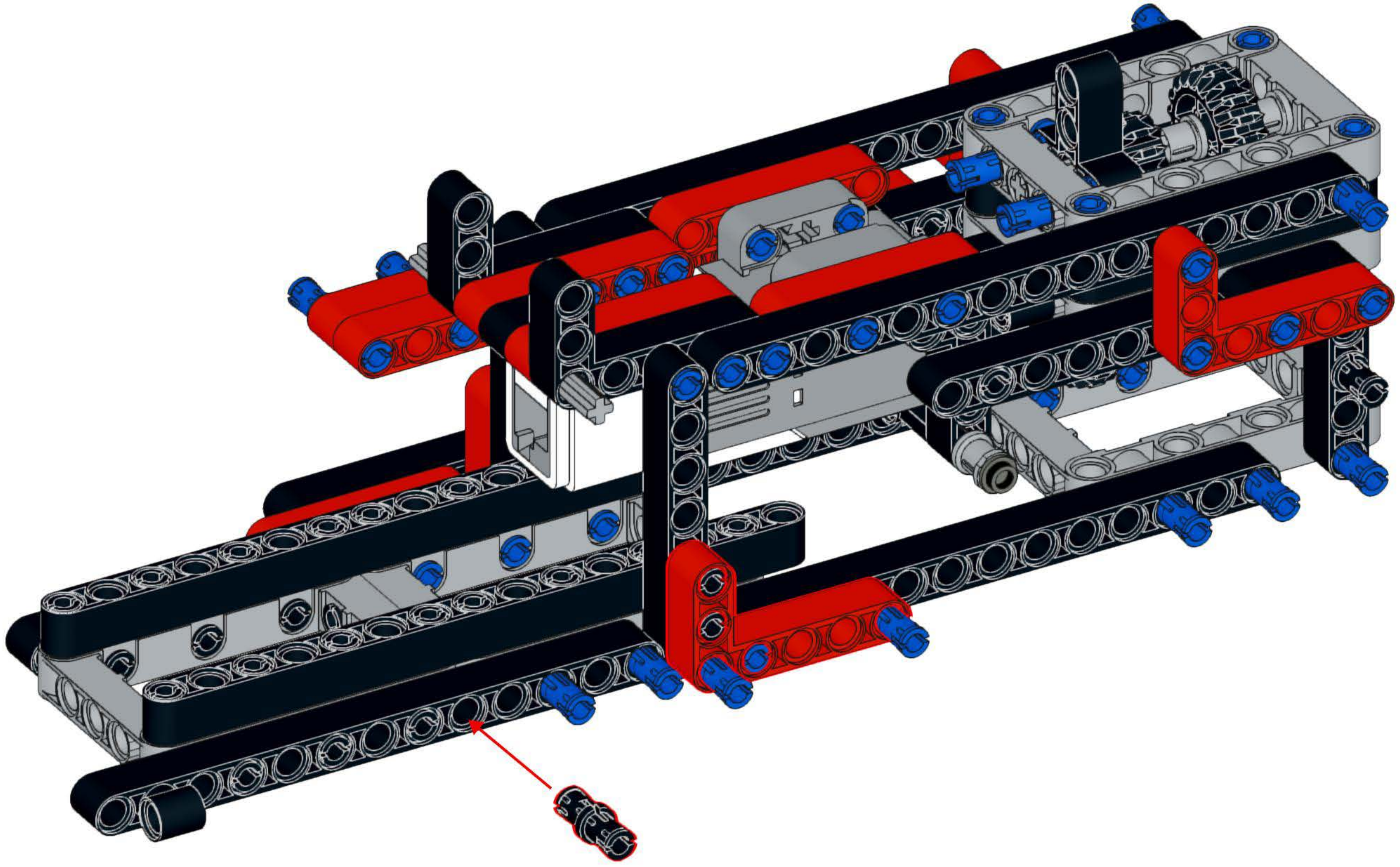

50

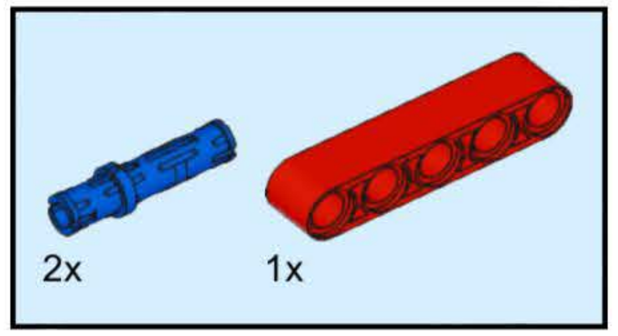


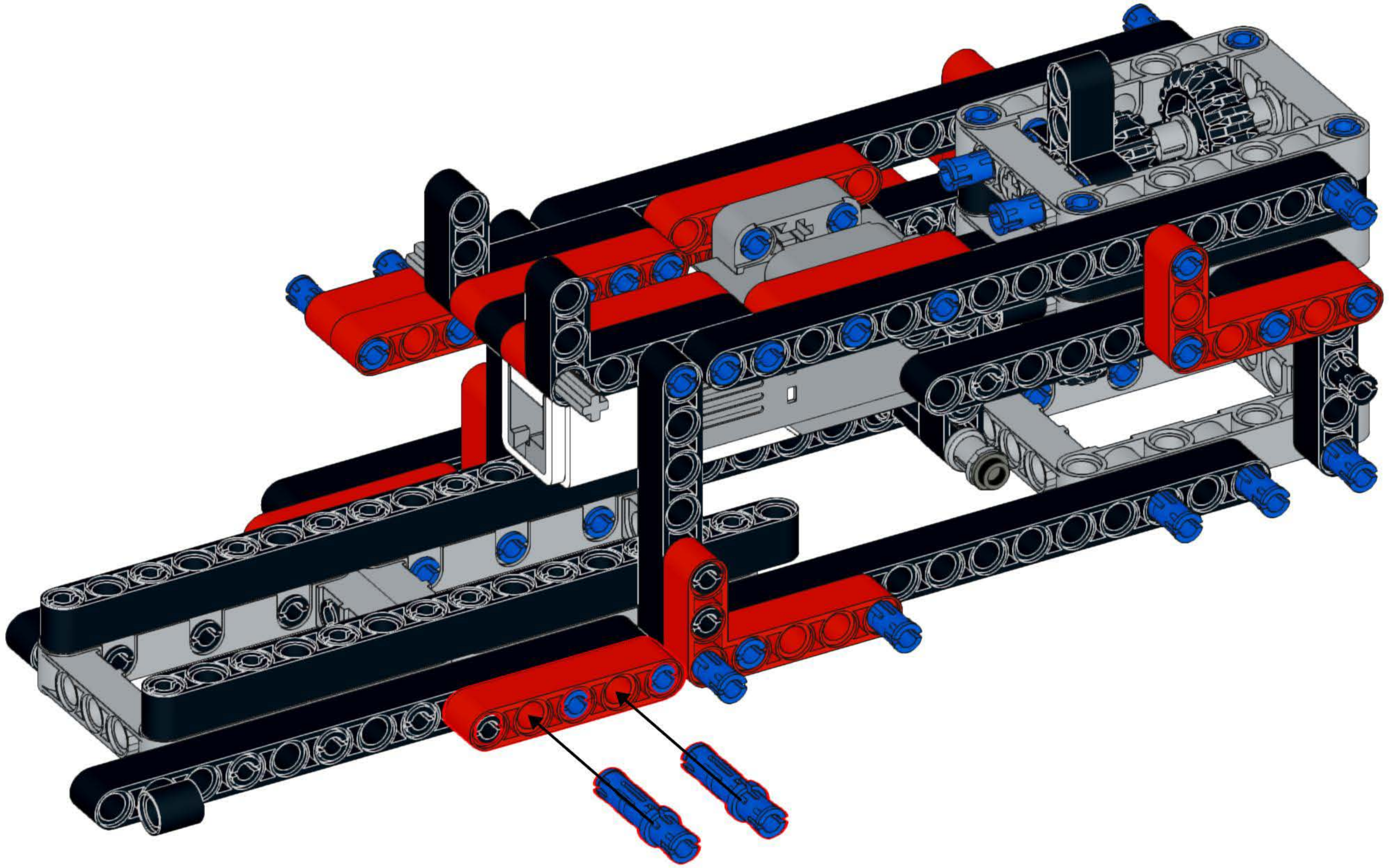

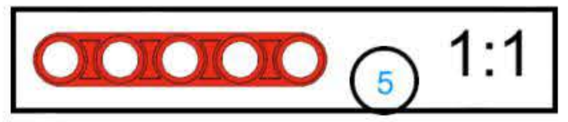

51

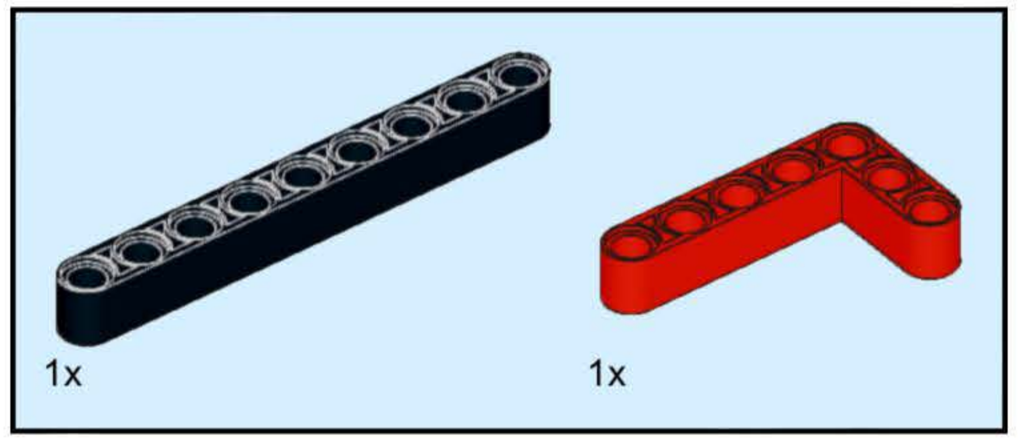


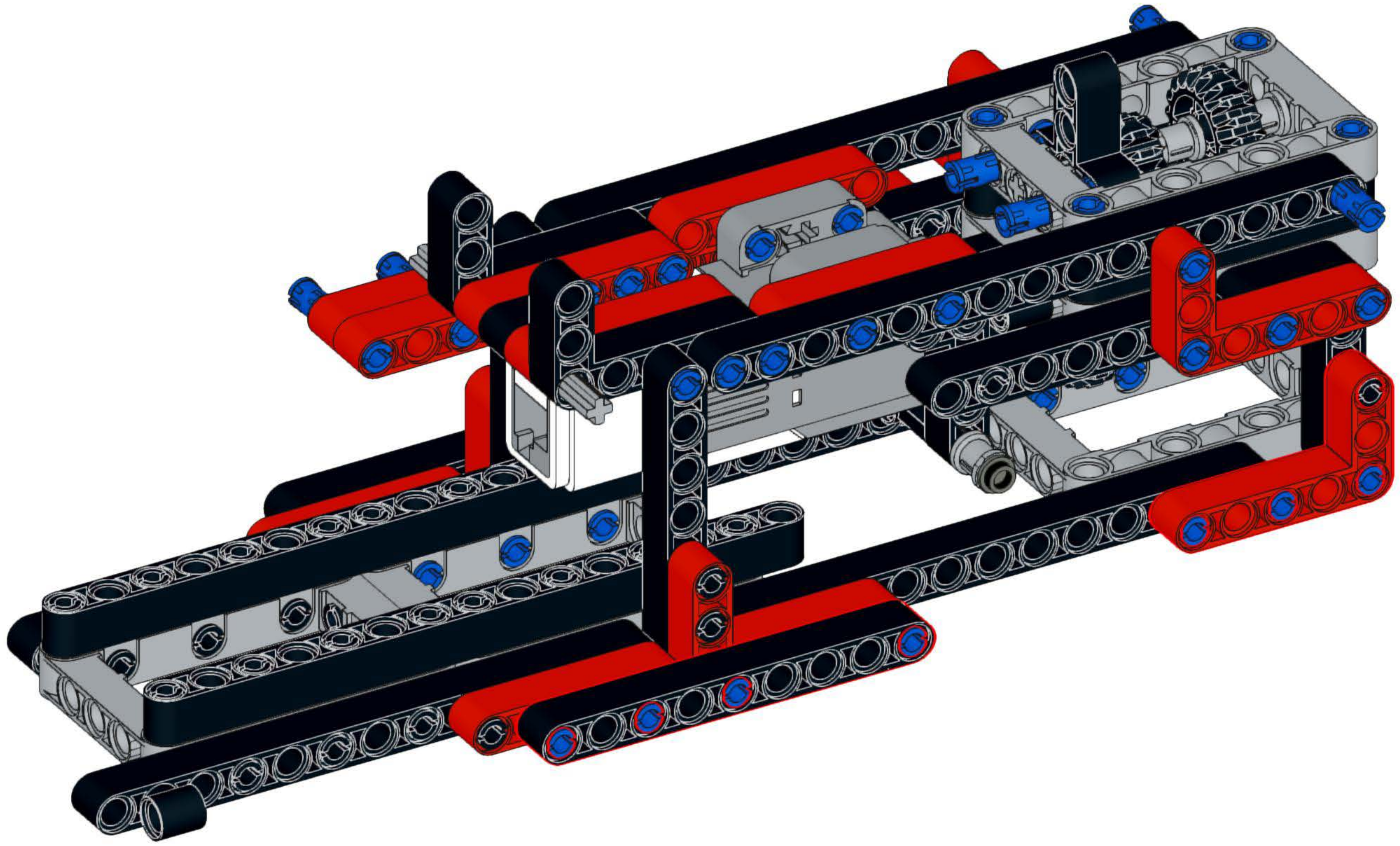

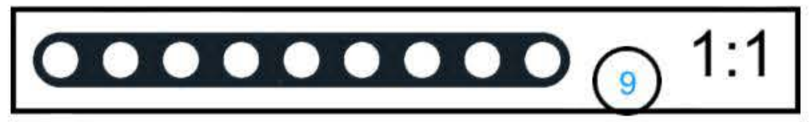

52

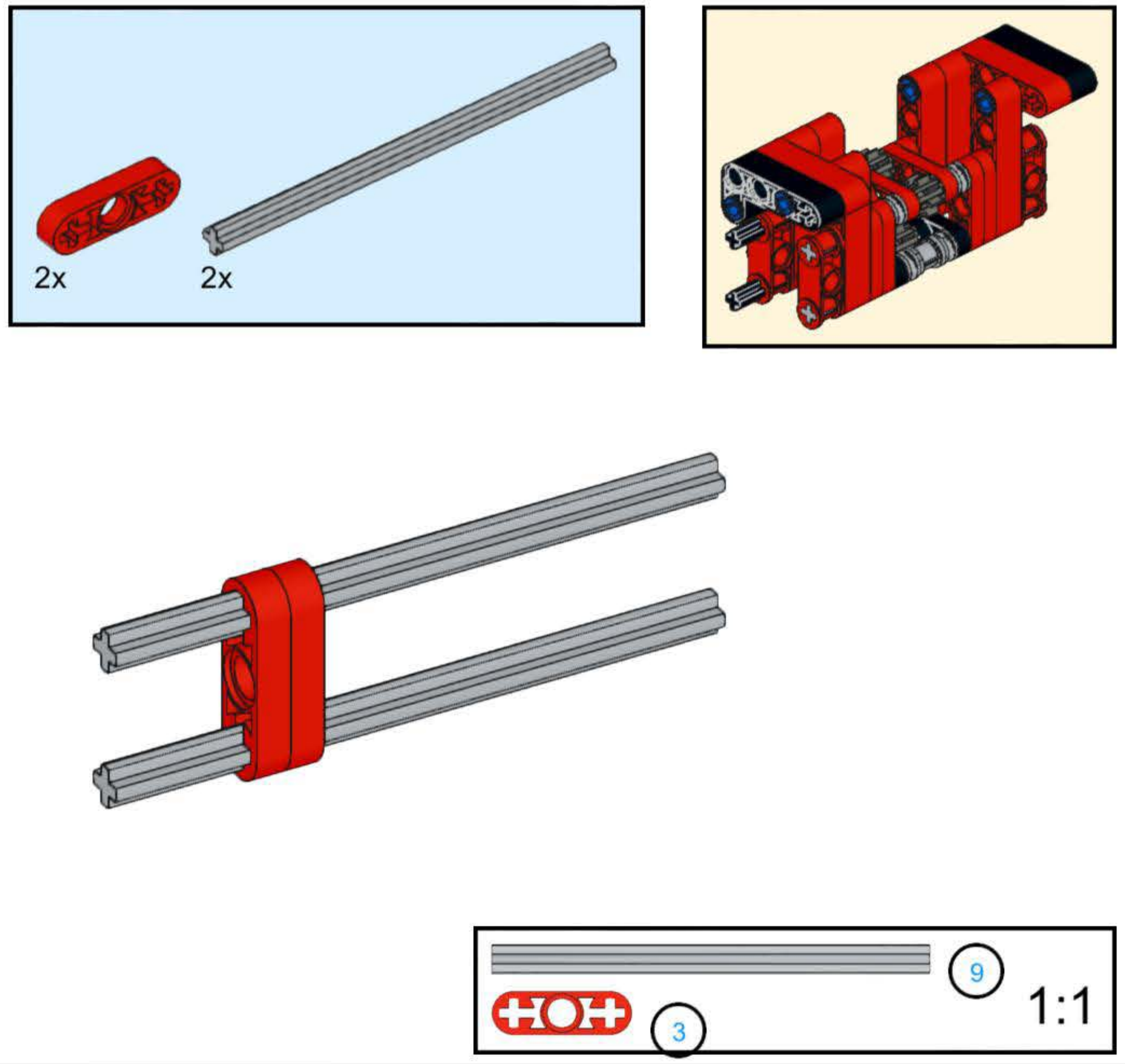


53

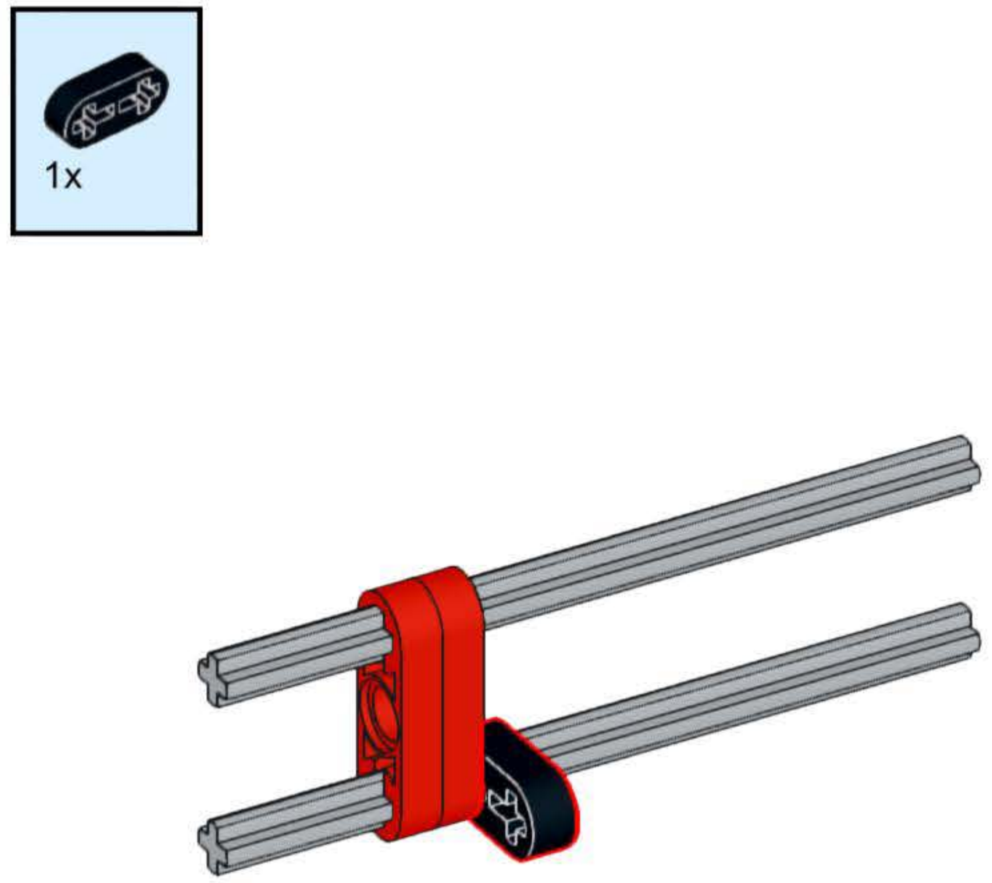


54

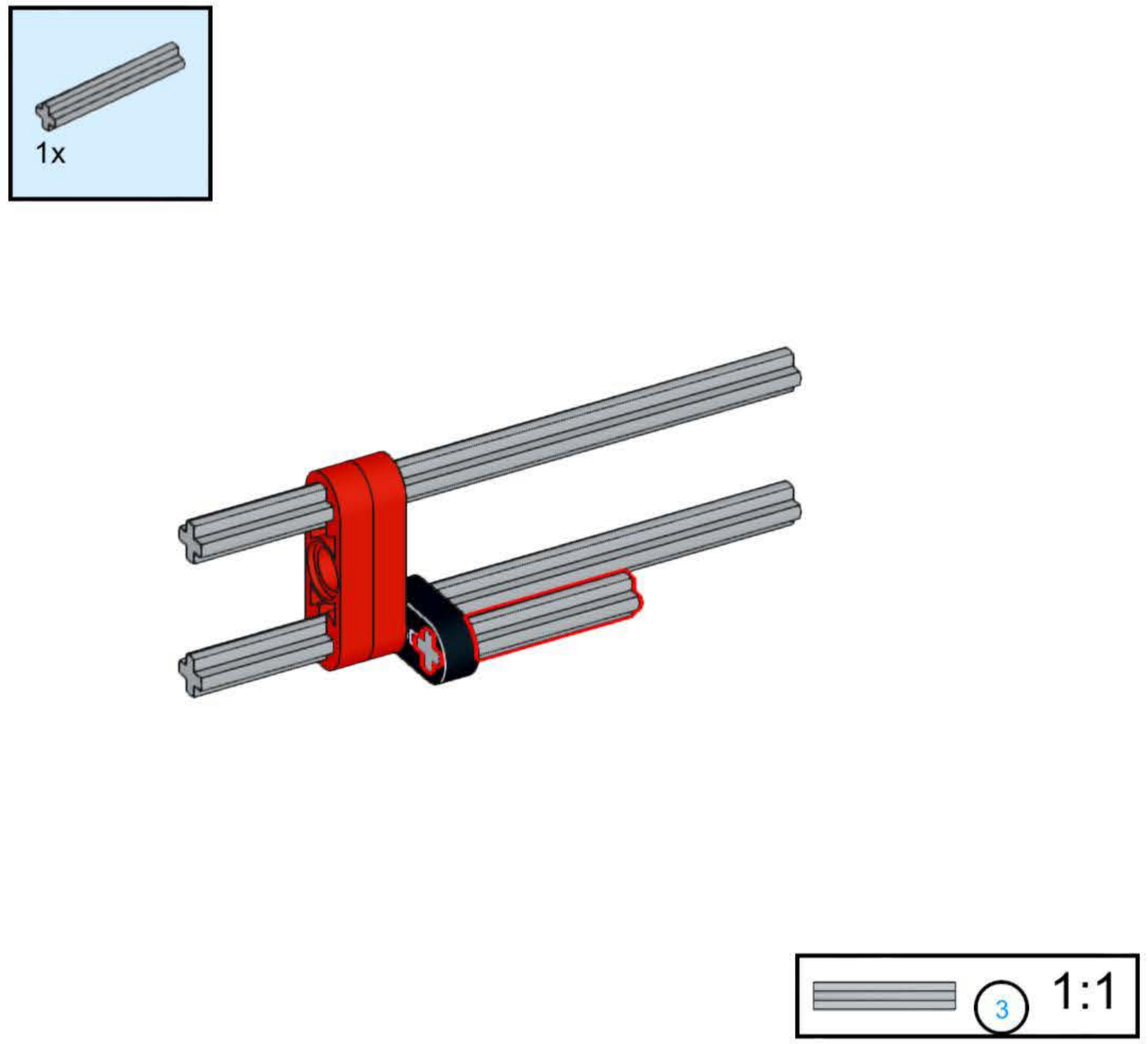


55

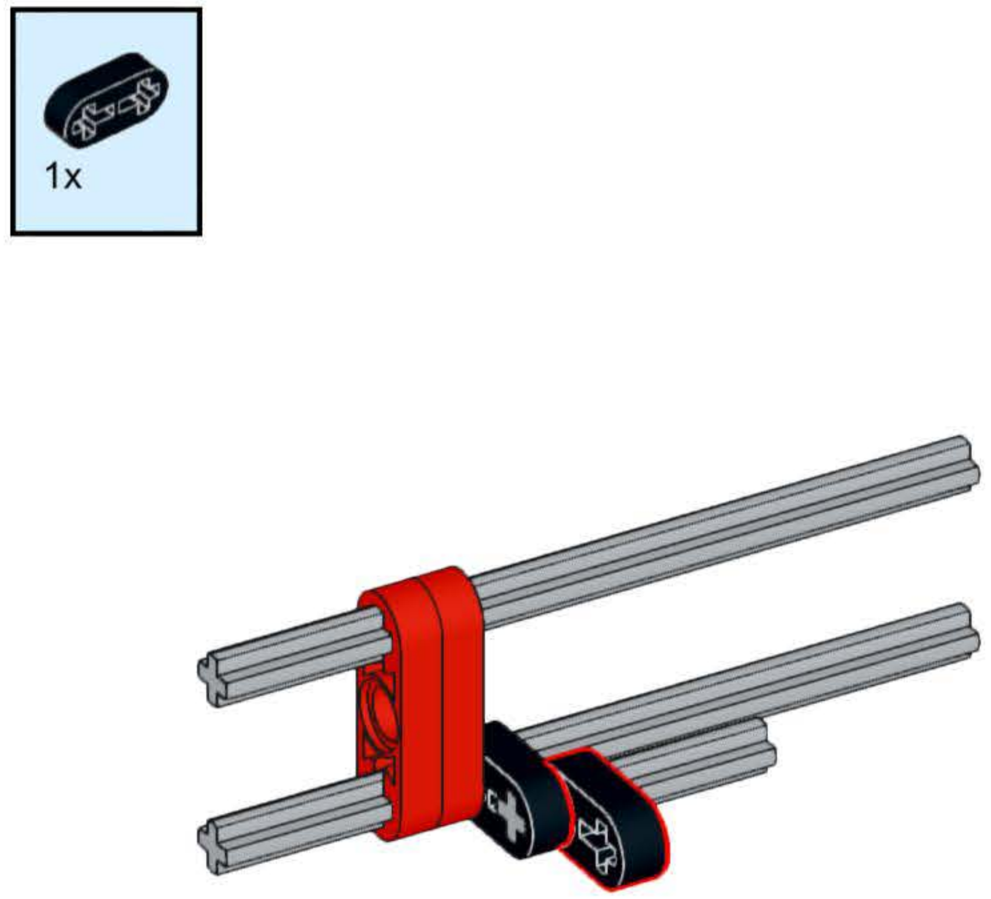


56

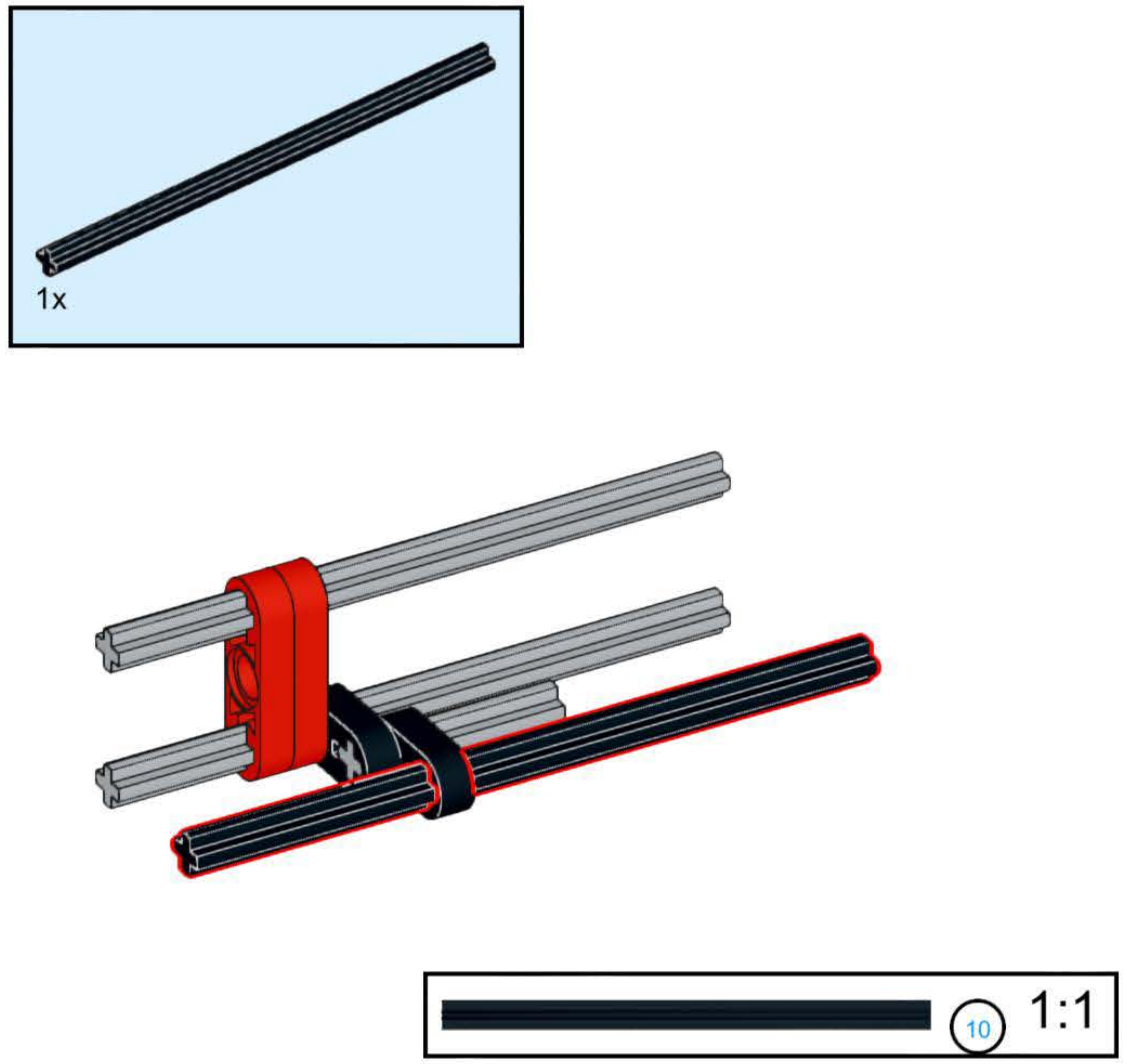


57

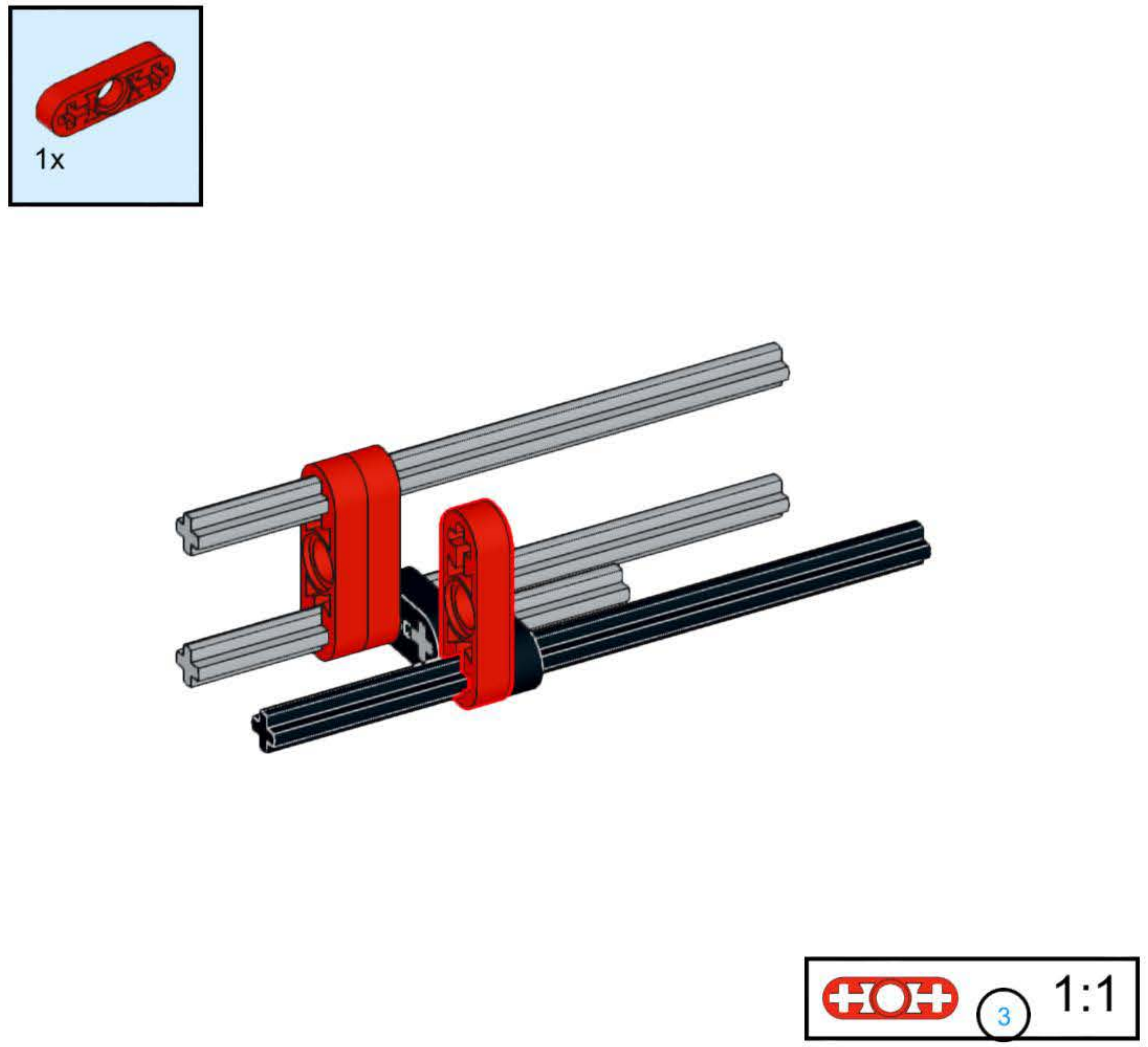

## 58

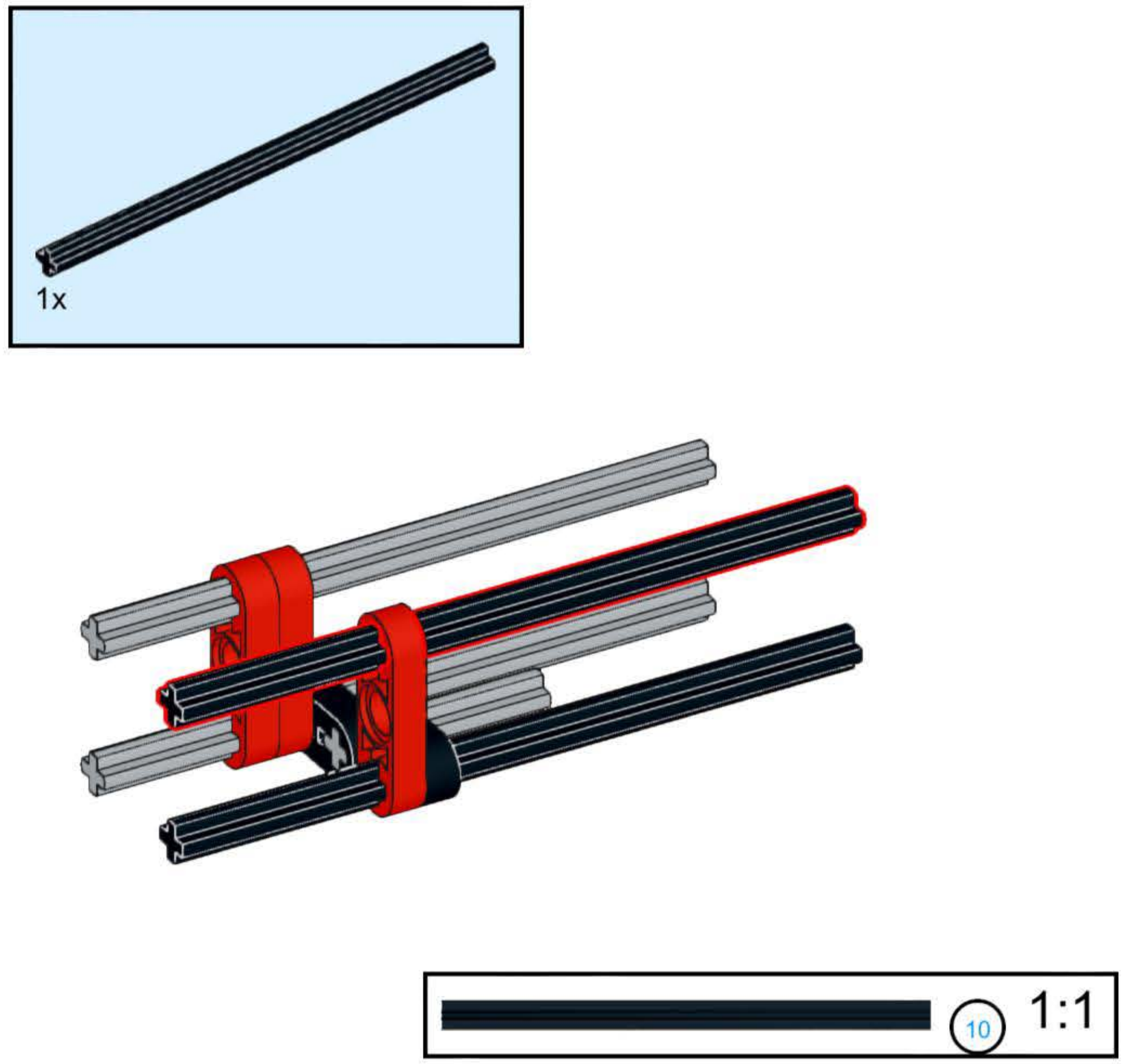


## 59

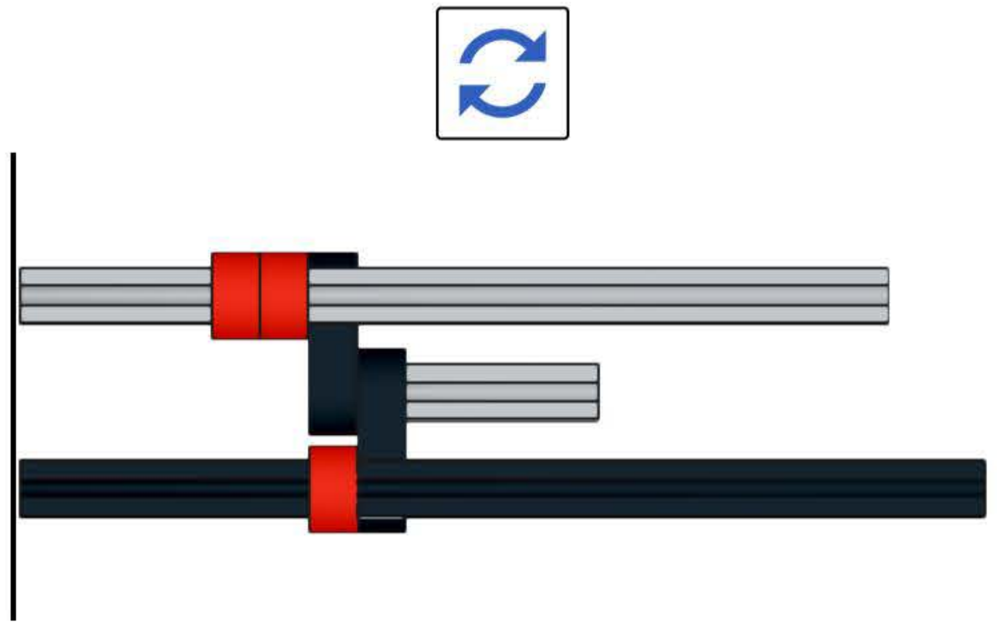

## 60

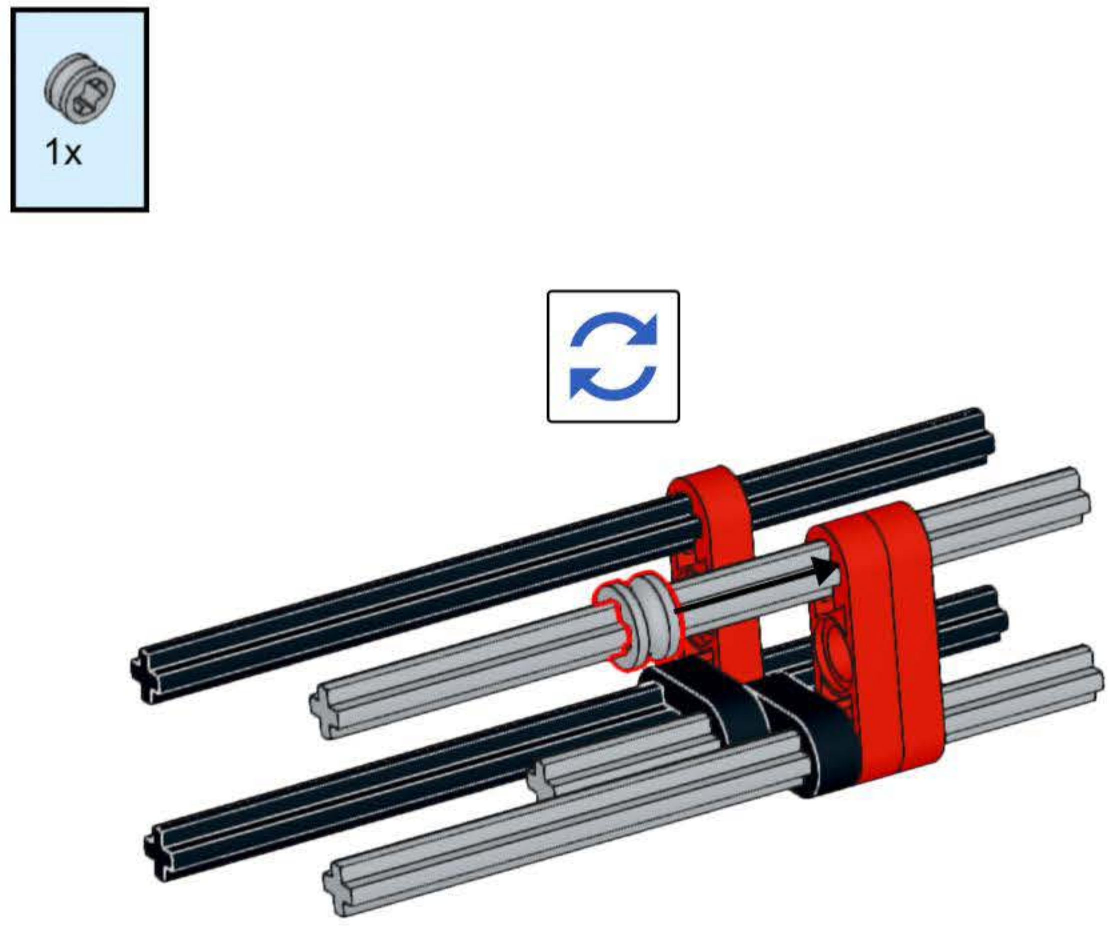


## 61

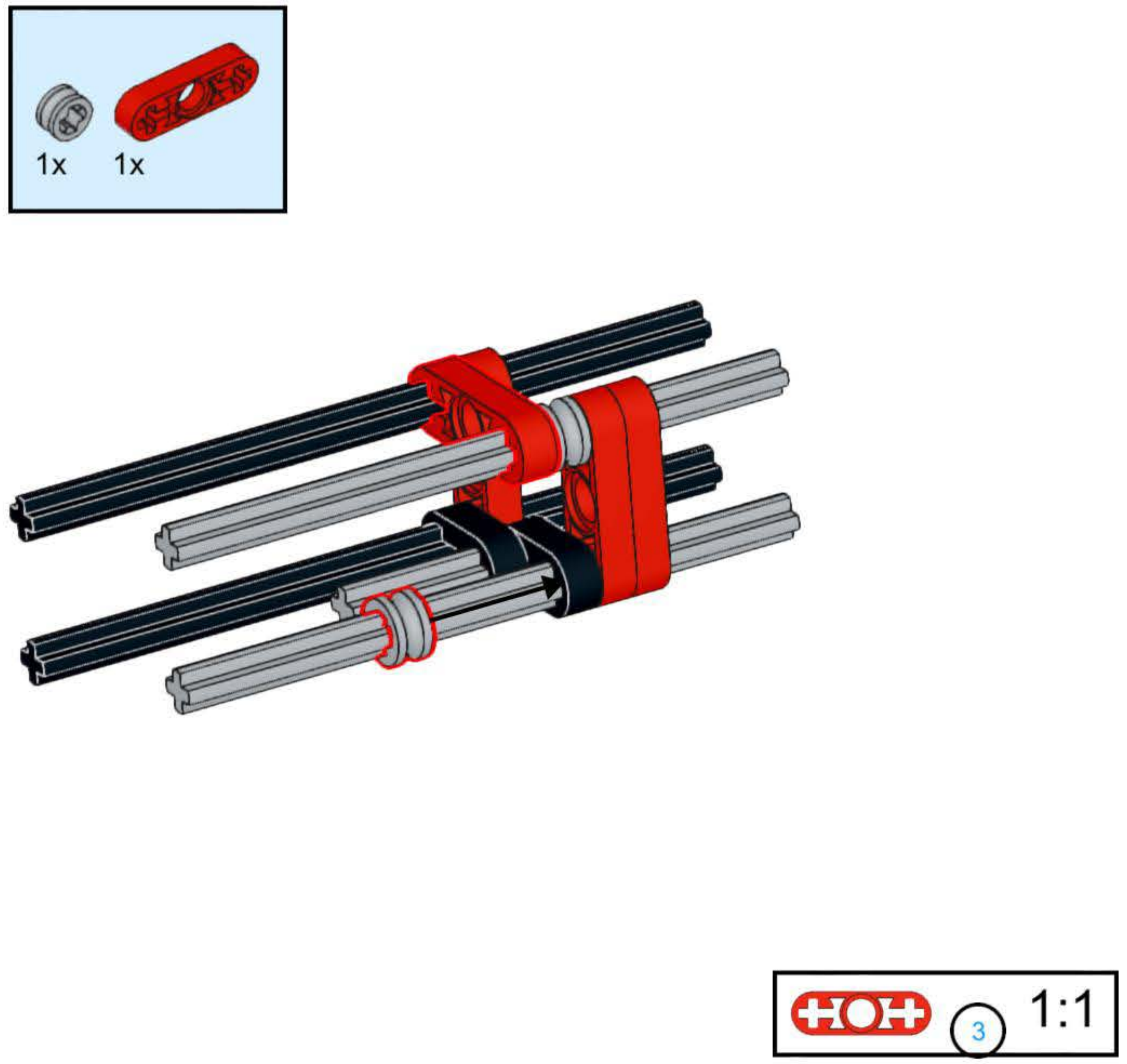


## 62

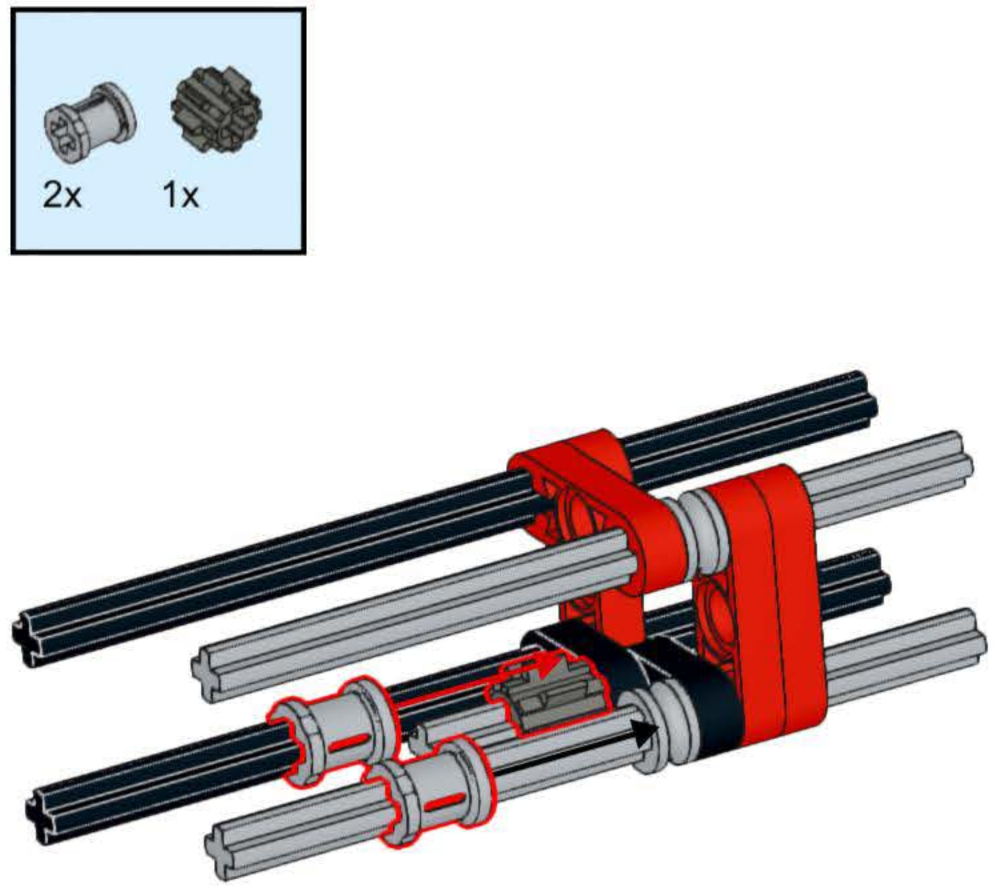


## 63

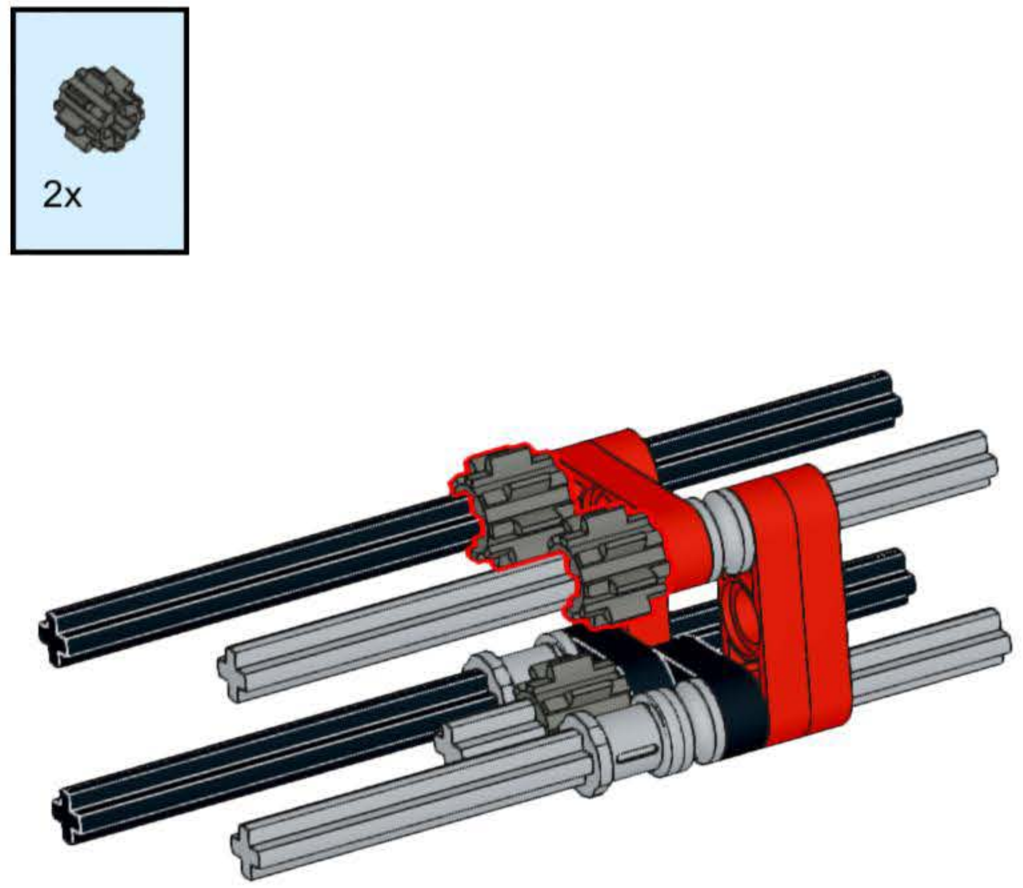

## 64

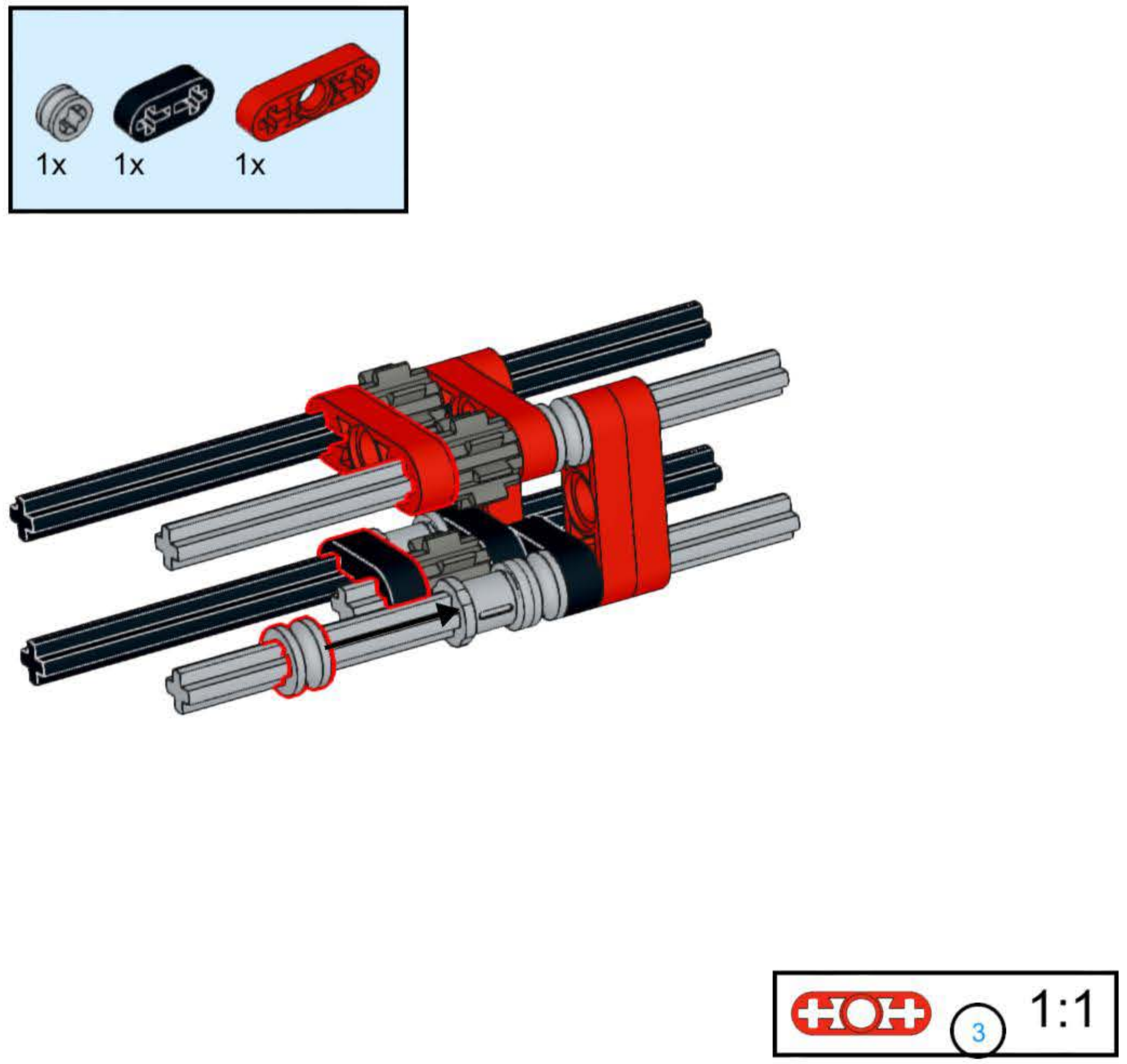


## 65

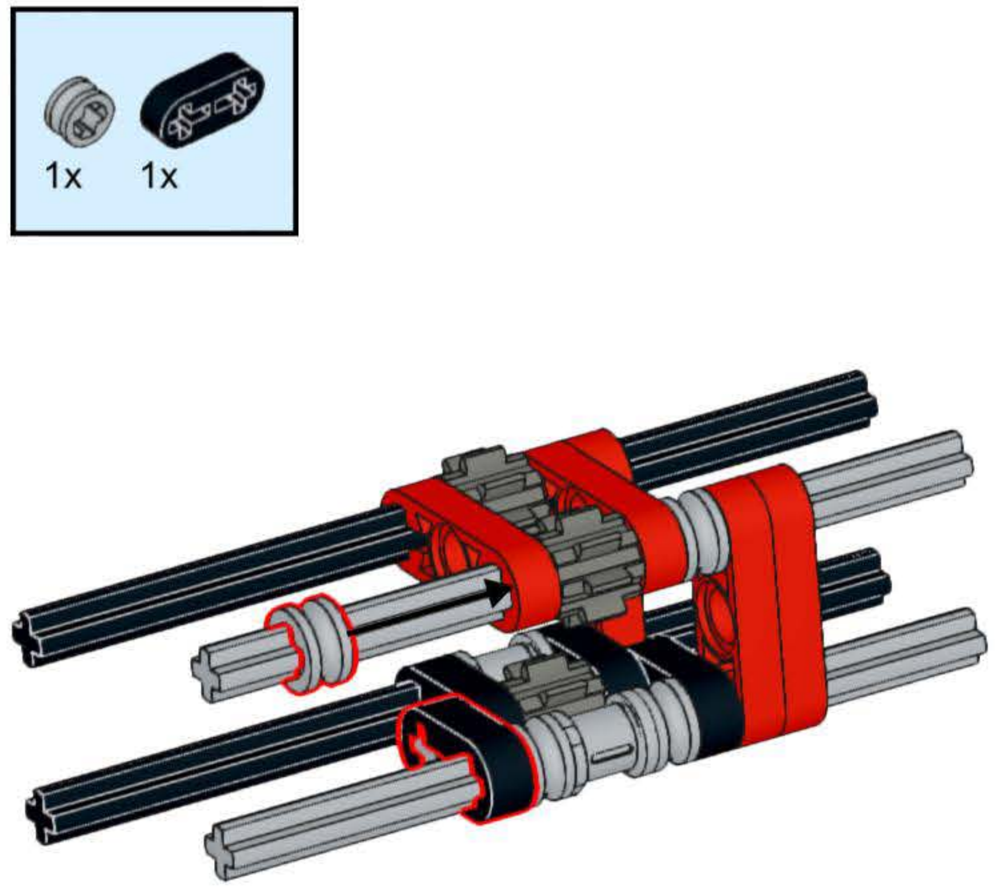


## 66

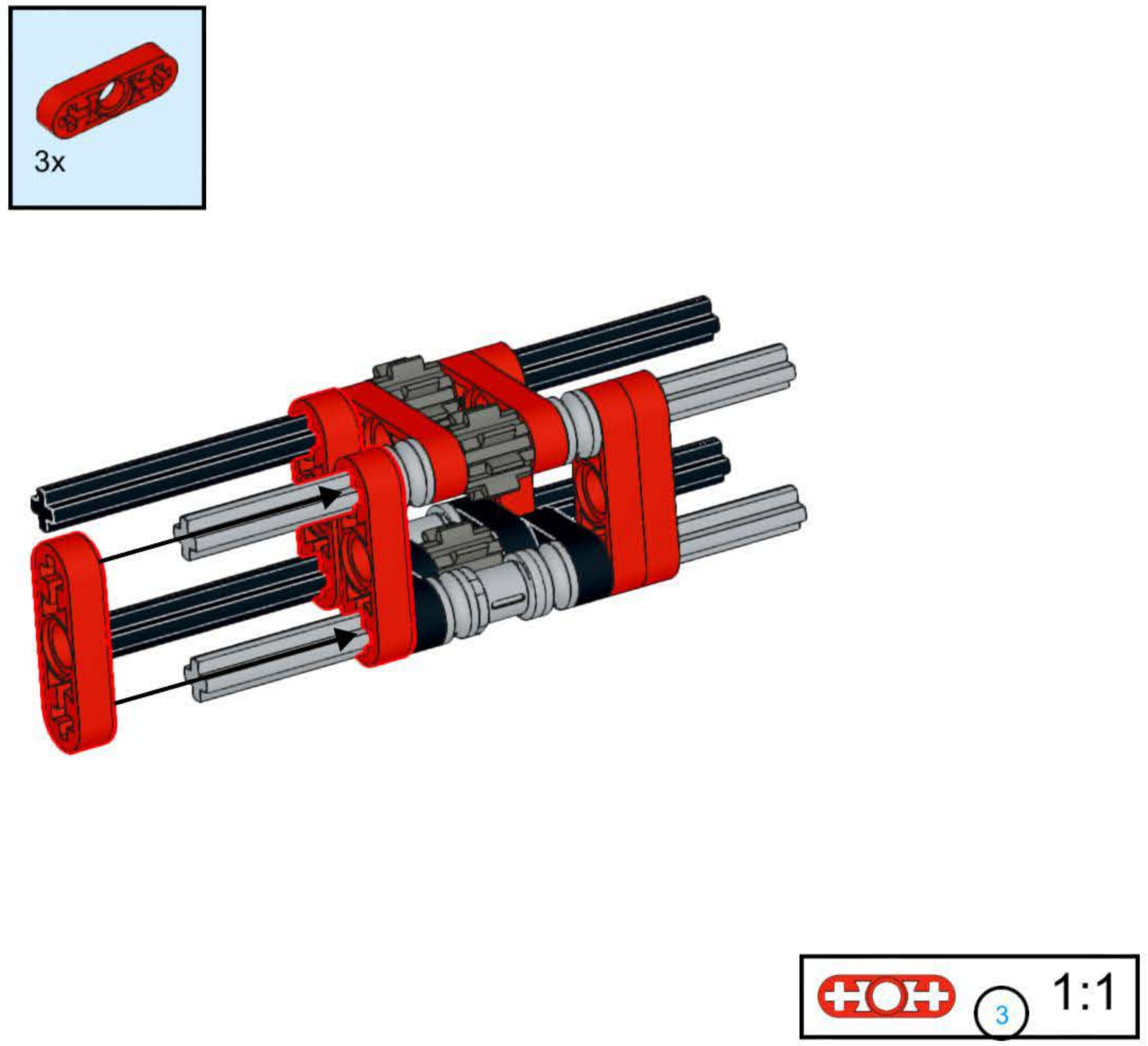


## 67

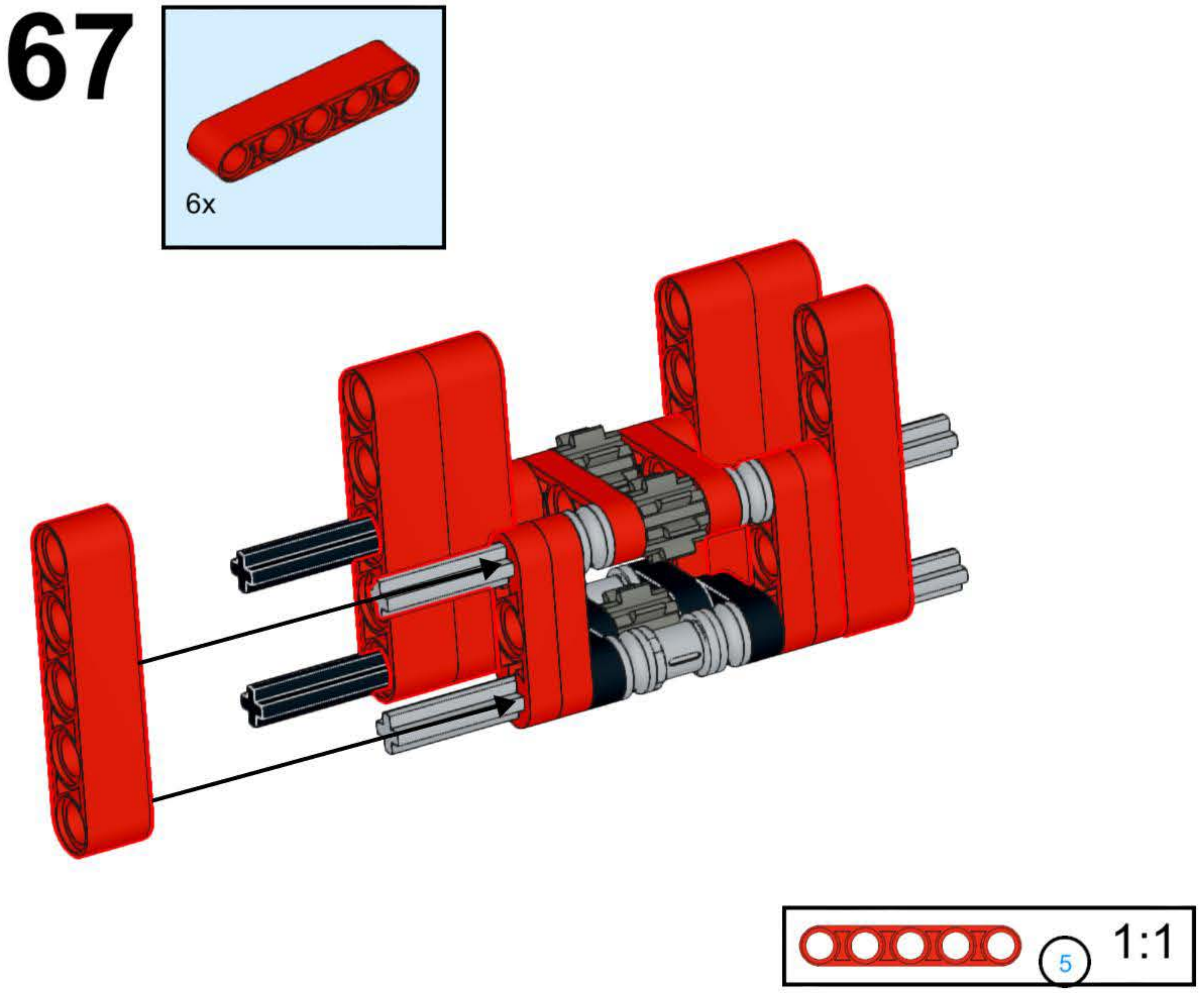


## 68

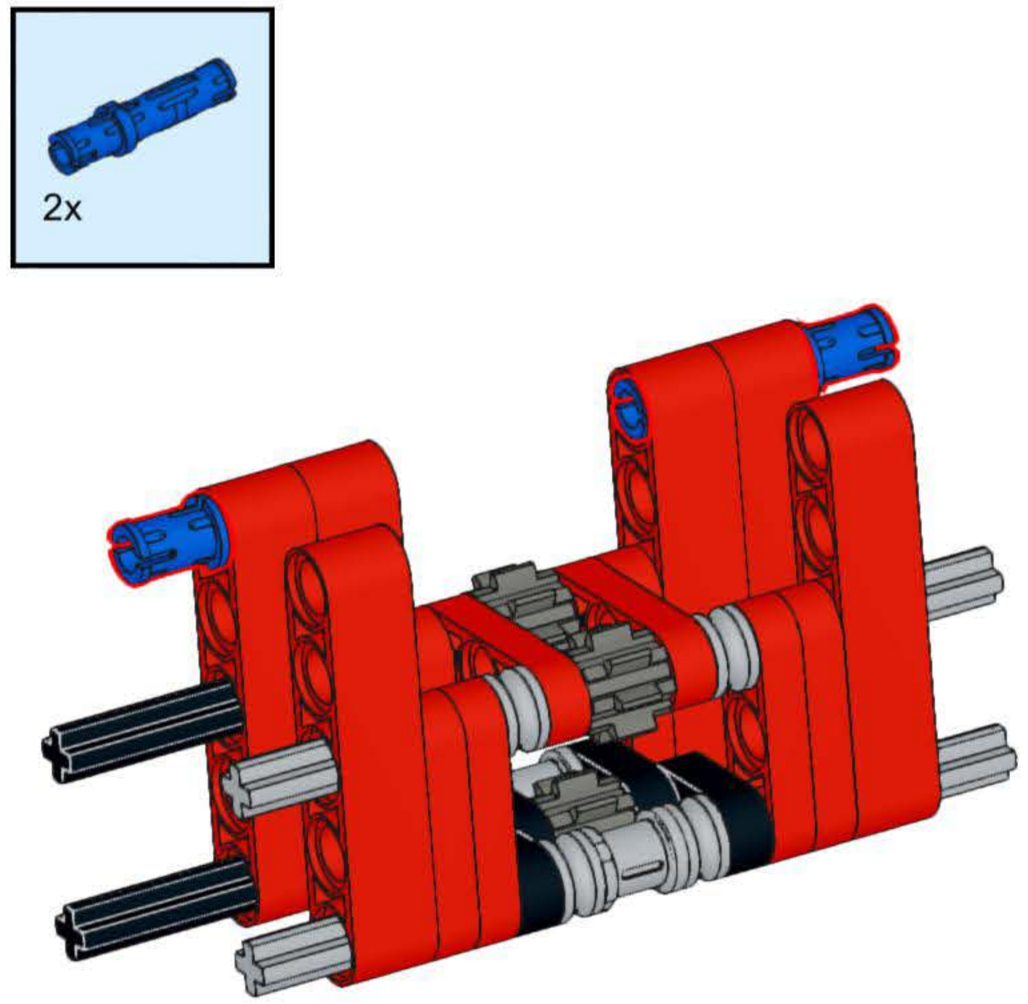


## 69

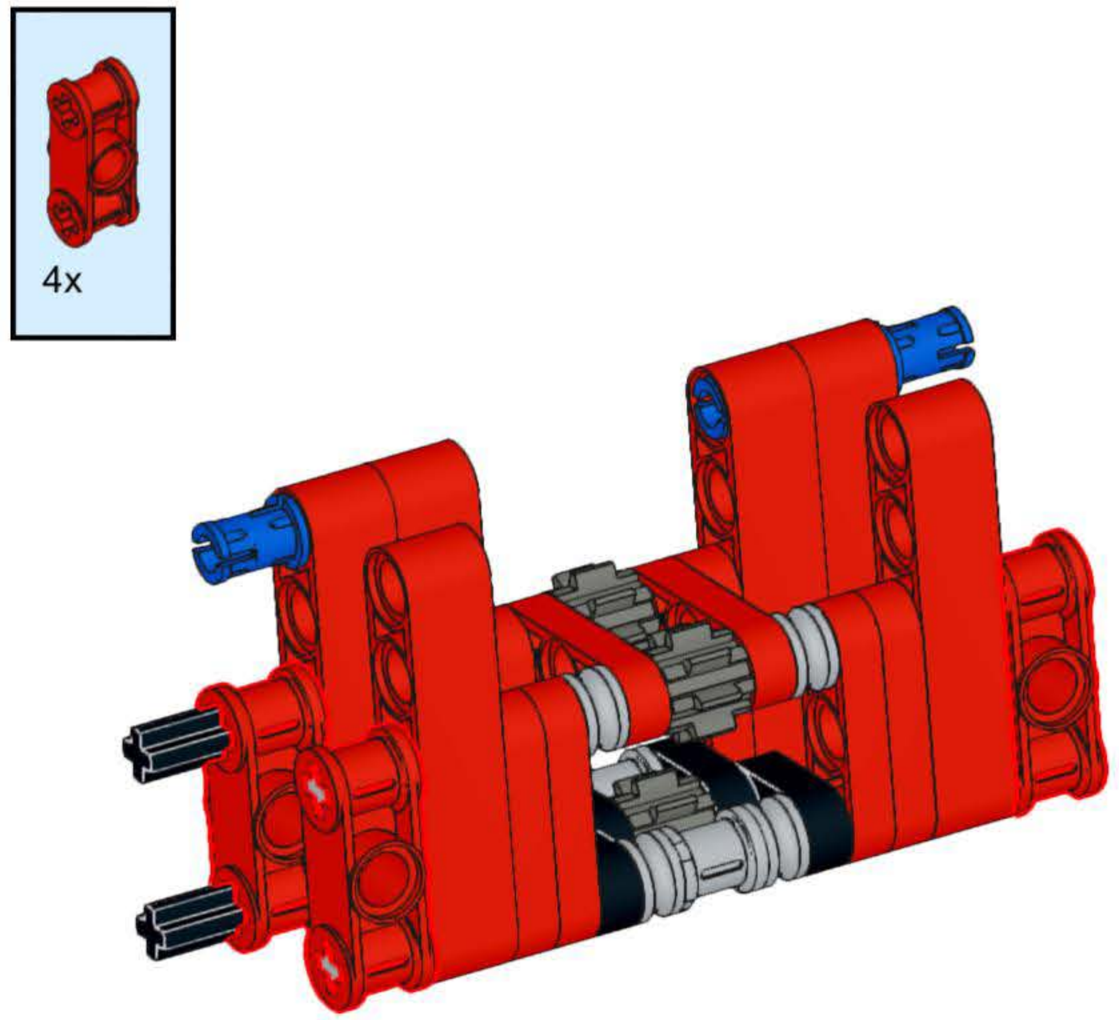

70

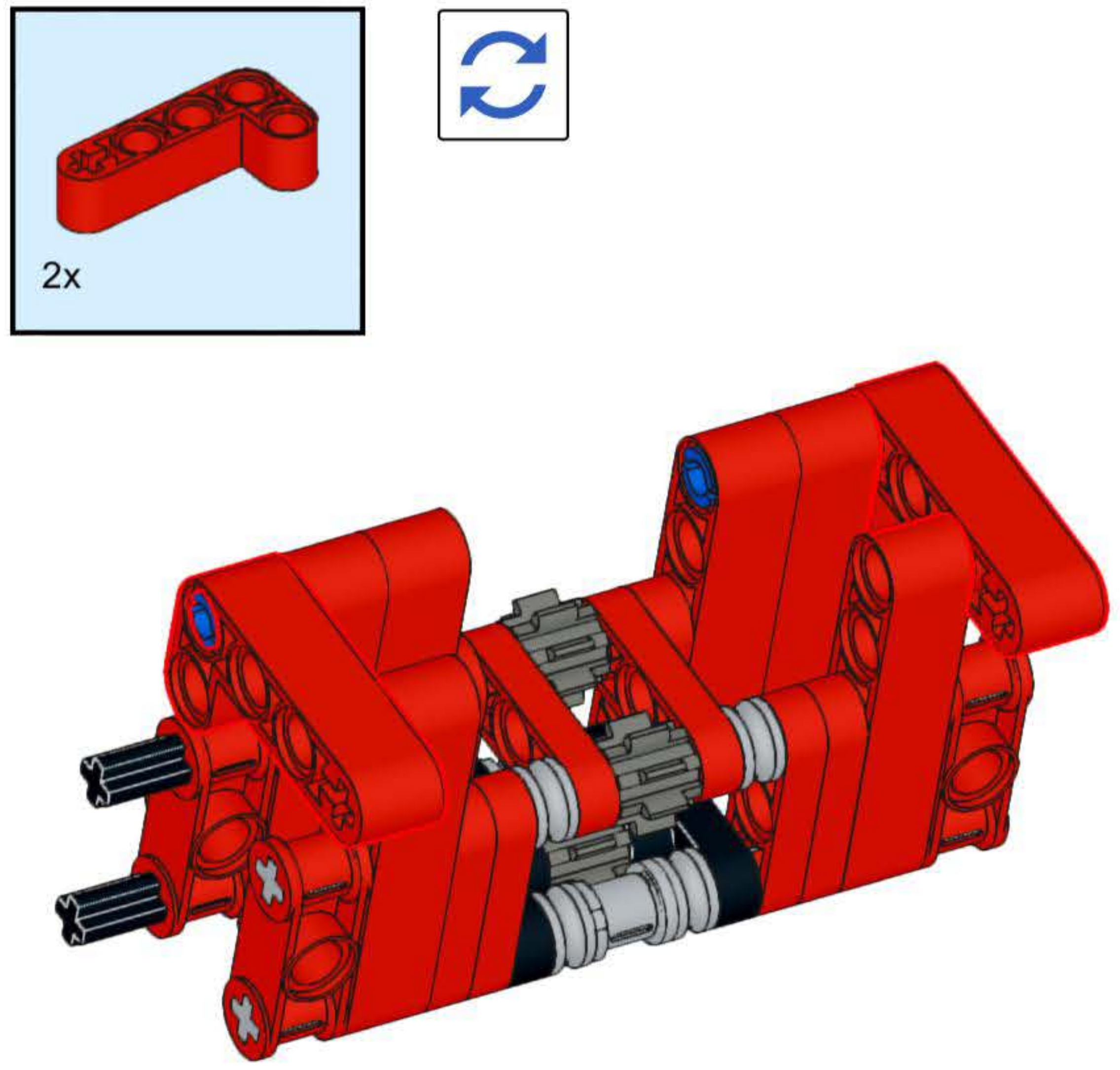


71

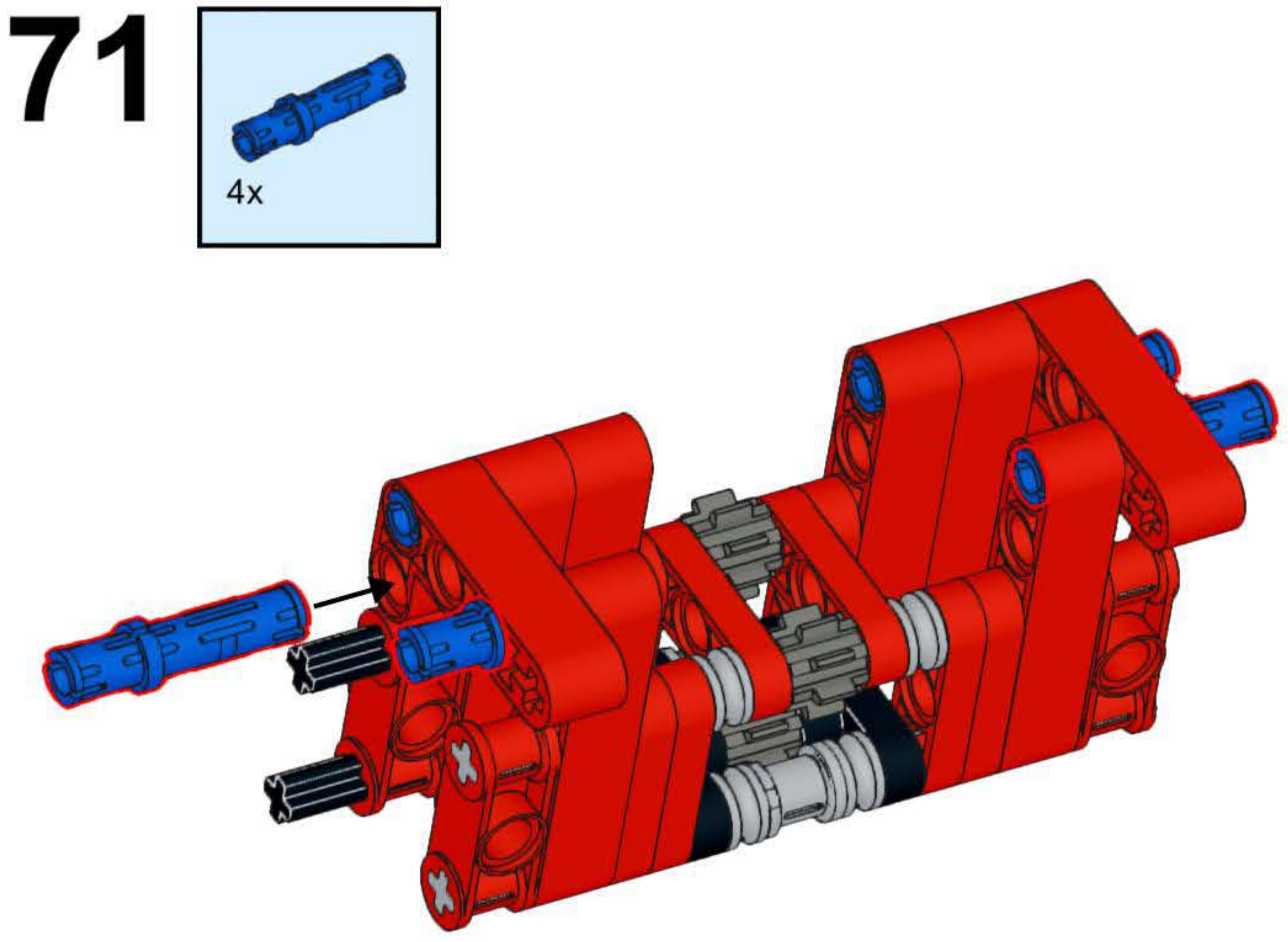


72

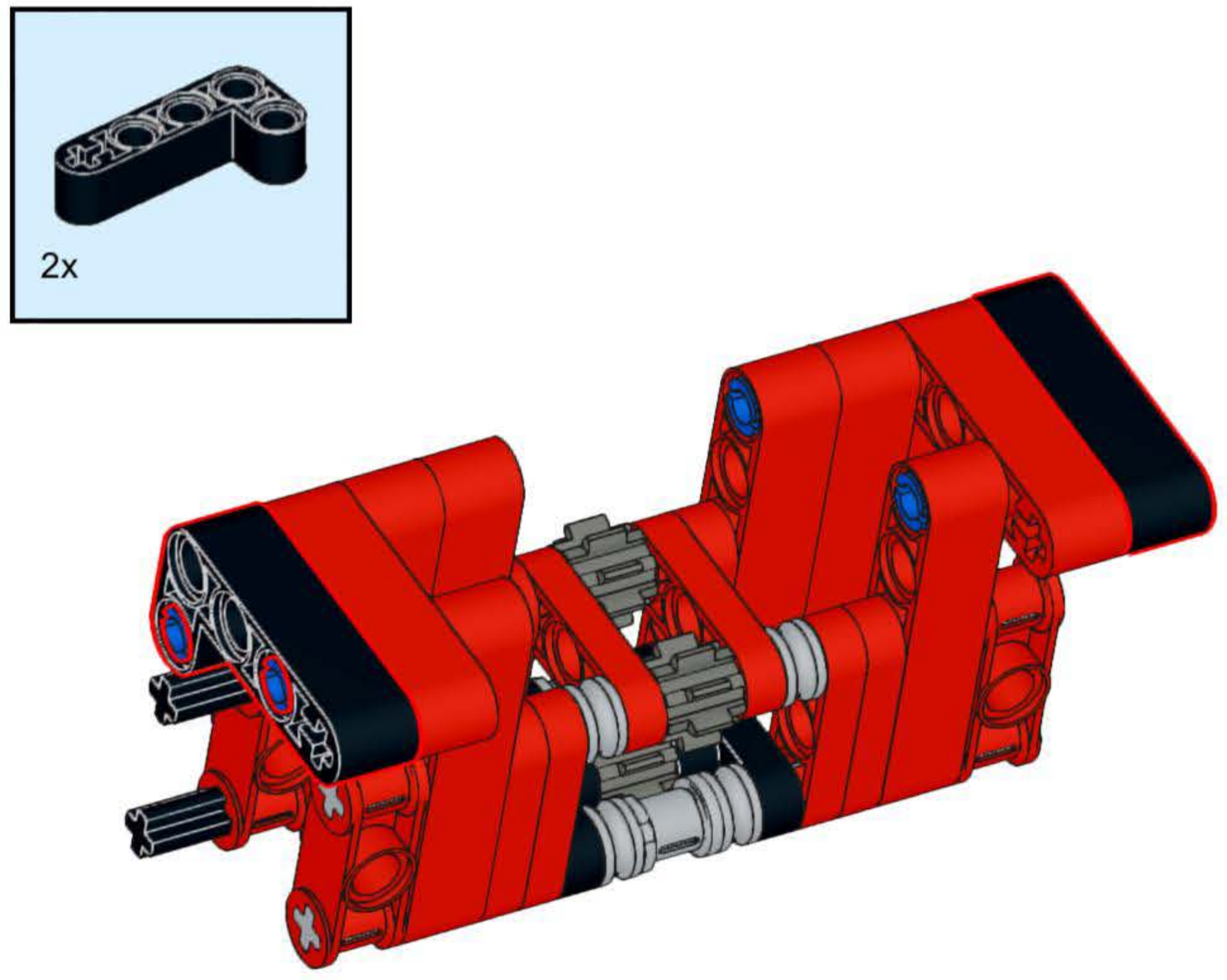


73

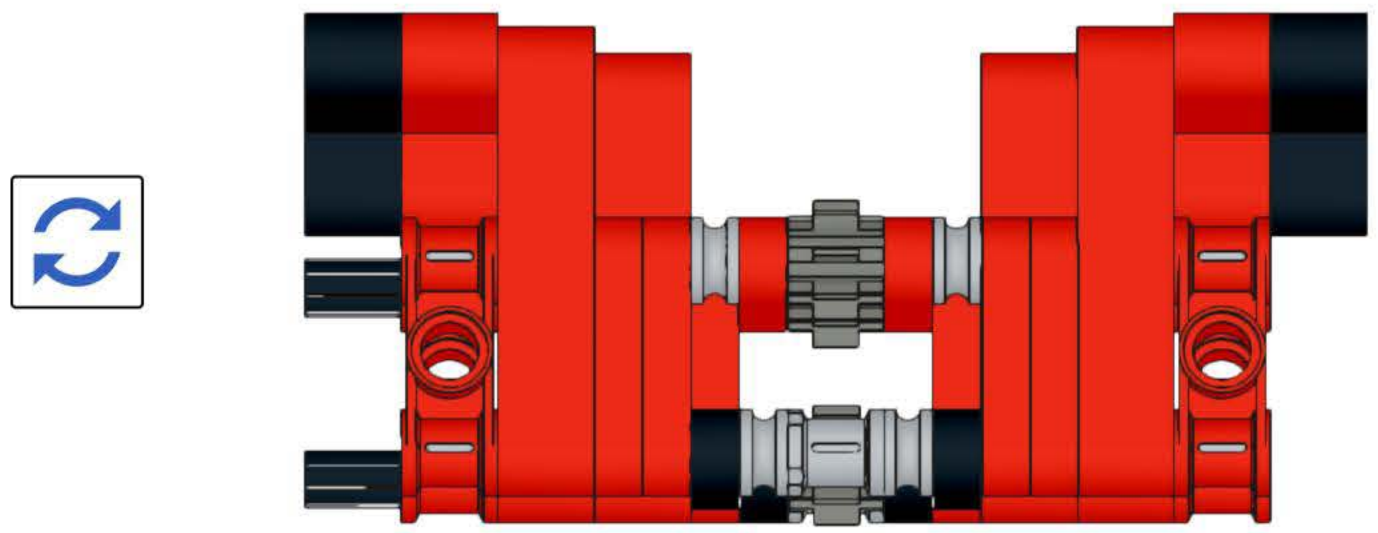

74

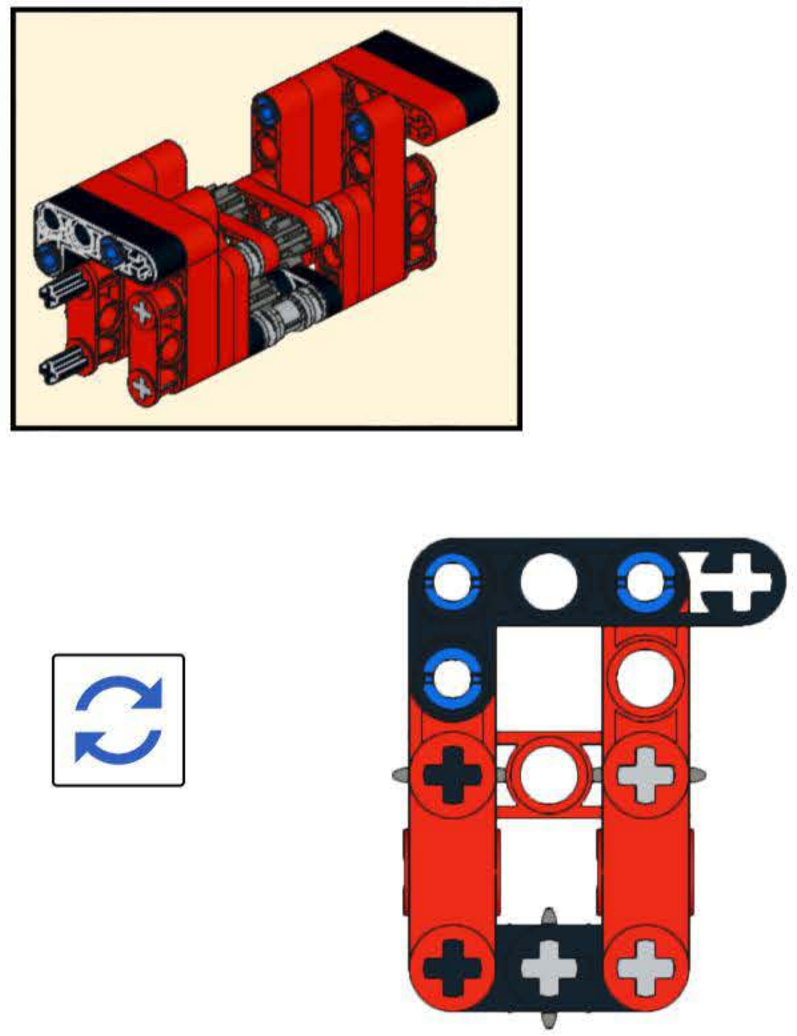

75

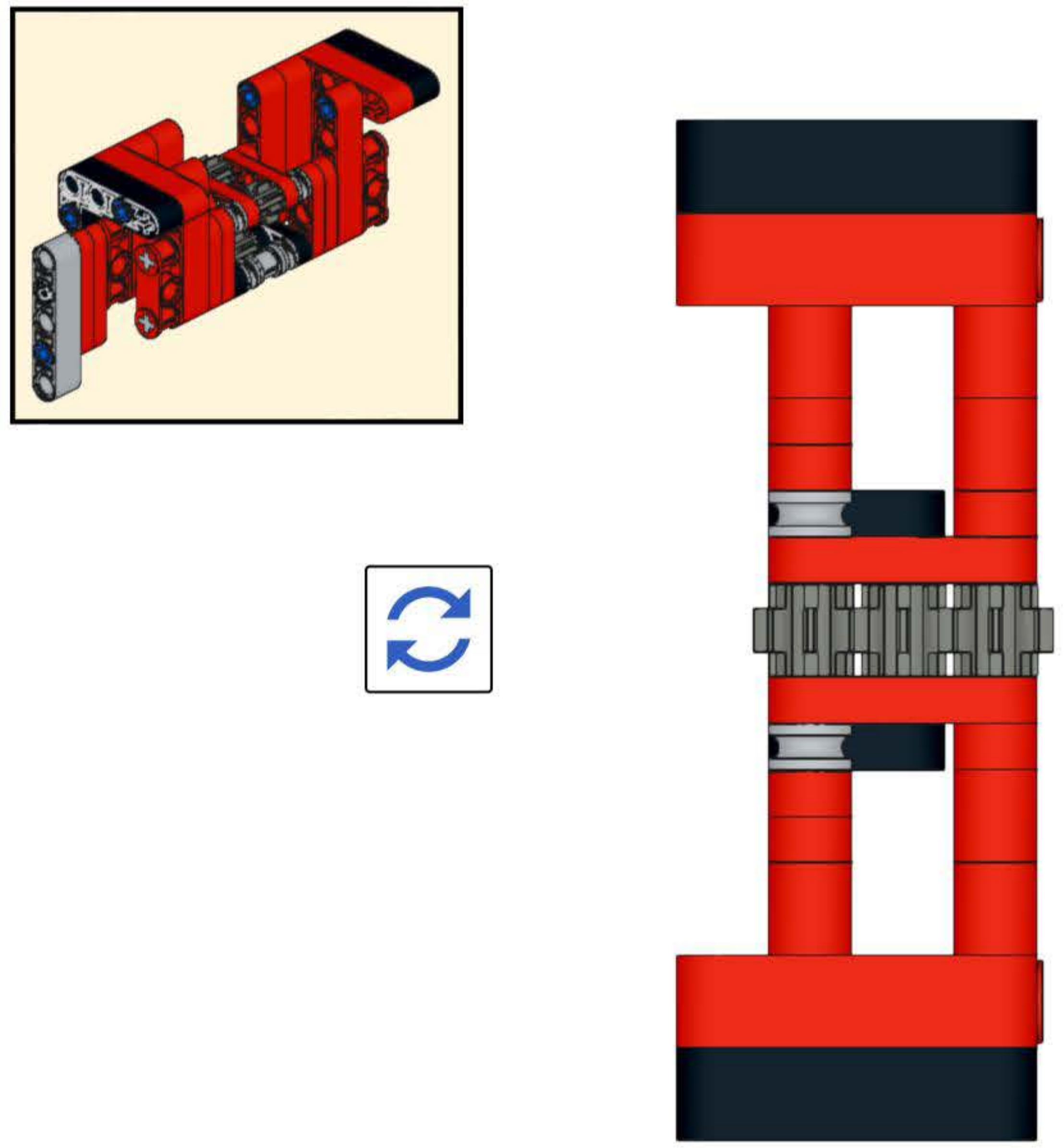

# 76

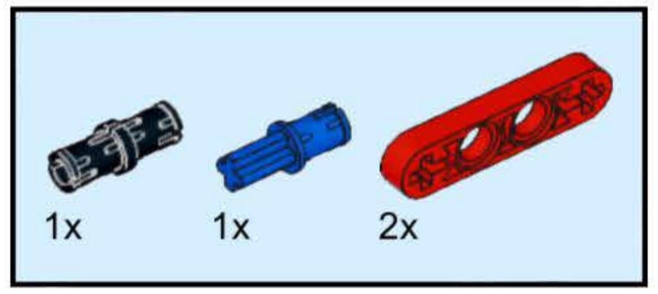


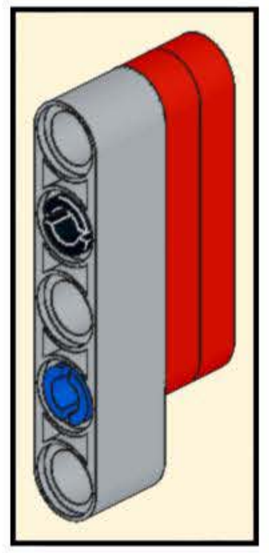

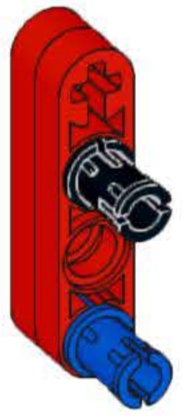

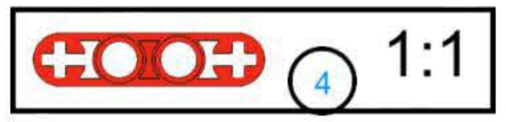


# 77

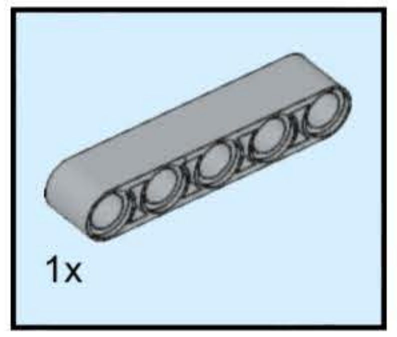


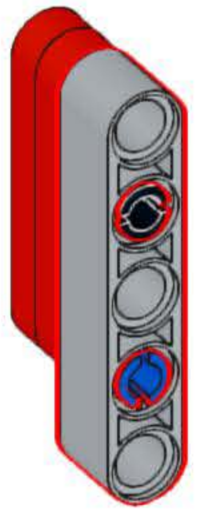

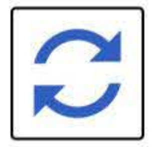

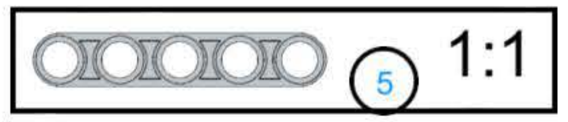


# 78

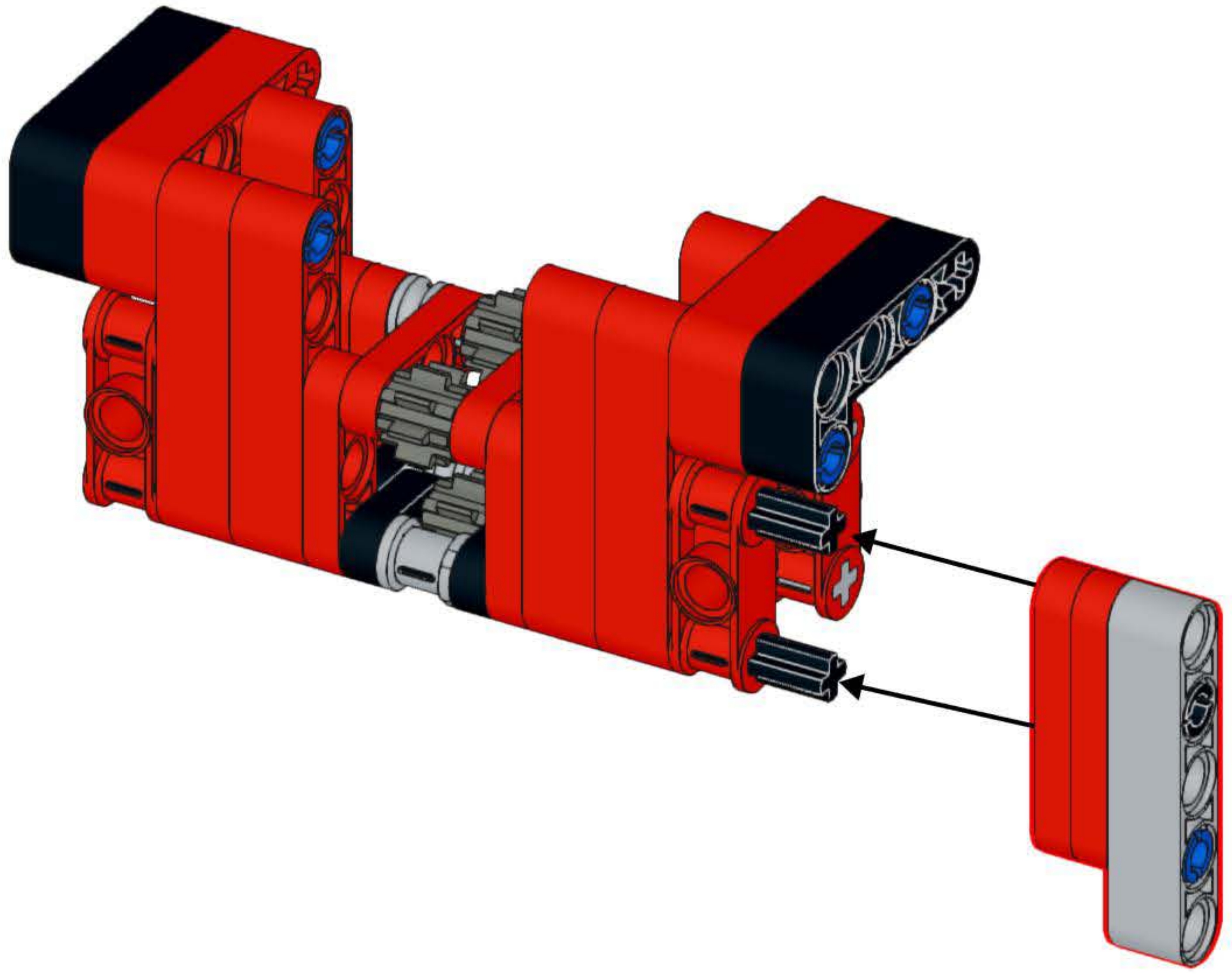

# 79

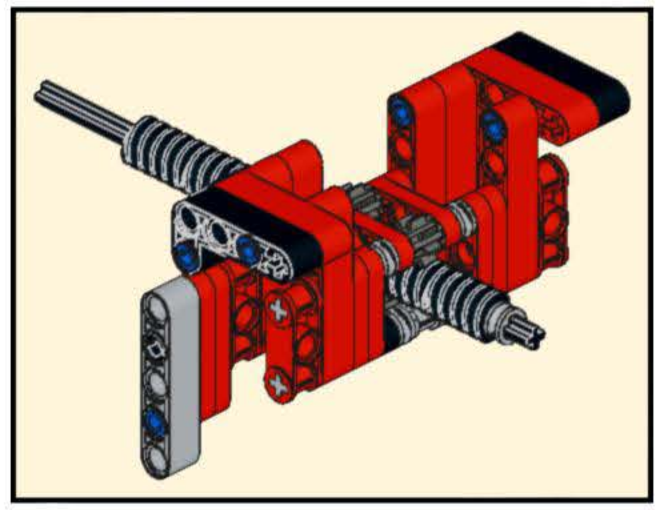

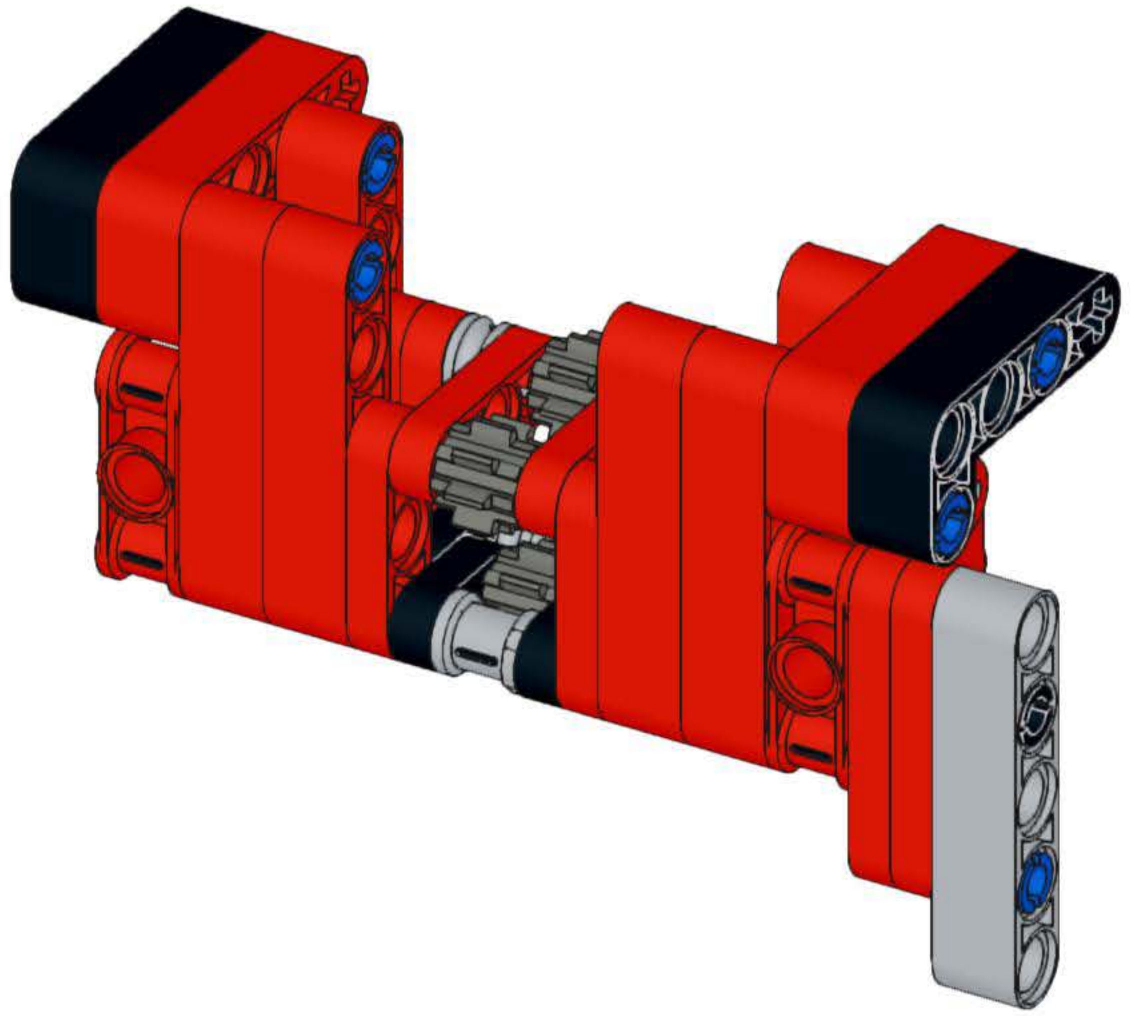

# 80

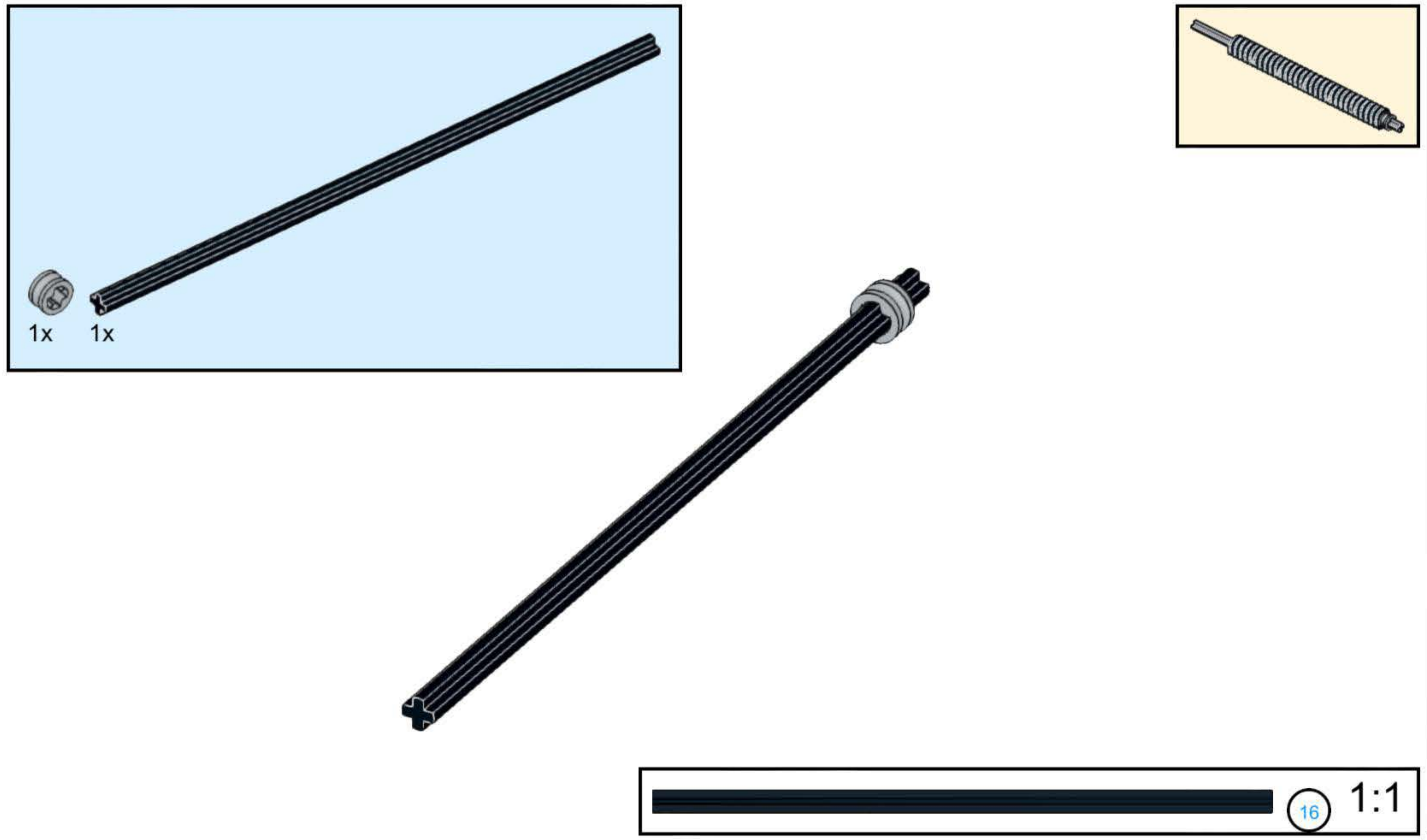


# 81

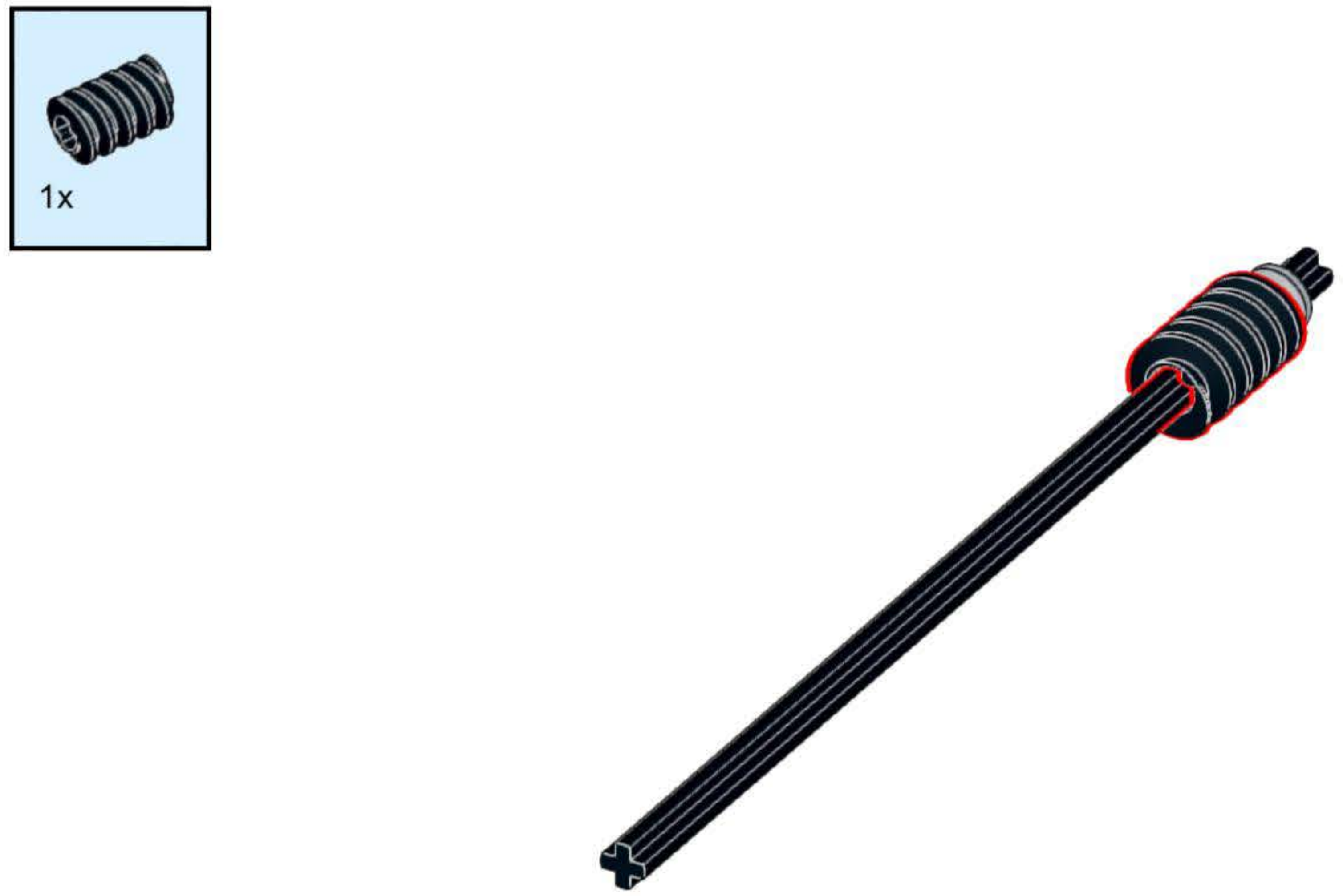


# 82

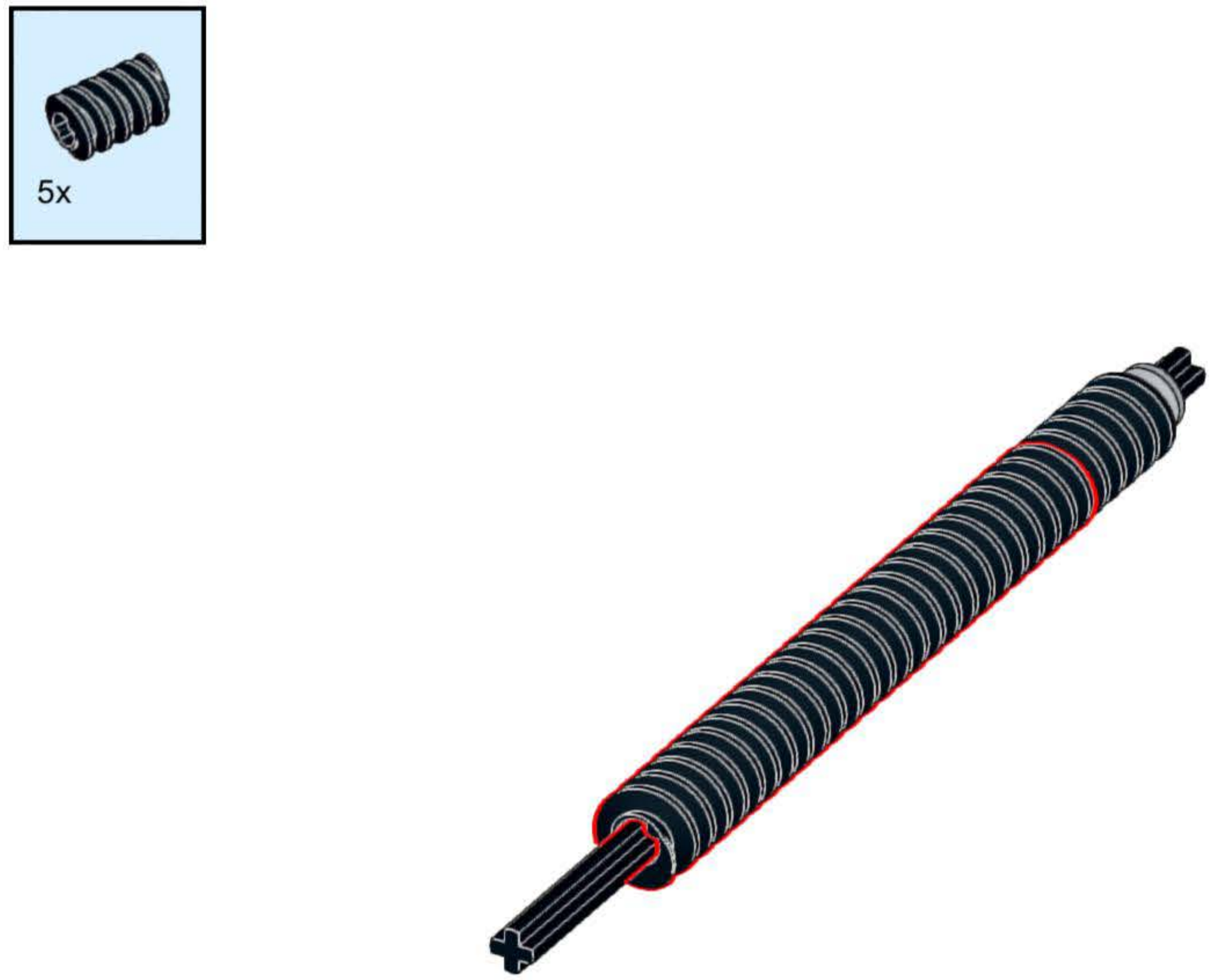


# 83

Worm screw threads must be aligned from one to the next, making one continuous worm screw.

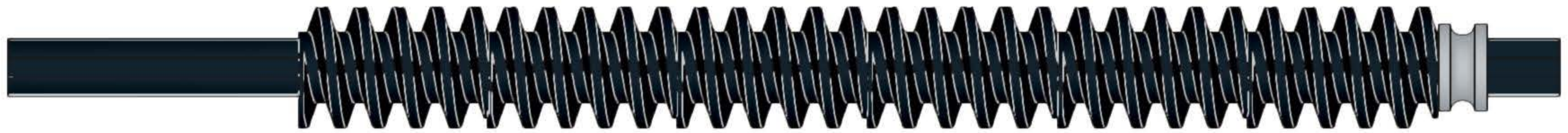

84

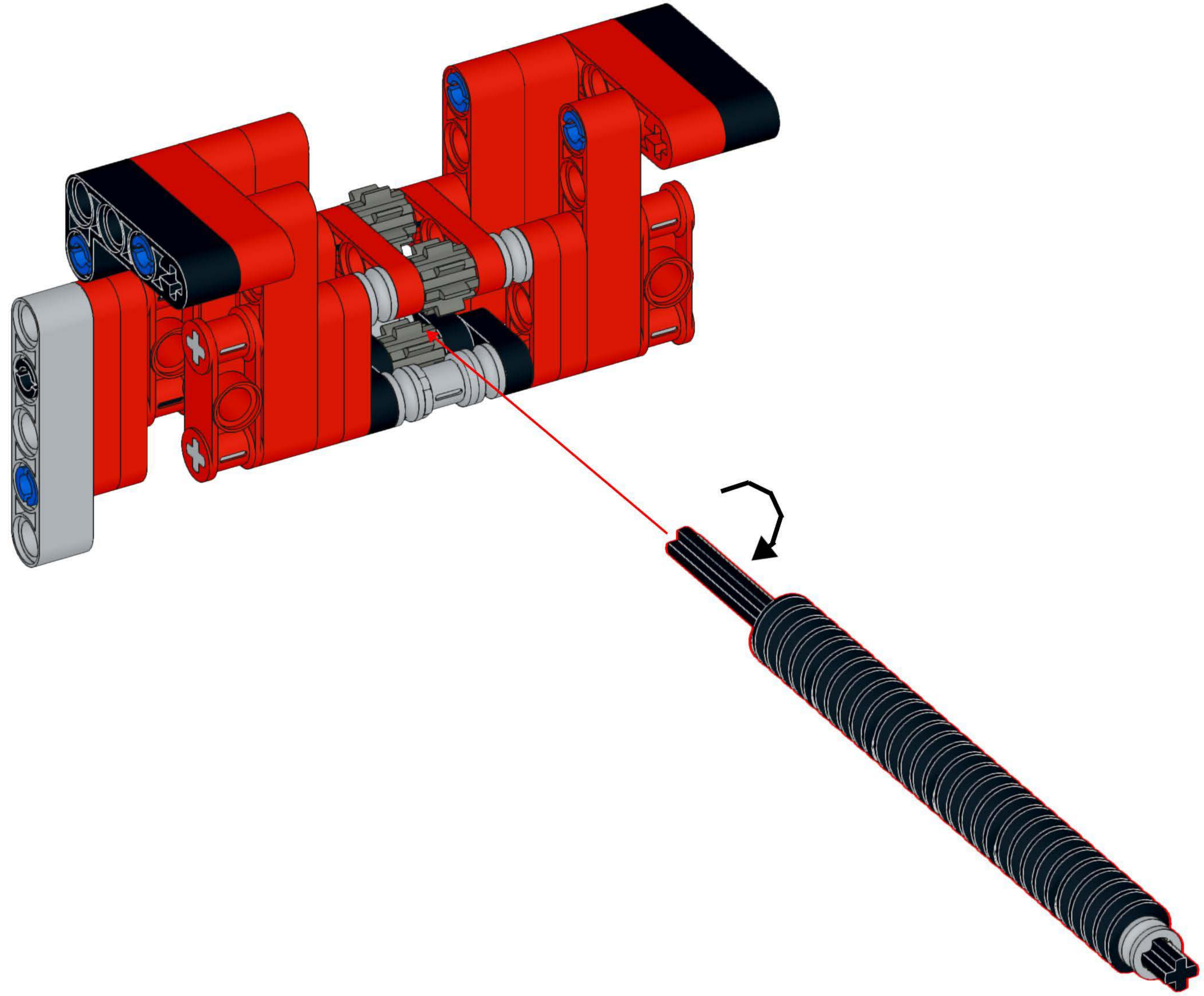

85

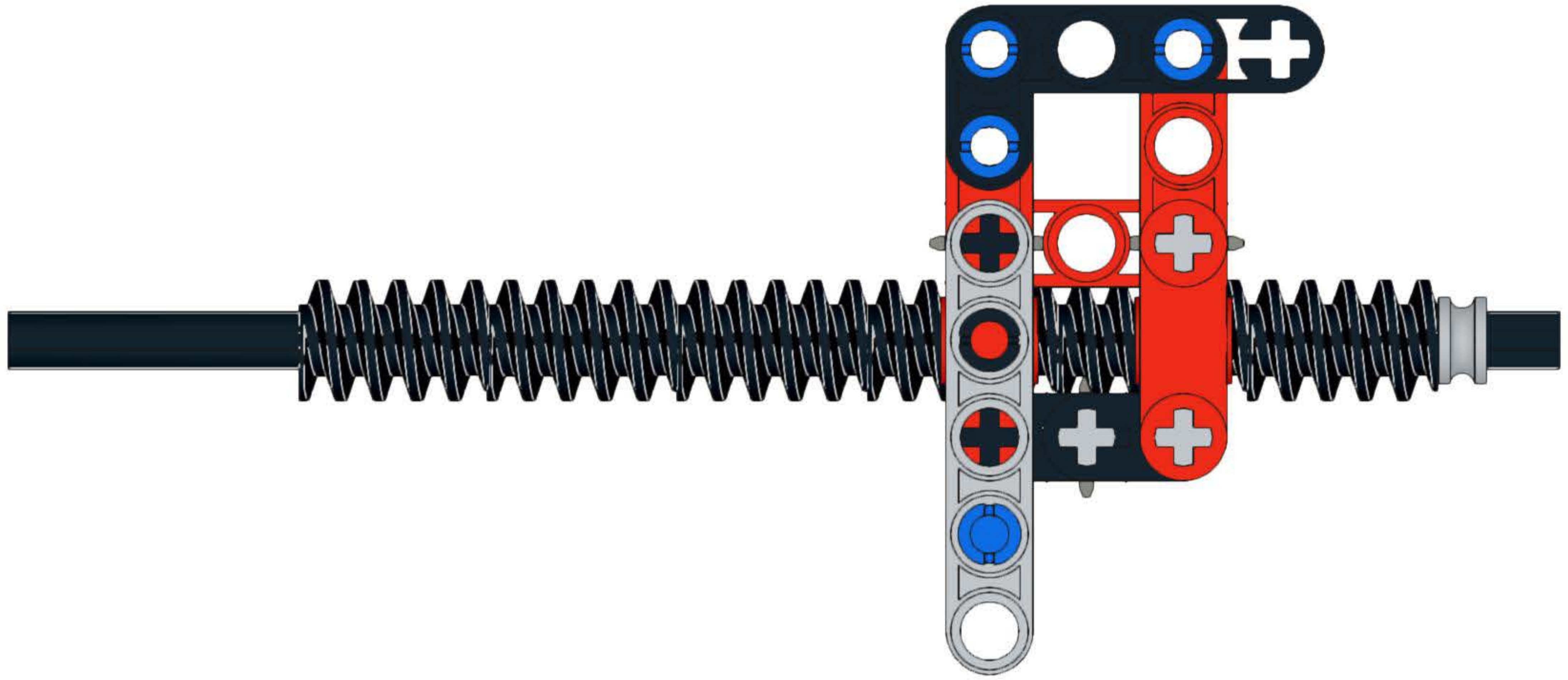

86

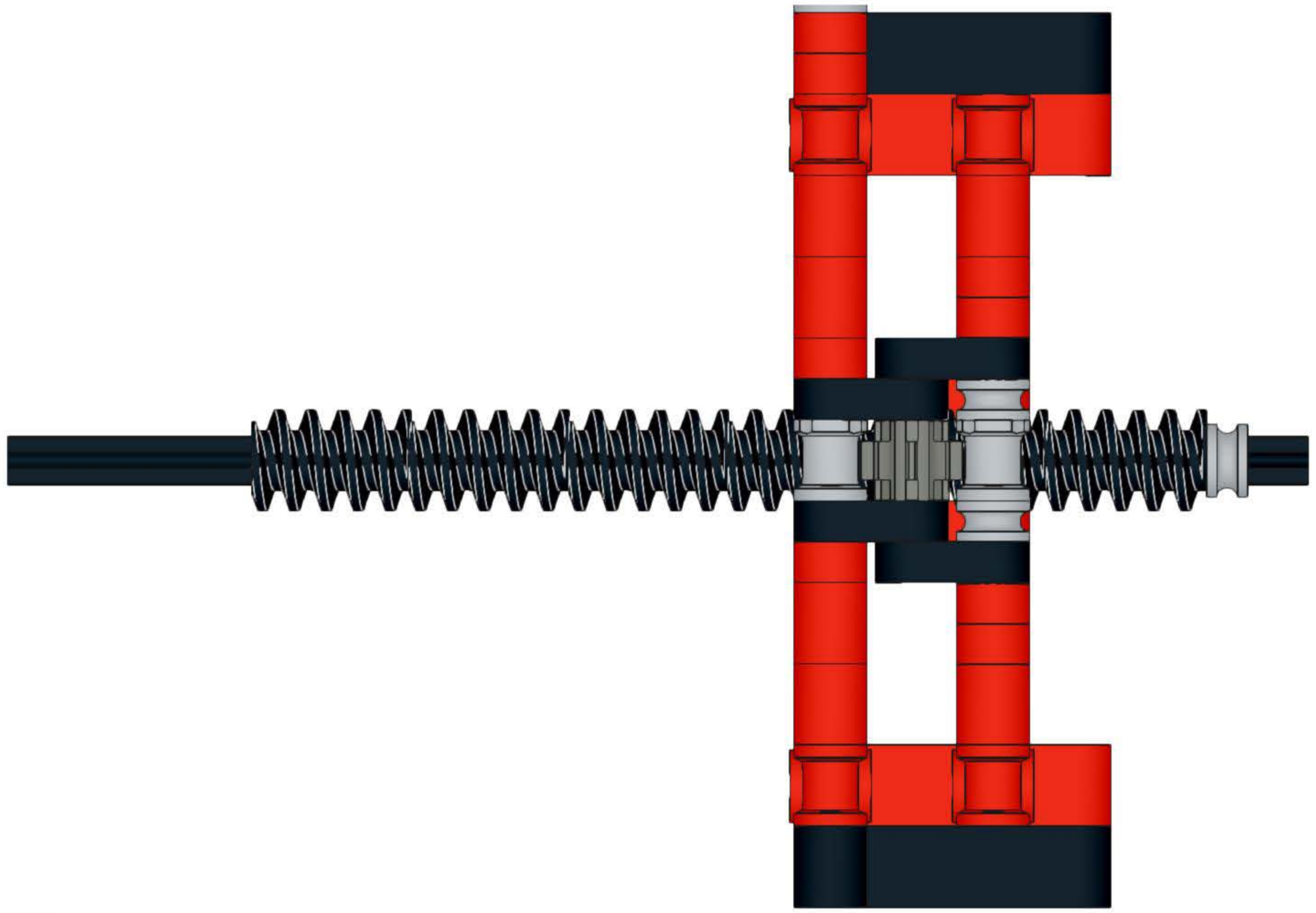

87

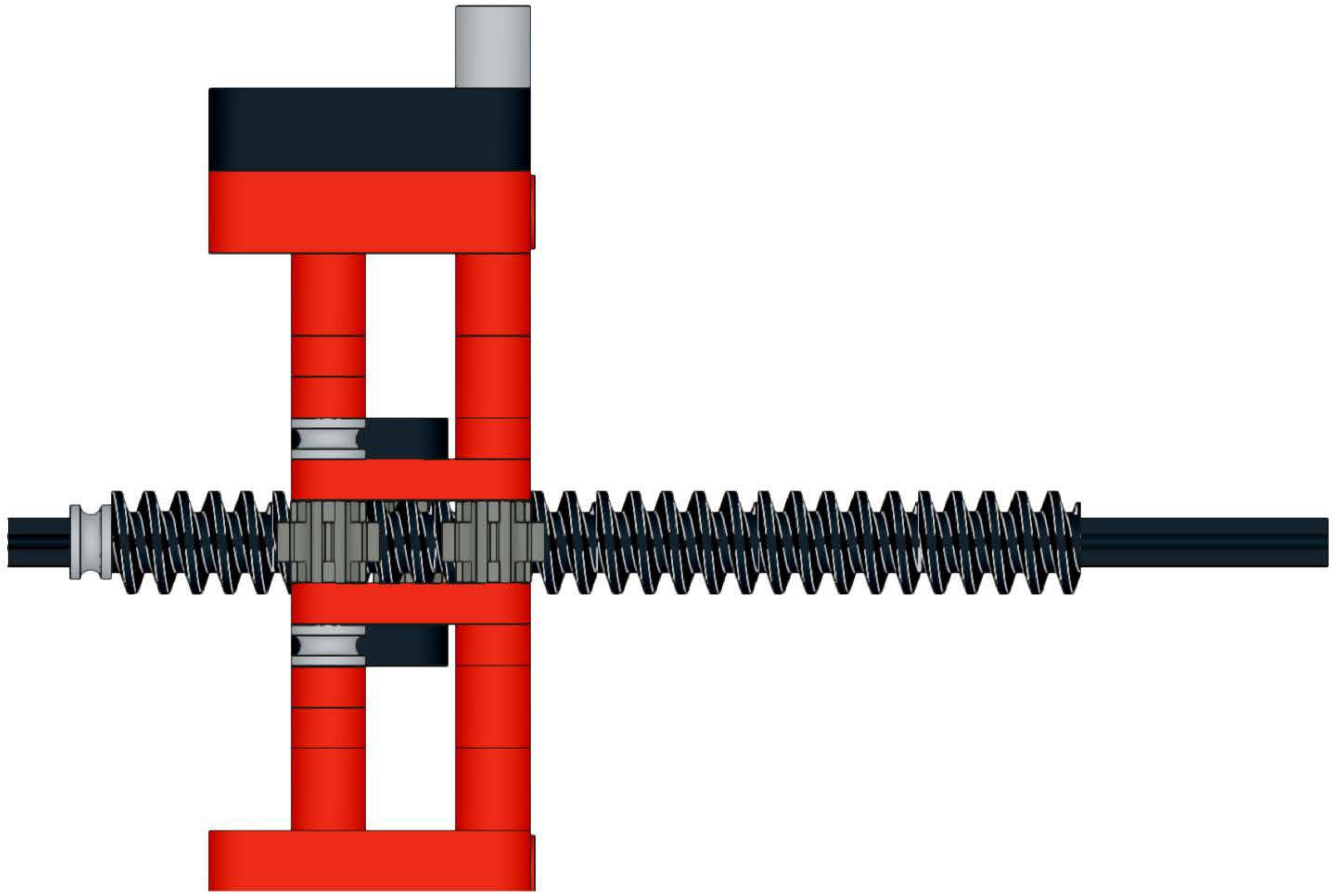

88

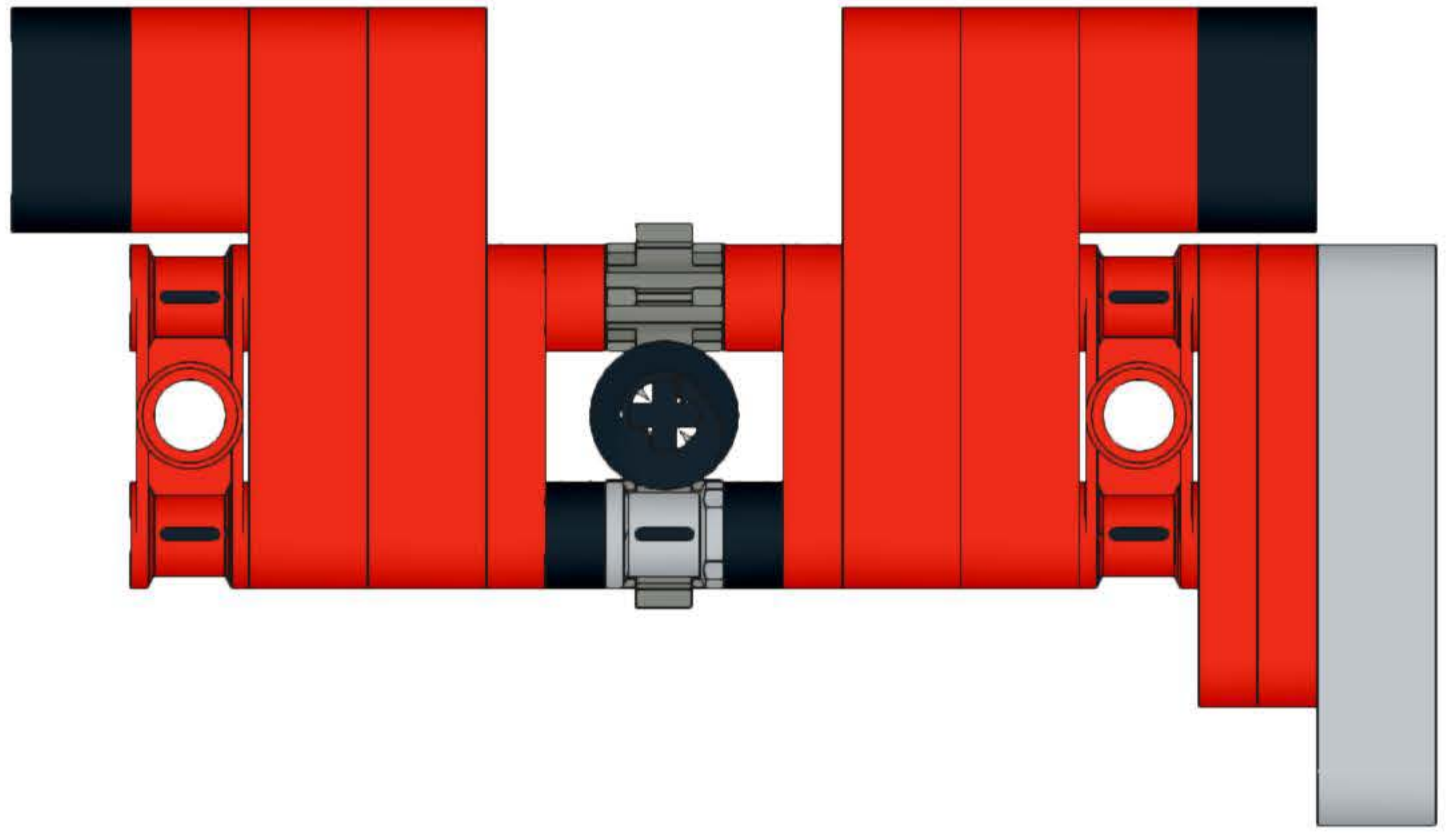

89

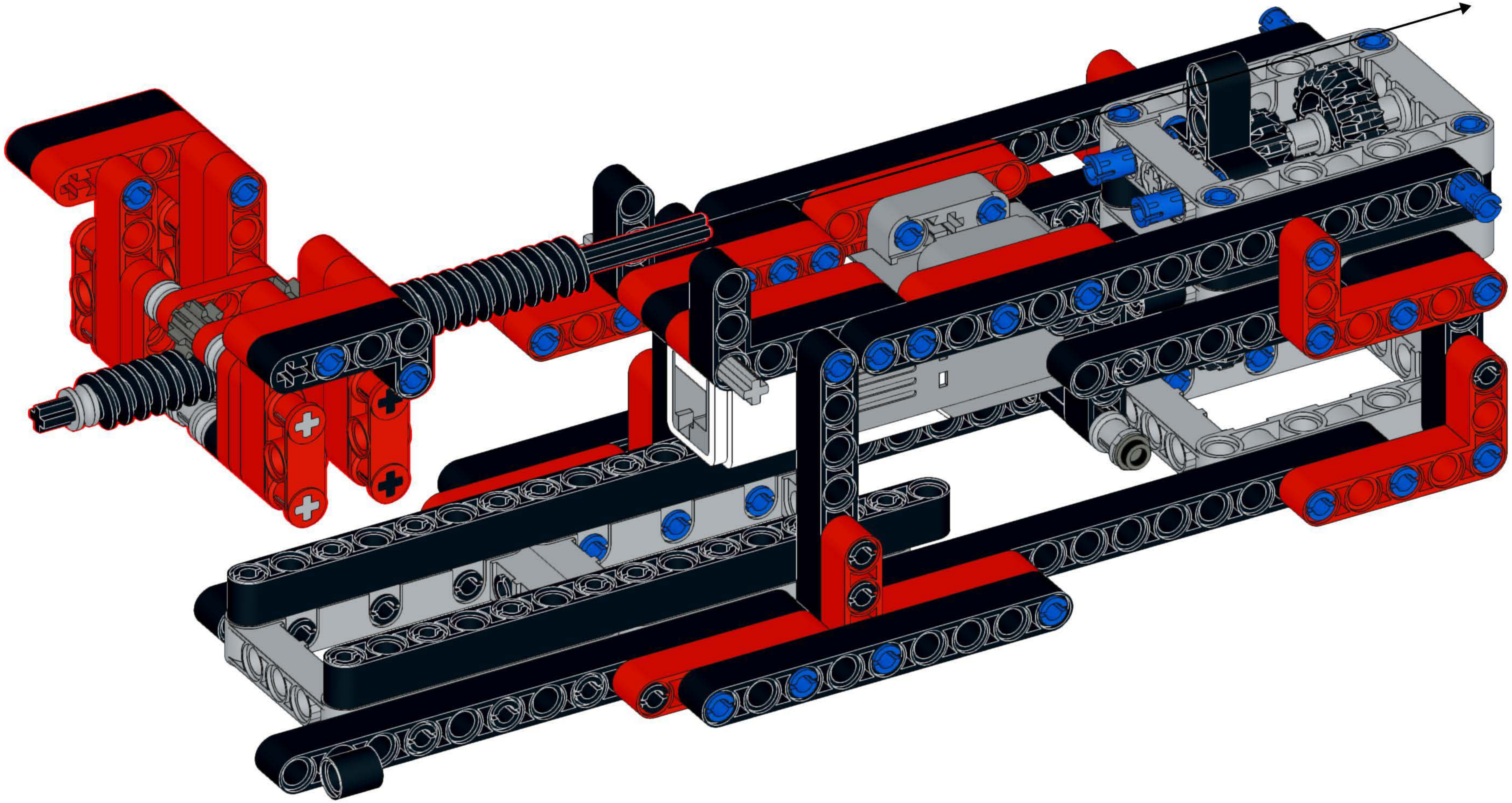

# 90

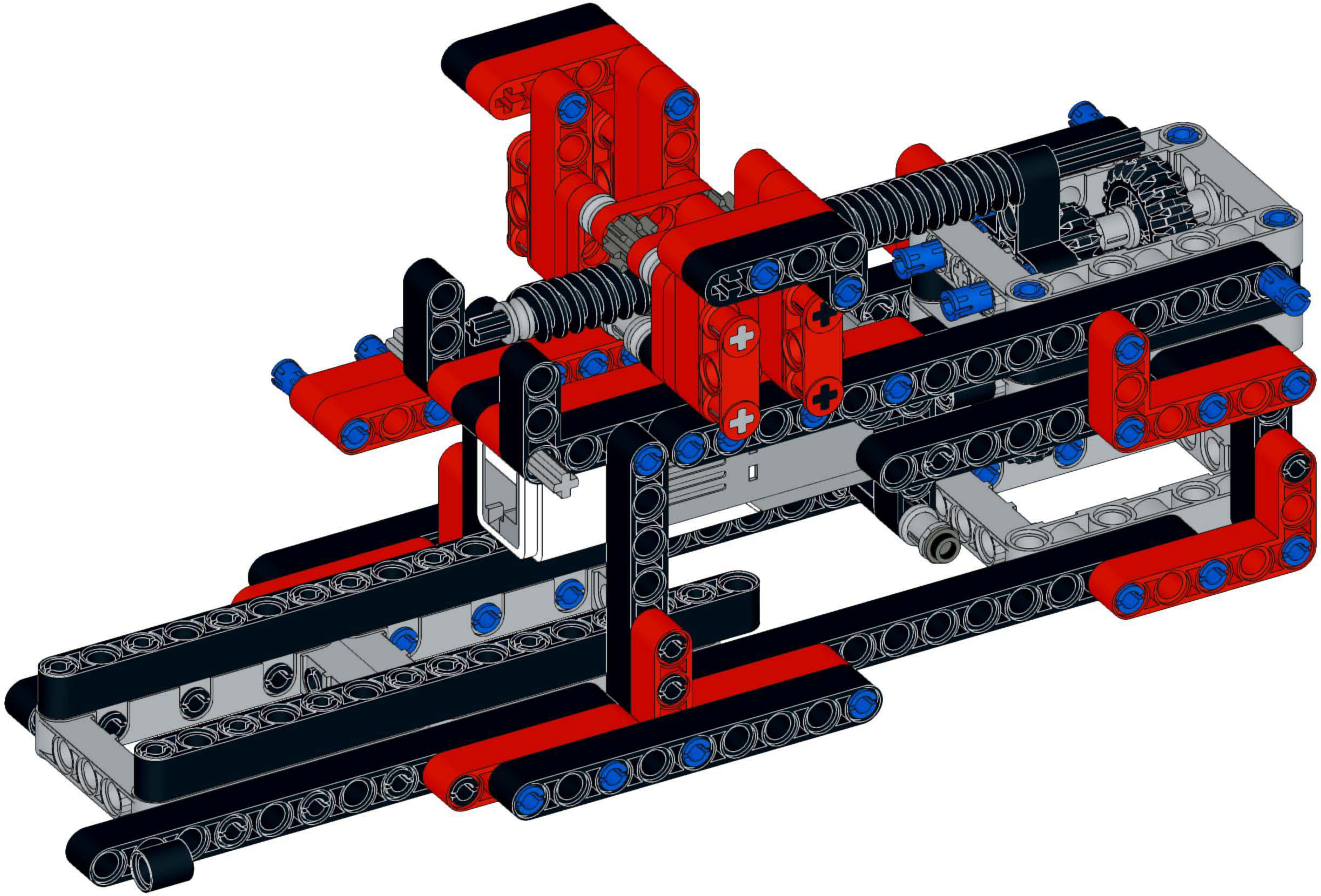

**91**

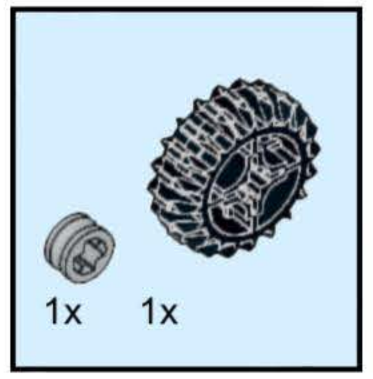


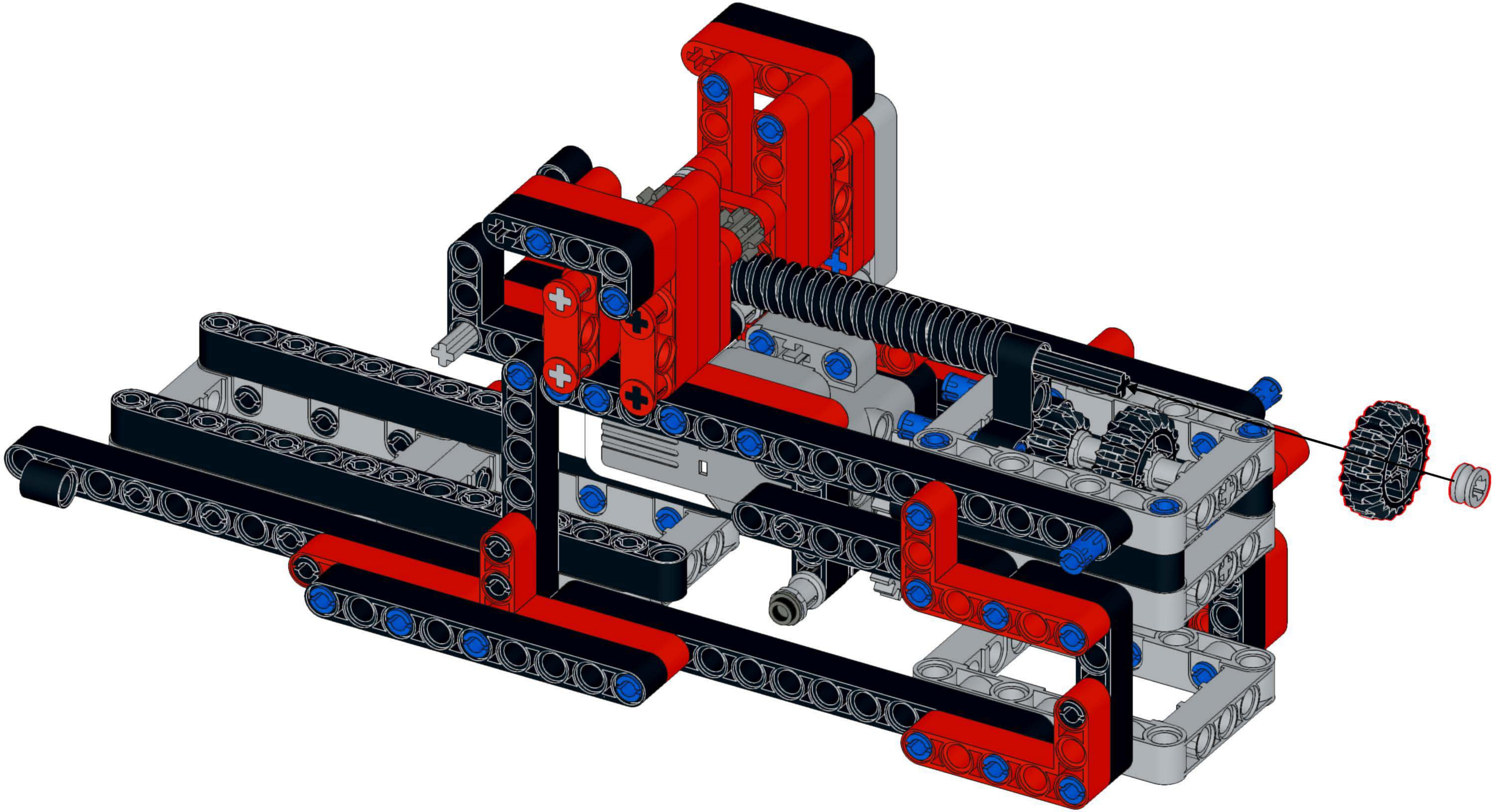

92

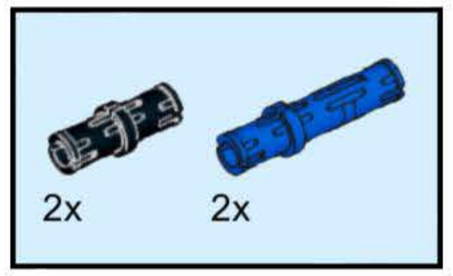


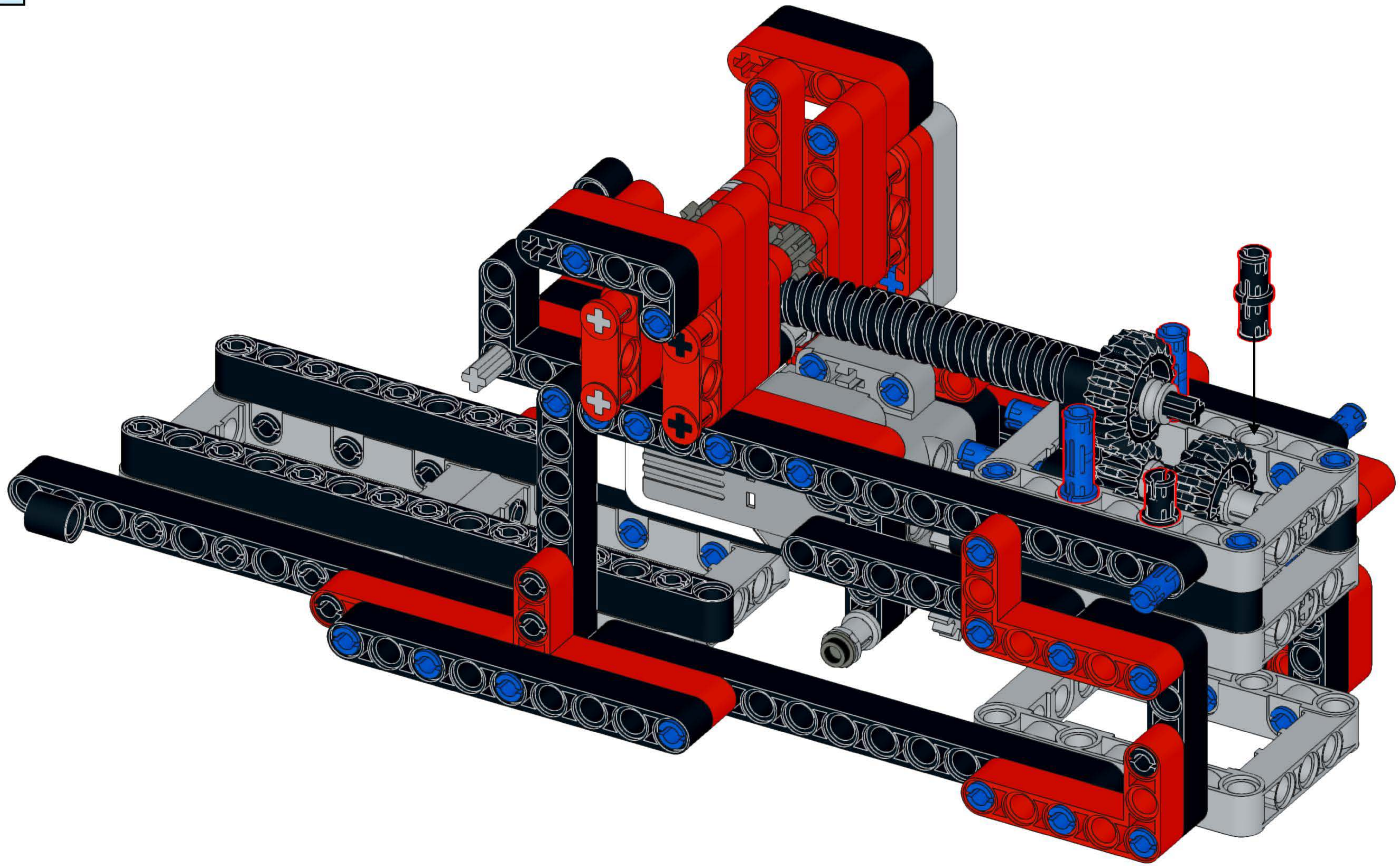

93

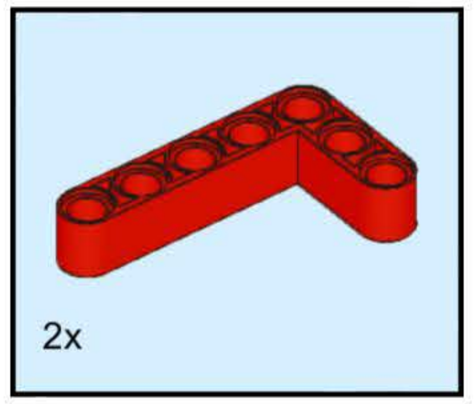


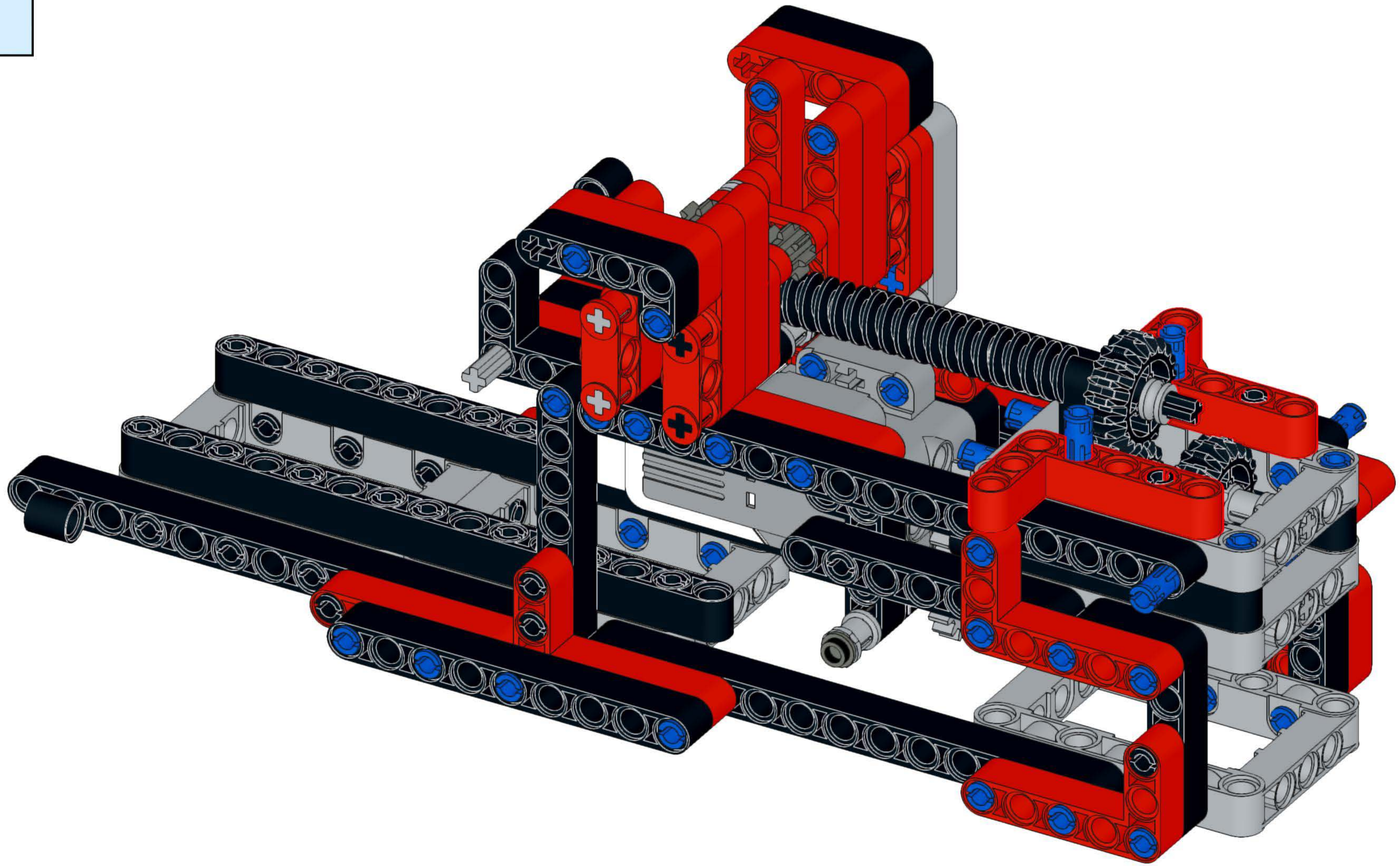

94

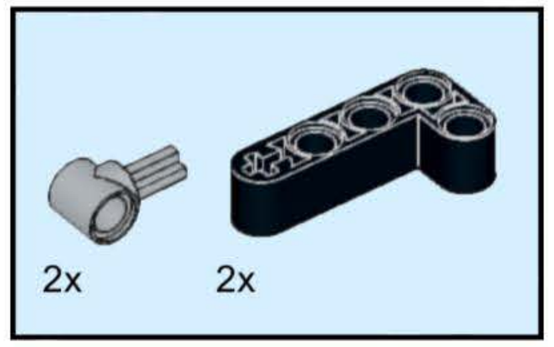


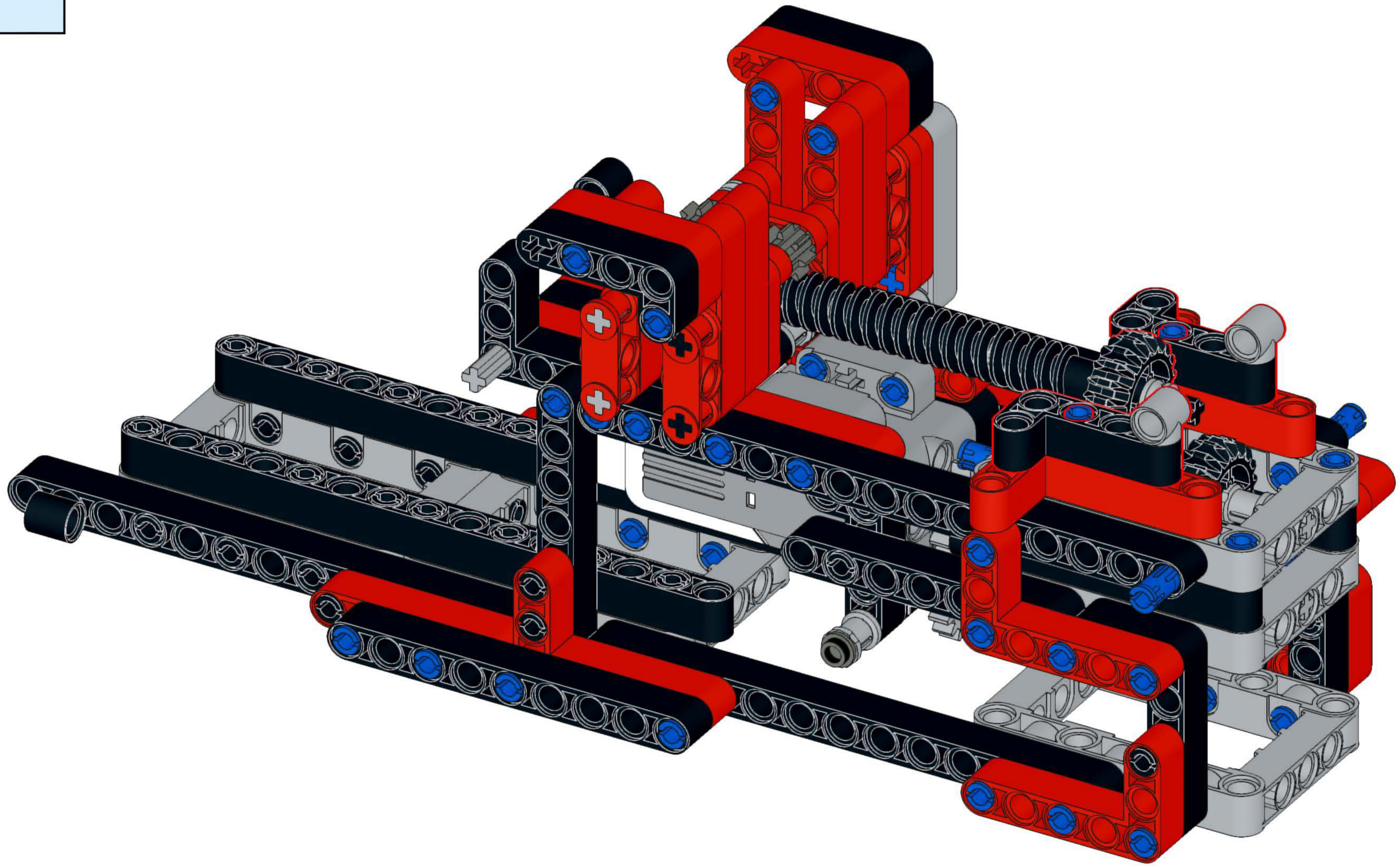

95

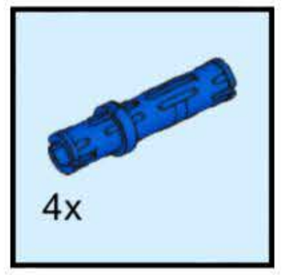


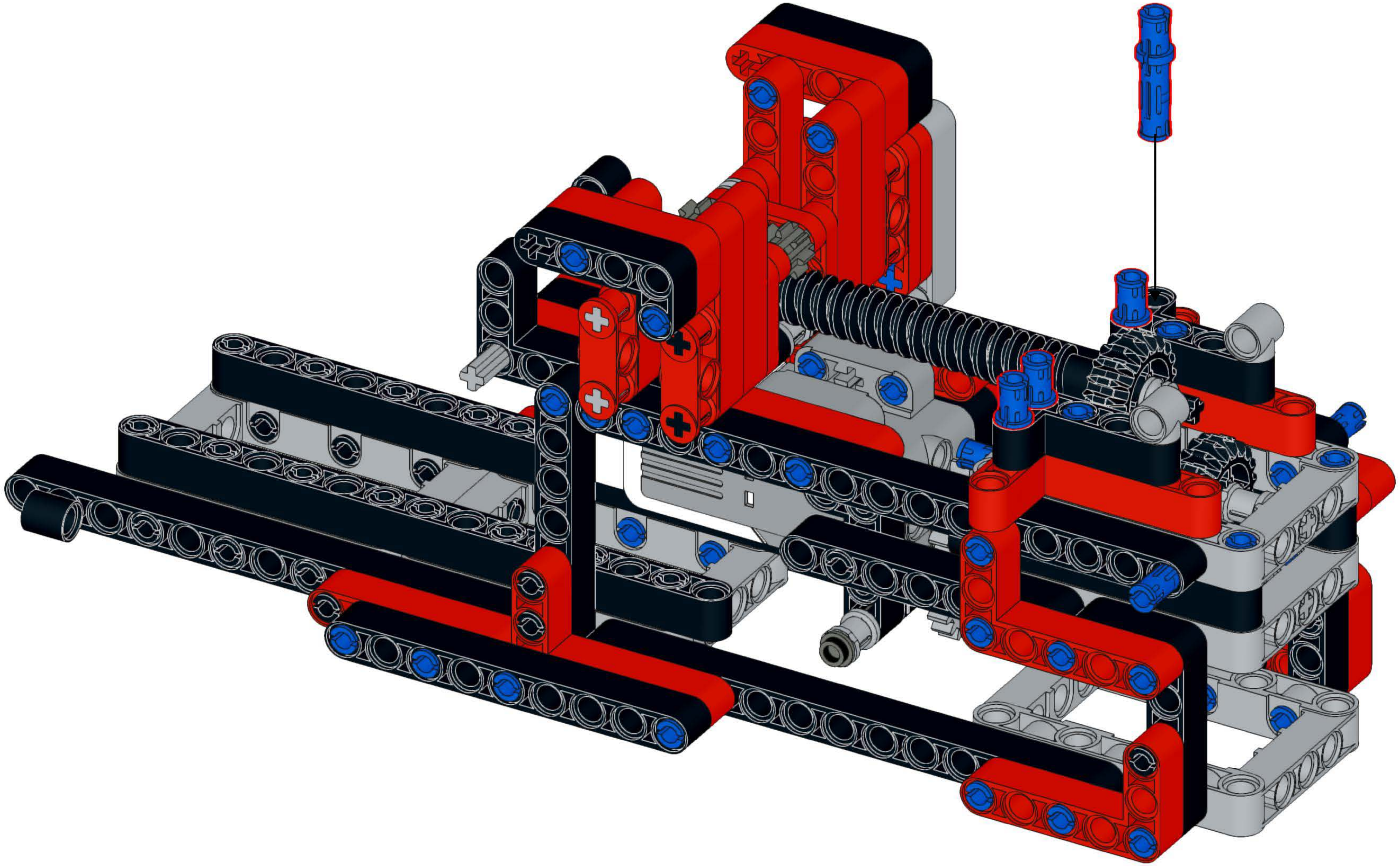

96

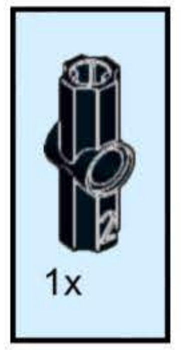


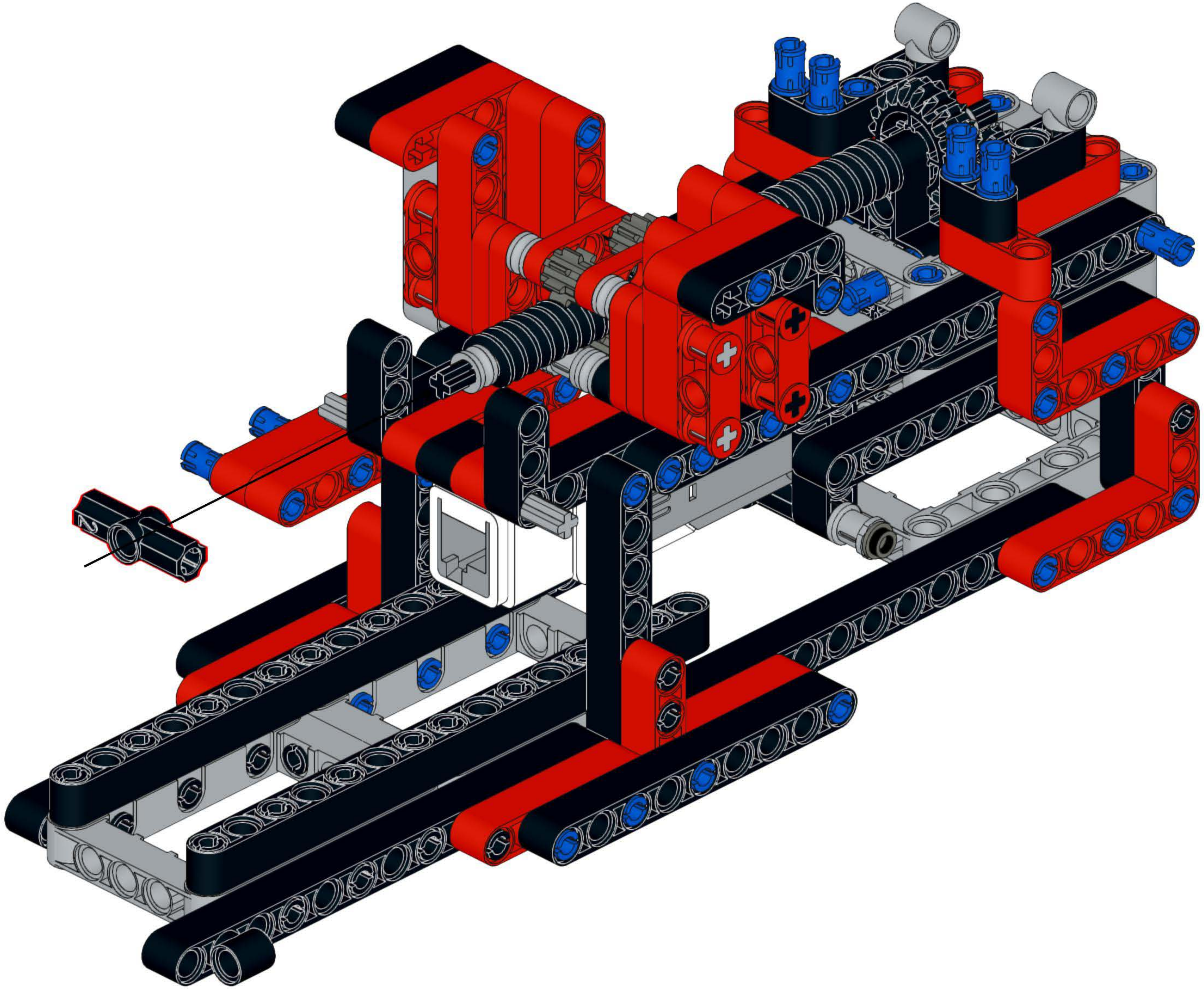

97

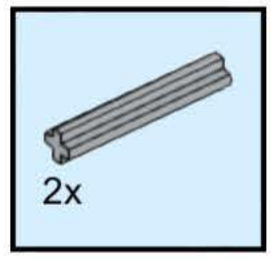


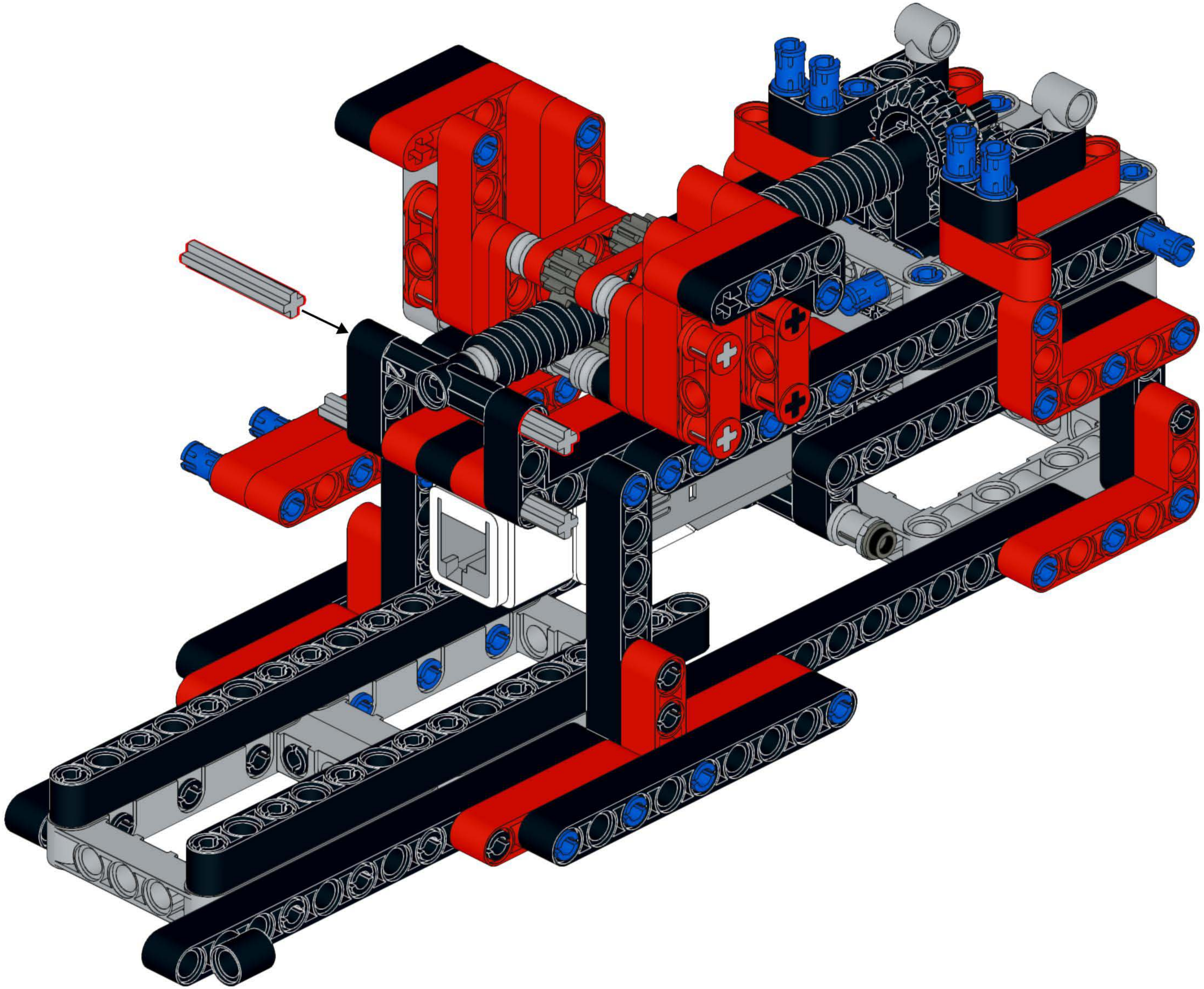

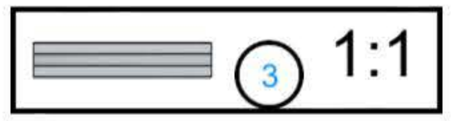

98

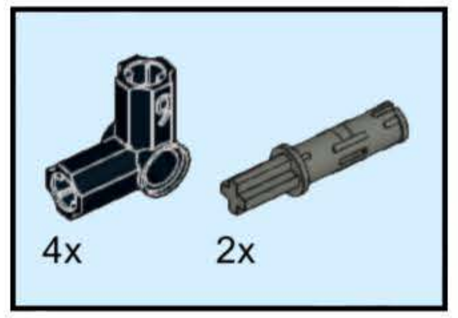


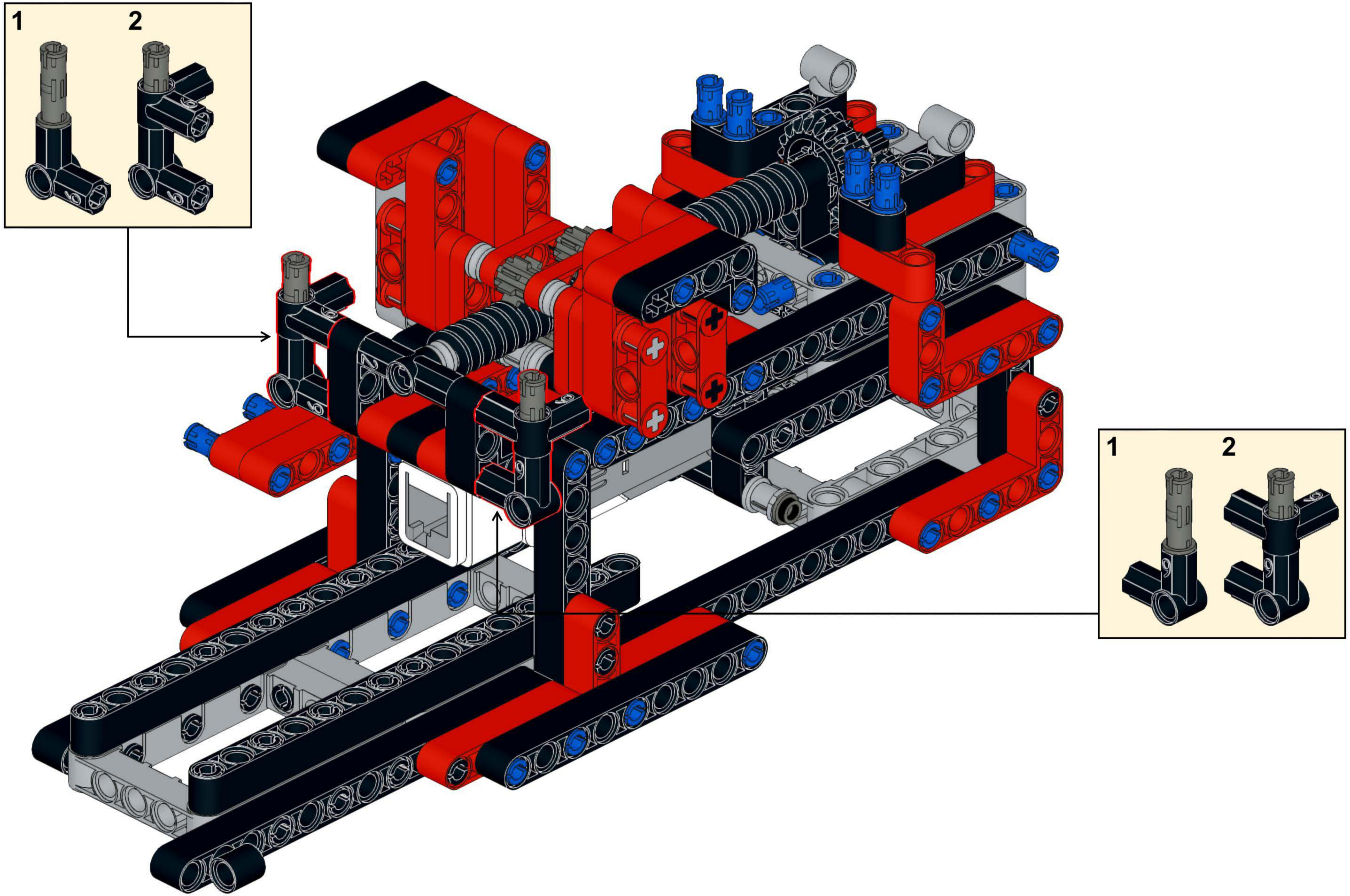

99

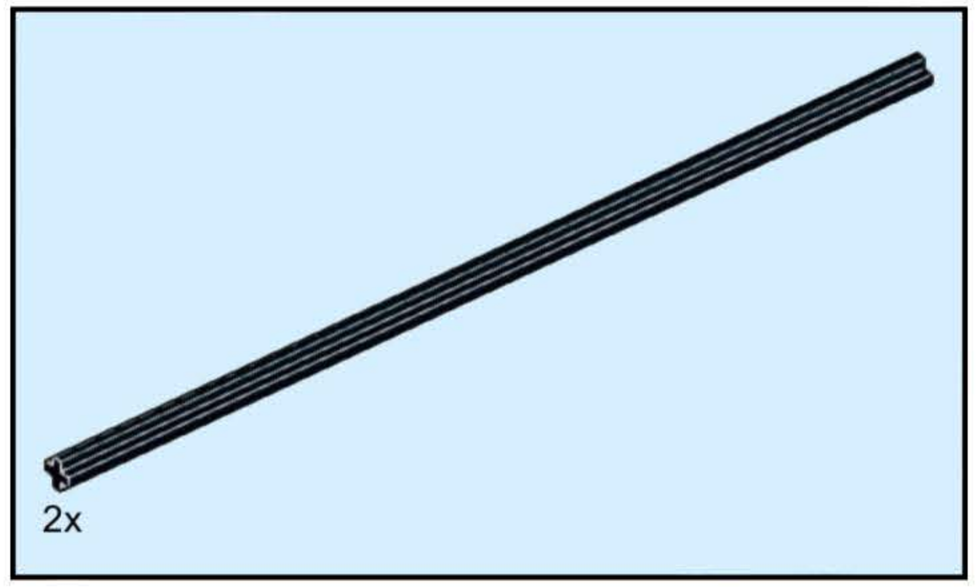


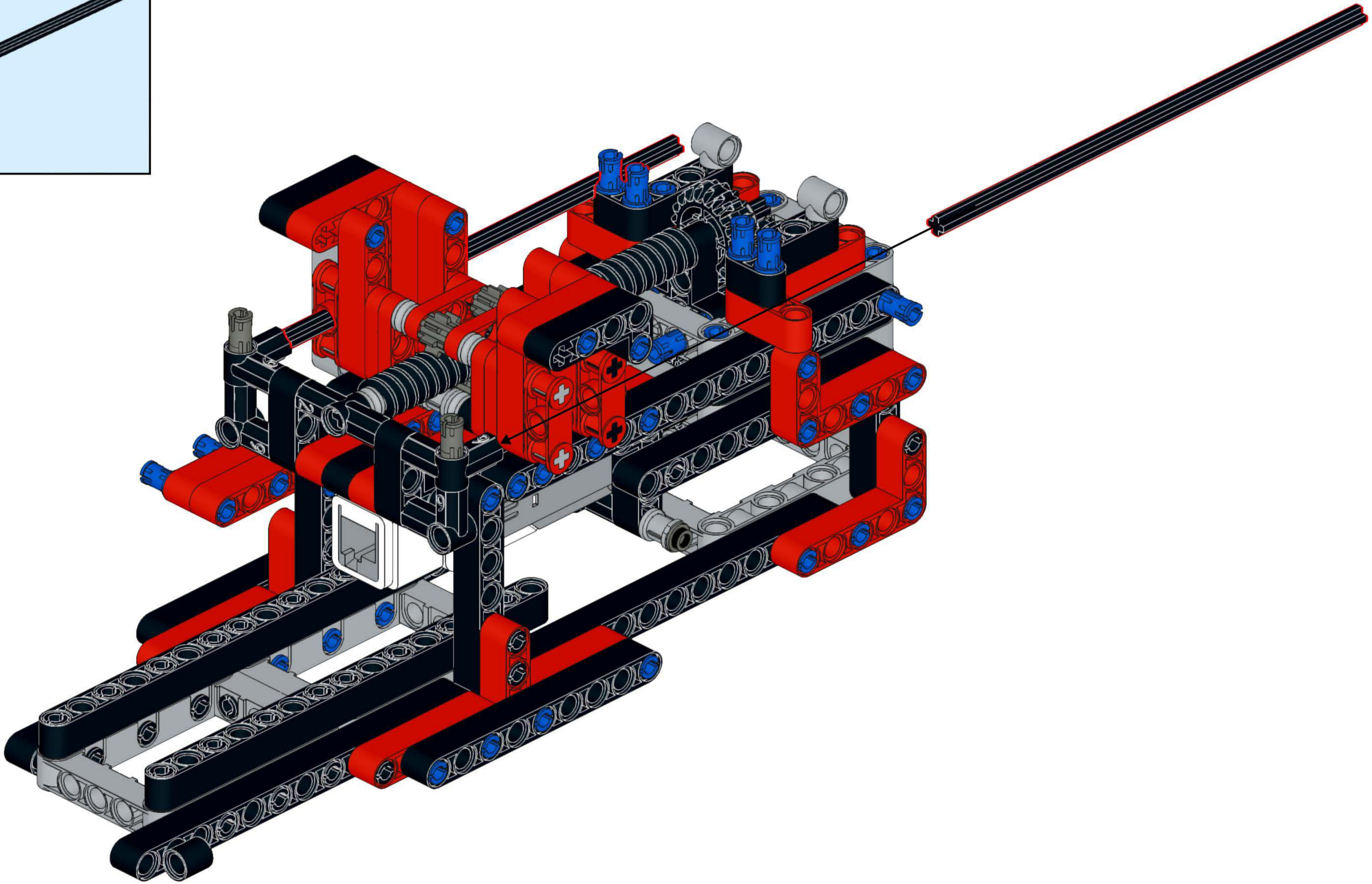

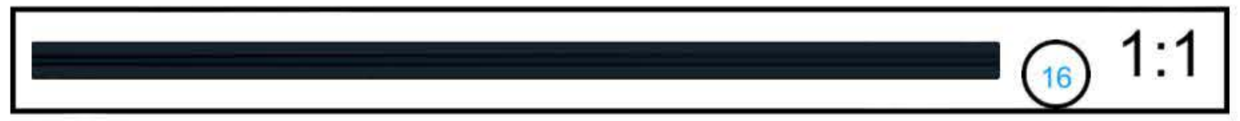

100

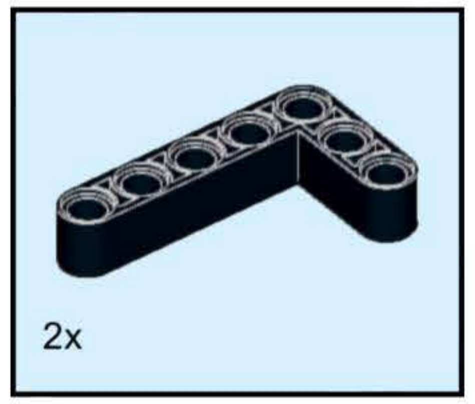


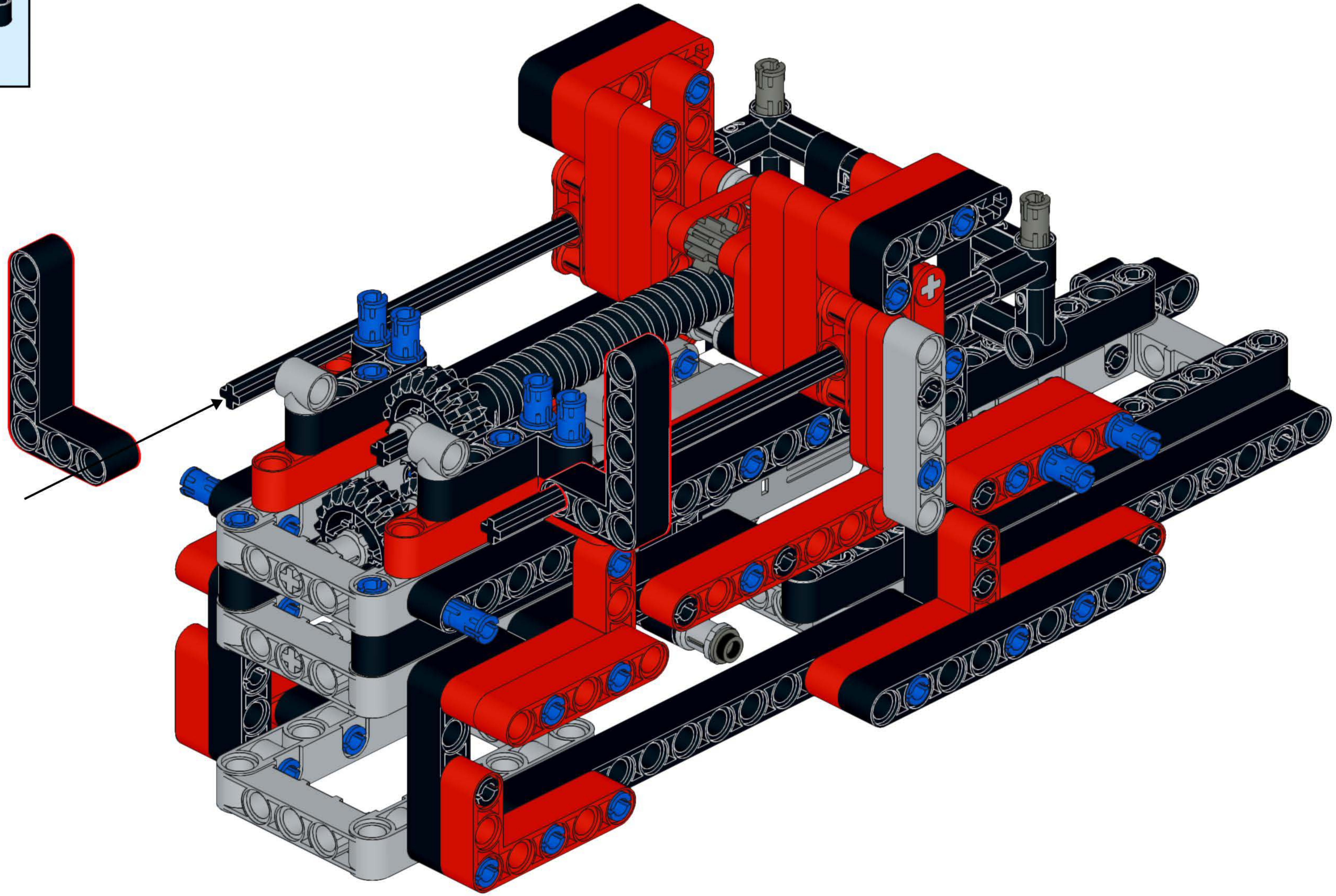

101

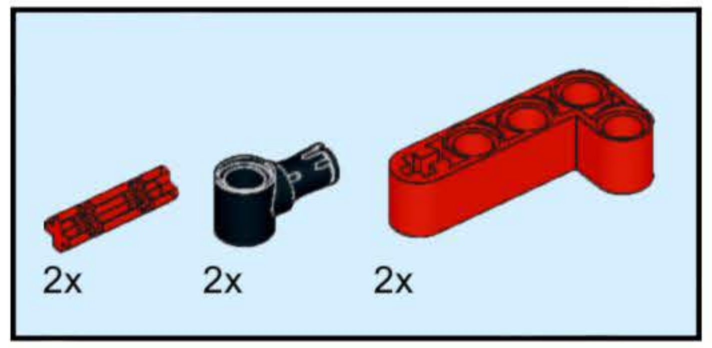


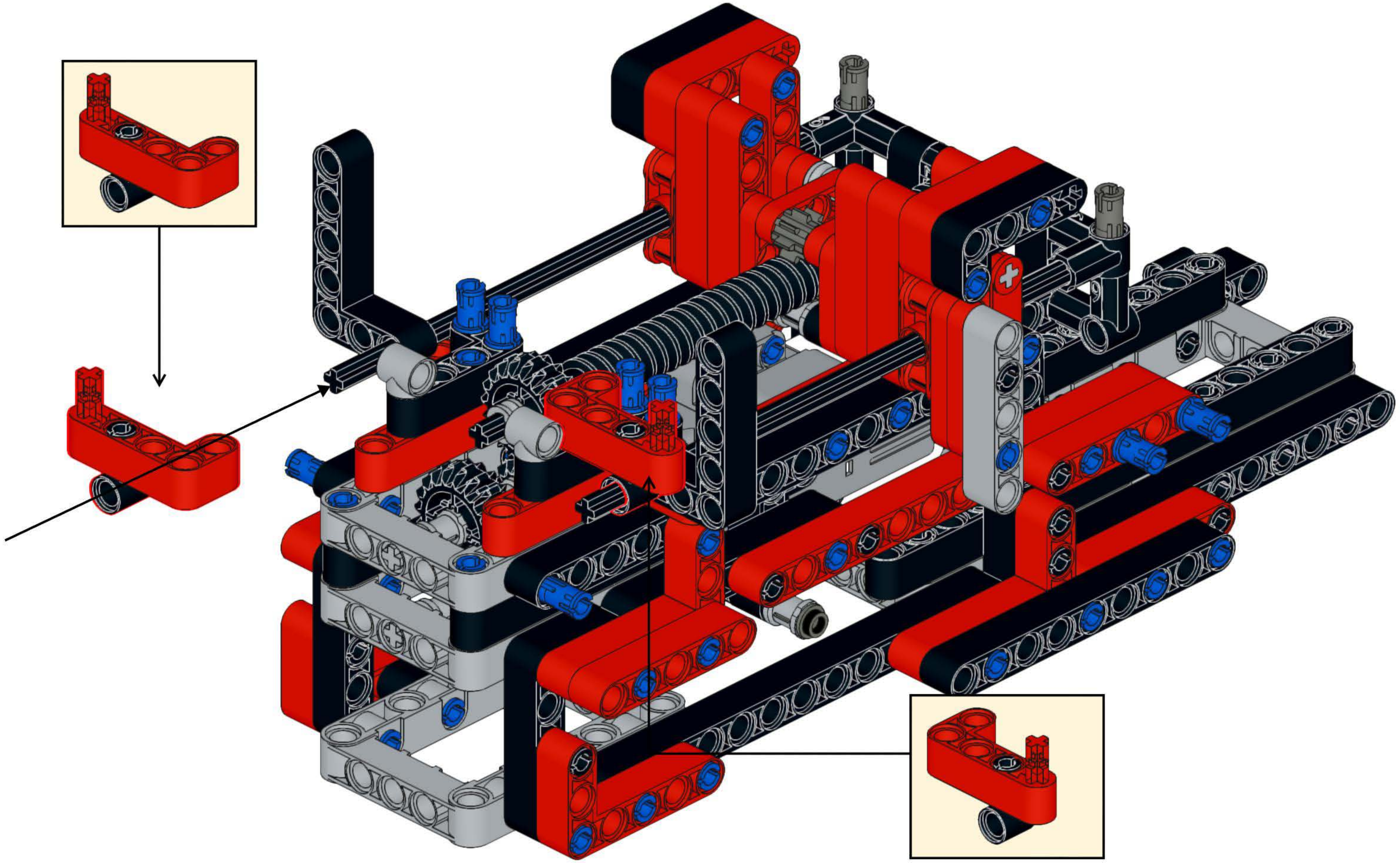

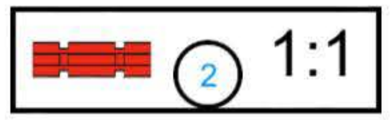

**102**

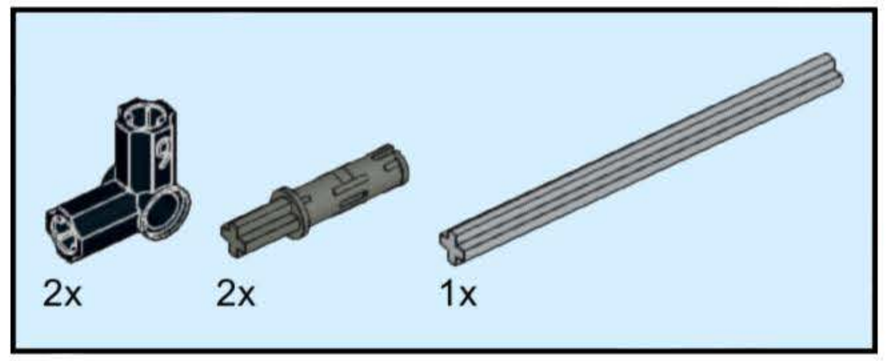


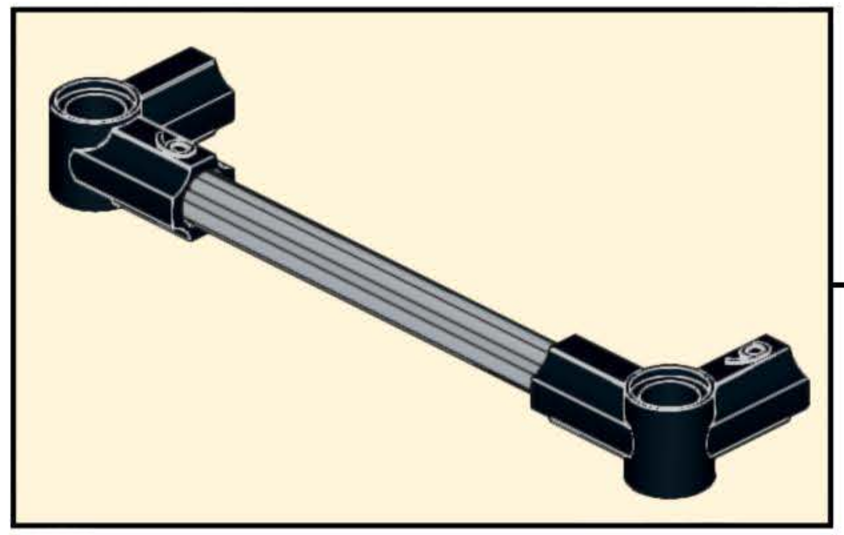

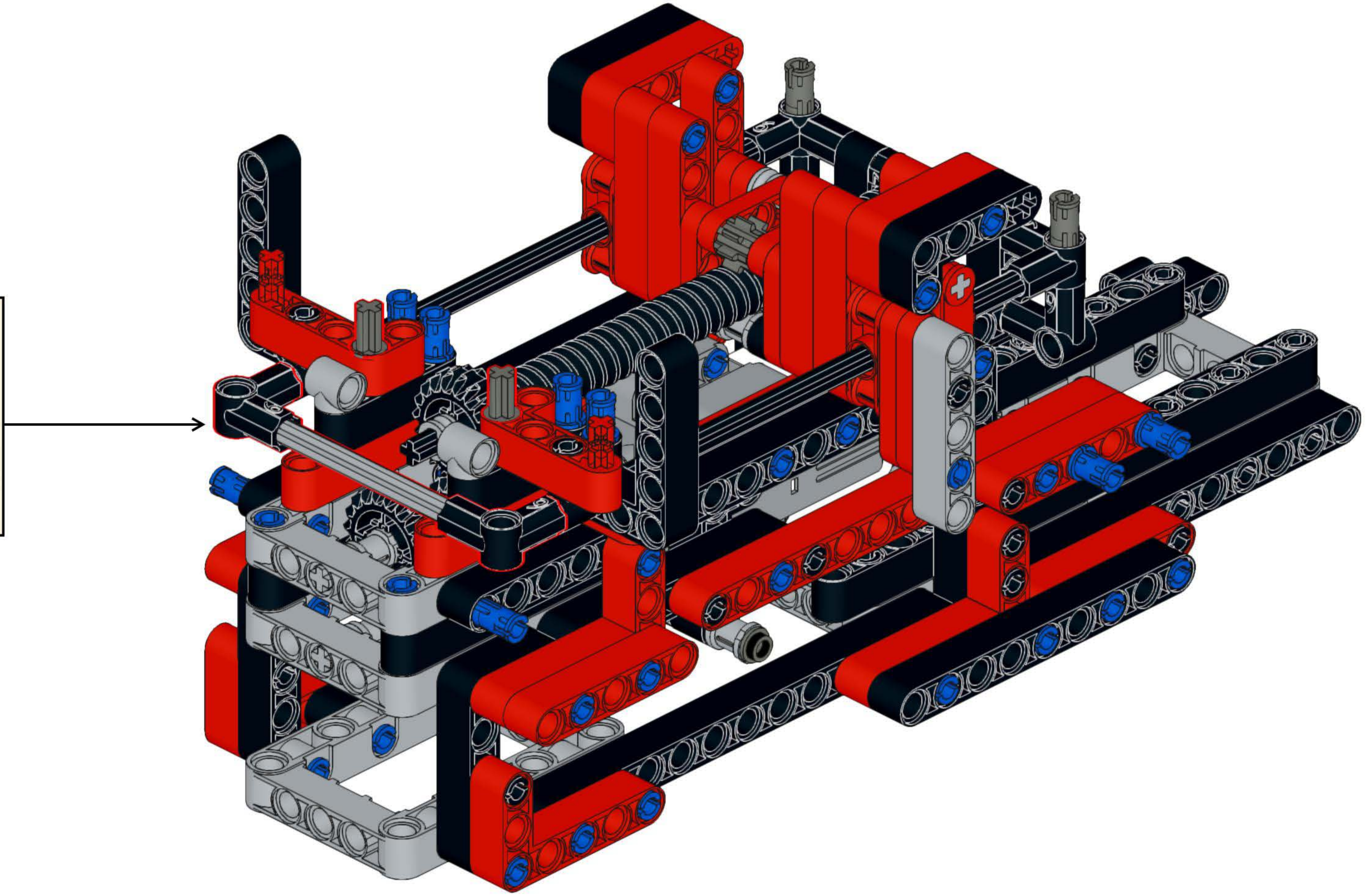

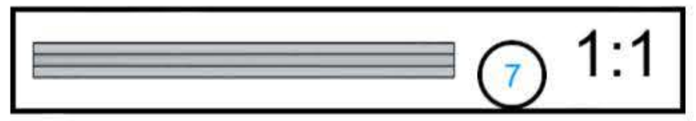

103

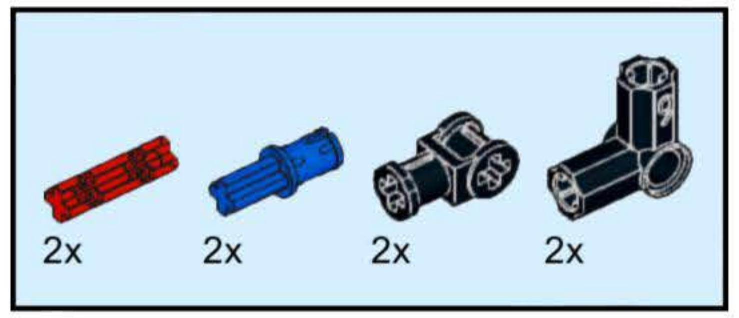


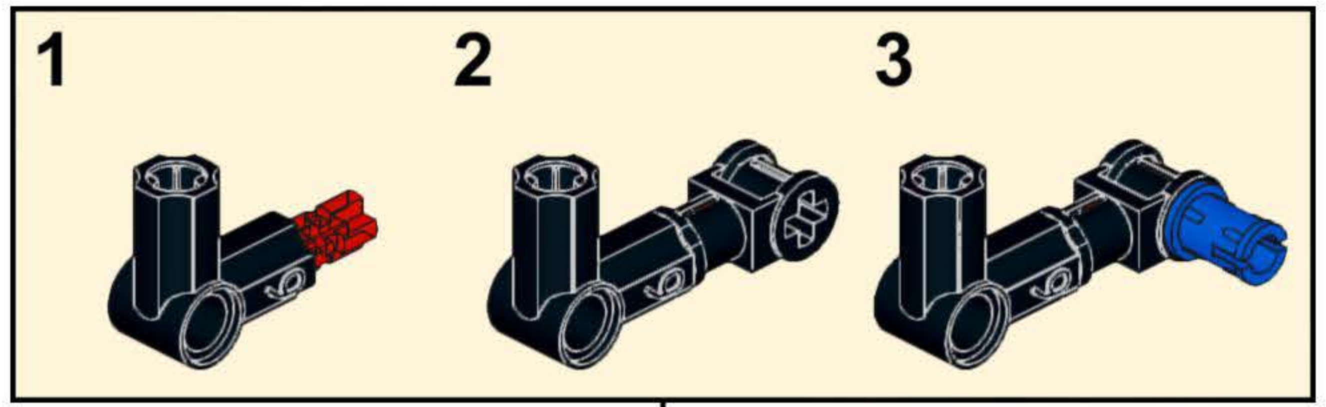


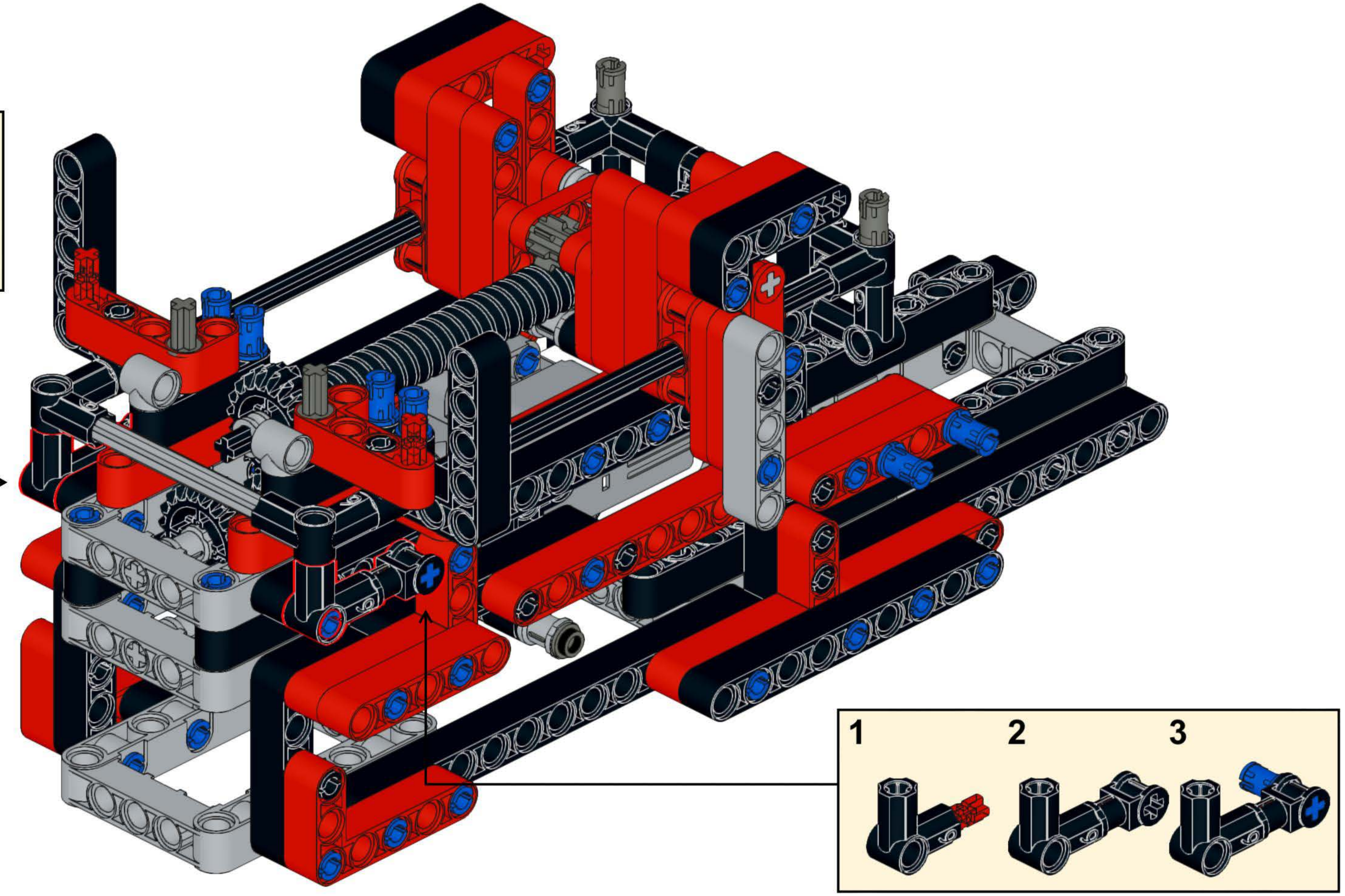

104

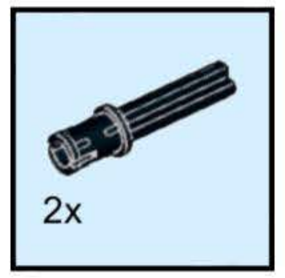


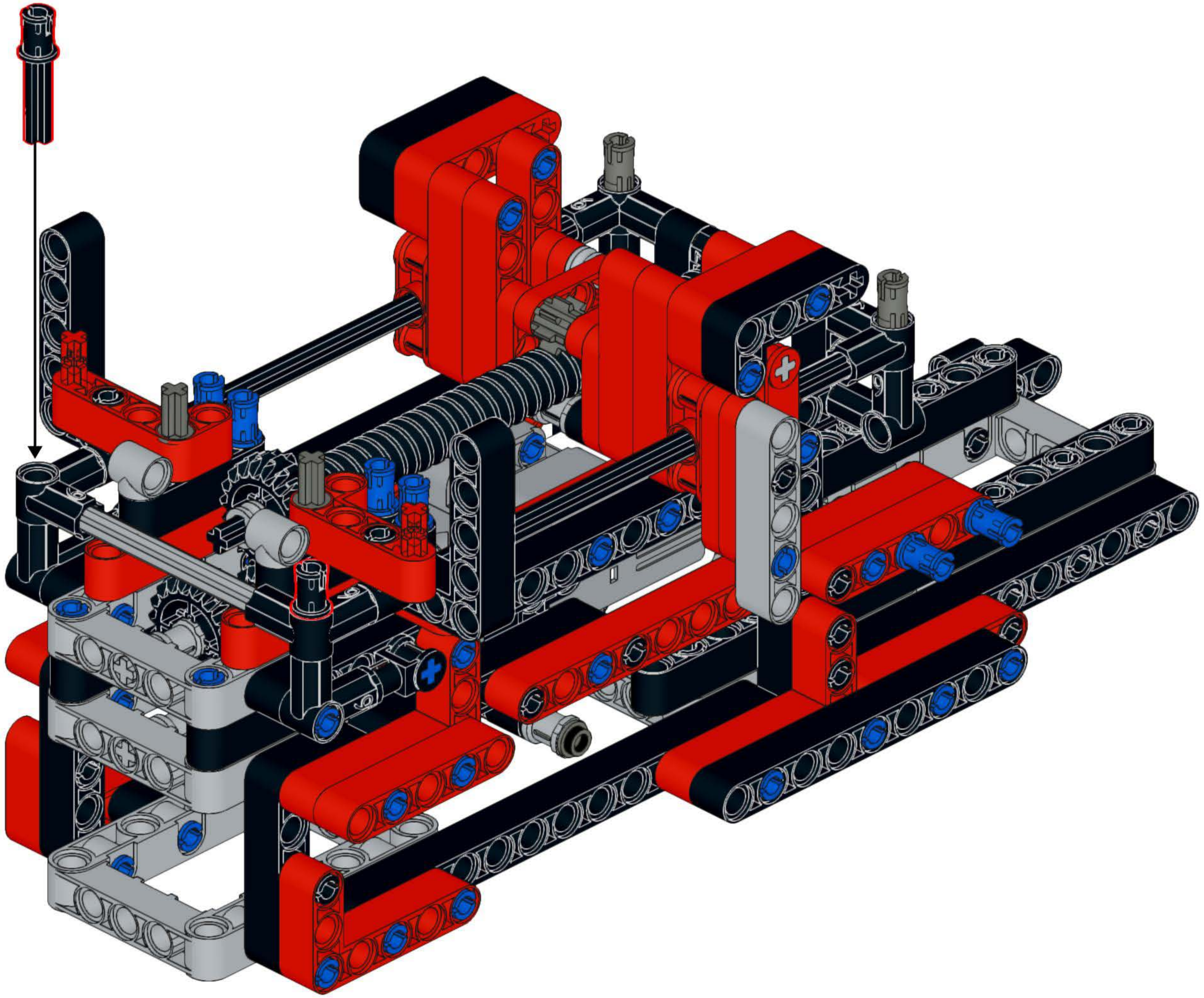

105

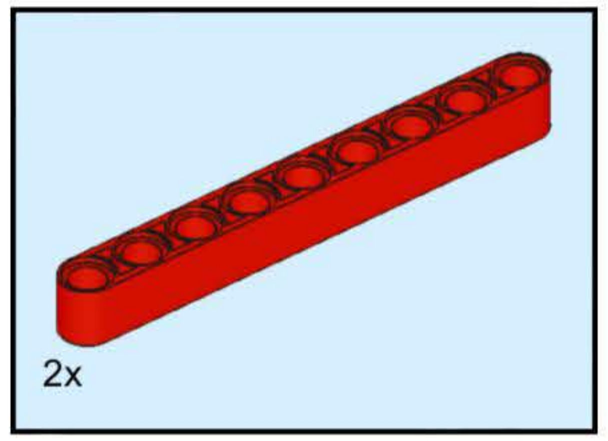


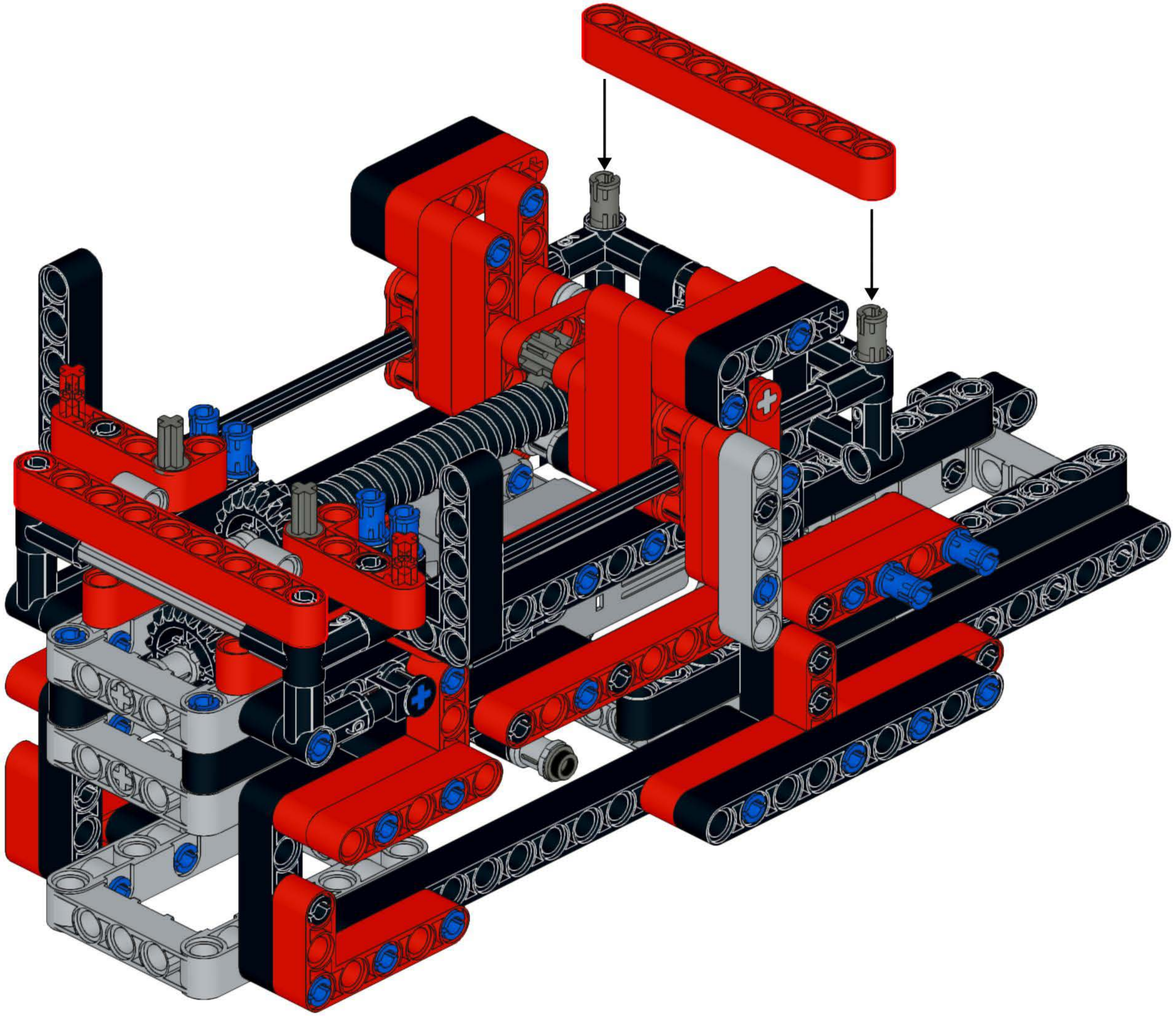

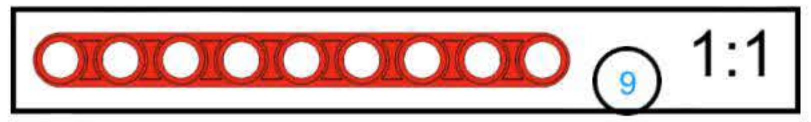

# 106

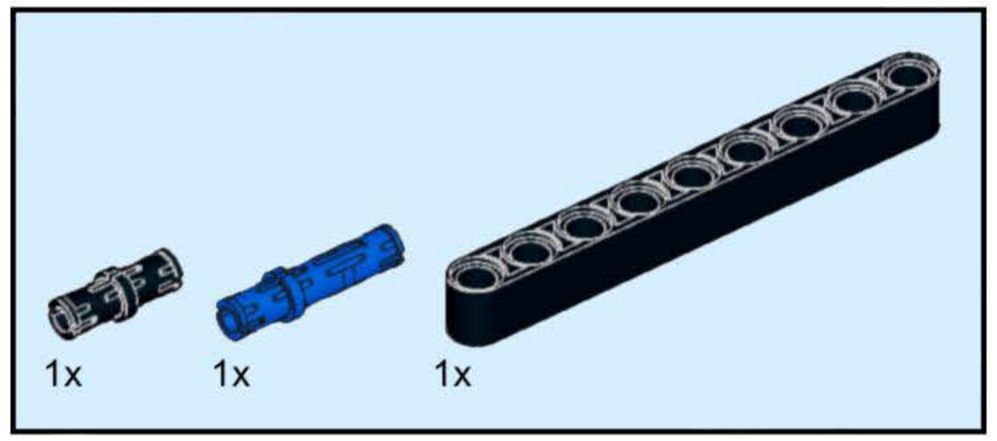


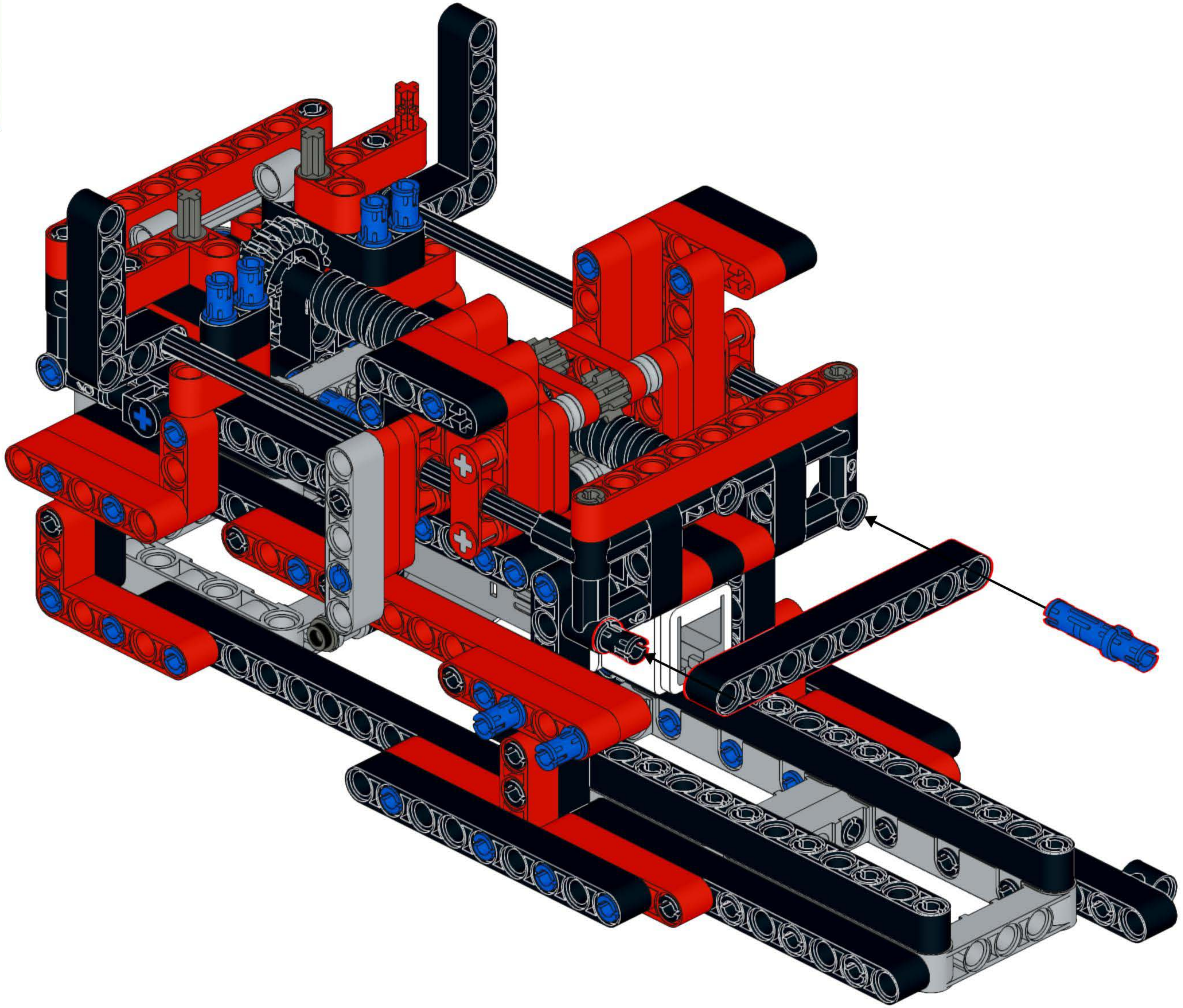

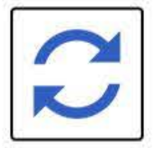

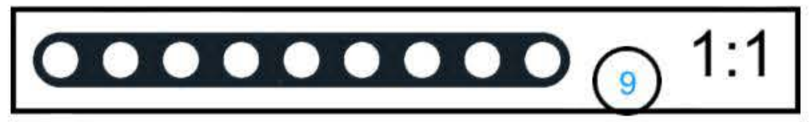

107

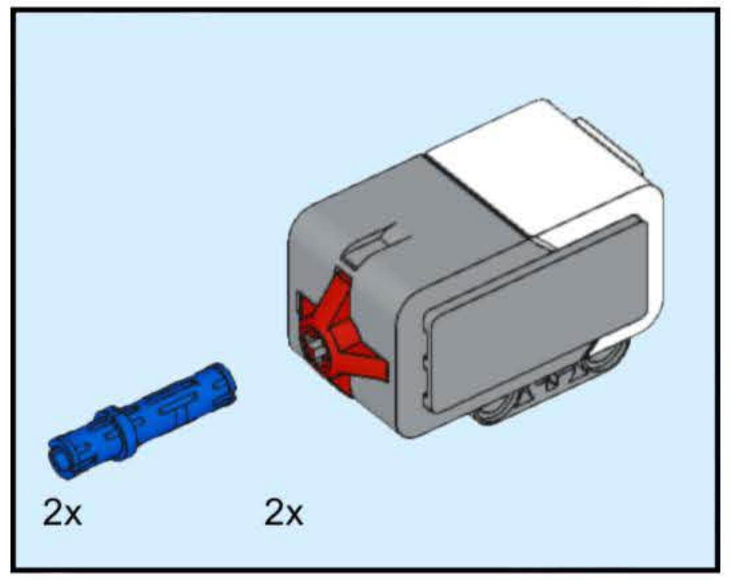


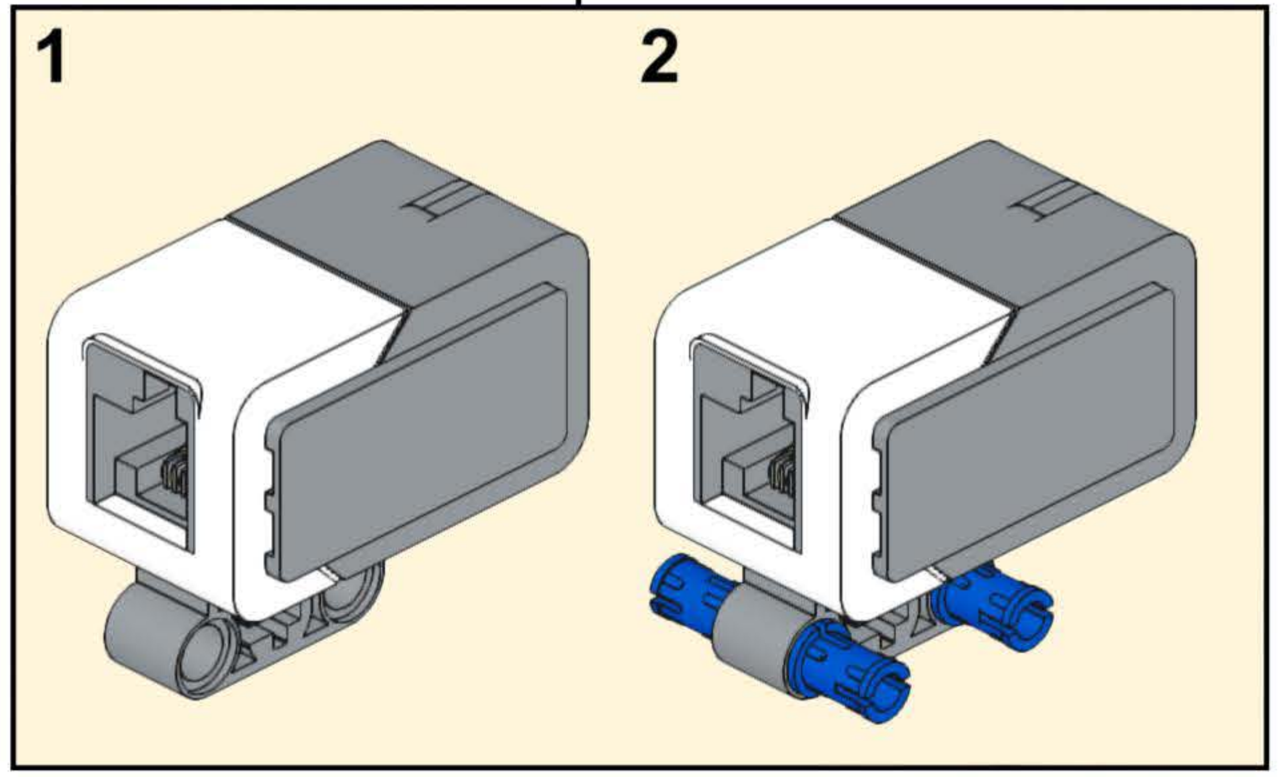


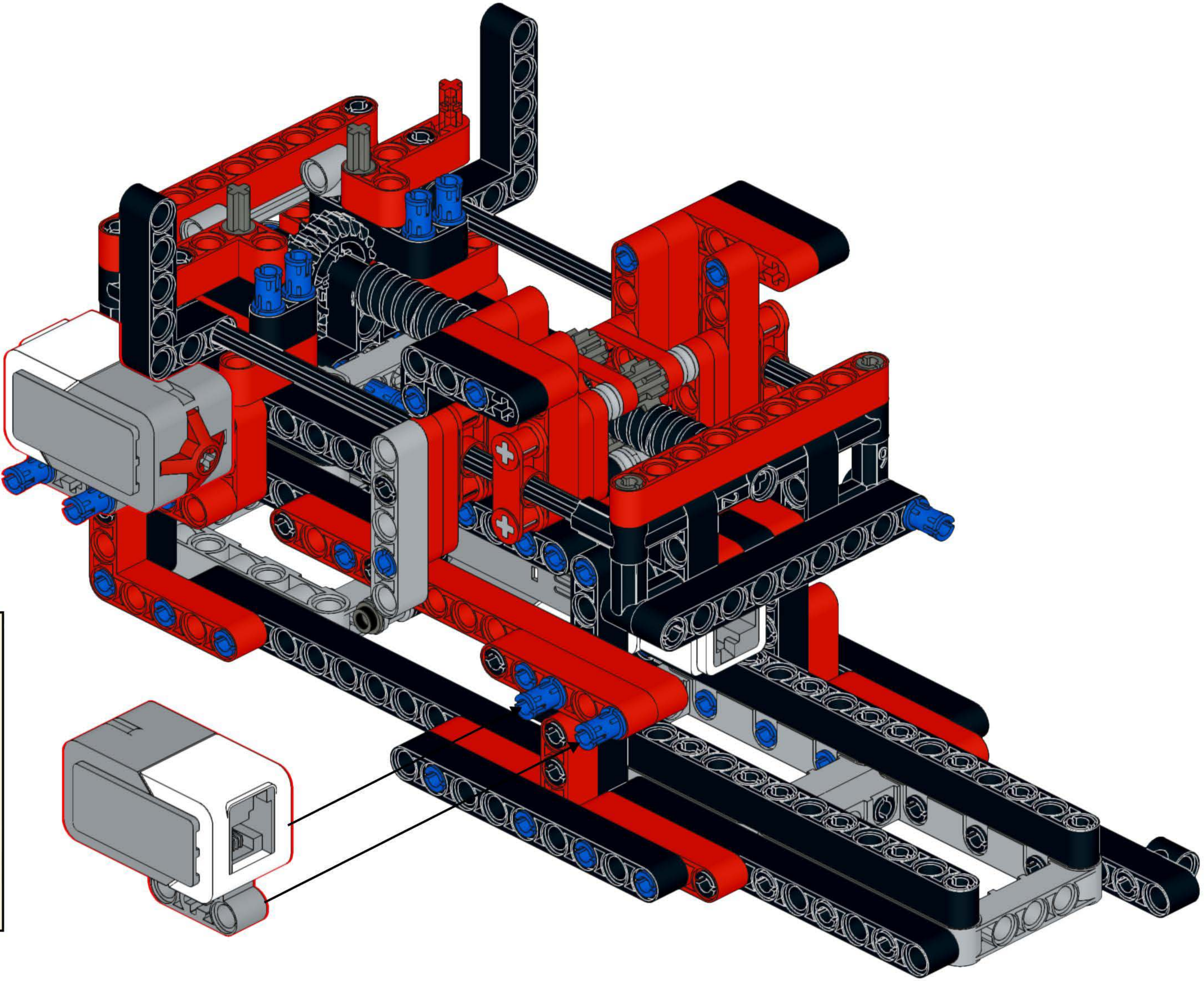

108

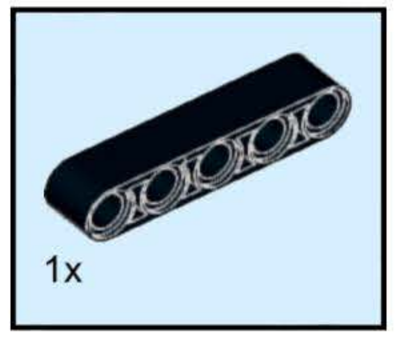


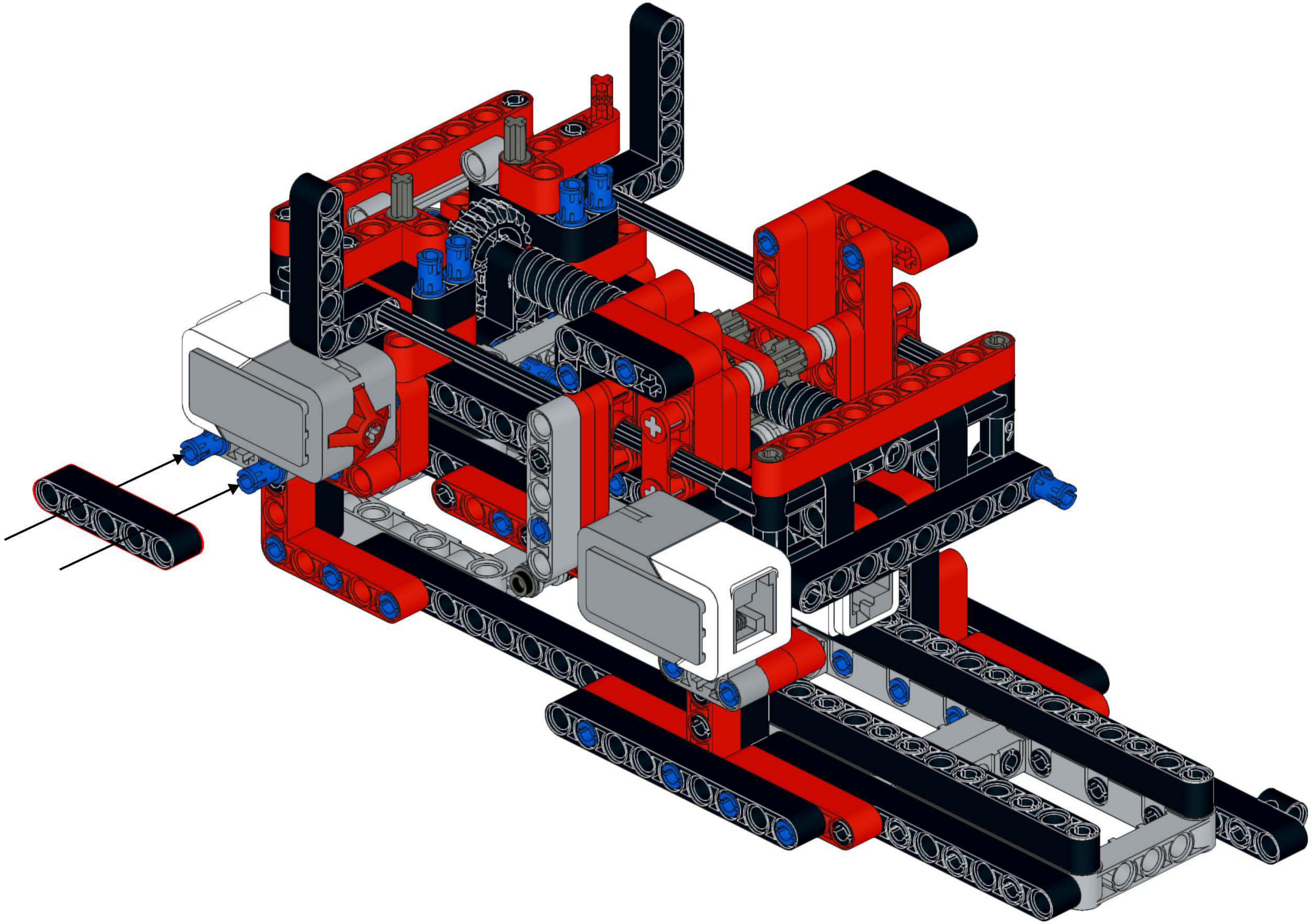

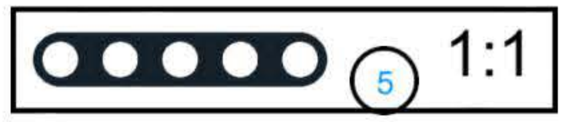

# 109

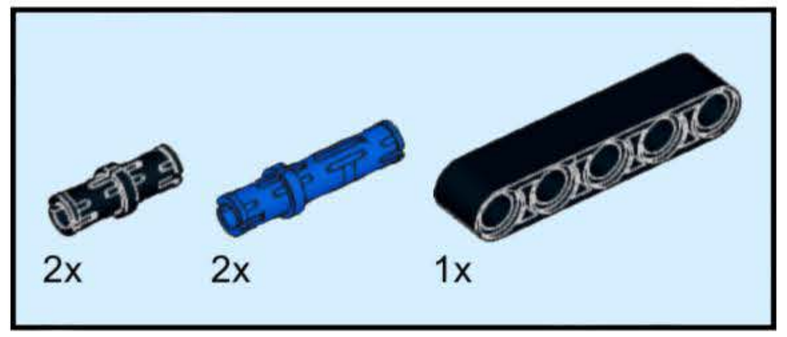


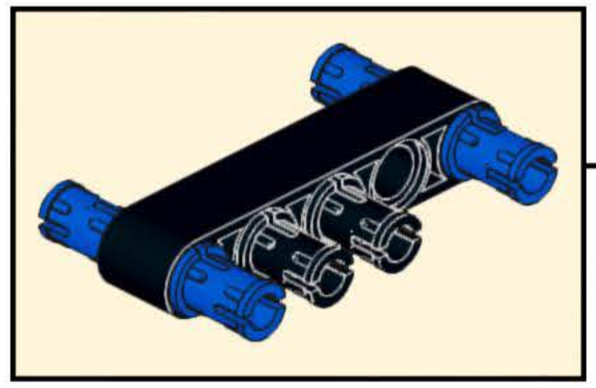

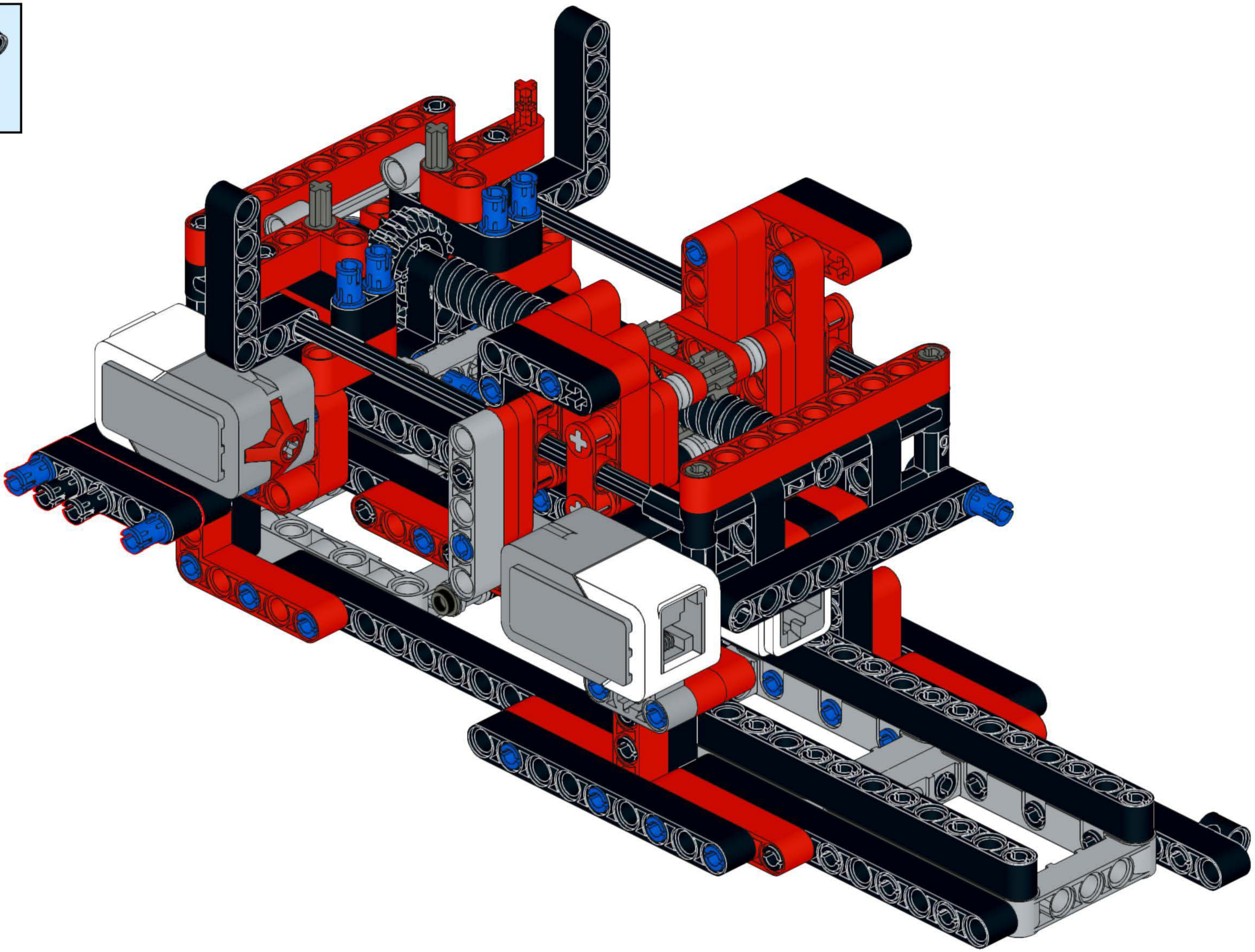

# 110

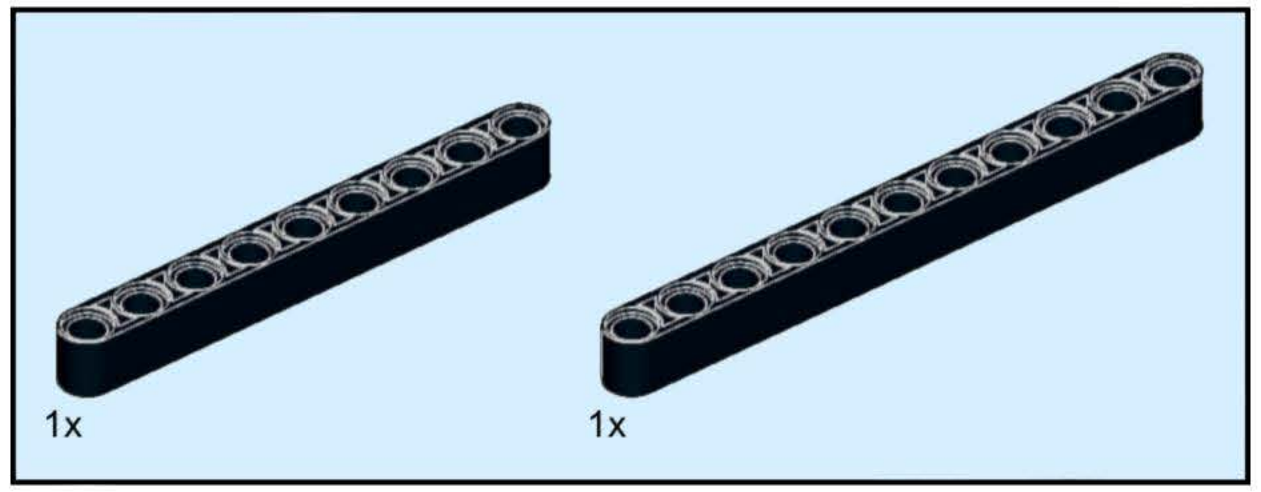


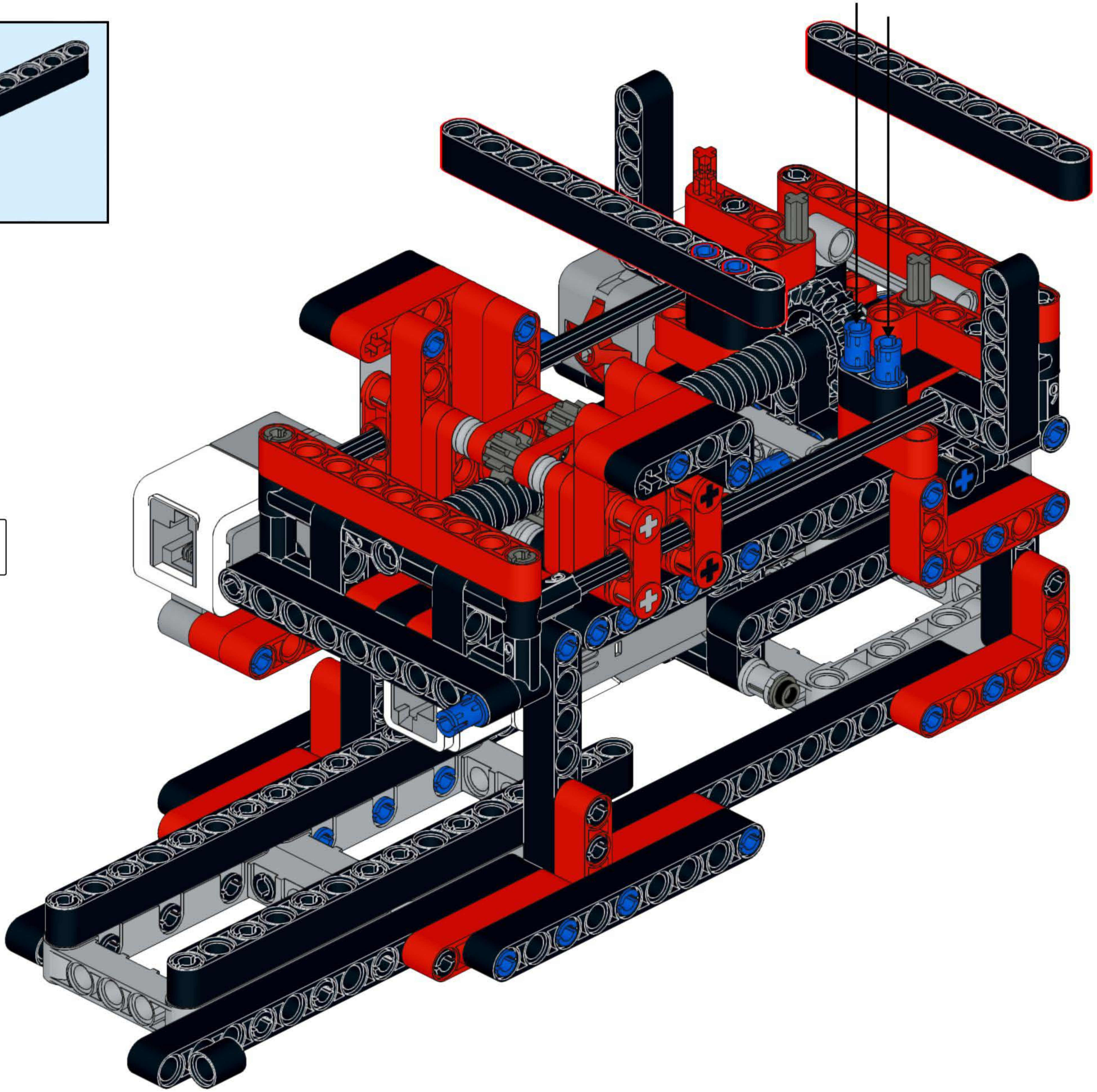

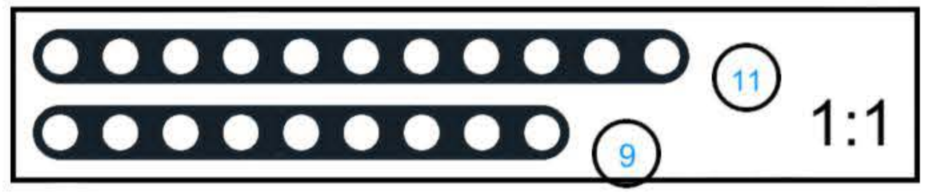

111

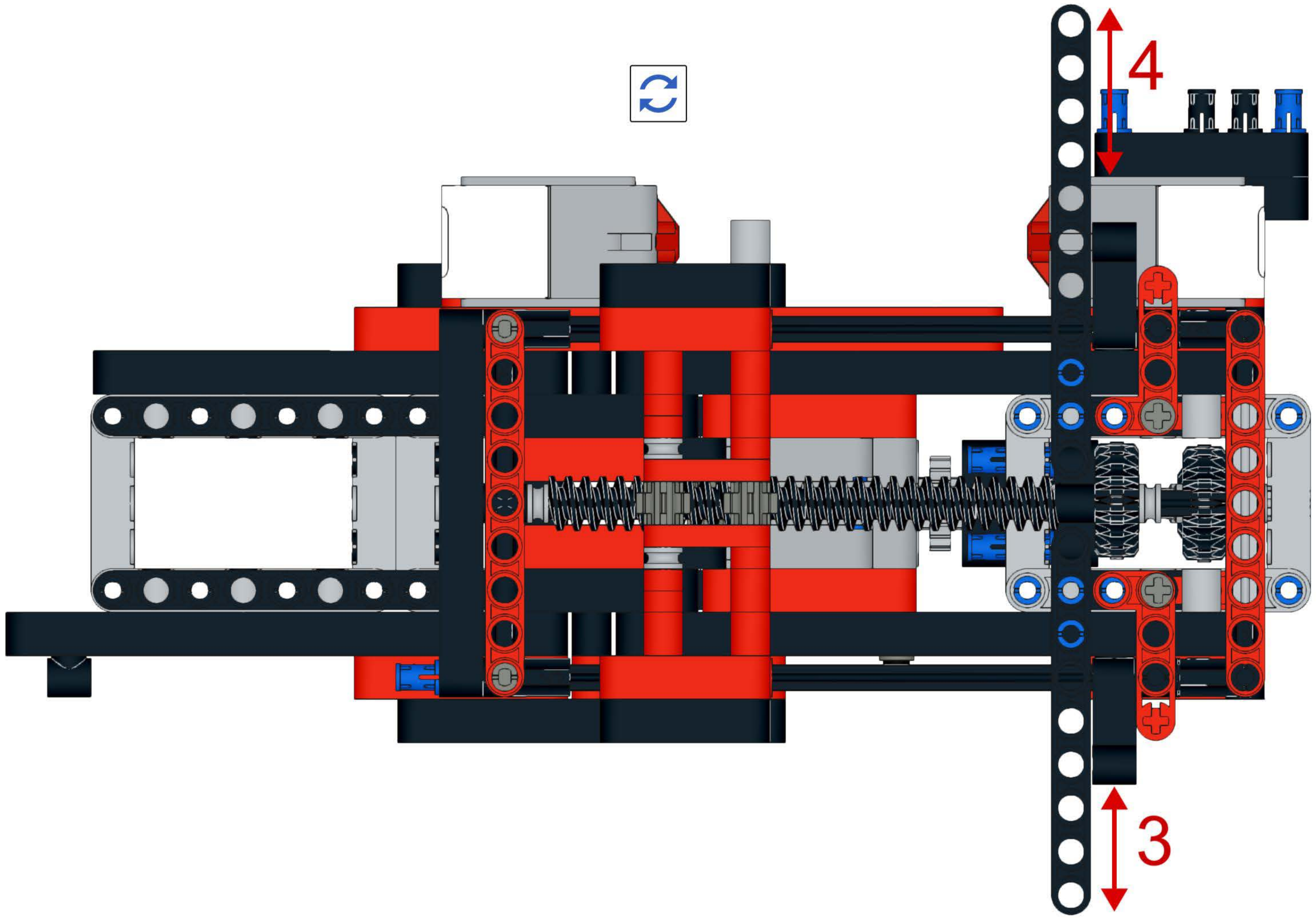

# 112

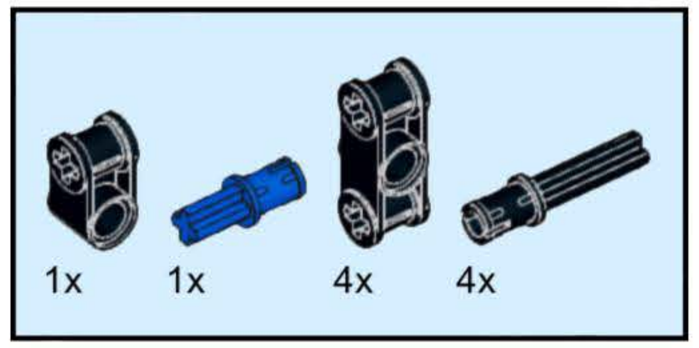


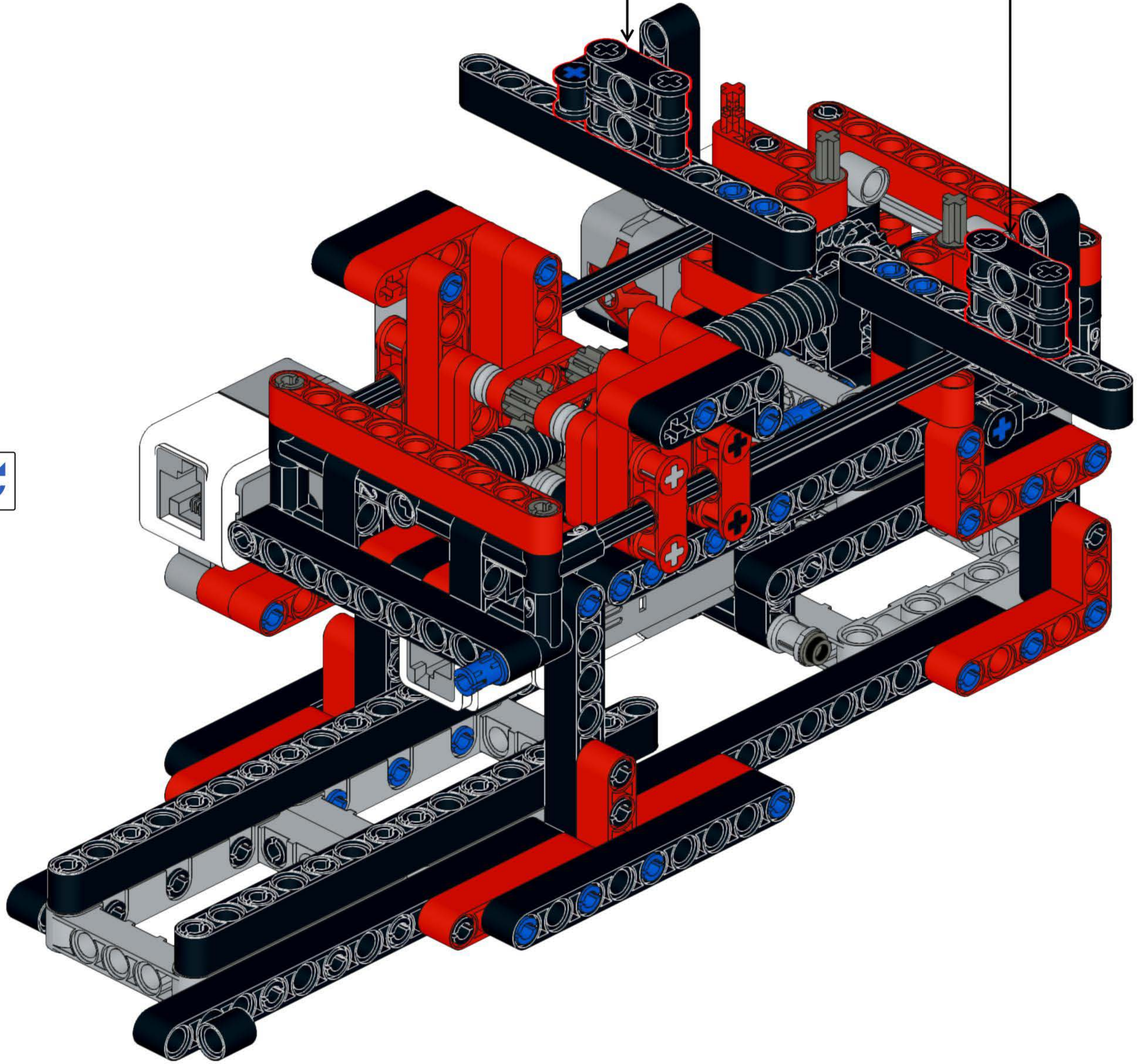

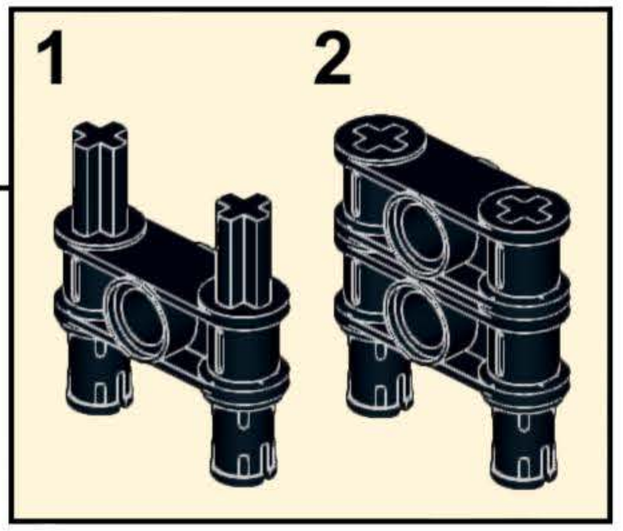


2x

# 113

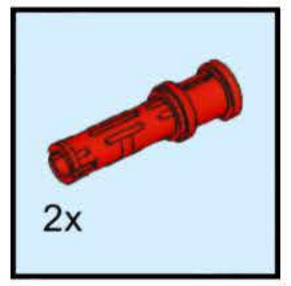


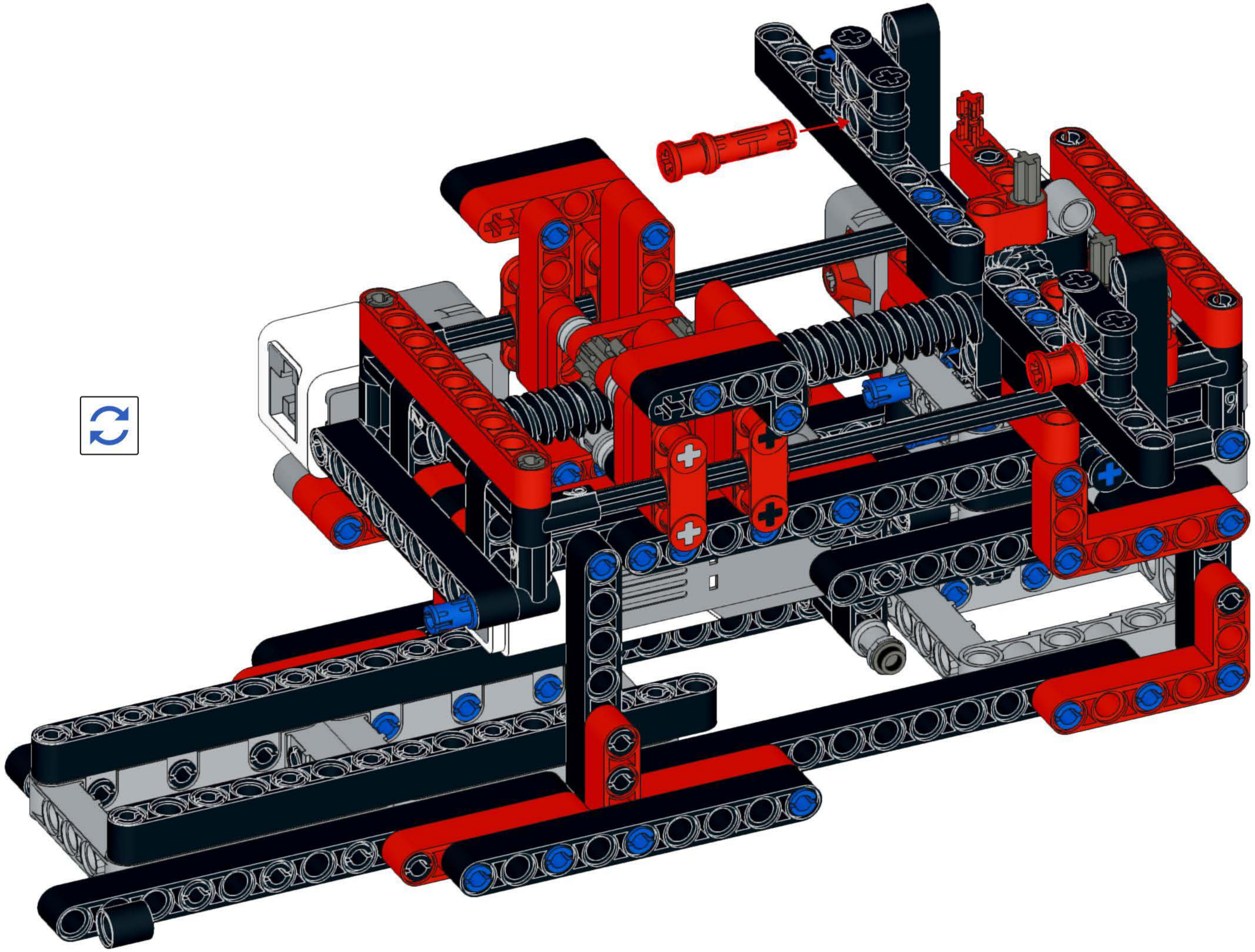

# 114

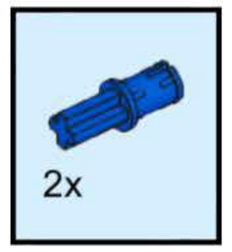


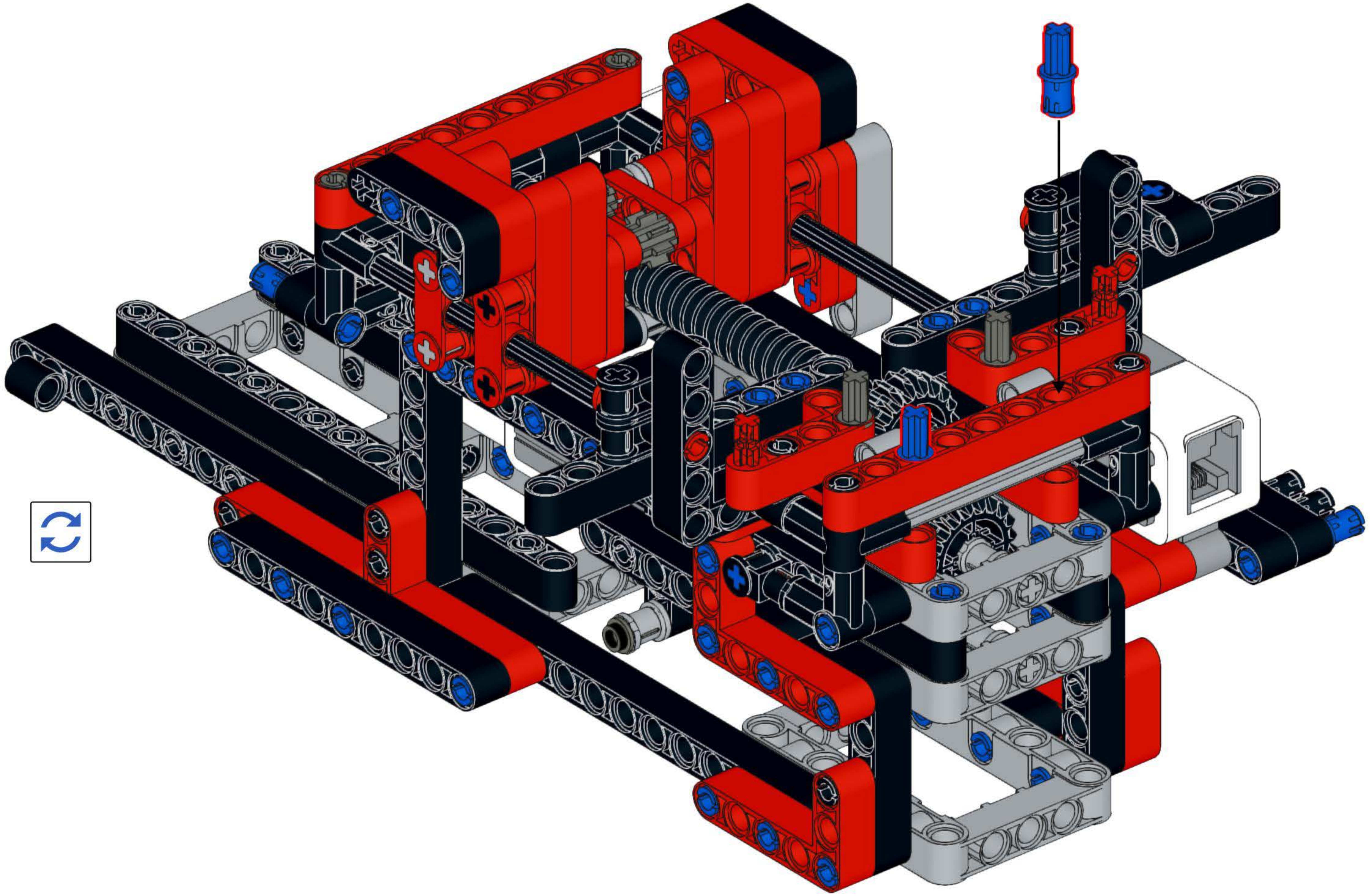

# 115

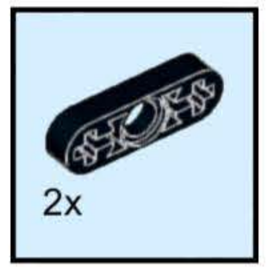


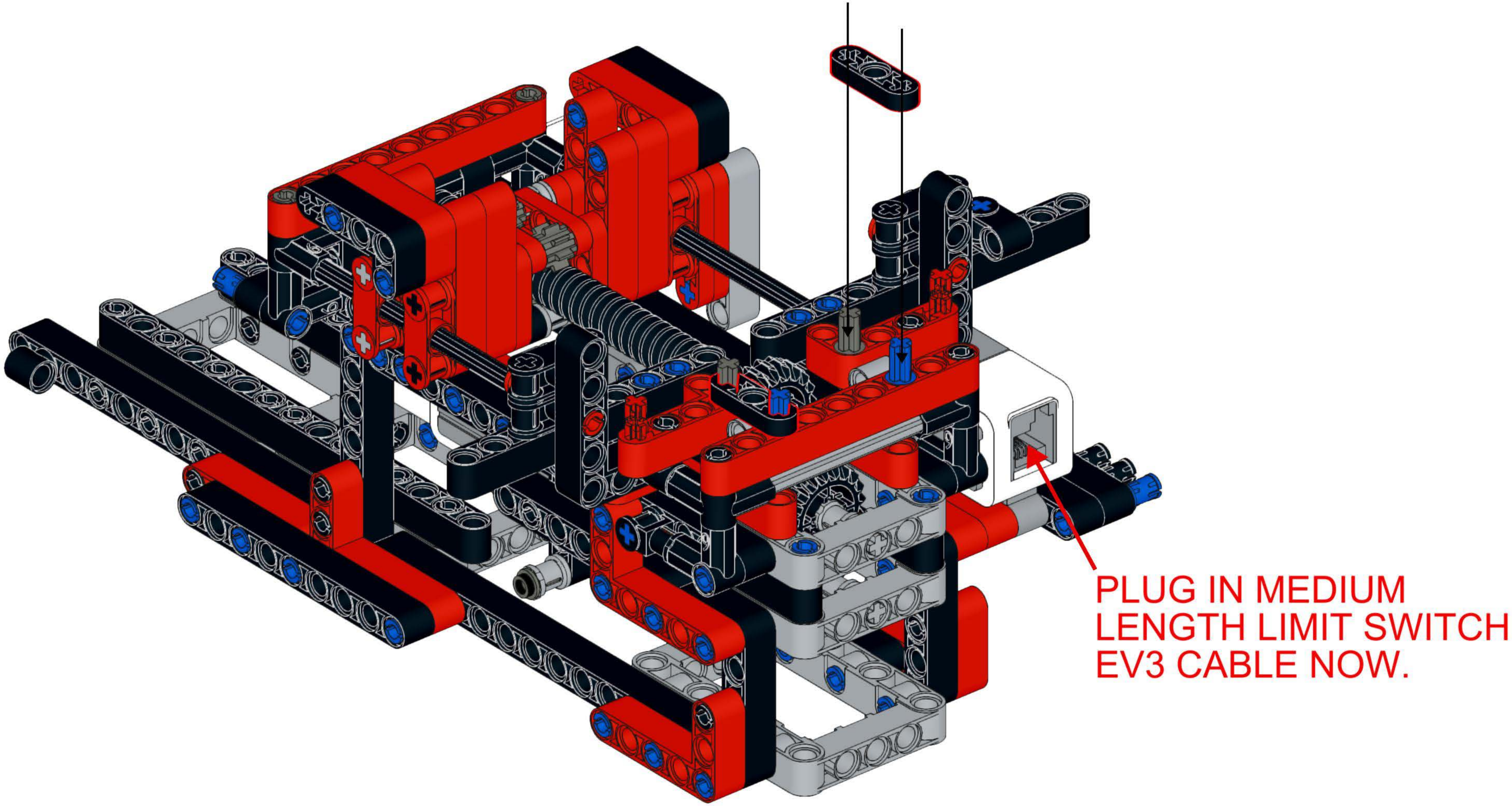


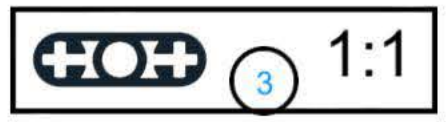

# 116

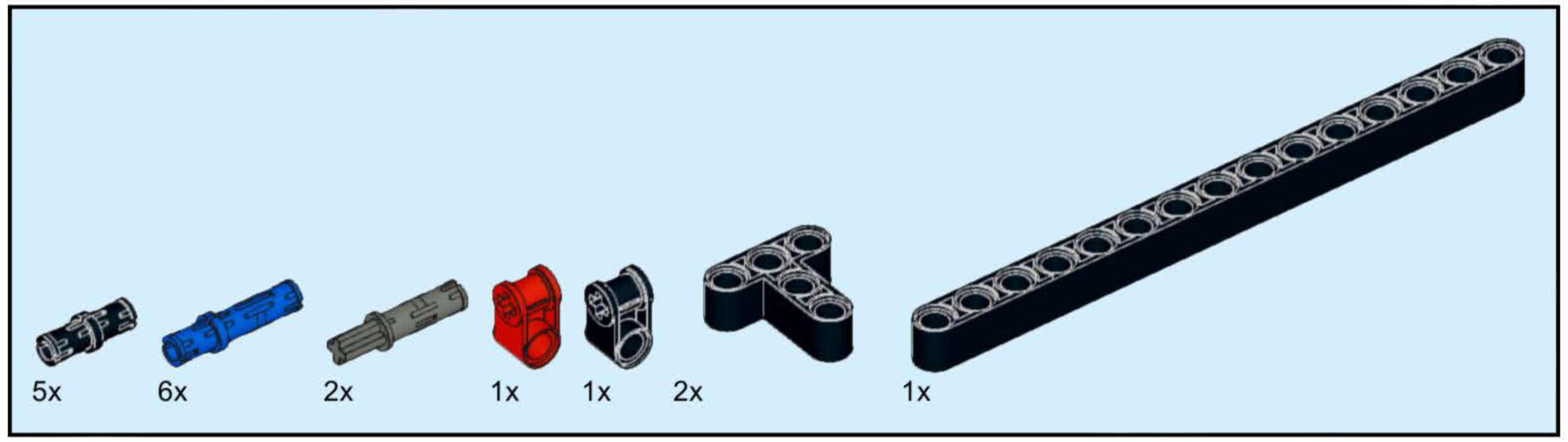


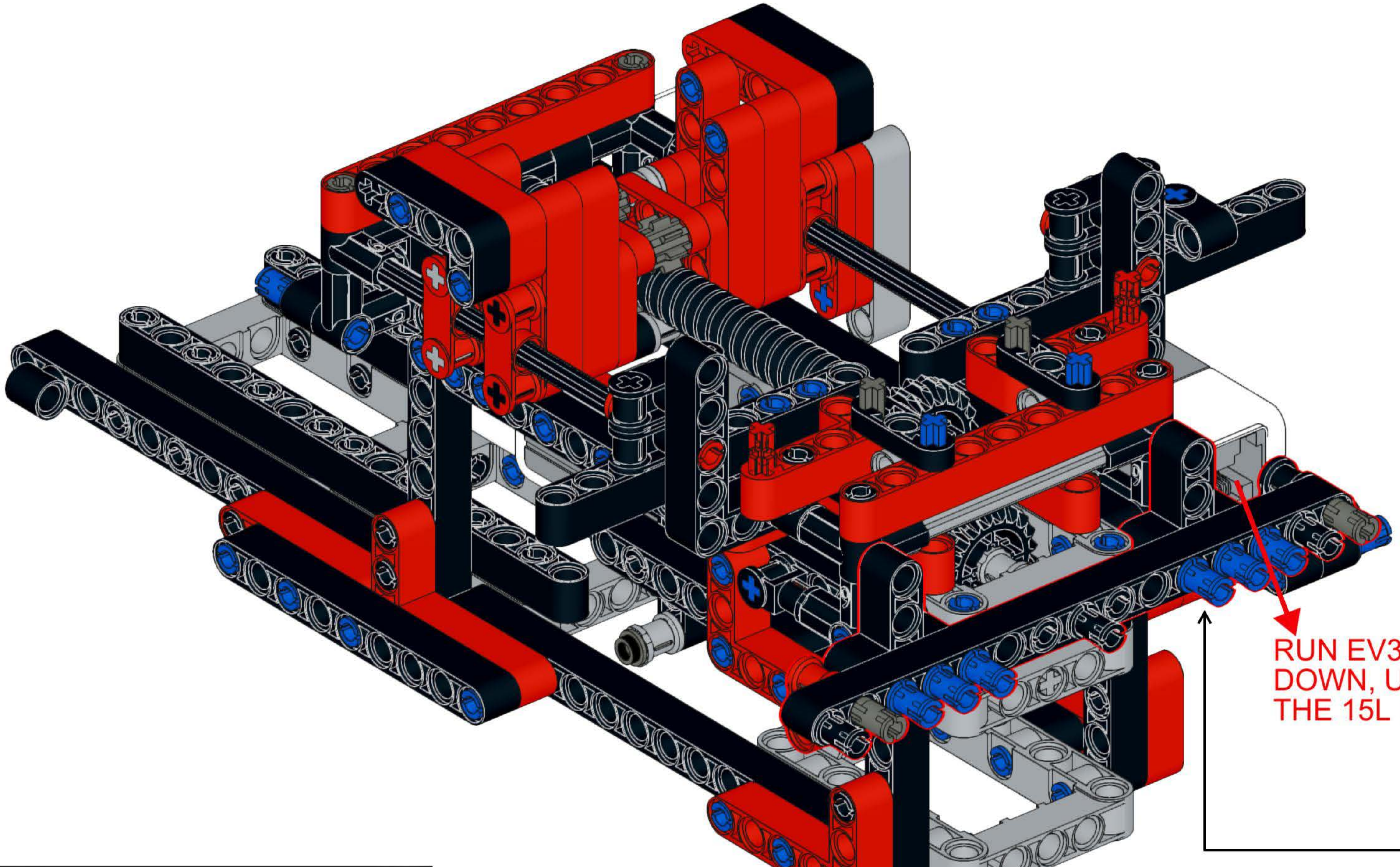

RUN EV3 CABLE DOWN, UNDER THE 15L LIFTARM.

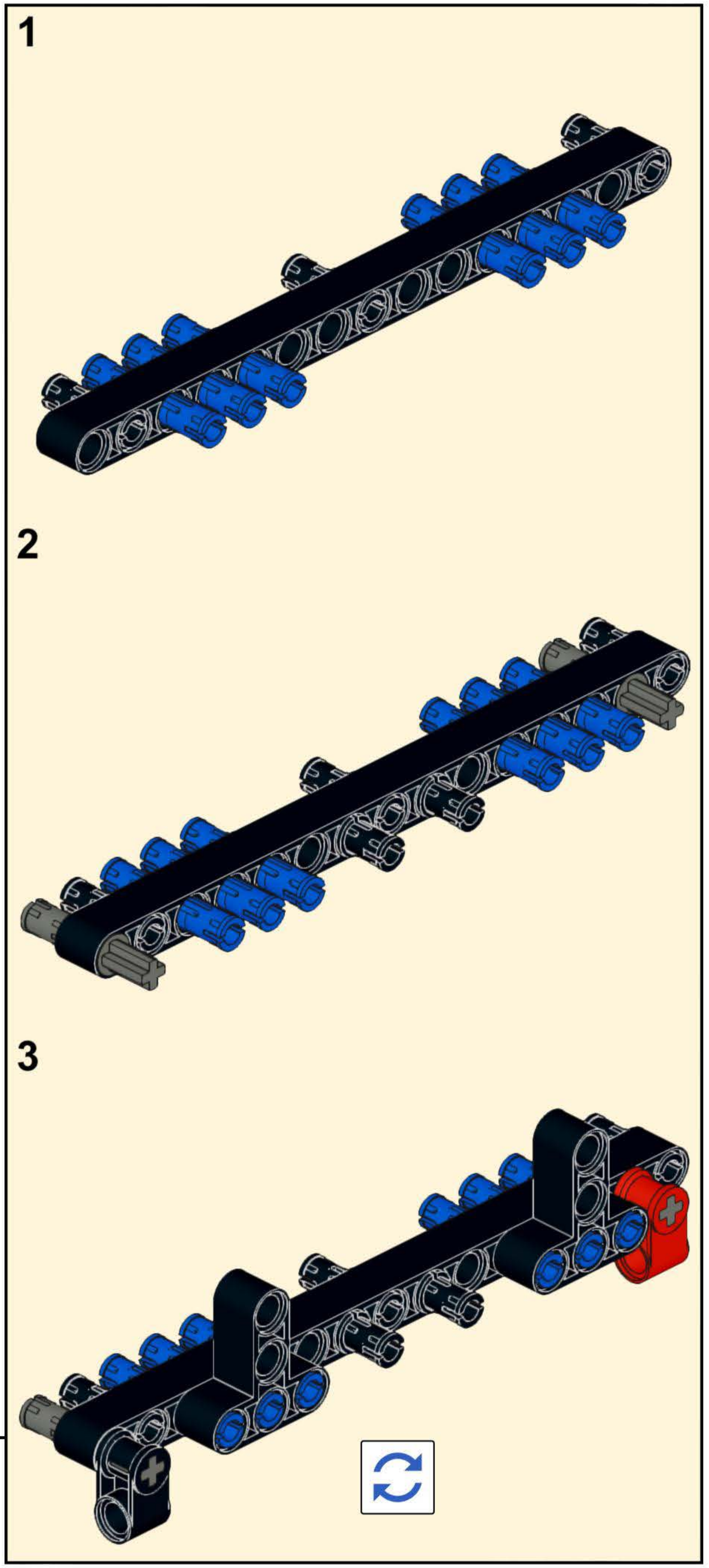


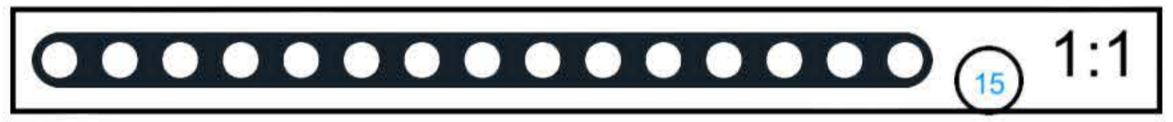

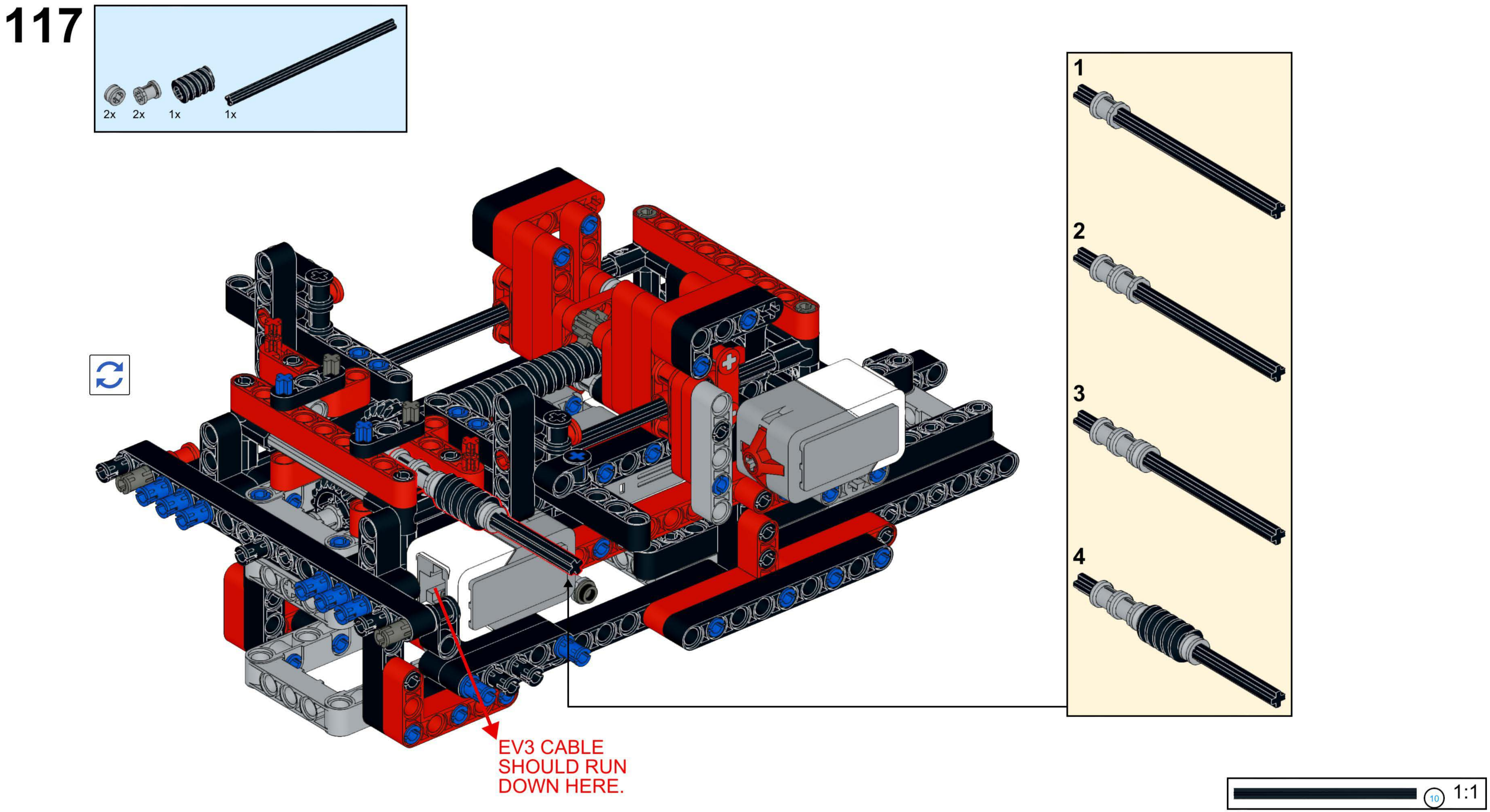
117
2x
2x
1x
1x
1
2
3
4
EV3 CABLE
SHOULD RUN
DOWN HERE.
10
1:1

118

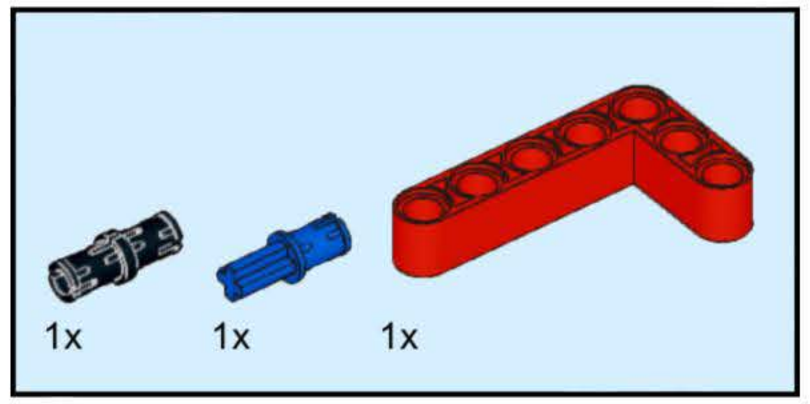


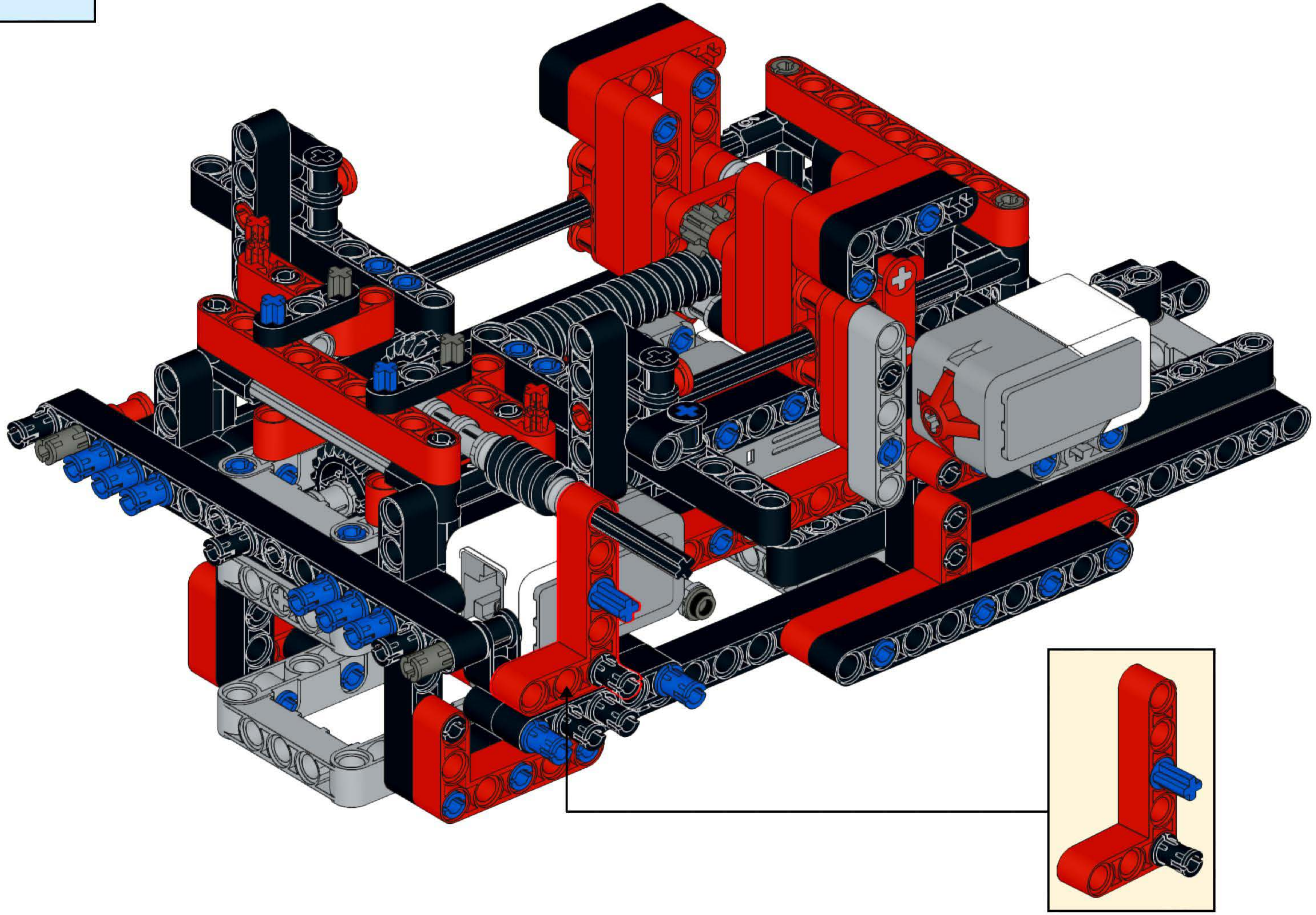

119

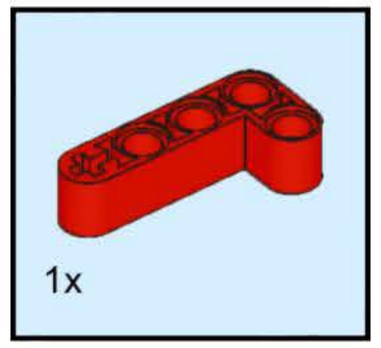


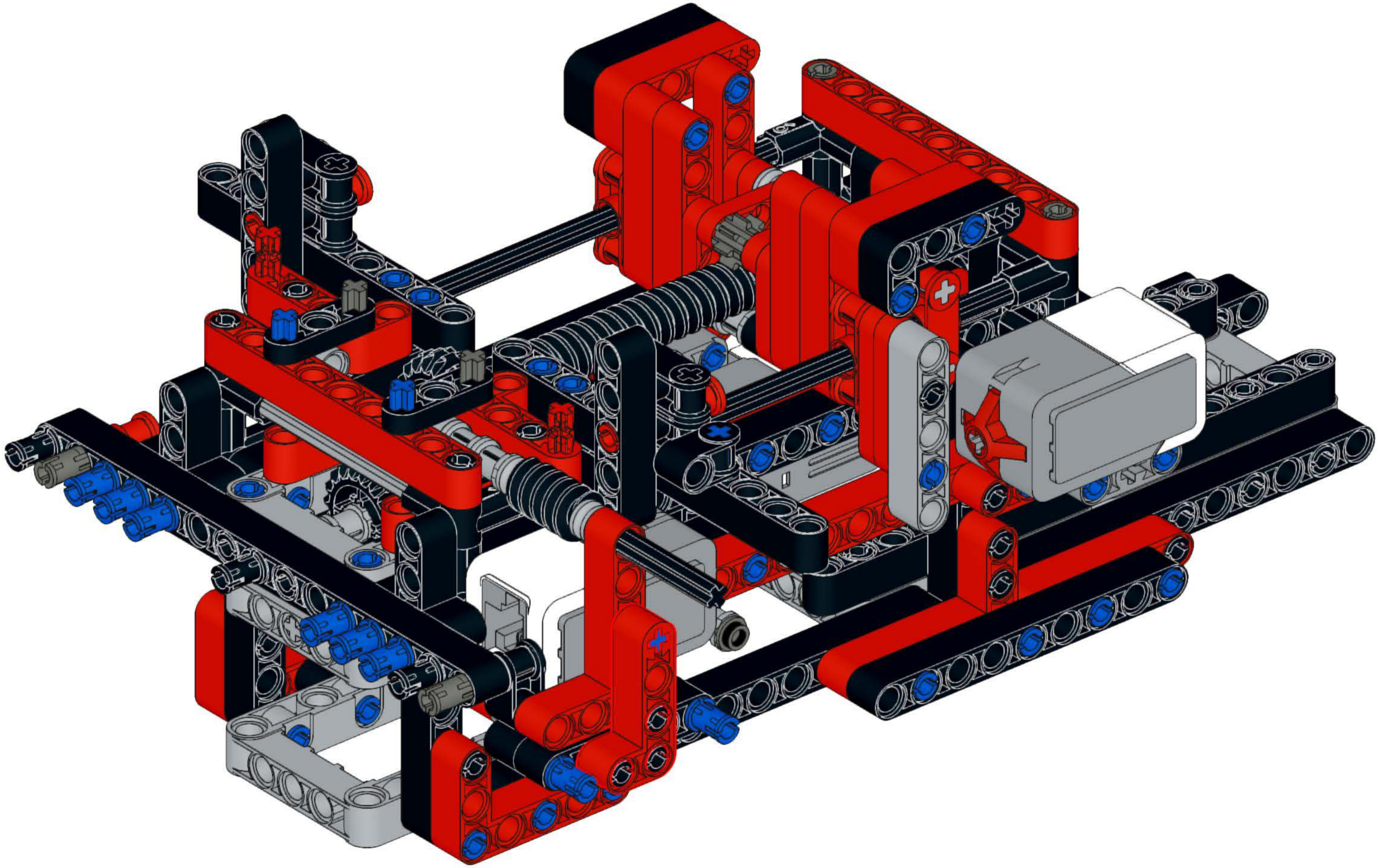

120

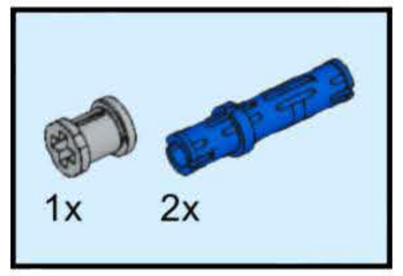


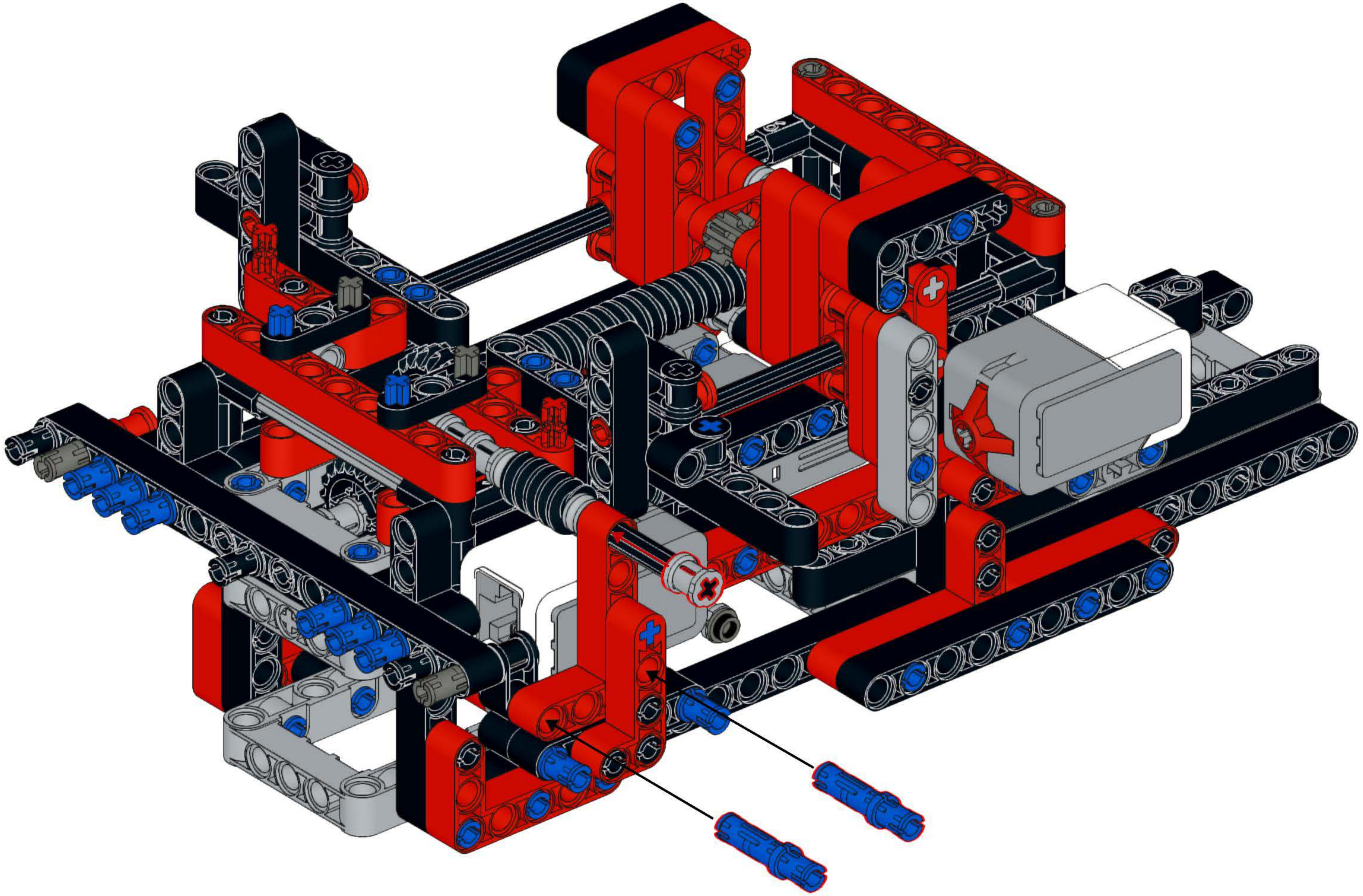

# 121

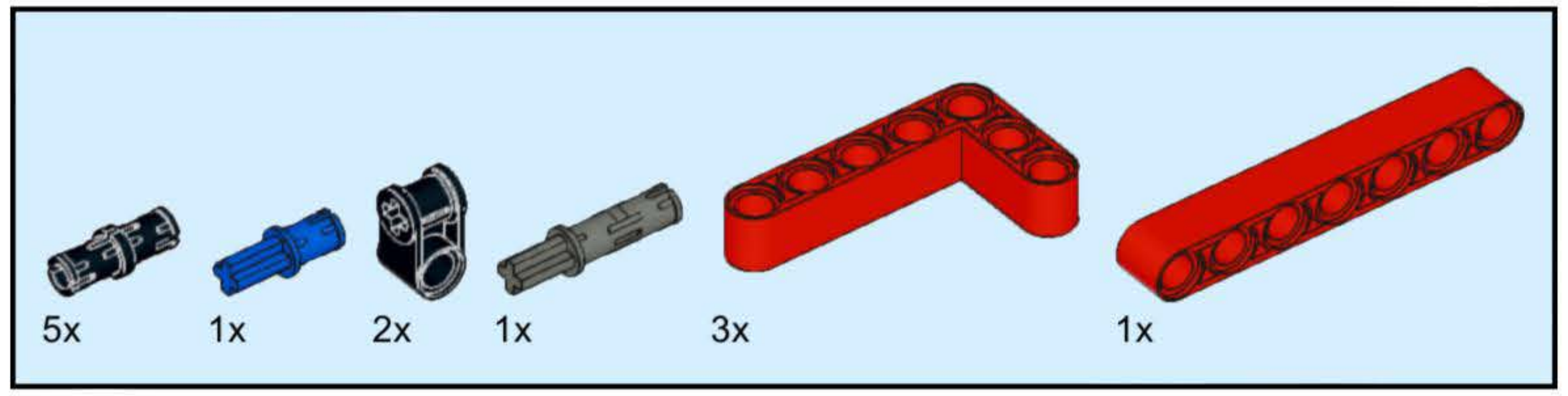


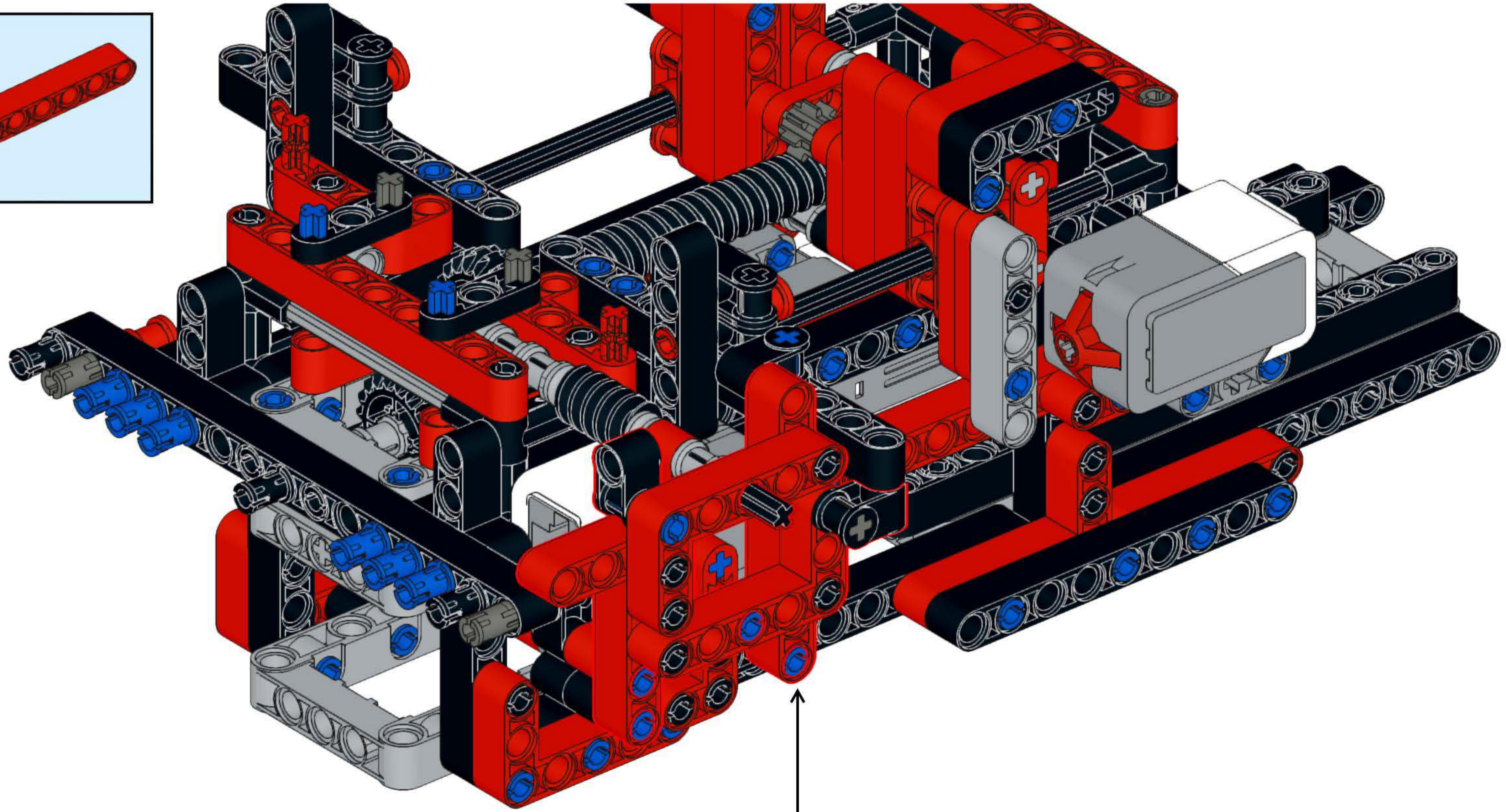

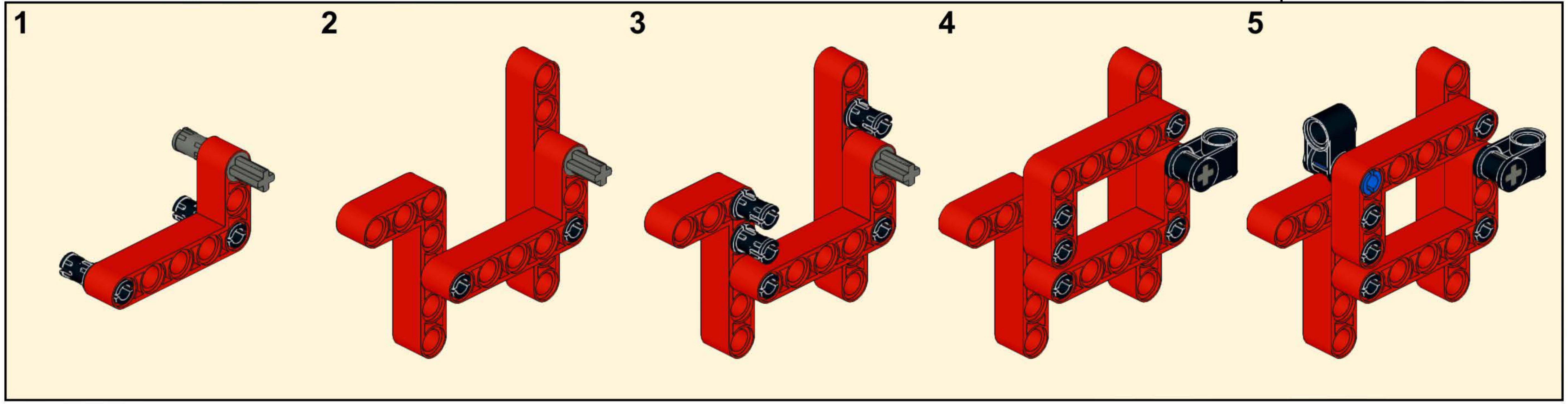


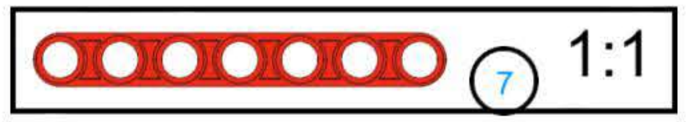

122

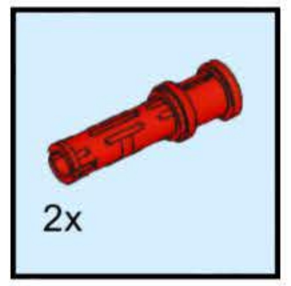


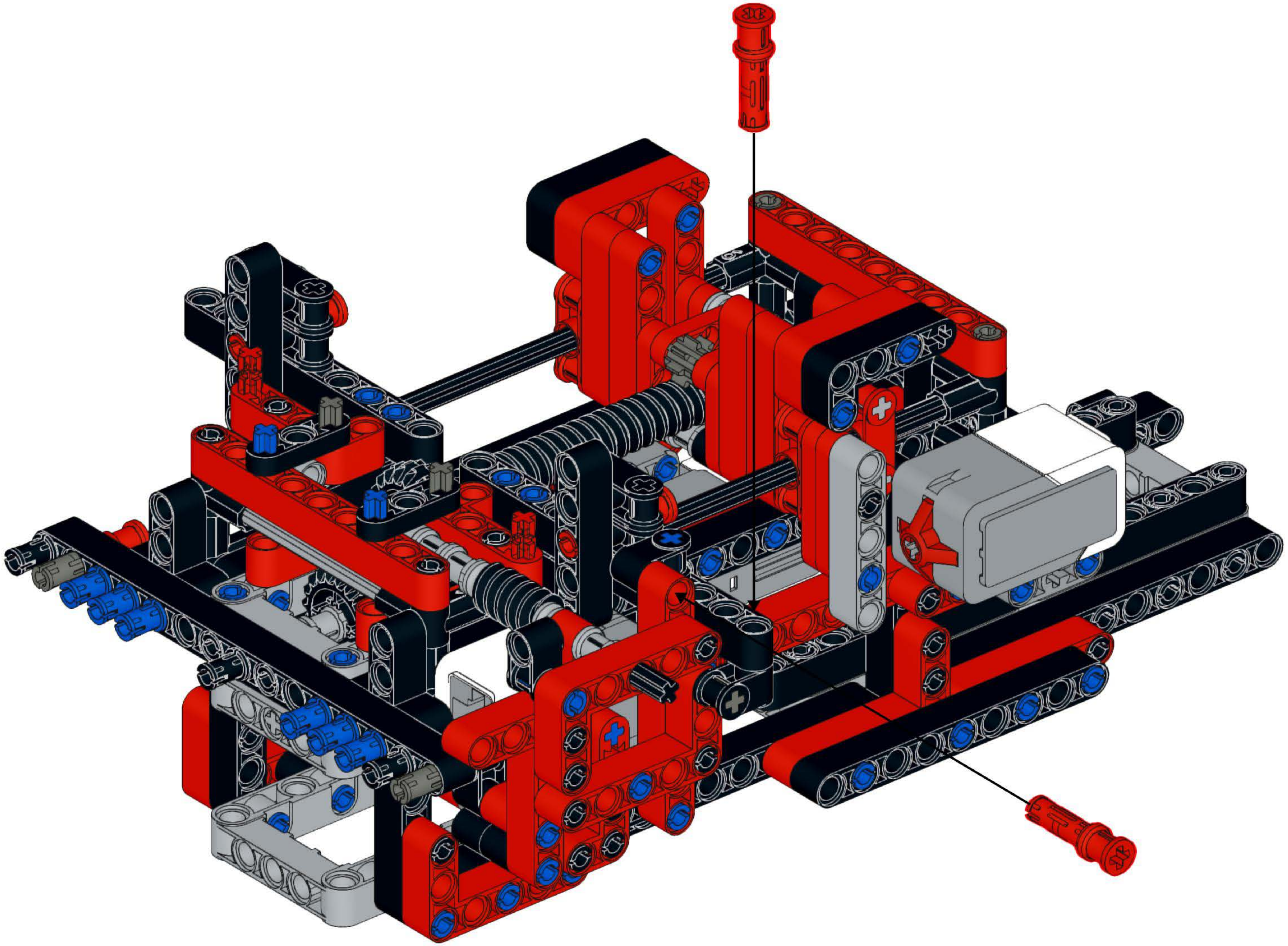

123

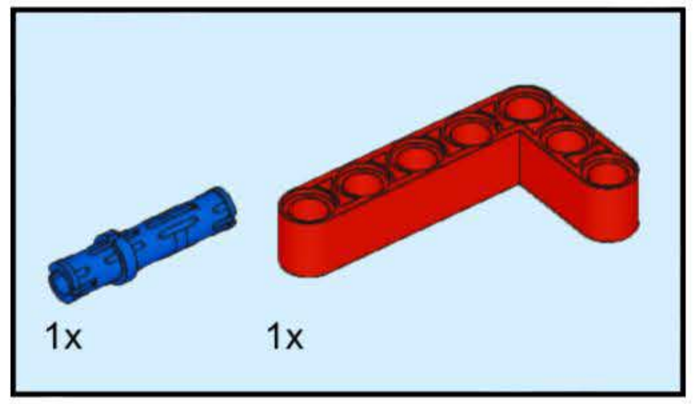


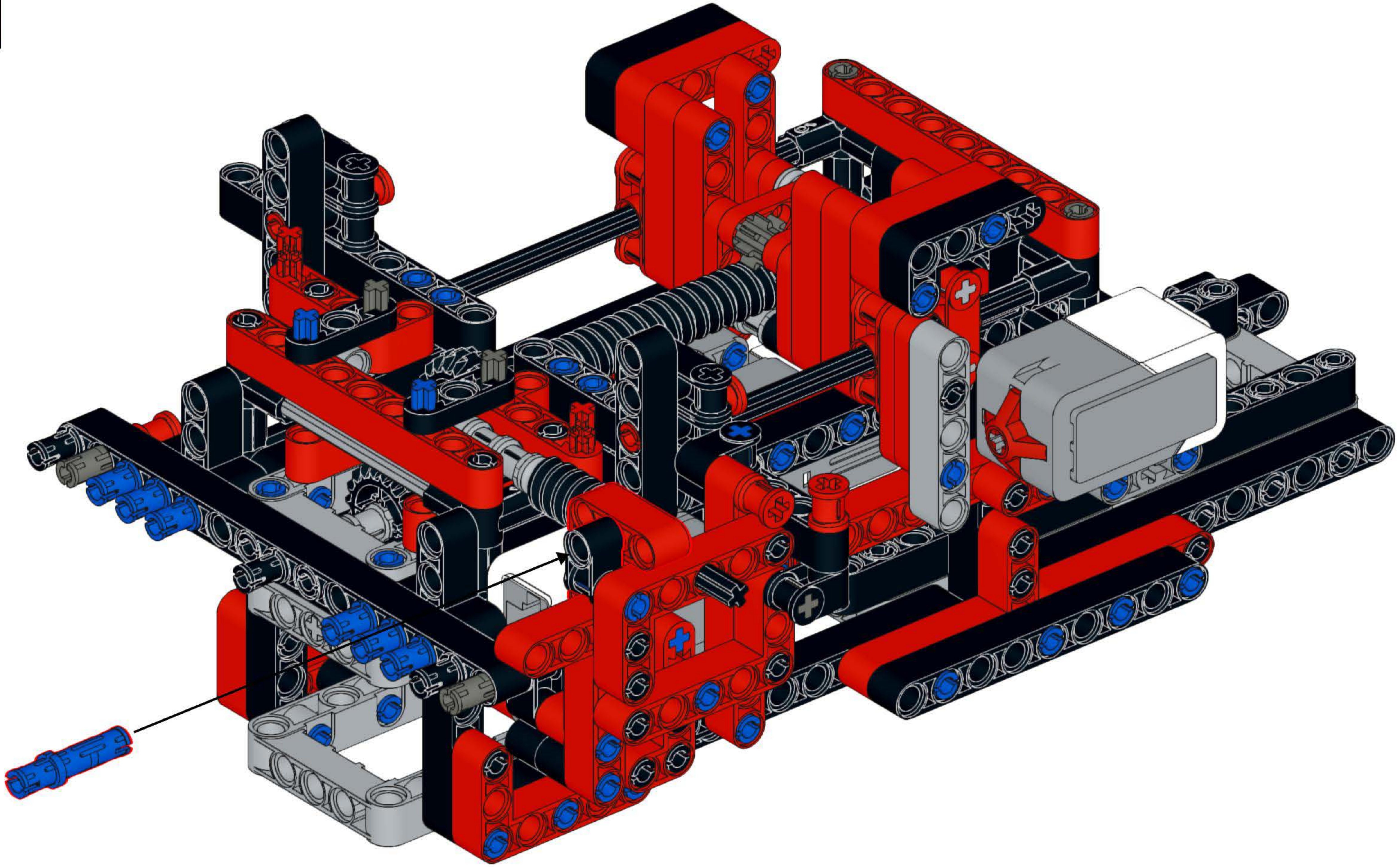

**124**

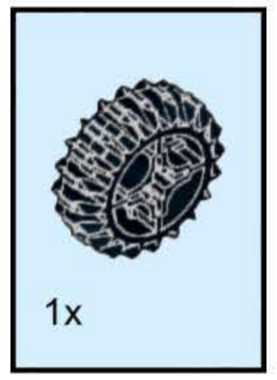


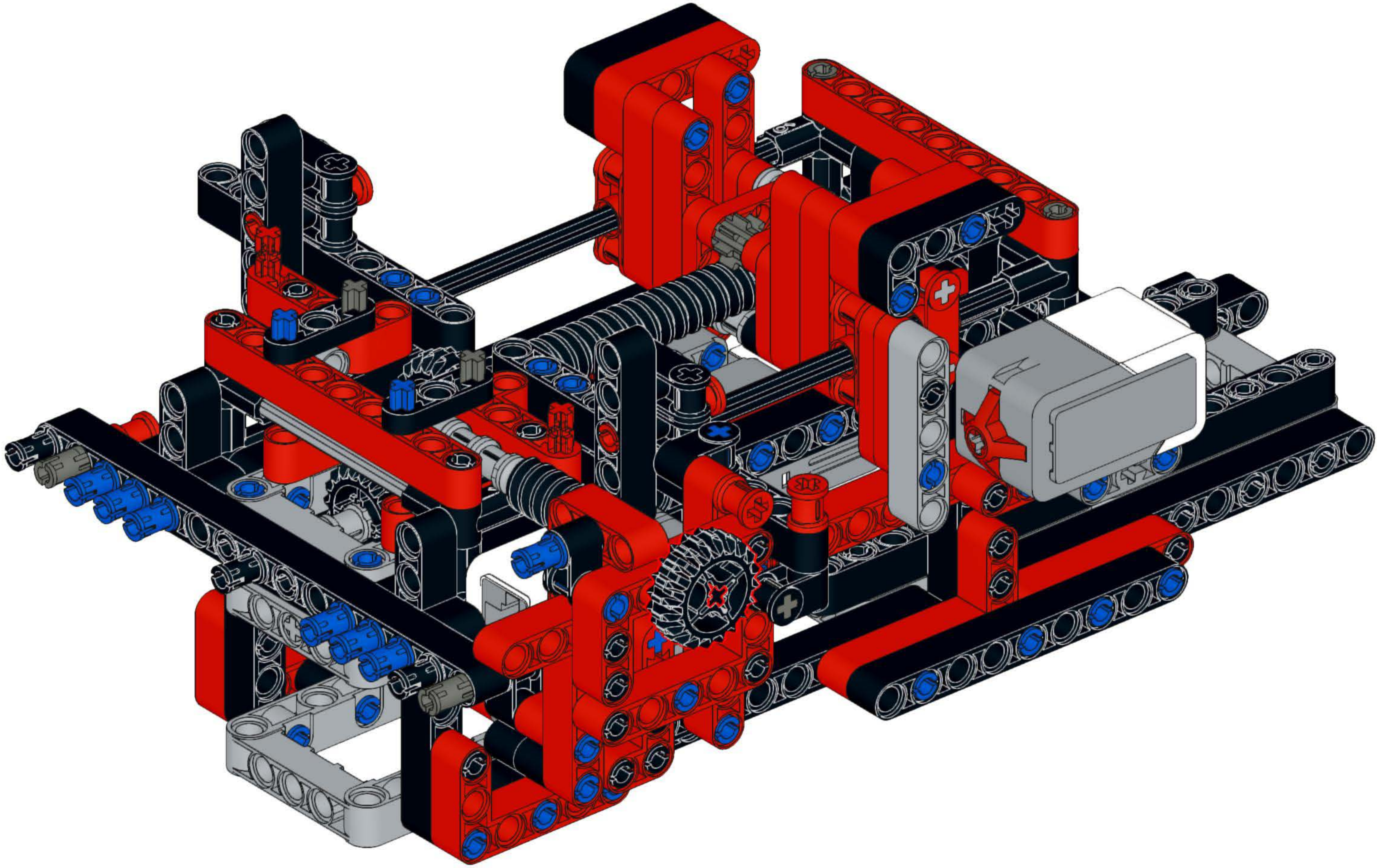

**125**

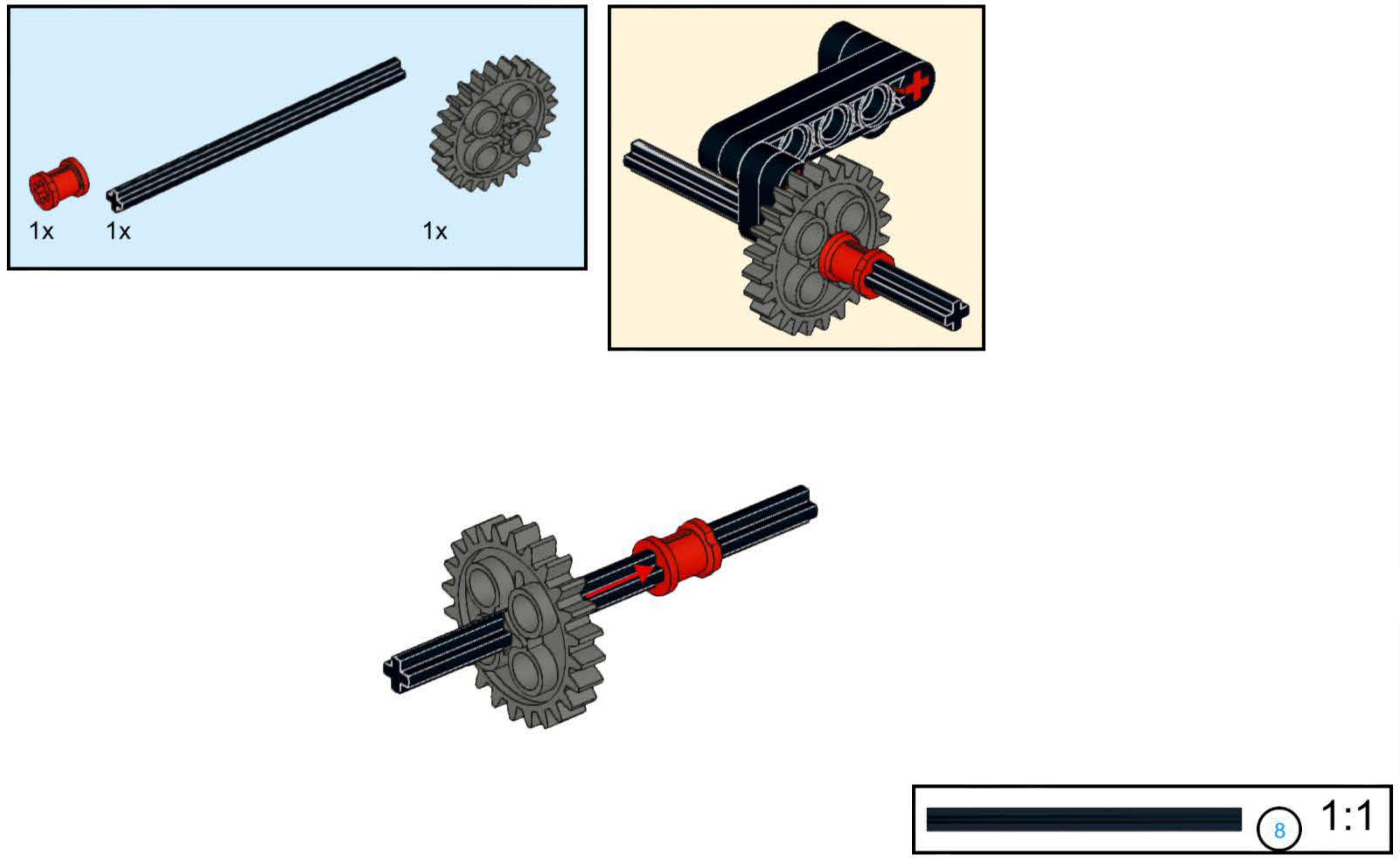


**126**

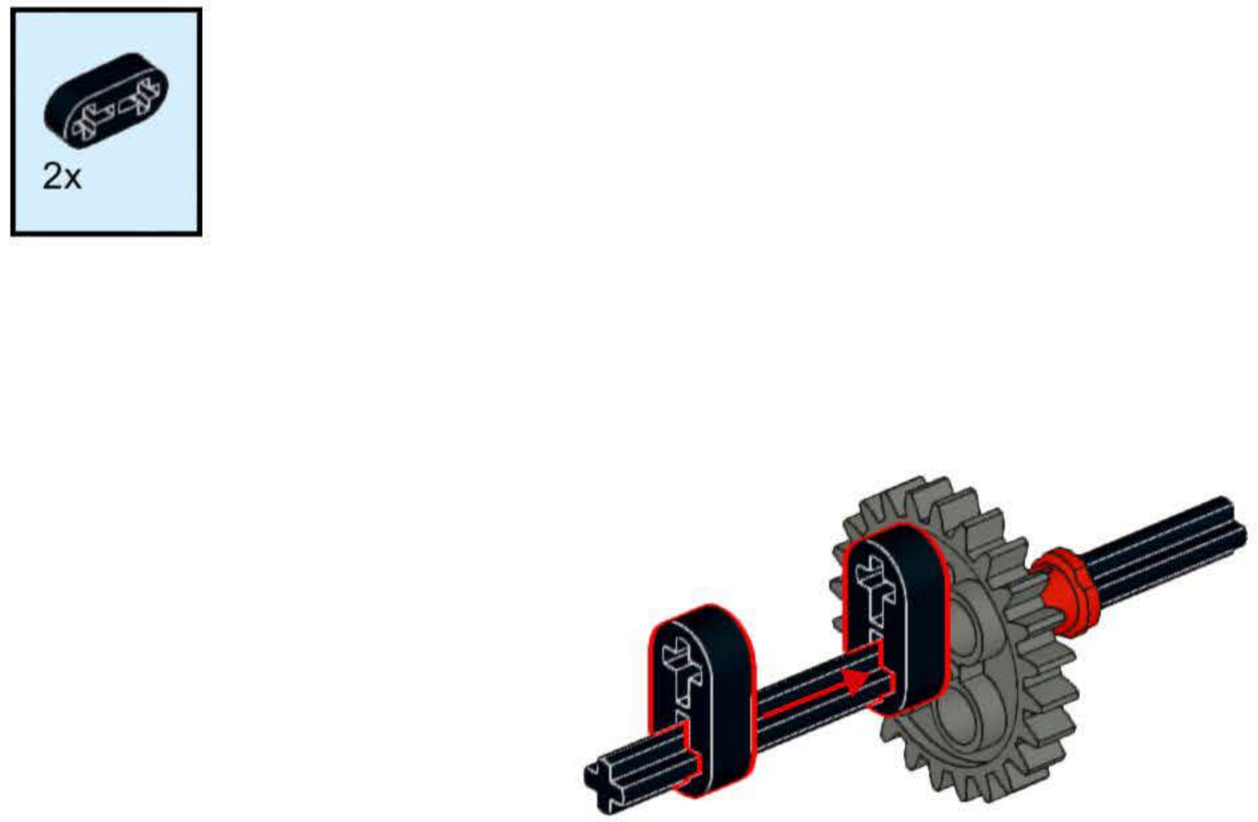


**127**

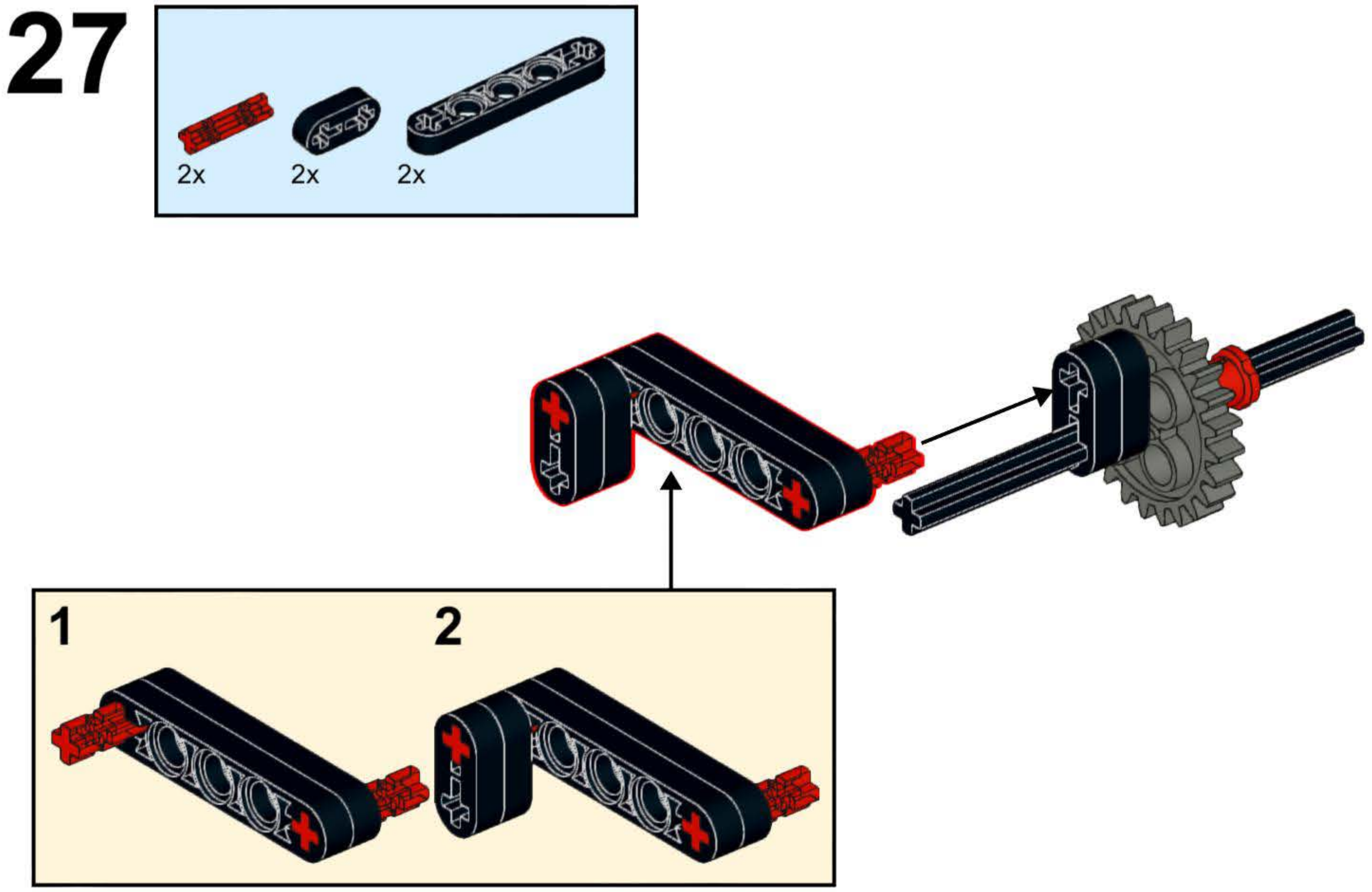


**128**

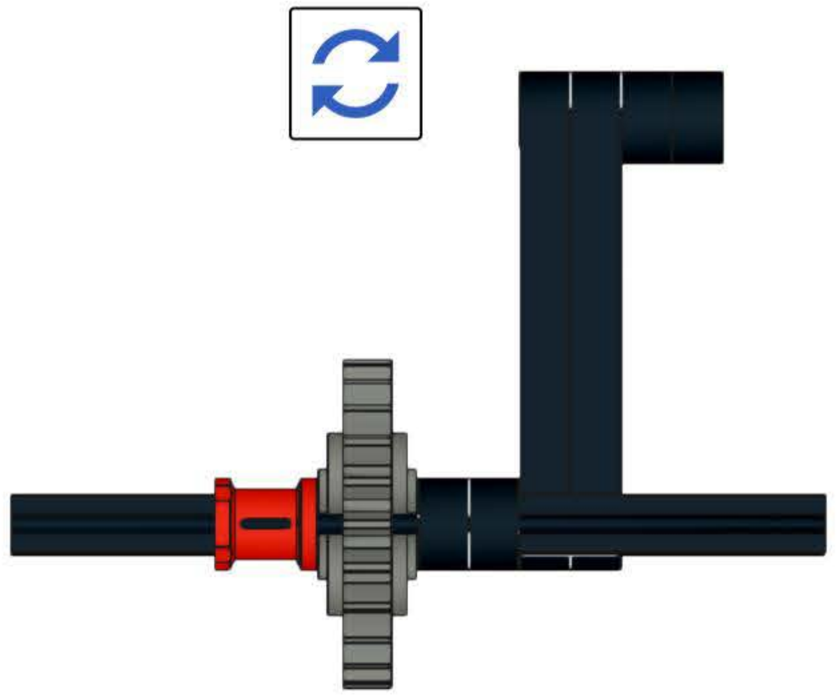

129

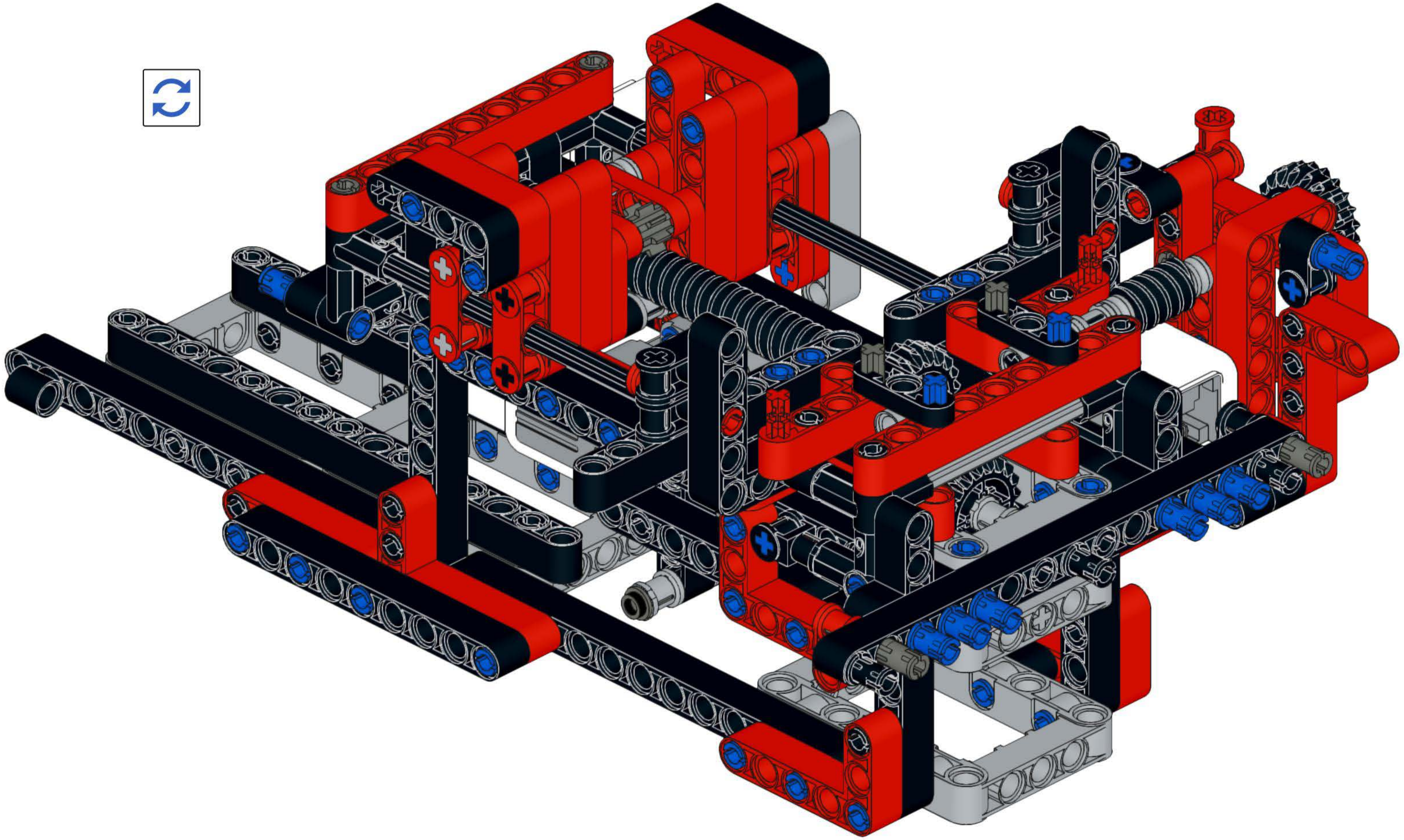

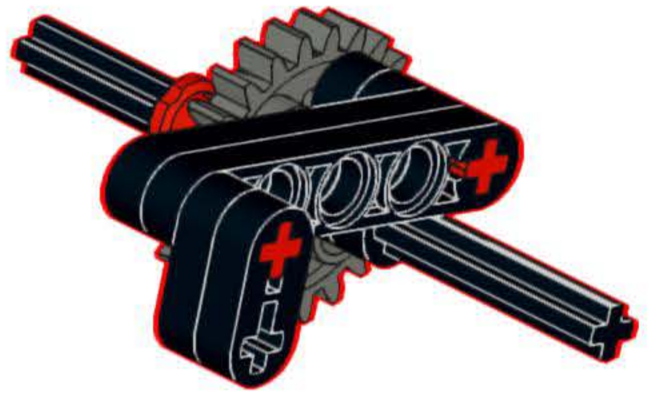

**130**

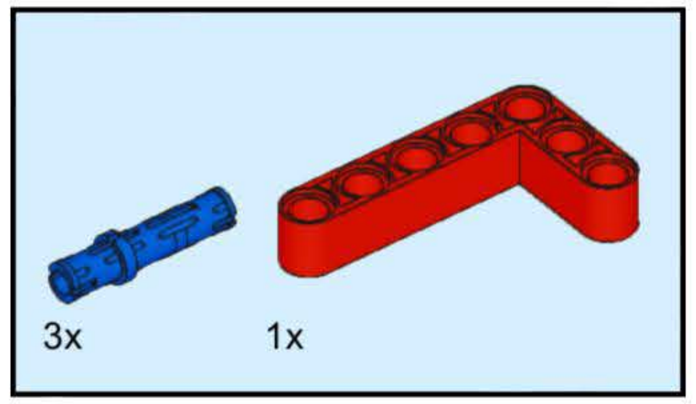


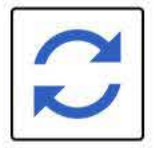

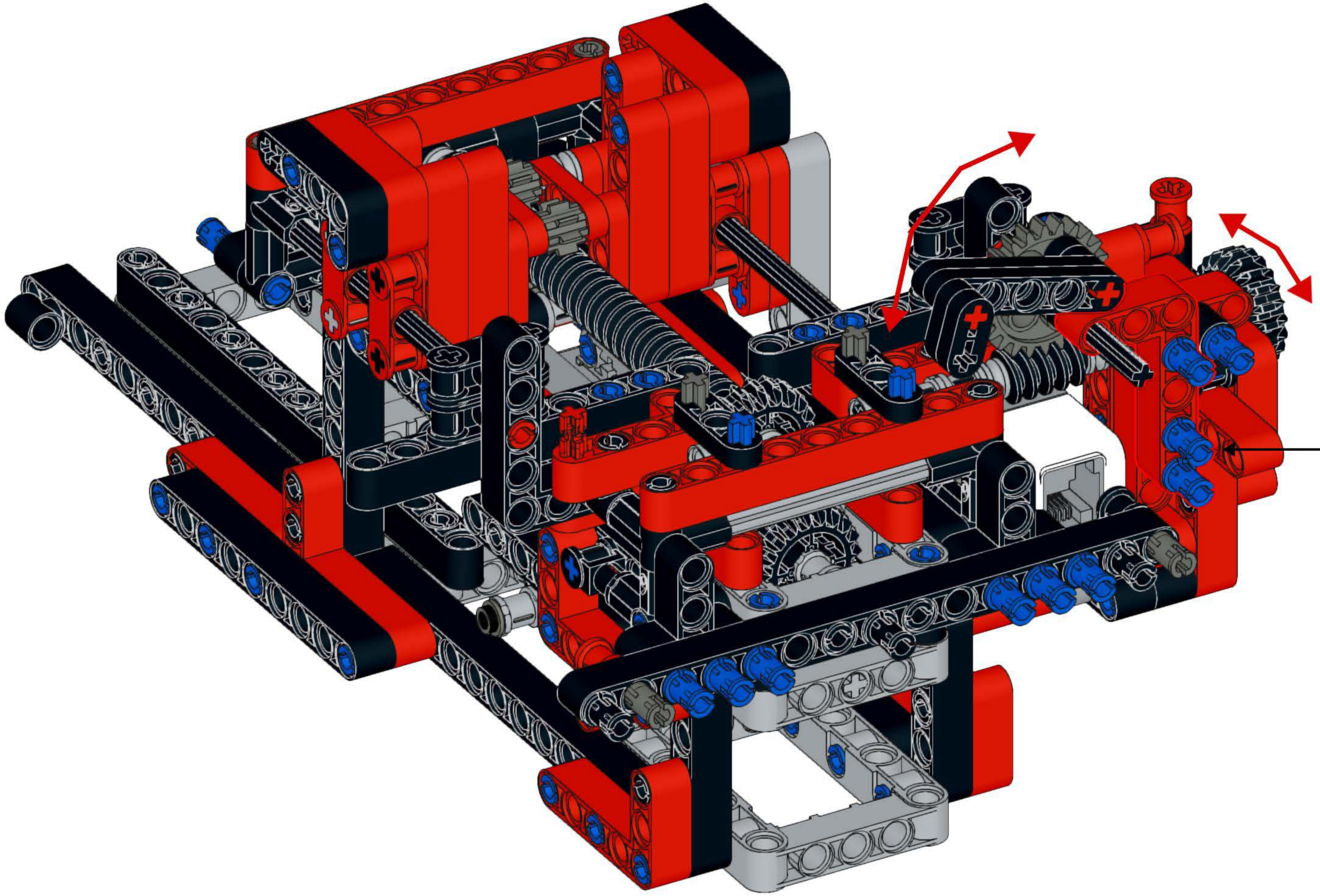

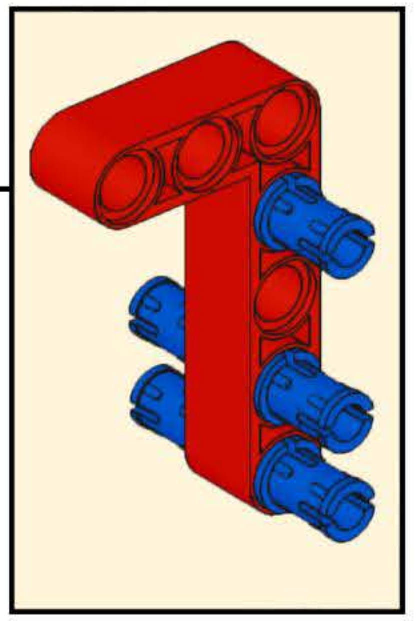

131

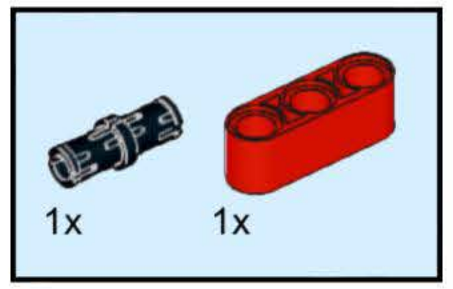


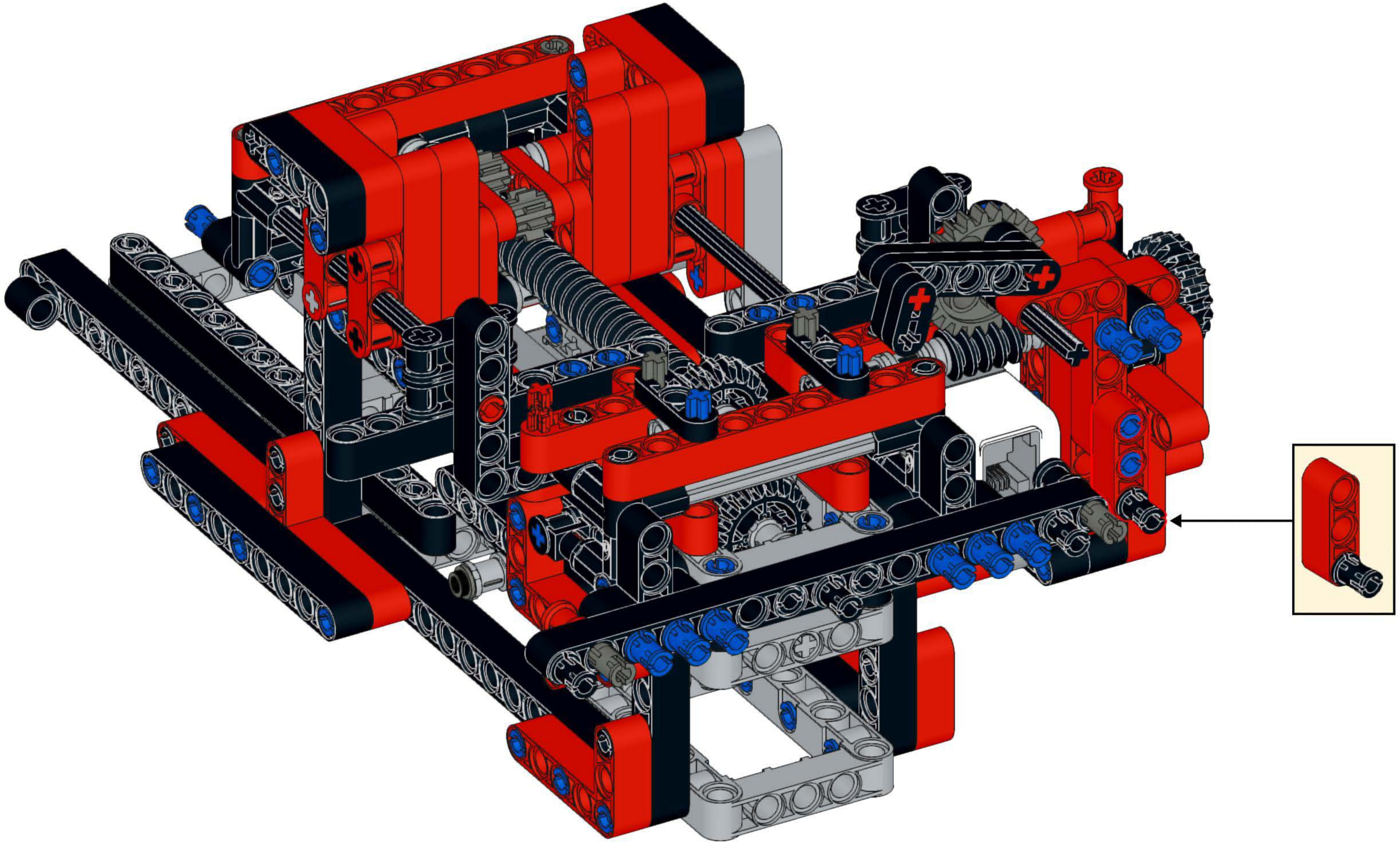

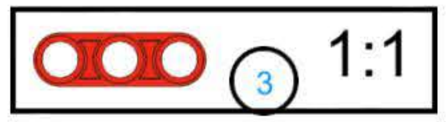

132

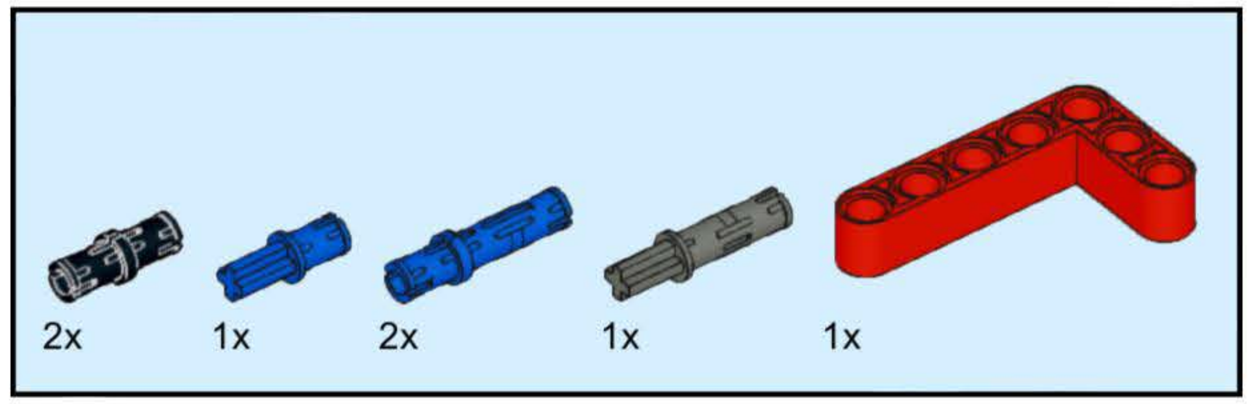


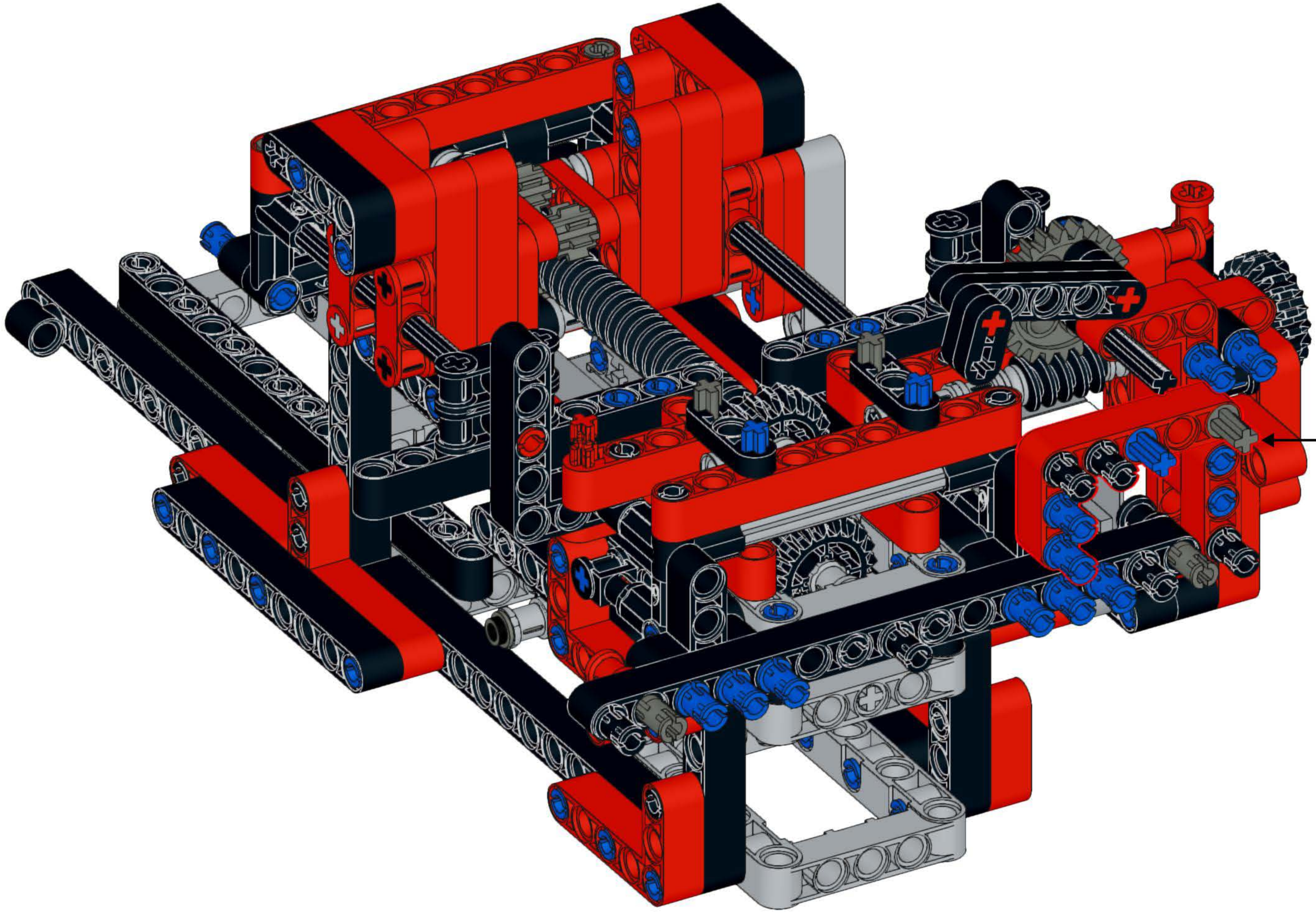

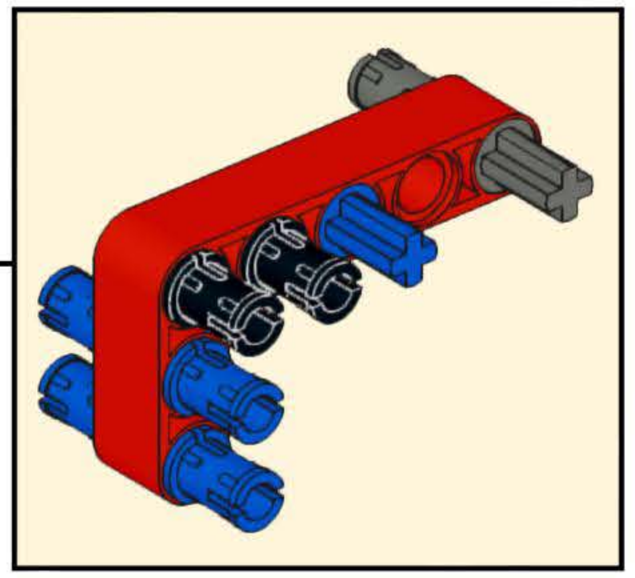

133

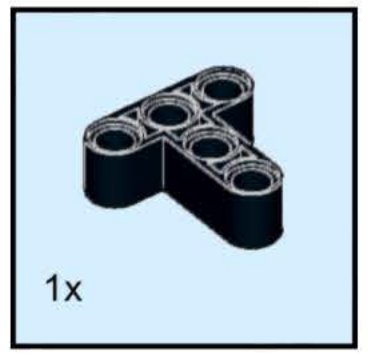


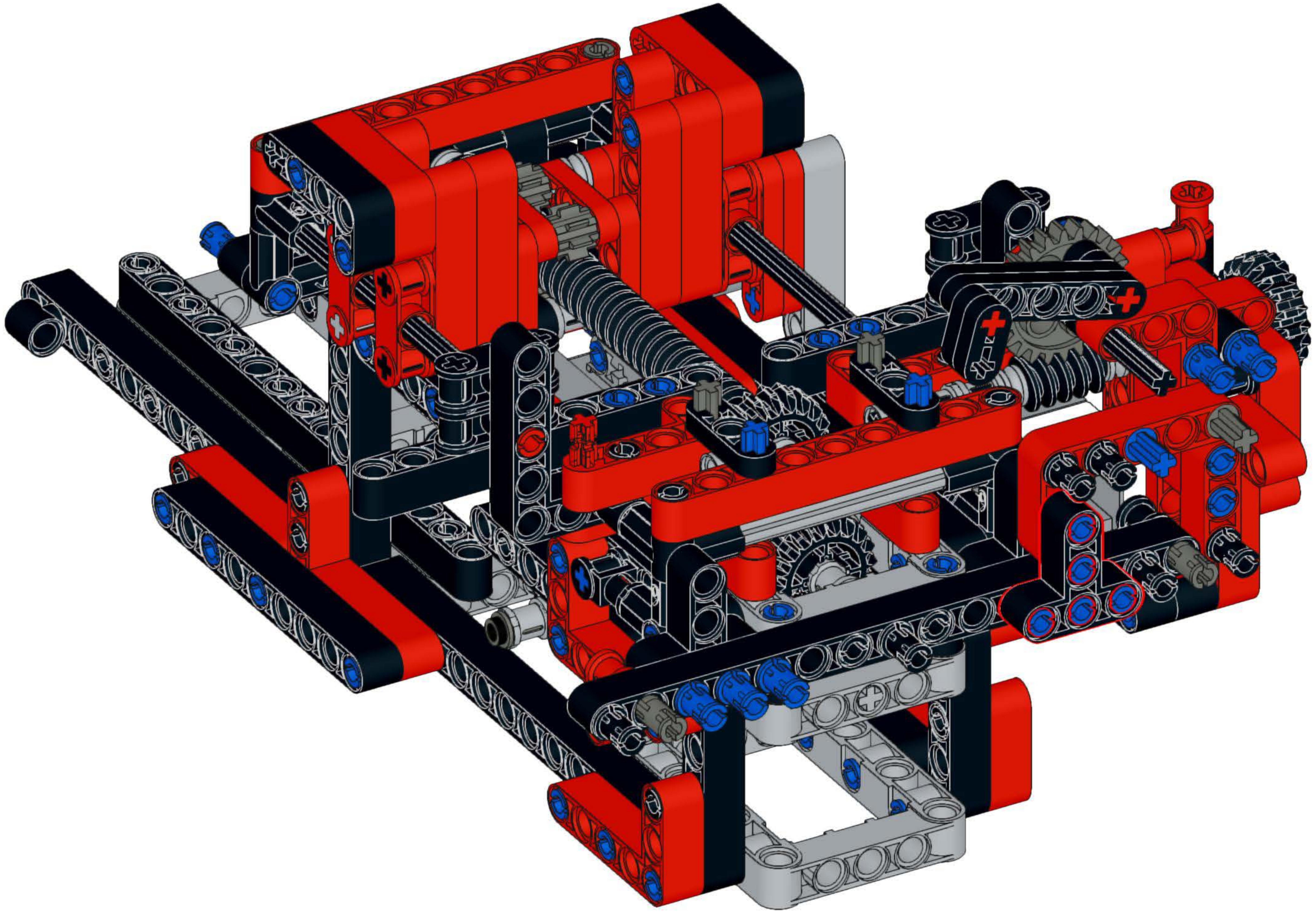

# 134

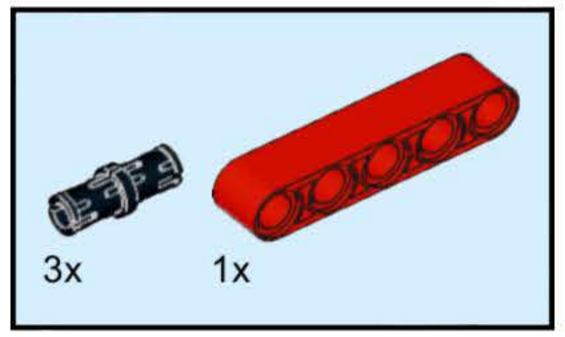


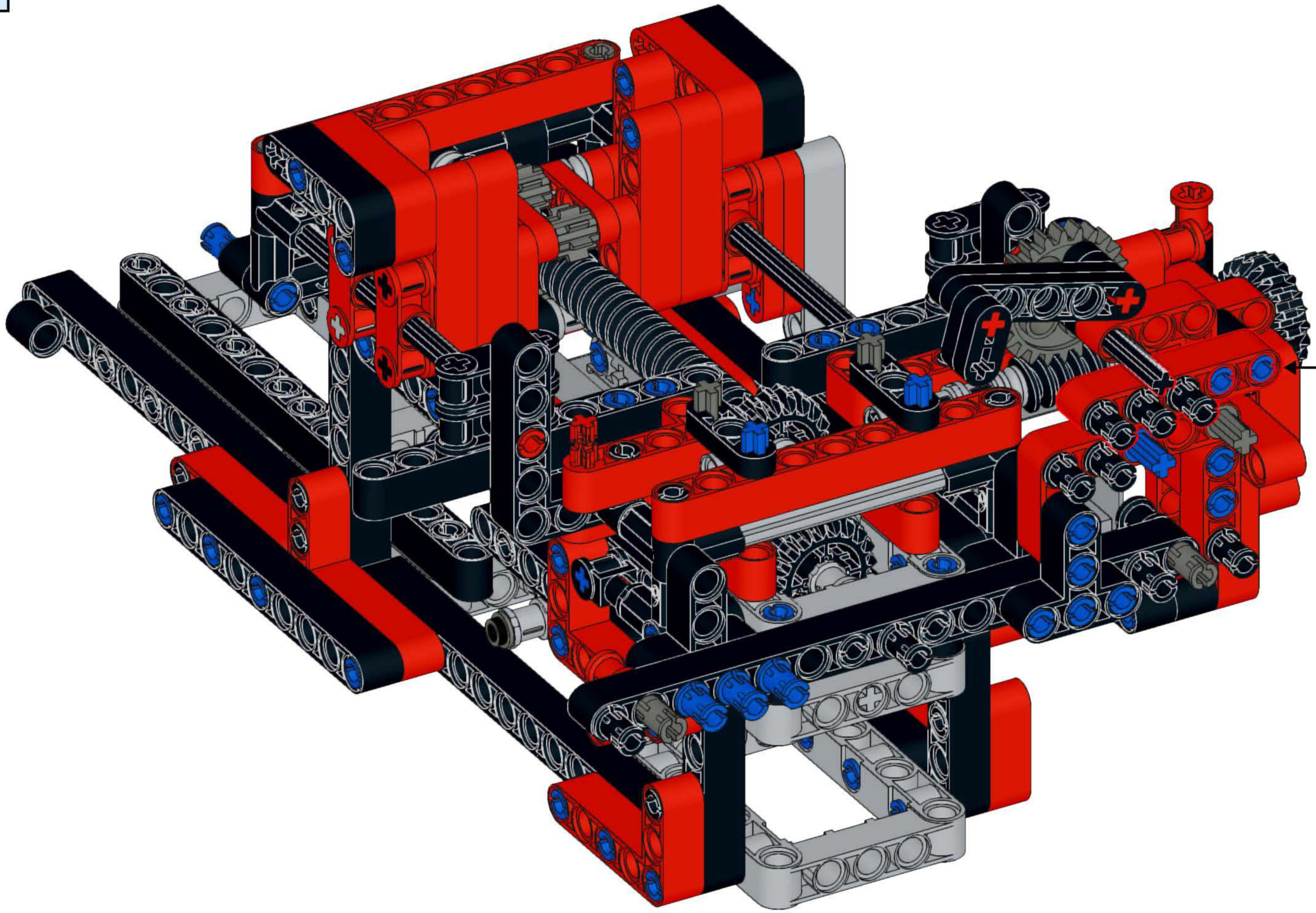

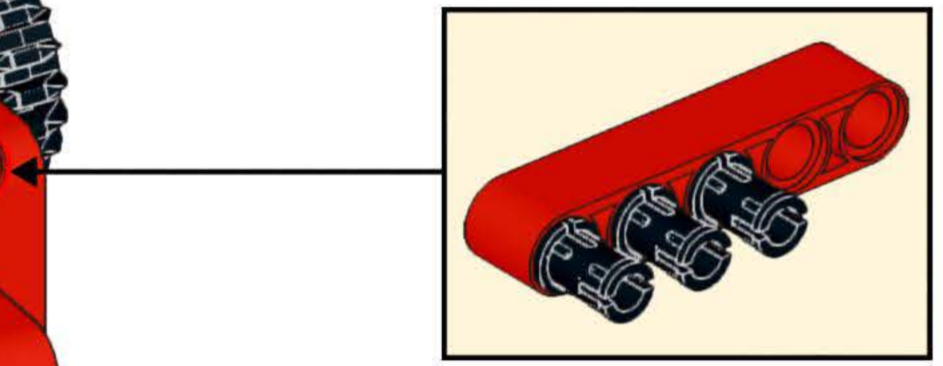

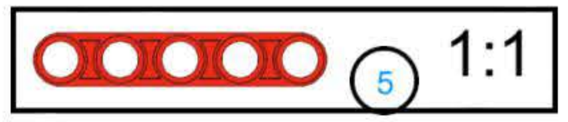

135

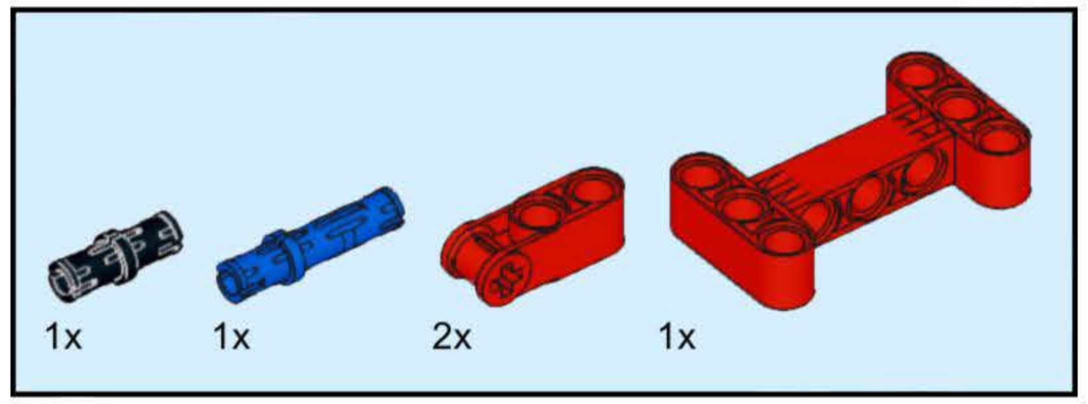


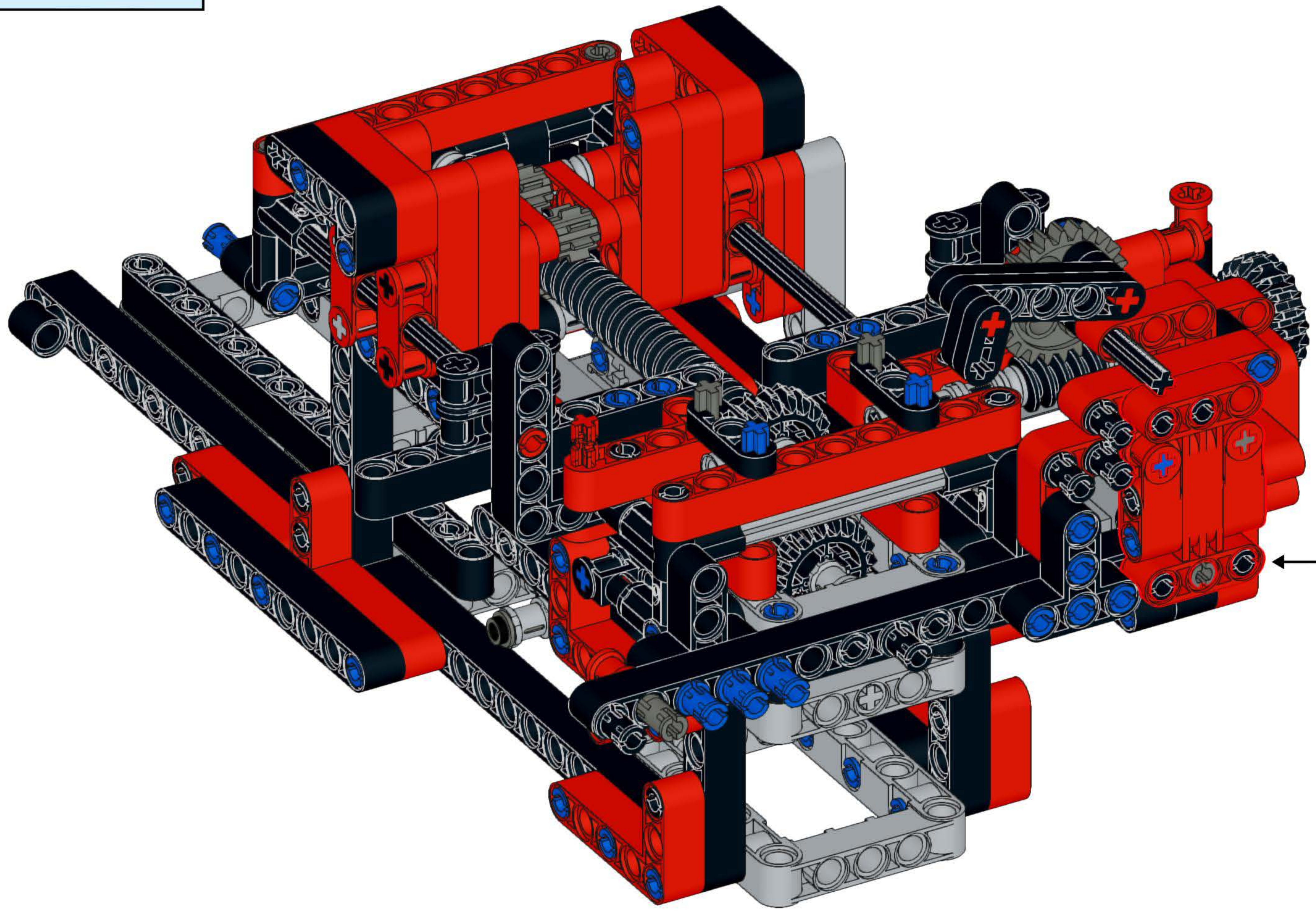

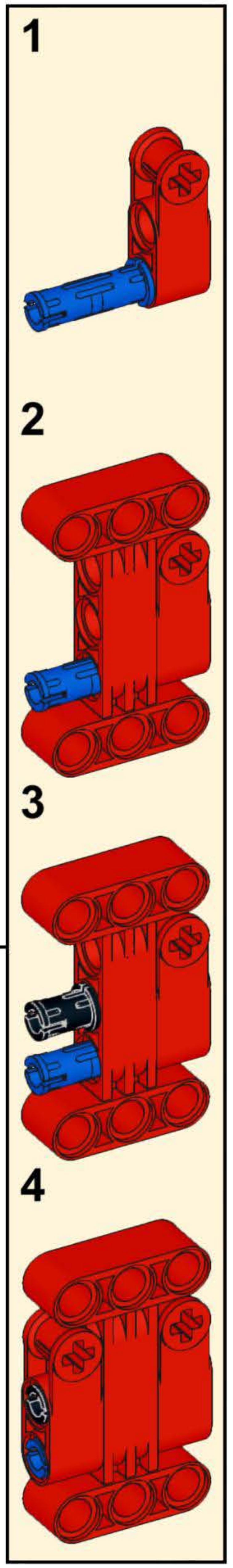

136

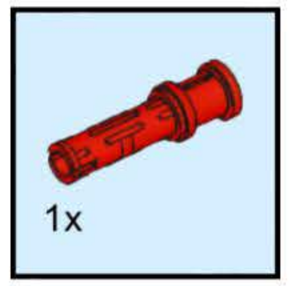


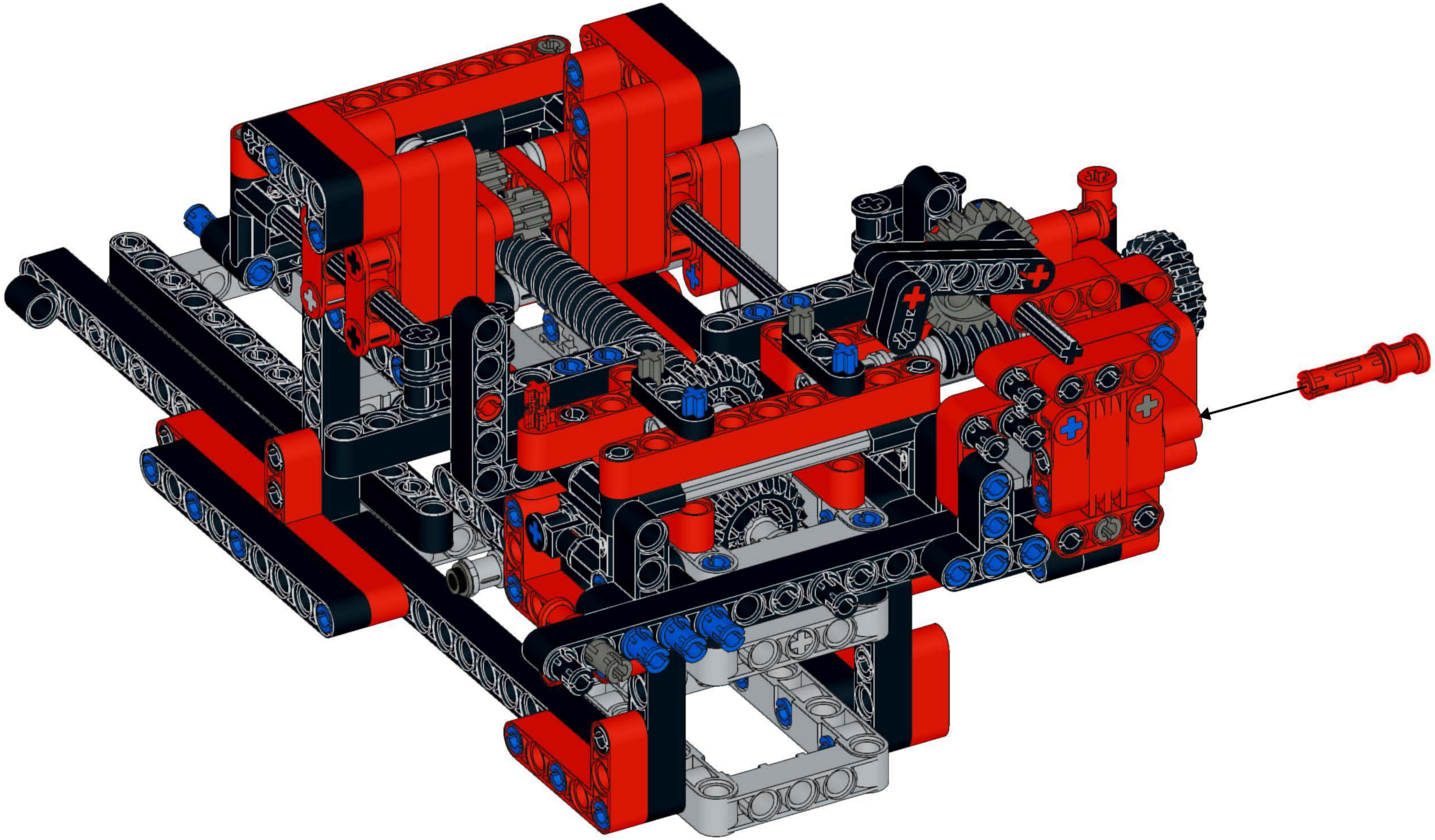

# 137

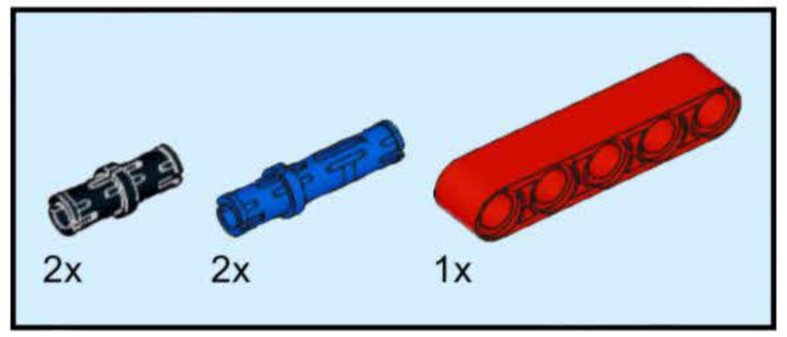


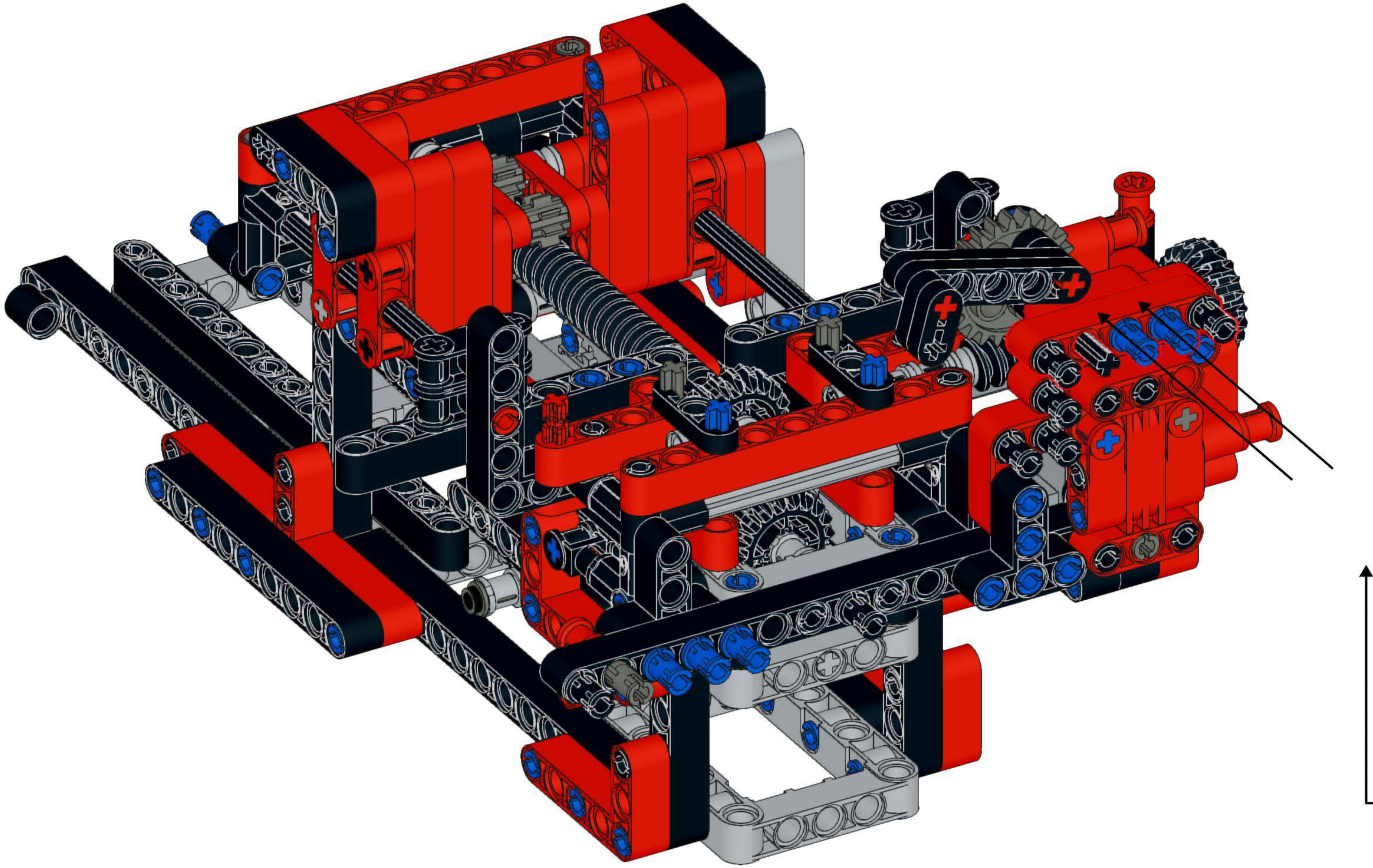

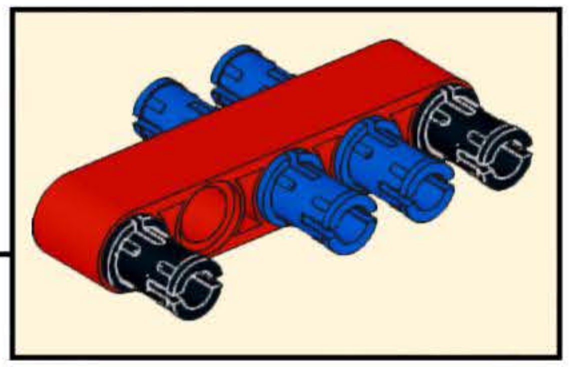

138

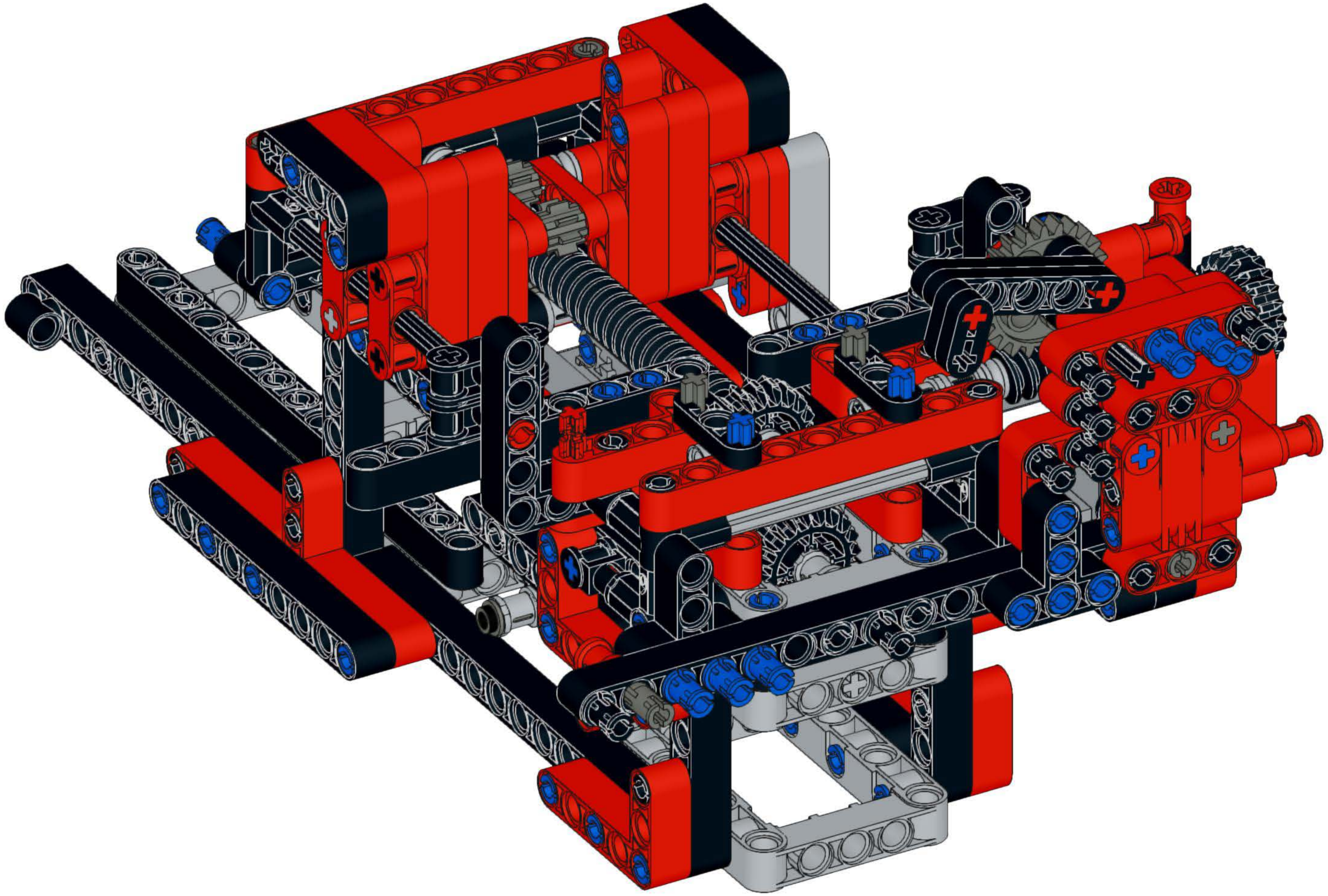

139

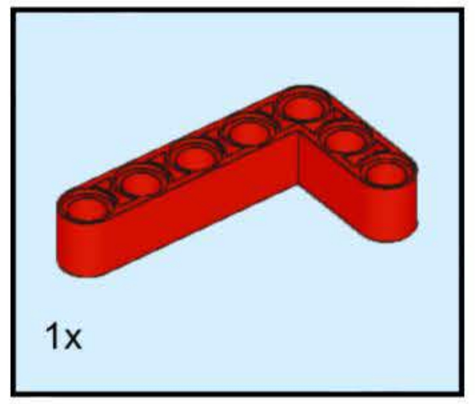


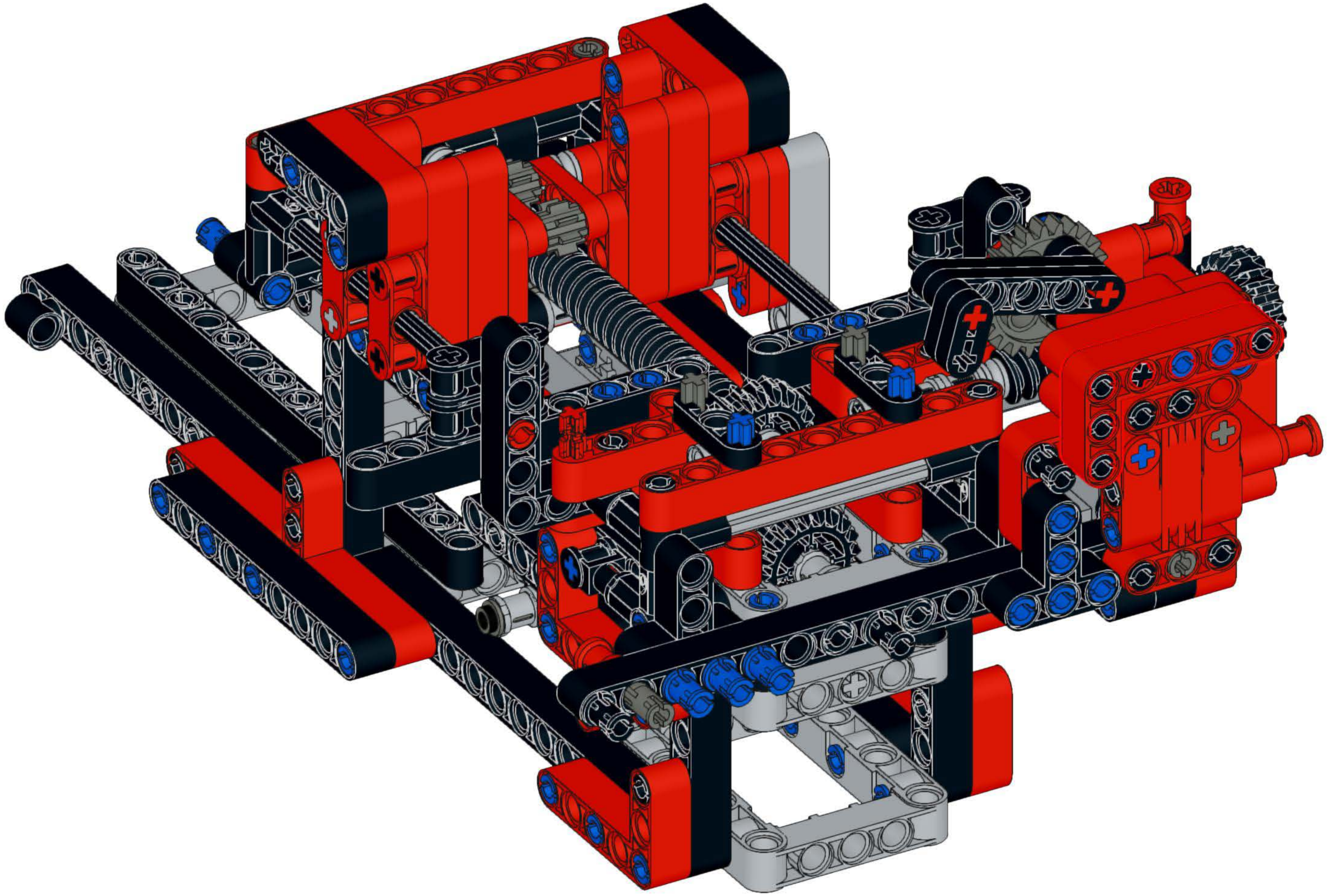

140

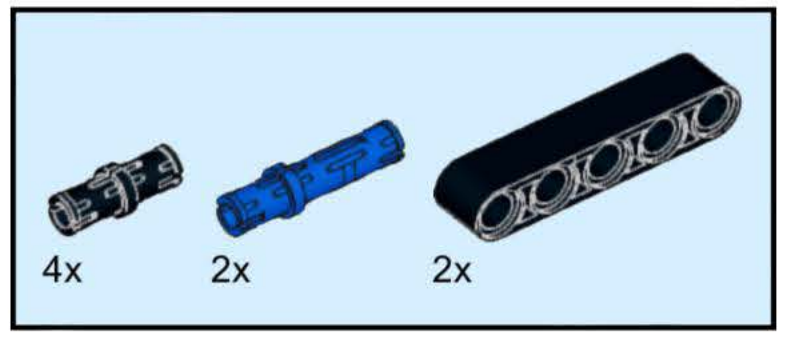


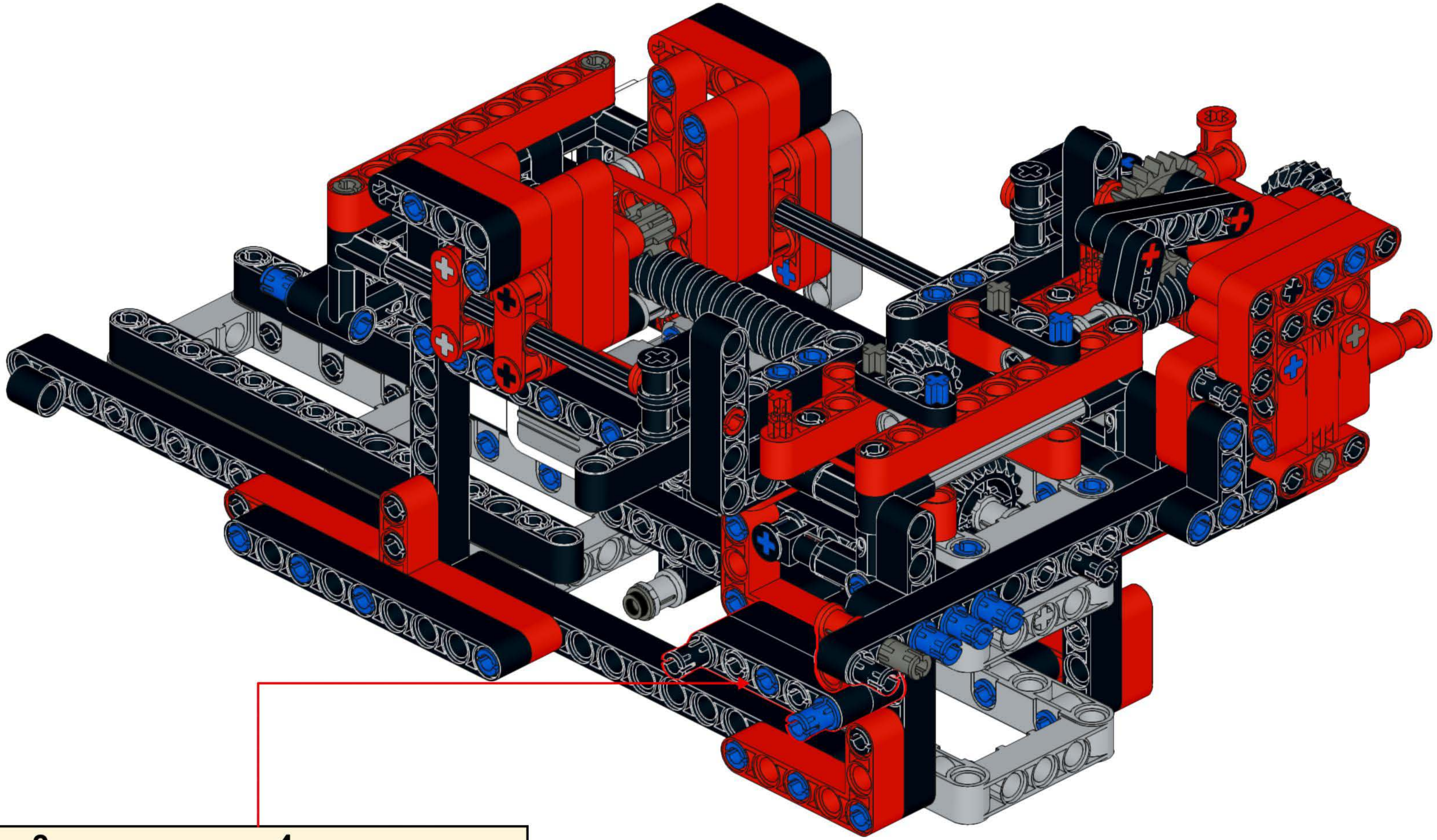

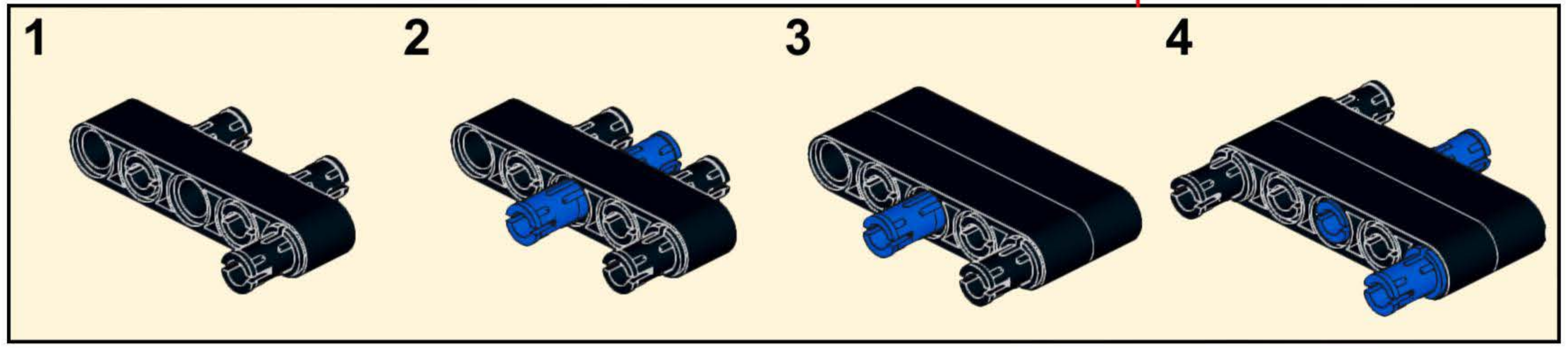


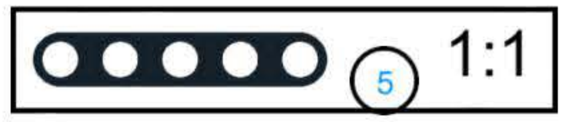

141

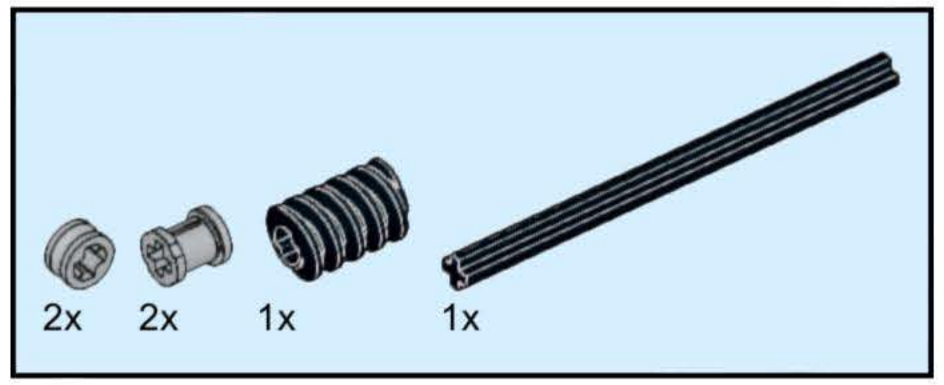


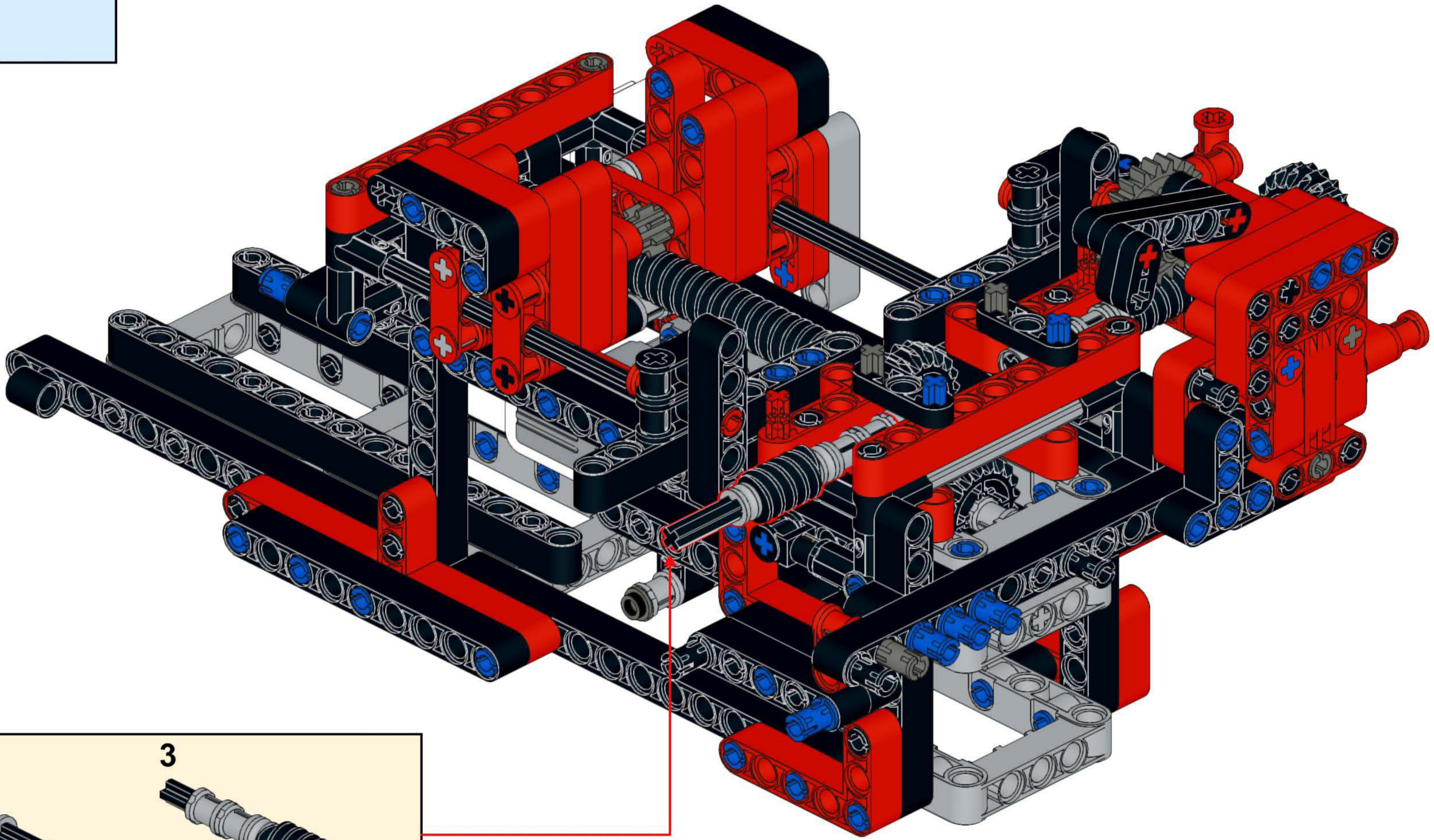

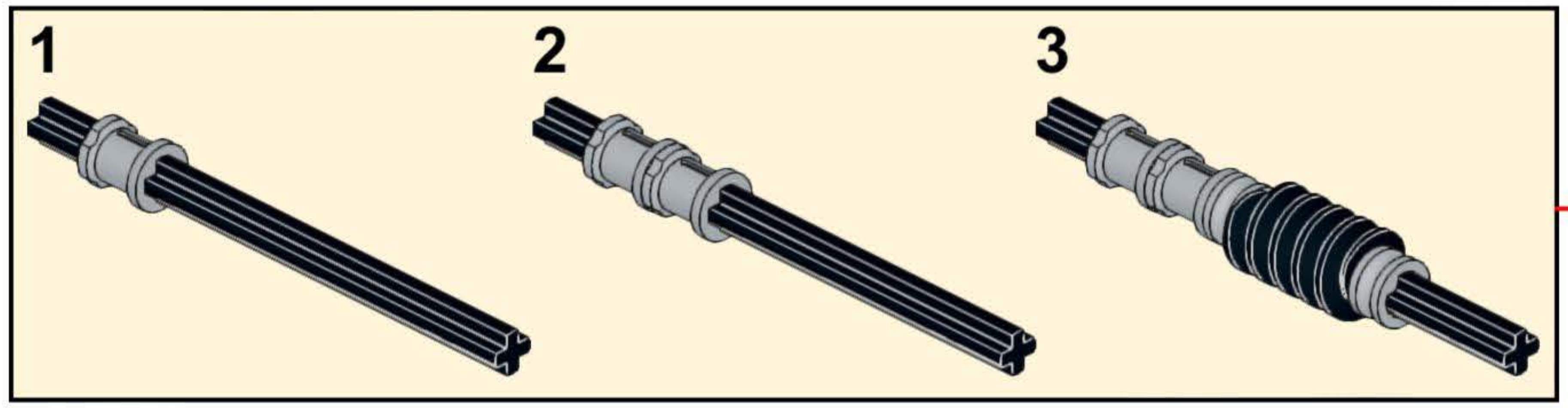


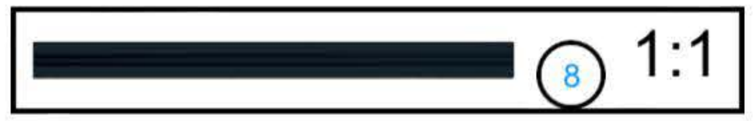

142

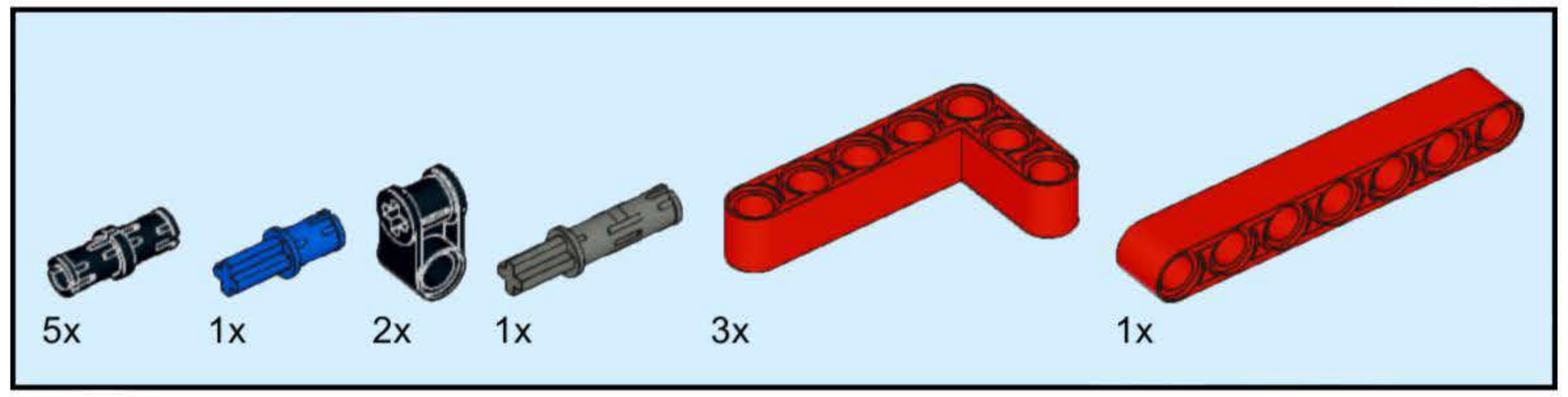


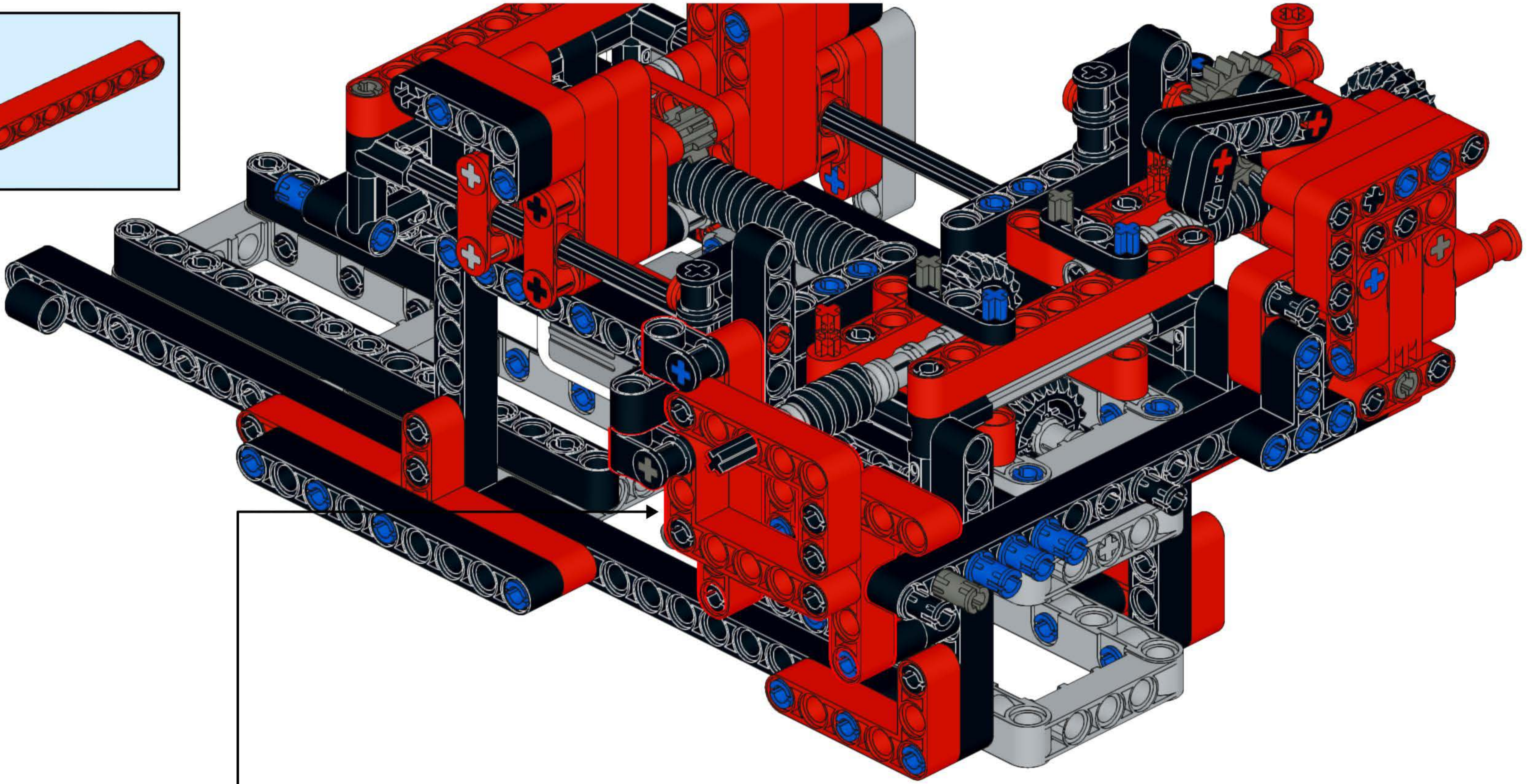

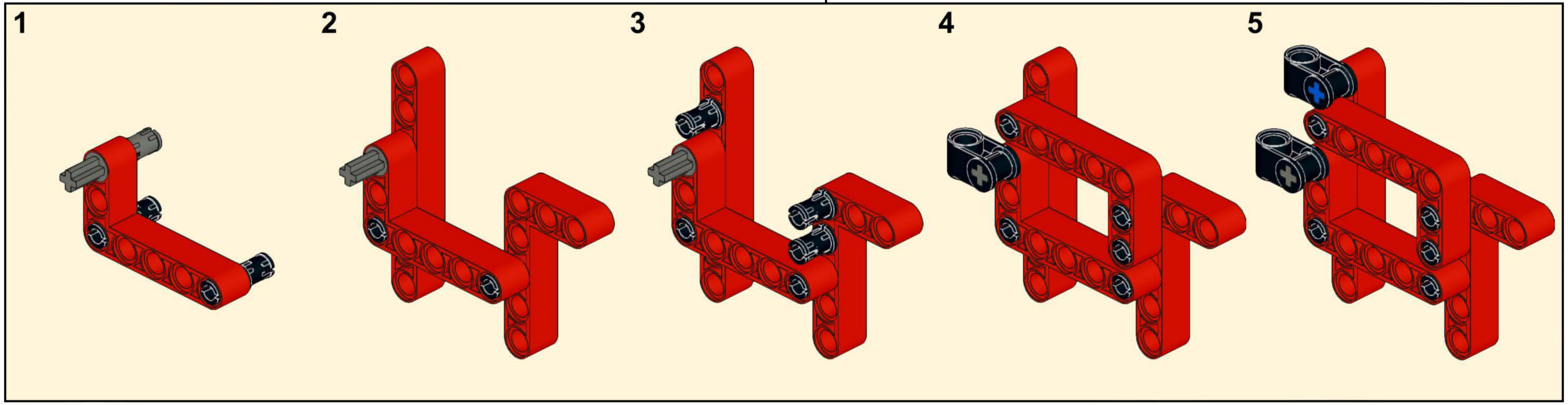


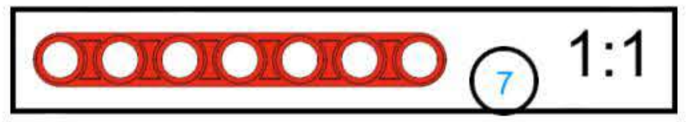

143

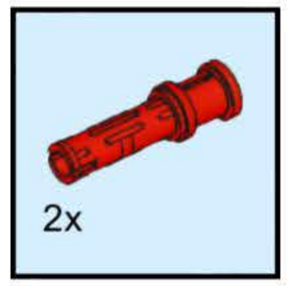


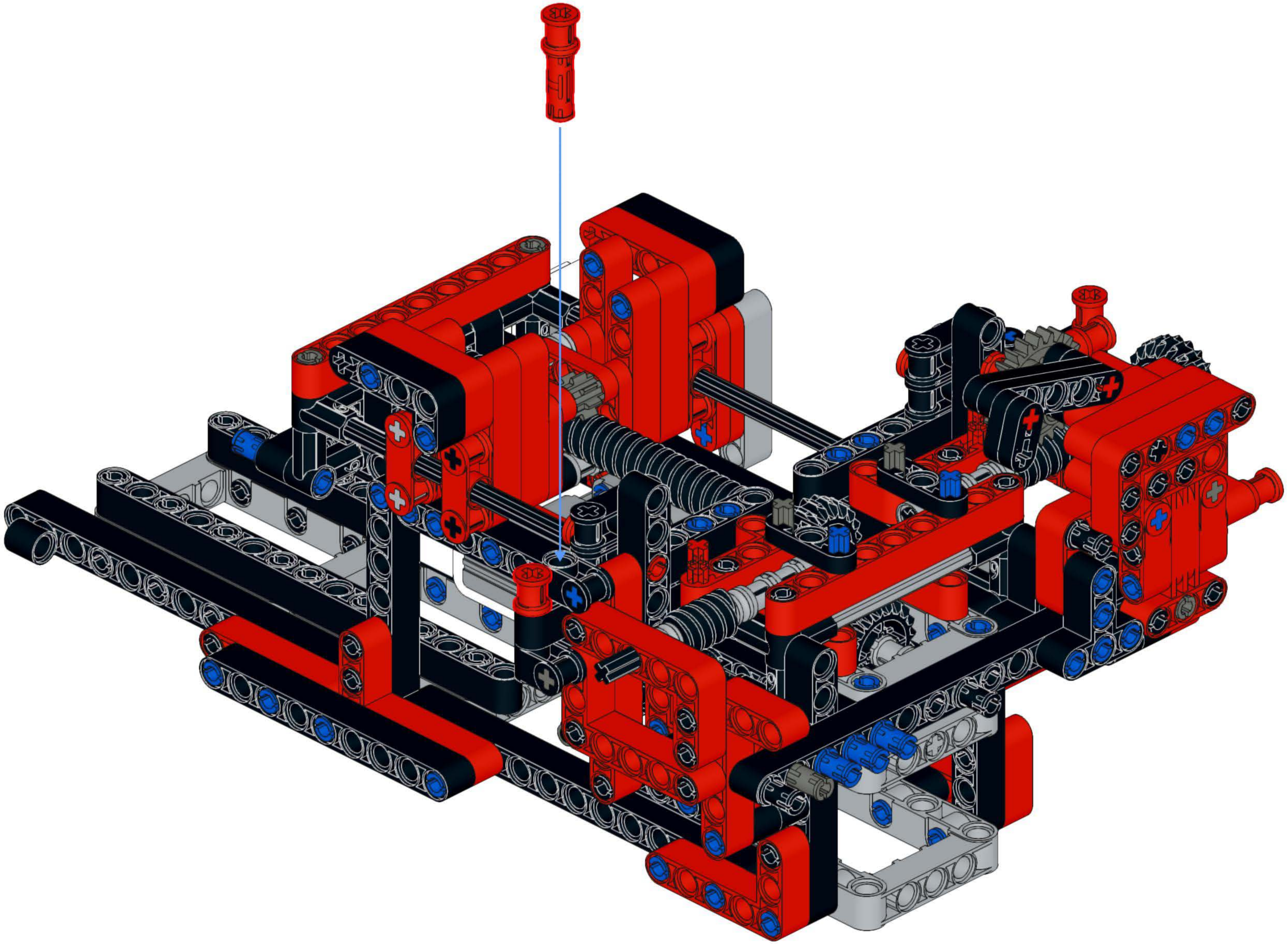

# 144

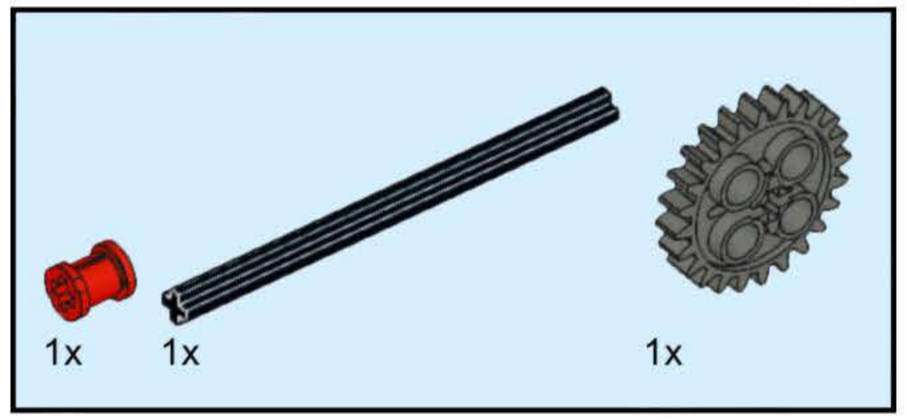


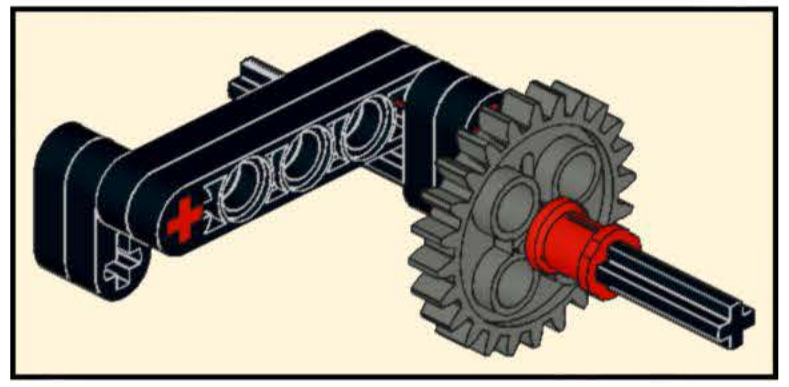

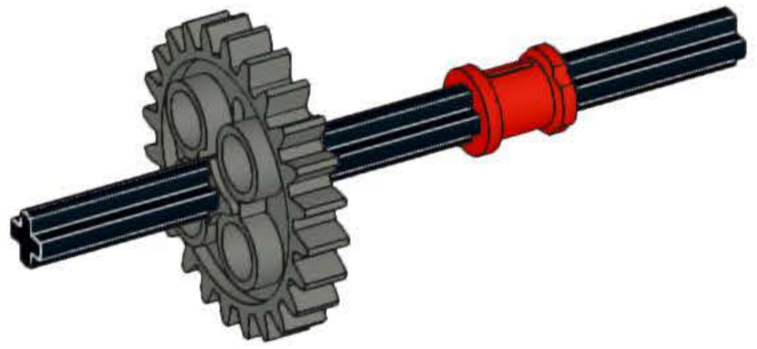

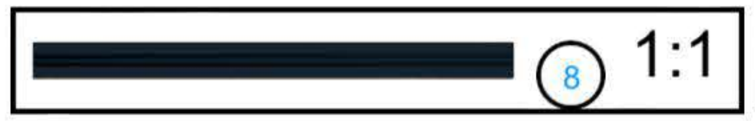


# 145

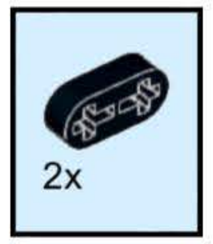


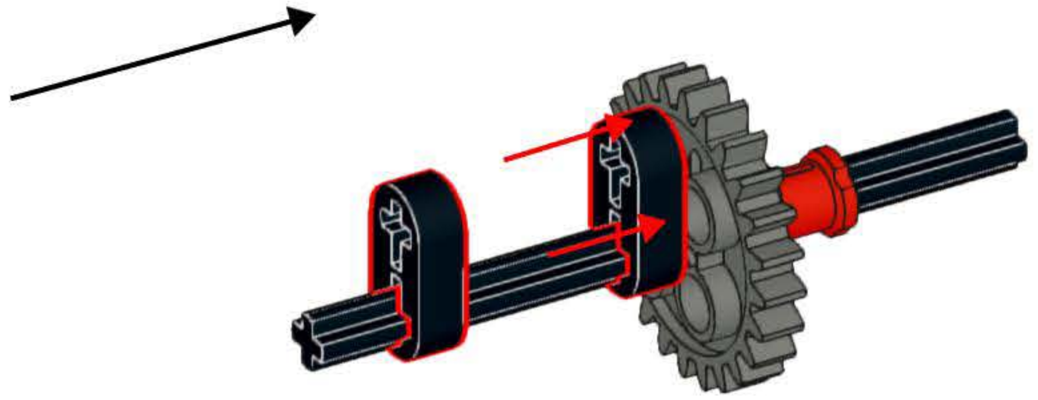

# 146

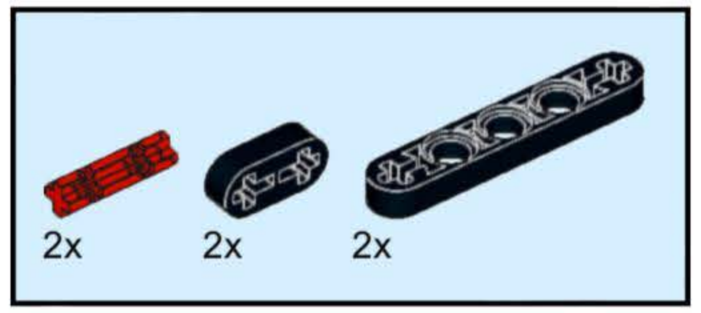


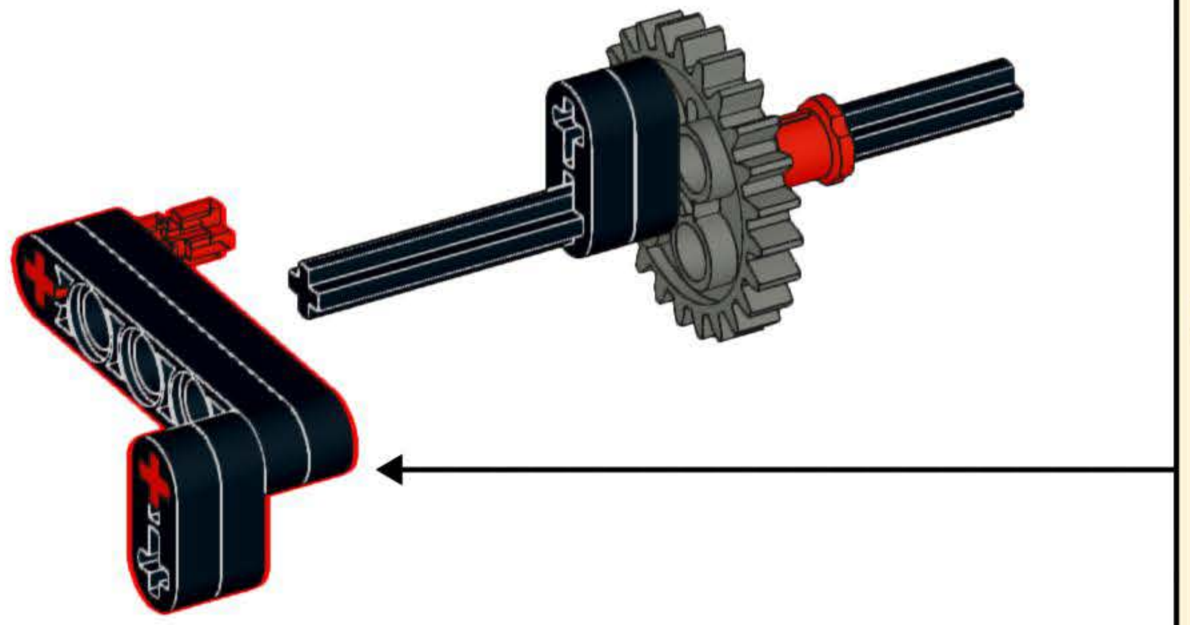

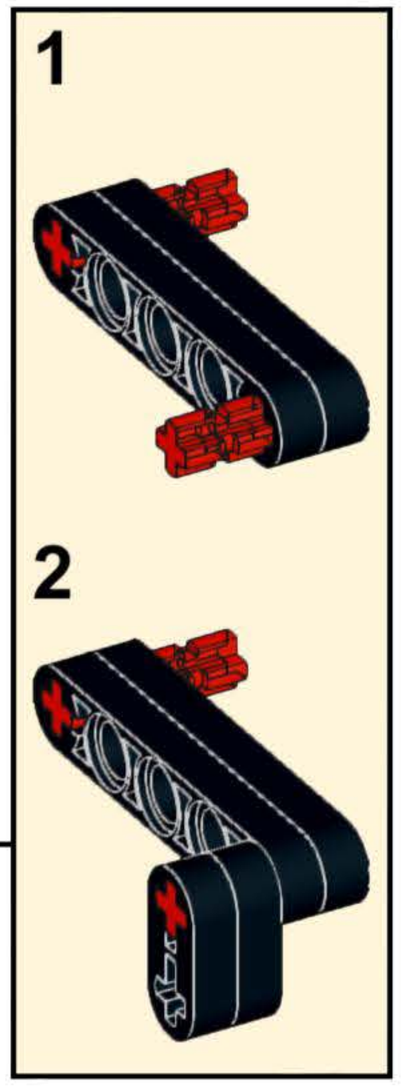


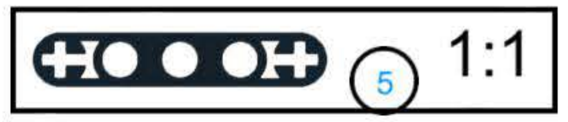


# 147

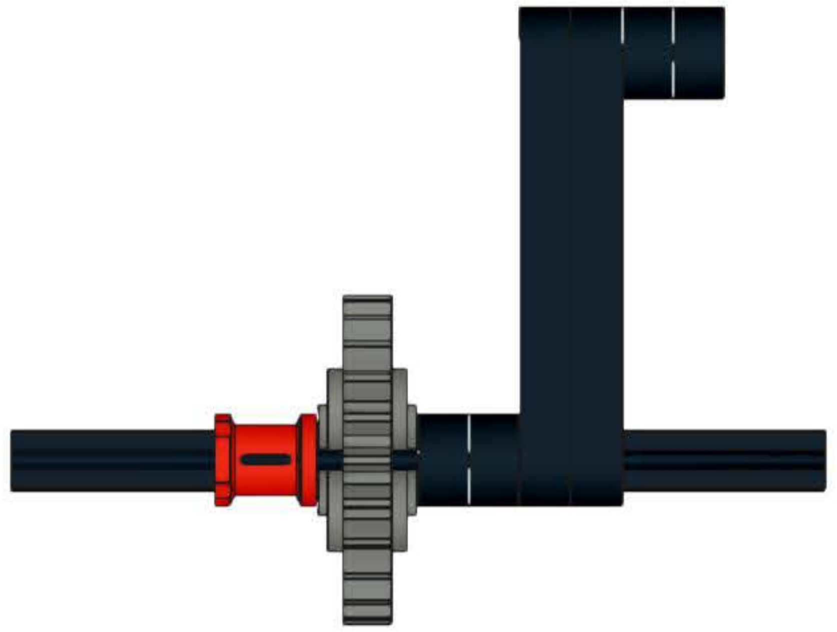

# 148

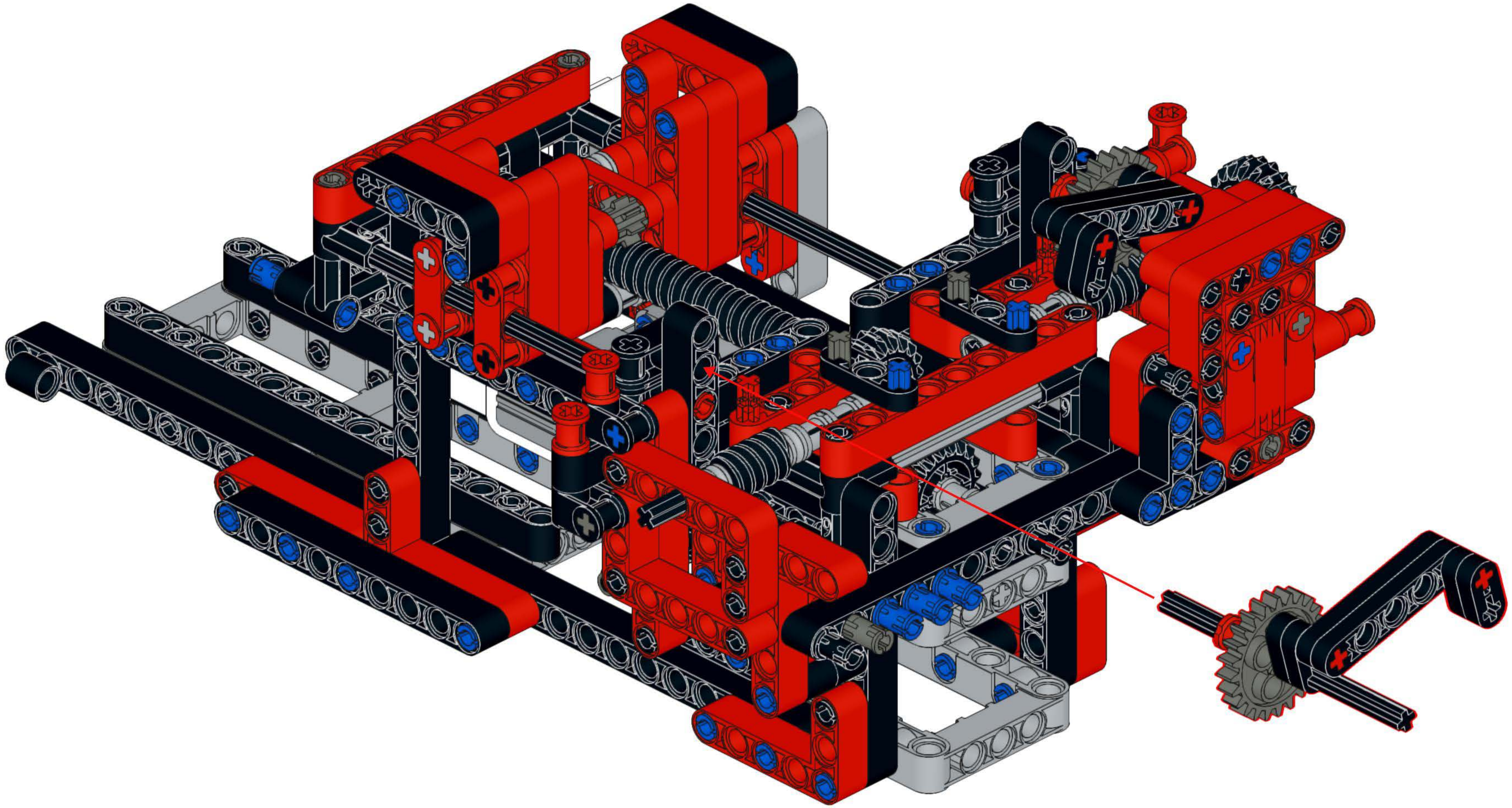

149

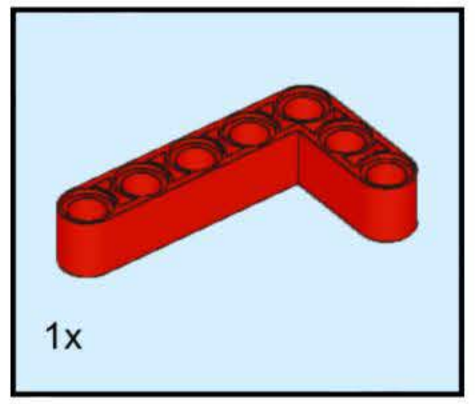


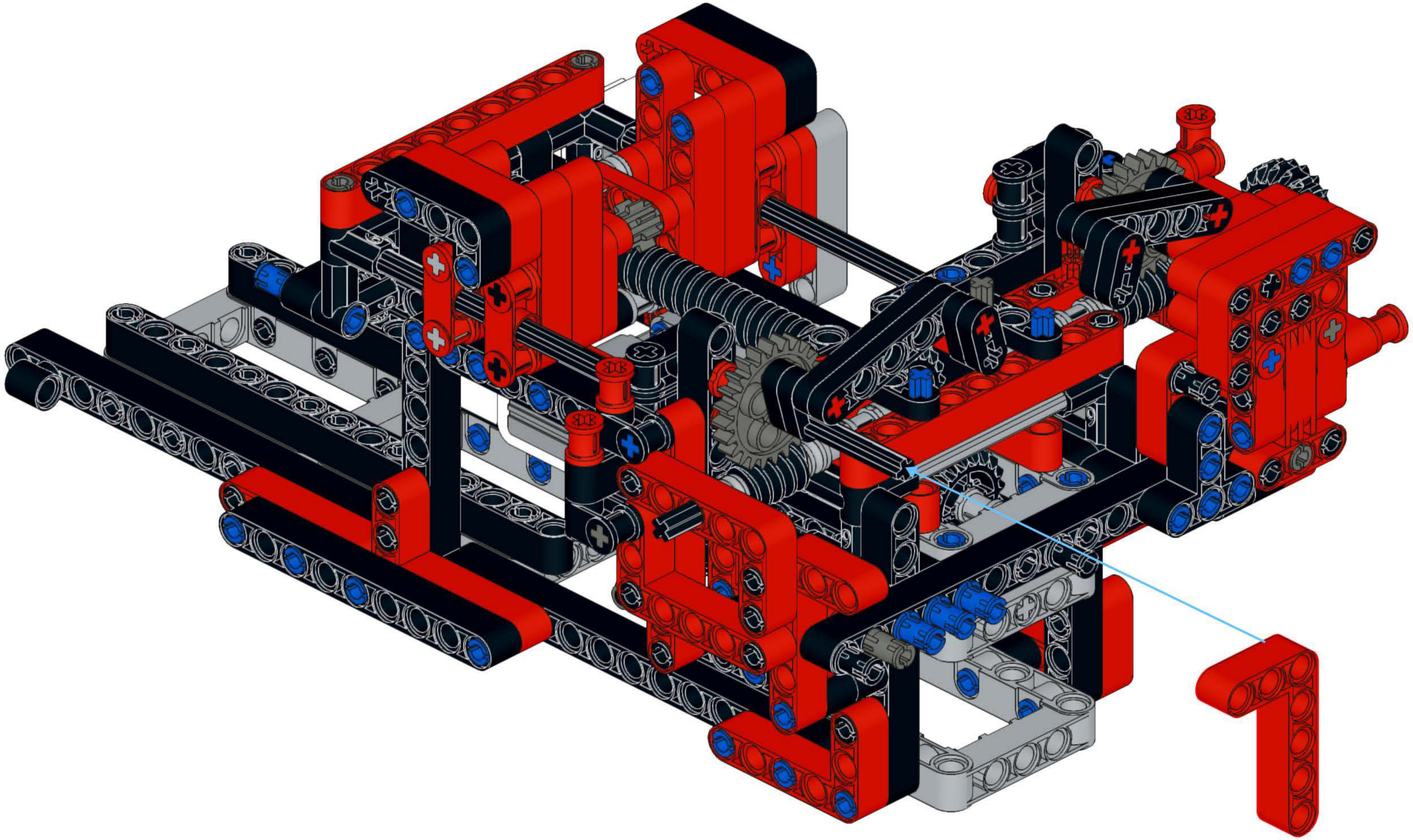

150

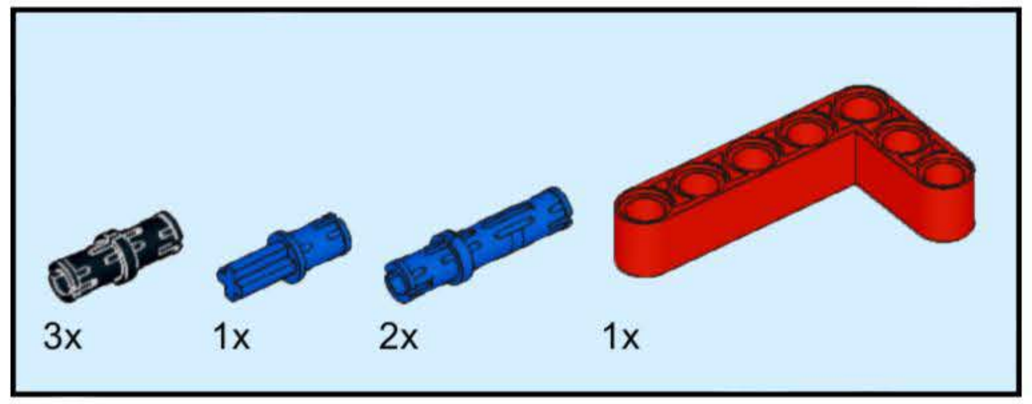


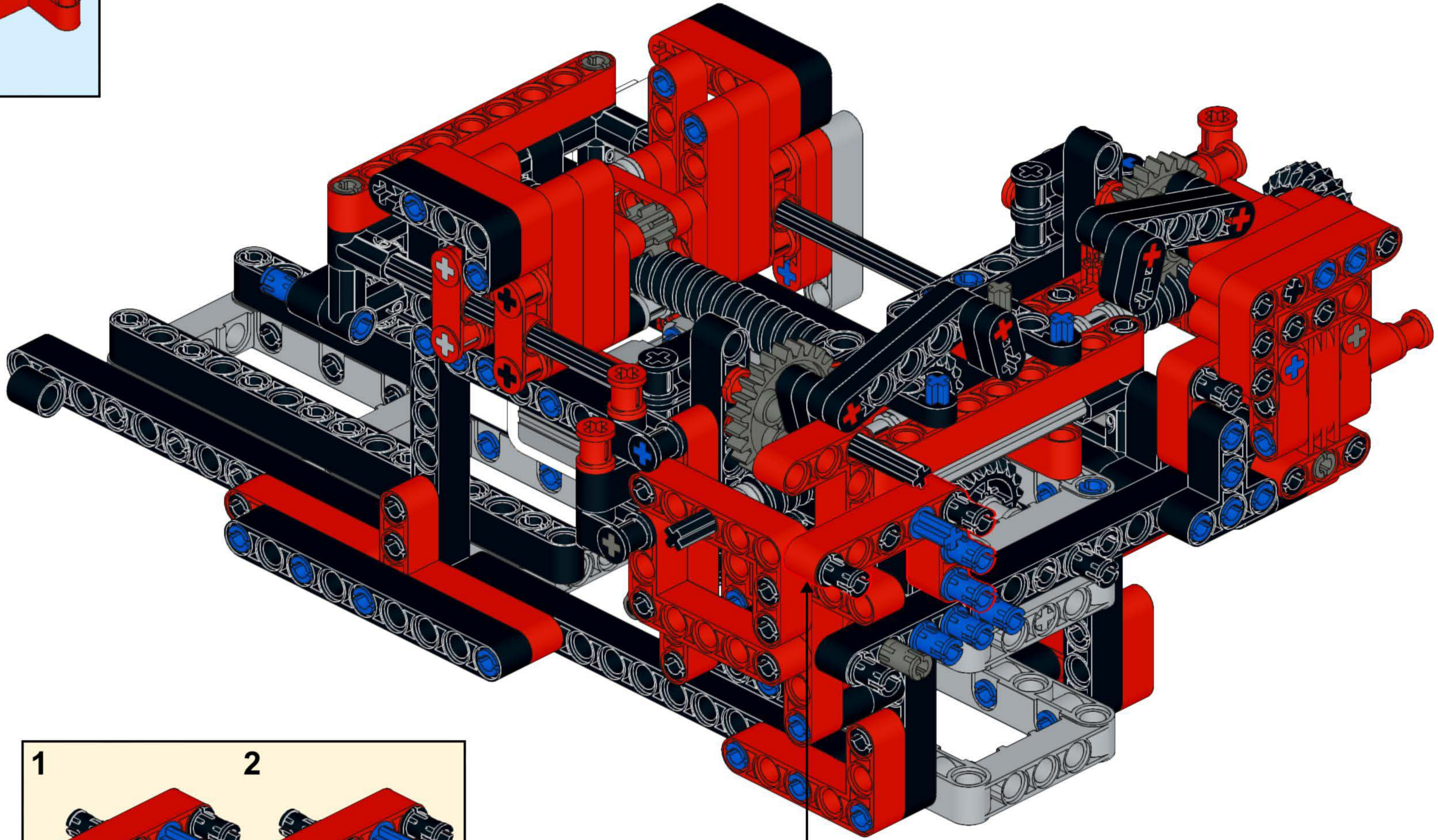

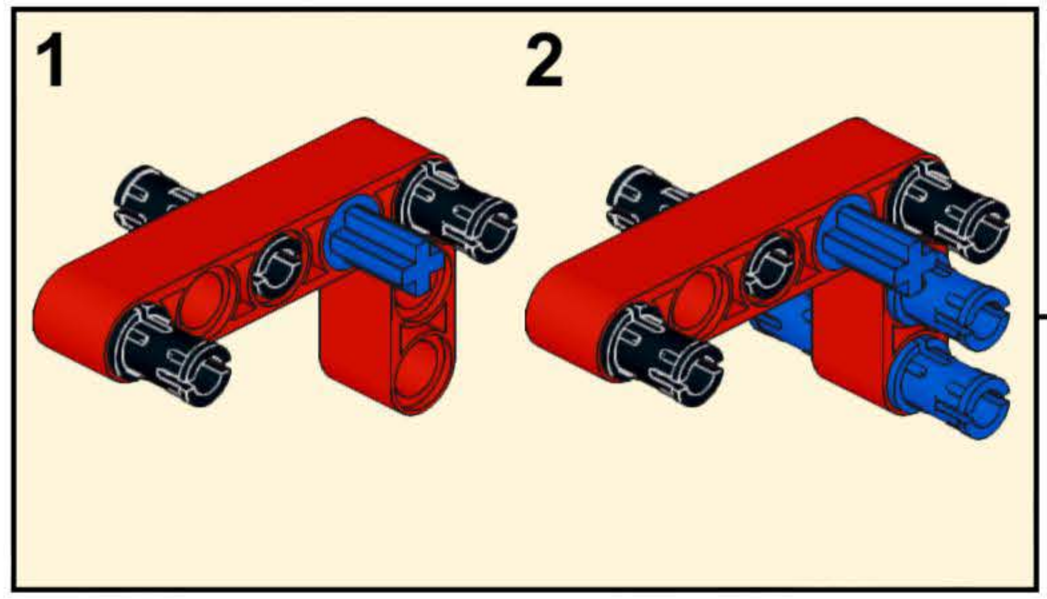

151

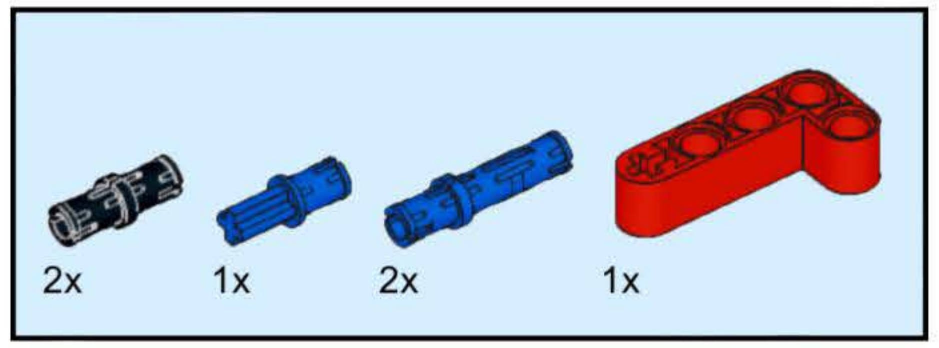


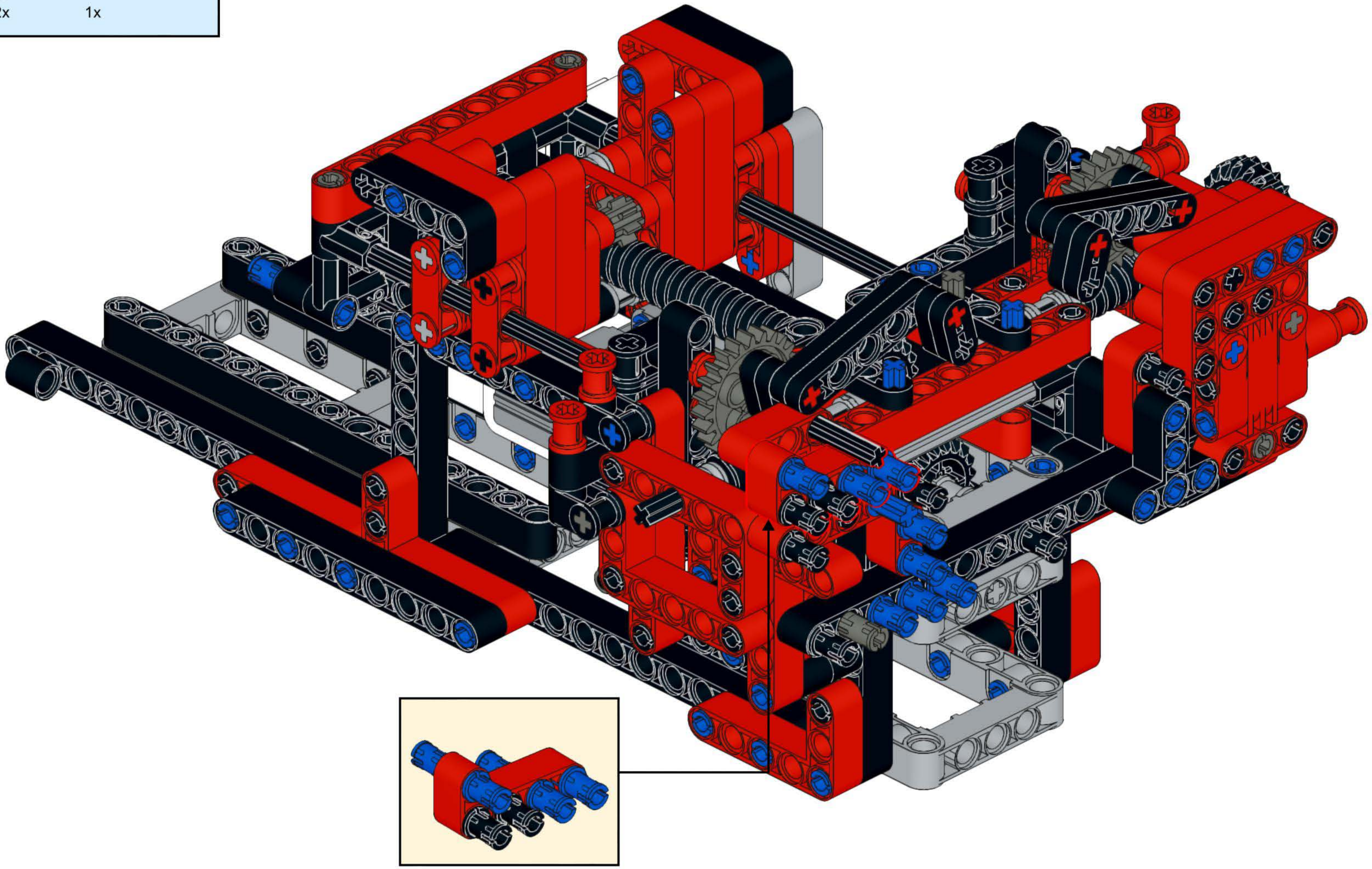

# 152

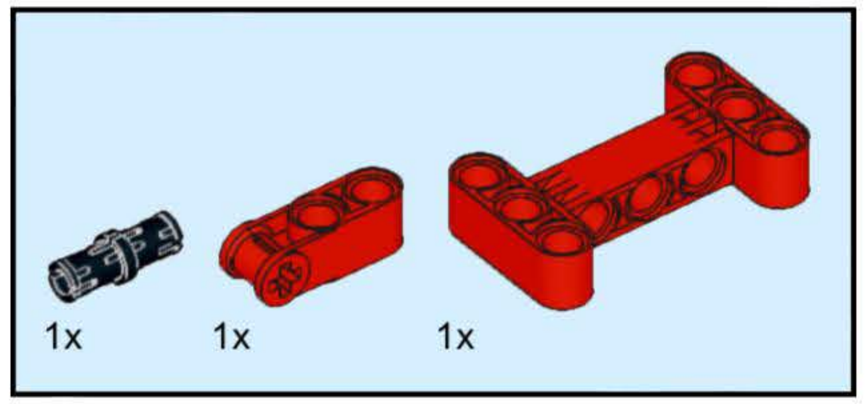


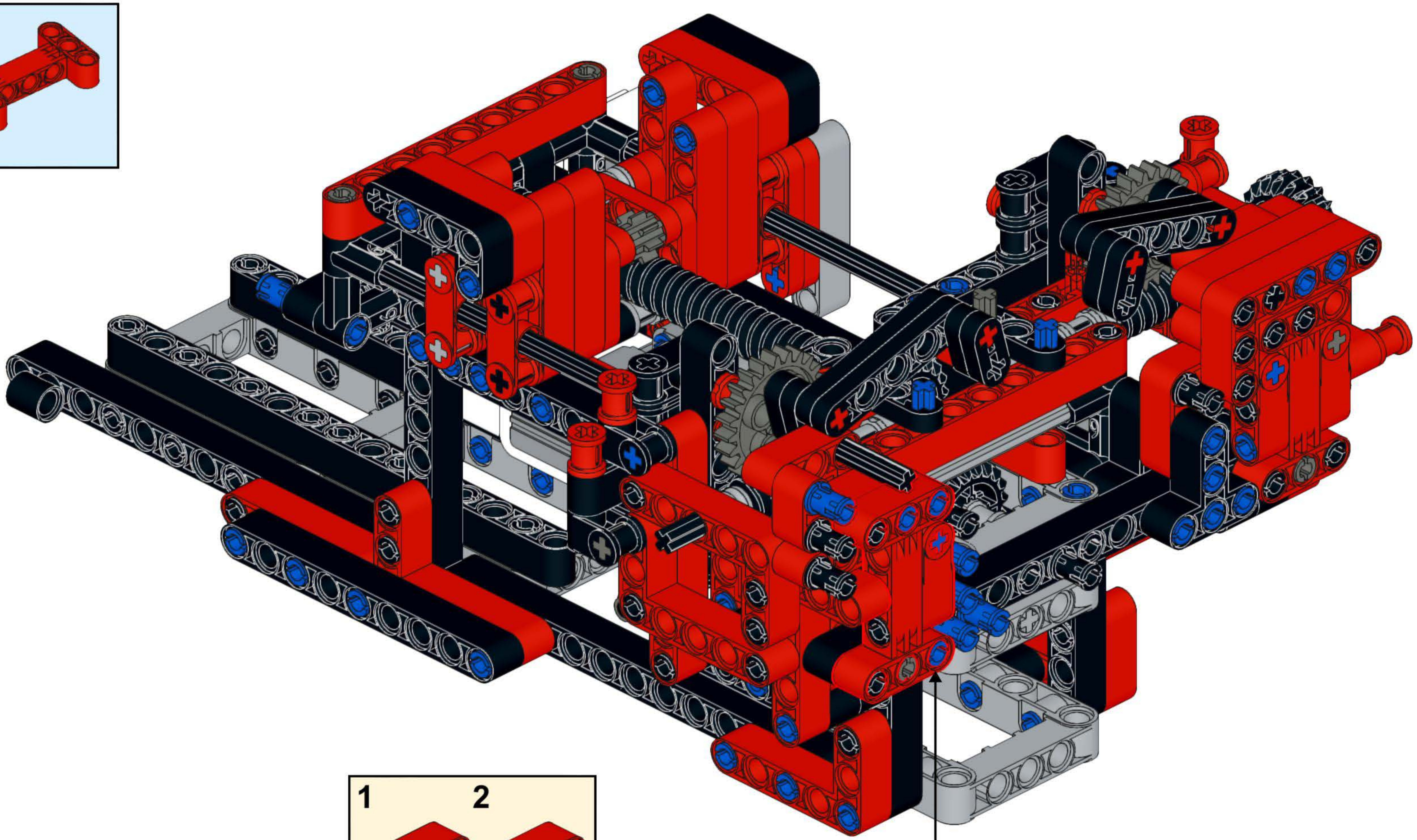

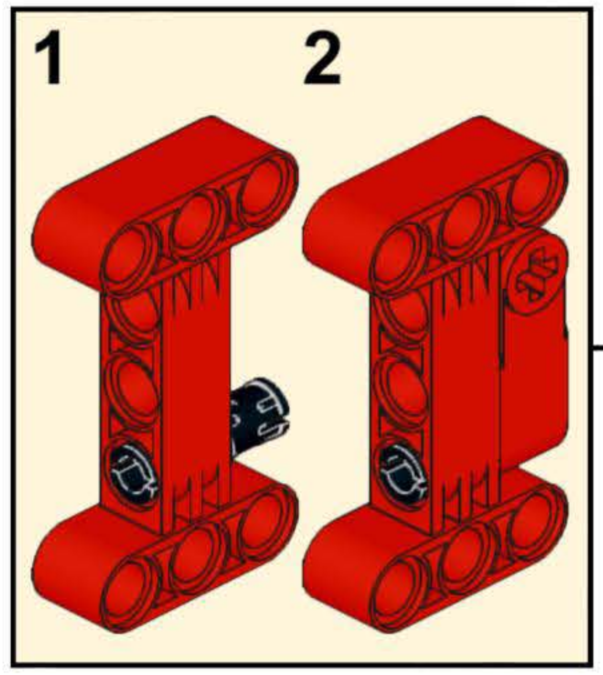

153

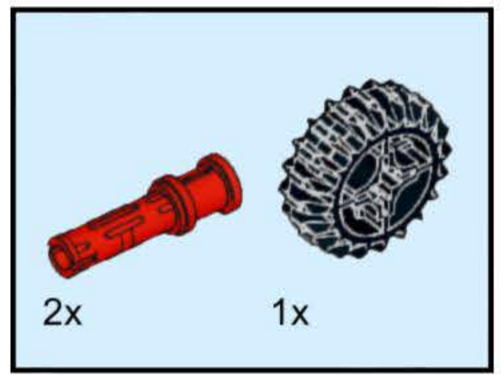


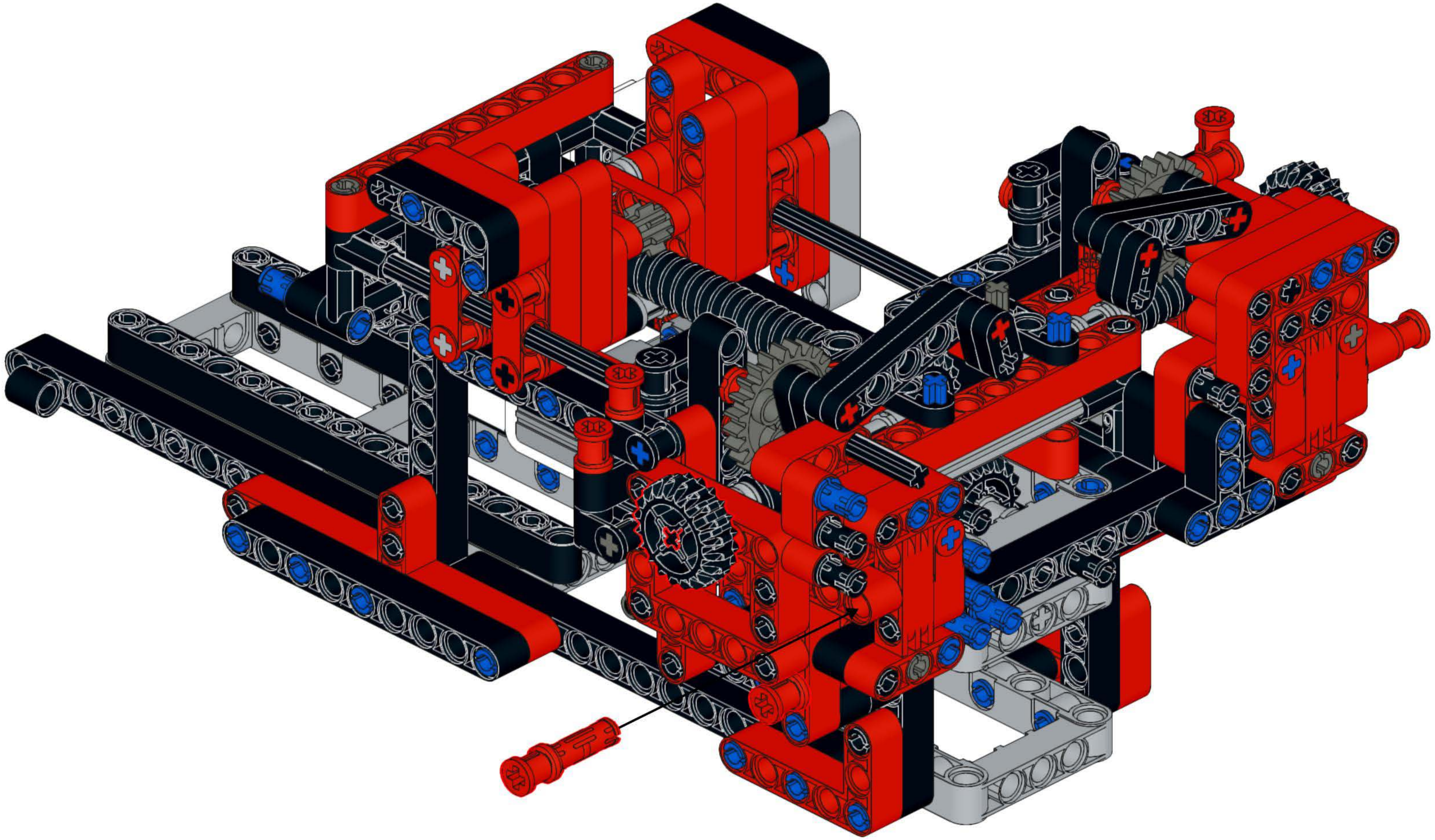

154

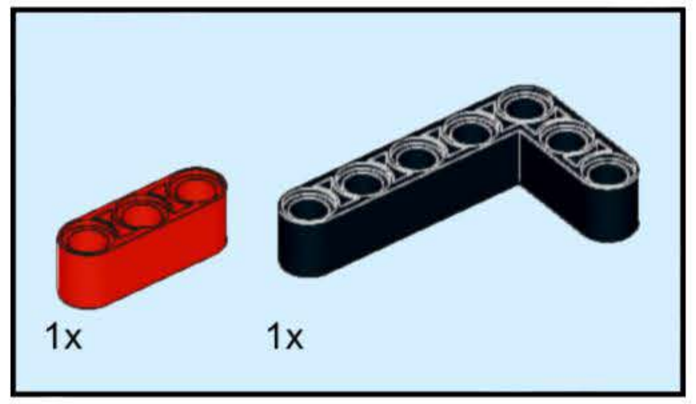


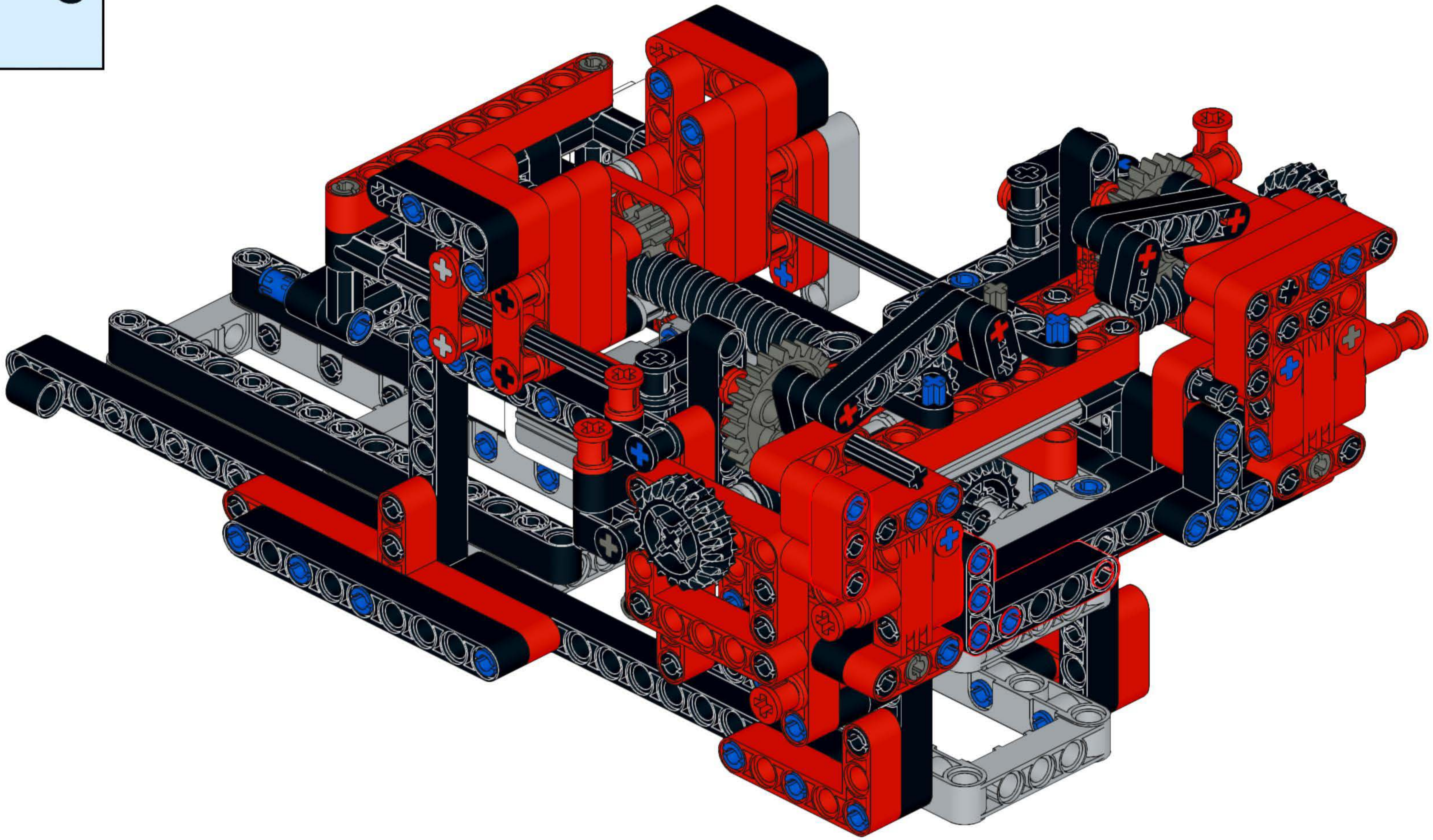

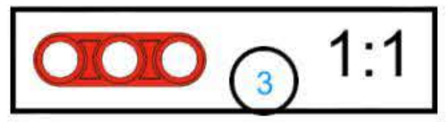

155

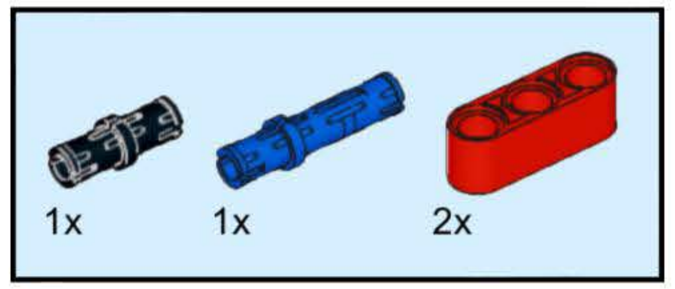


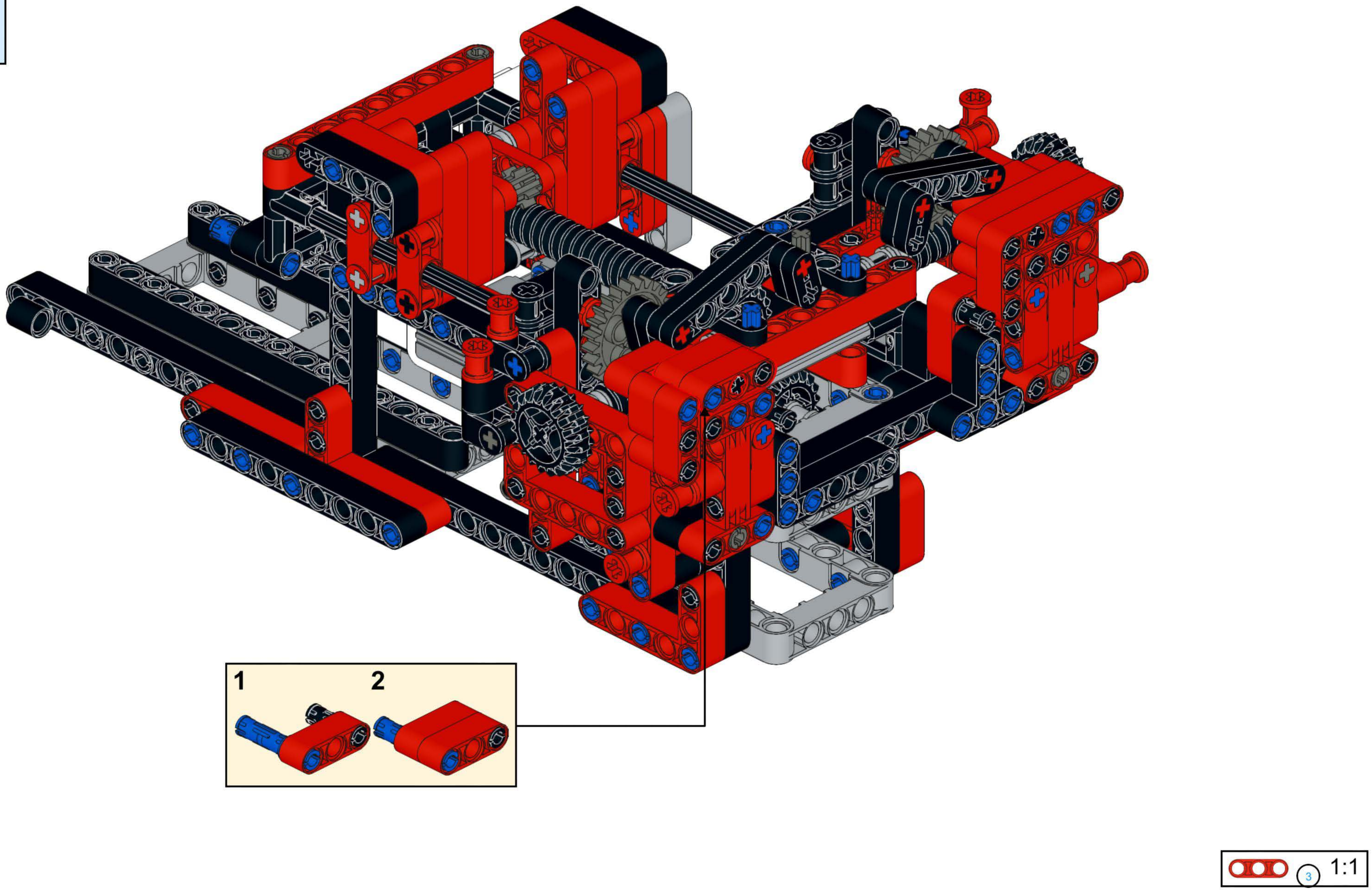

# 156

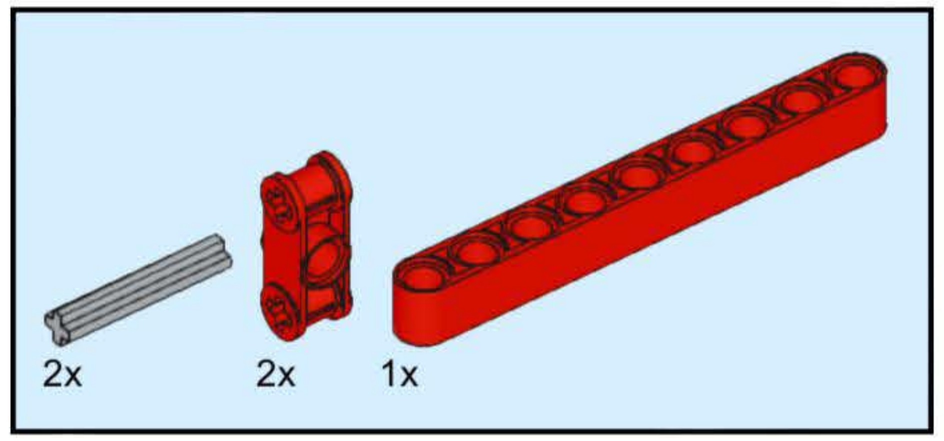


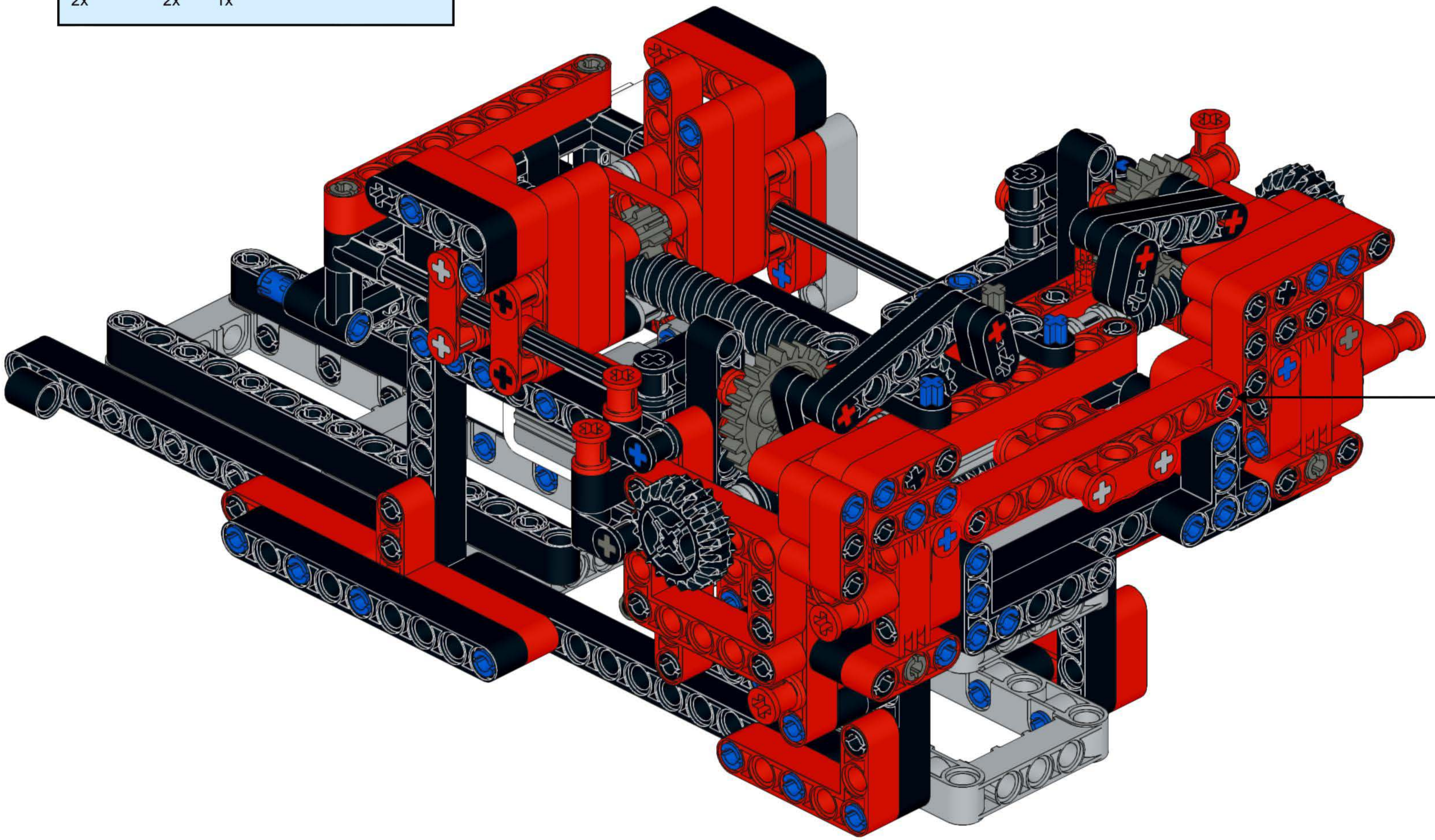

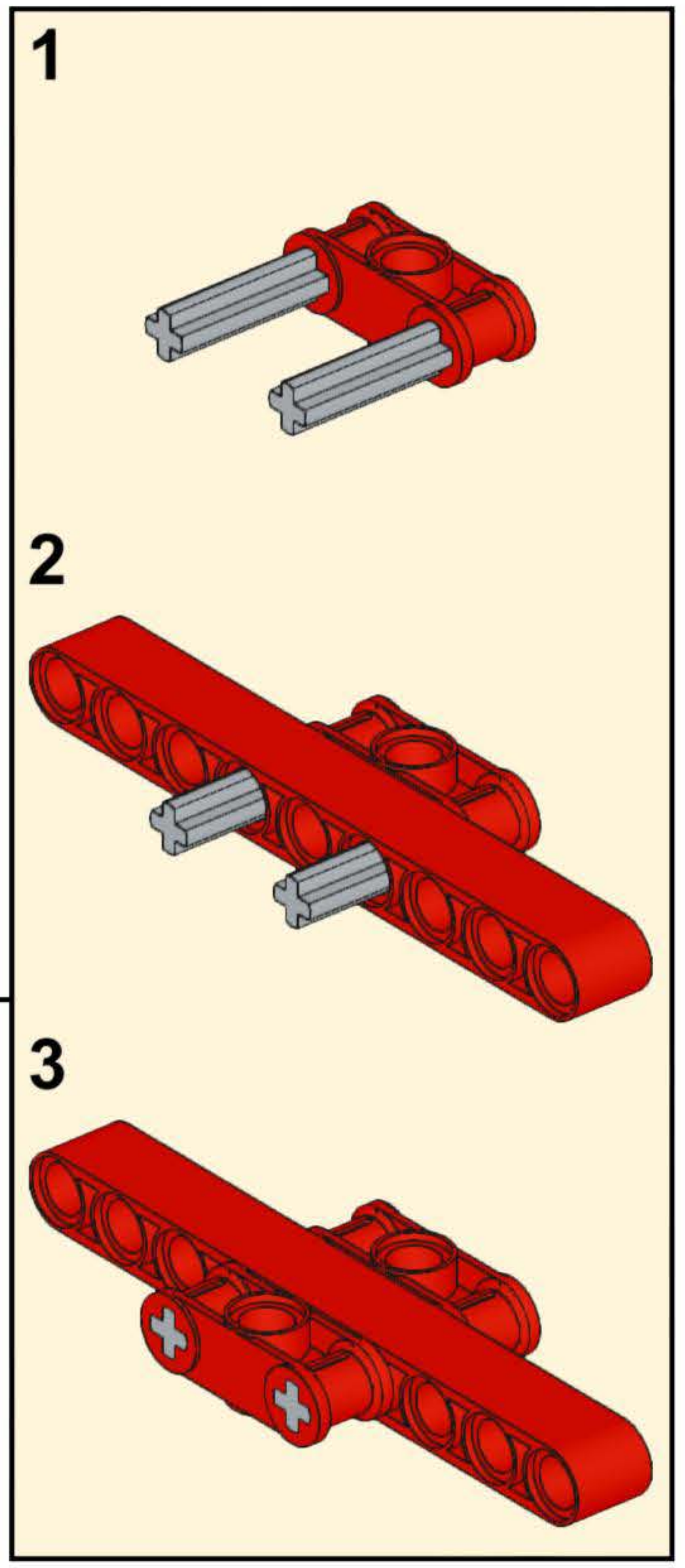


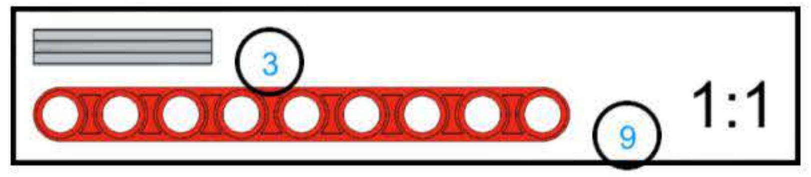

157

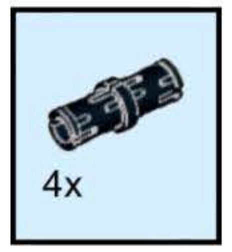


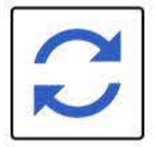

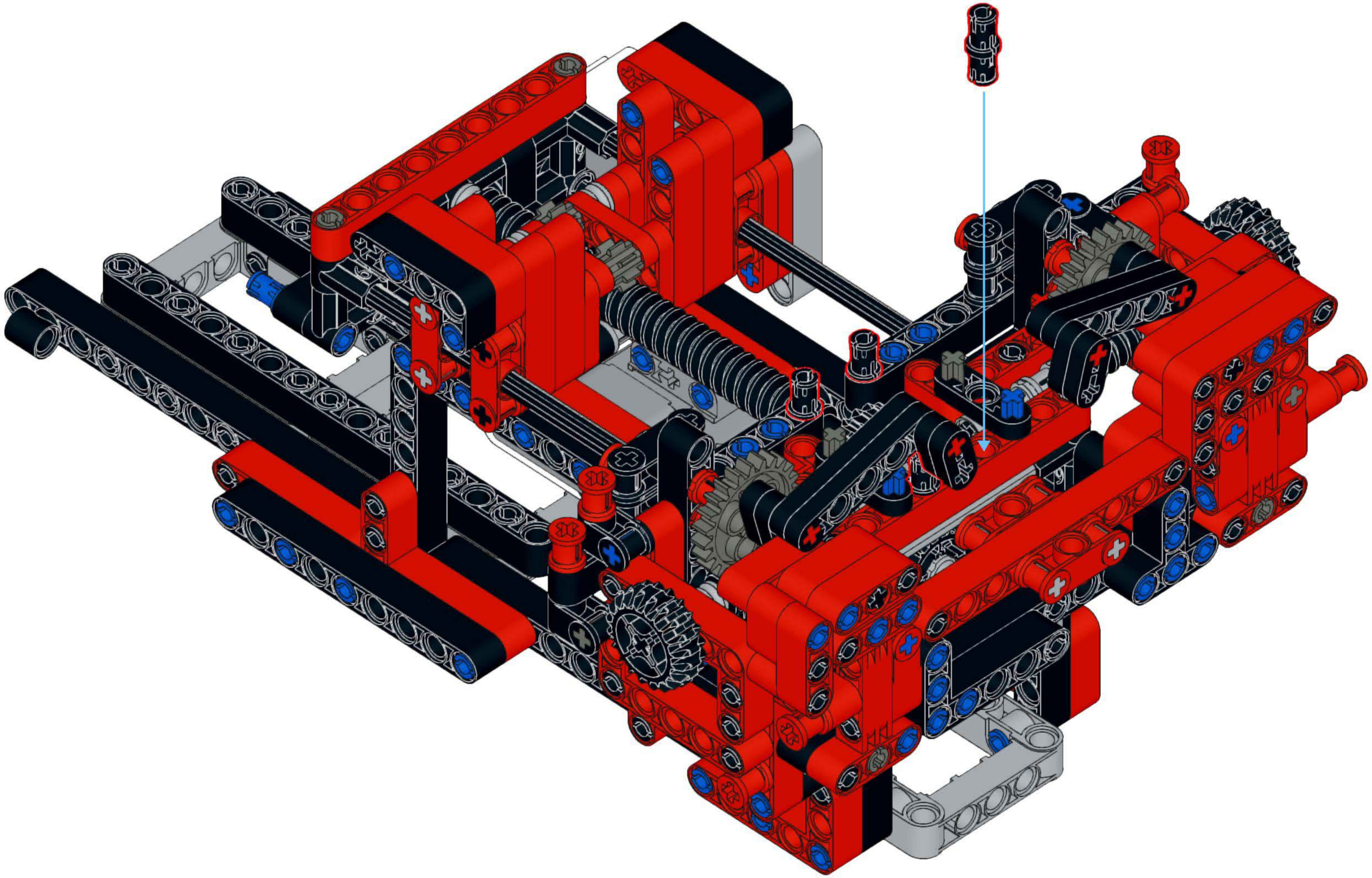

158

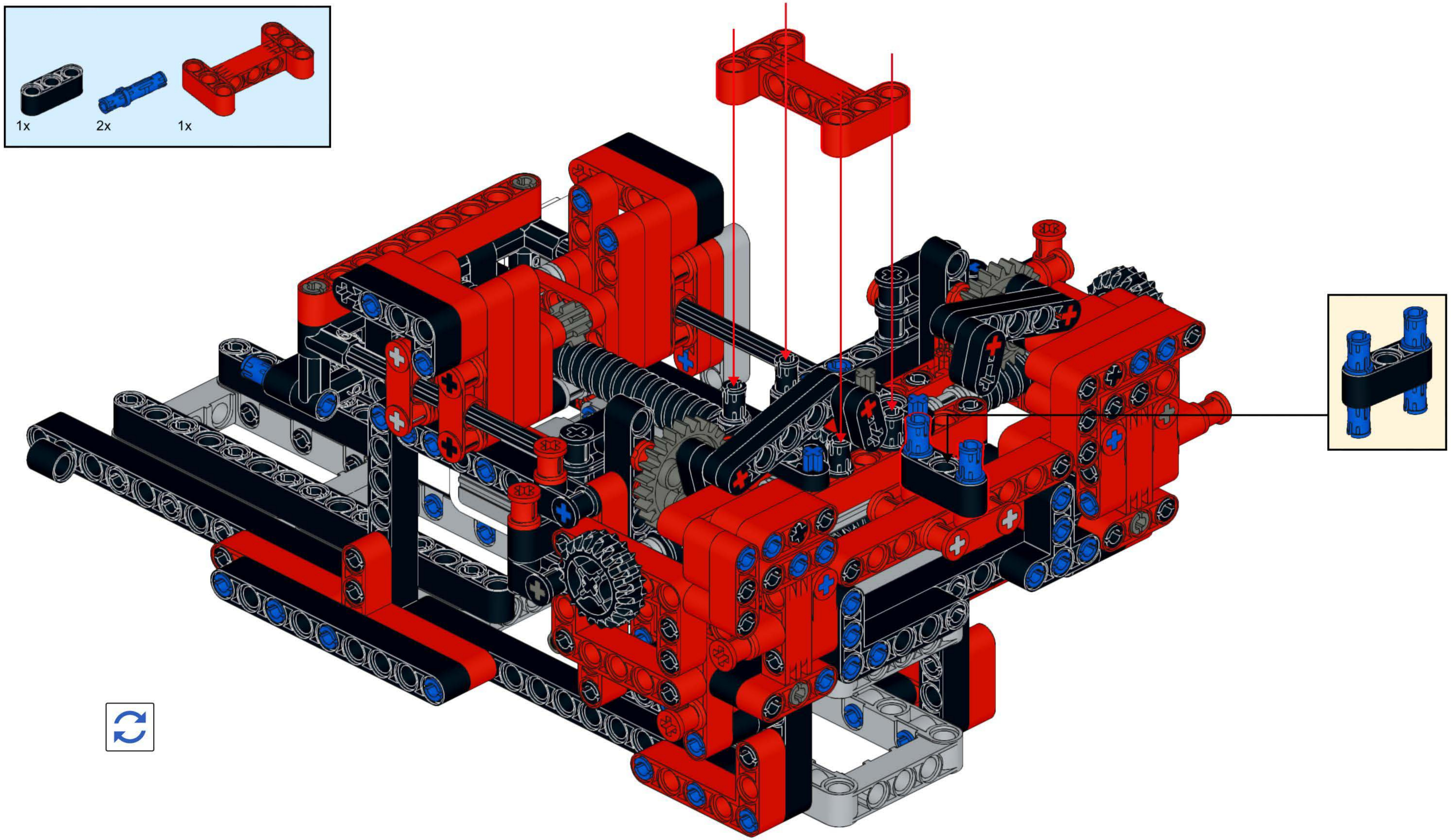


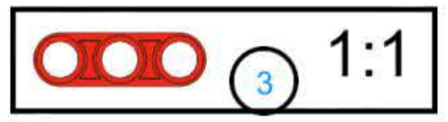

# 159

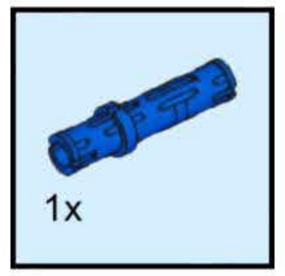
1x

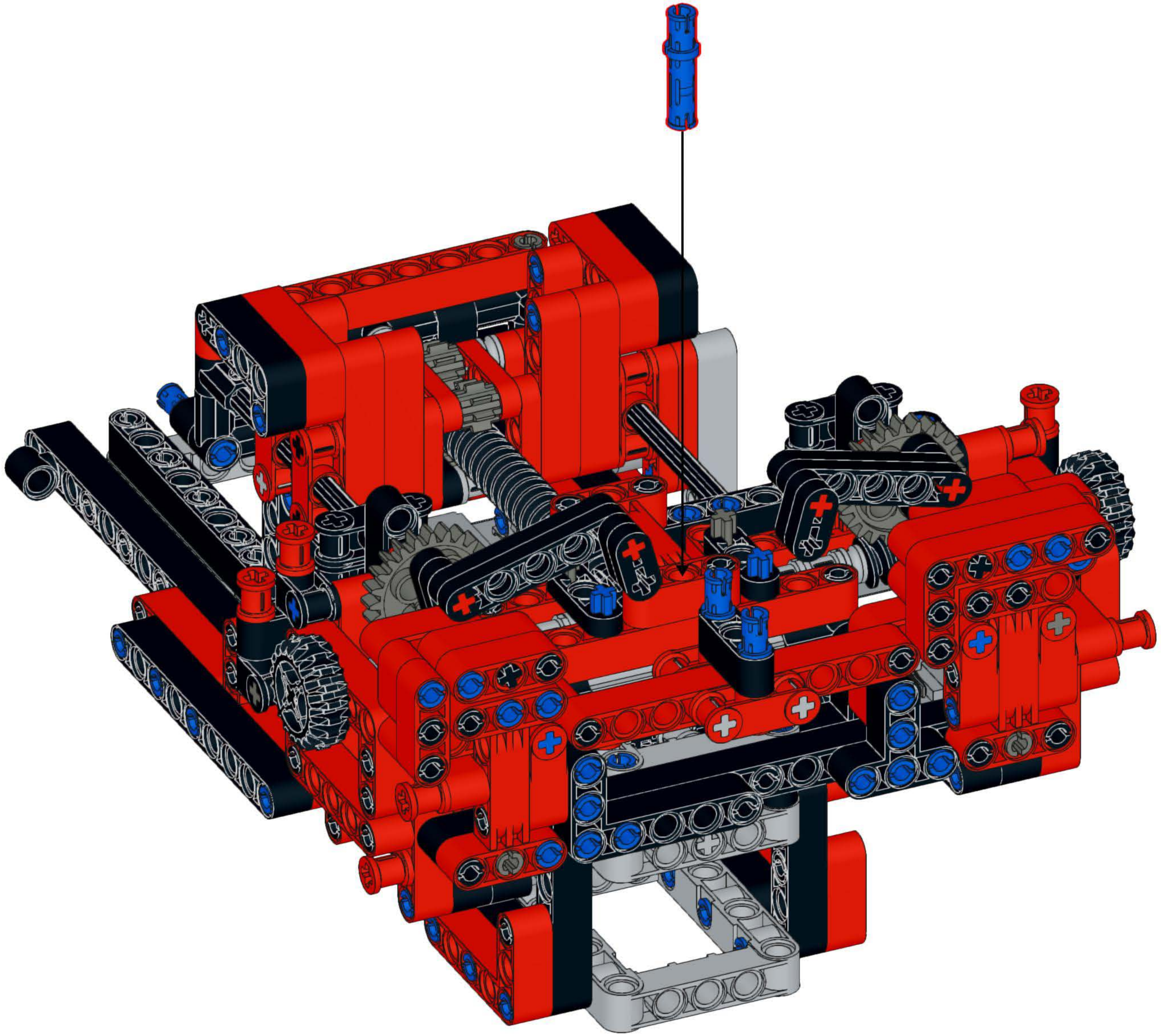

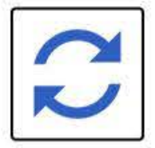

160

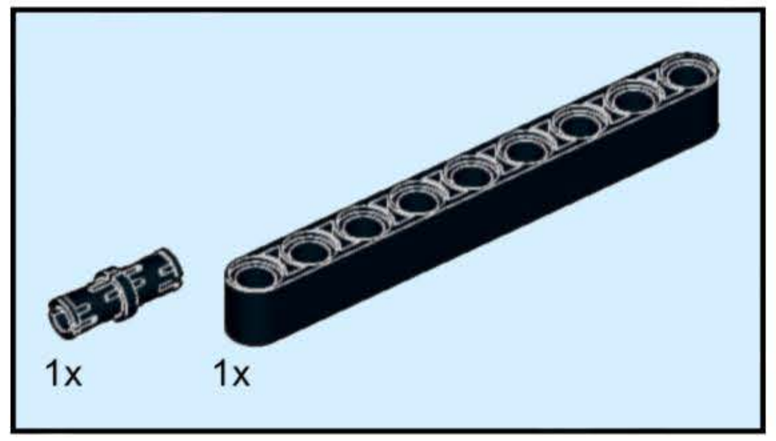


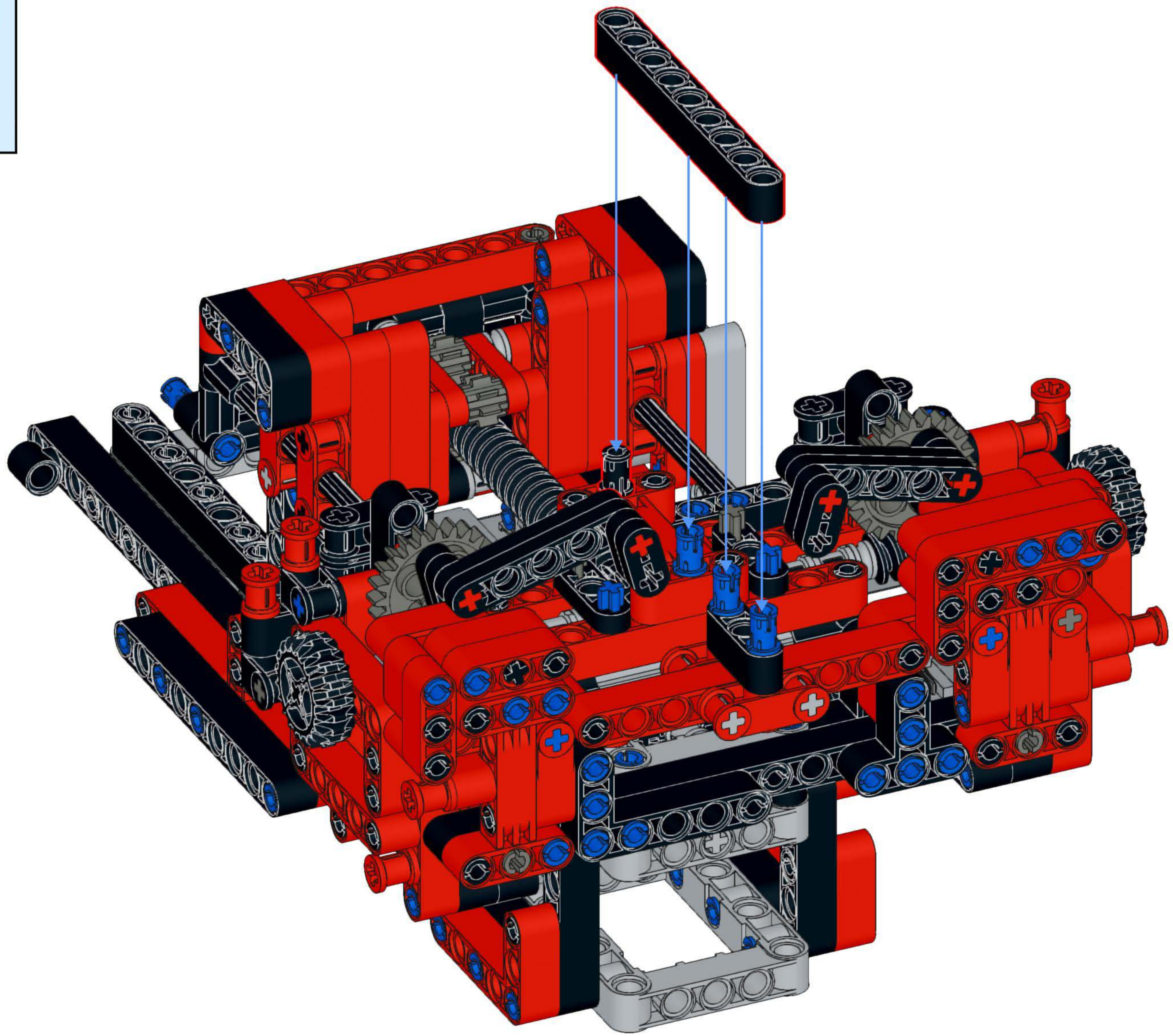

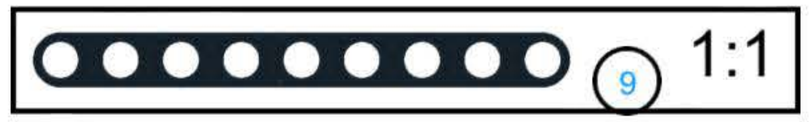

# 161

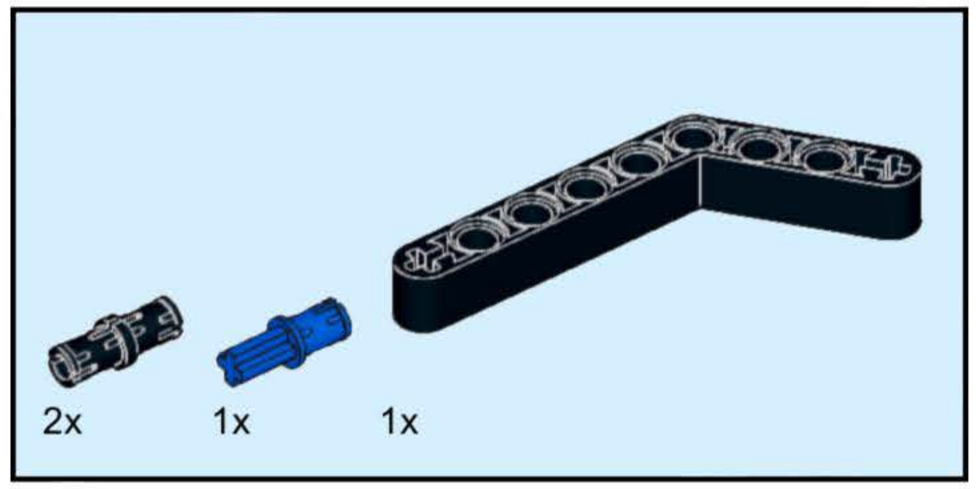


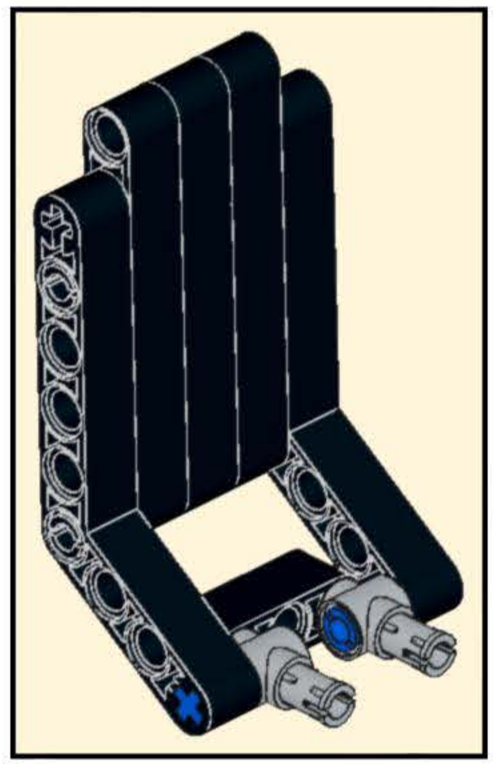

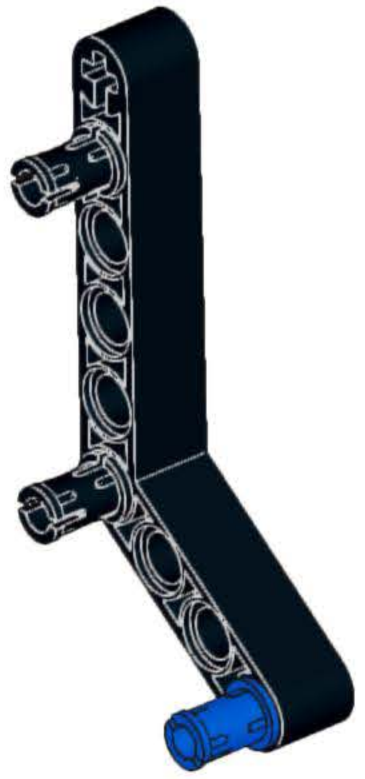

# 162

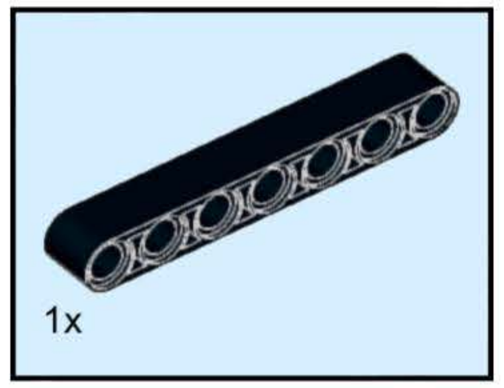


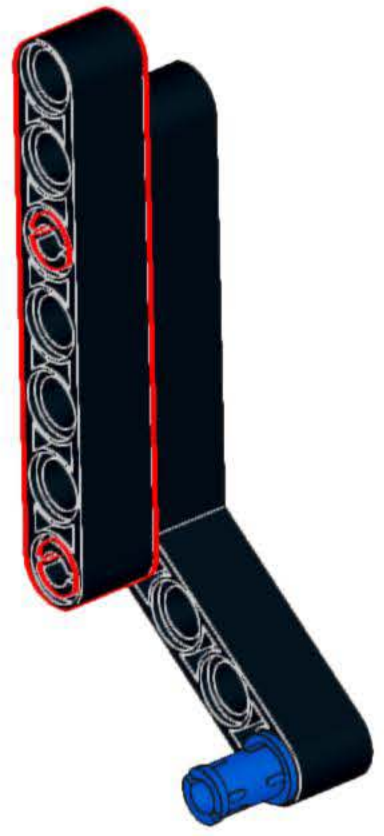

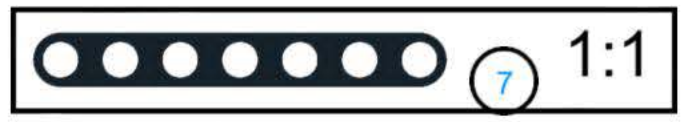


# 163

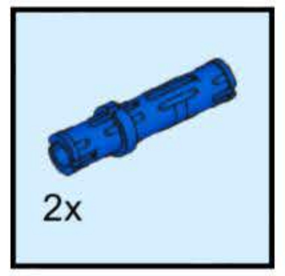


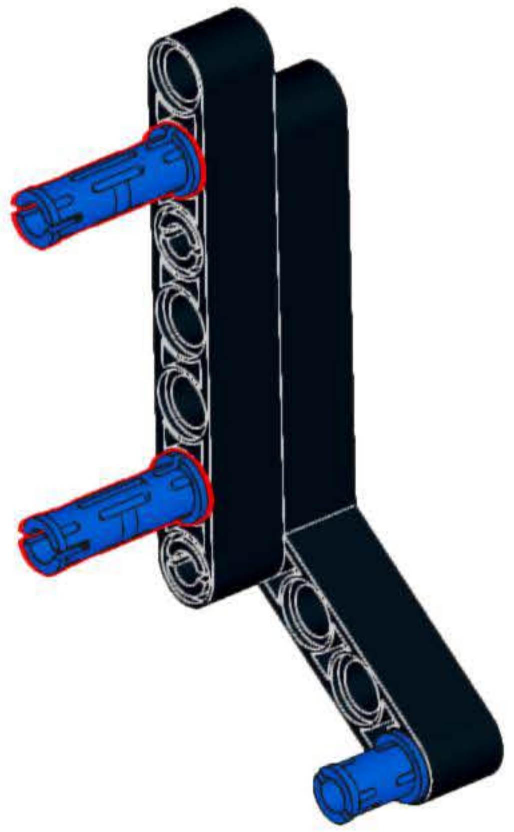

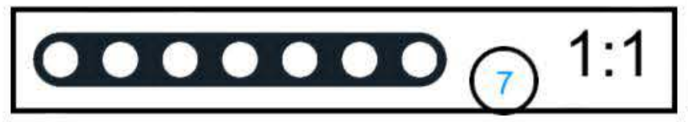


# 164

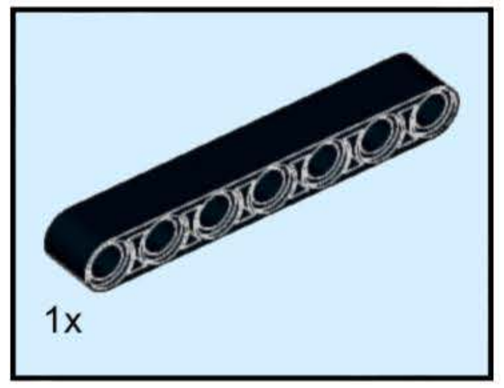


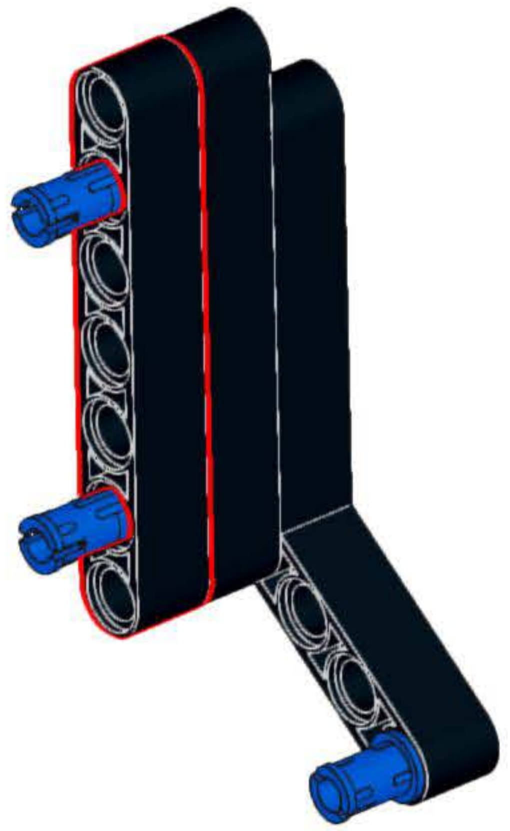

**165**

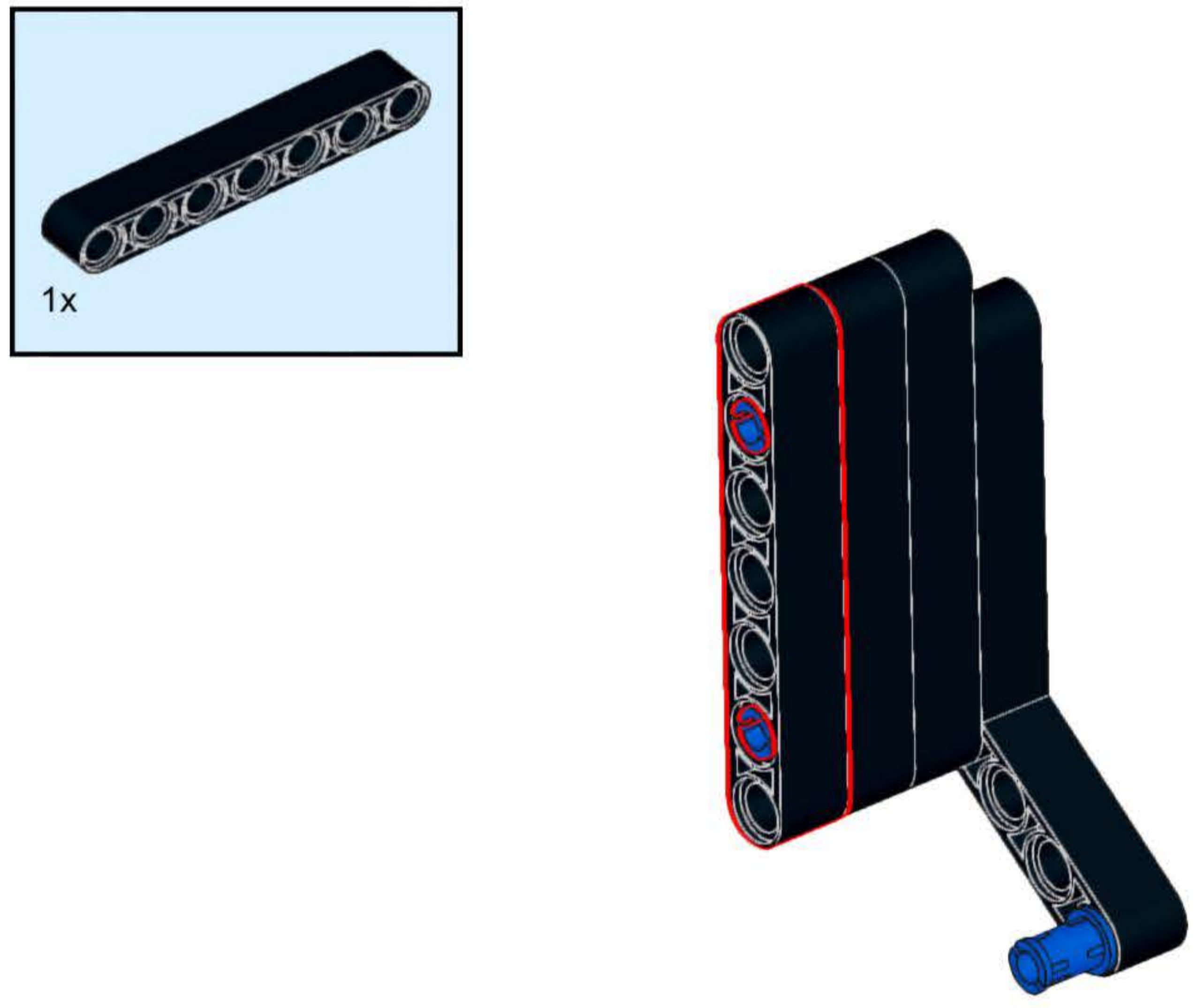


**166**

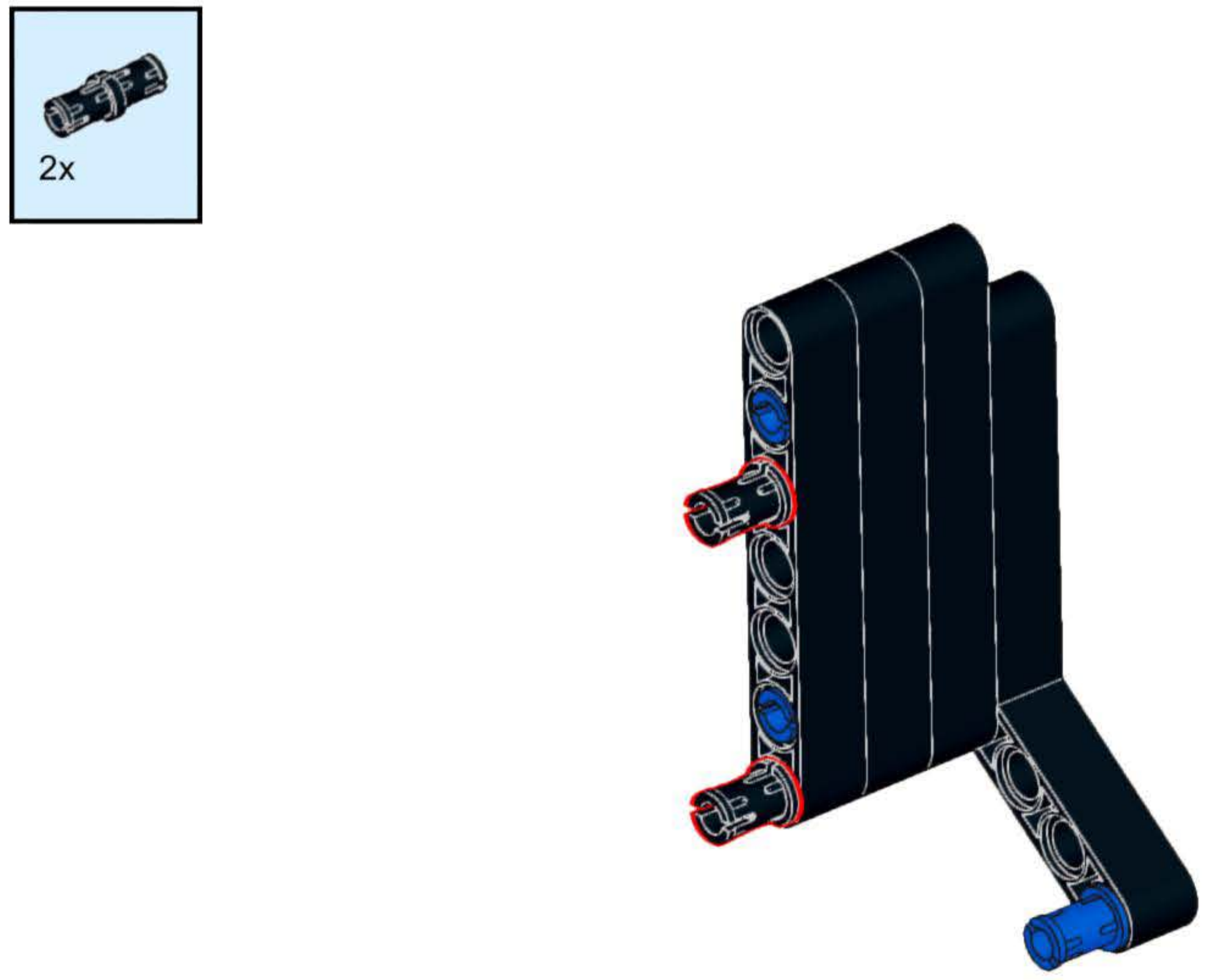


**167**

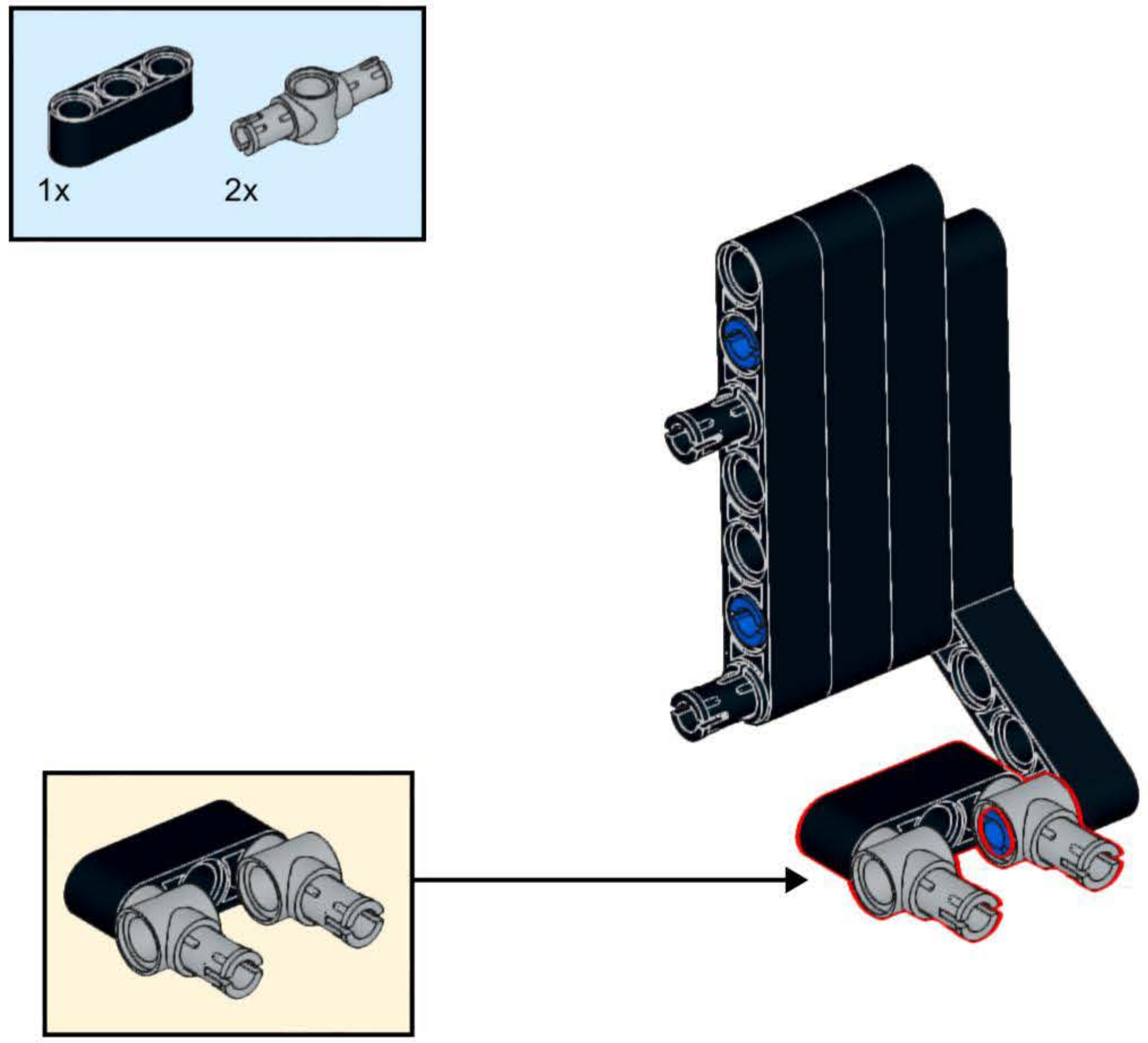


**168**

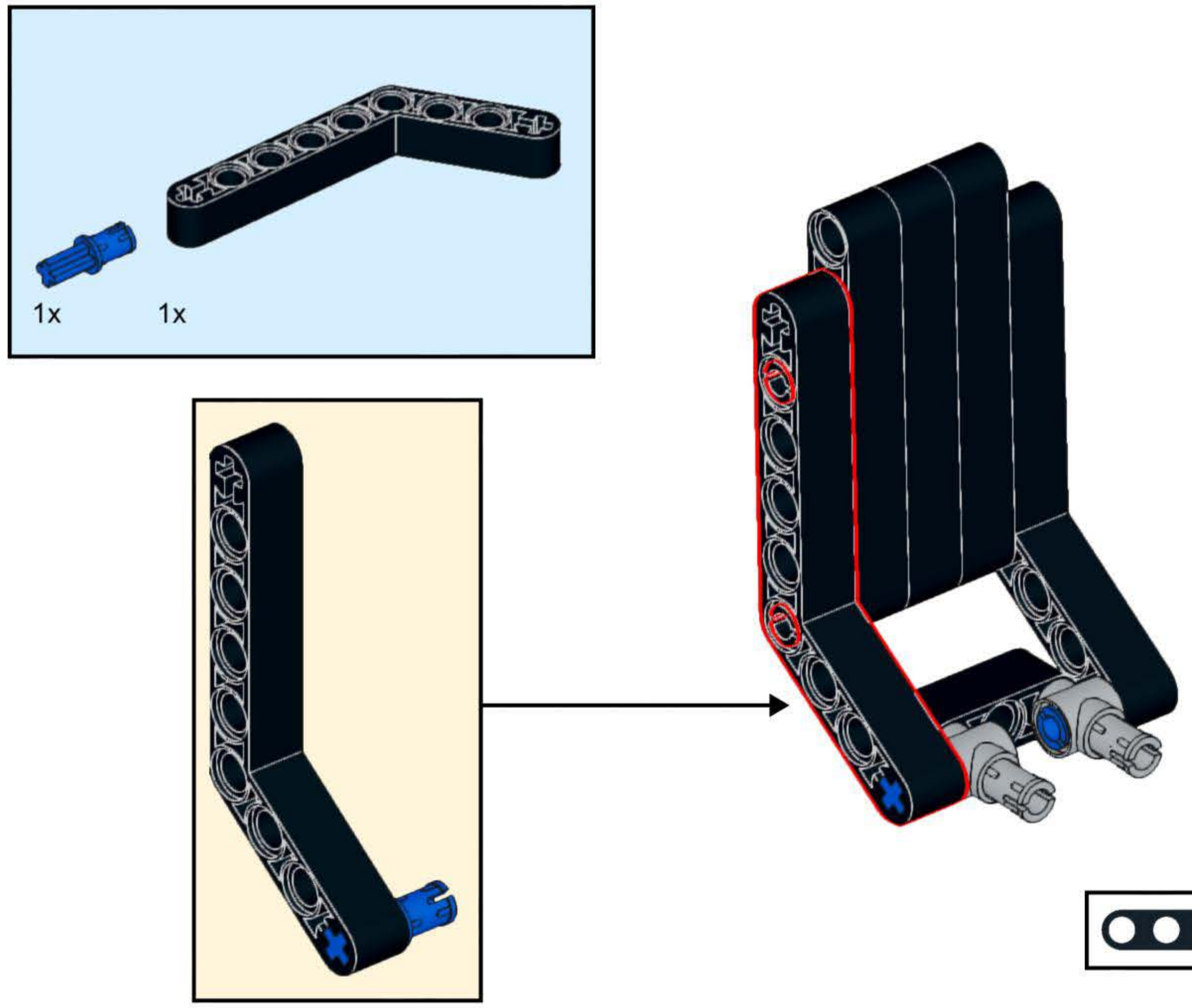


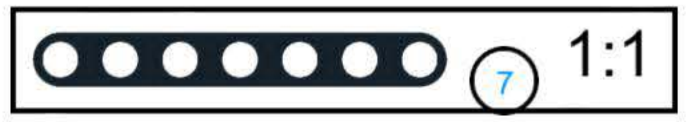

169

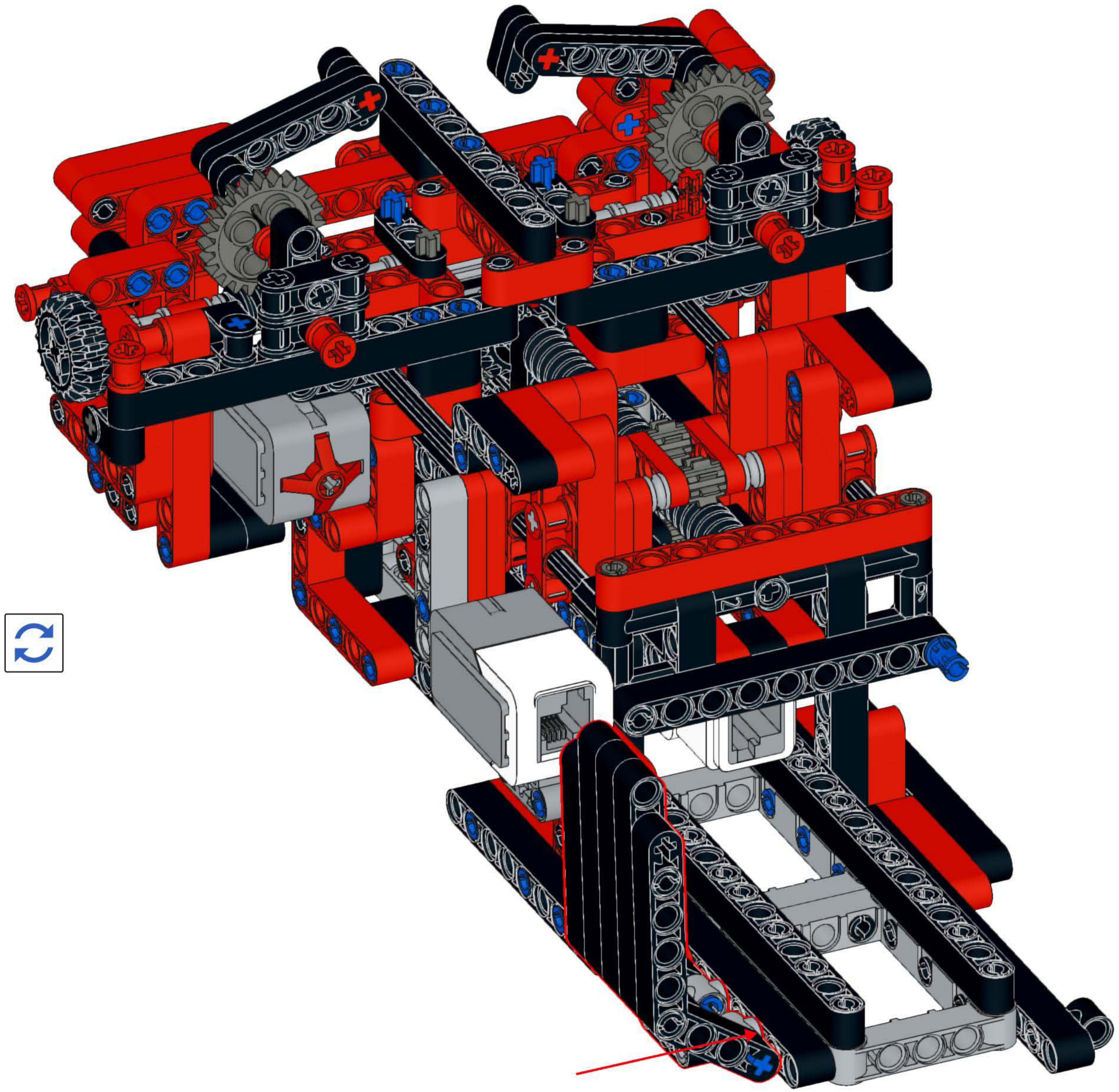

# 170

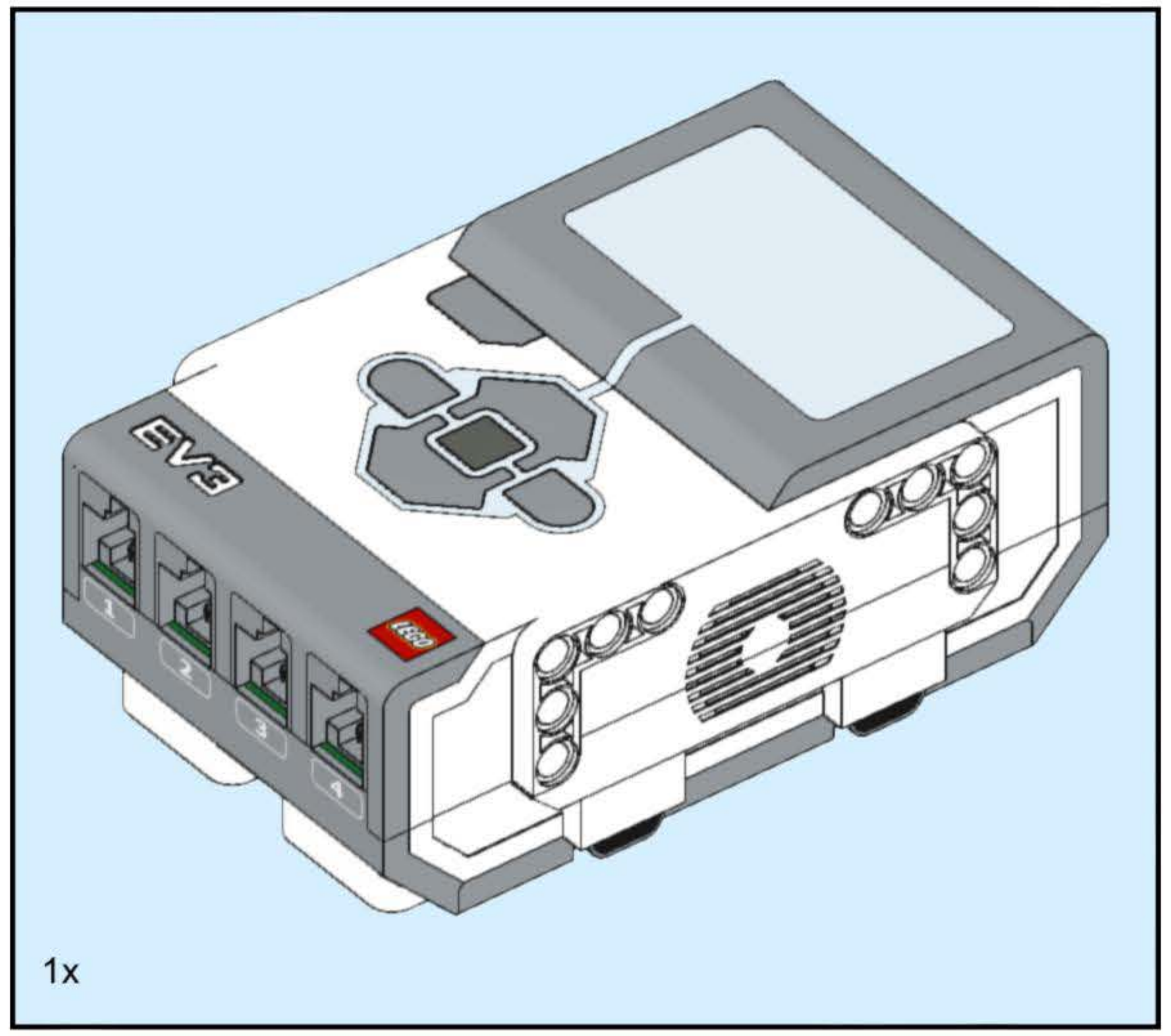


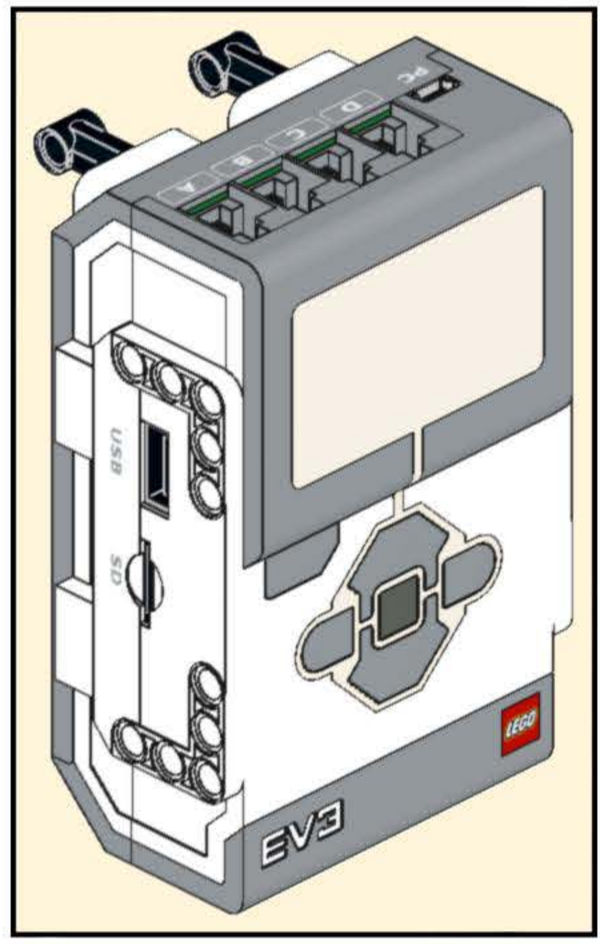

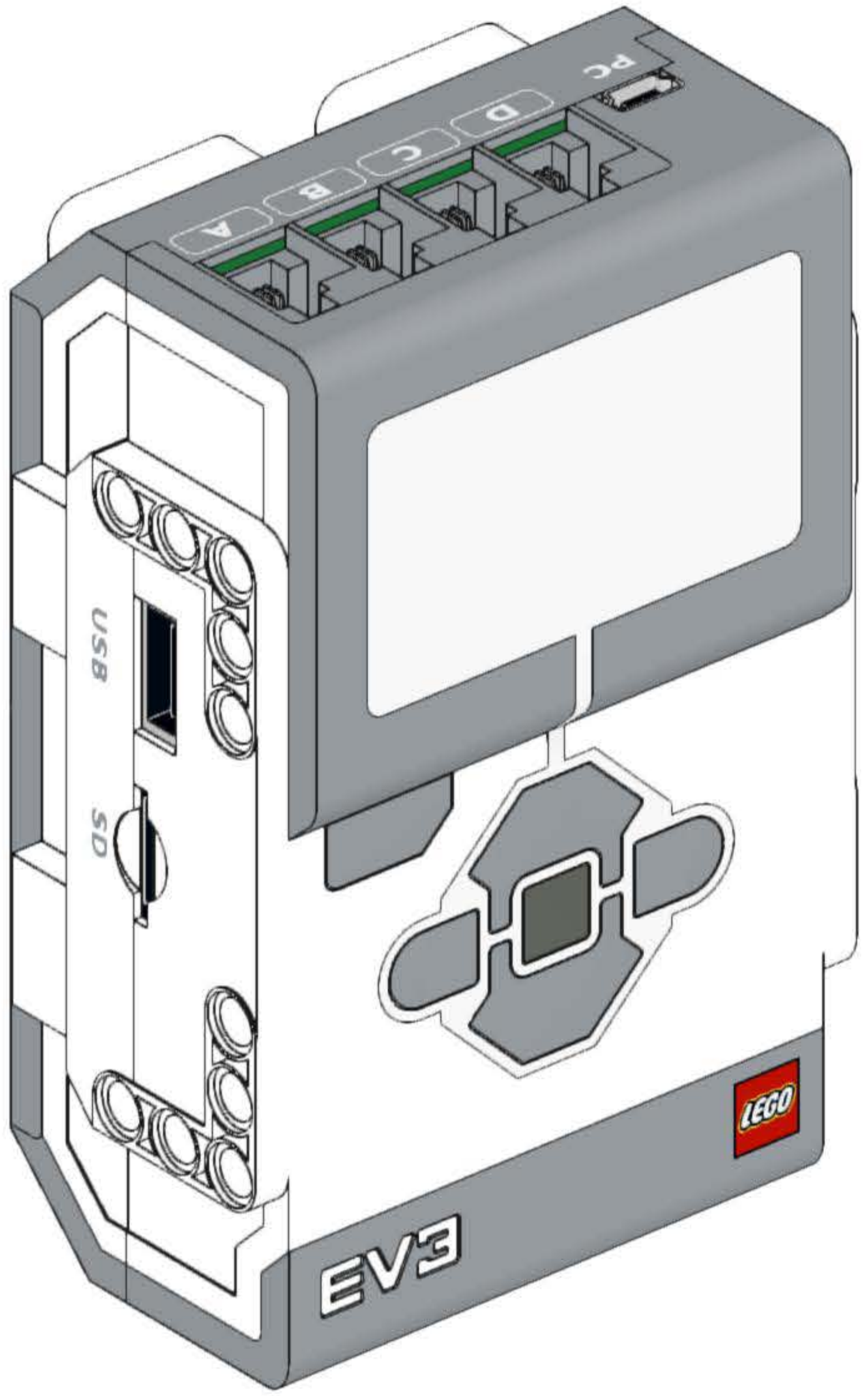

# 171

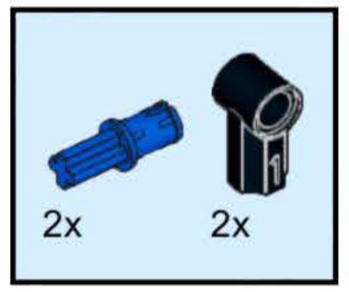


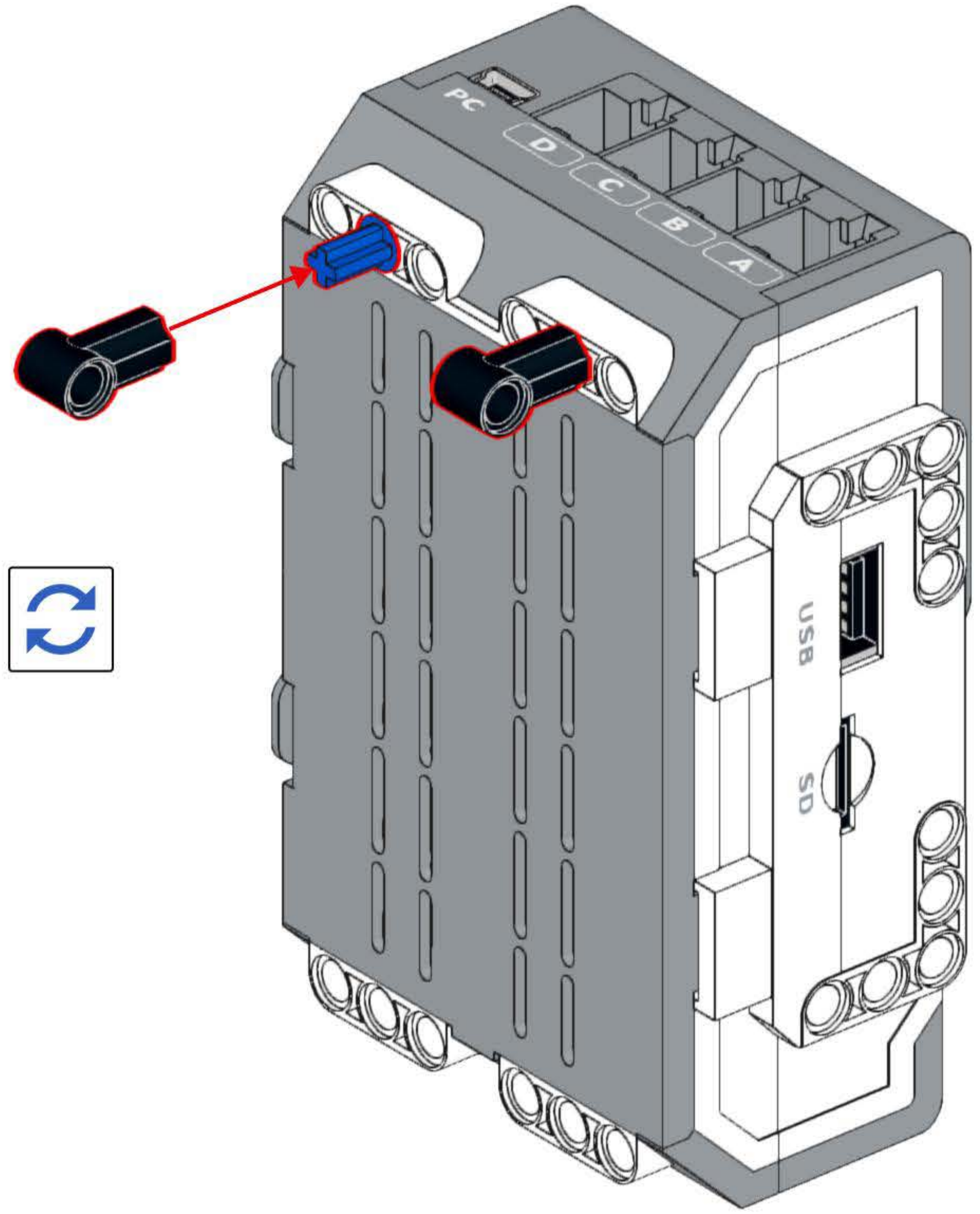

172

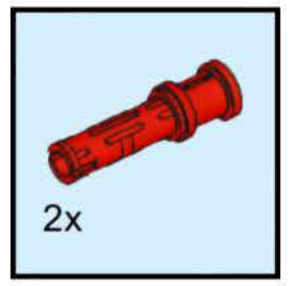


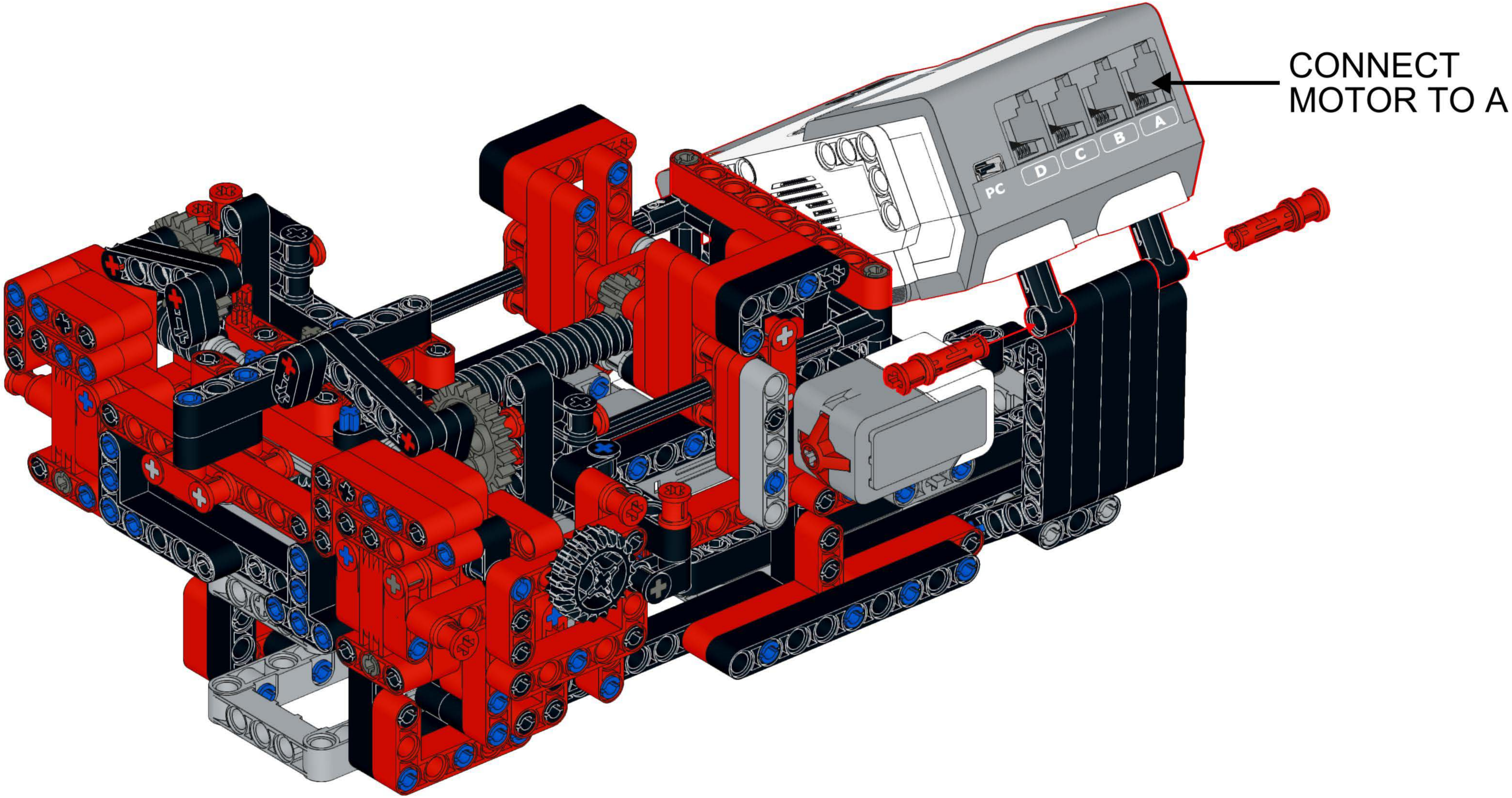

173

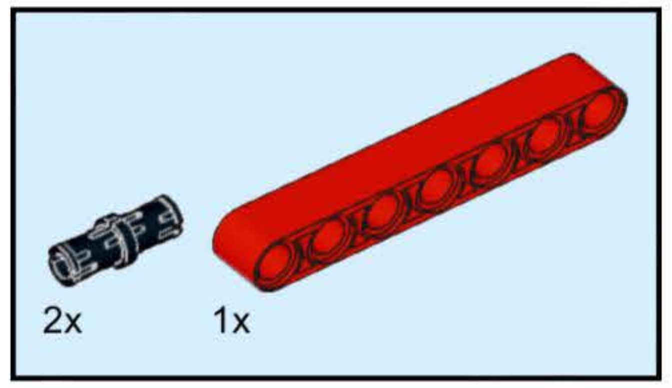


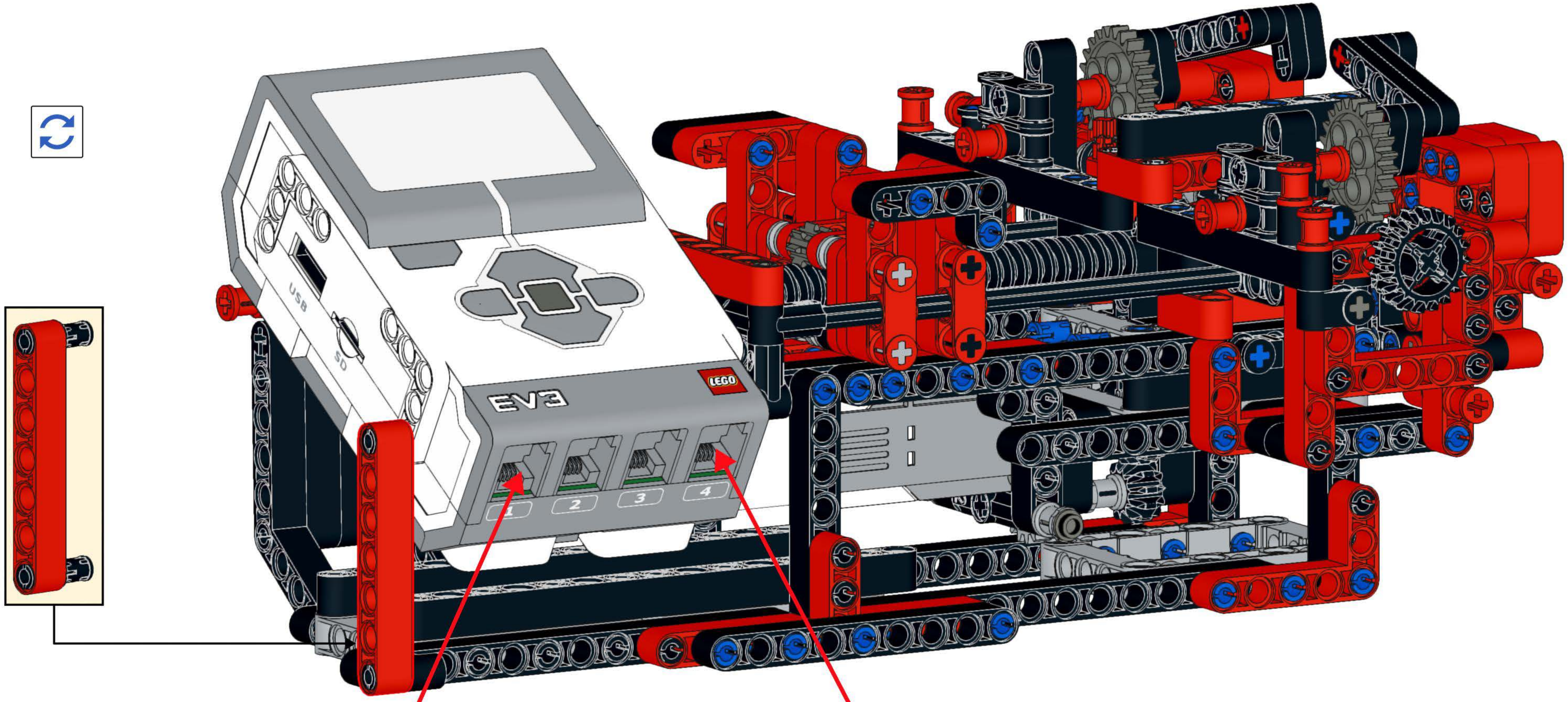


CONNECT LIMIT
SWITCH CLOSEST
TO EV3 BRICK TO 1

CONNECT LIMIT
SWITCH FURTHEST
FROM EV3 BRICK TO 4

174

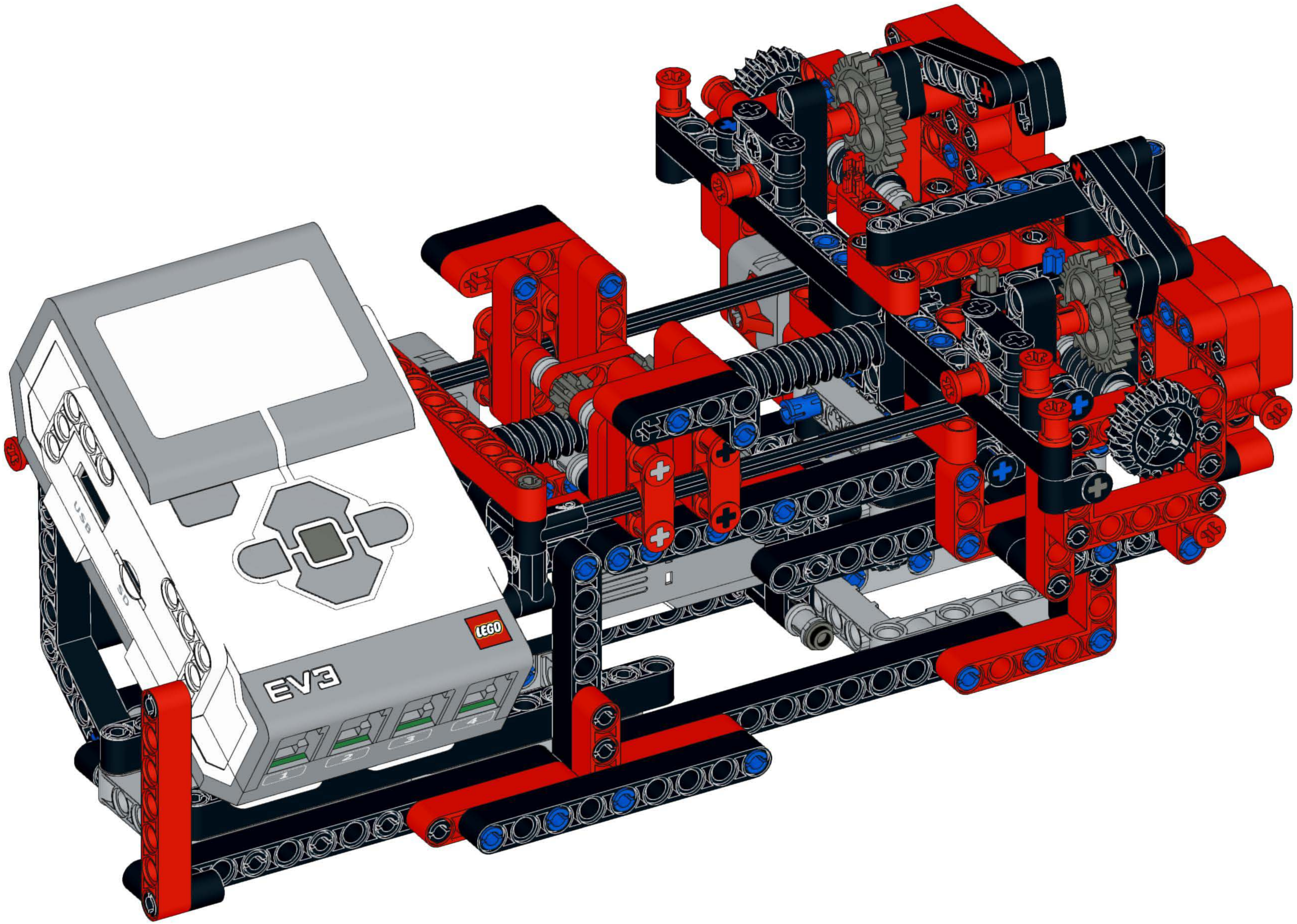

# Assembly Instructions
Orbital Shaker

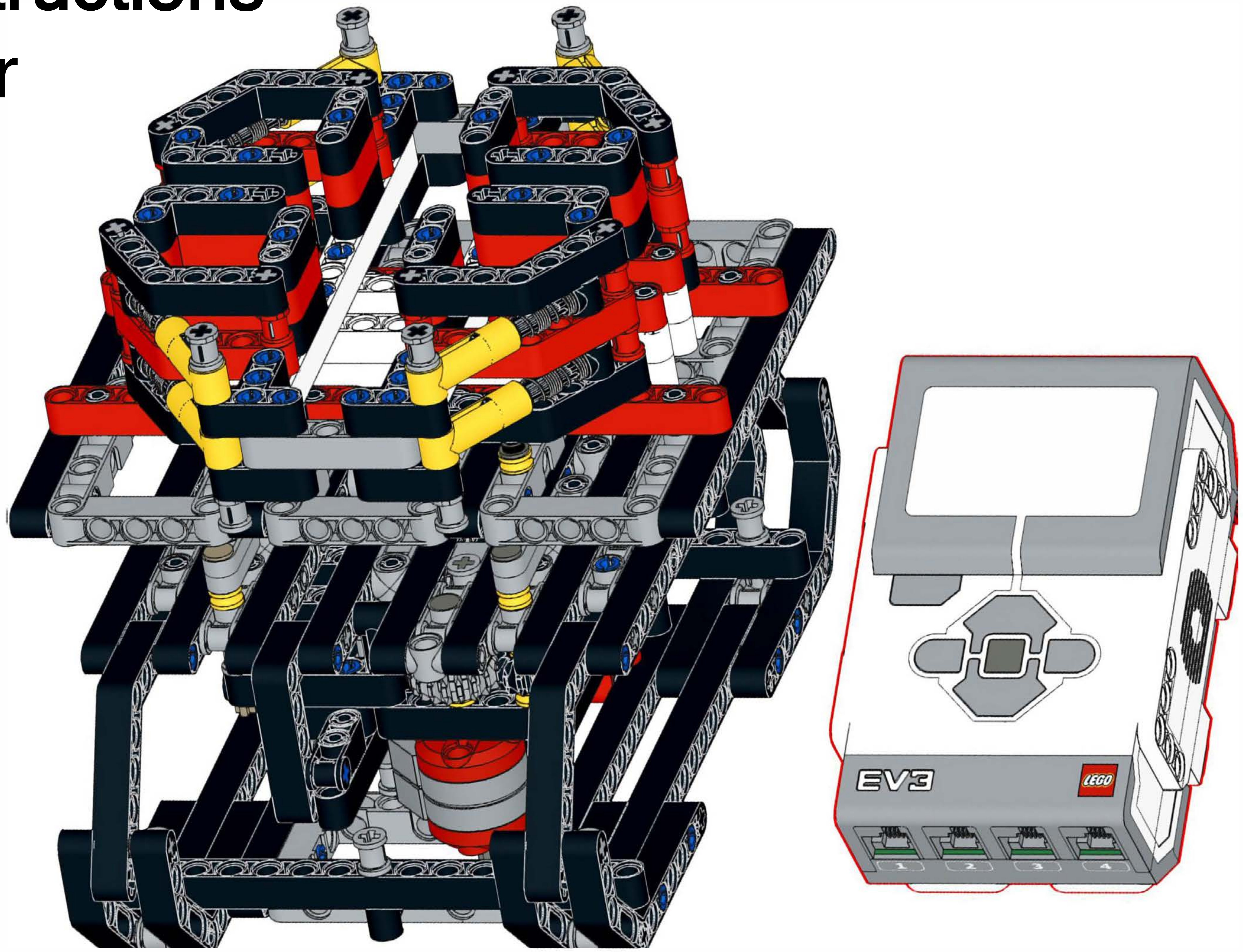

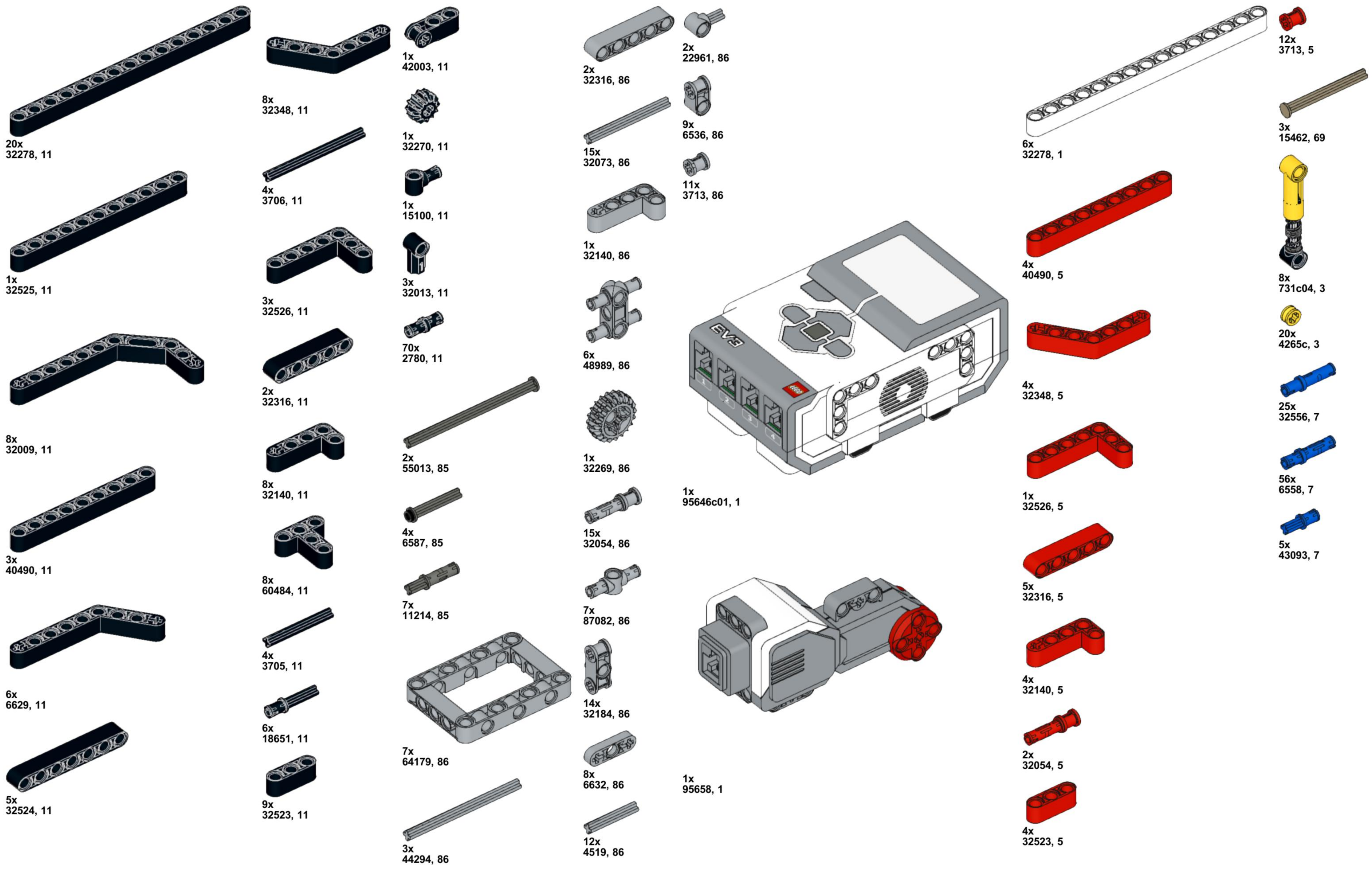

20x
32278, 11
1x
32525, 11
8x
32009, 11
3x
40490, 11
6x
6629, 11
5x
32524, 11
8x
32348, 11
4x
3706, 11
3x
32526, 11
2x
32316, 11
8x
32140, 11
8x
60484, 11
4x
3705, 11
6x
18651, 11
9x
32523, 11
1x
42003, 11
1x
32270, 11
1x
15100, 11
3x
32013, 11
70x
2780, 11
2x
55013, 85
4x
6587, 85
7x
11214, 85
7x
64179, 86
3x
44294, 86
2x
32316, 86
15x
32073, 86
1x
32140, 86
6x
48989, 86
1x
32269, 86
15x
32054, 86
7x
87082, 86
14x
32184, 86
8x
6632, 86
12x
4519, 86
2x
22961, 86
9x
6536, 86
11x
3713, 86
EV3
1x
95646c01, 1
1x
95658, 1
6x
32278, 1
4x
40490, 5
4x
32348, 5
1x
32526, 5
5x
32316, 5
4x
32140, 5
2x
32054, 5
4x
32523, 5
12x
3713, 5
3x
15462, 69
8x
731c04, 3
20x
4265c, 3
25x
32556, 7
56x
6558, 7
5x
43093, 7

1

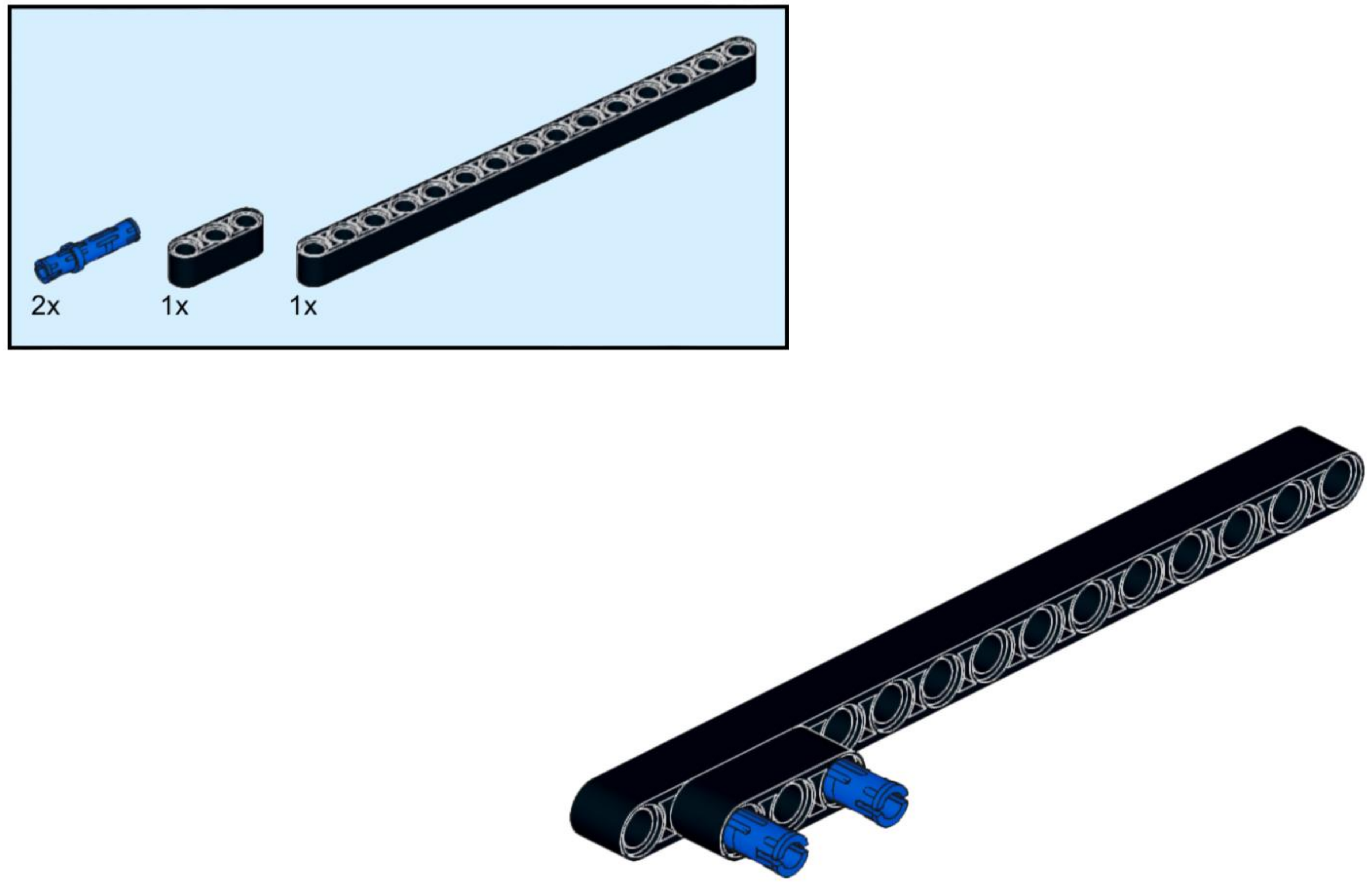


2

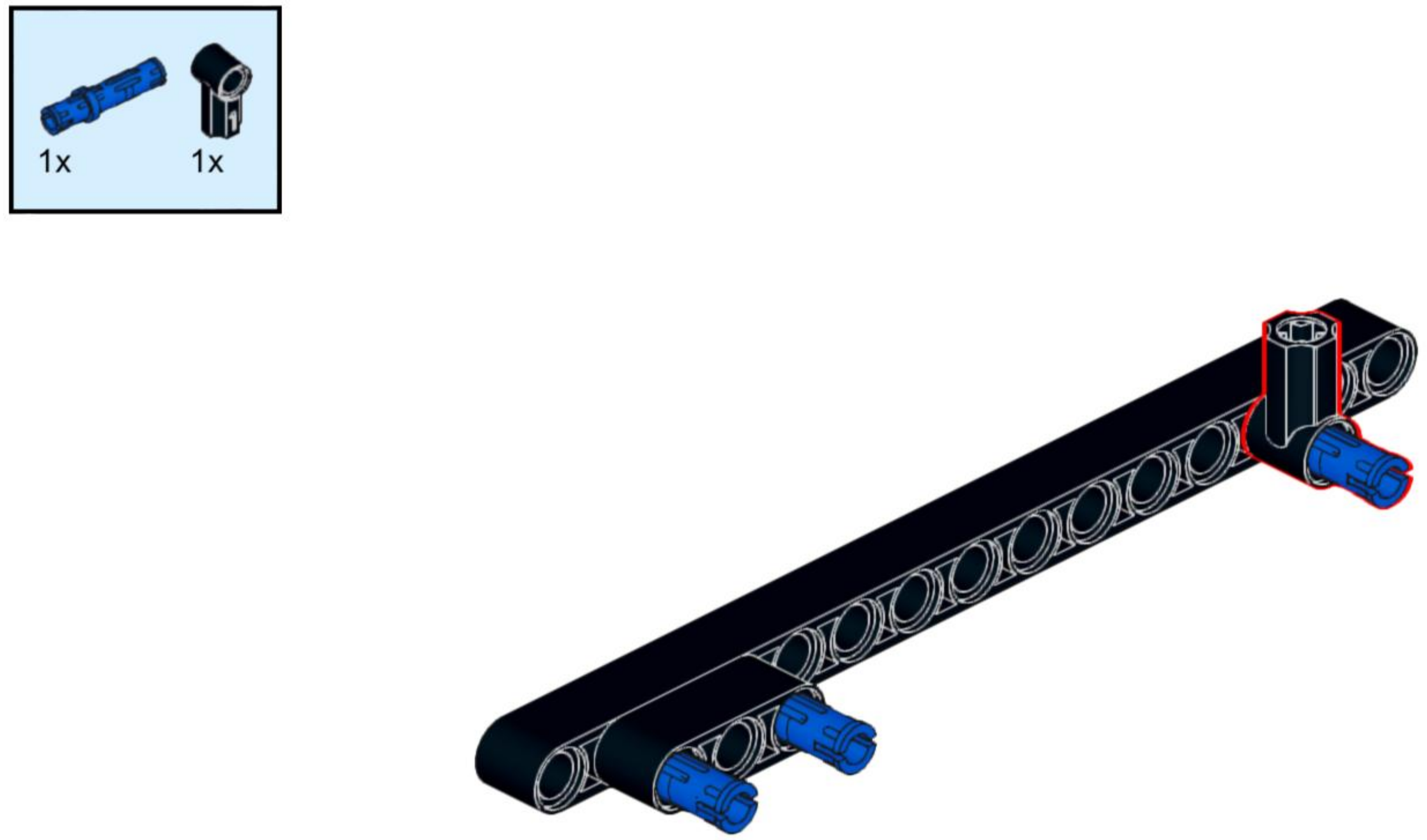


3

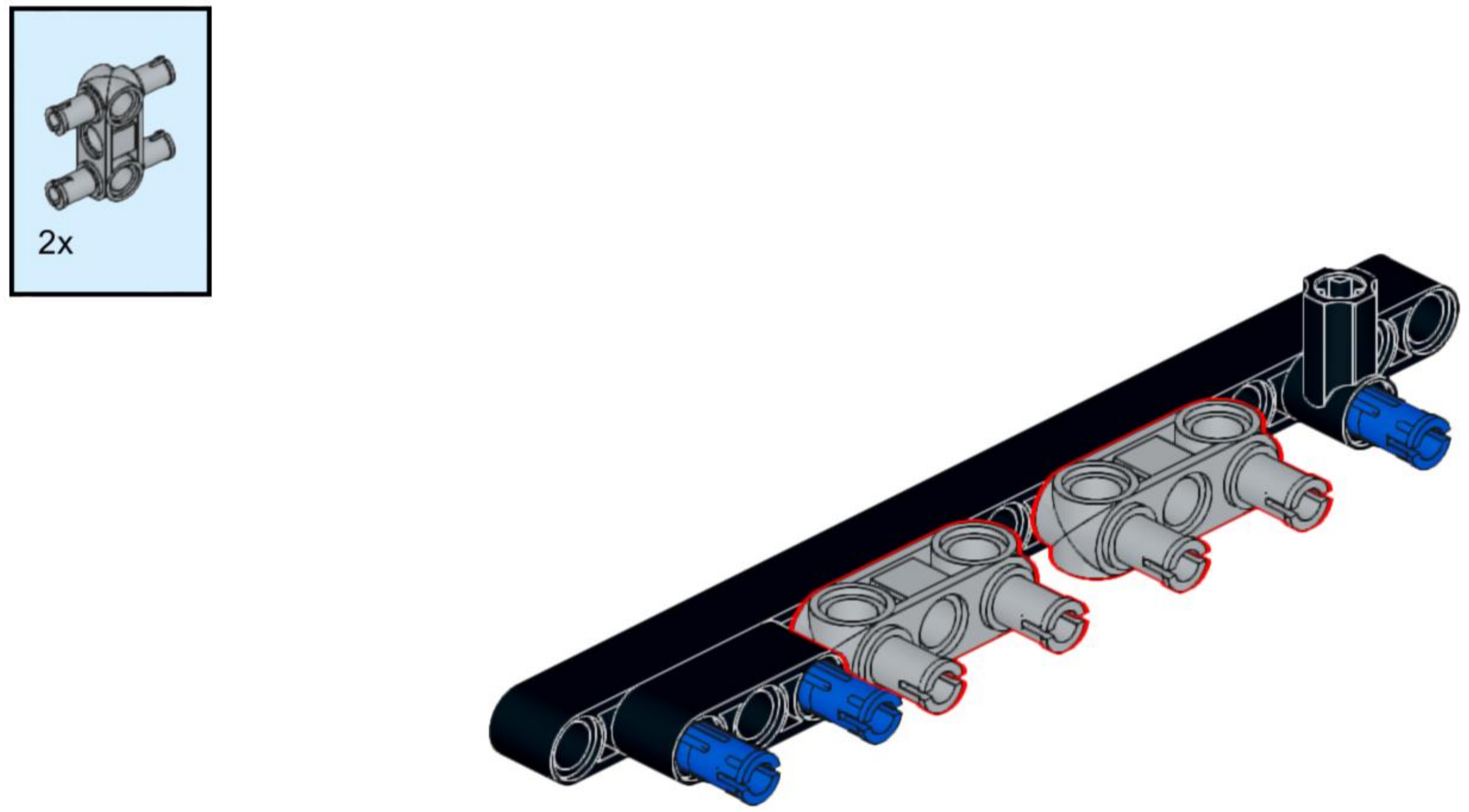


4

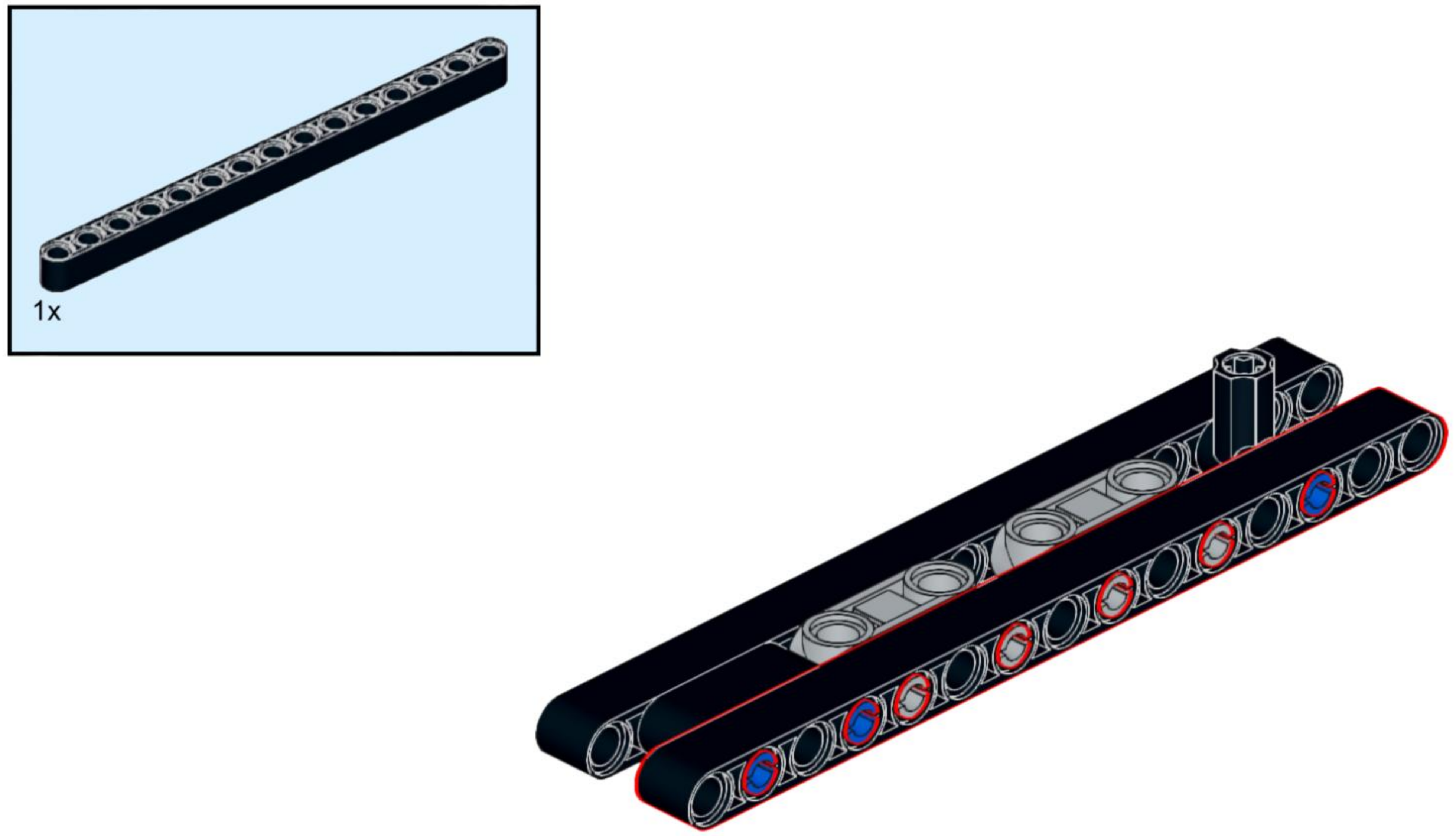


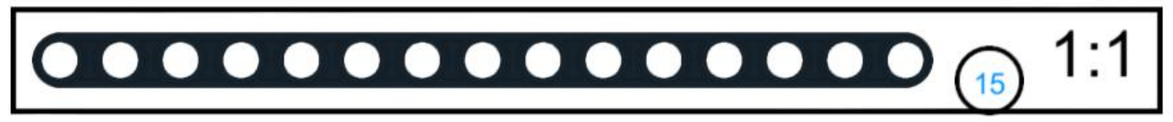

5

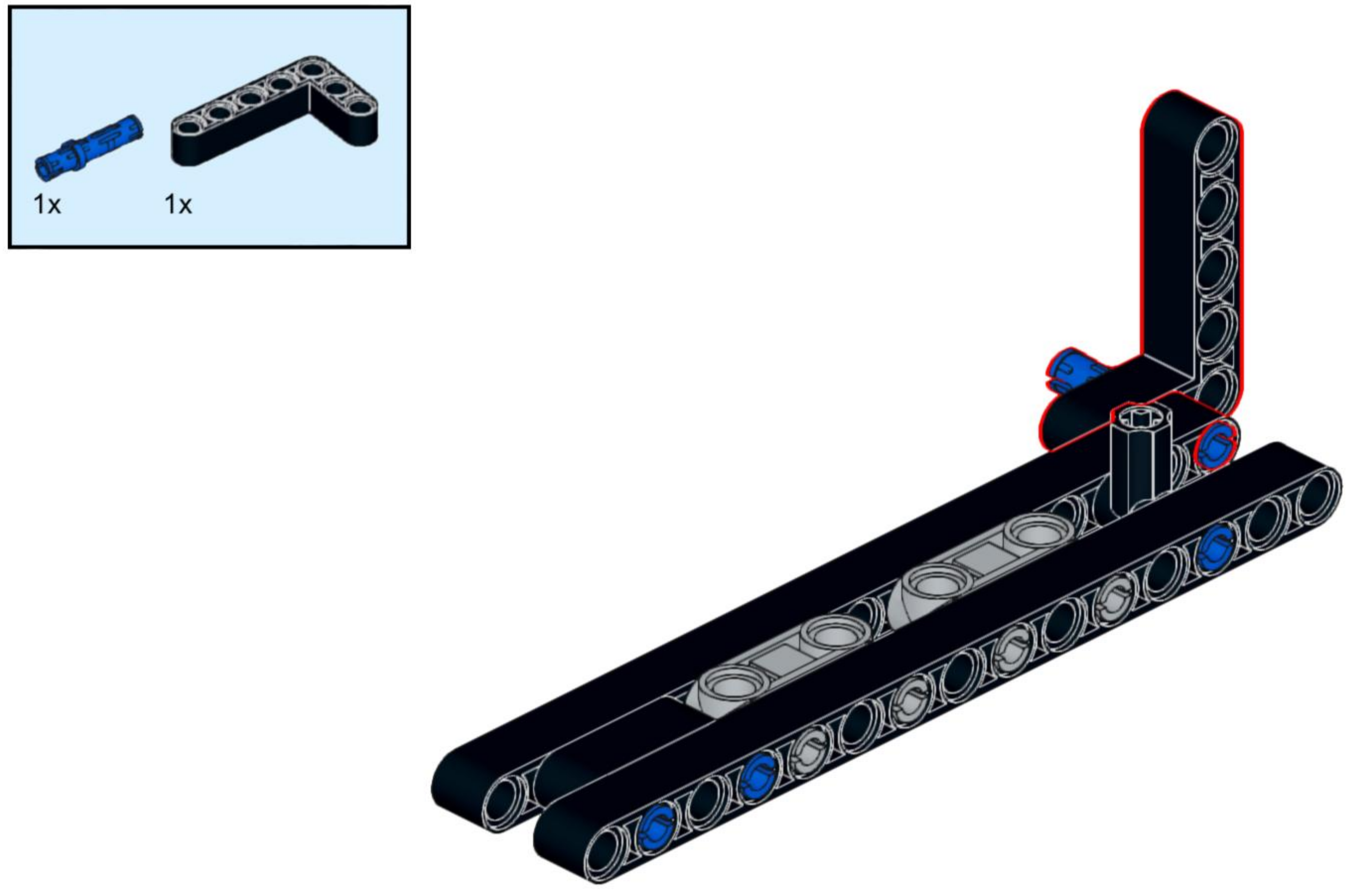


6

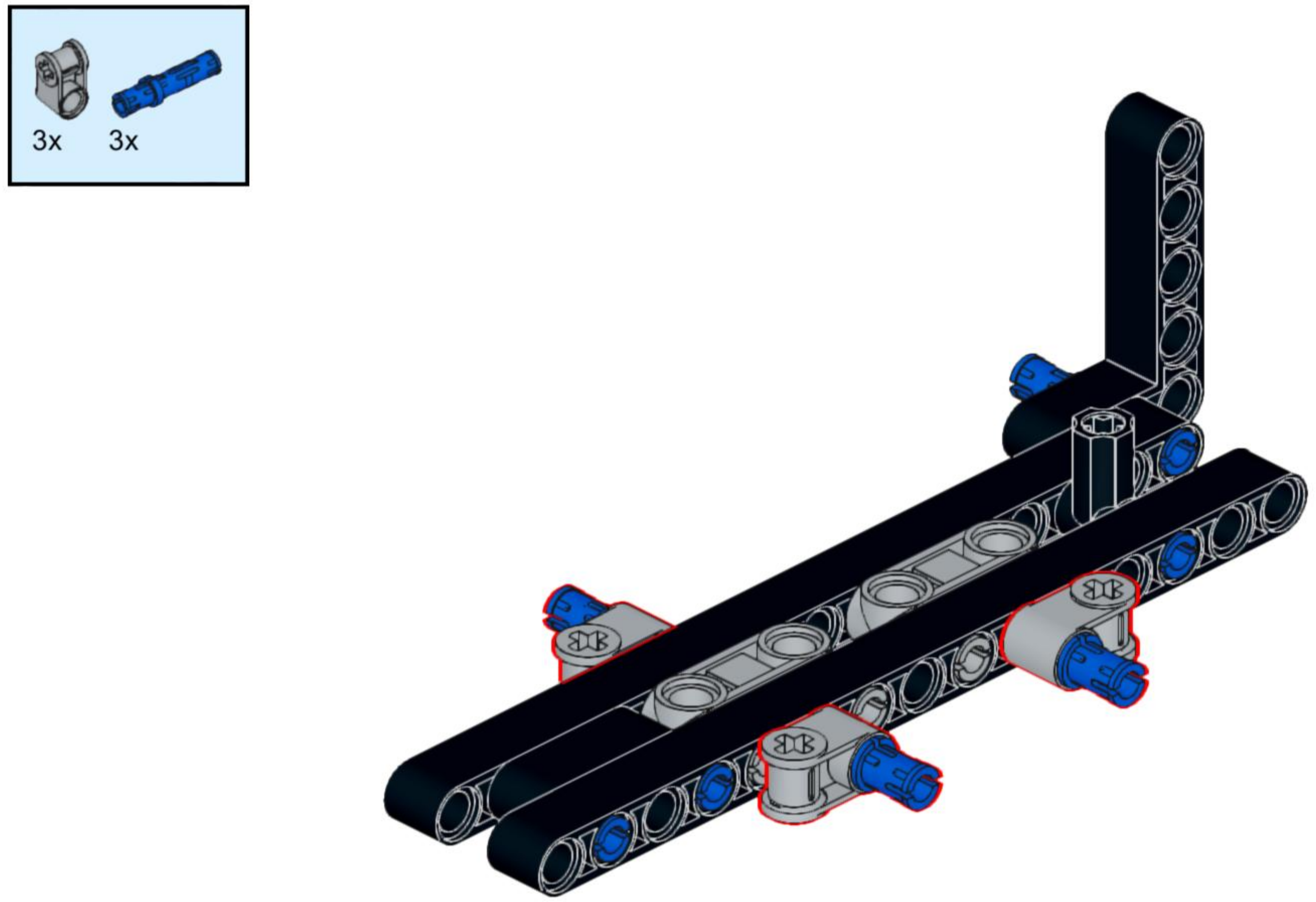


7

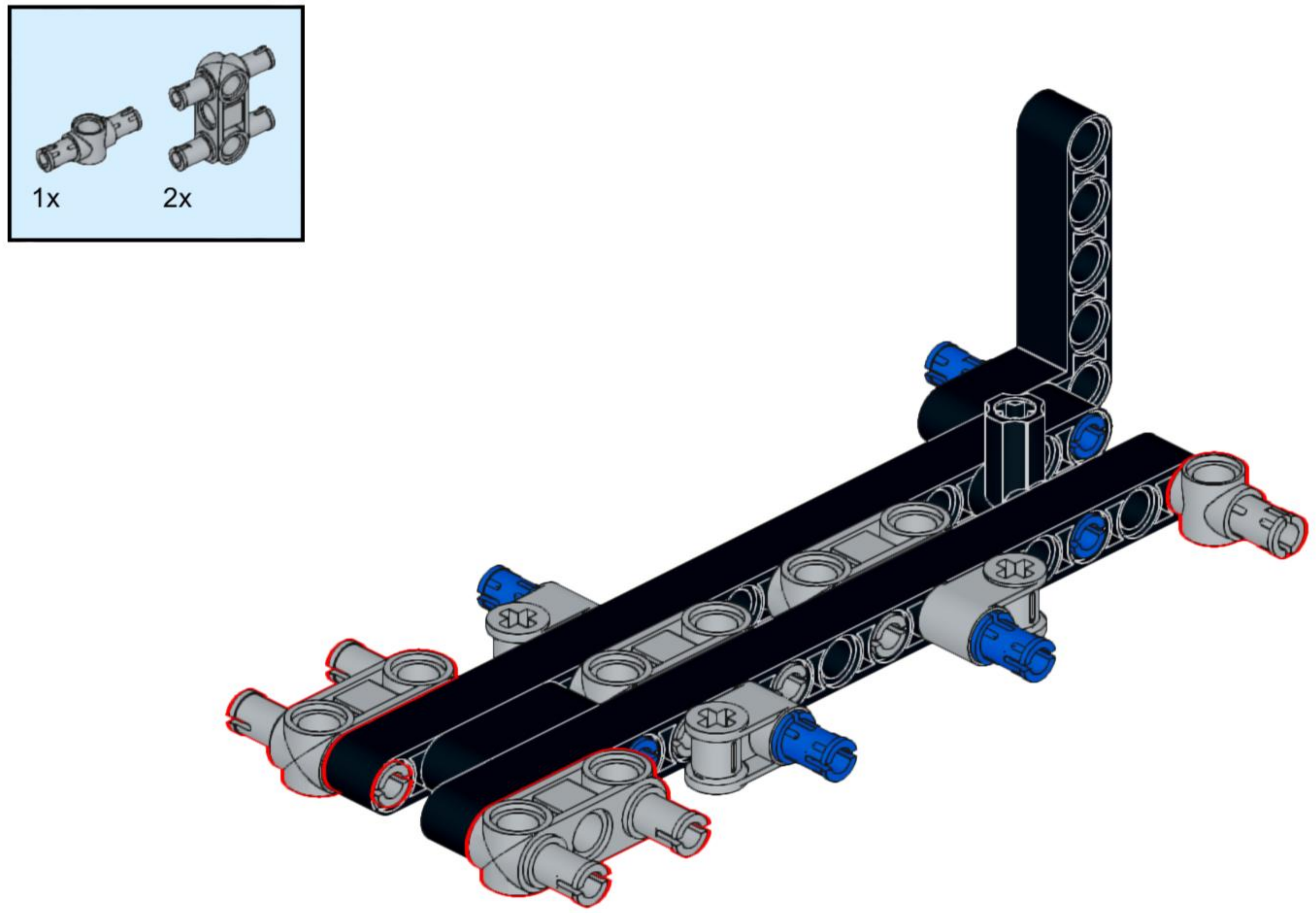


8

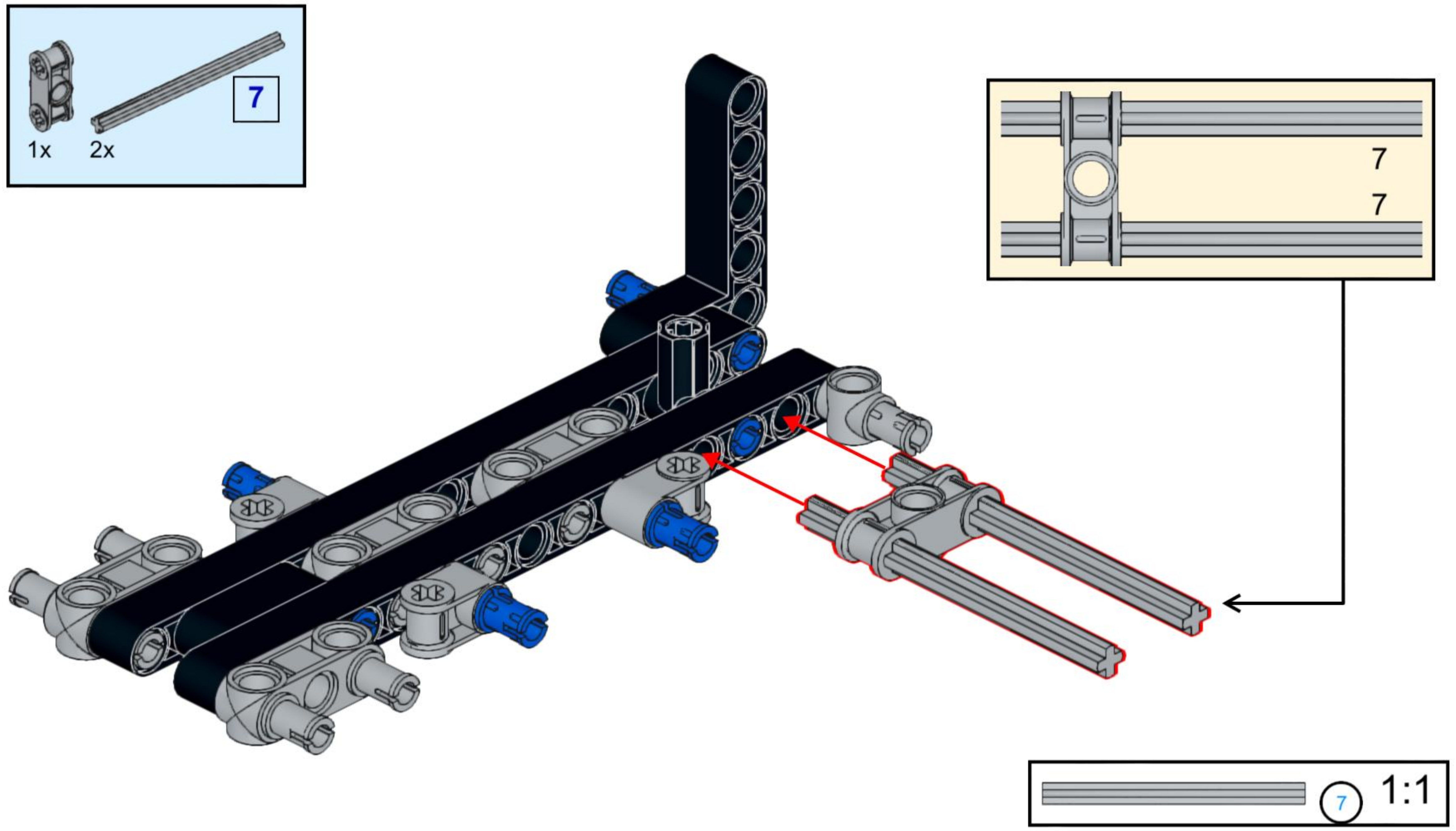

9

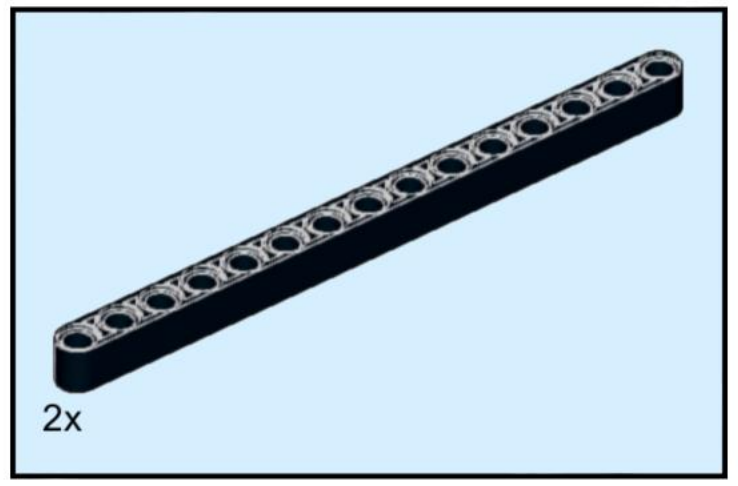


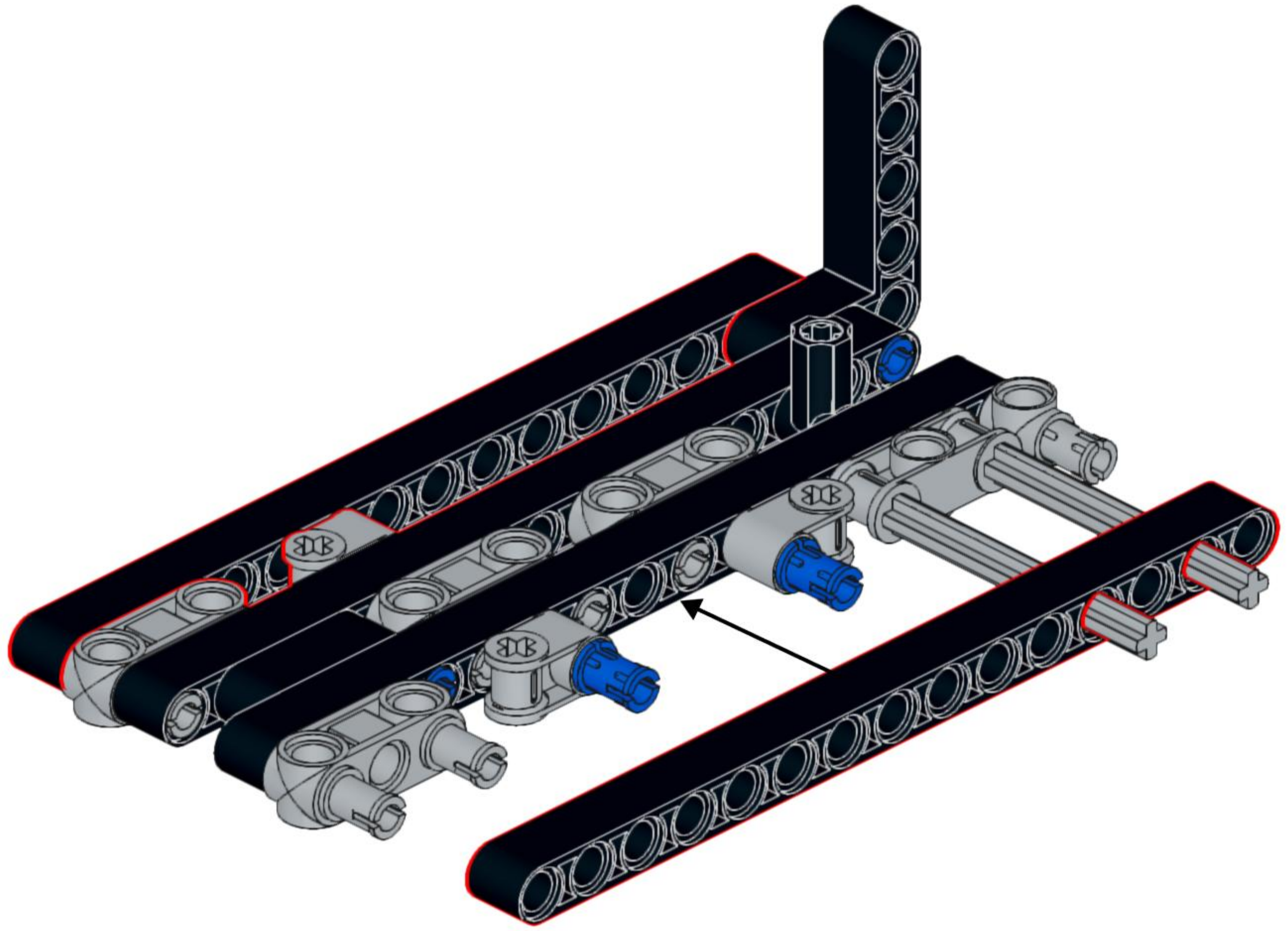

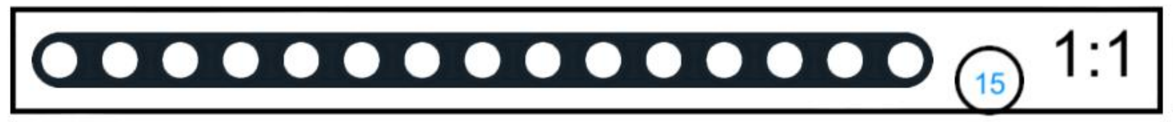


10

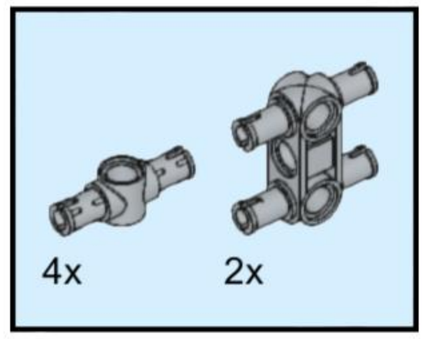


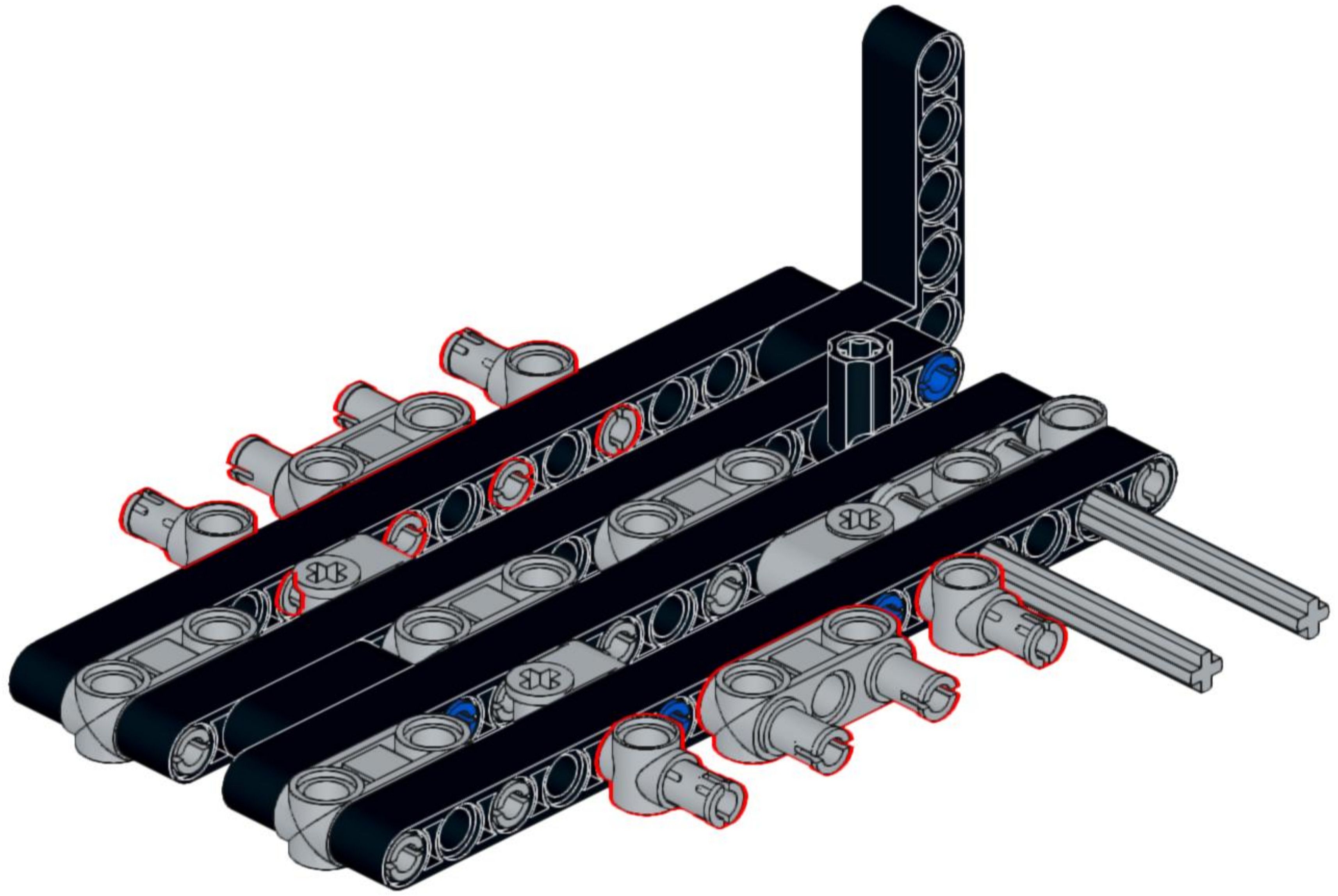

# 11

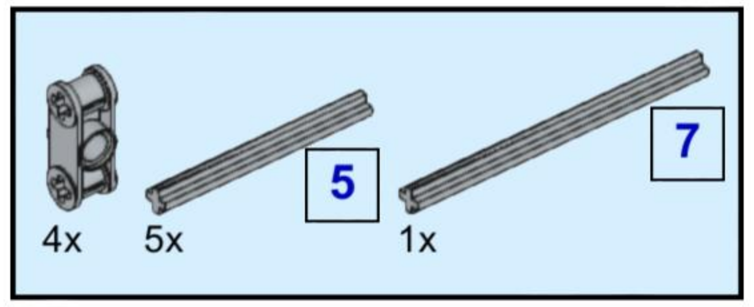


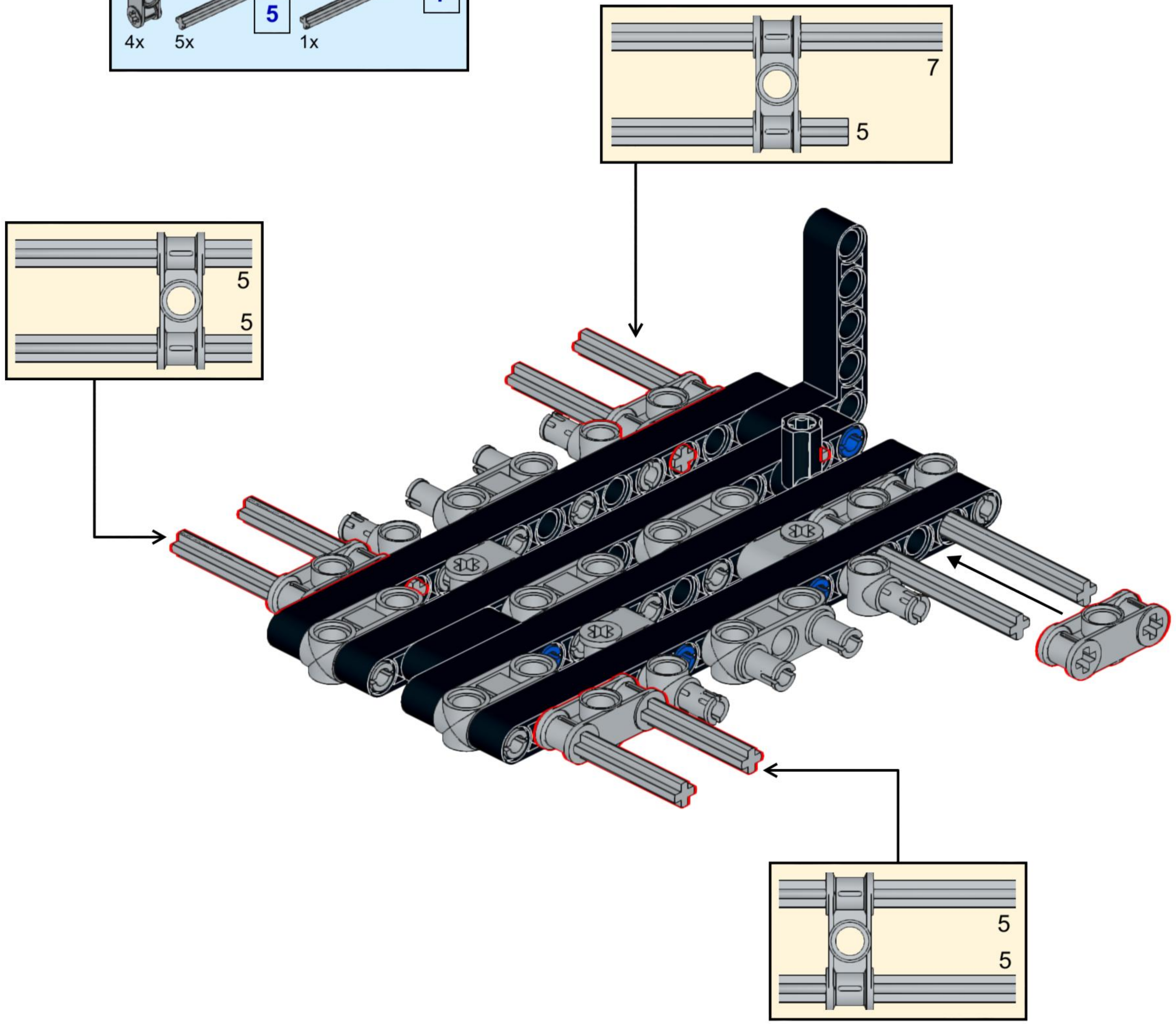


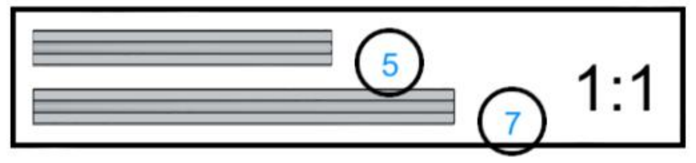


# 12

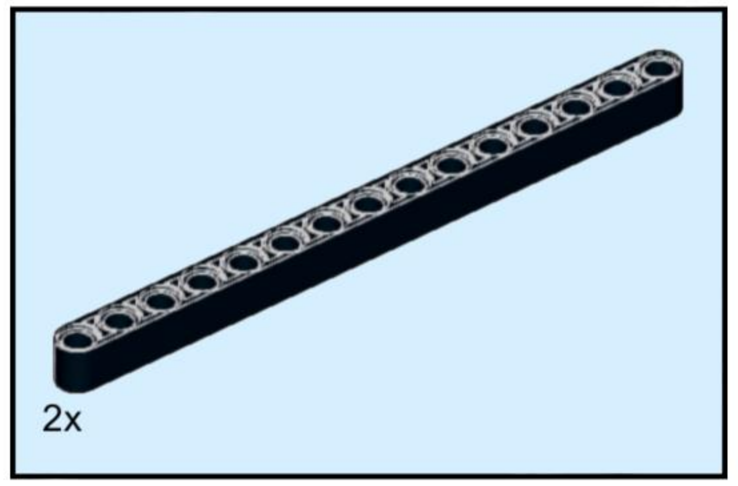


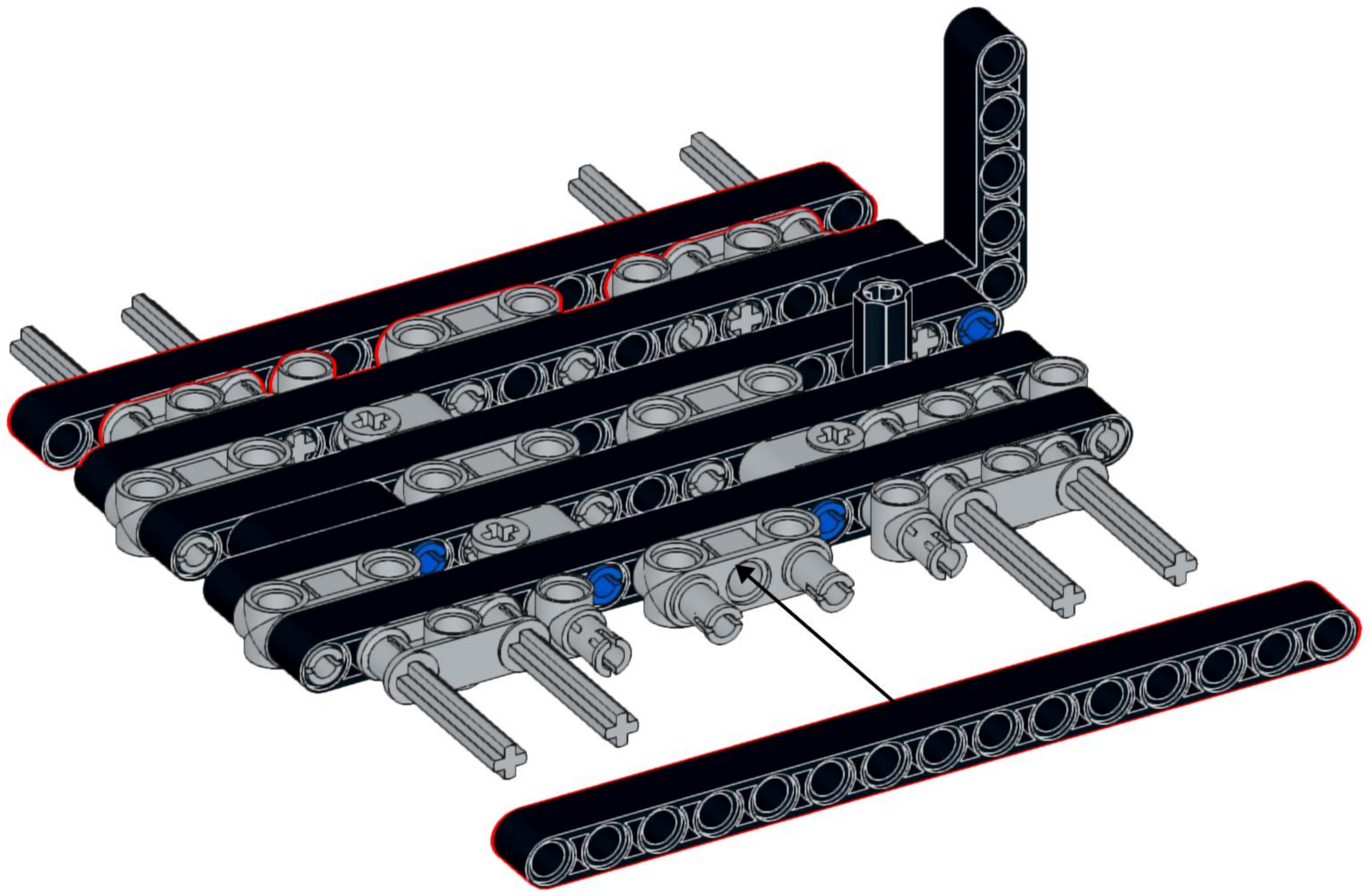

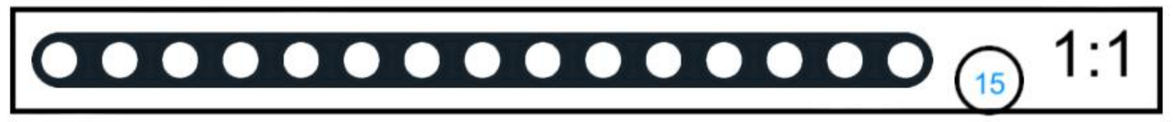

# 13

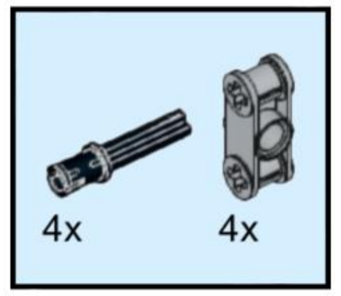


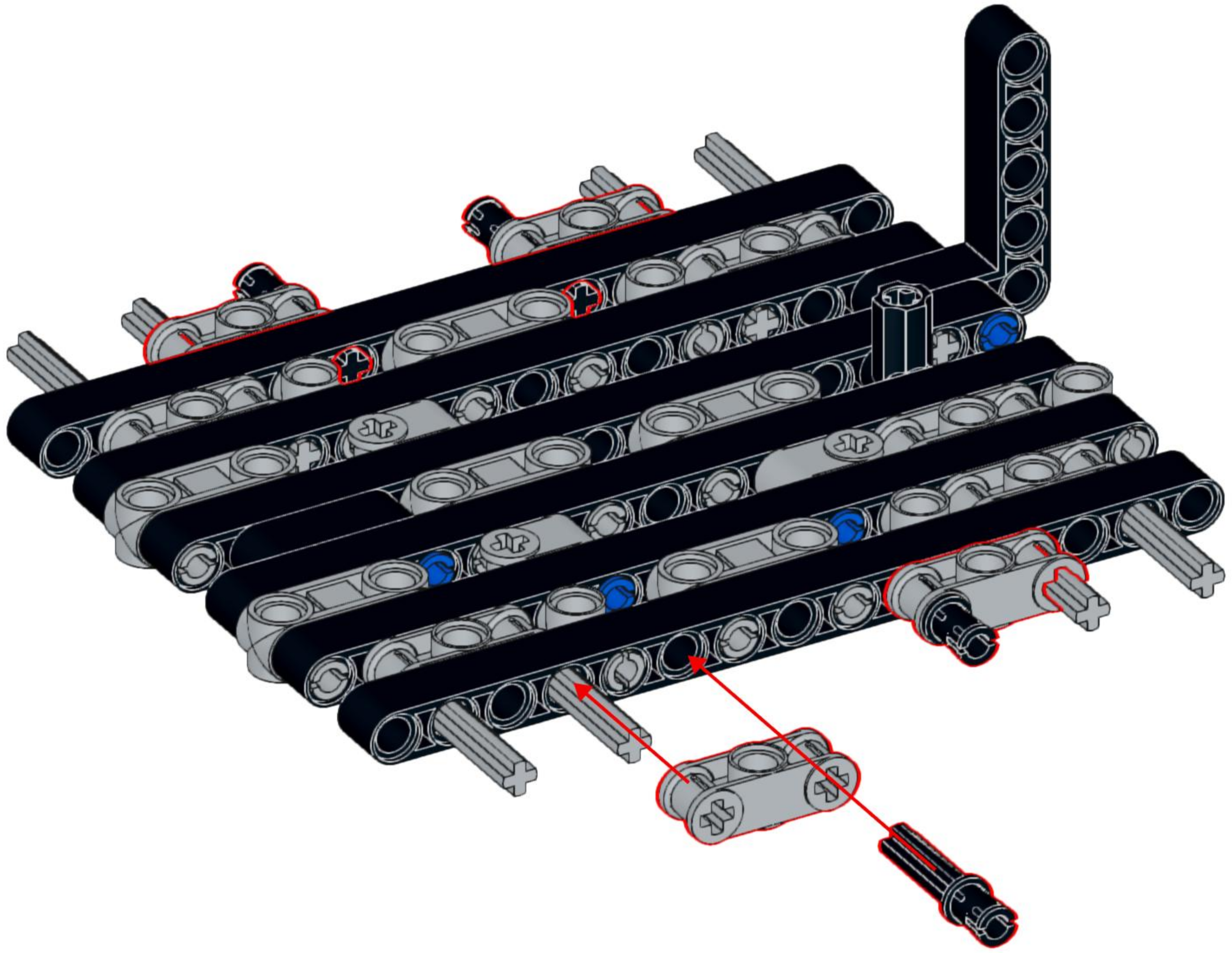

# 14

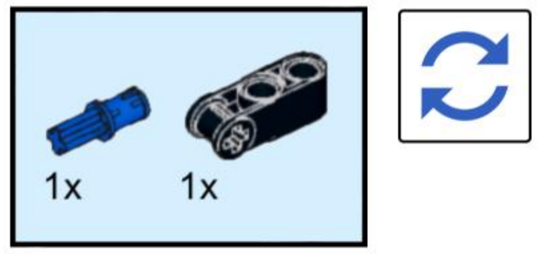


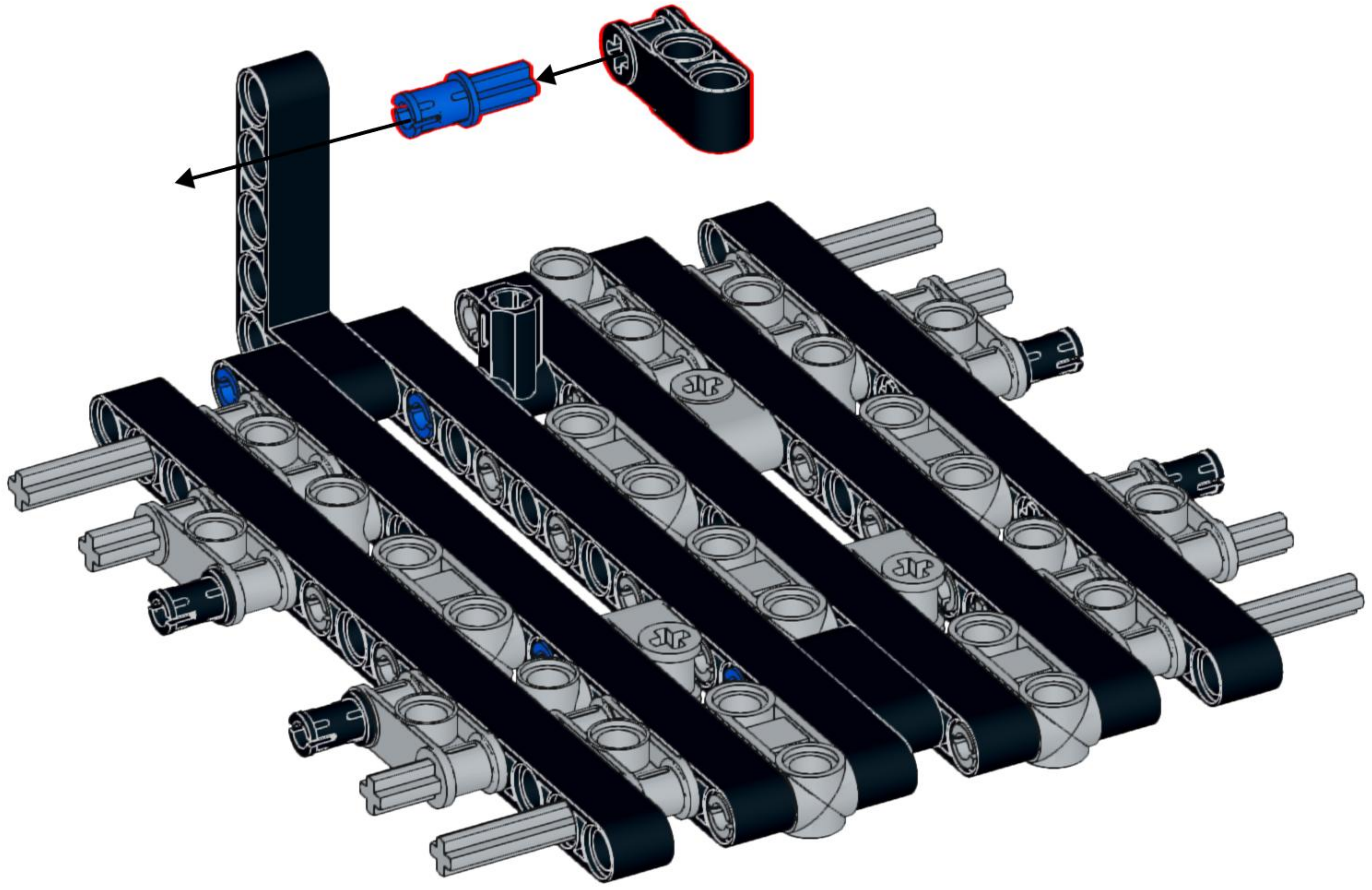

# 15

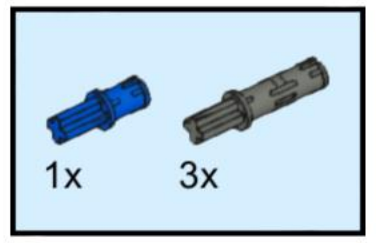


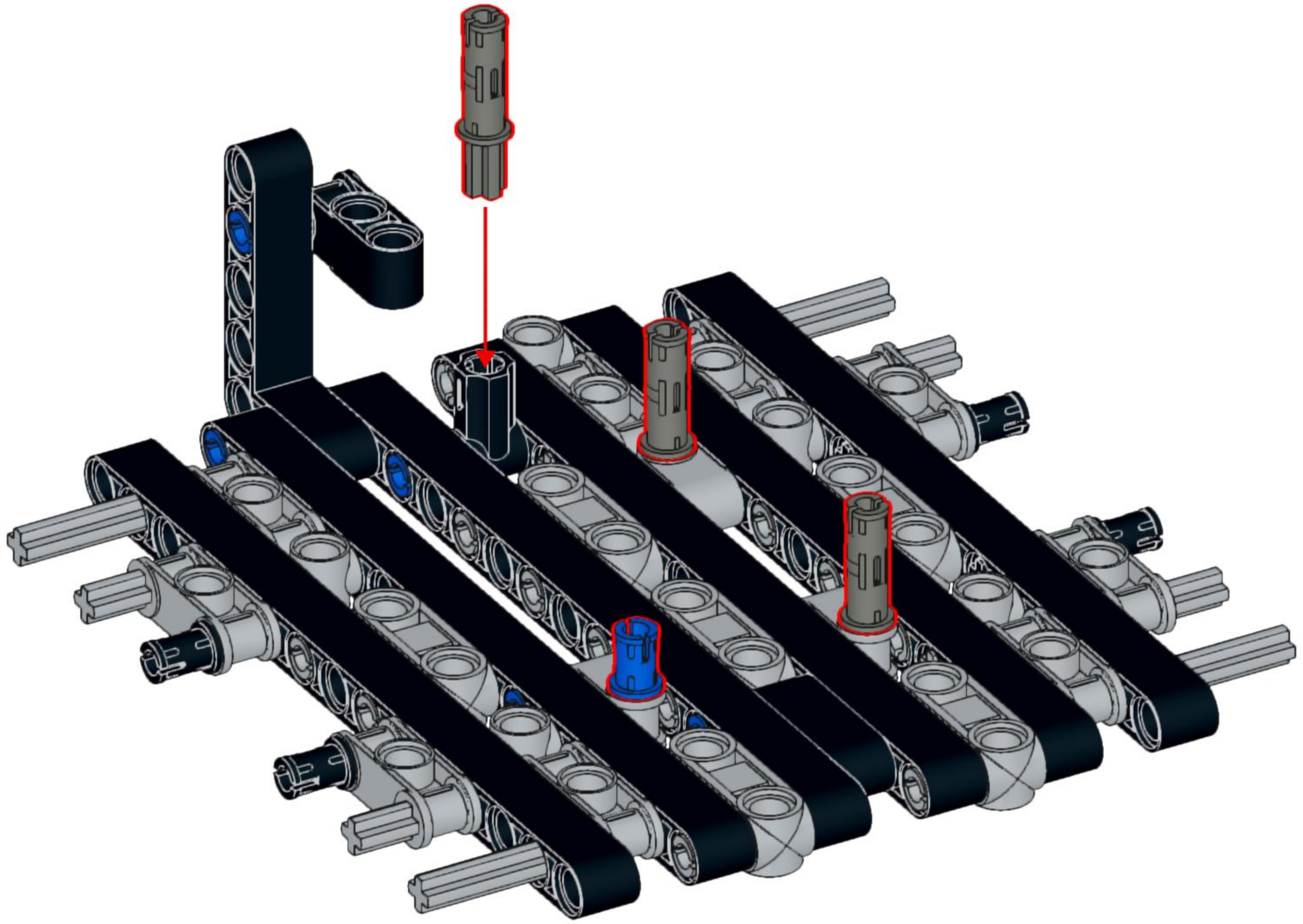

# 16

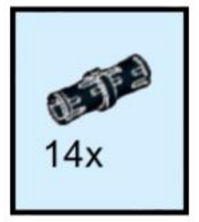


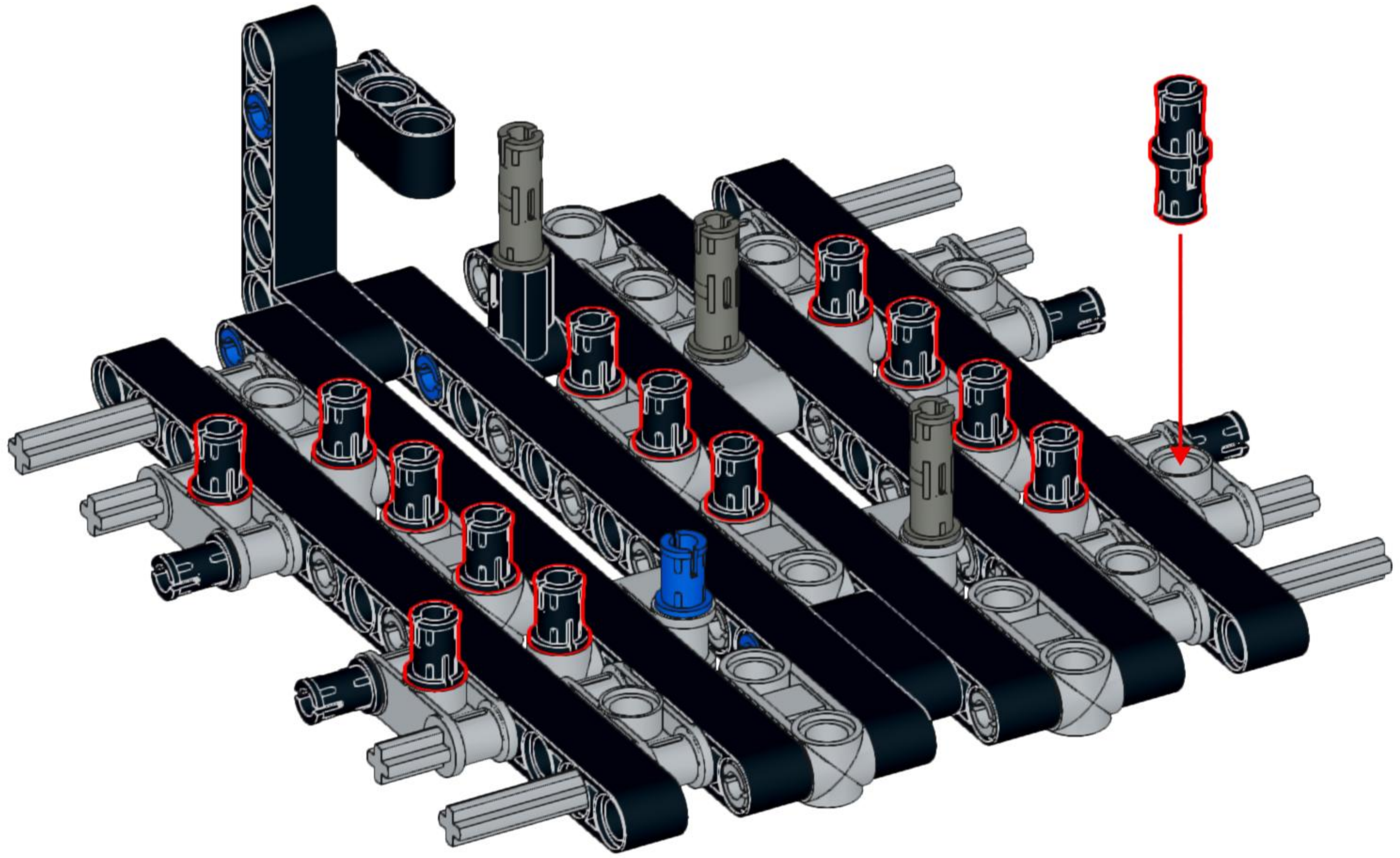

# 17

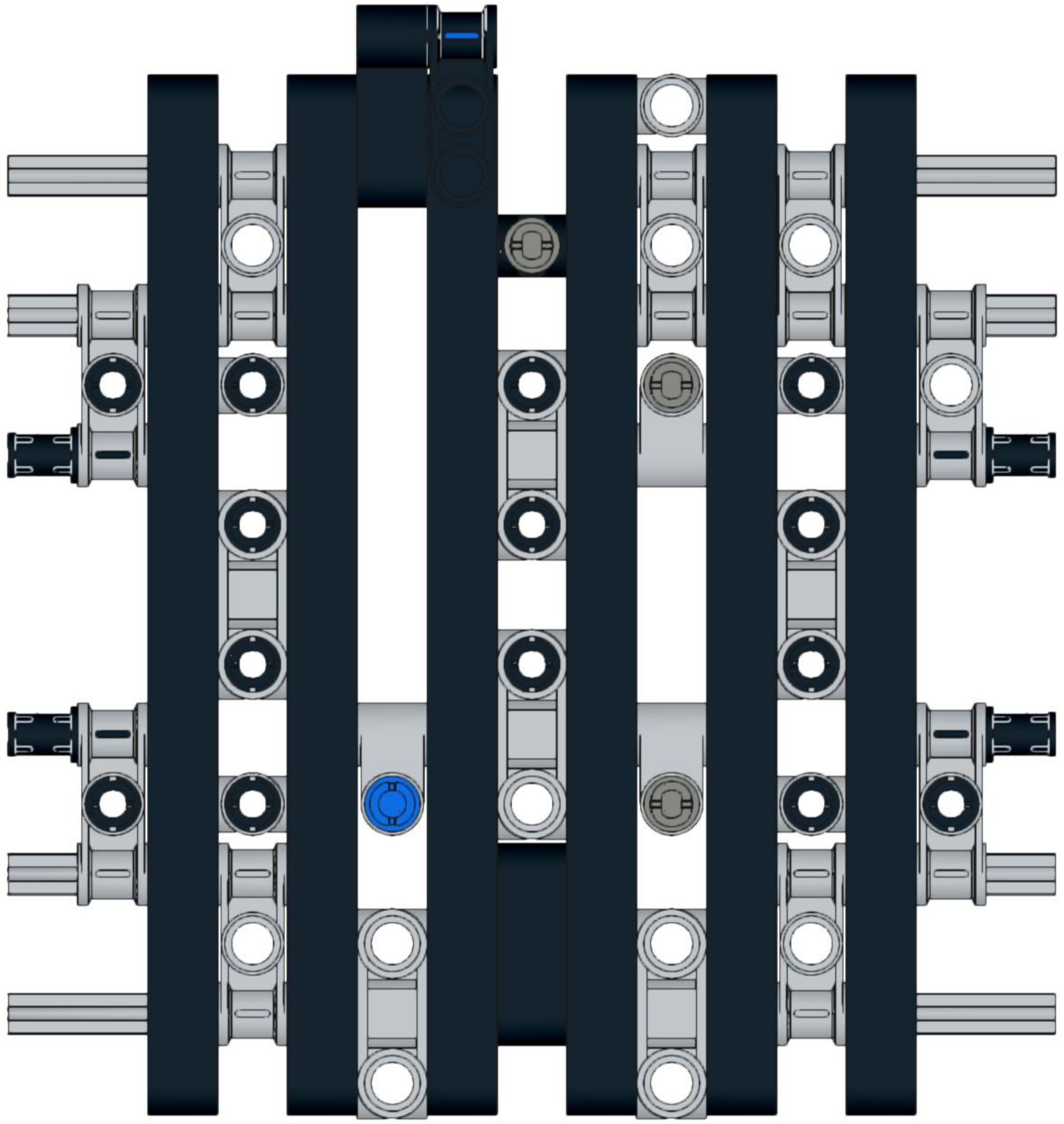

# 18

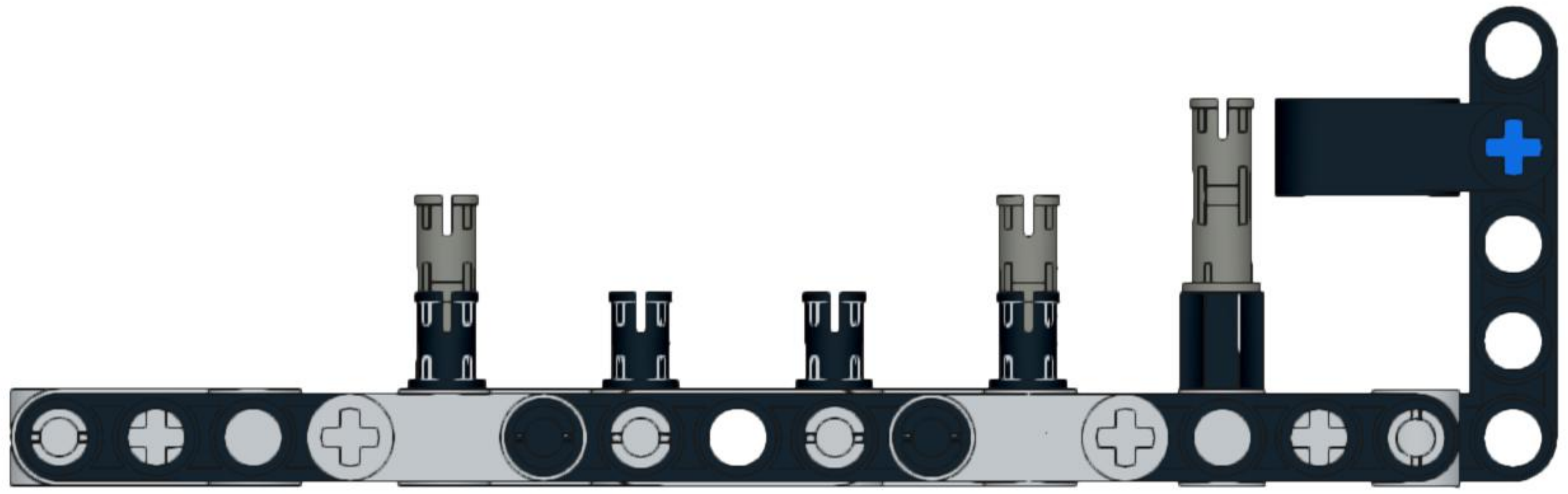

## 19

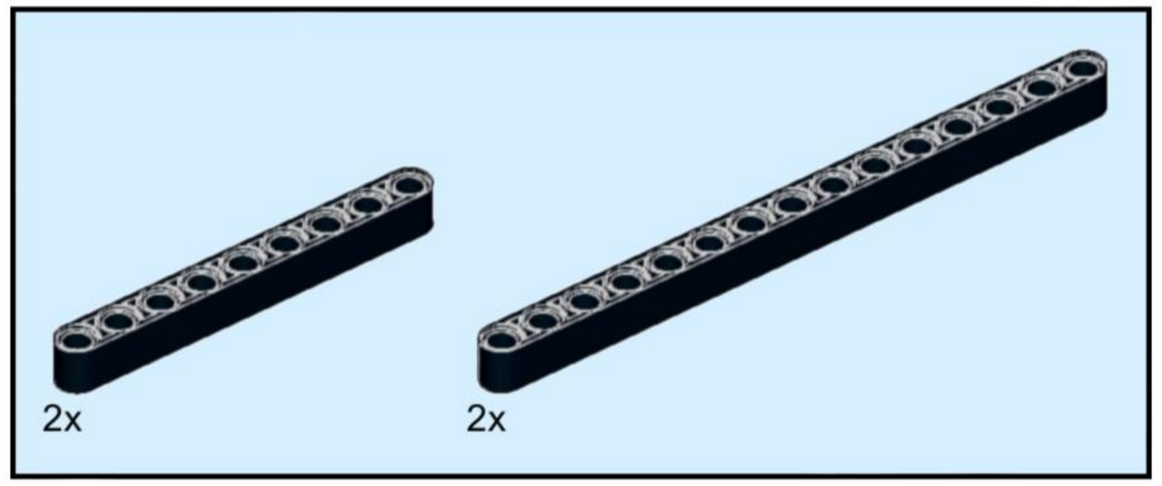


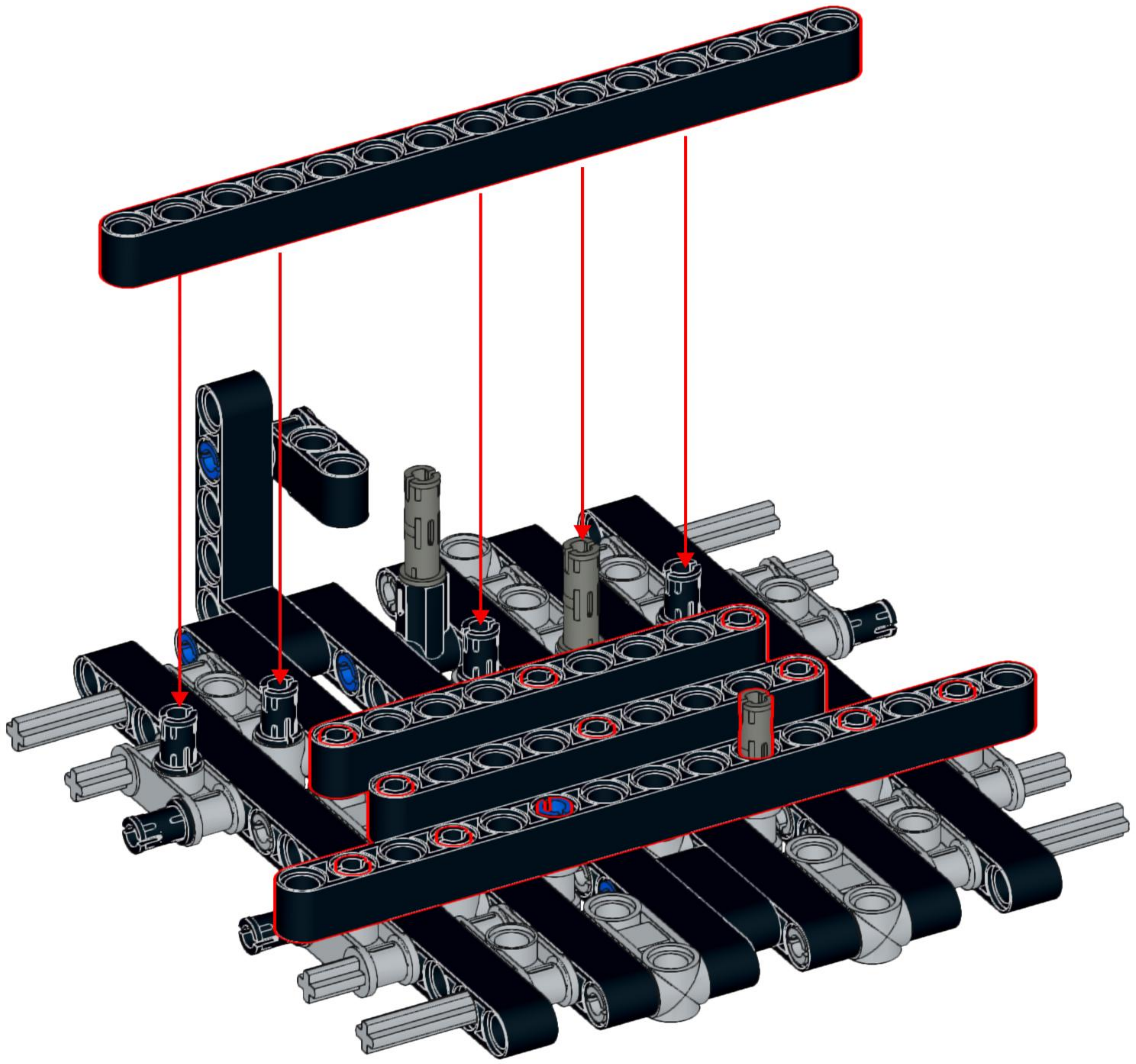

## 20

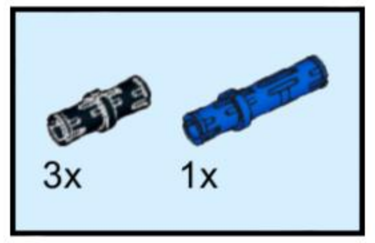


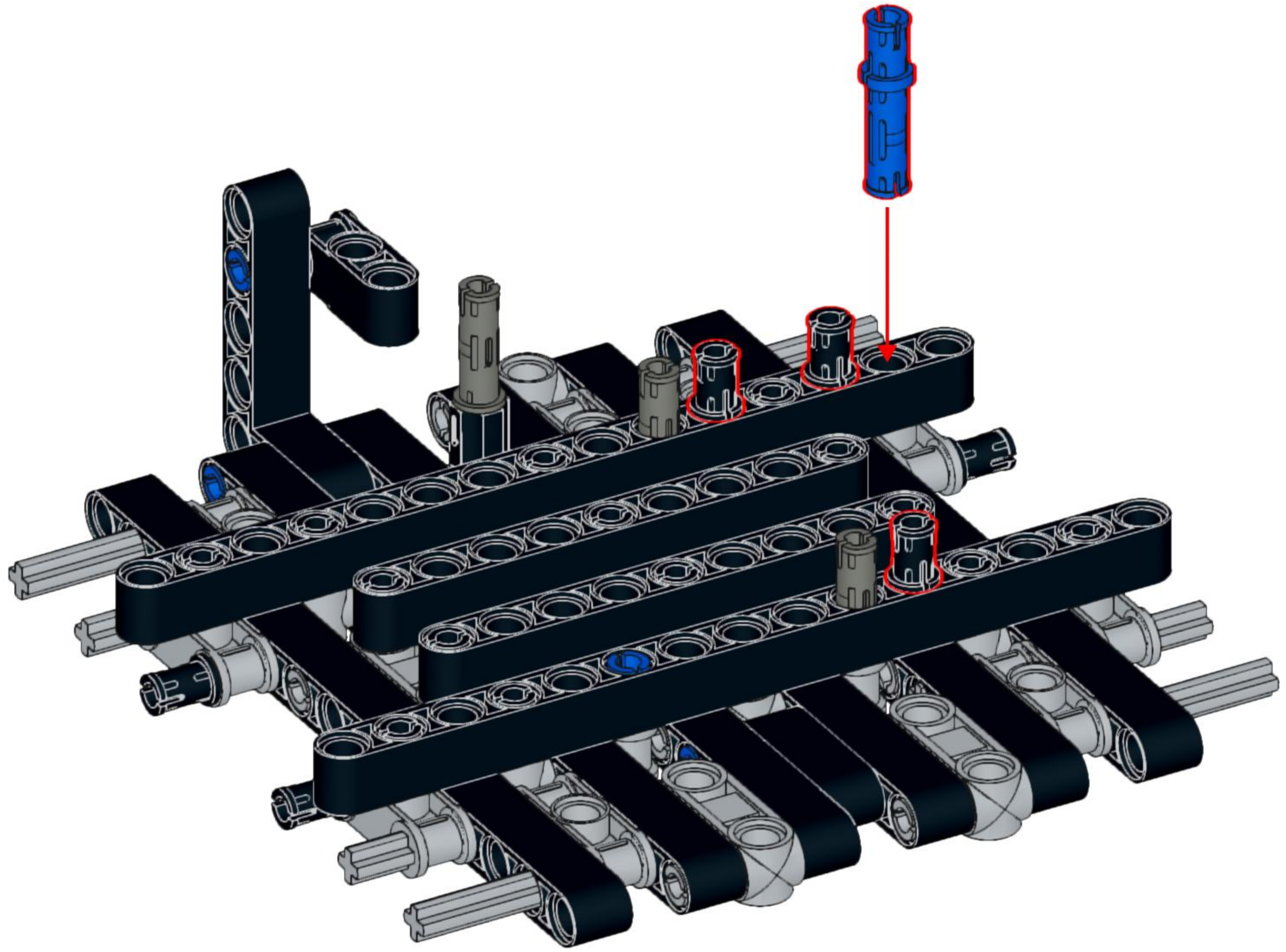

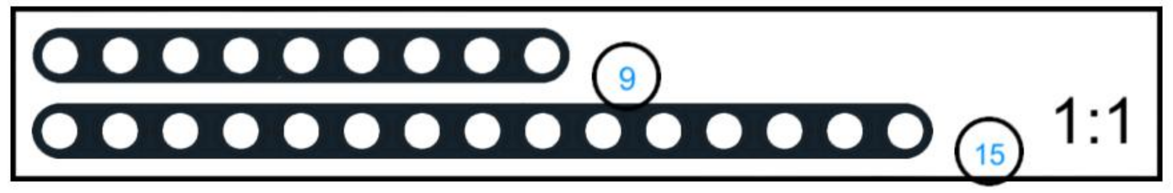

## 21

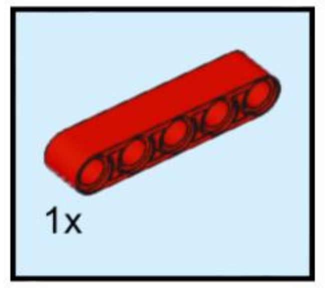


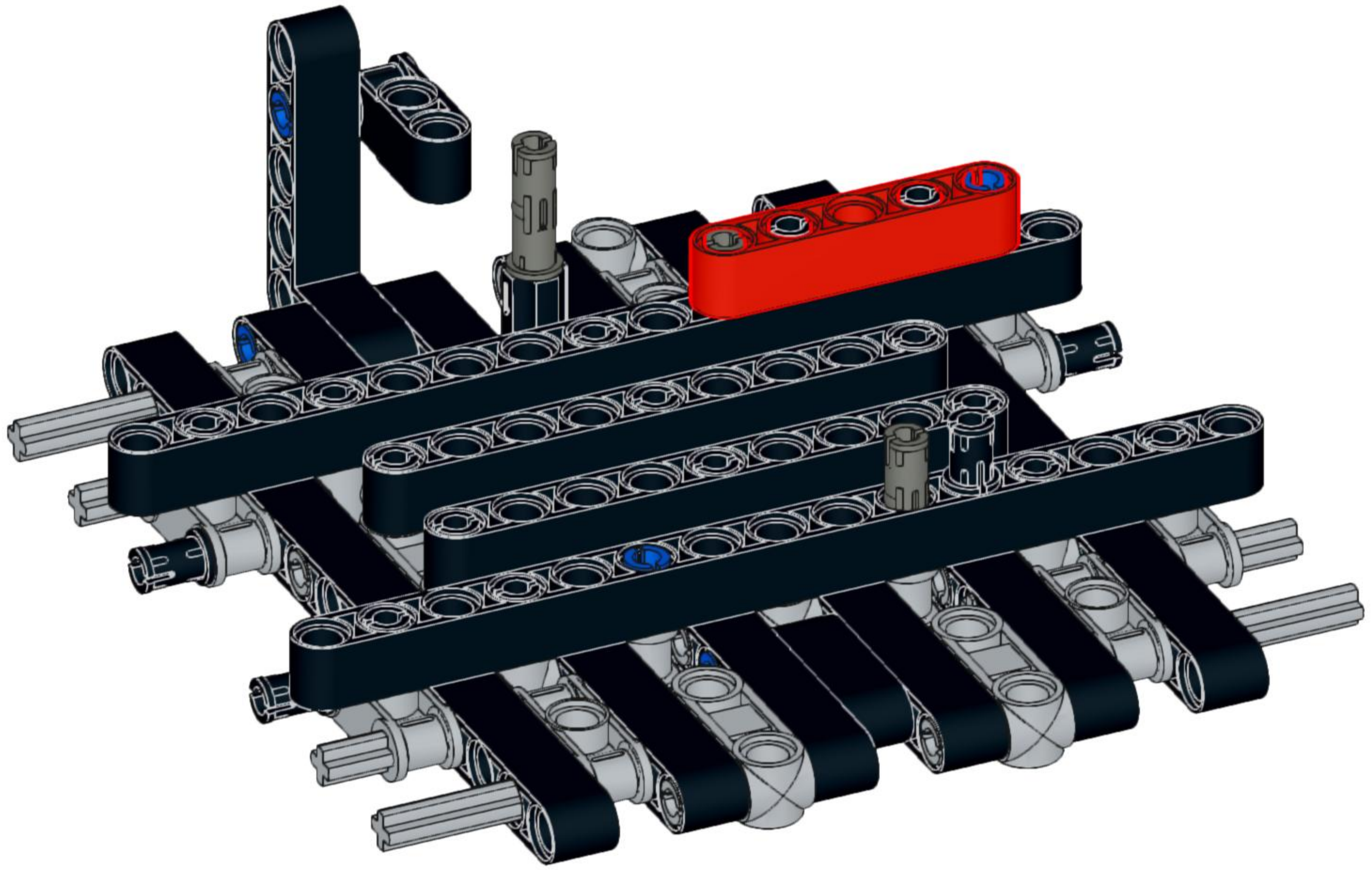

## 22

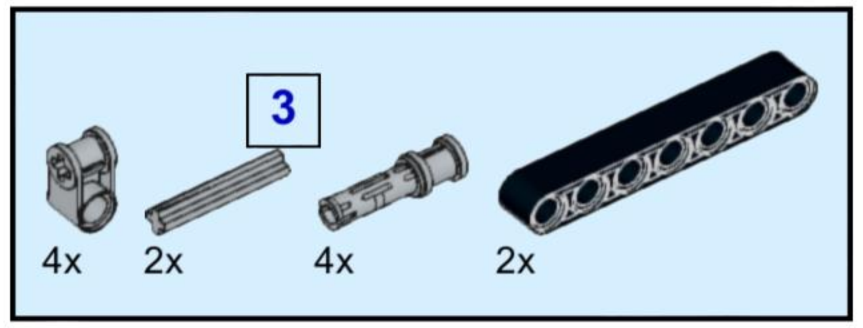


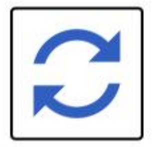

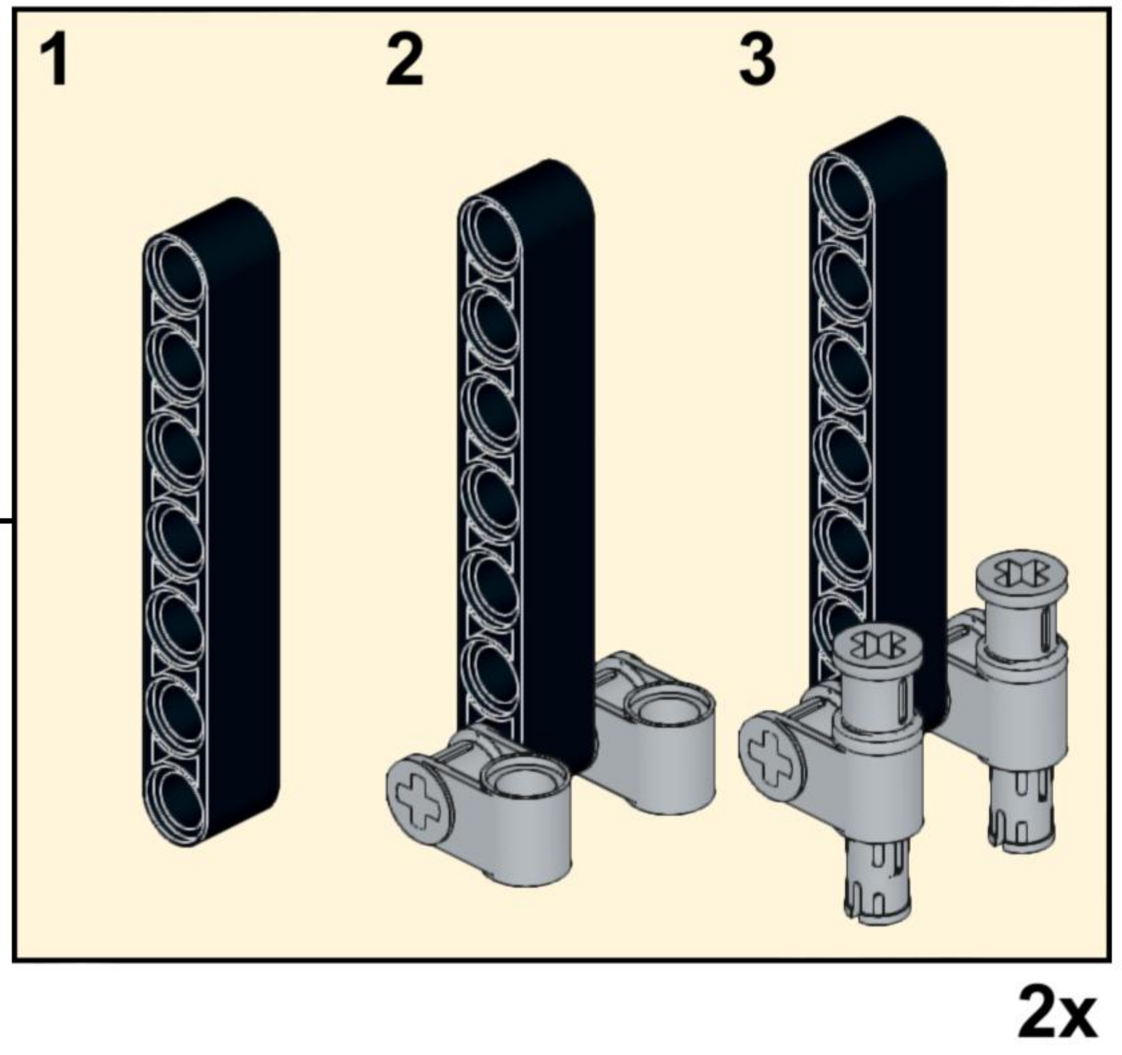


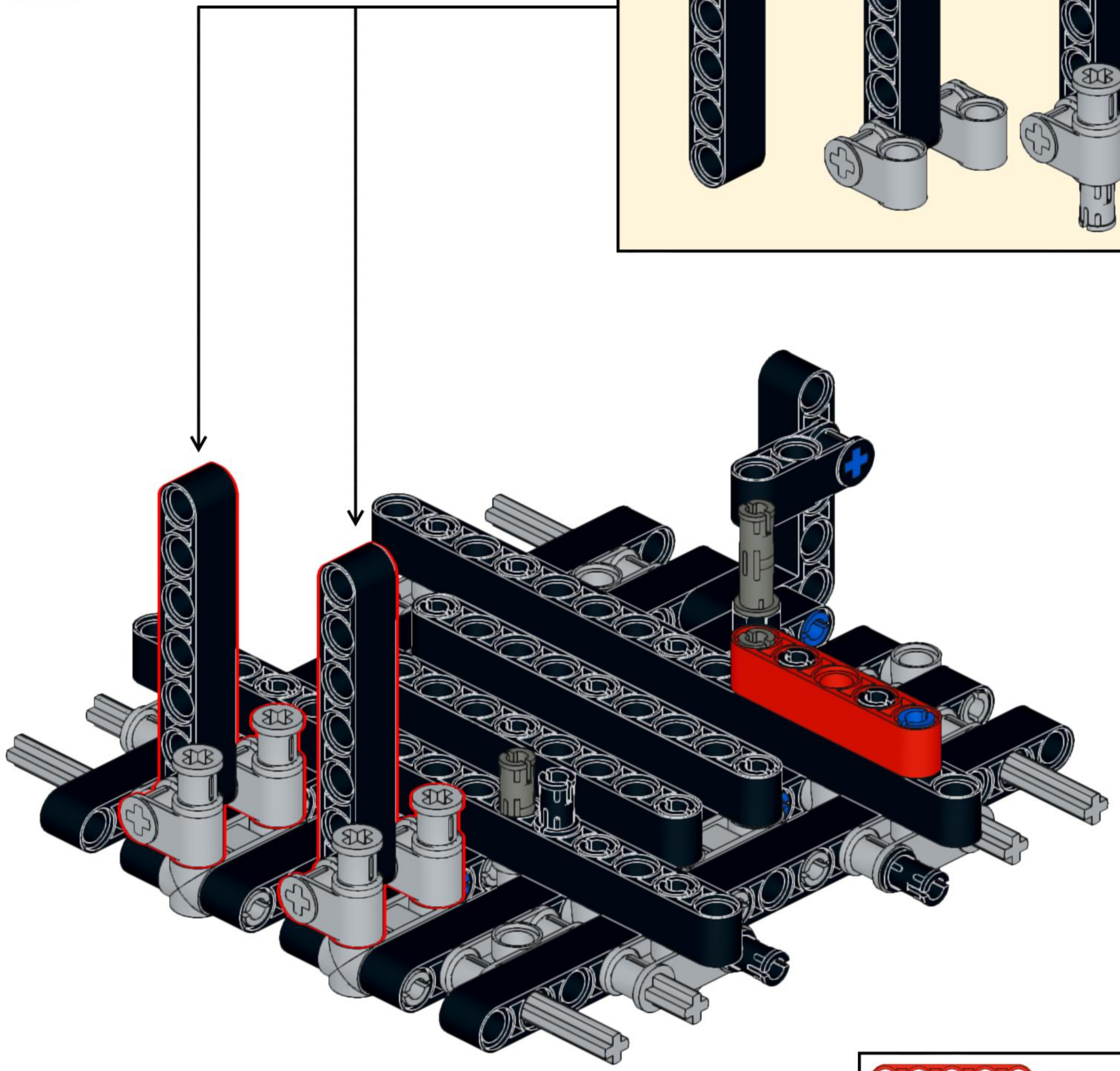

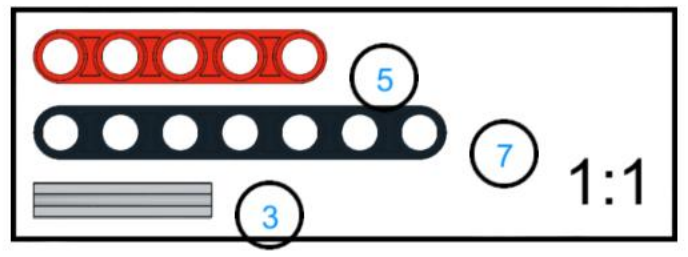

## 23

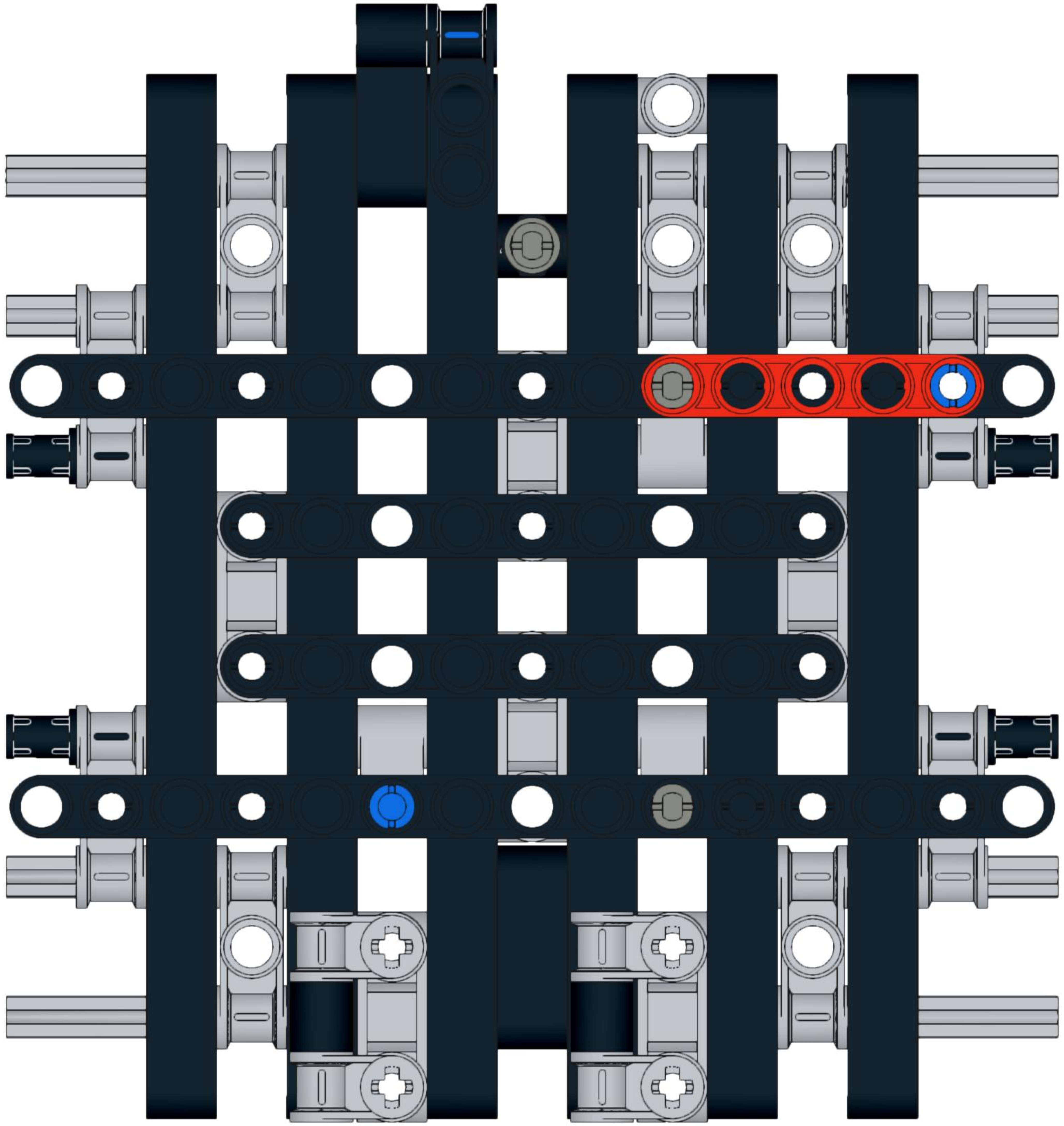

## 24

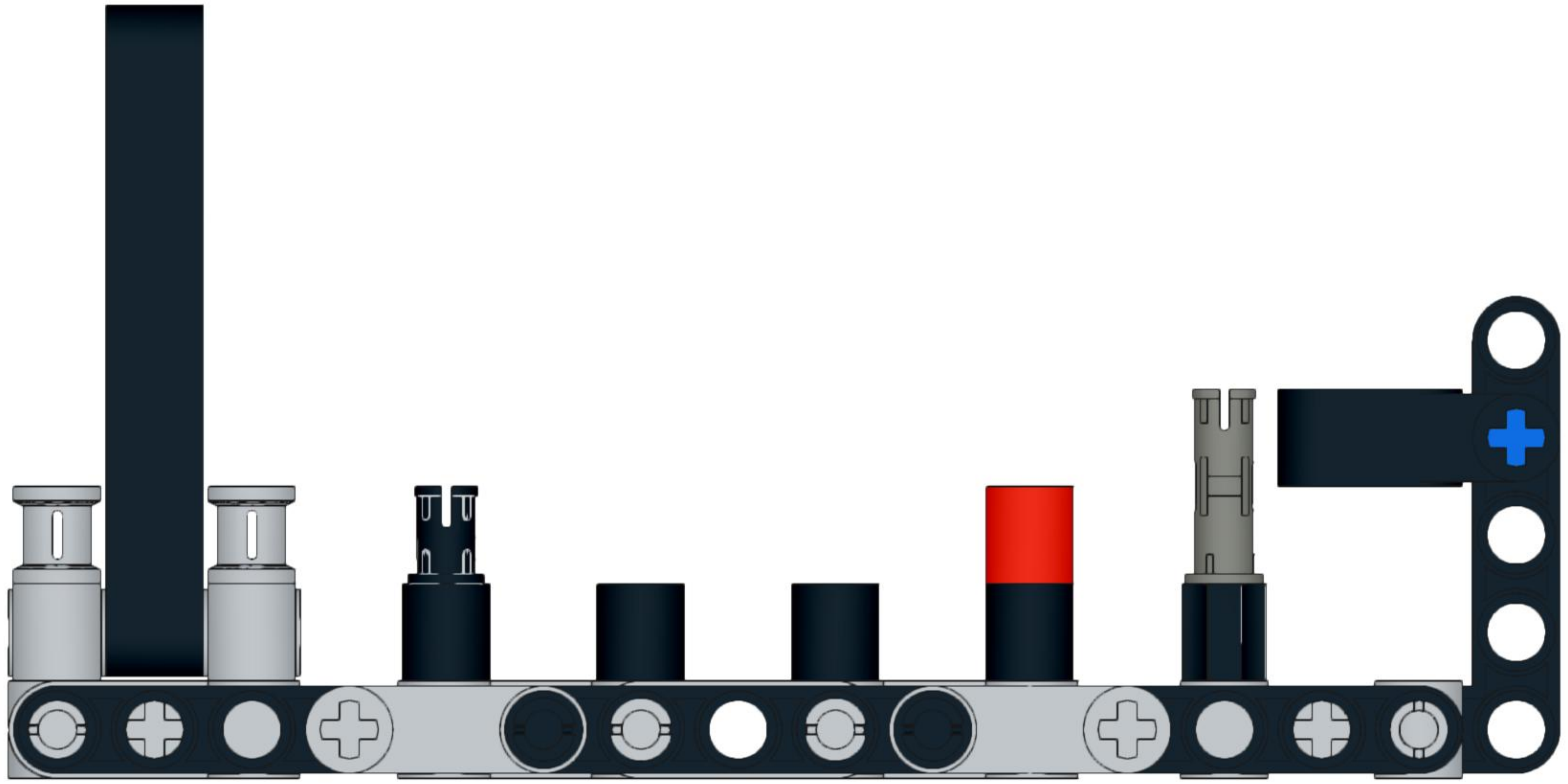

25

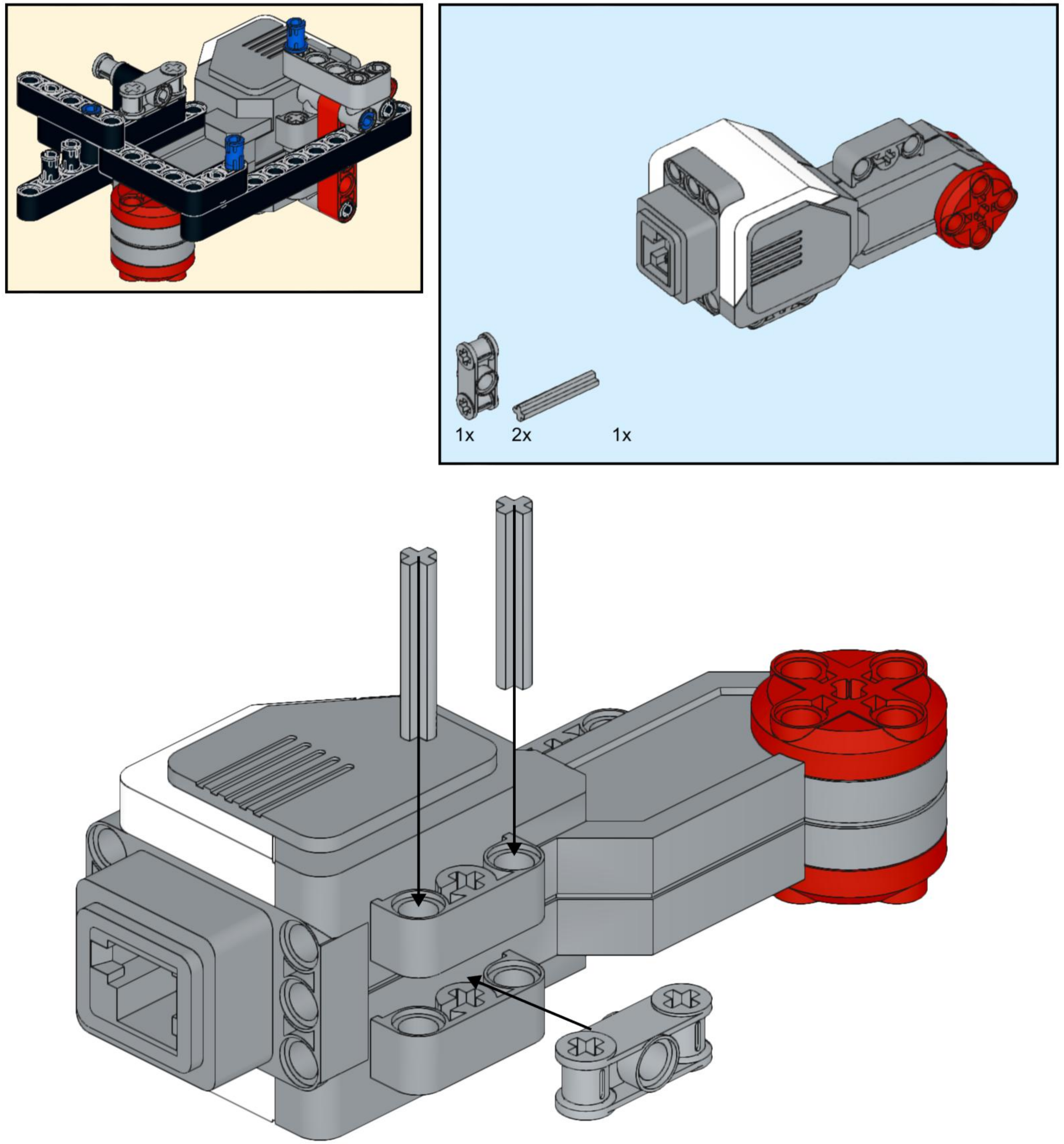


26

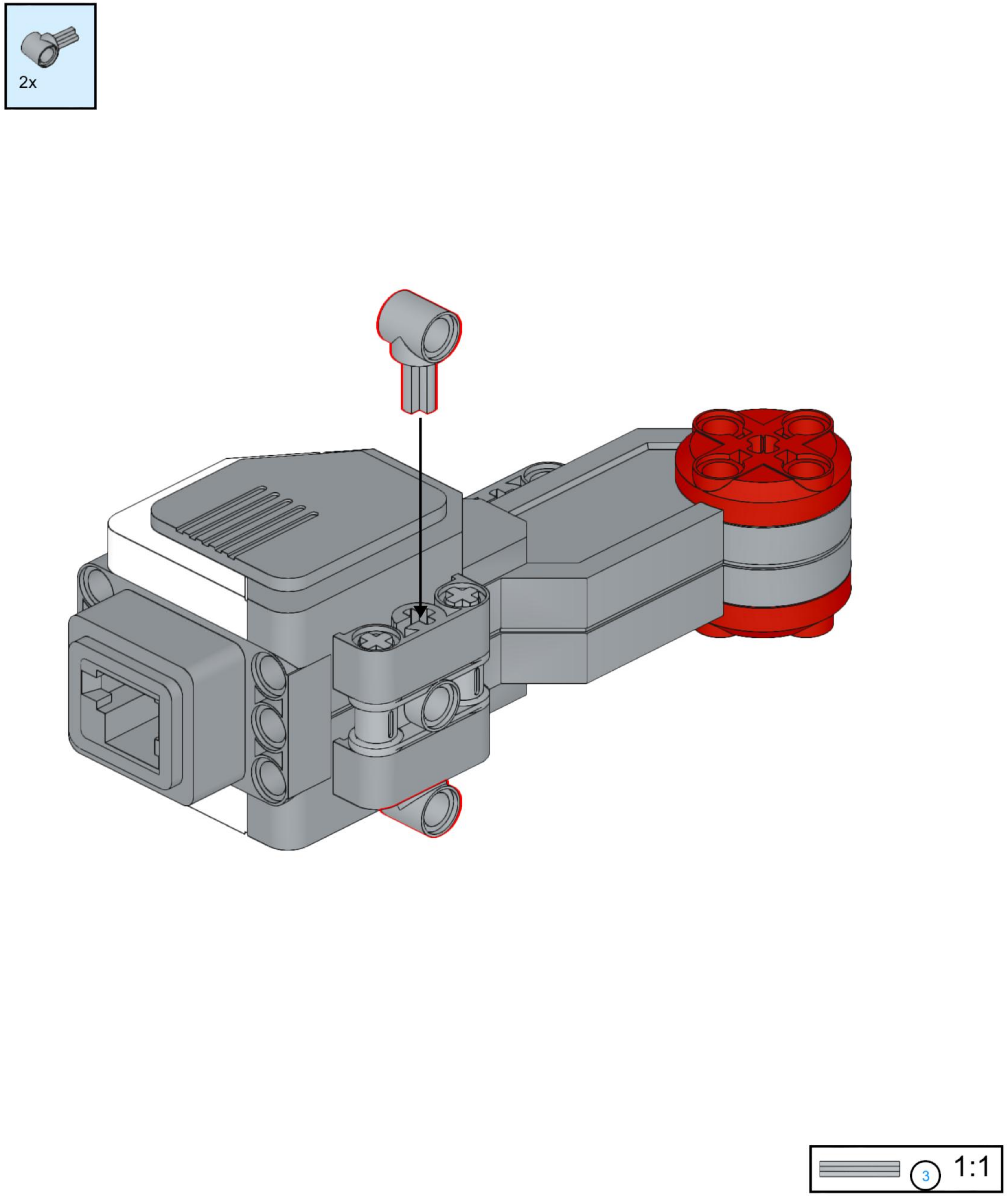

## 27

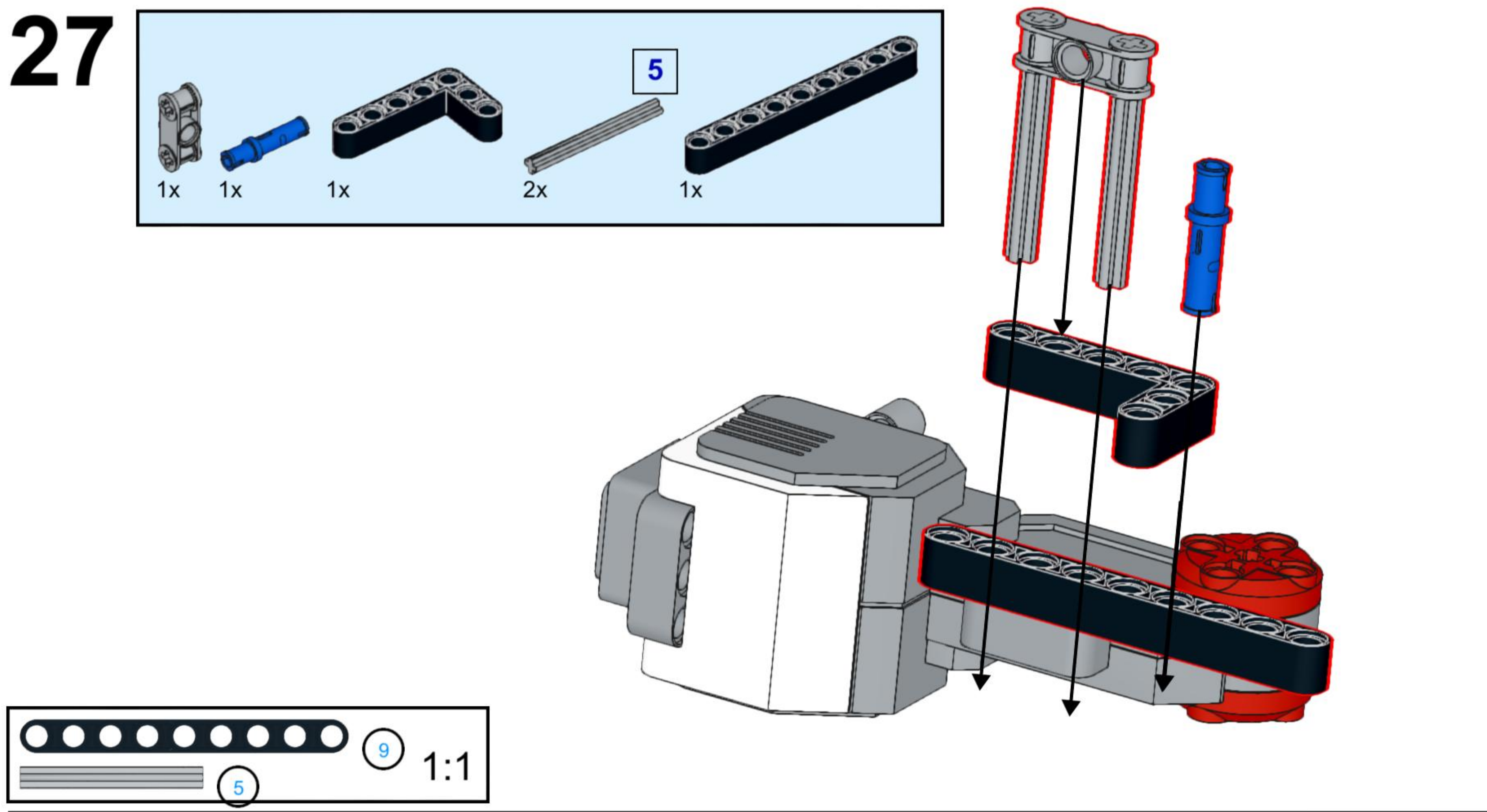


## 28

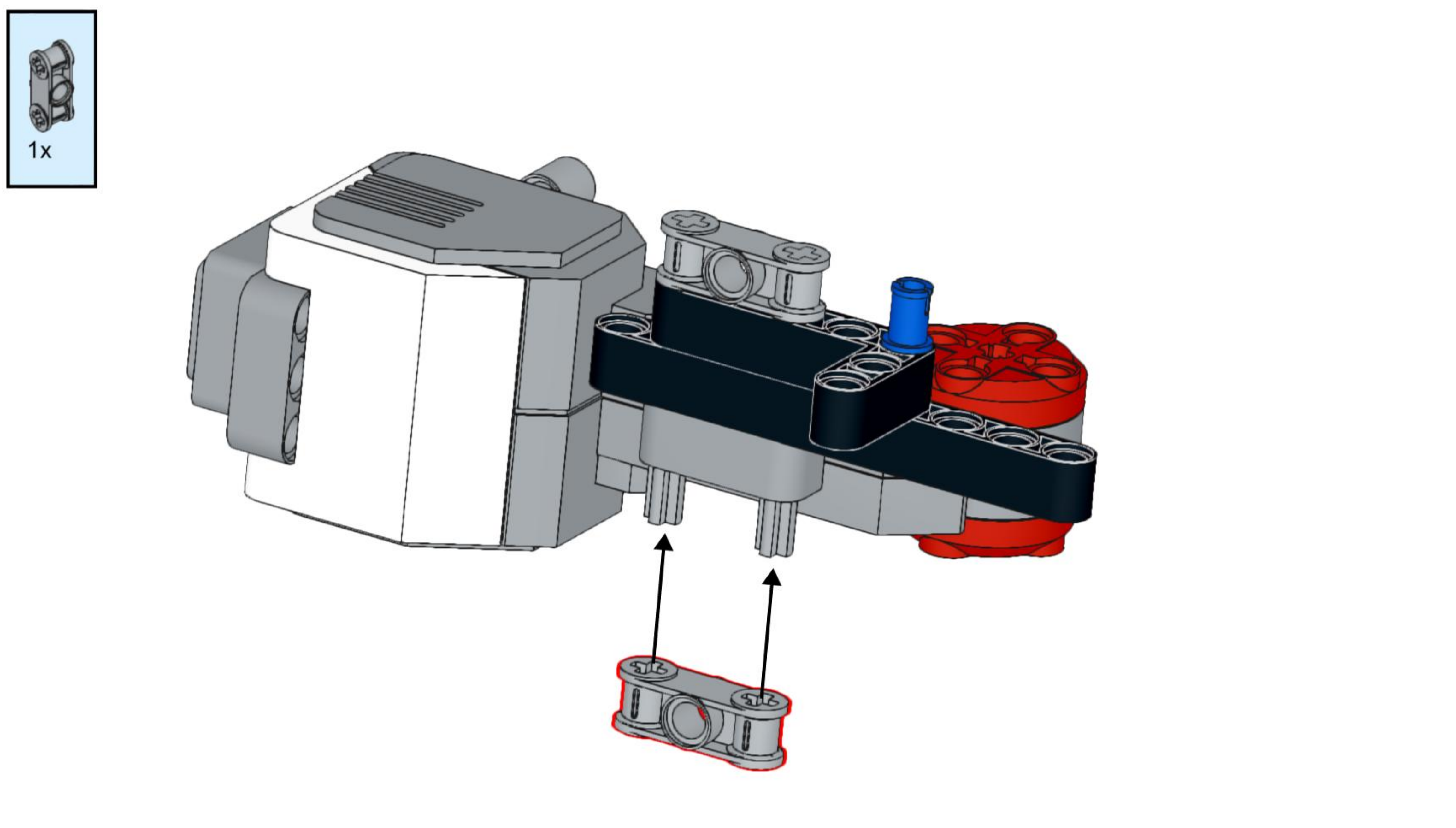


## 29

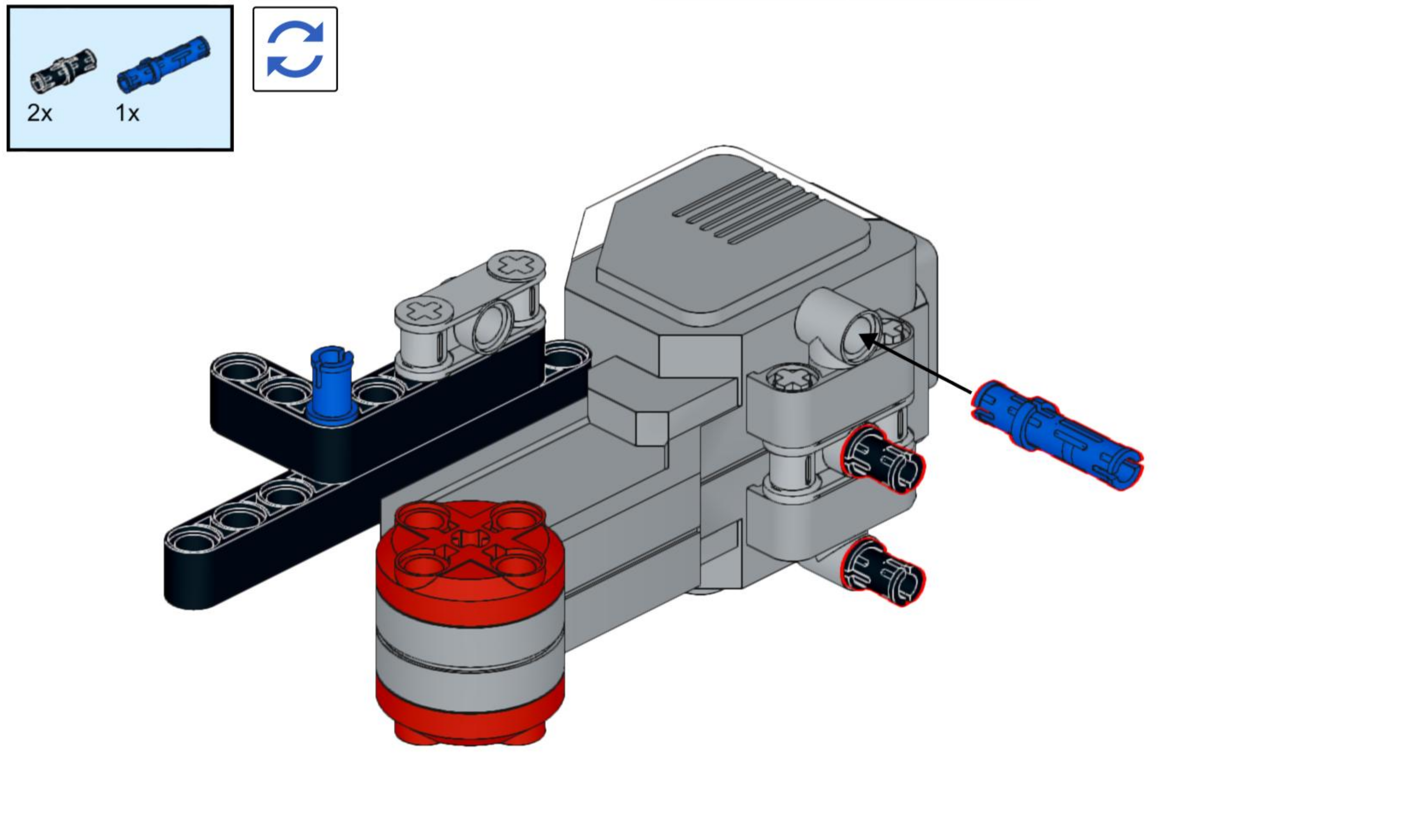


## 30

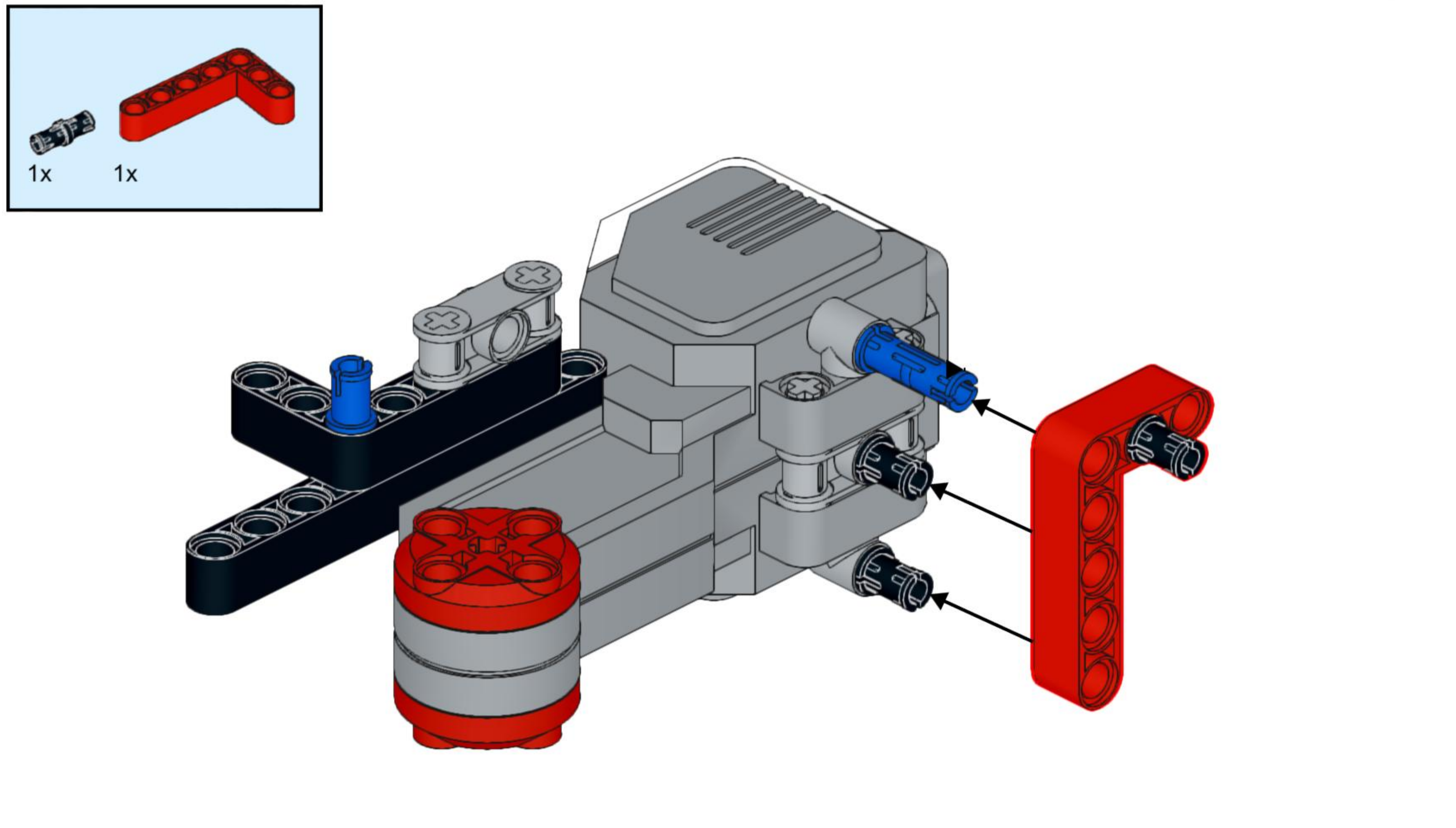

# 31

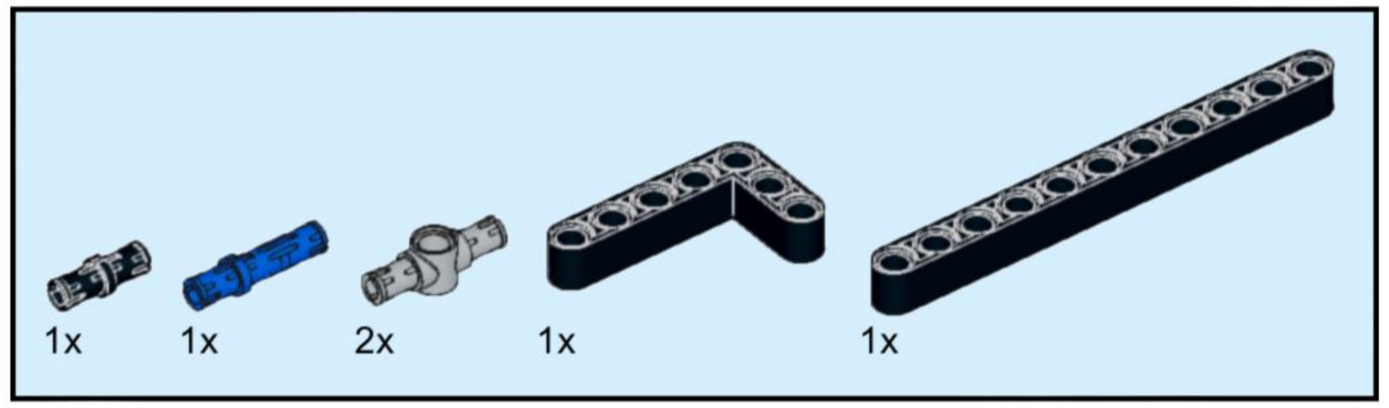


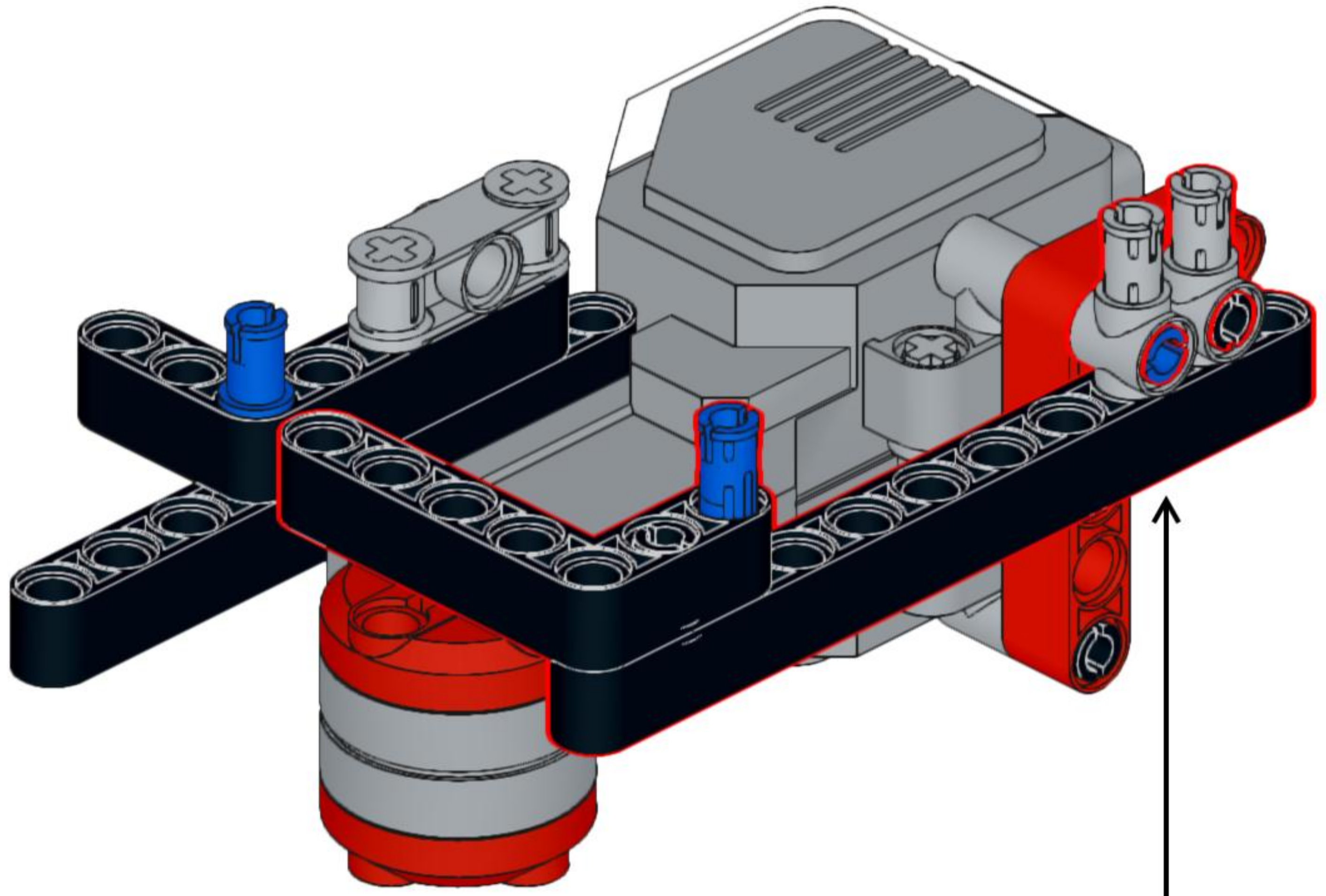

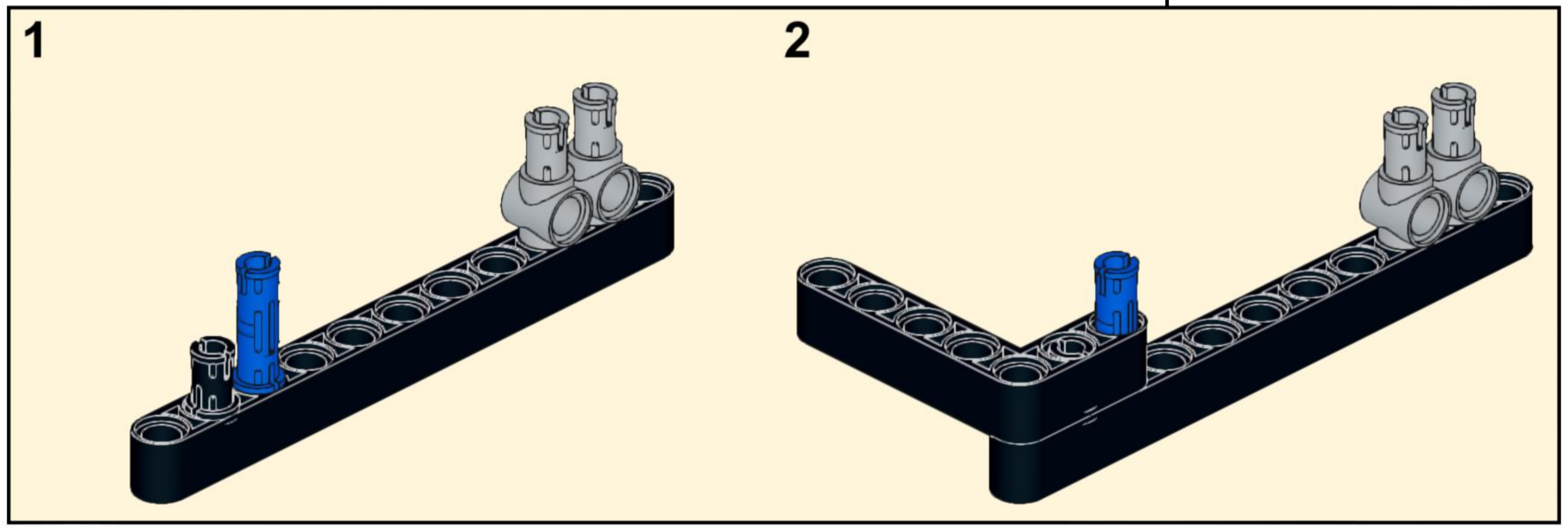


# 32

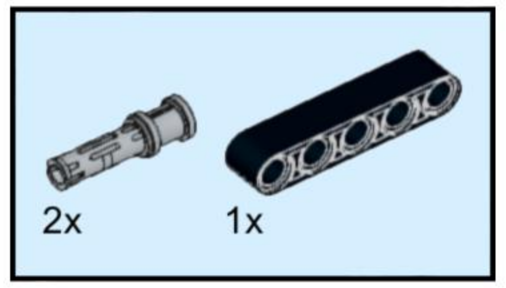


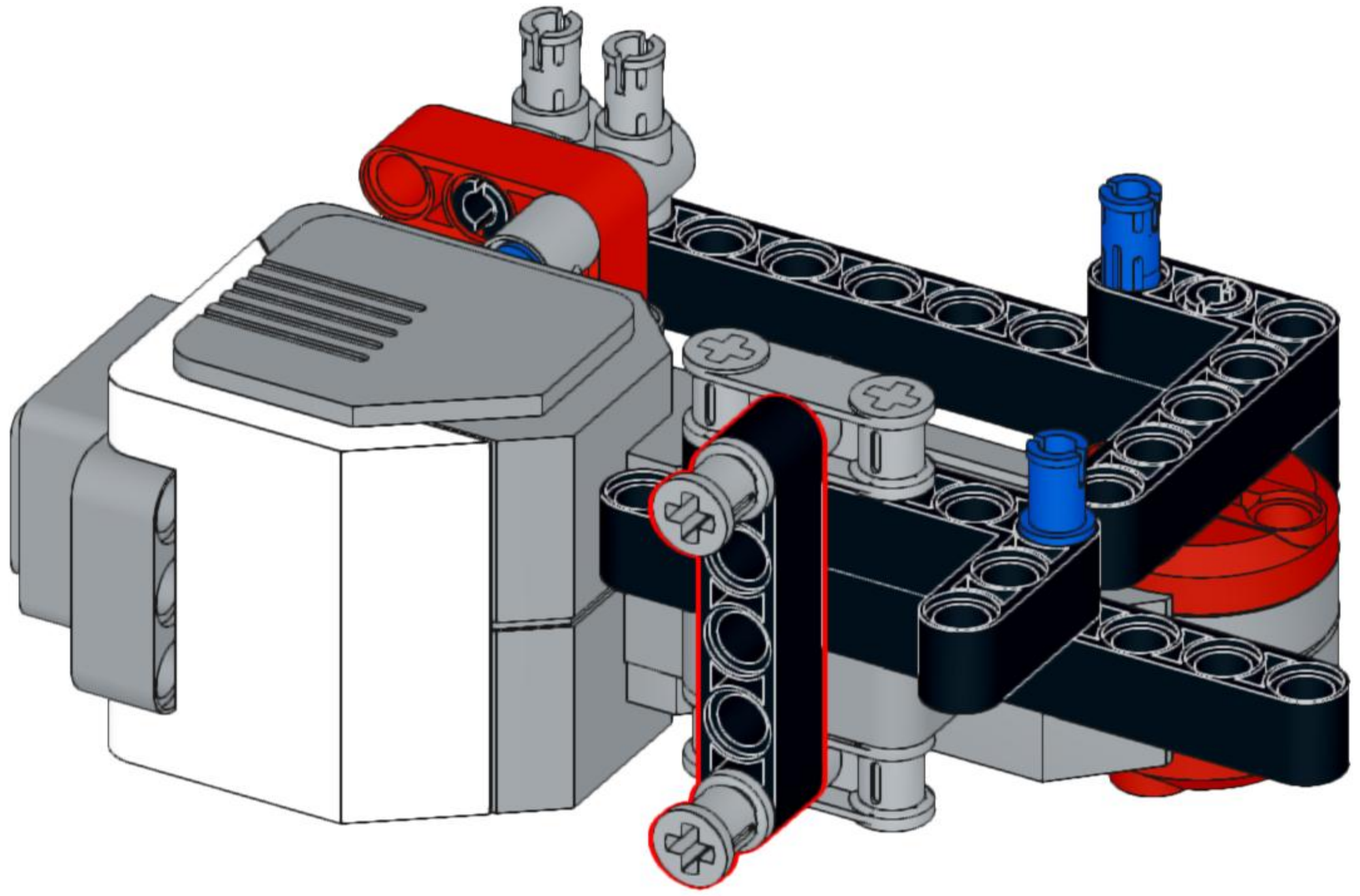

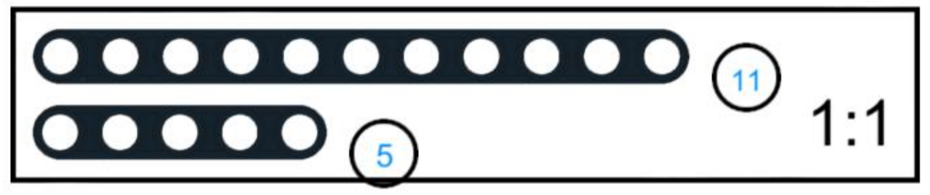

# 33

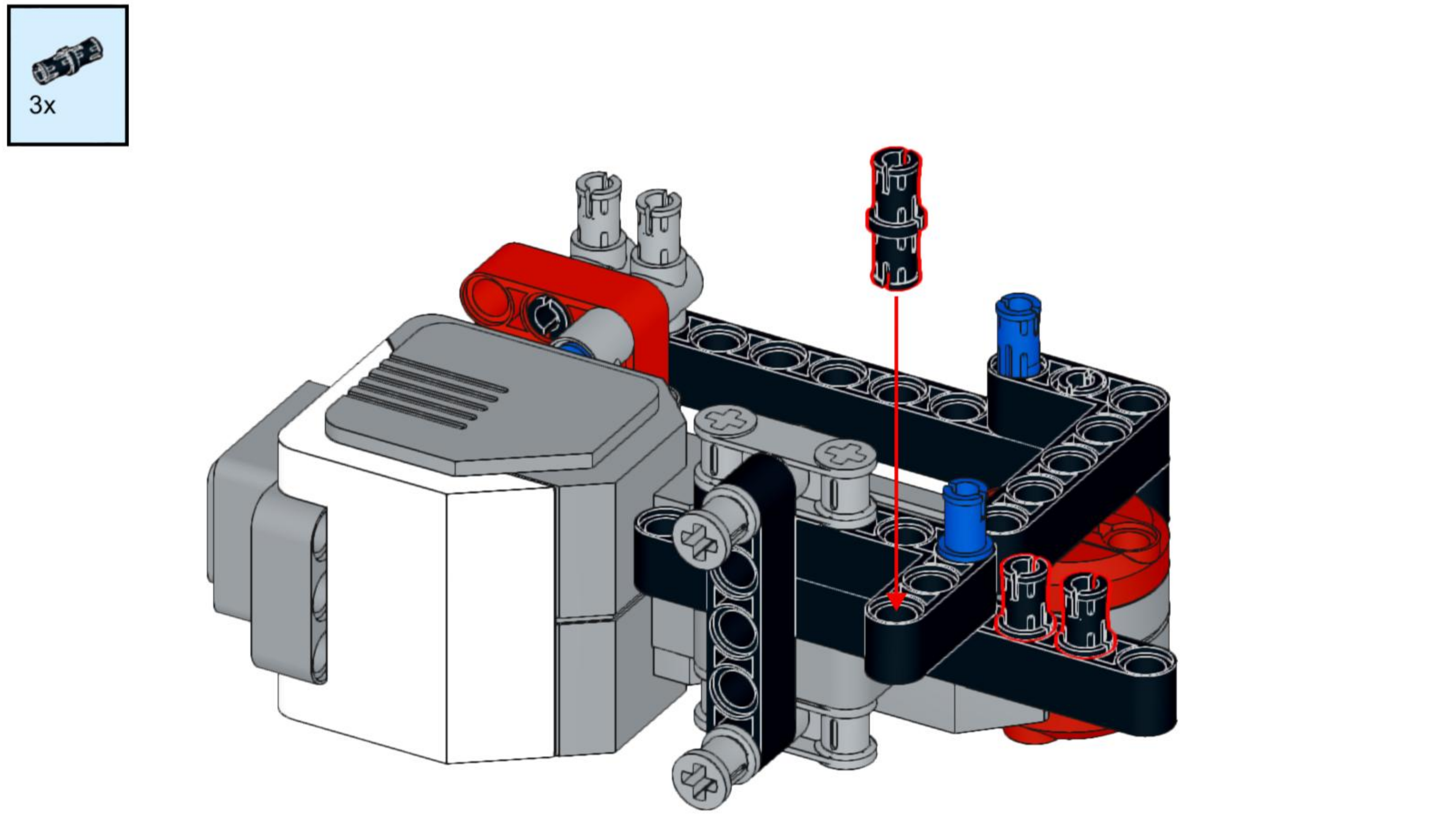


# 34

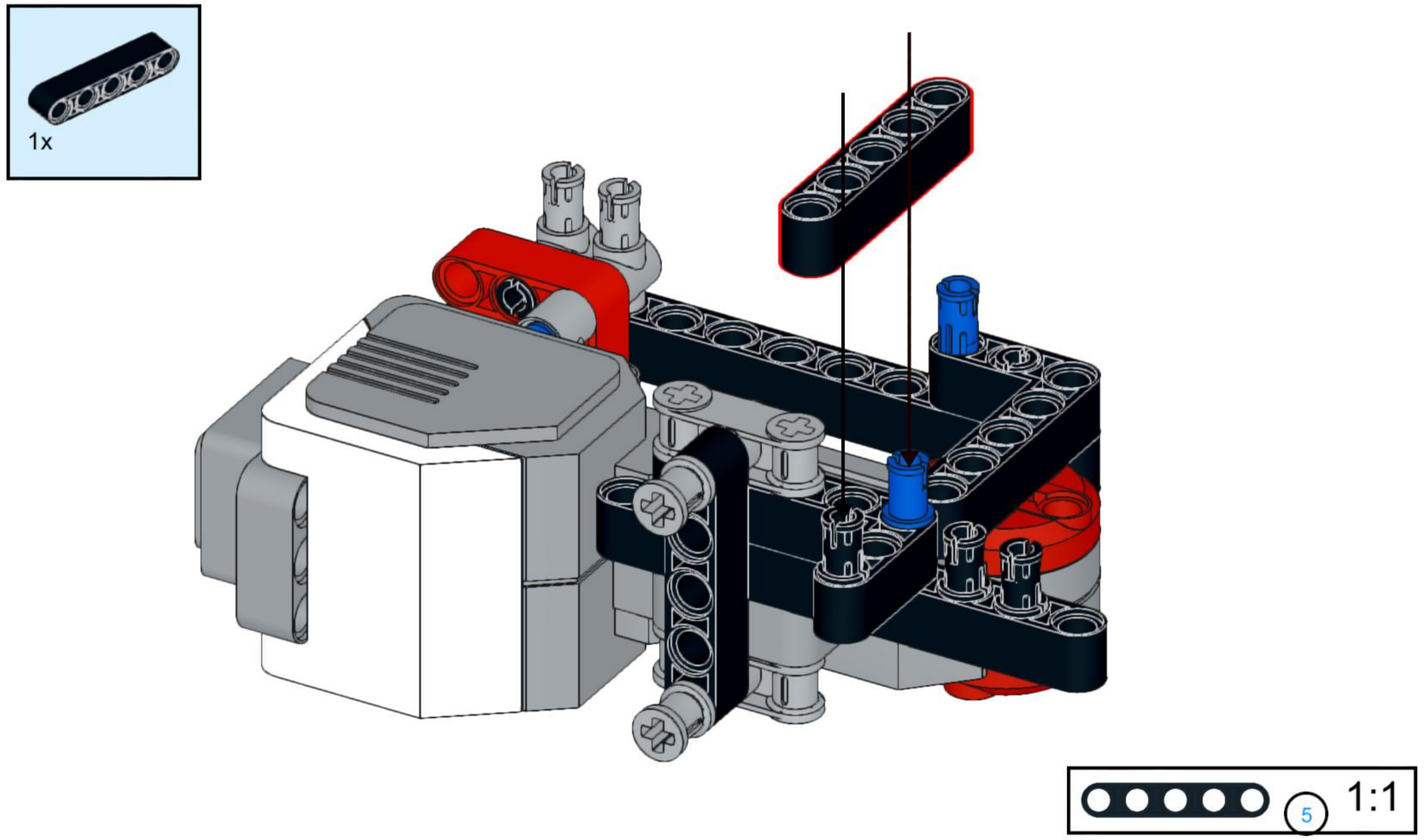


# 35

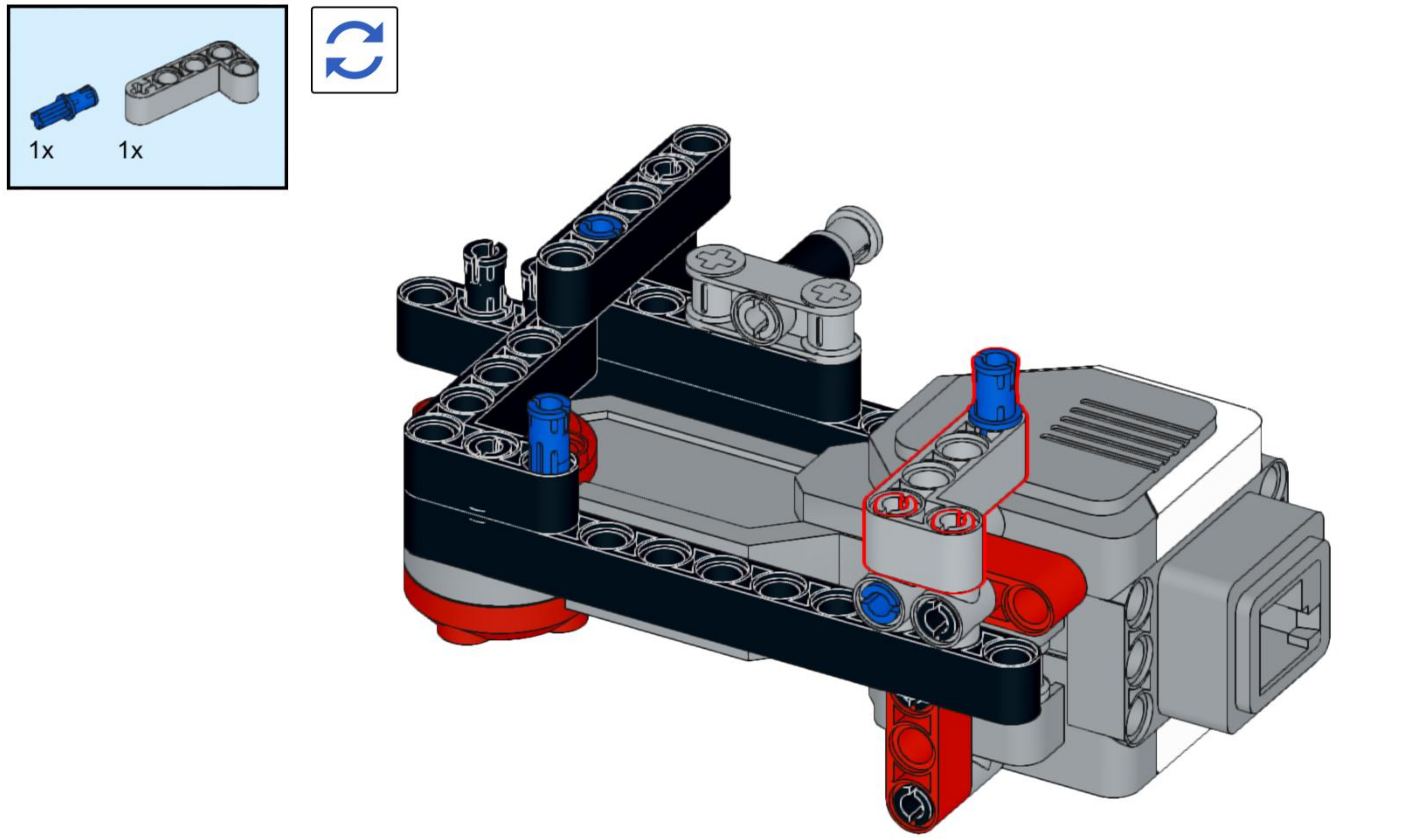


# 36

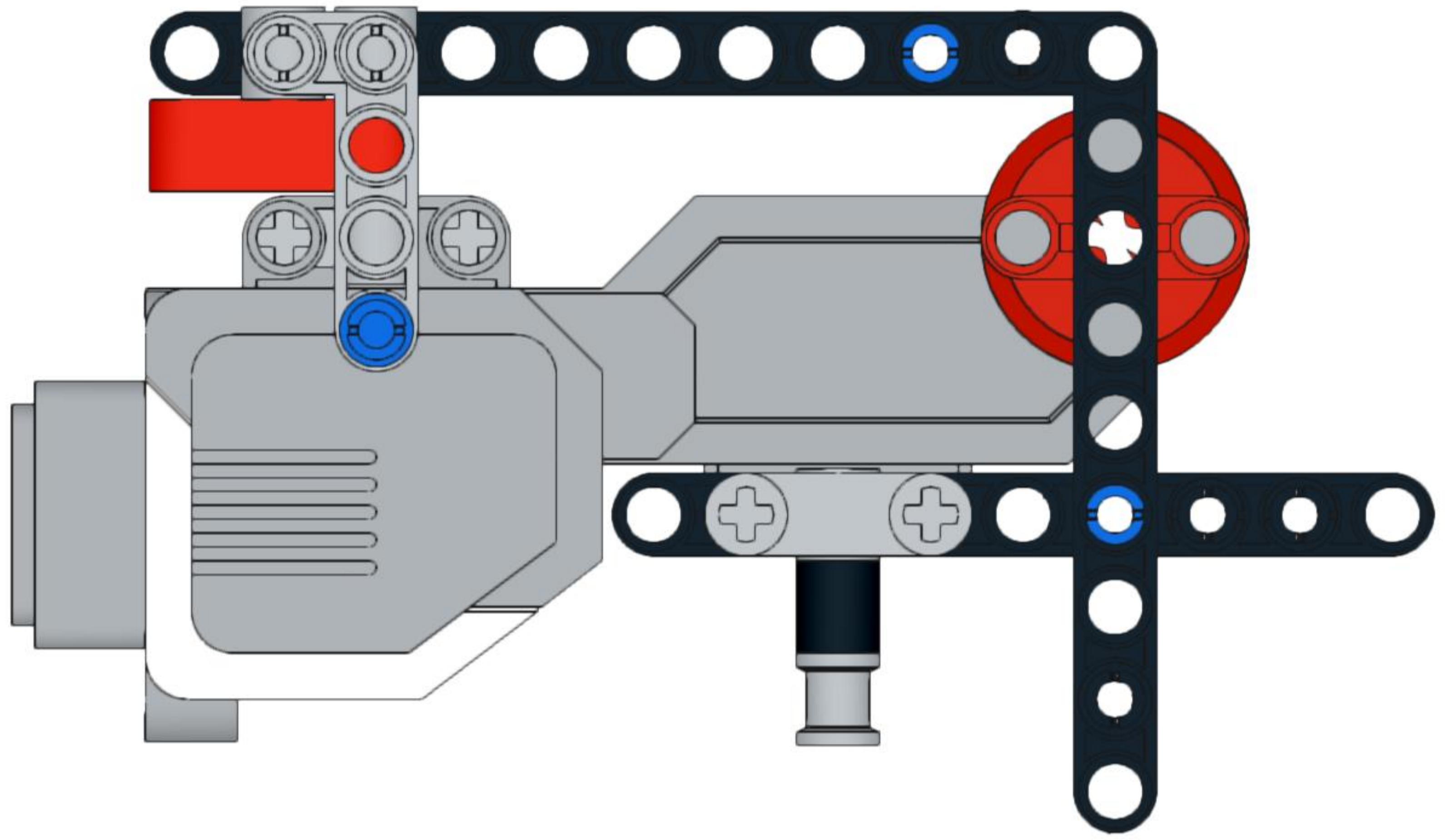

37

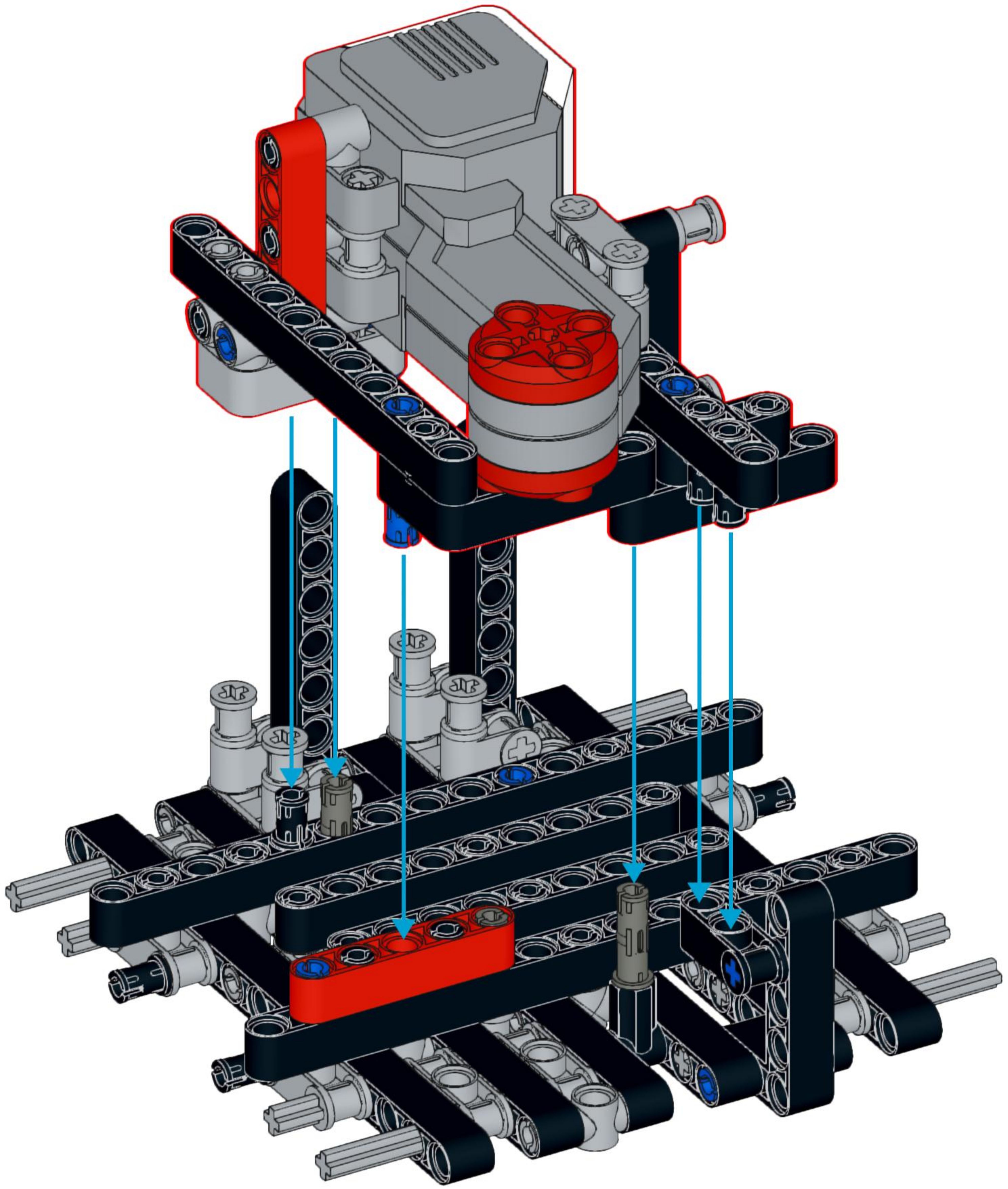

38

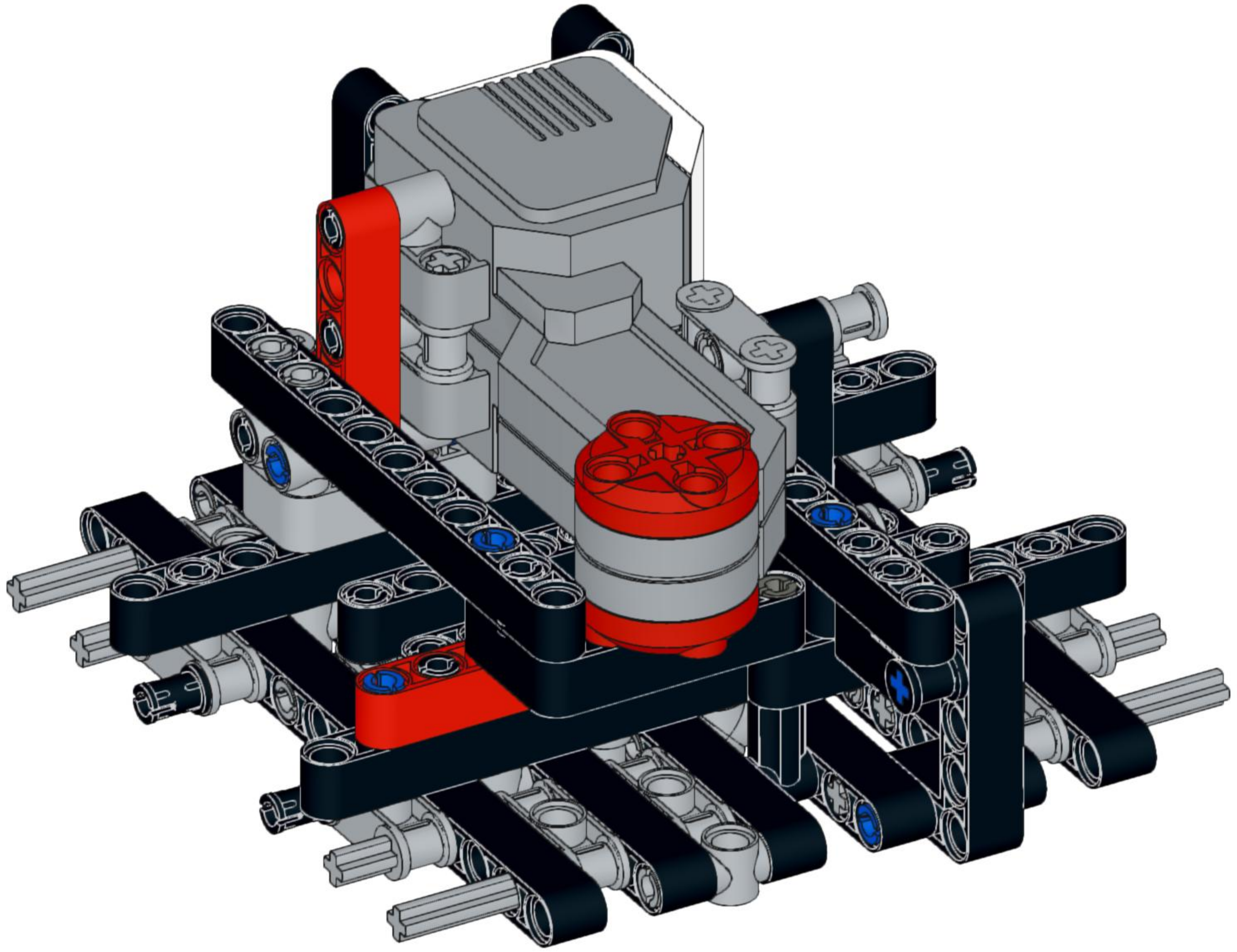

39

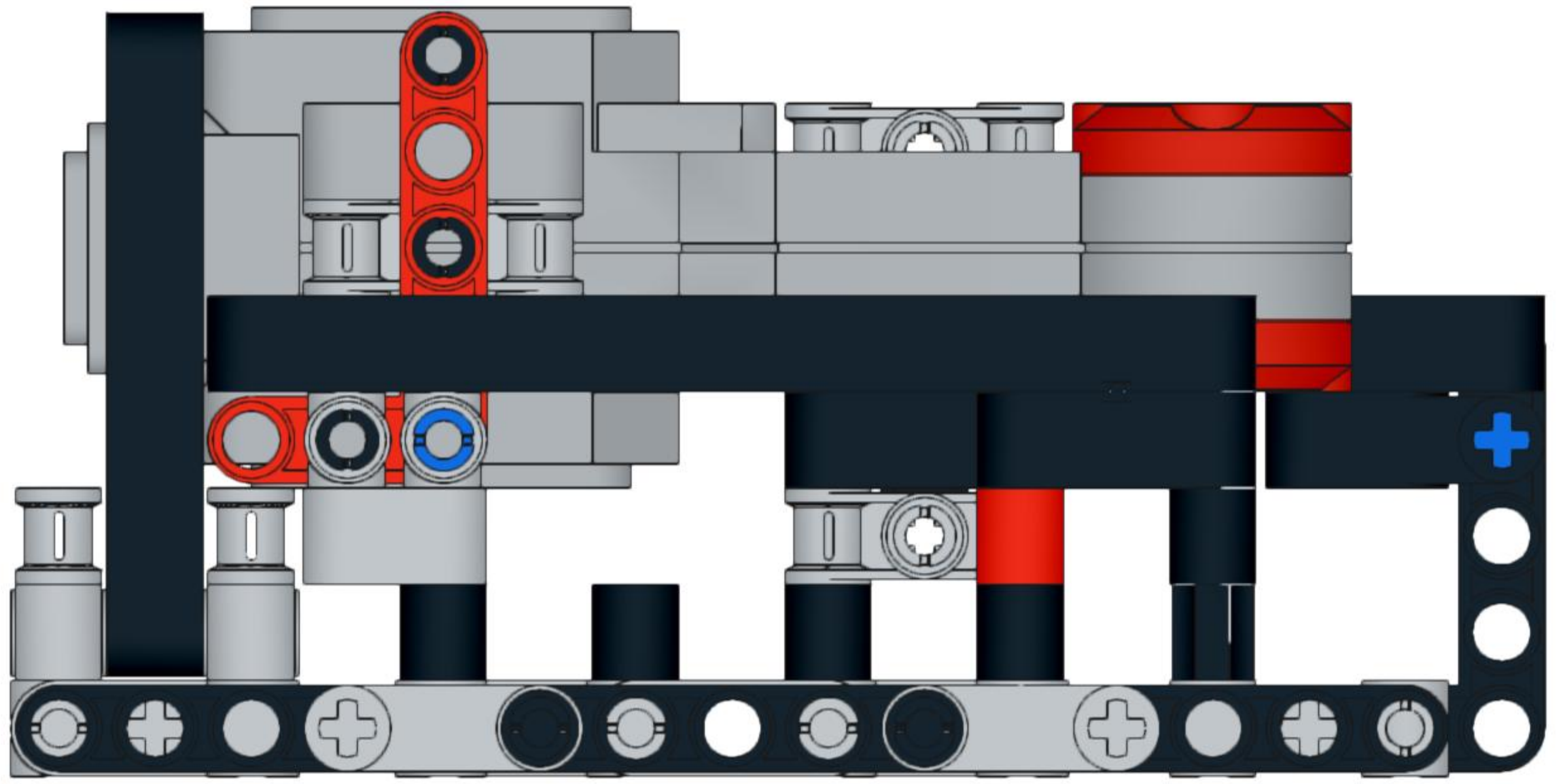

40

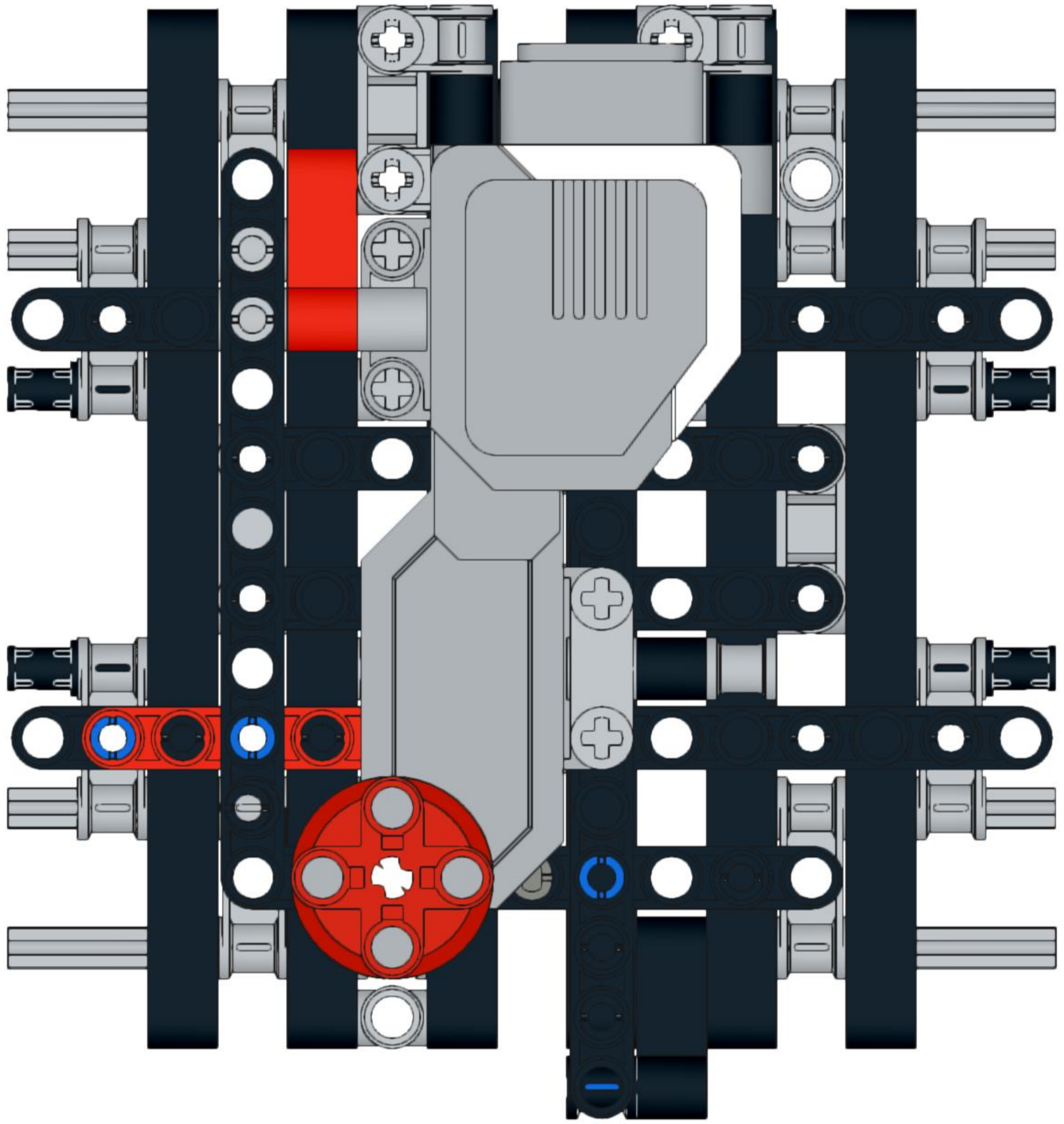

## 41

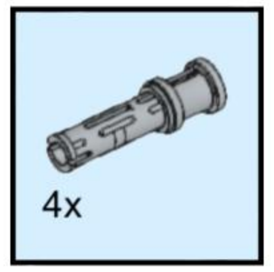


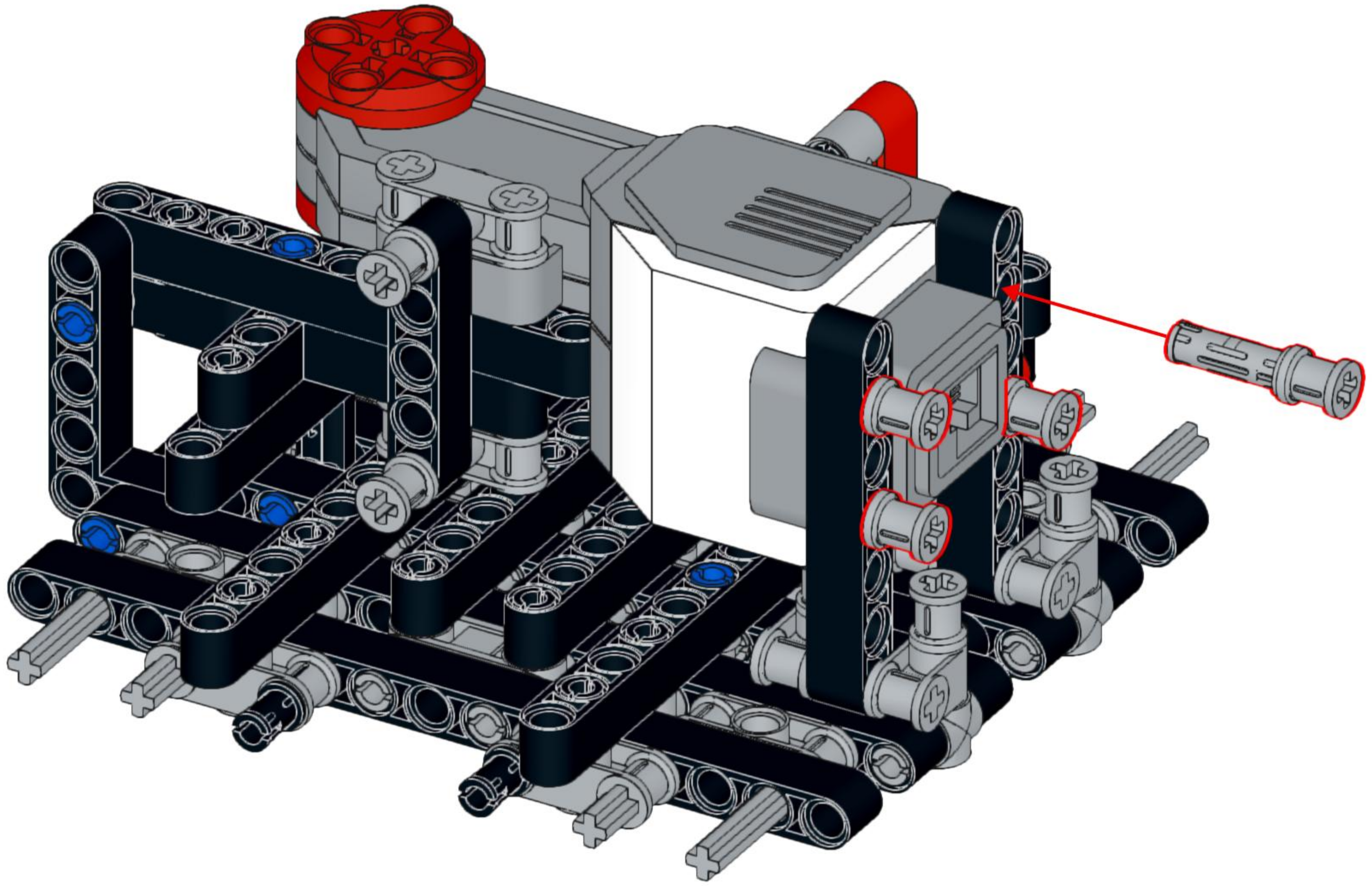

## 42

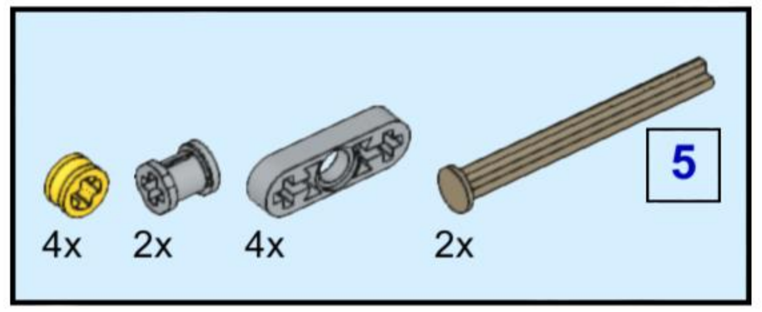


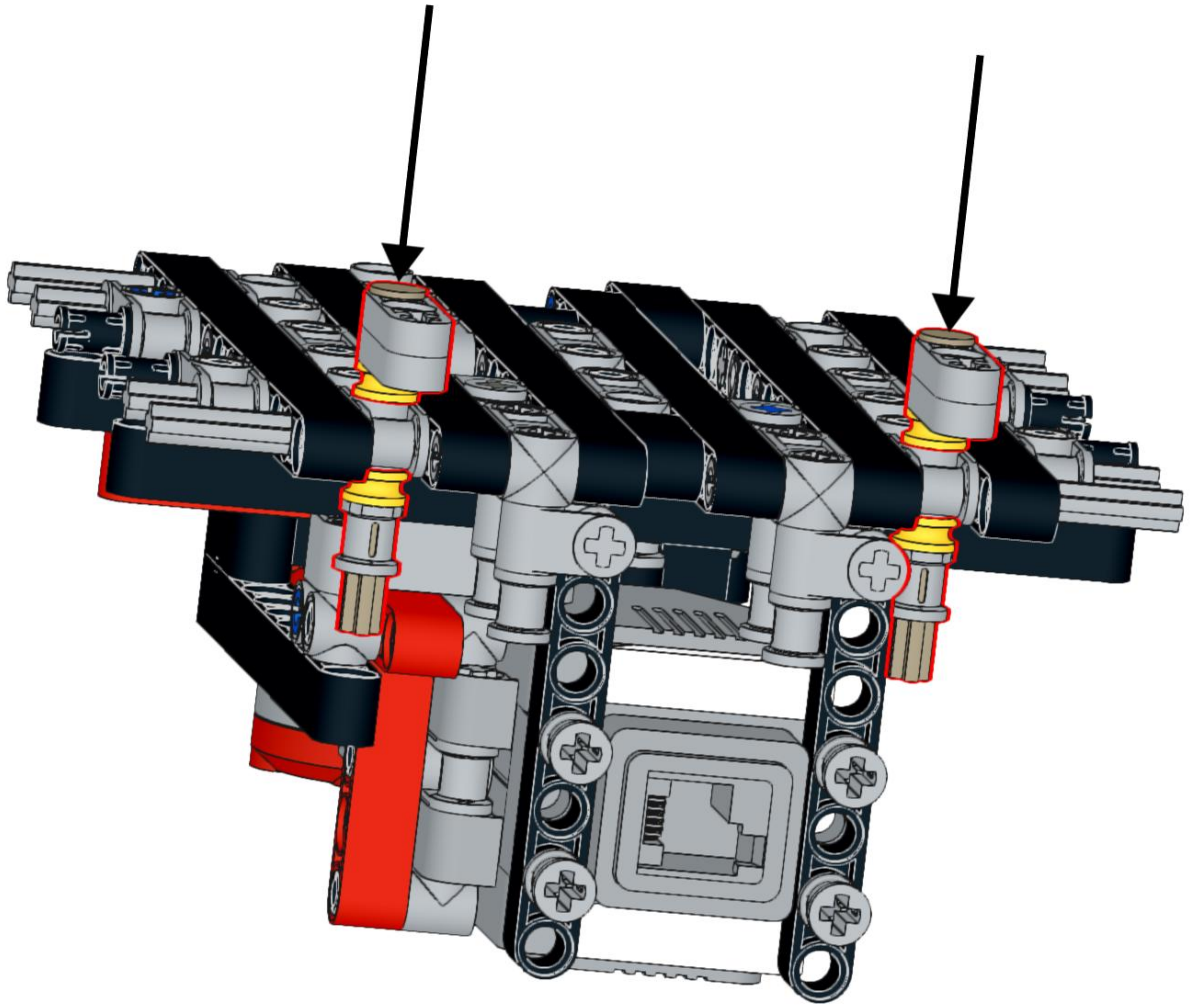

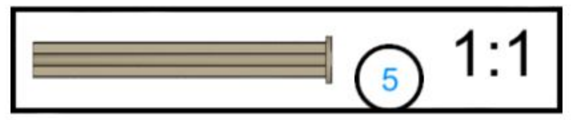

## 43

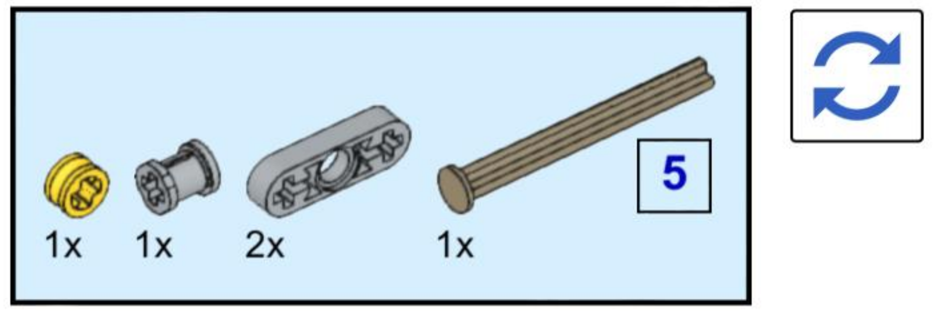


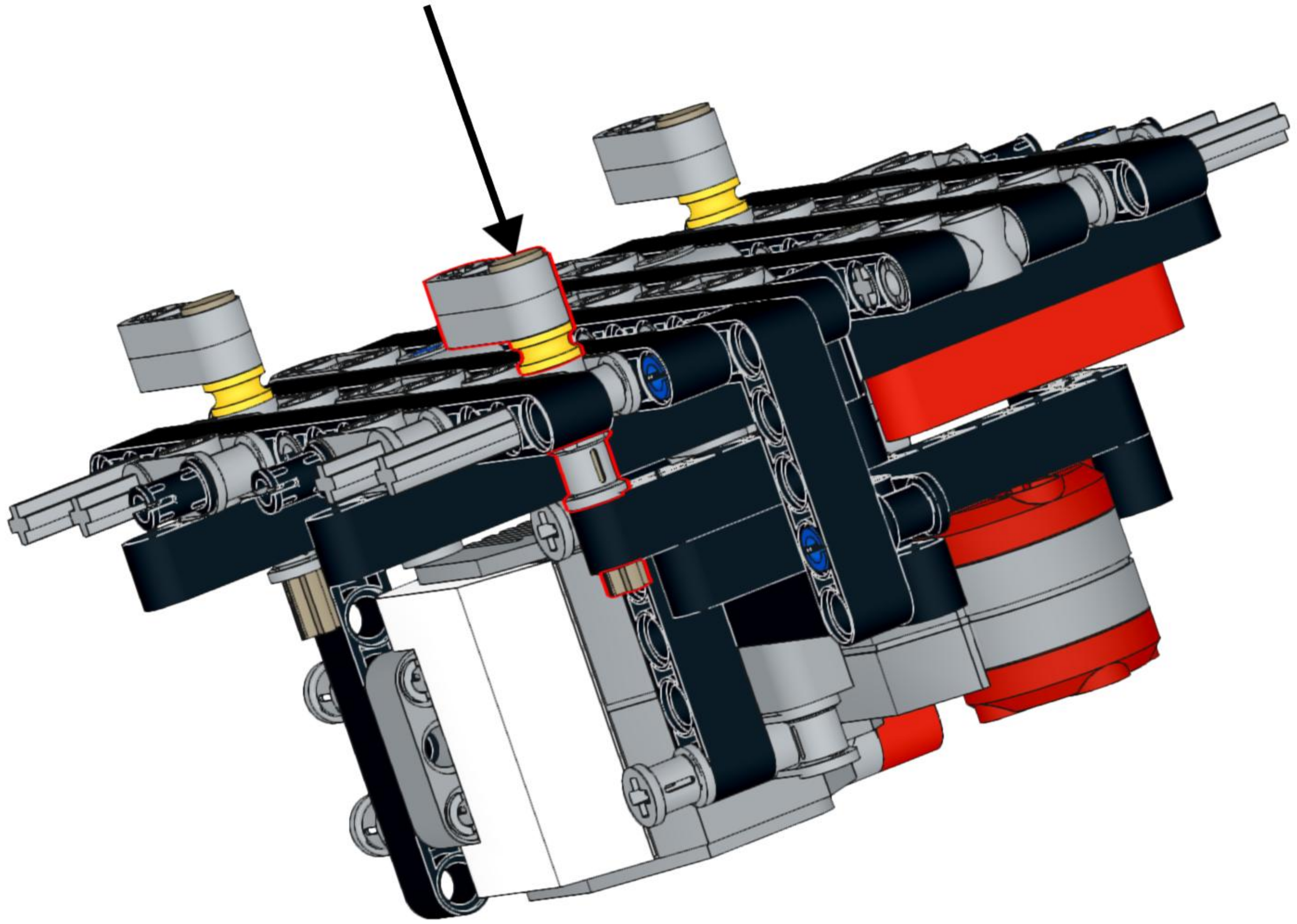

## 44

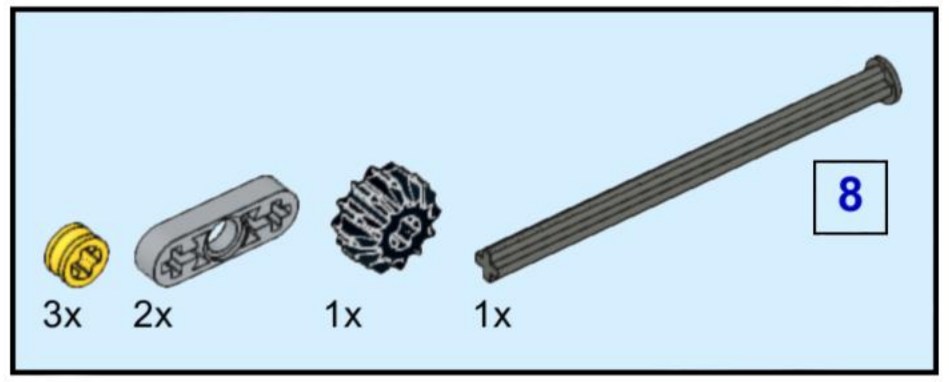


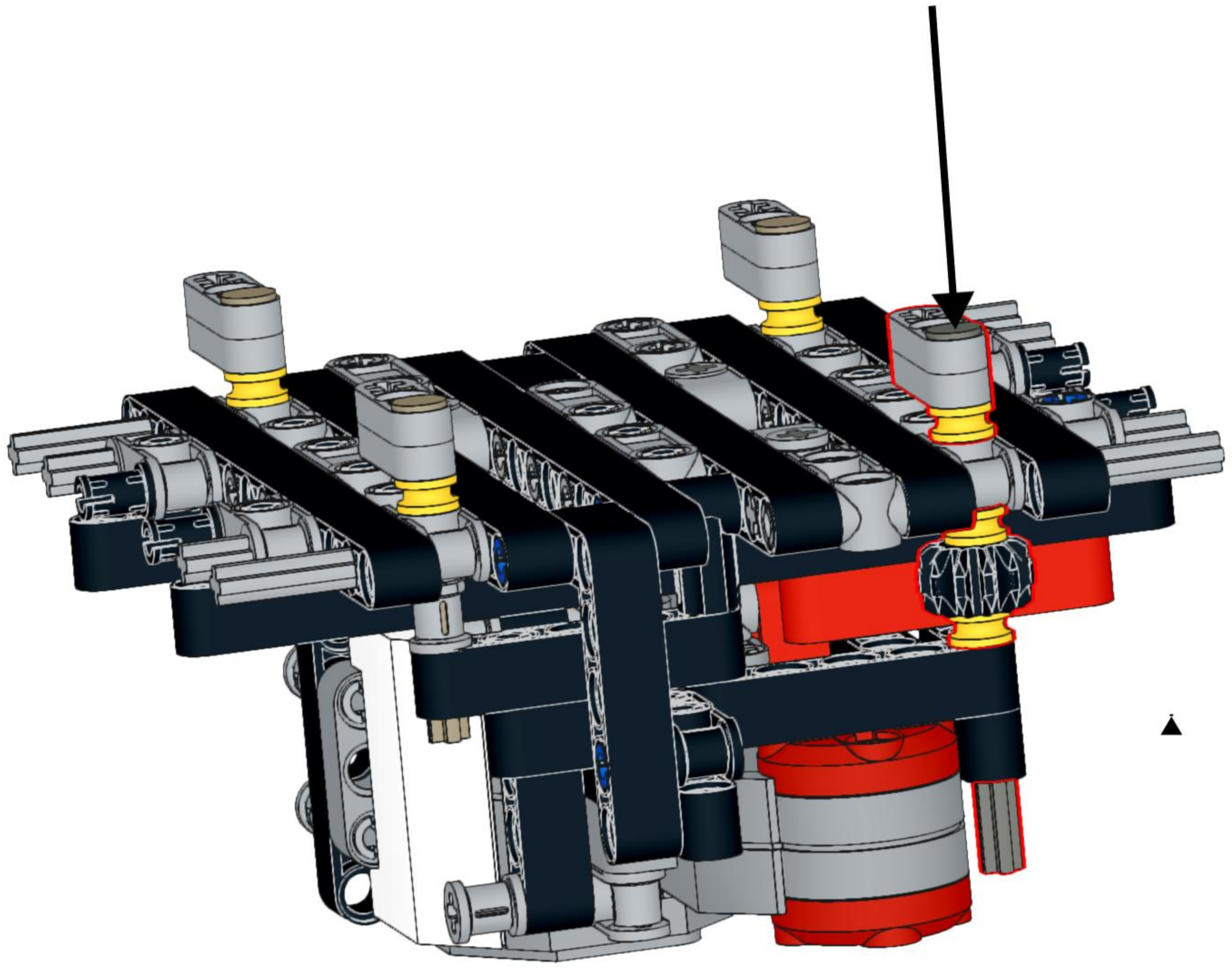

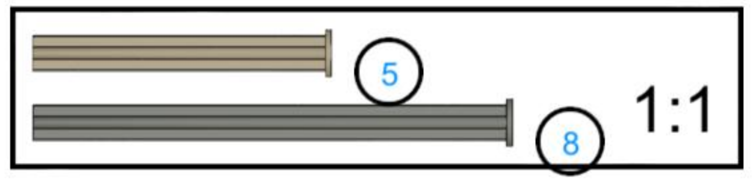

# 45

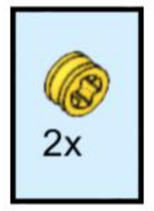


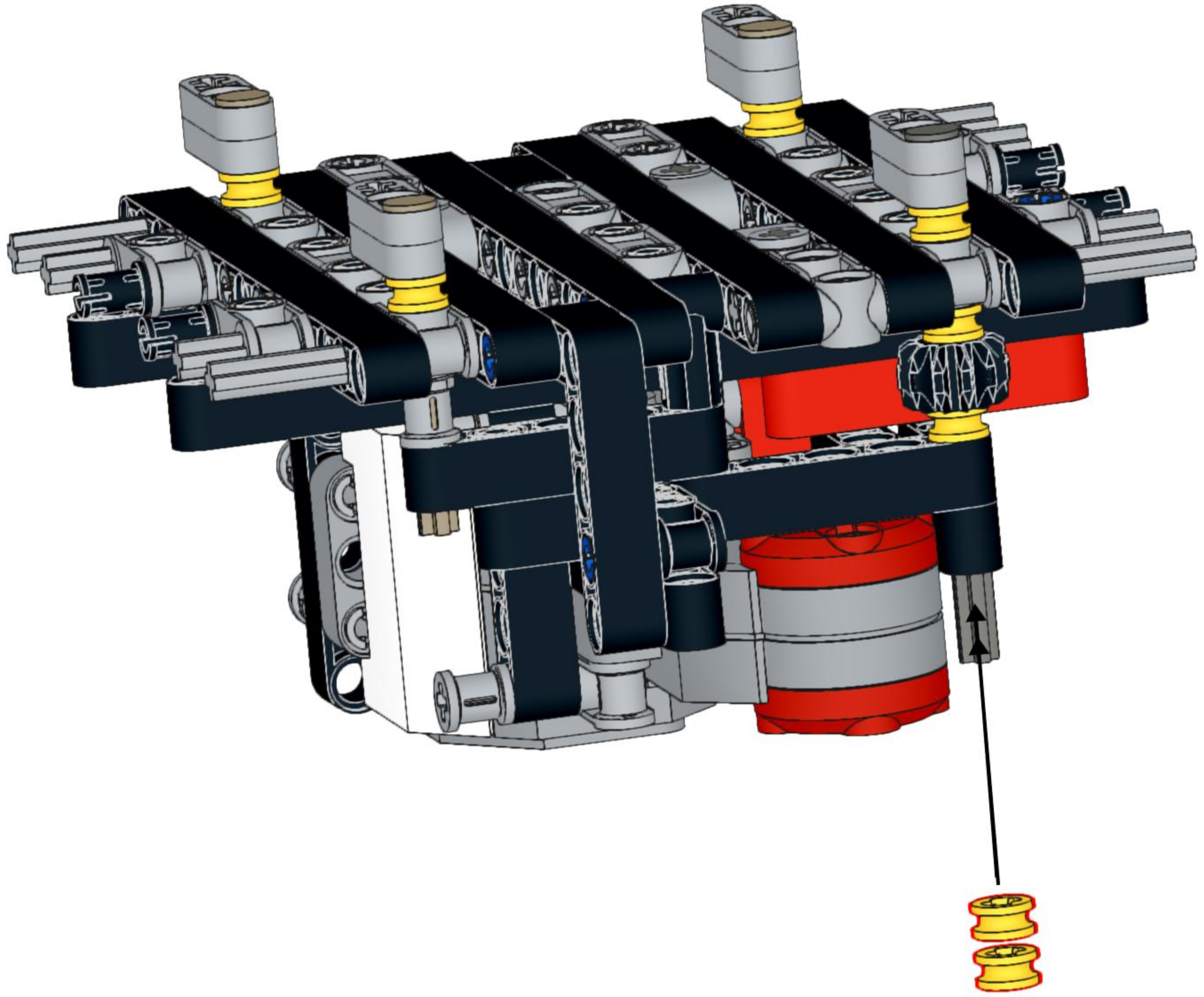

# 46

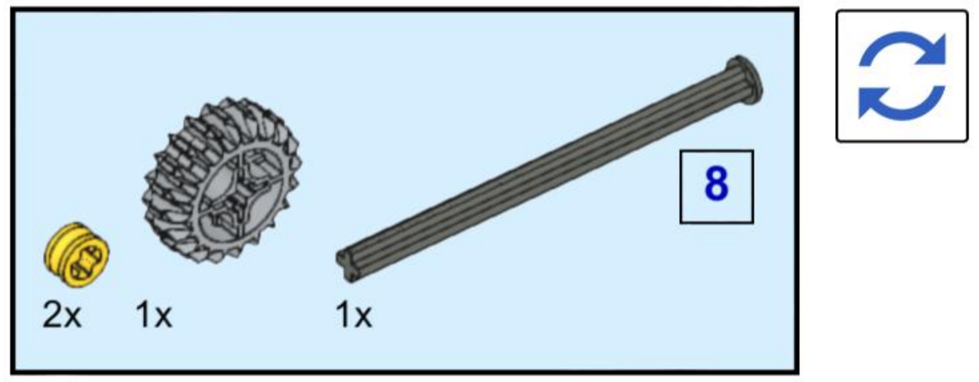


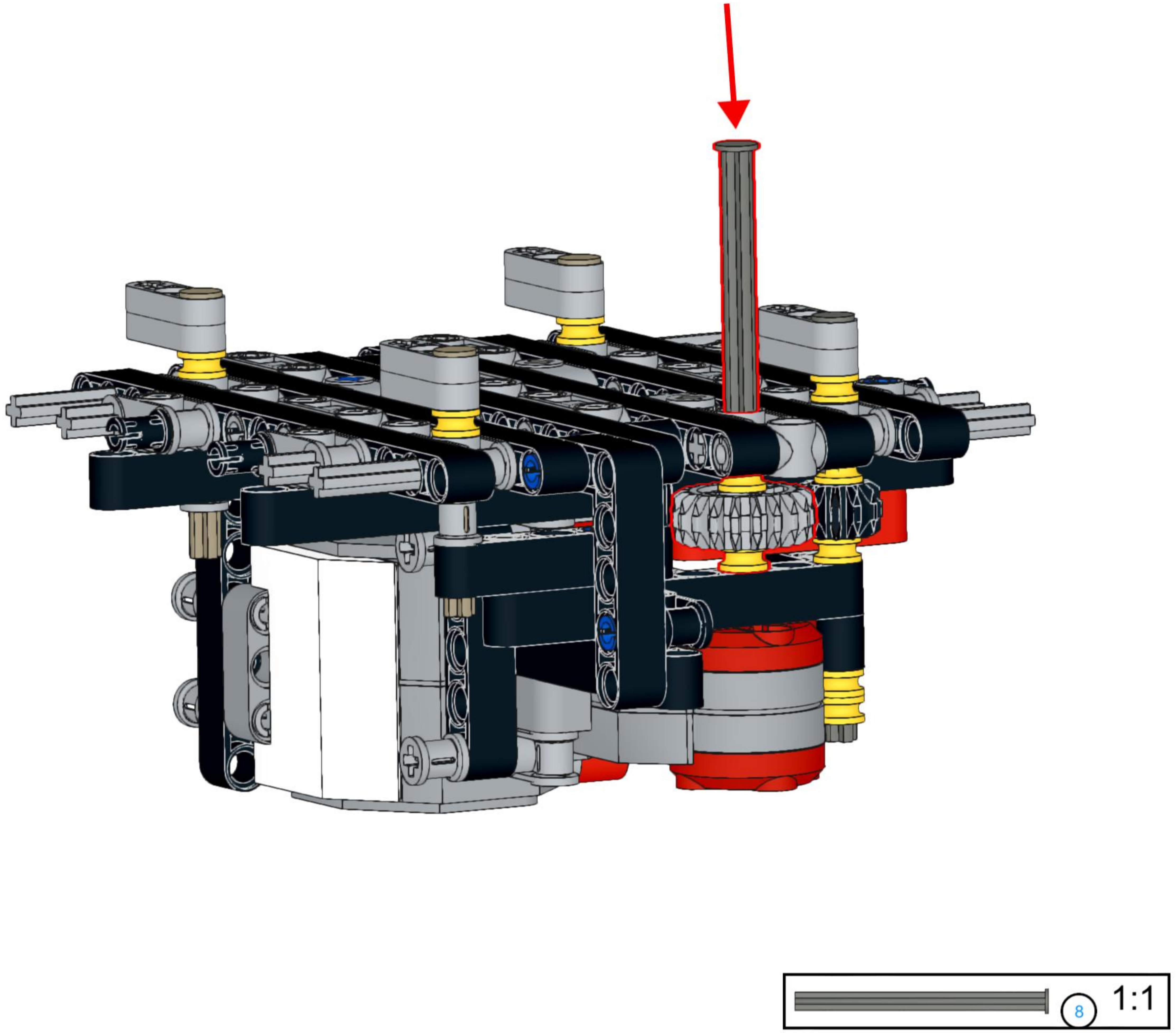

47

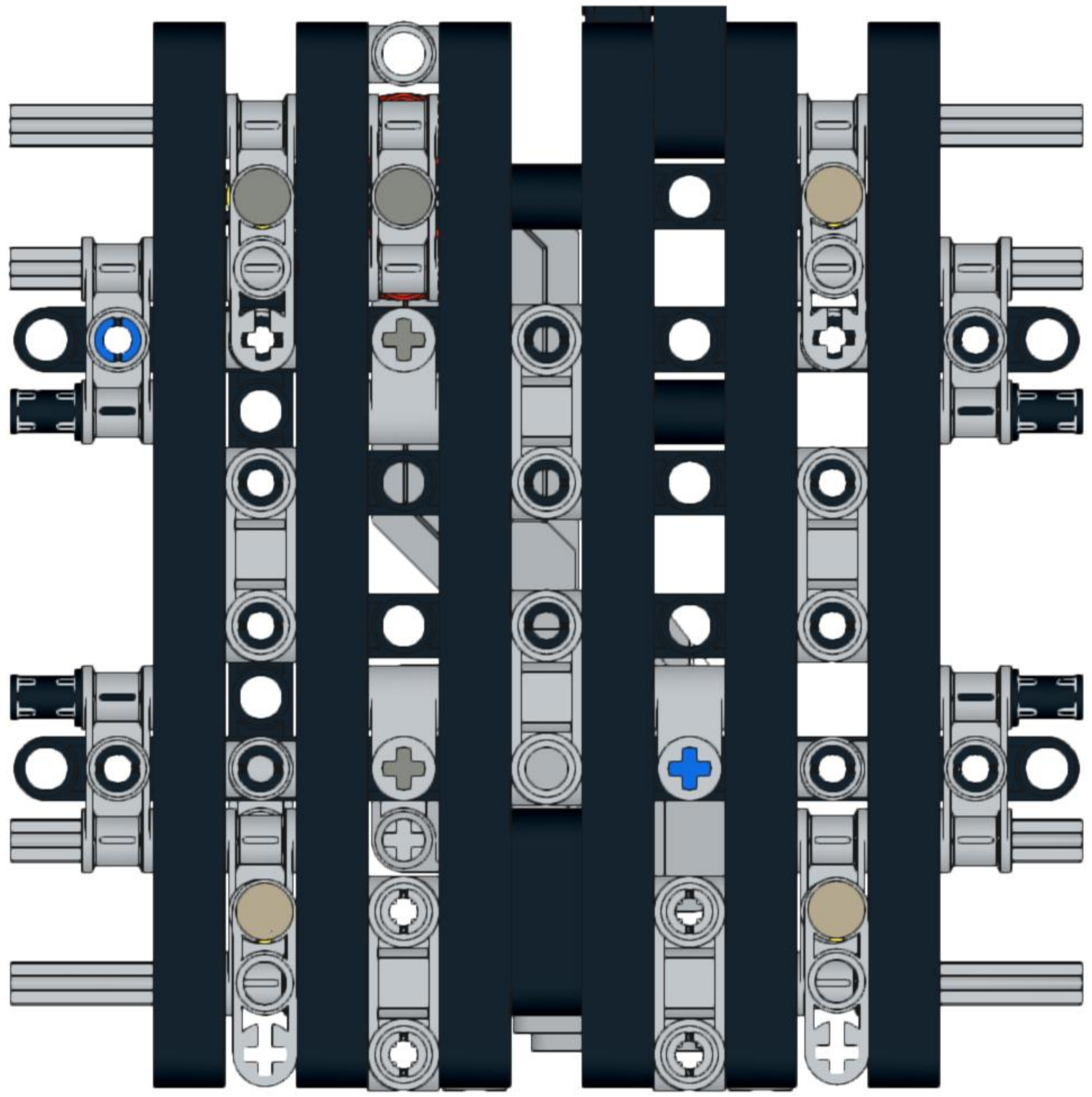

48

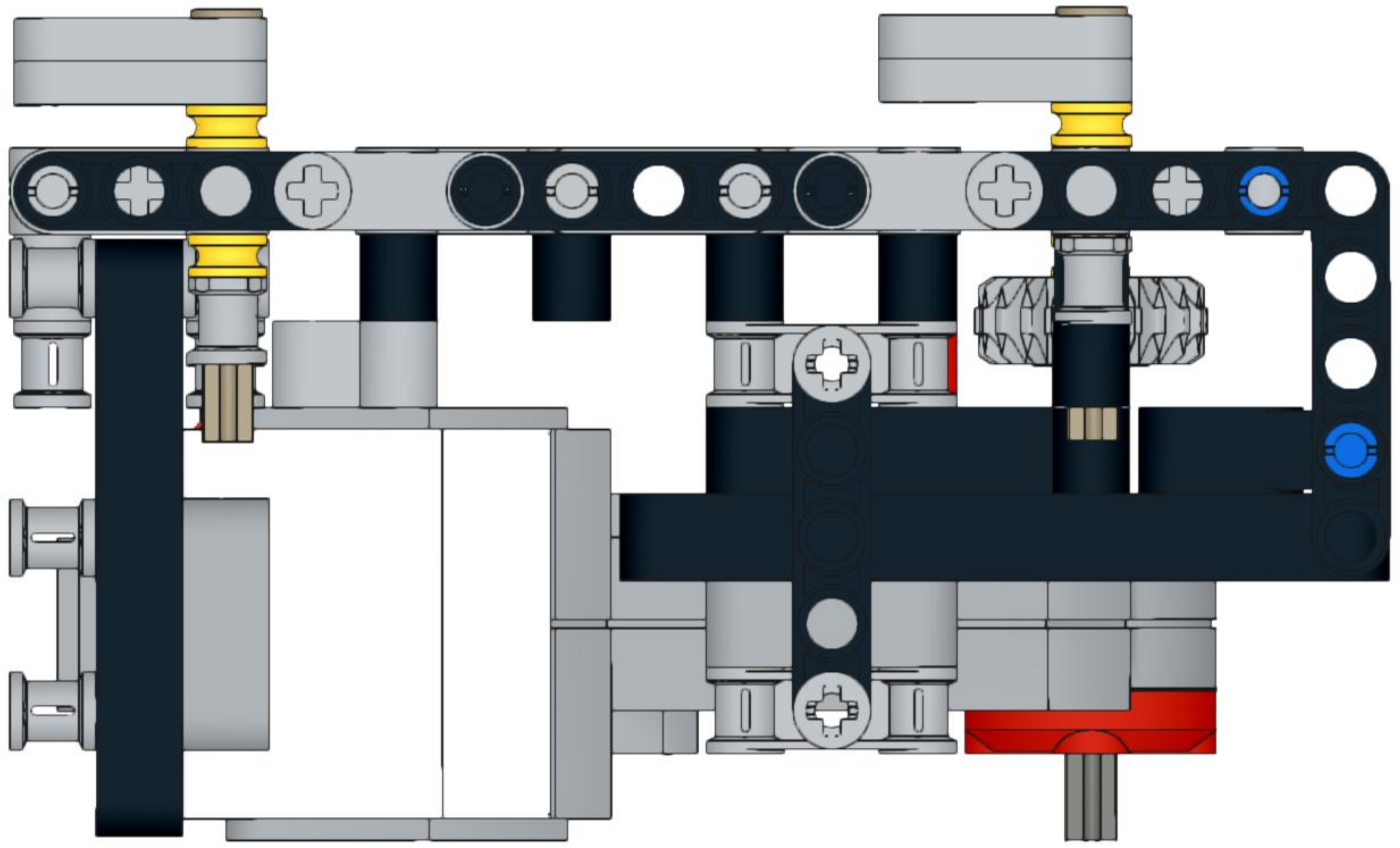

49

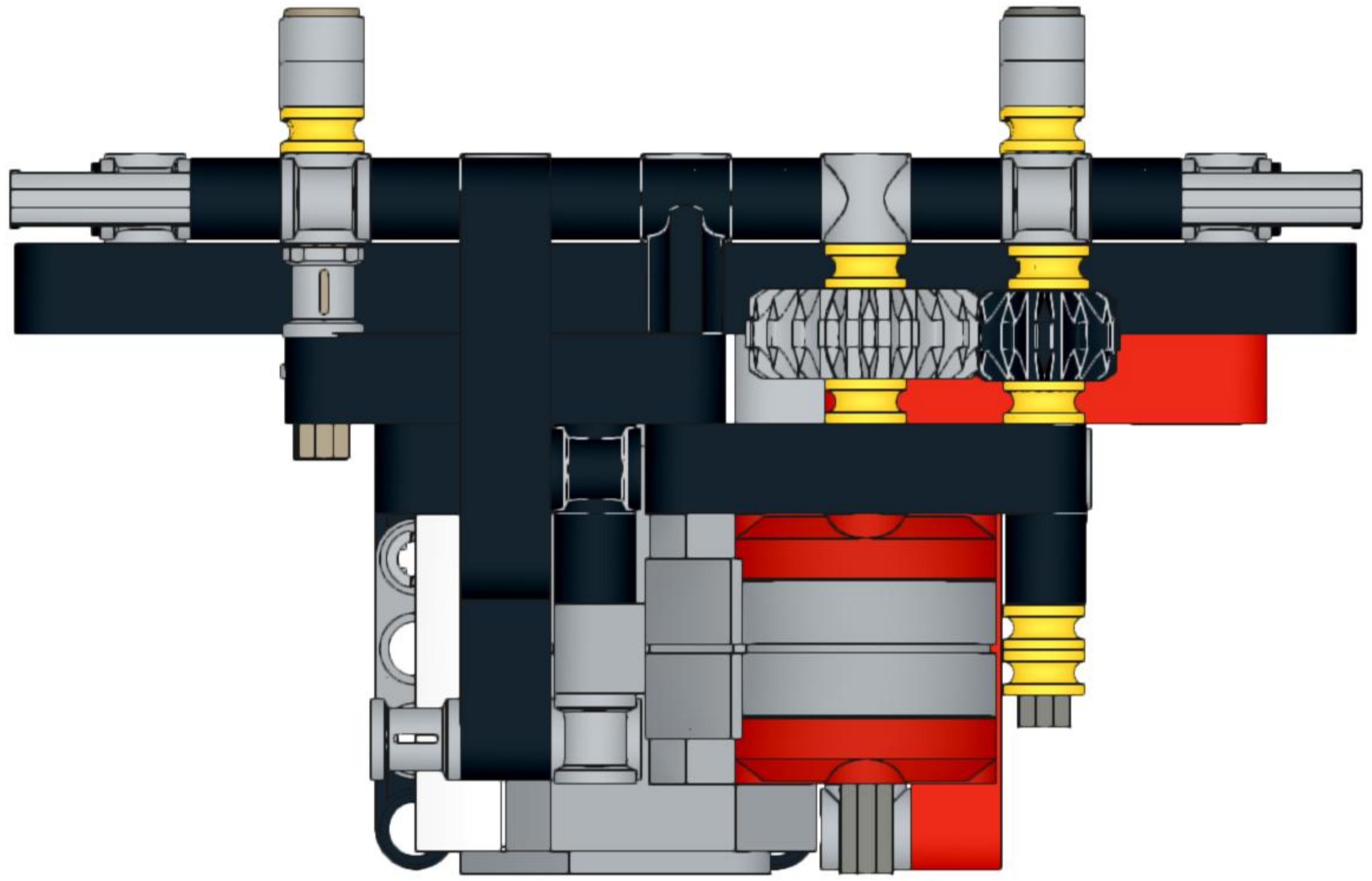

50

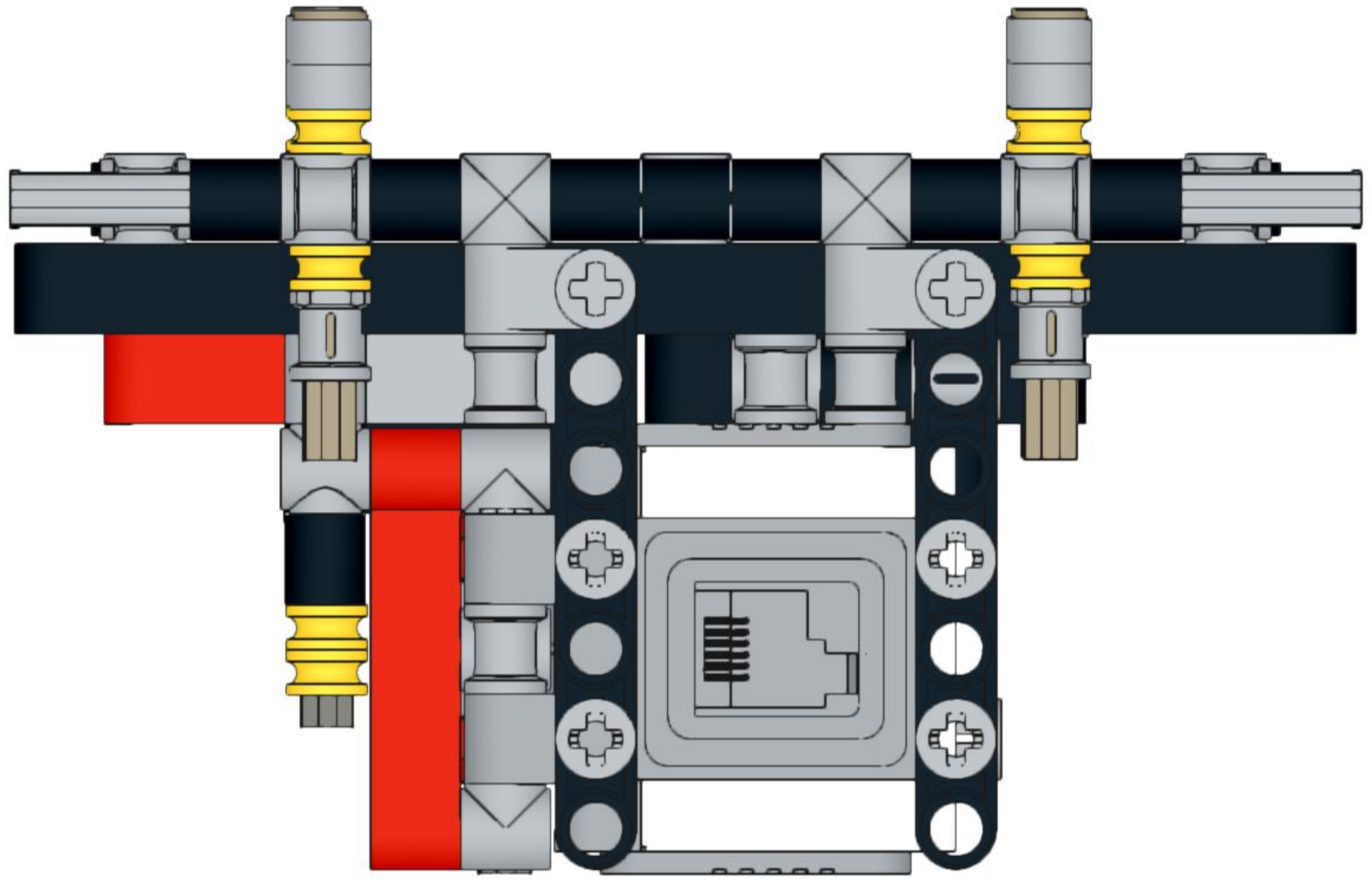

51

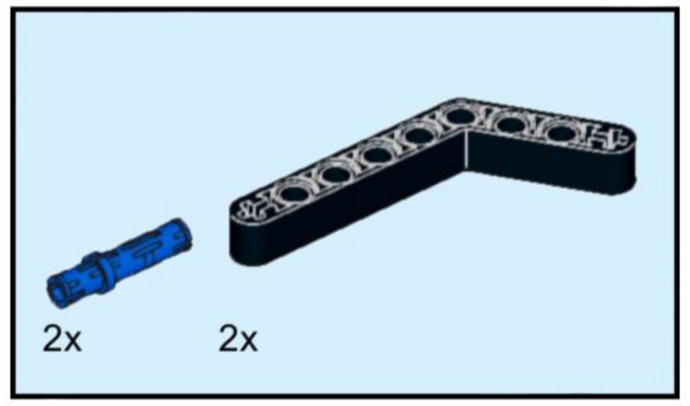


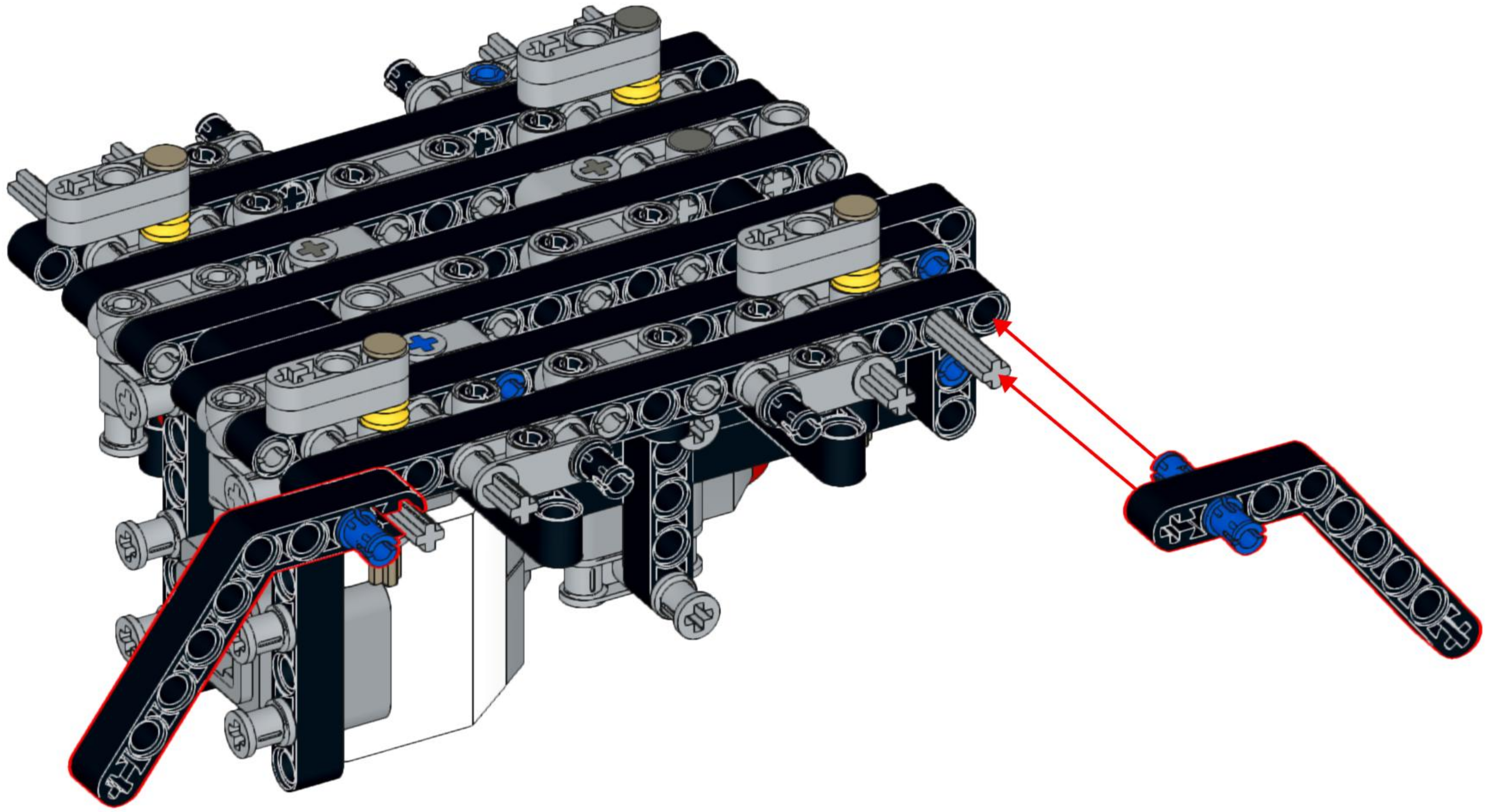

52

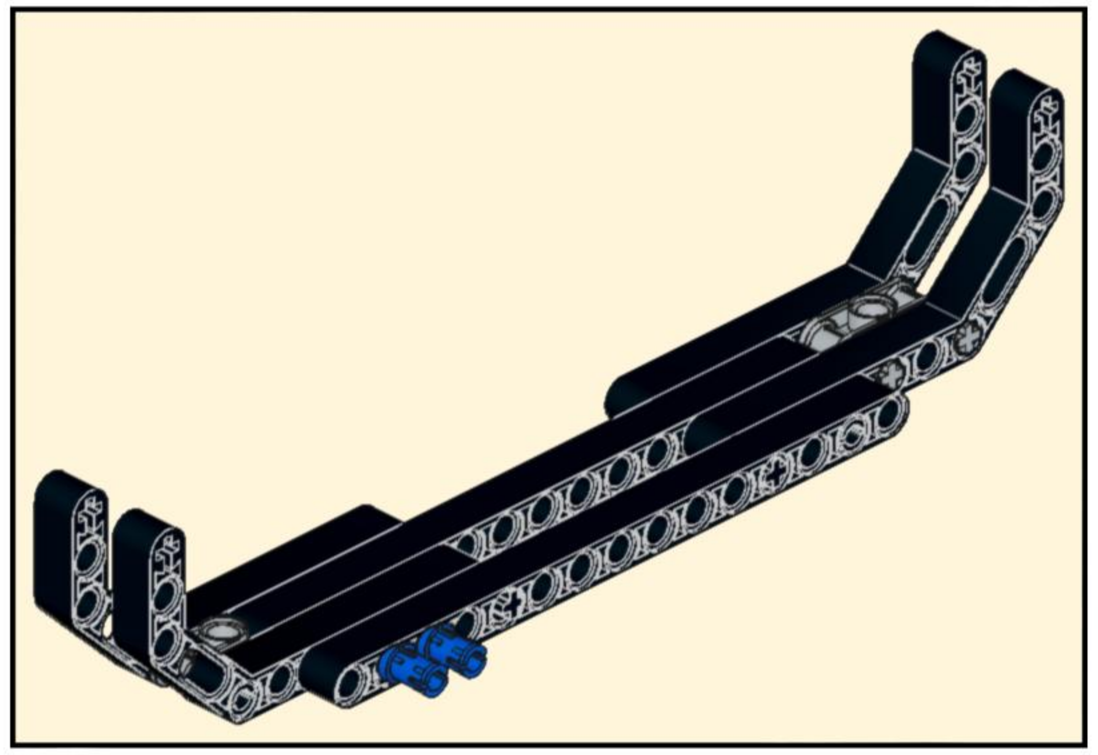

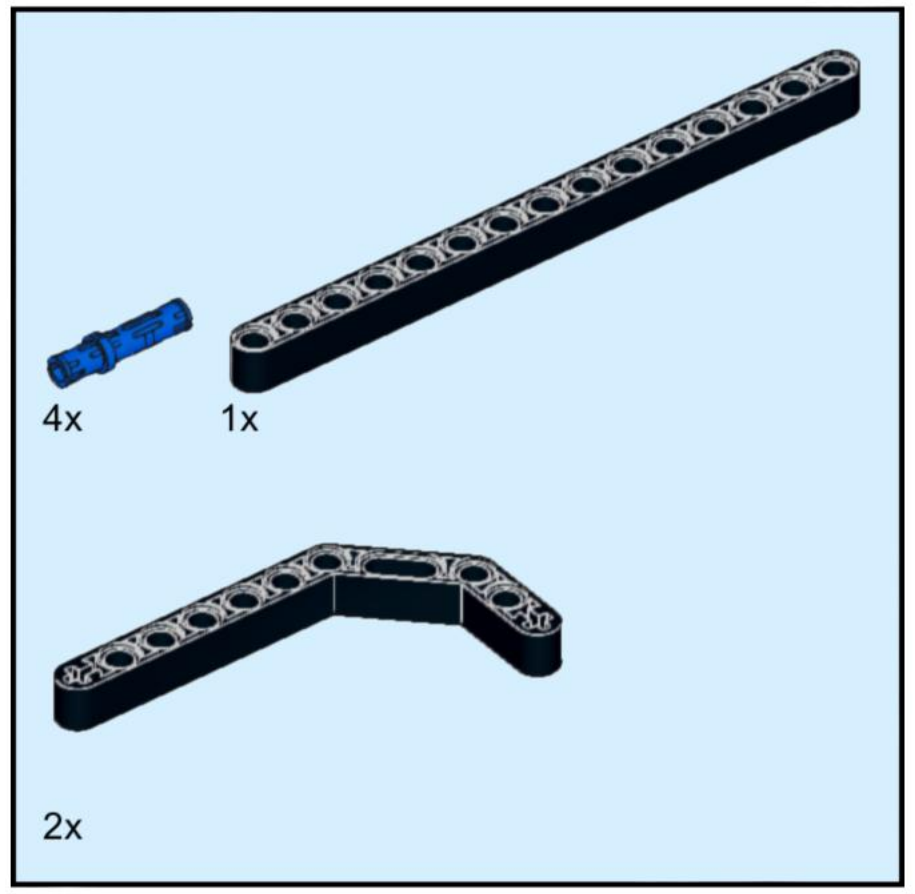


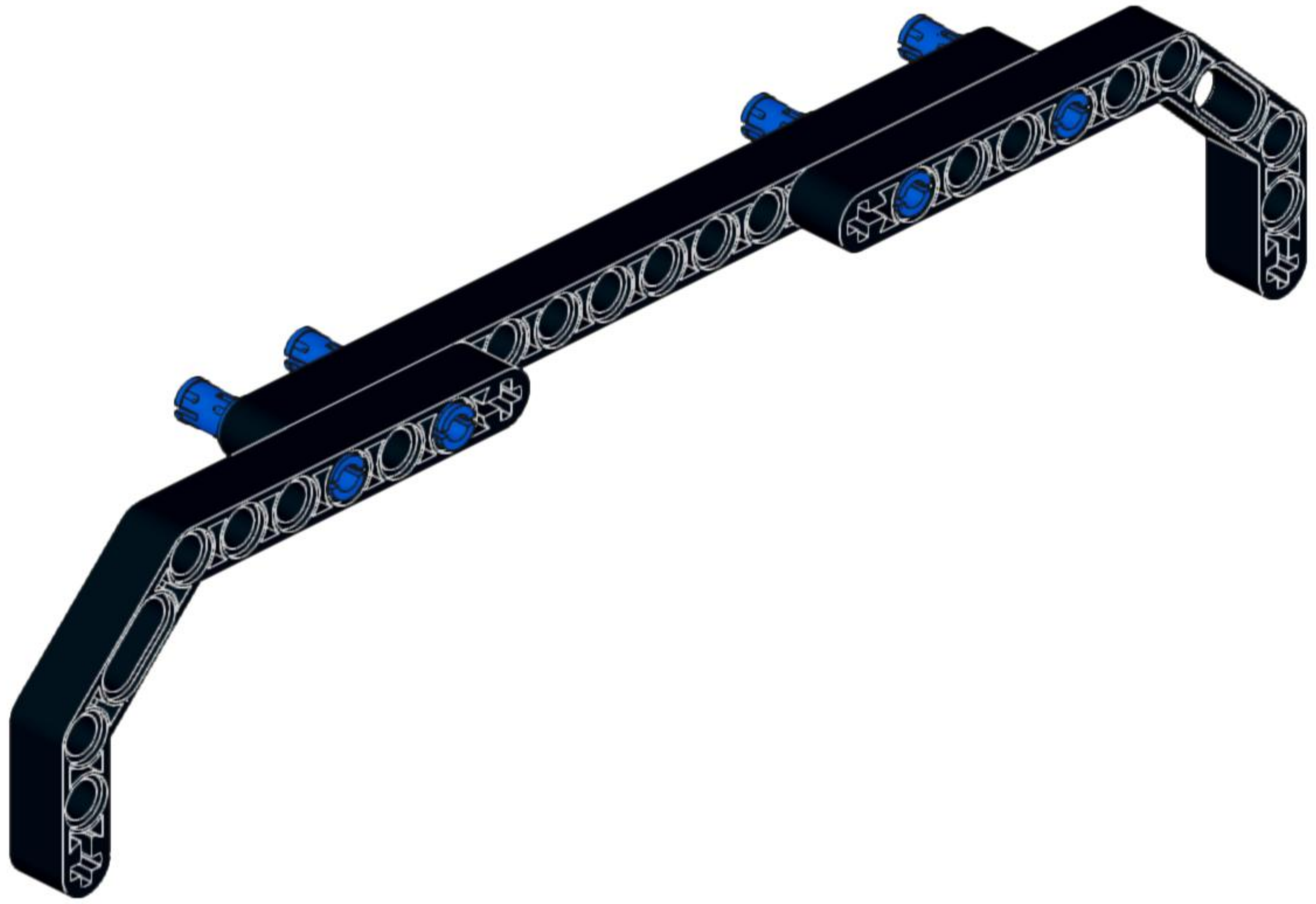

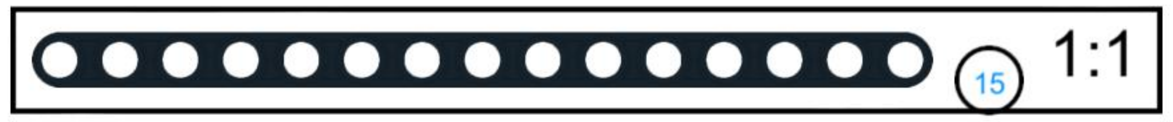

## 53

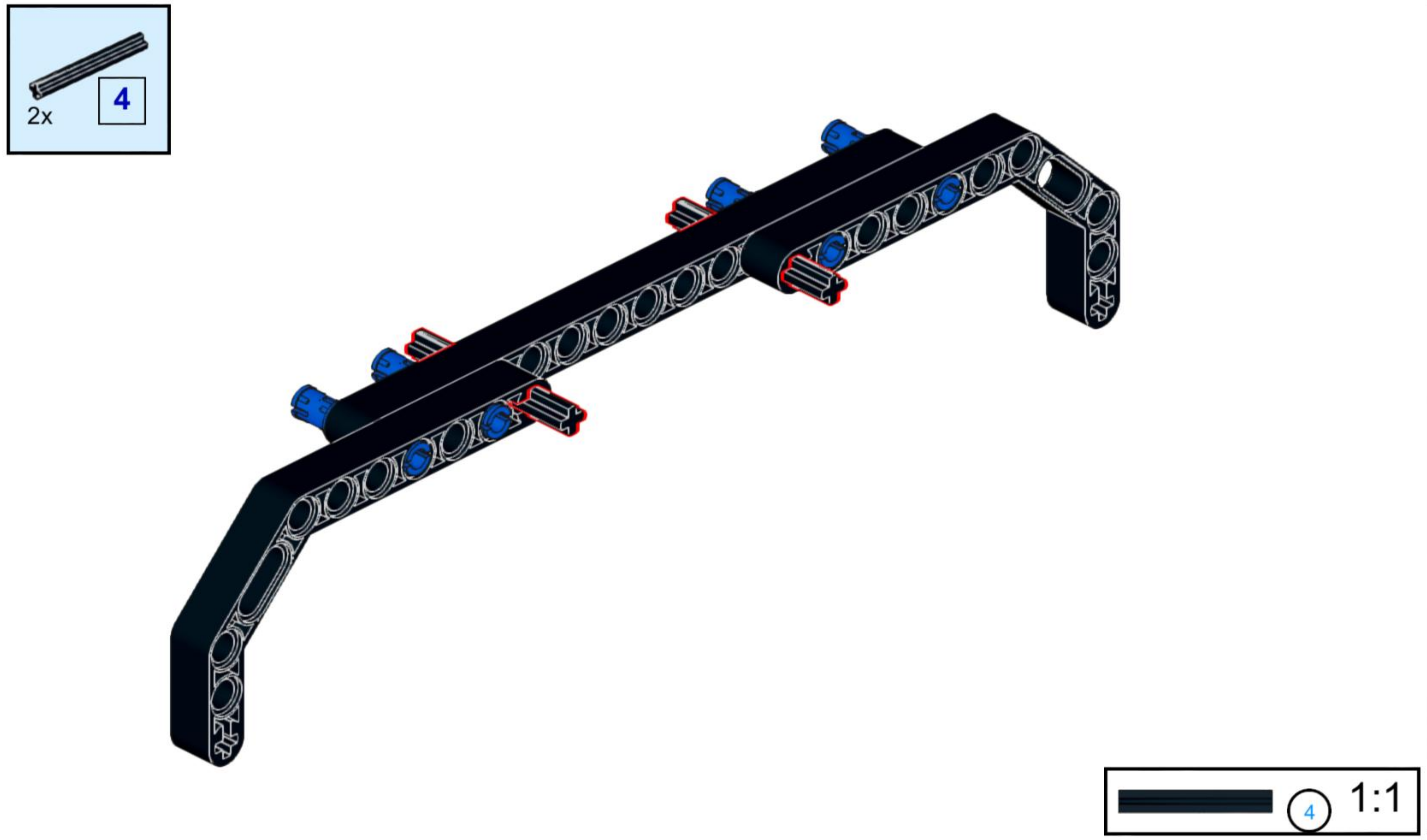


## 54

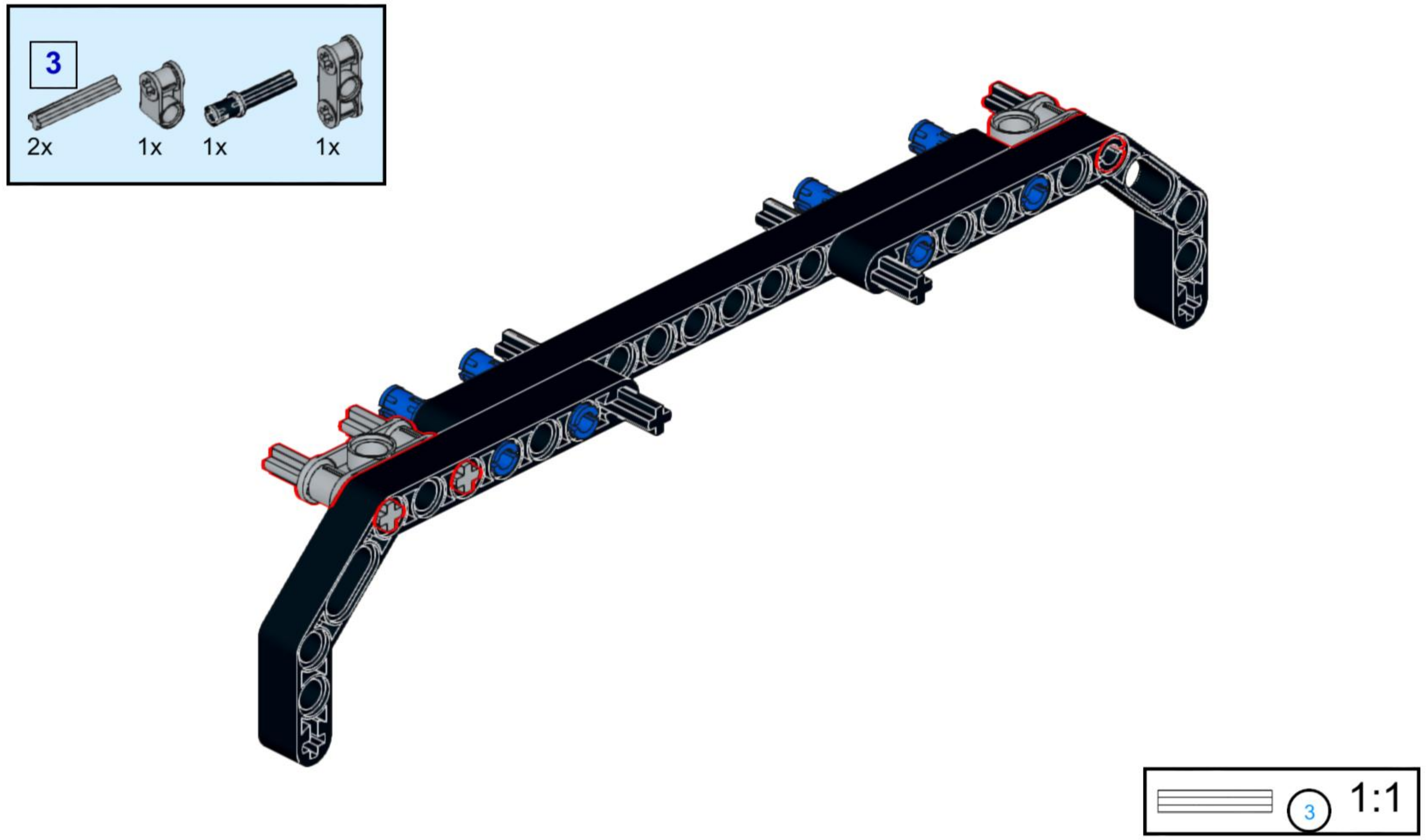


## 55

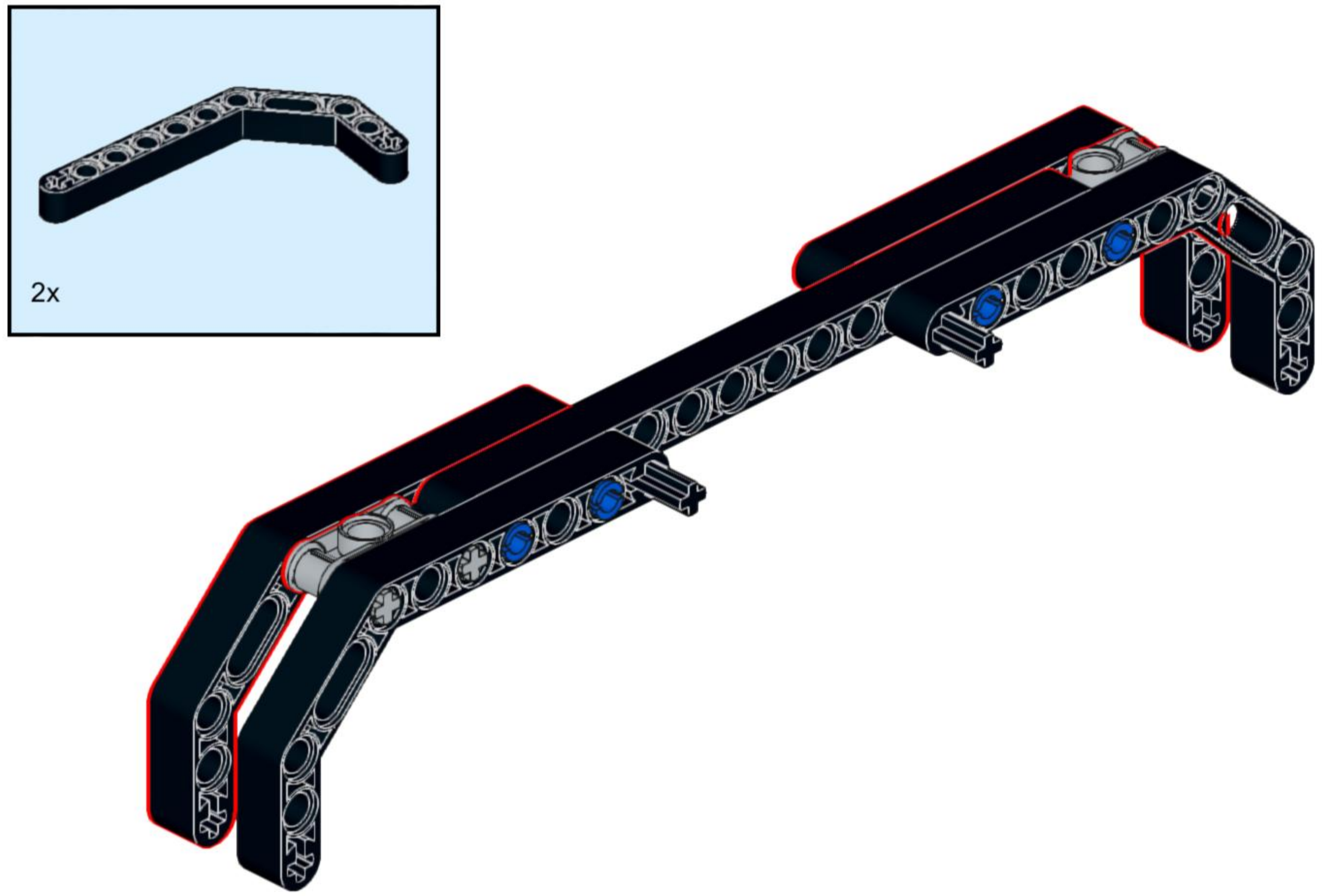


## 56

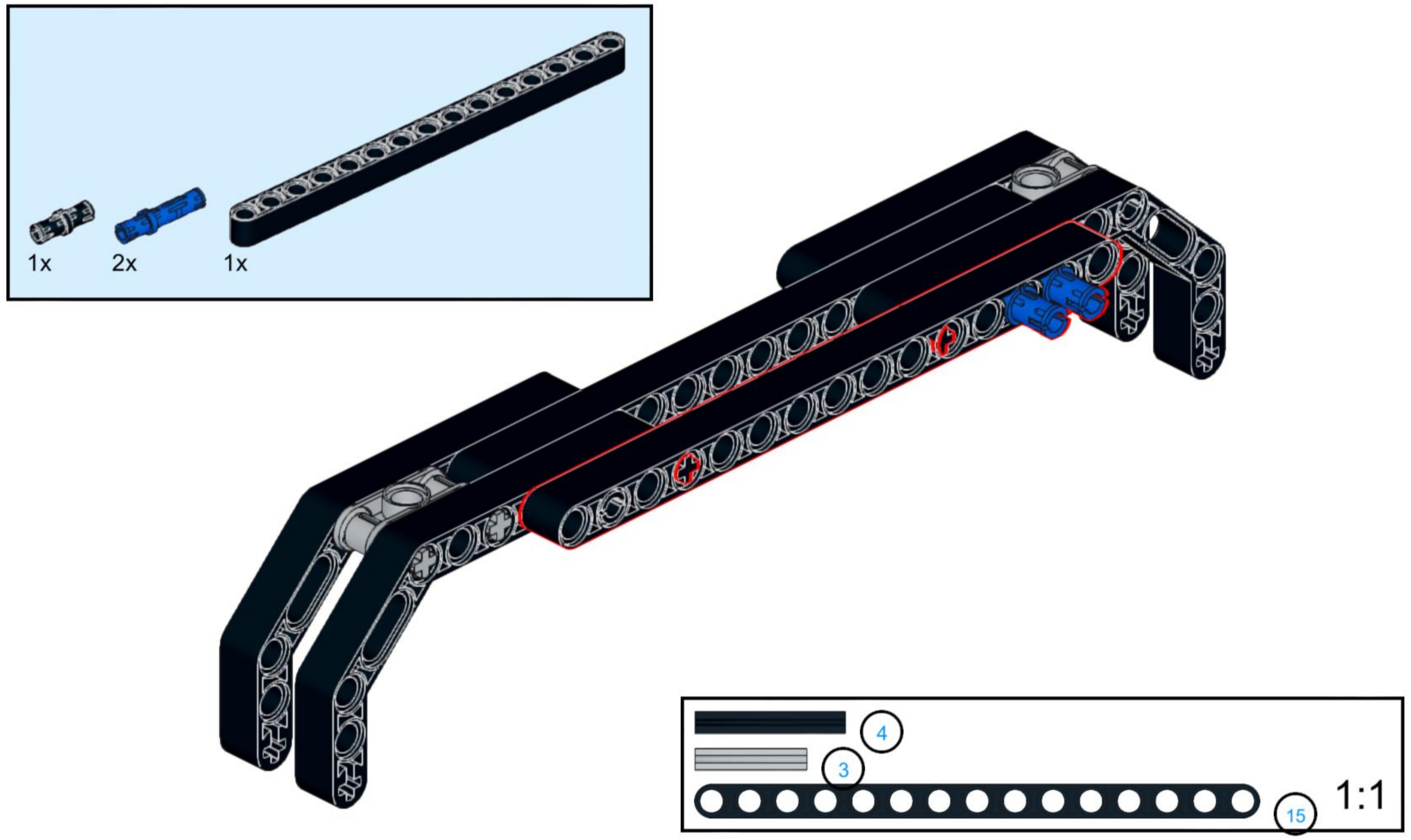

# 57

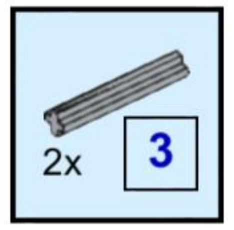


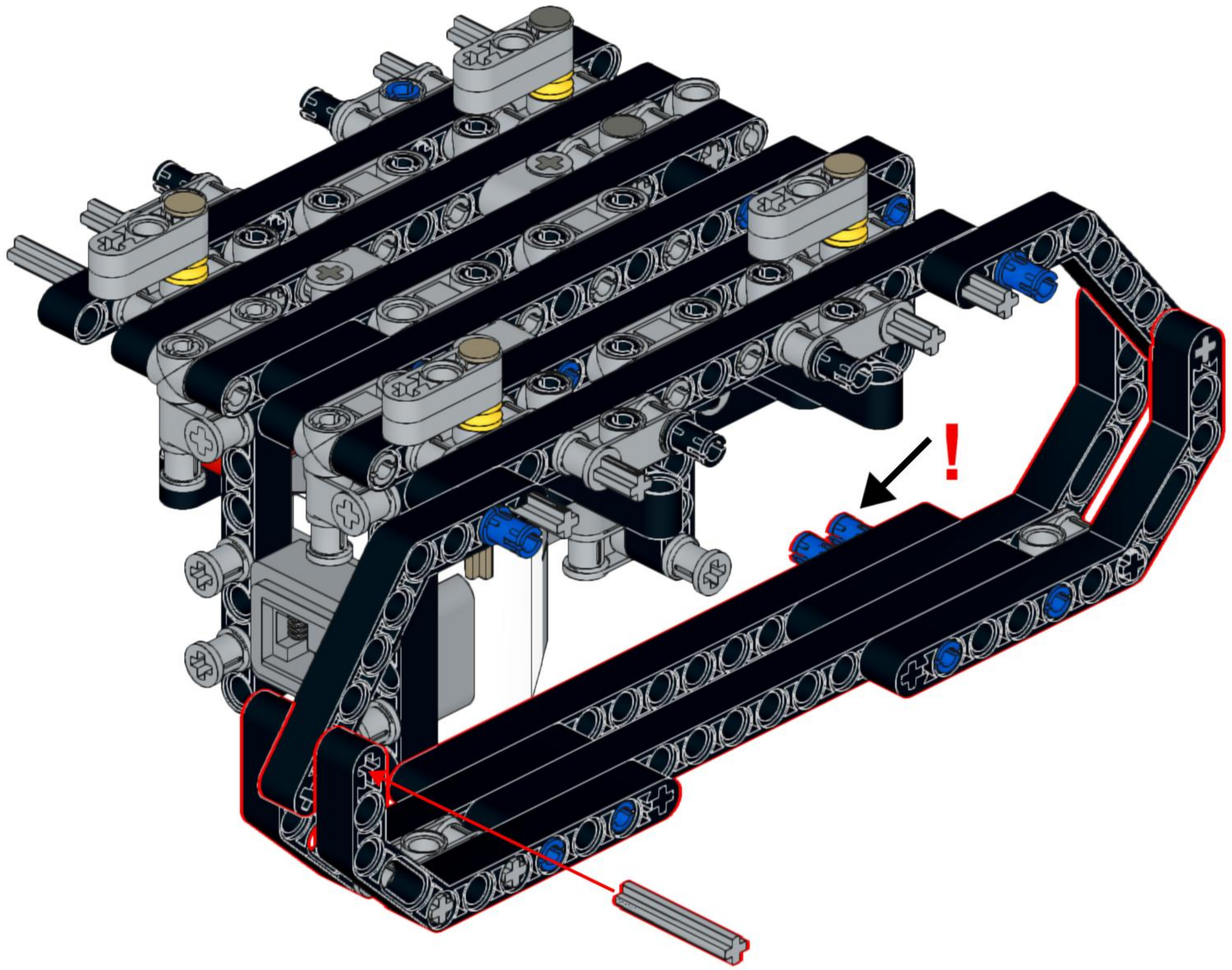


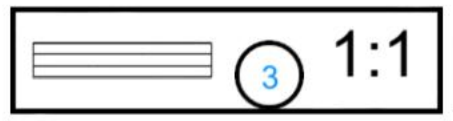


# 58

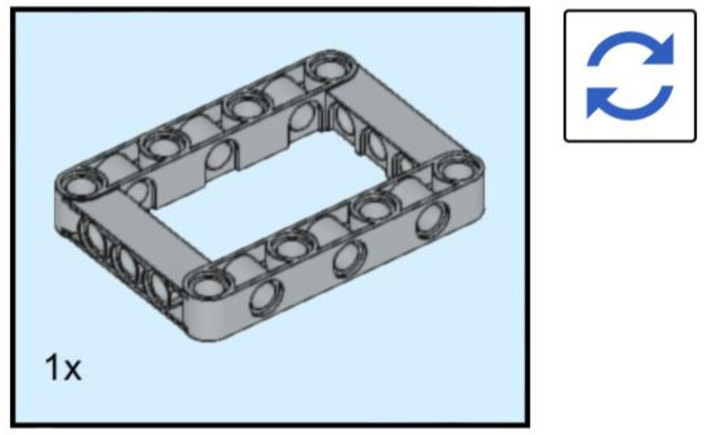


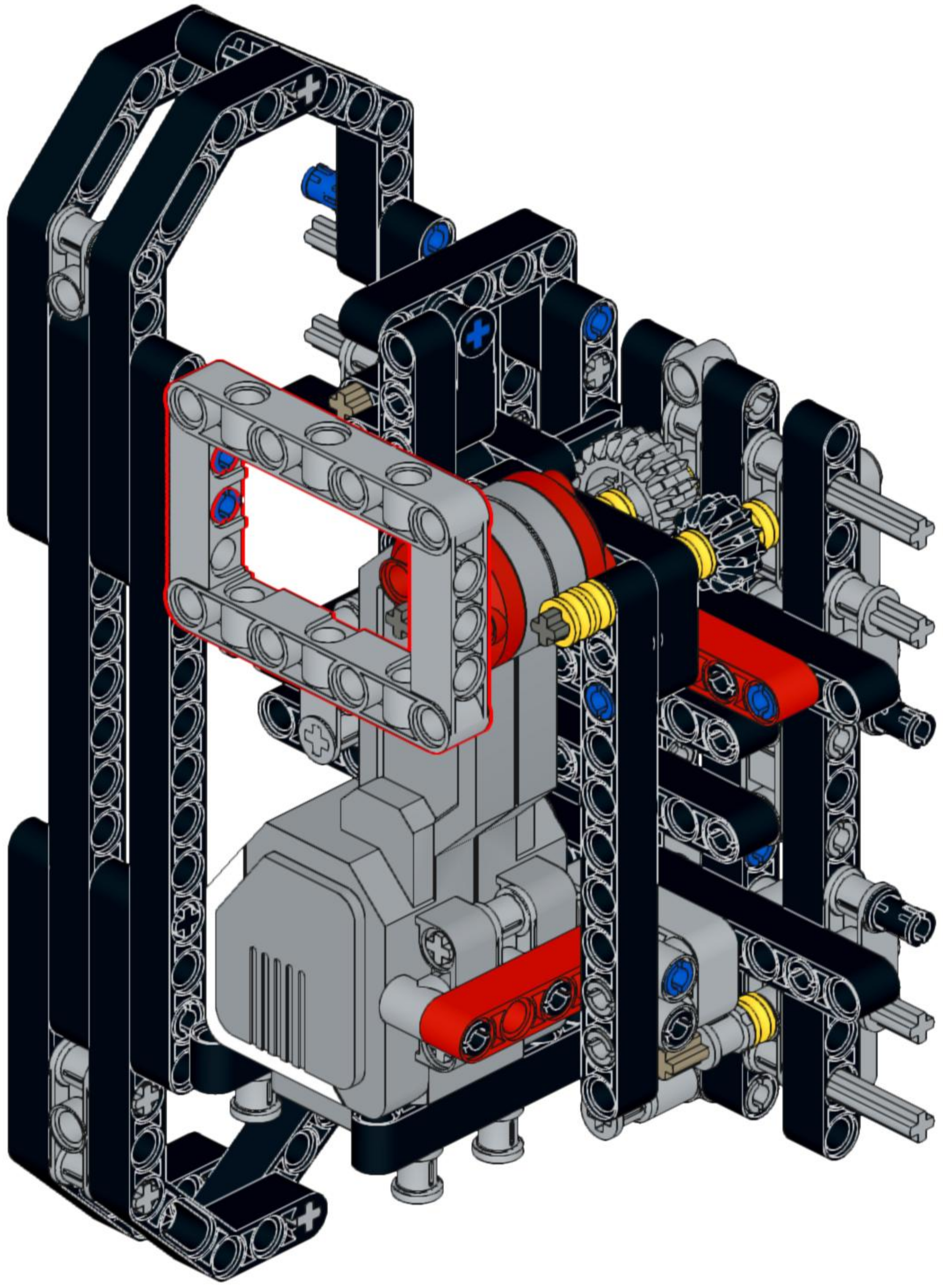

59

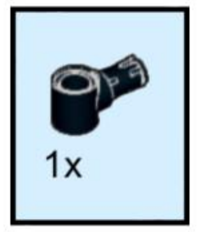


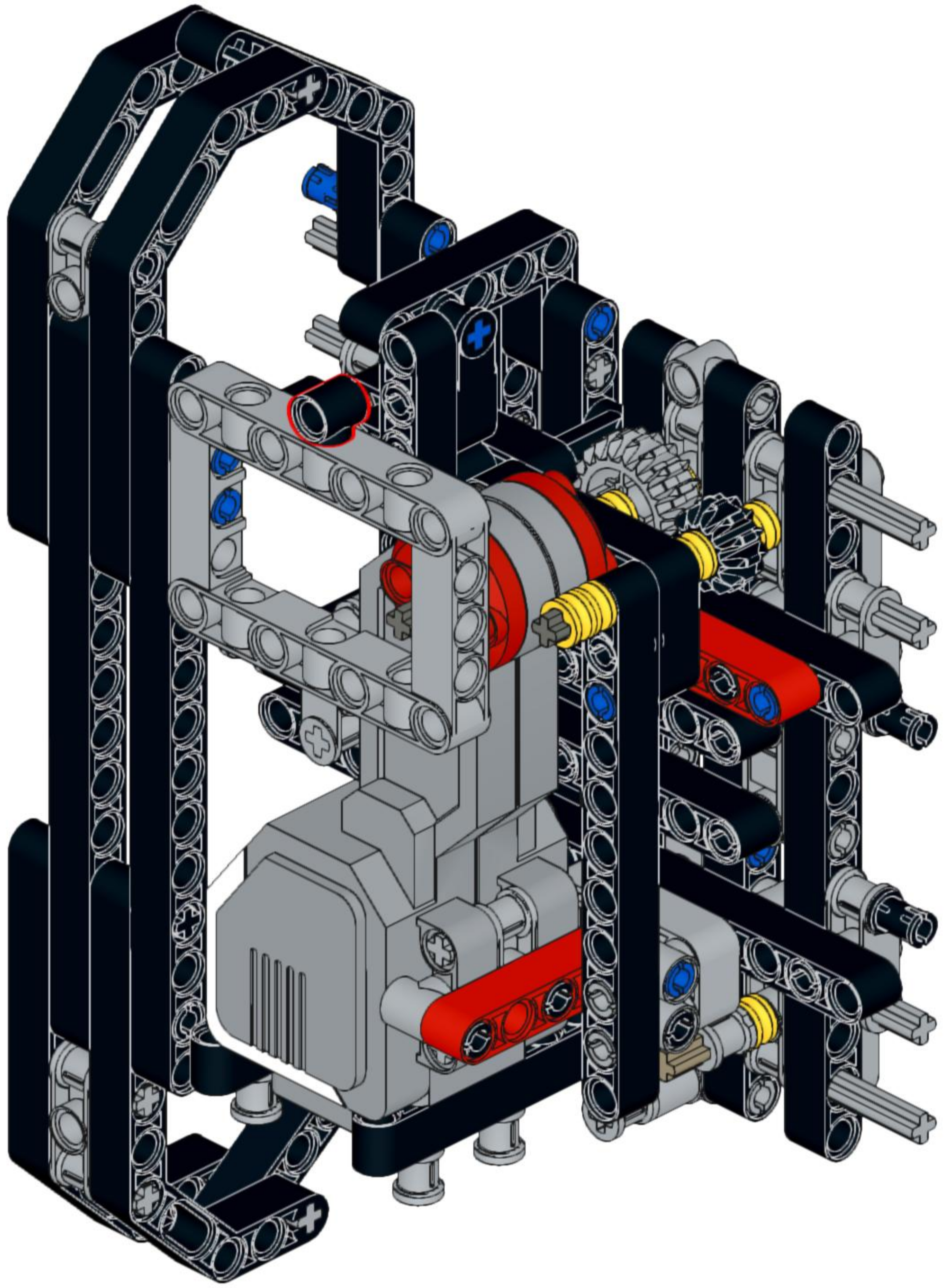

60

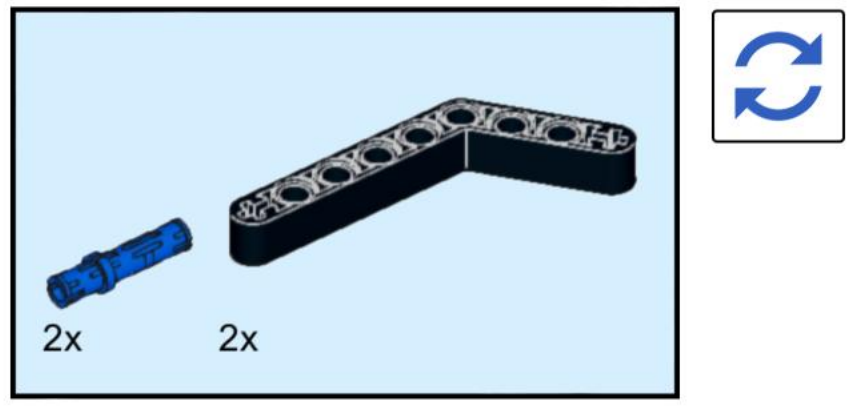


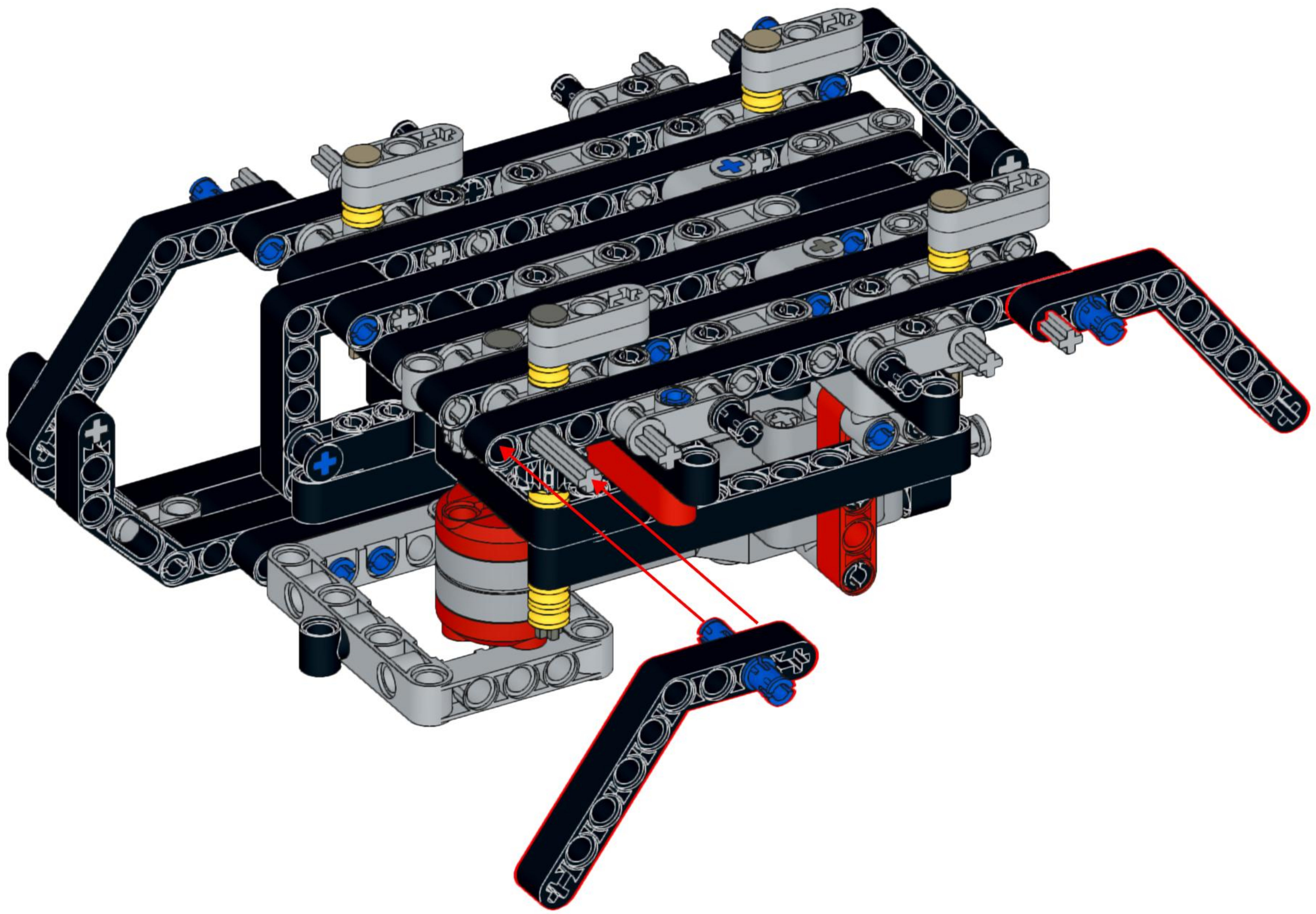

# 61

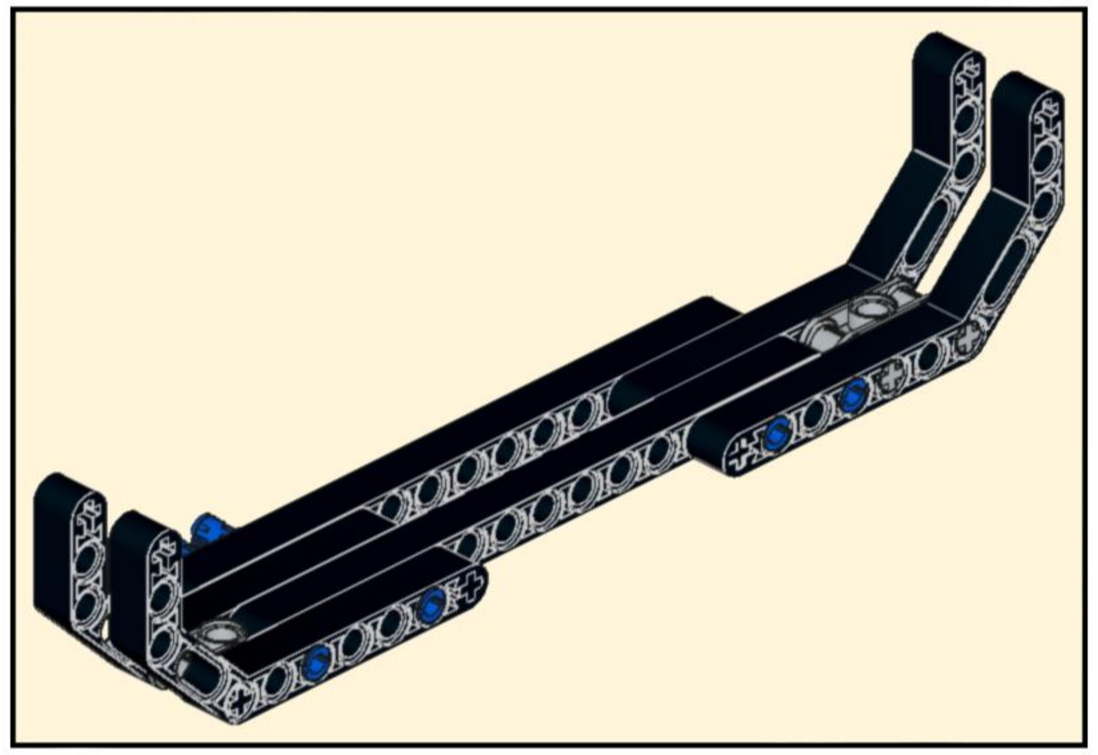

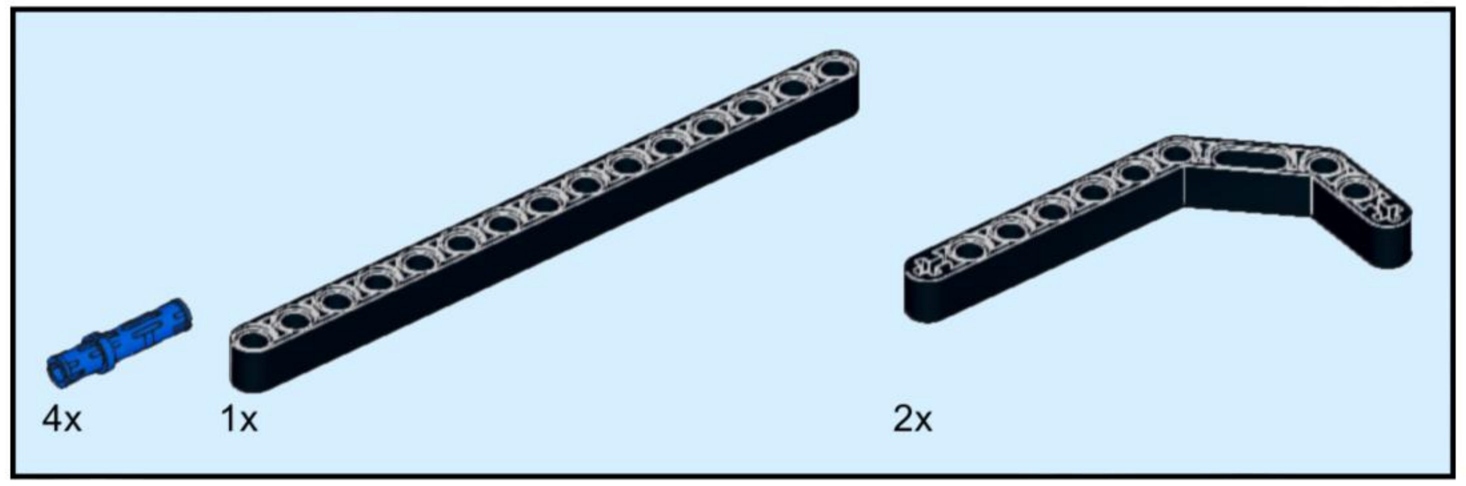


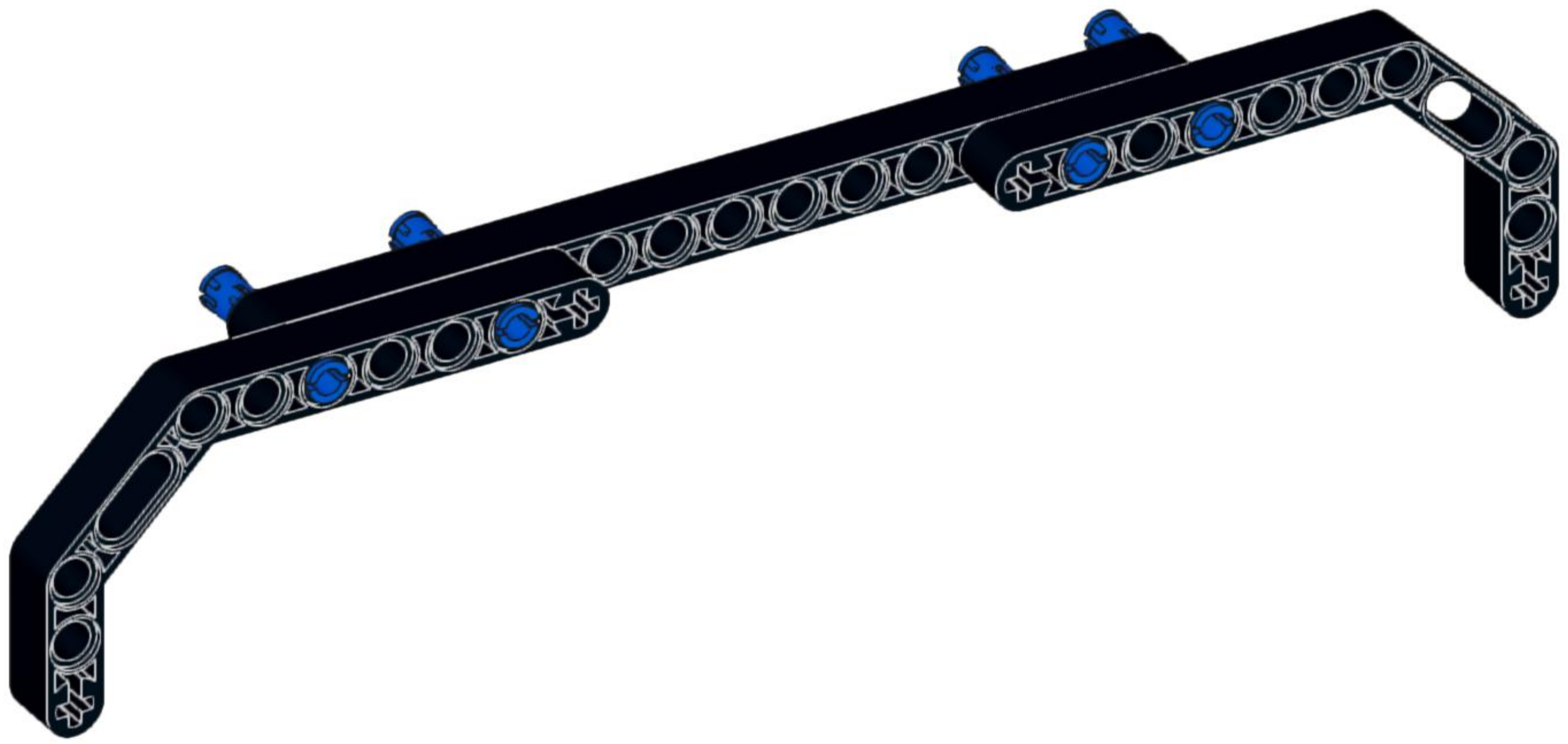

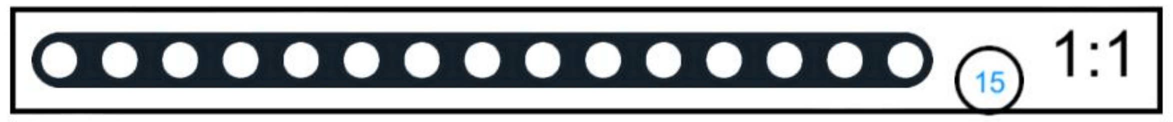

62

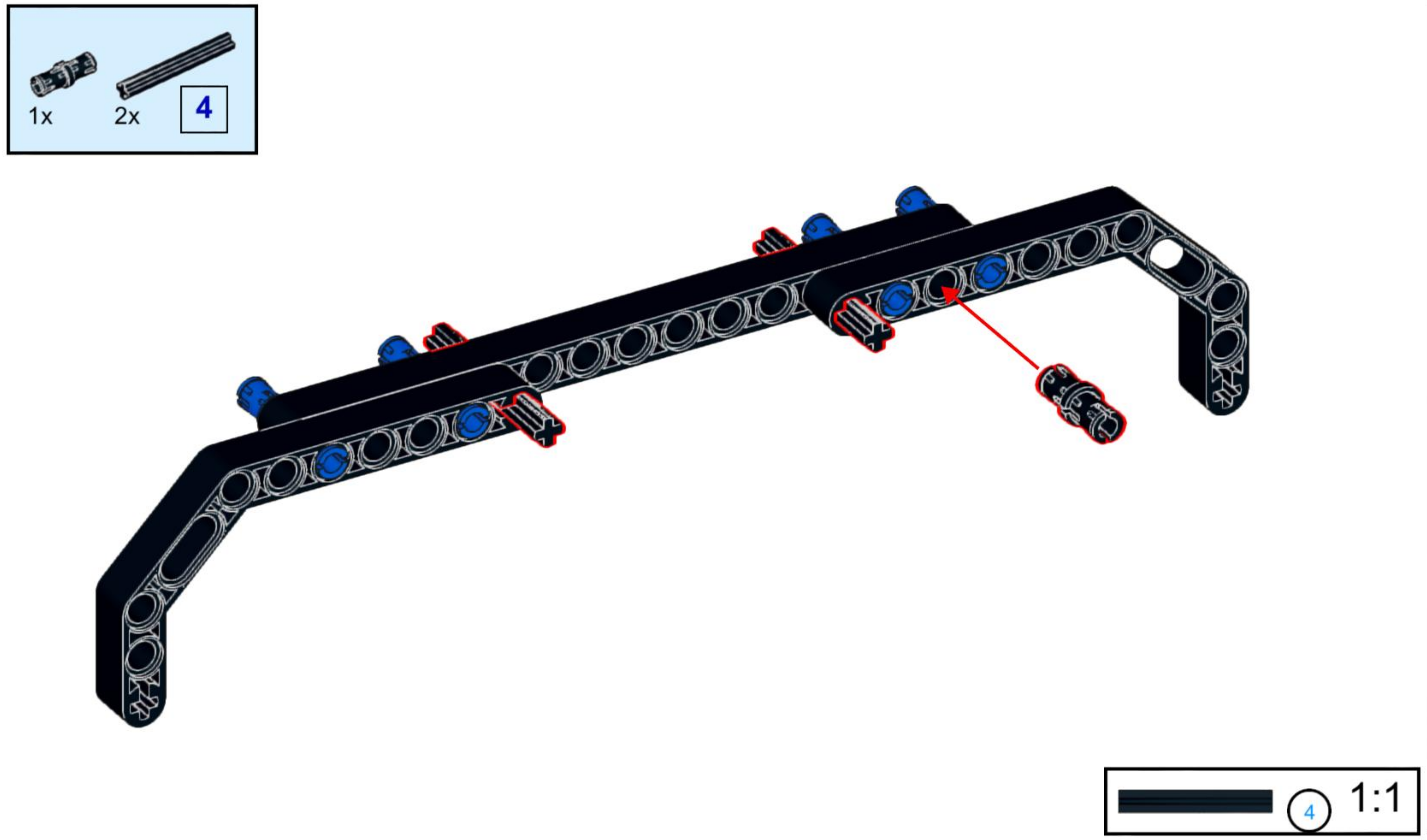


63

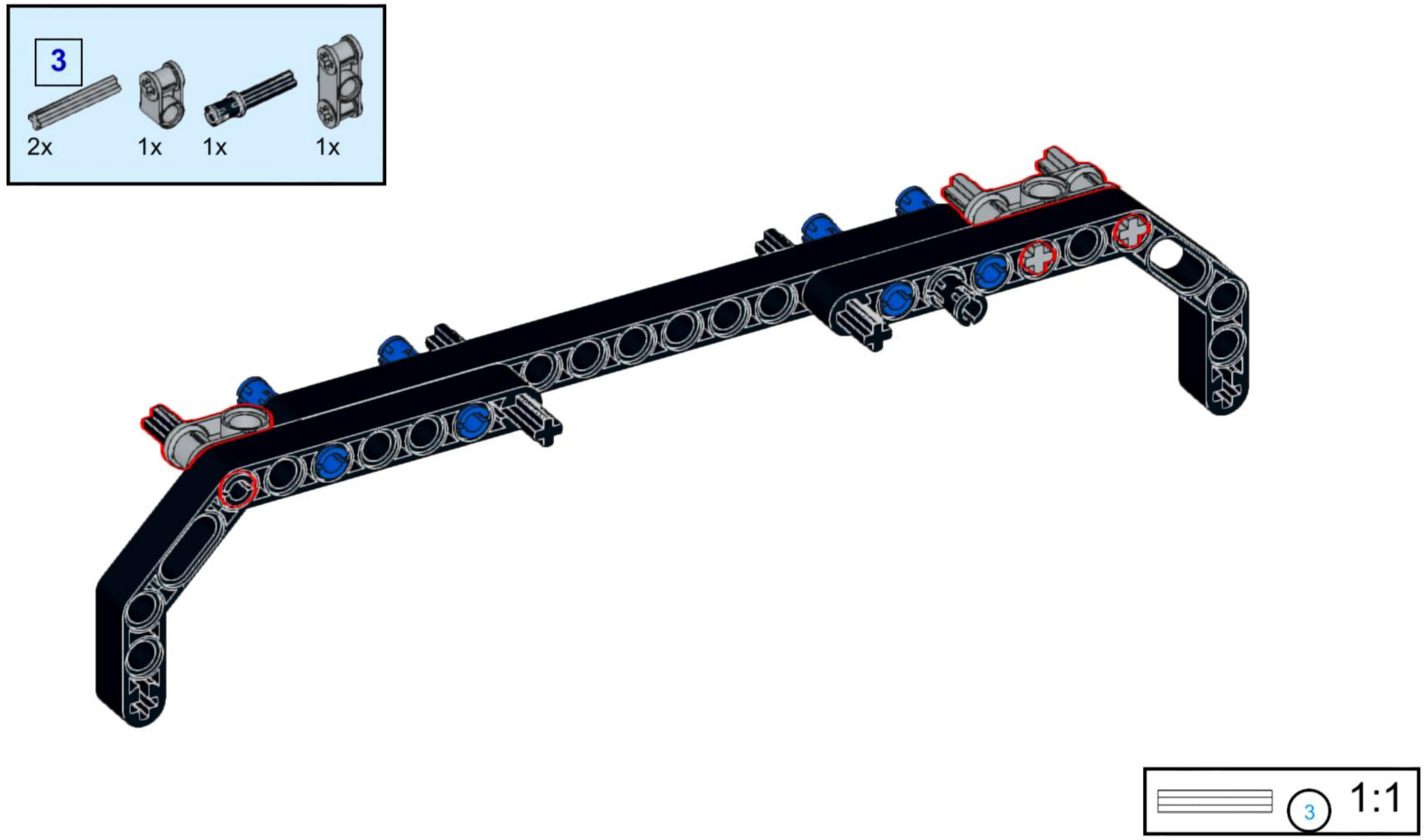


64

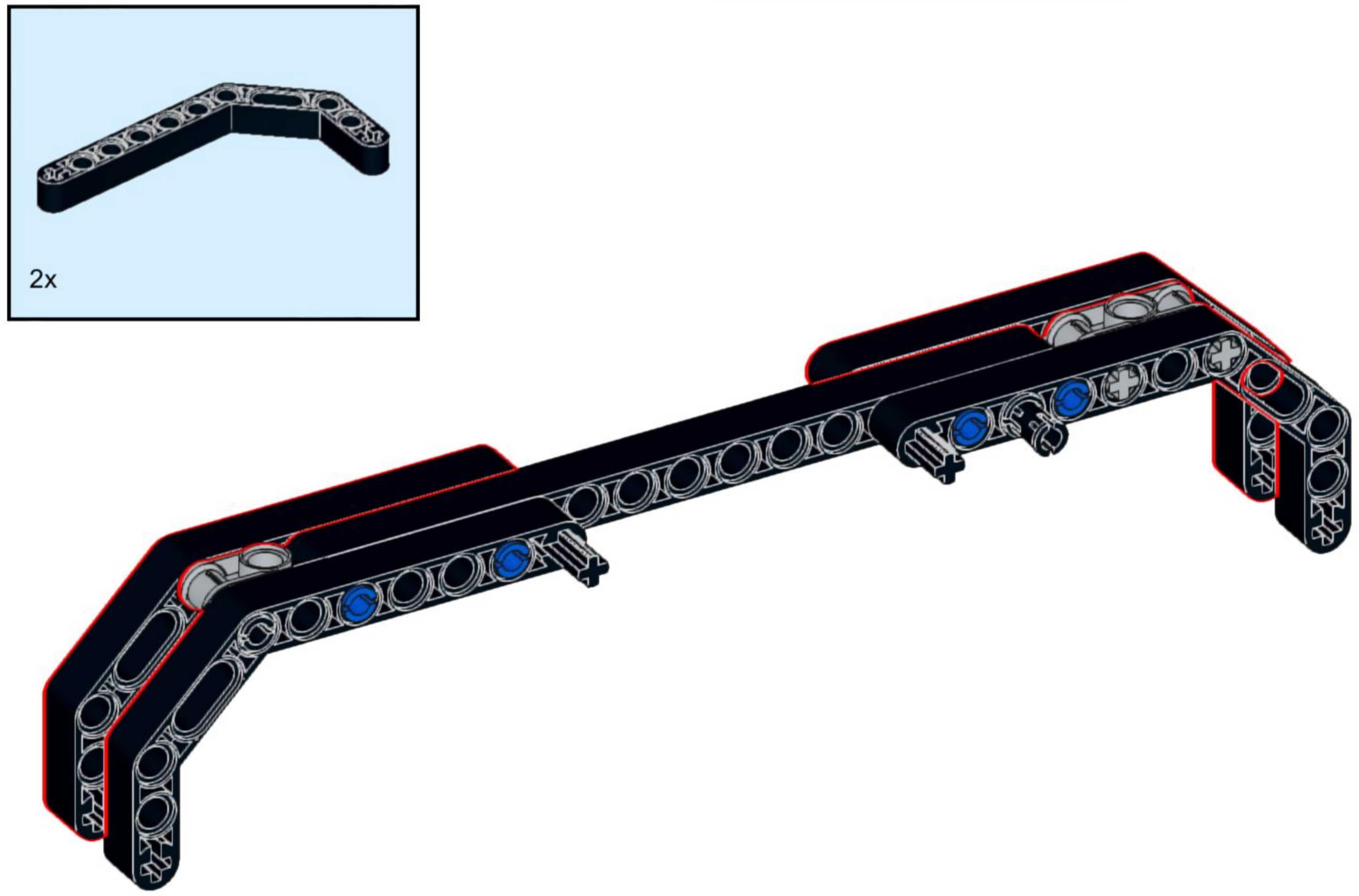


65

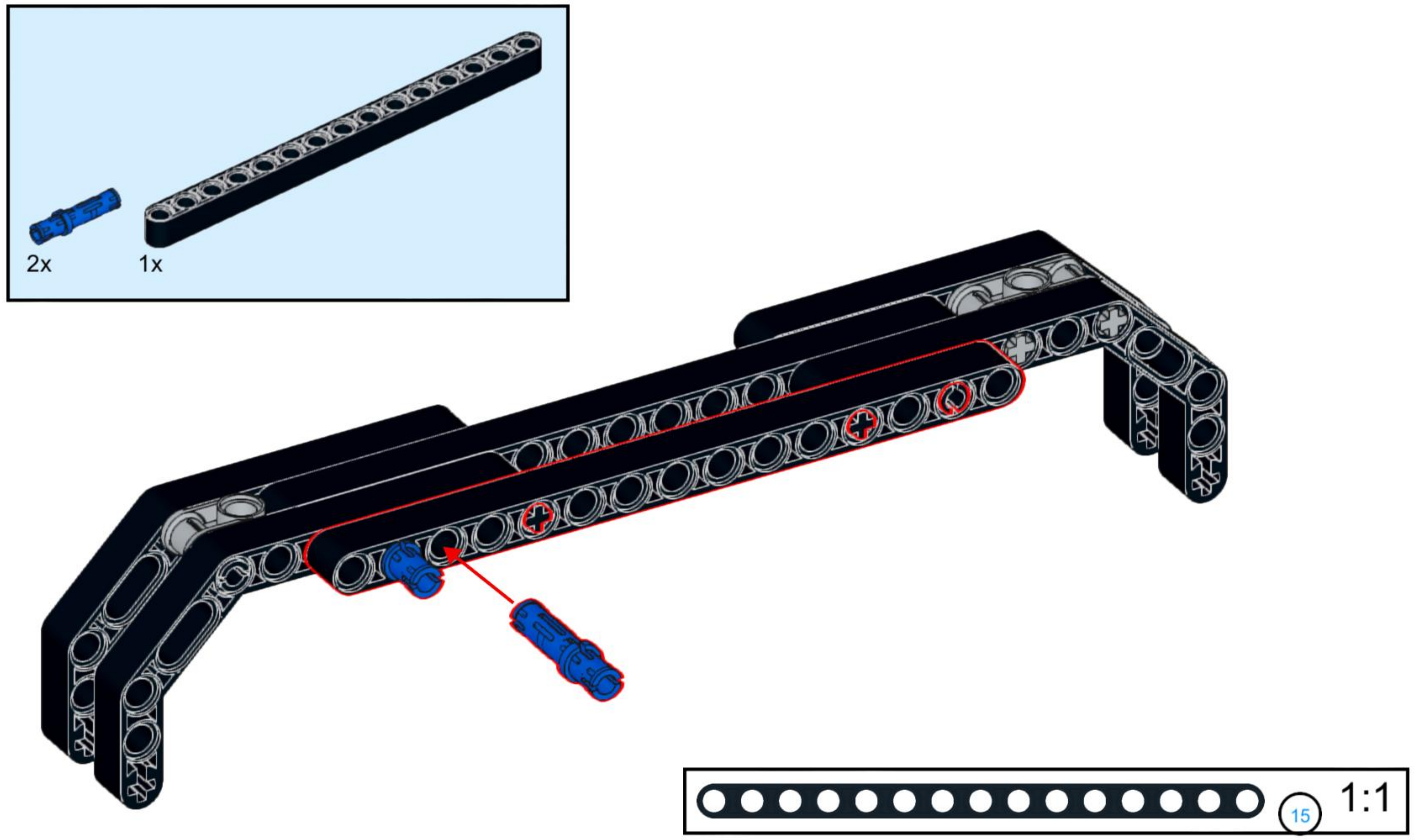

66

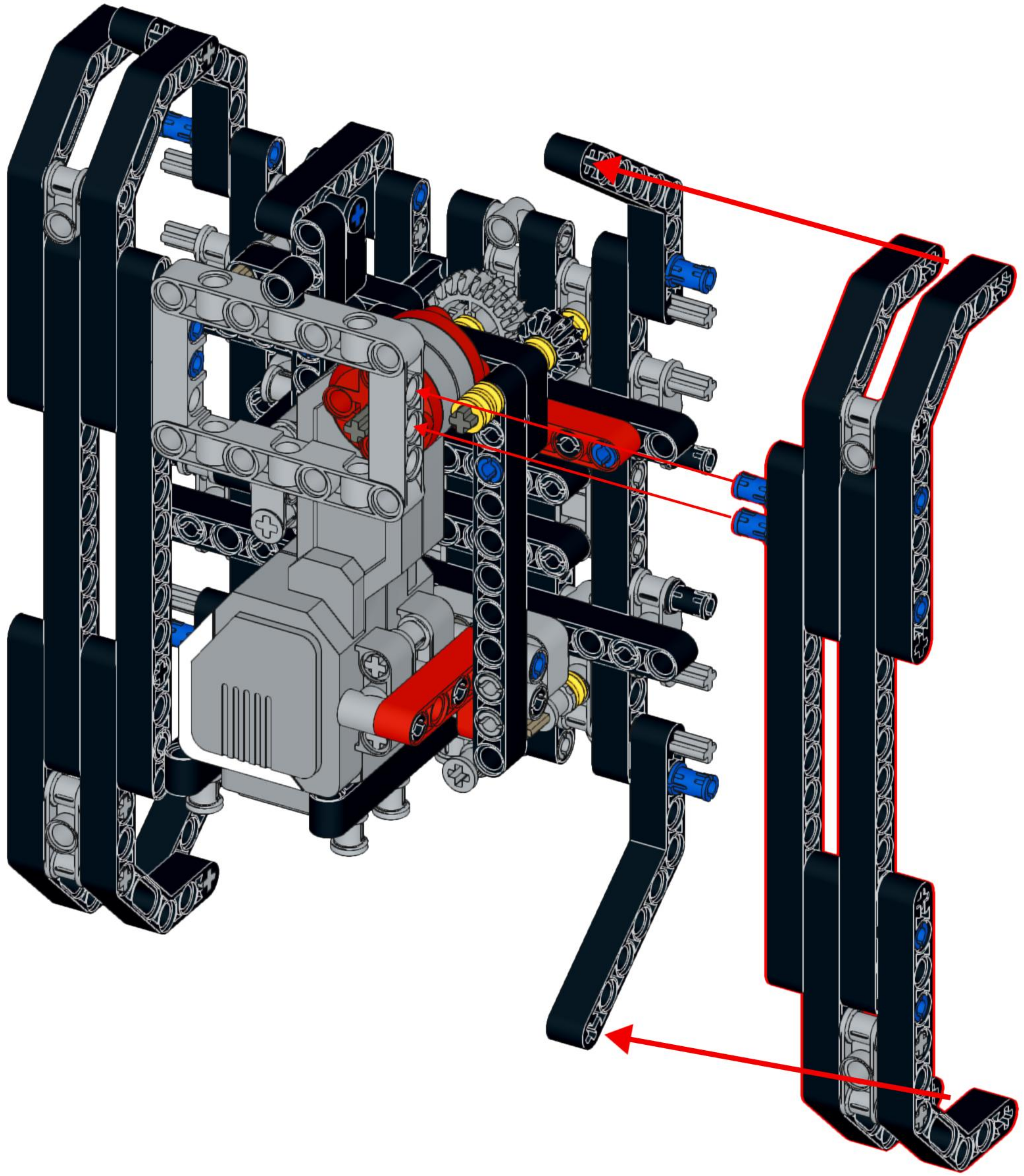

67

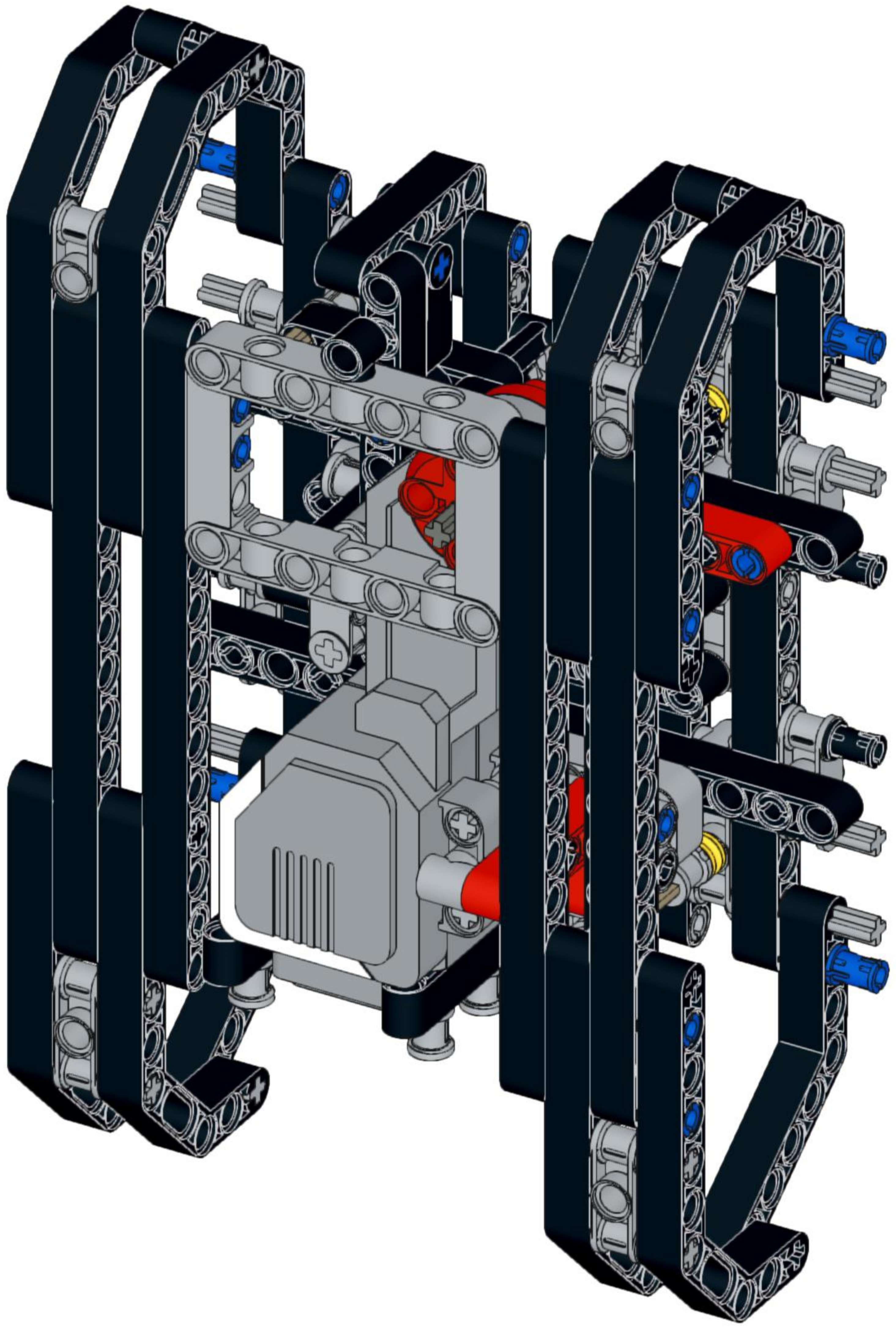

68

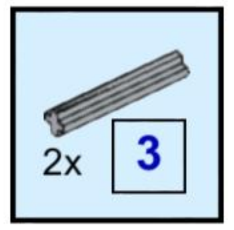


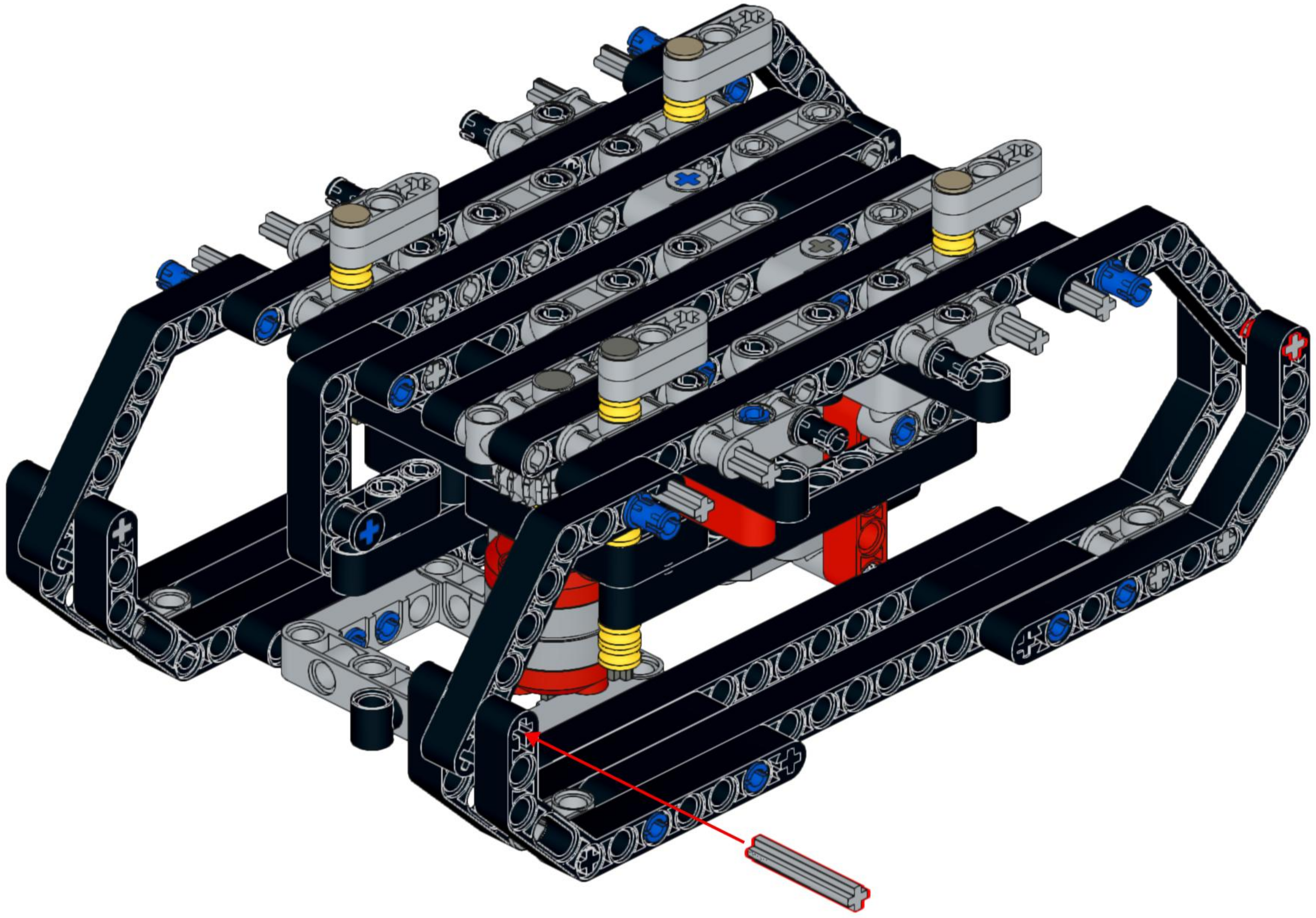

69

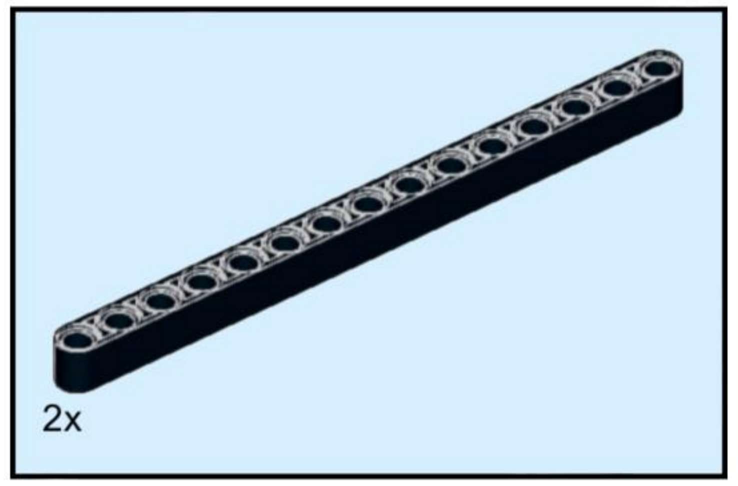


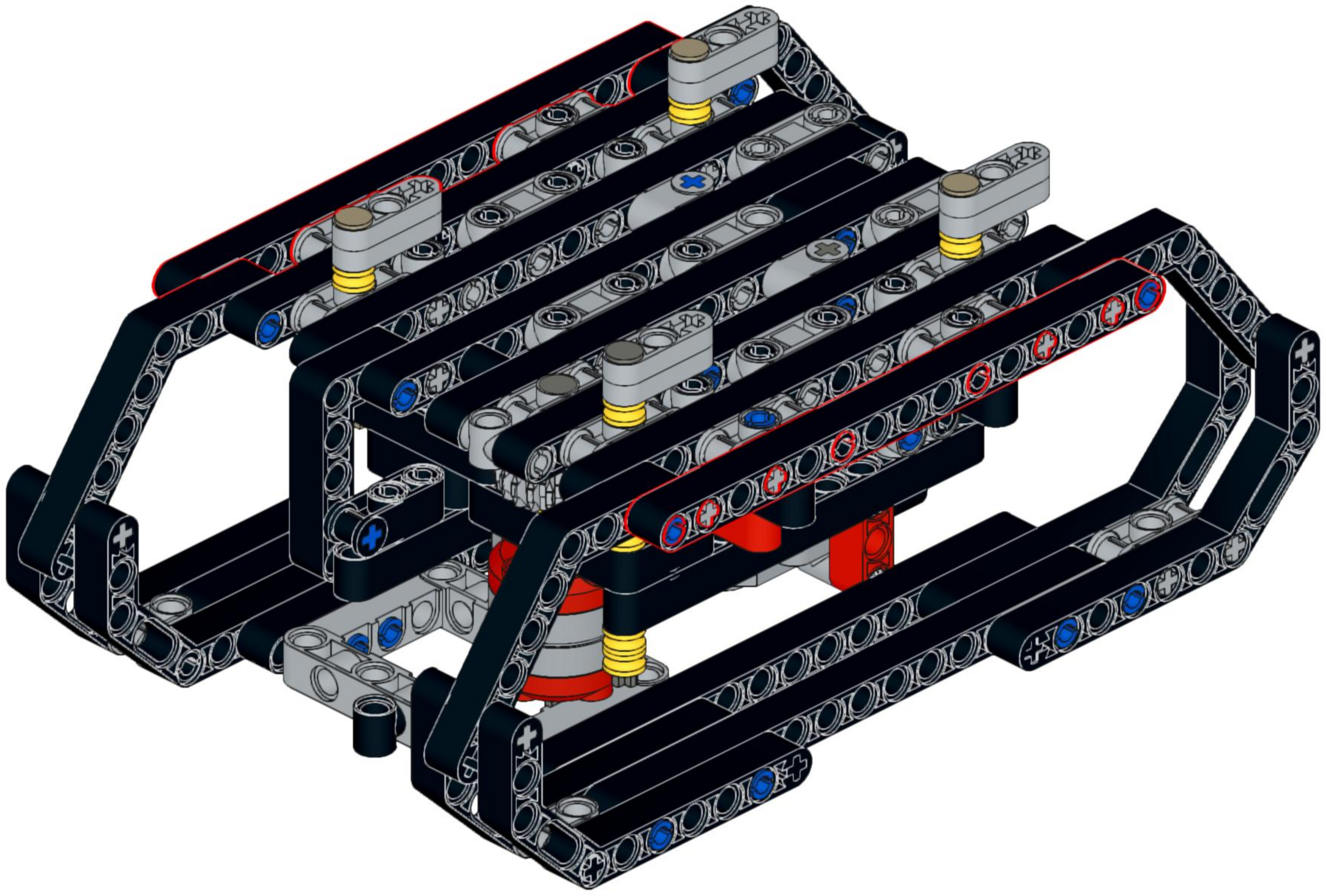

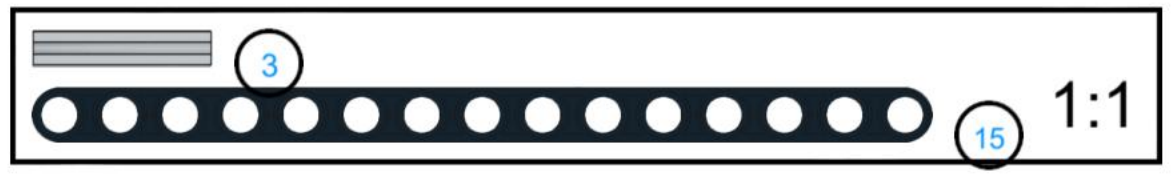

# 70

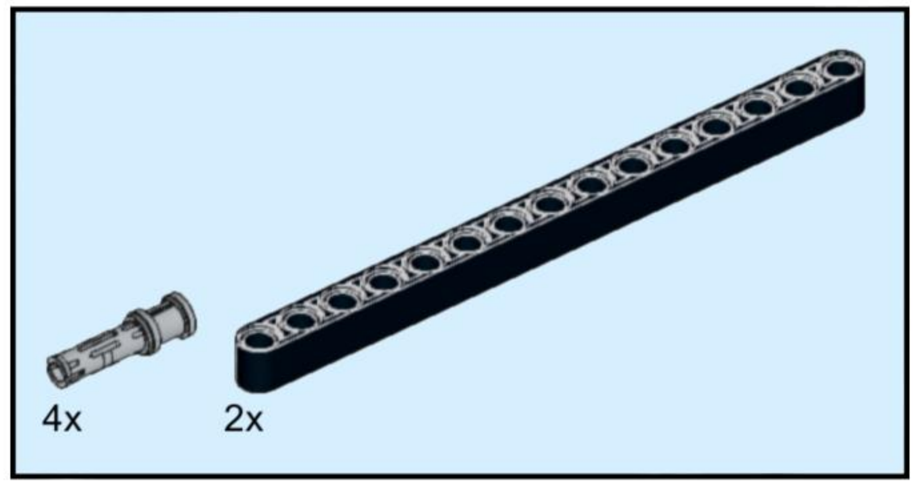


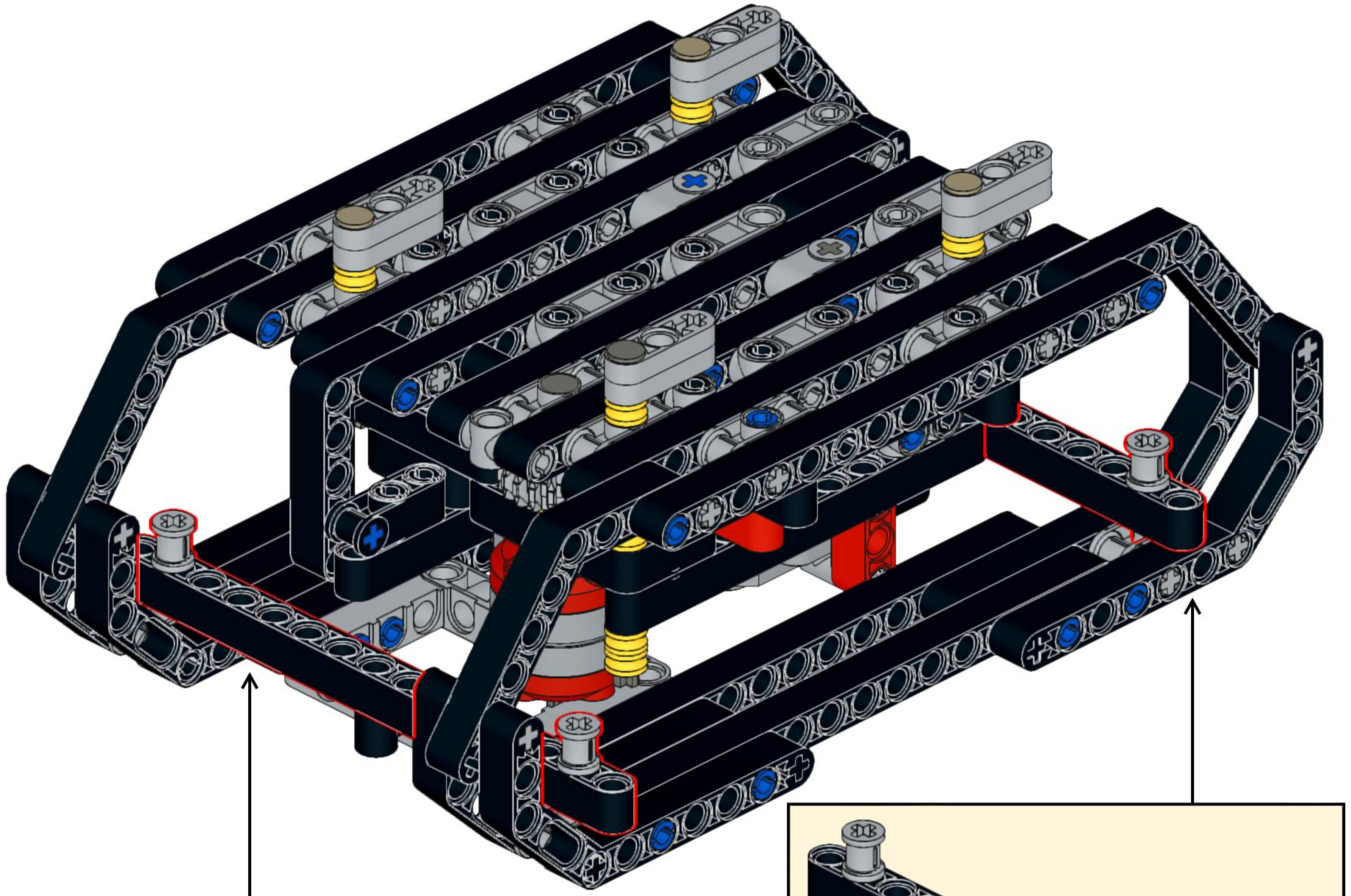

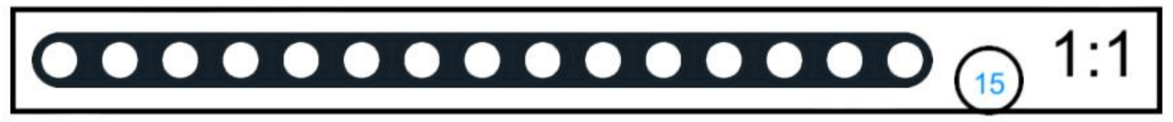


2x

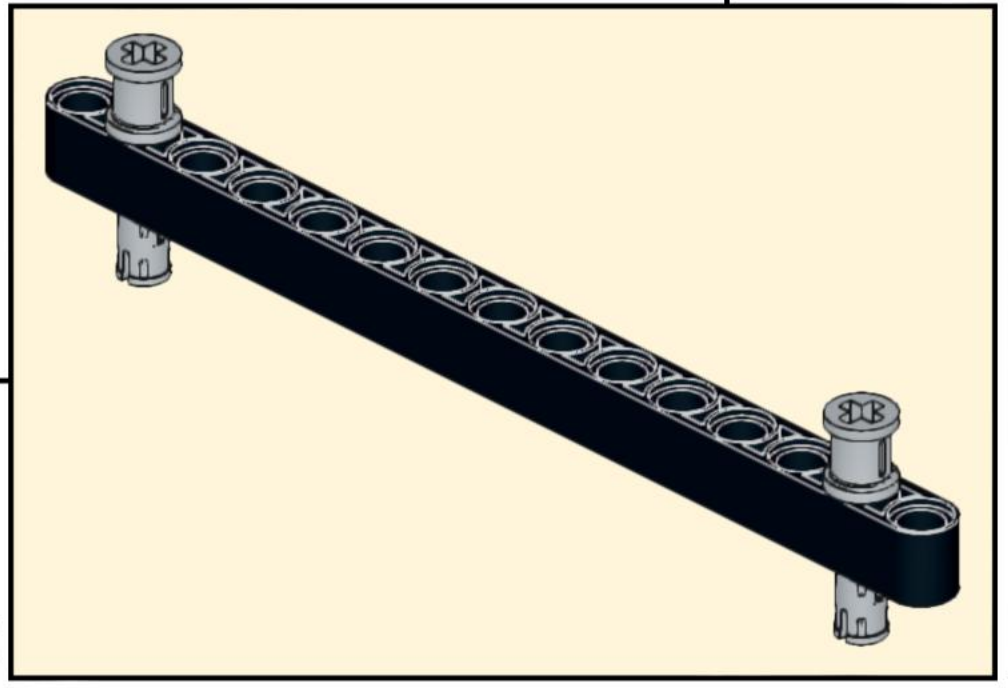

# 71

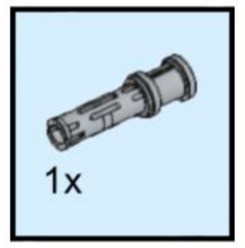


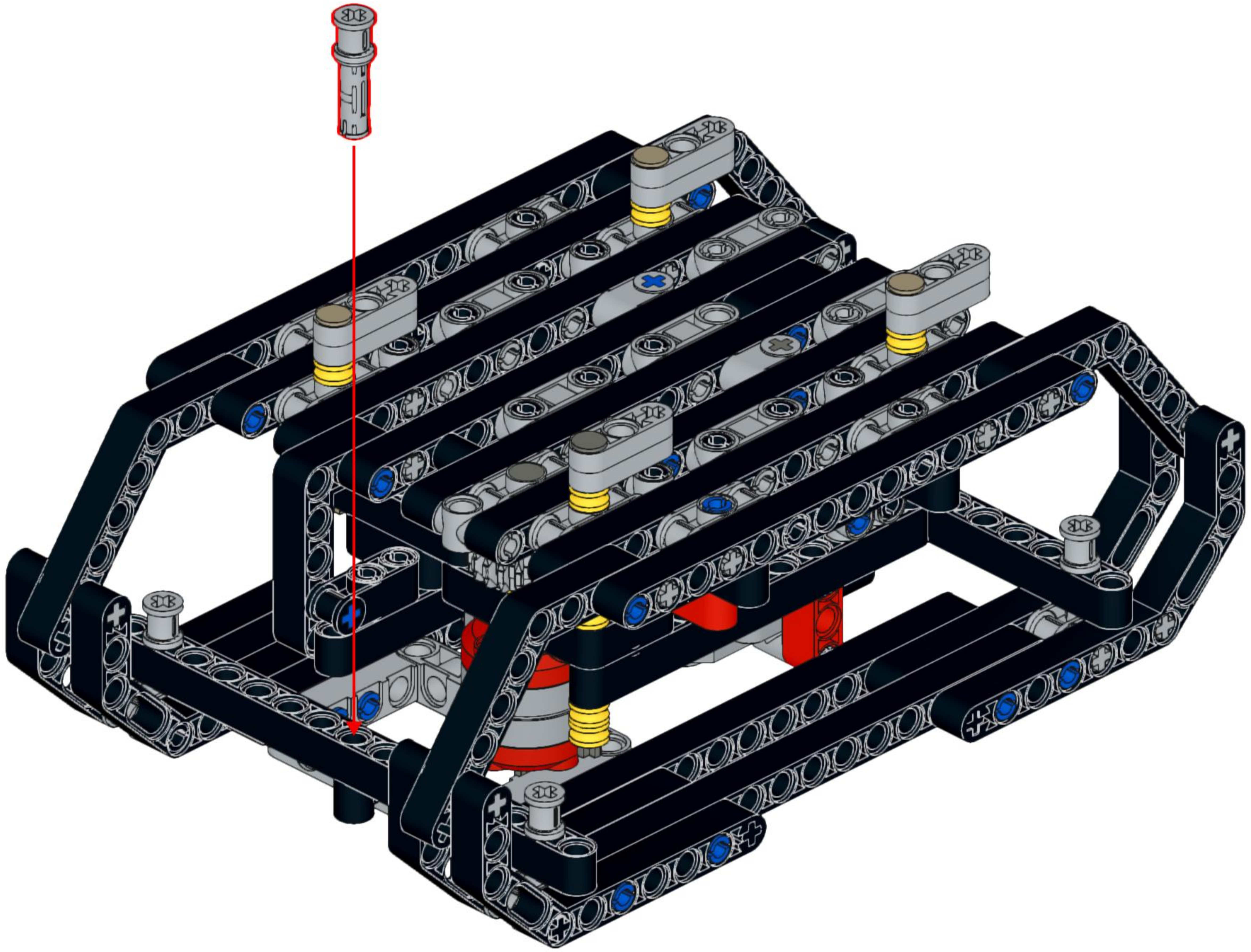

72

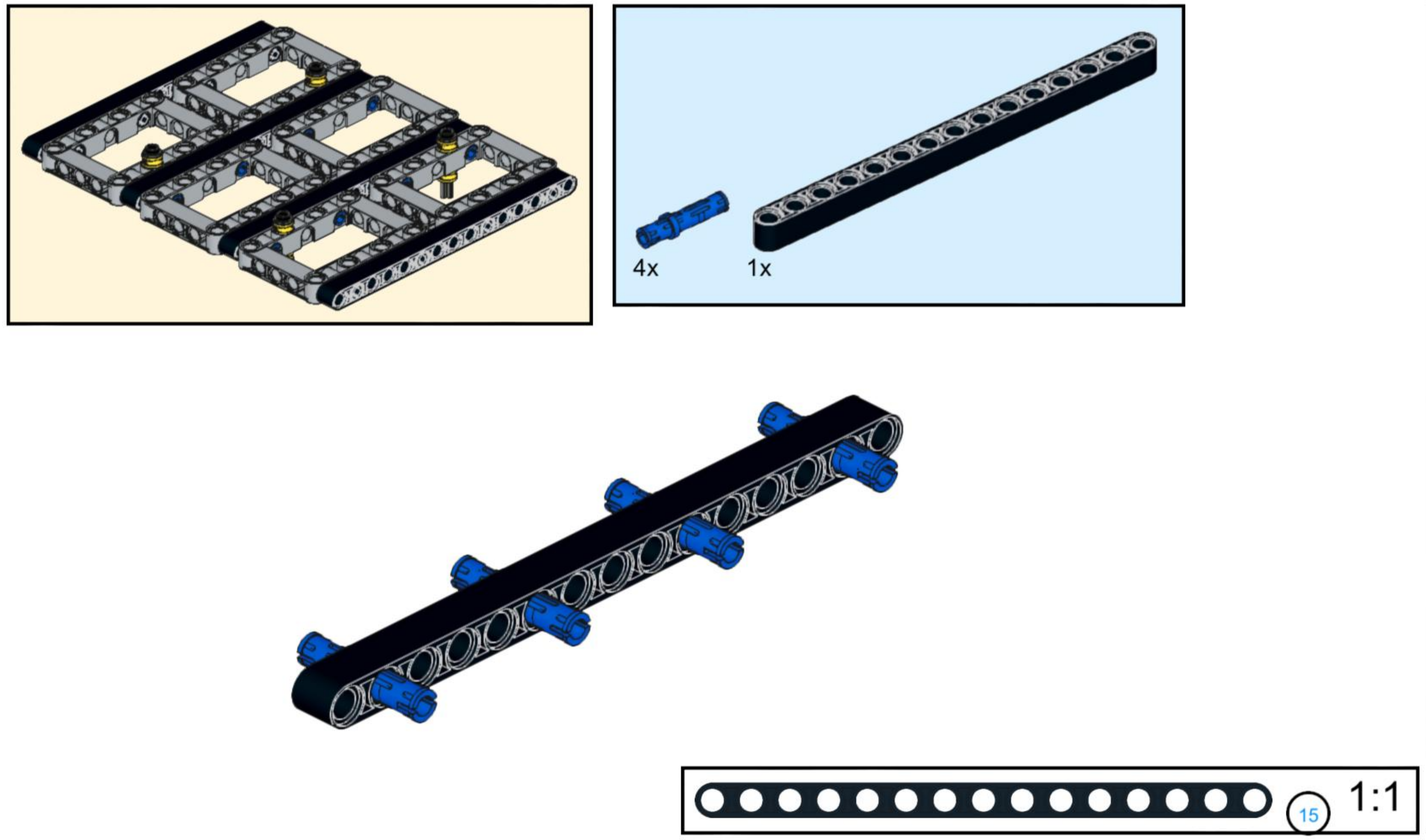


73

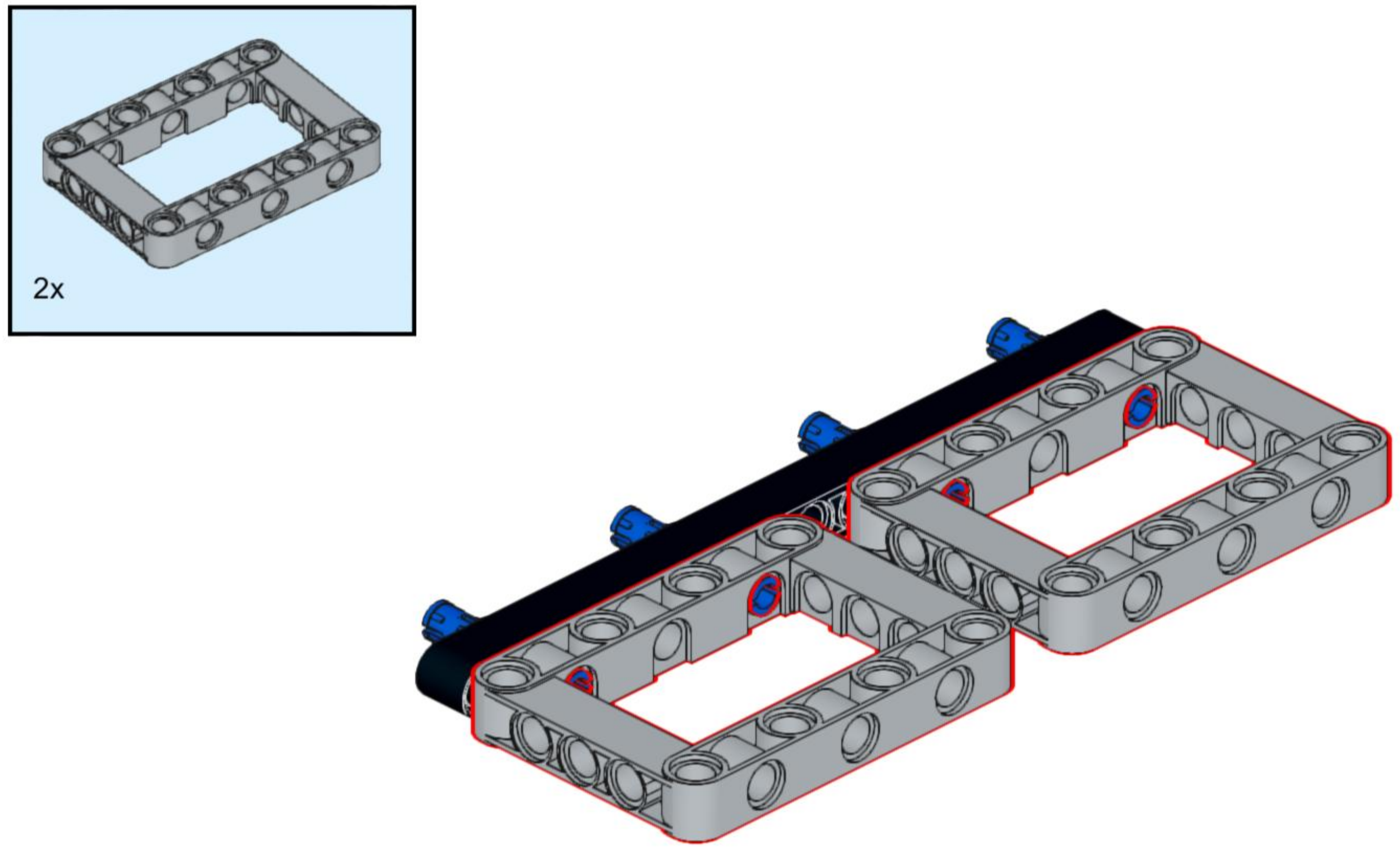


74

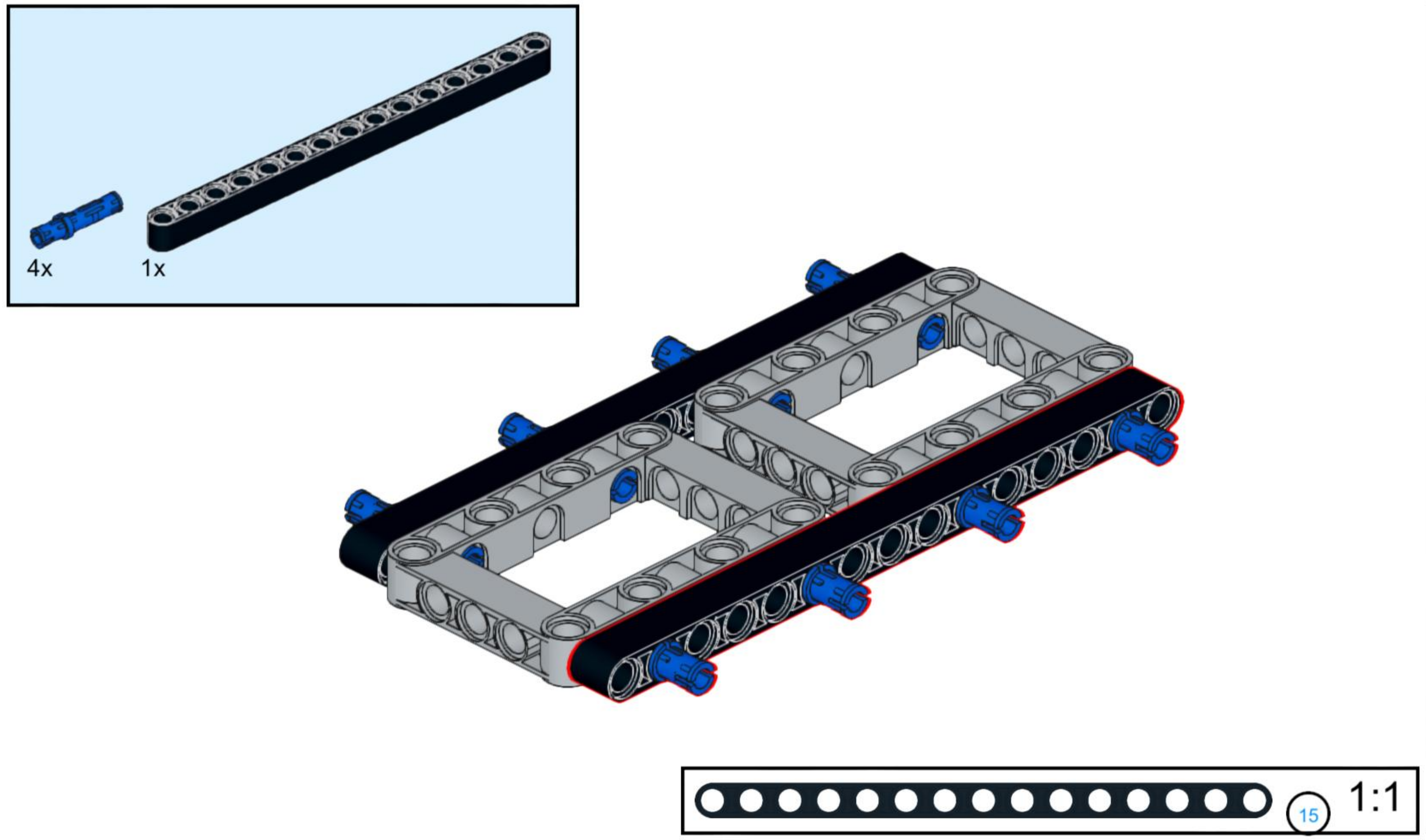


75

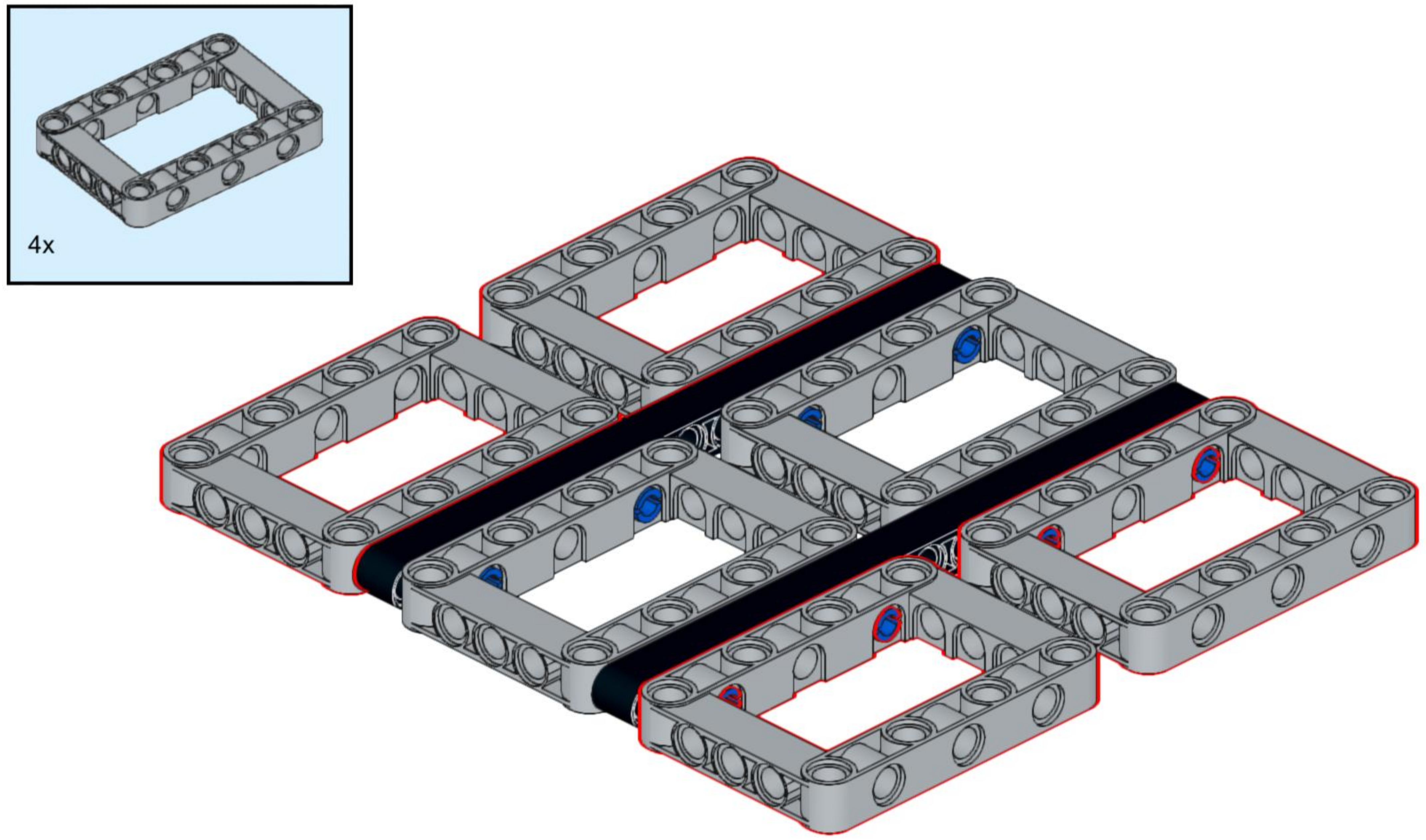

# 76

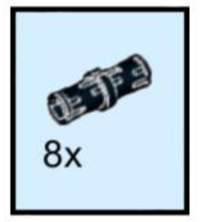


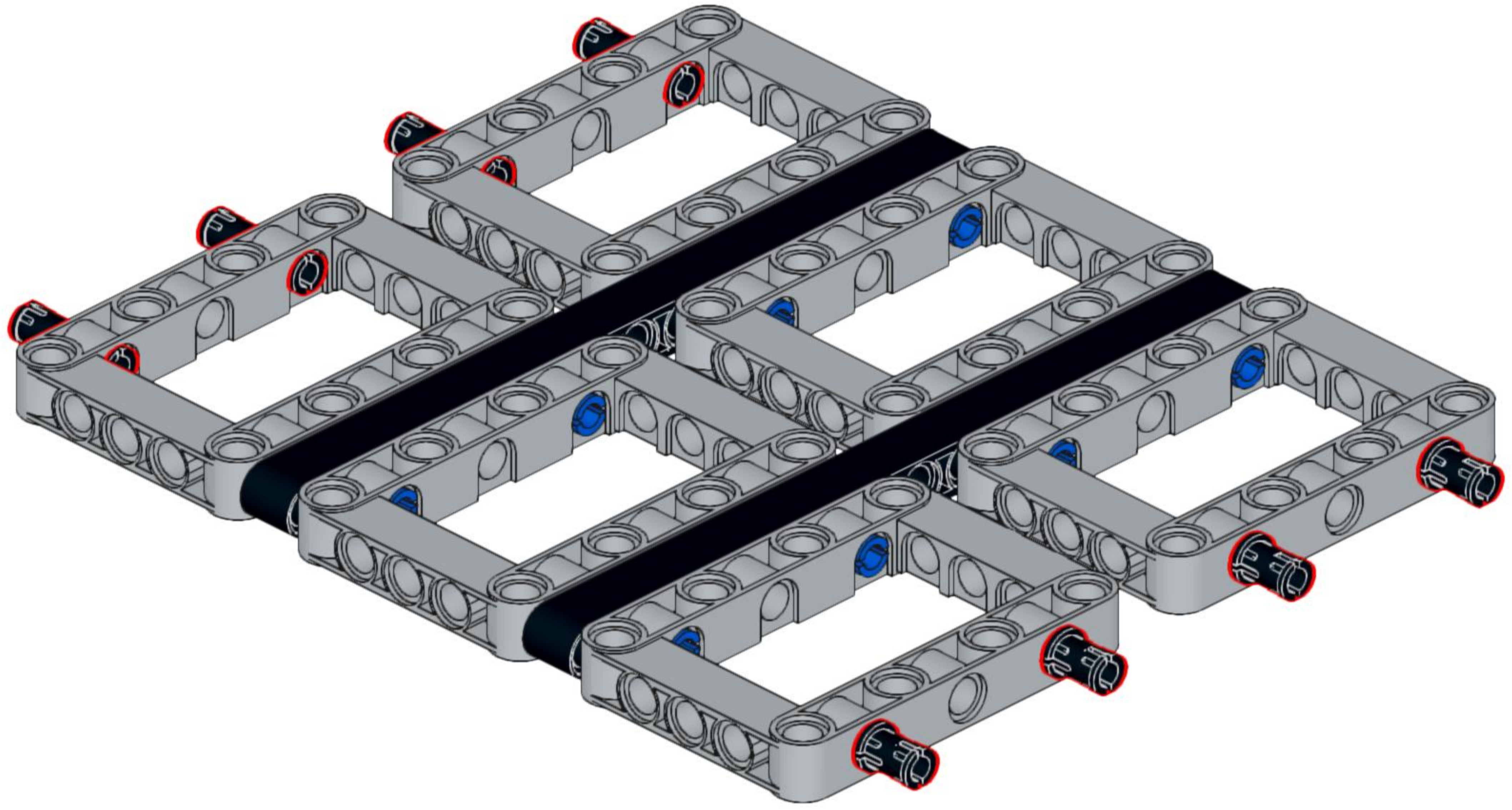

# 77

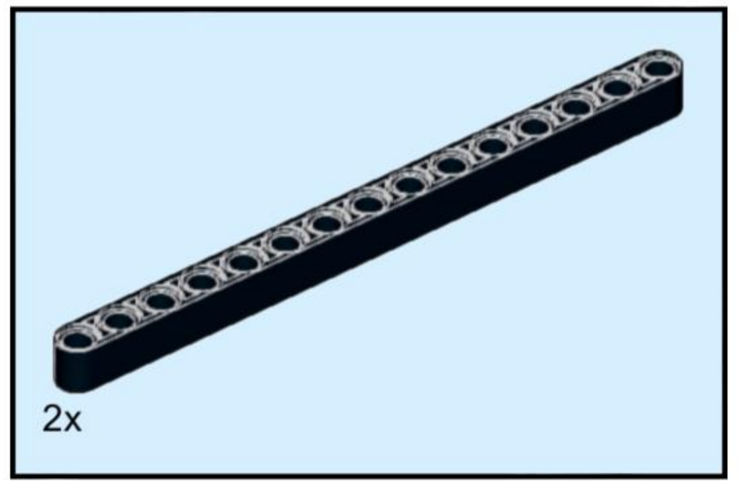


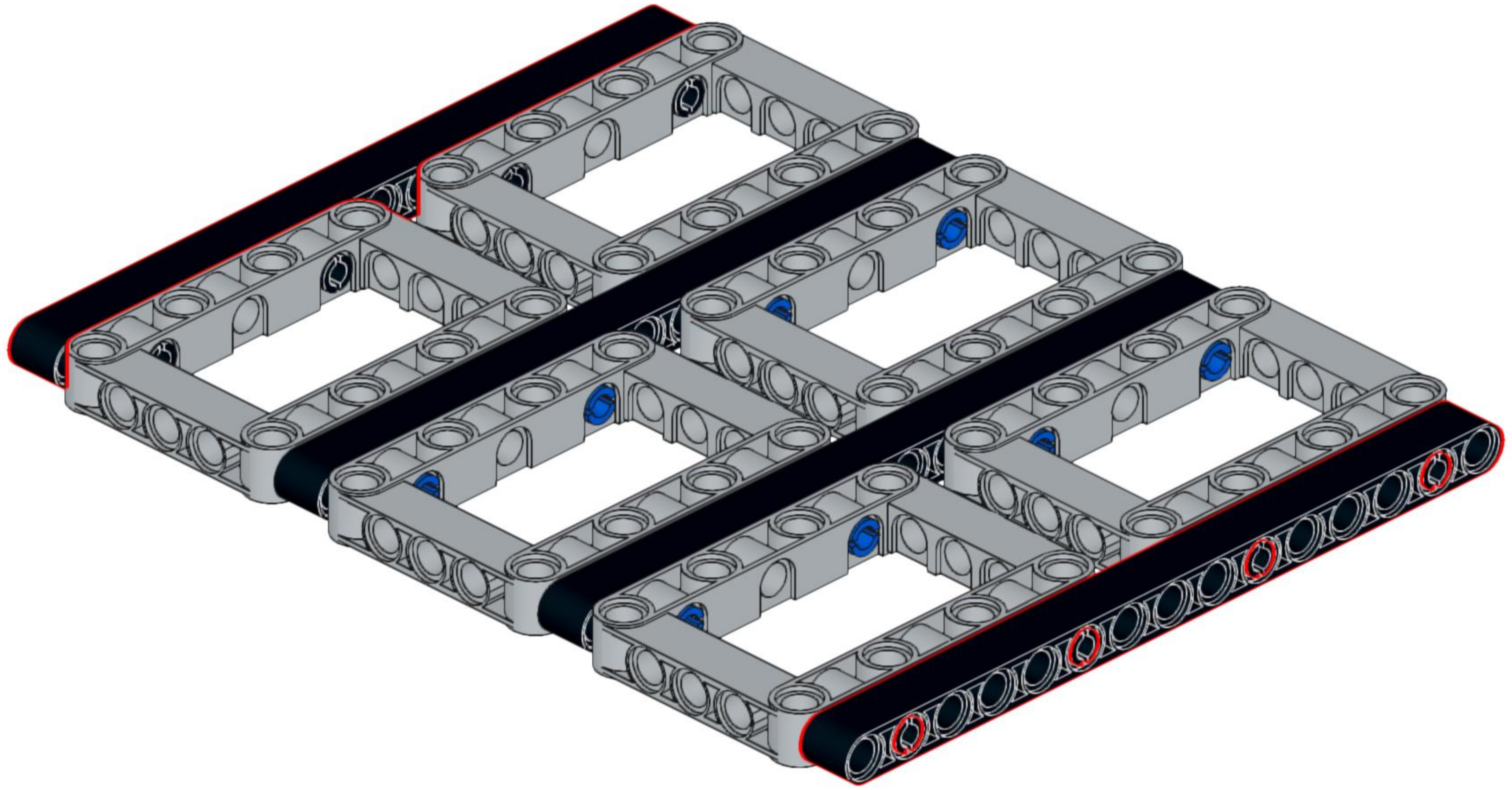

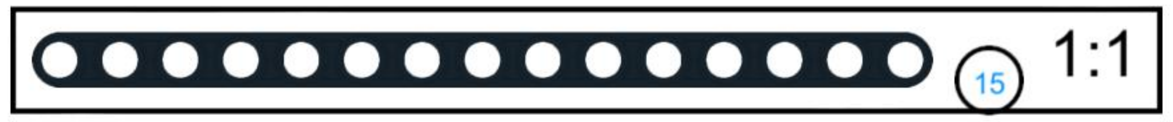

78

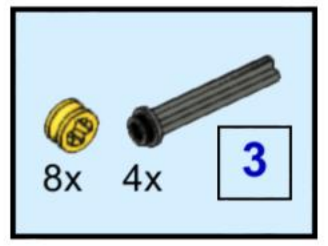


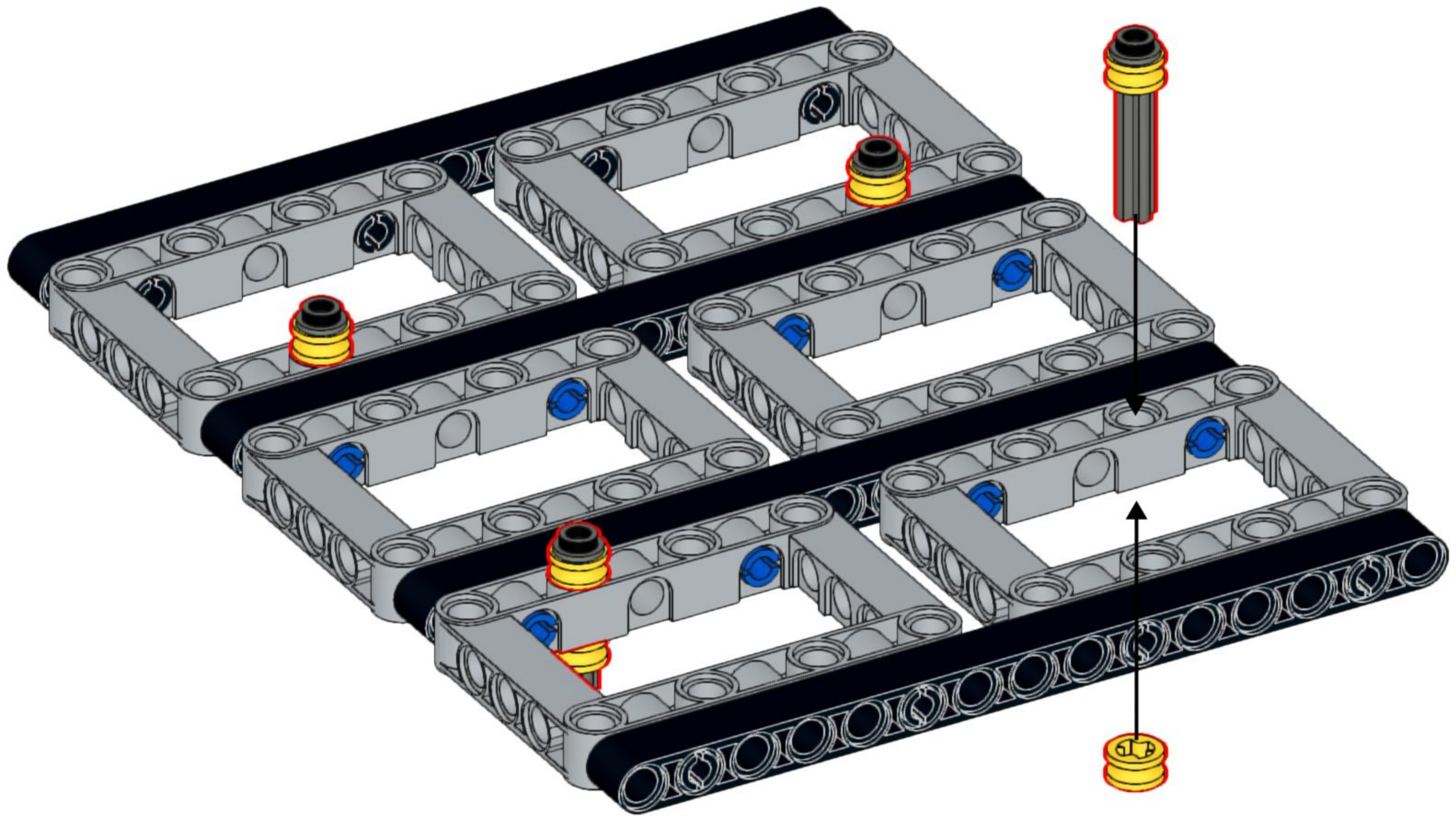

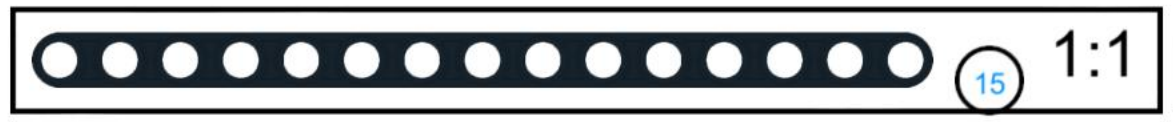


79

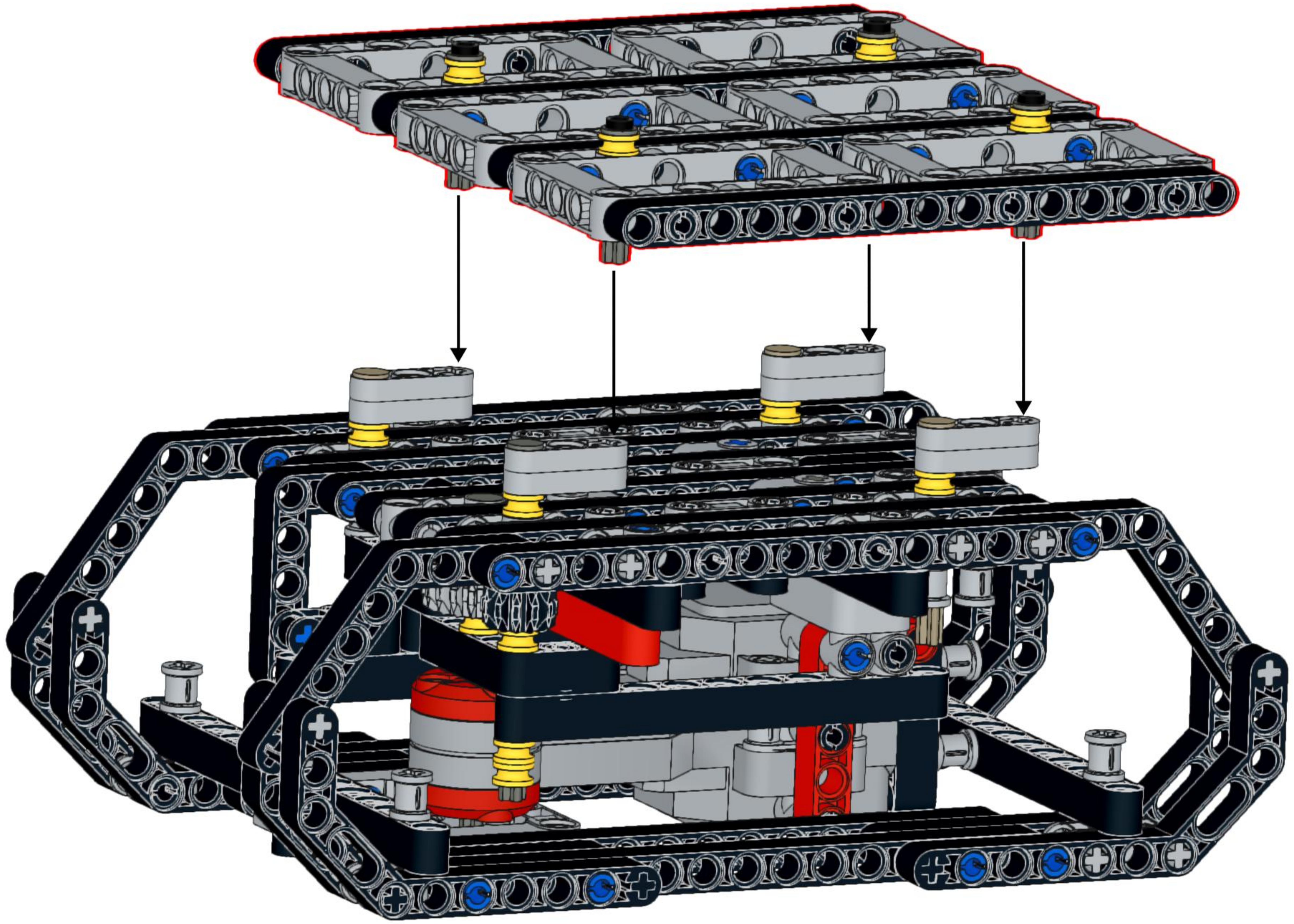

80

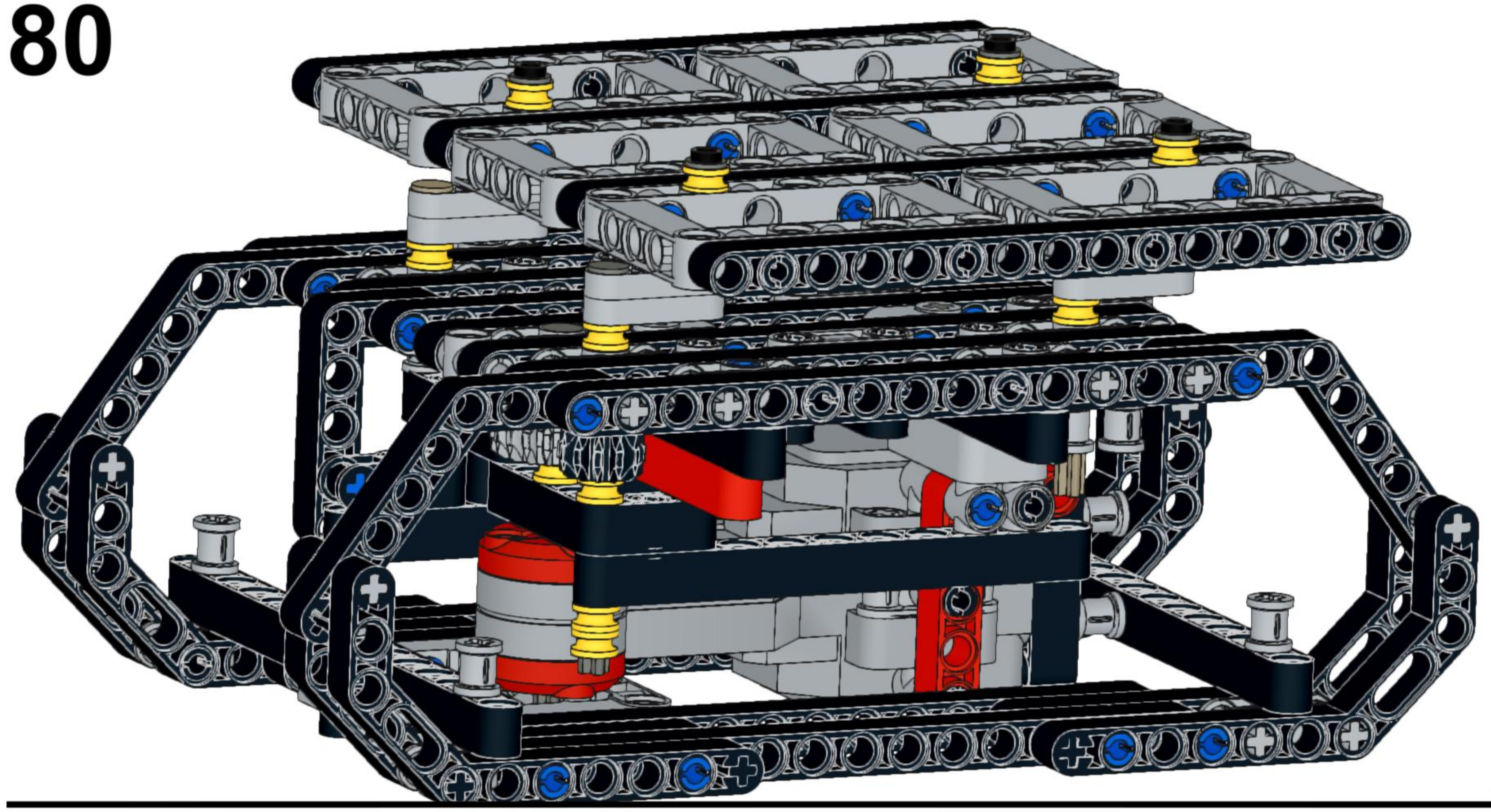

81

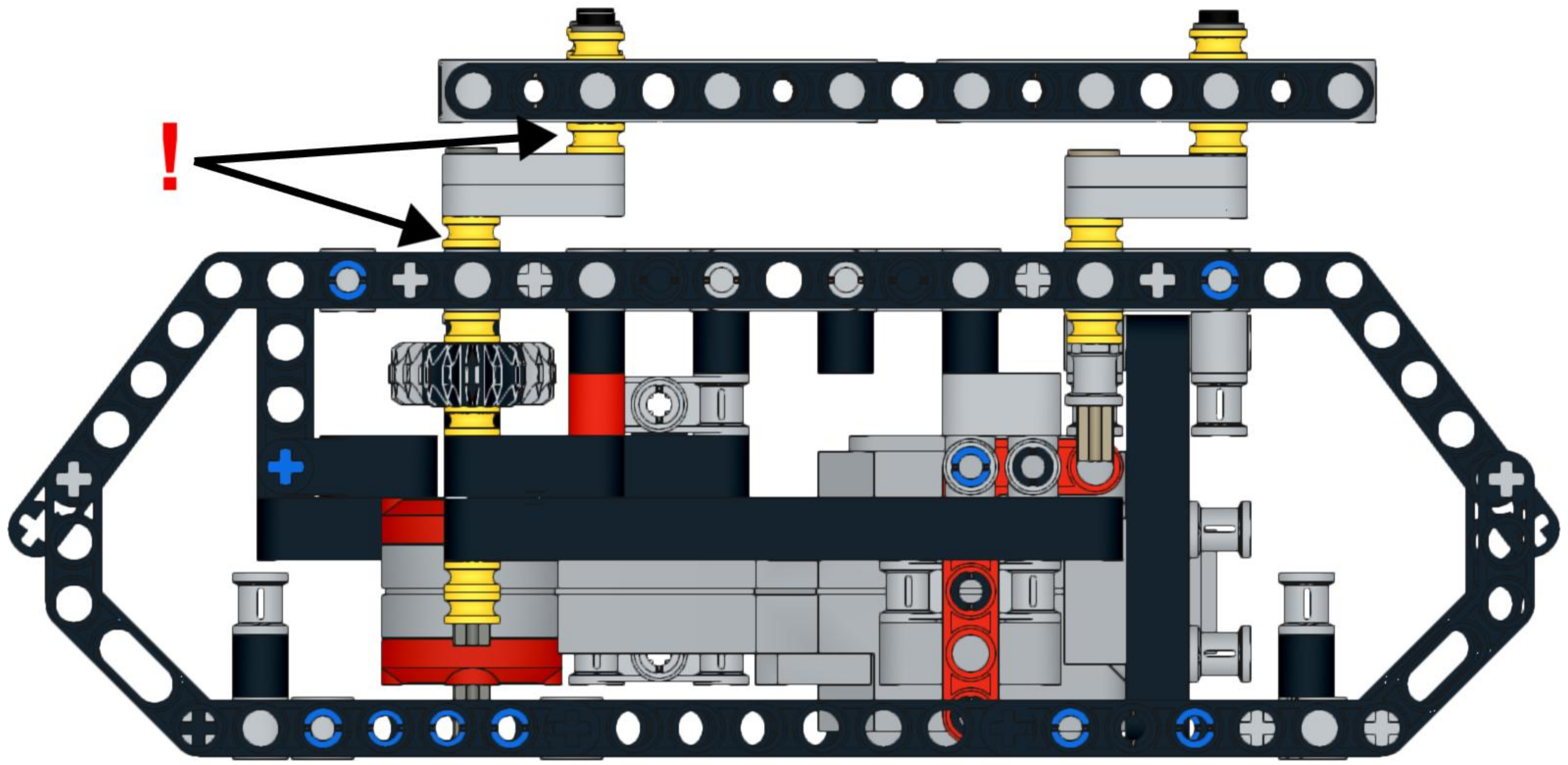


82

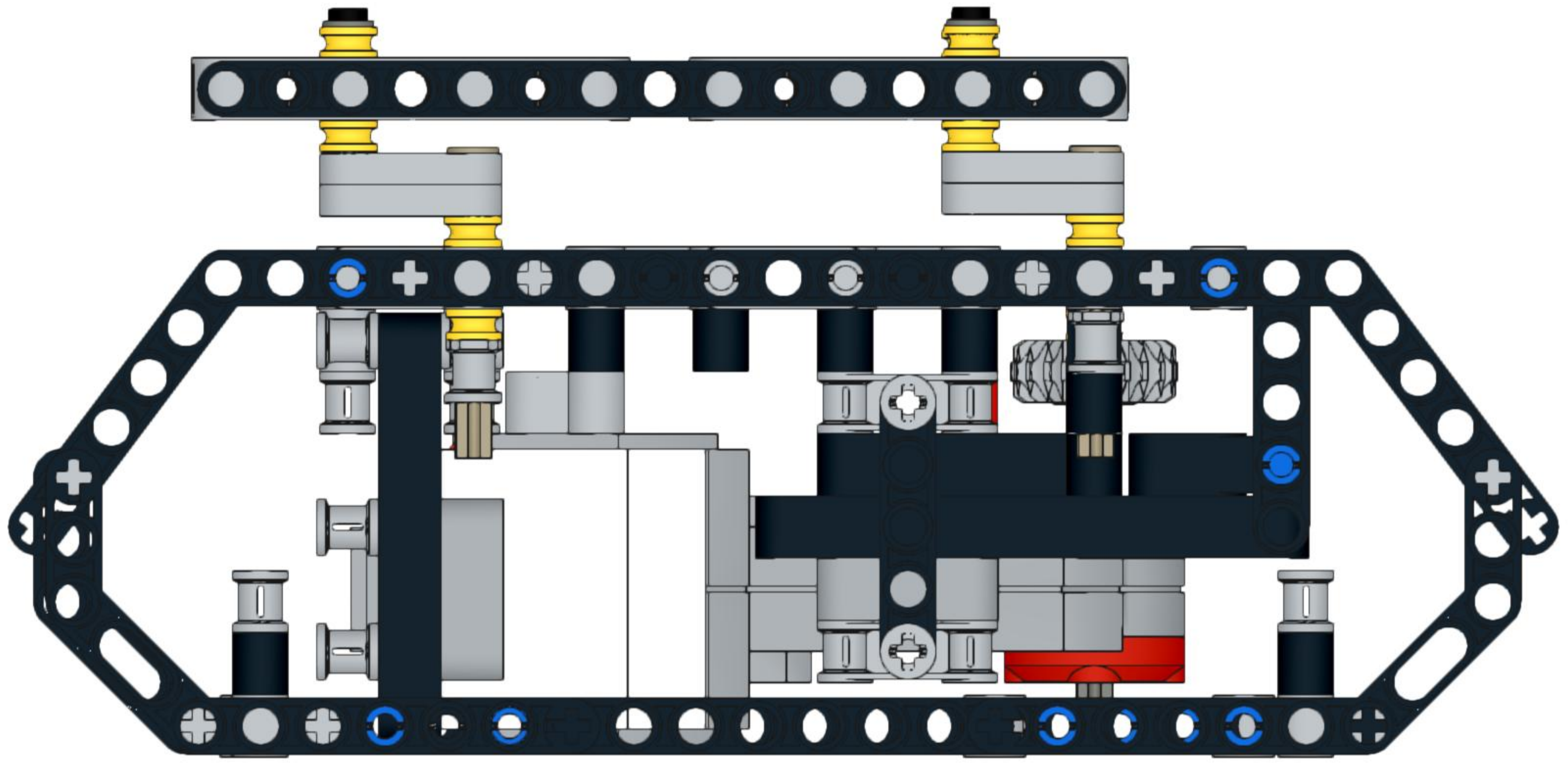

83

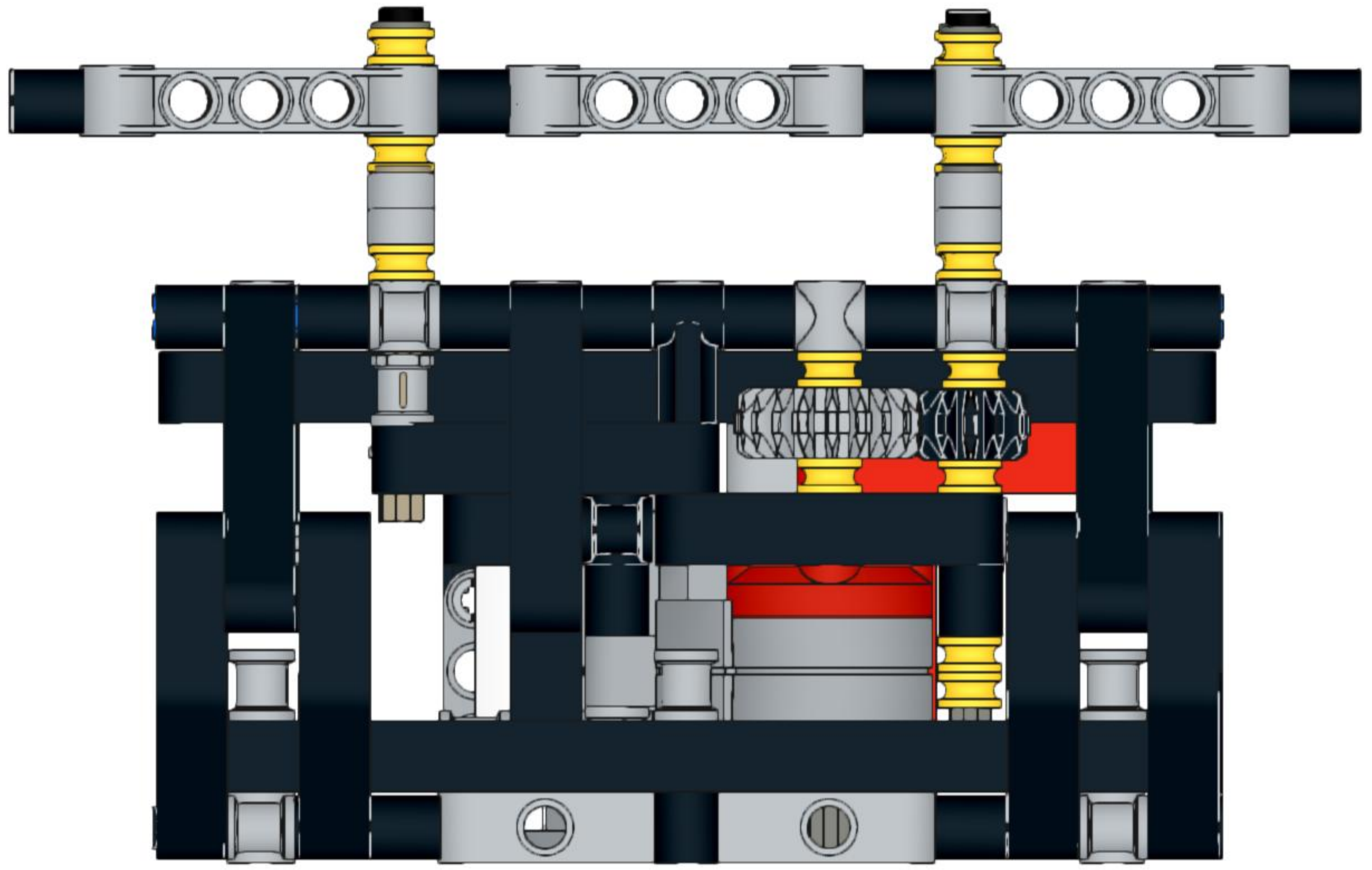

# 84

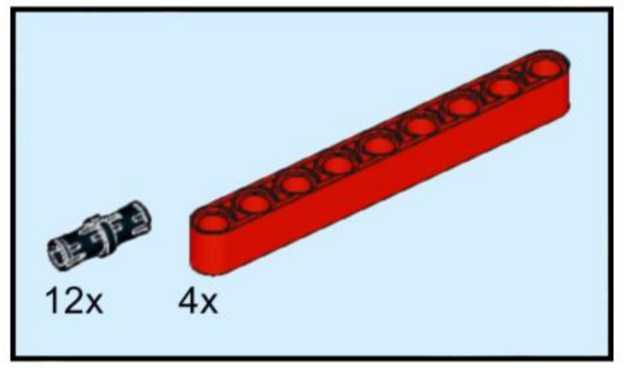


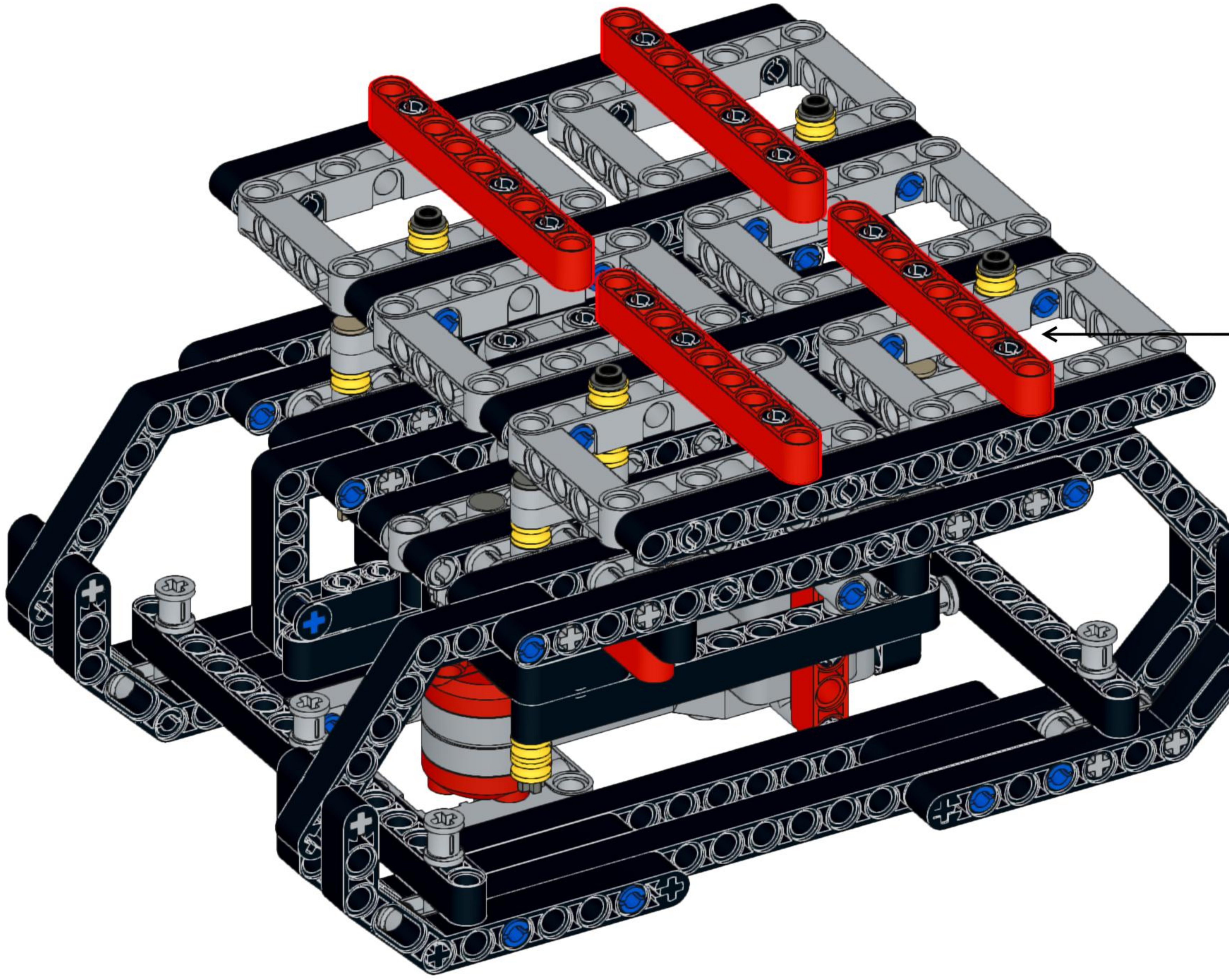

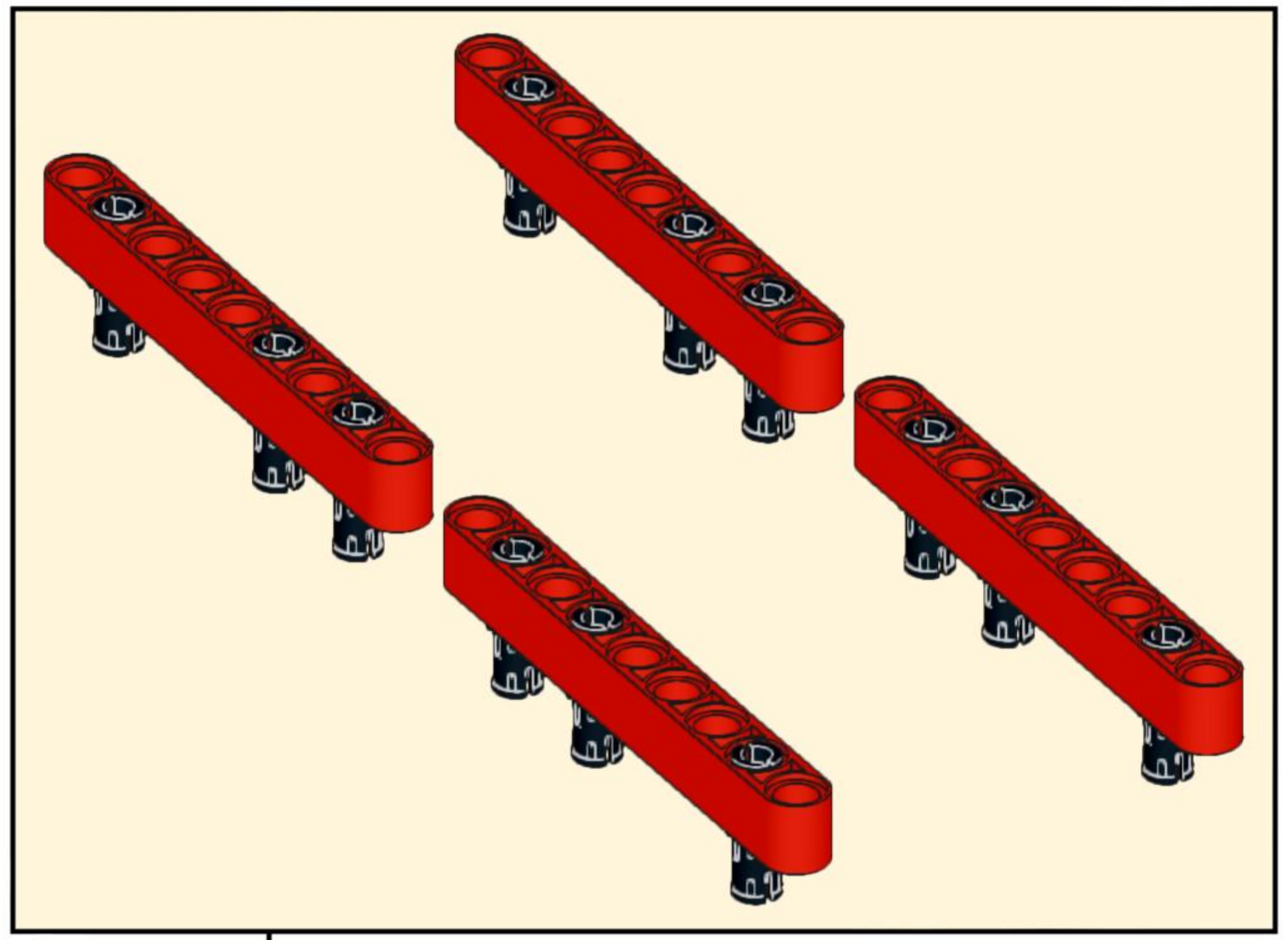

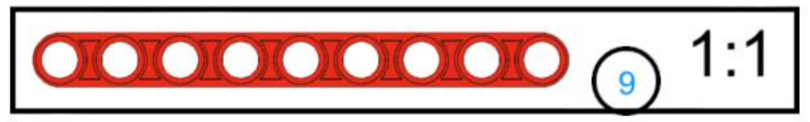

# 85

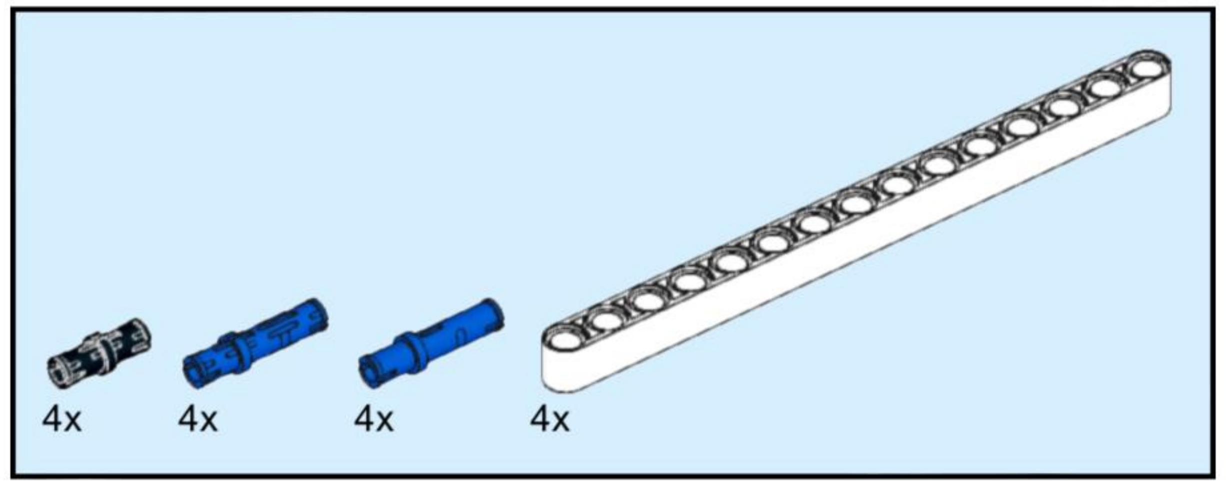


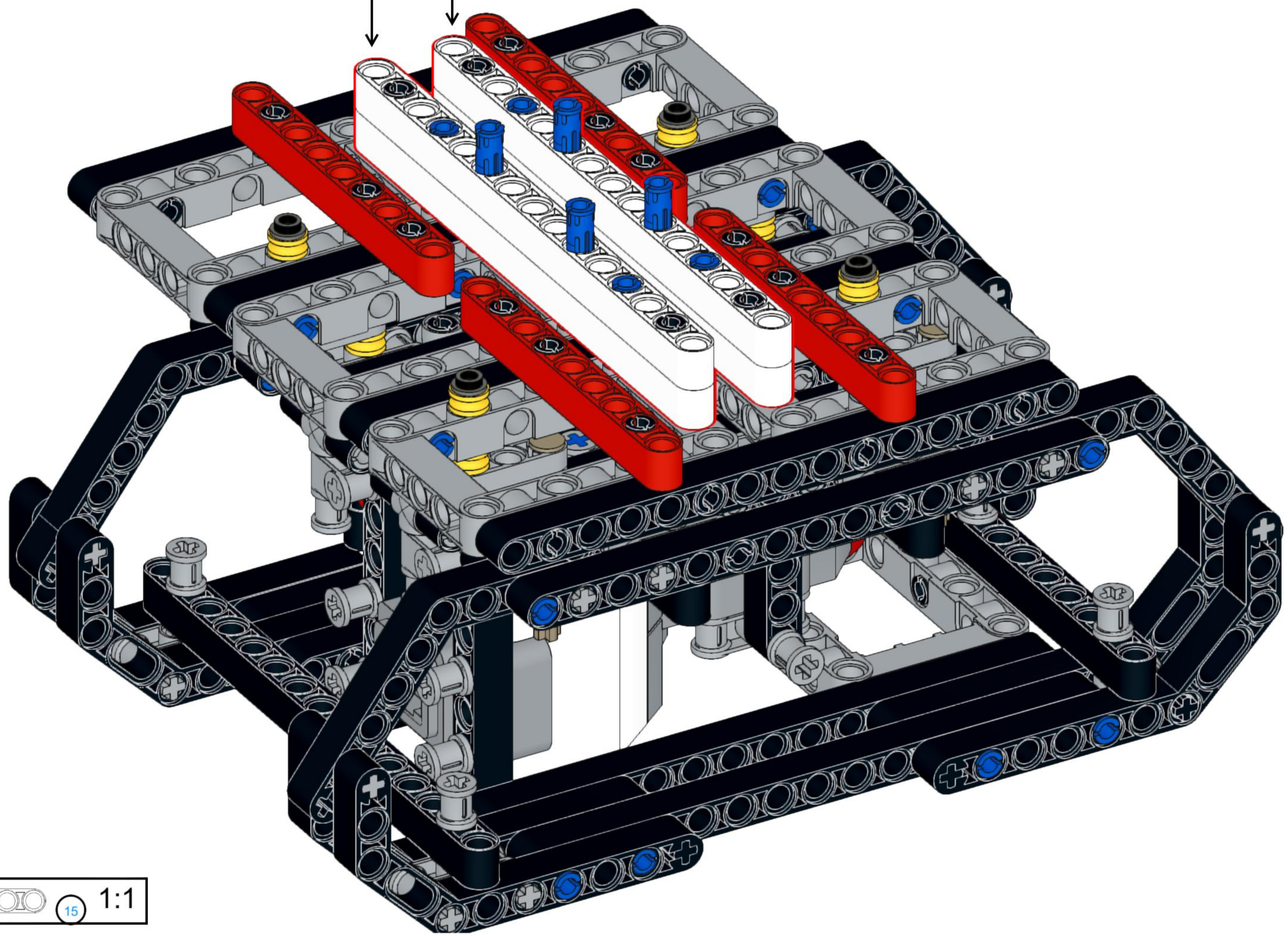

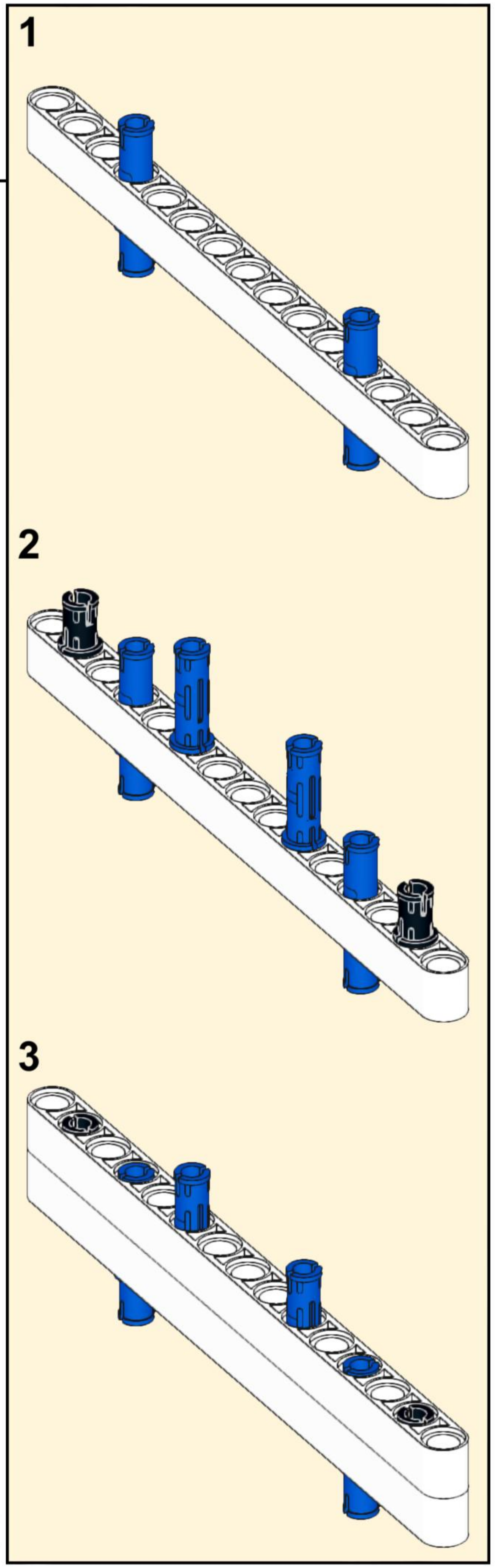


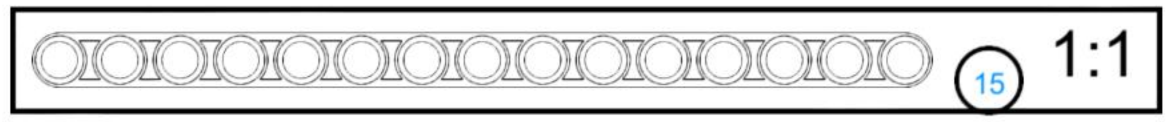

## 86

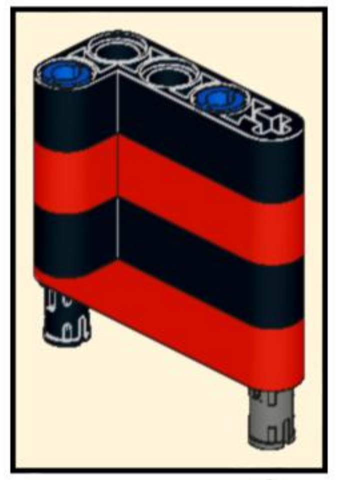

2x

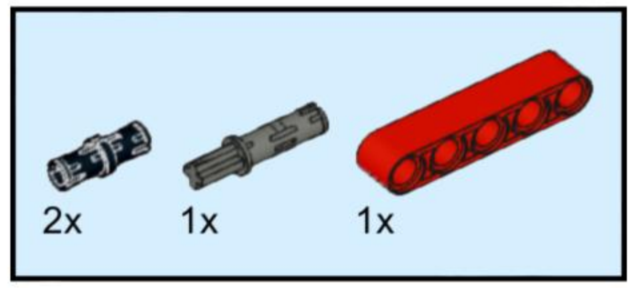


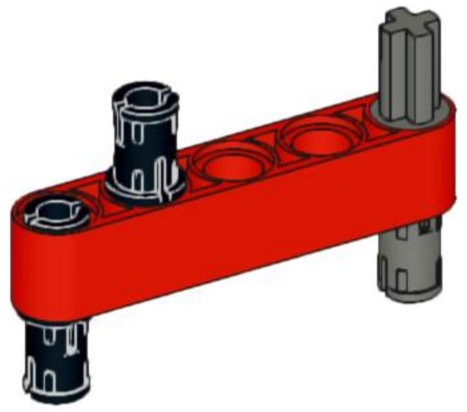

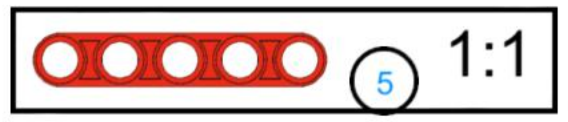


## 87

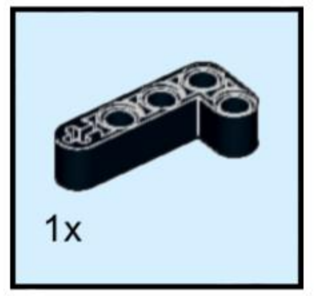


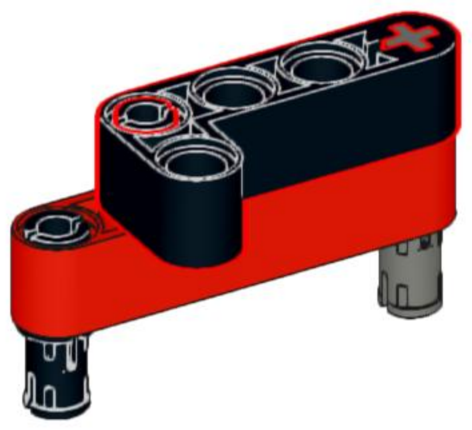

## 88

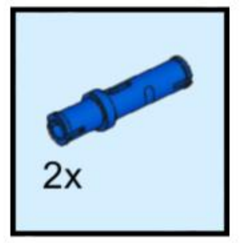


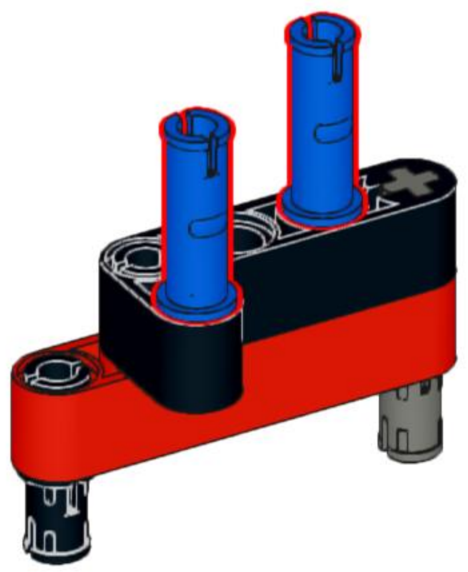

## 89

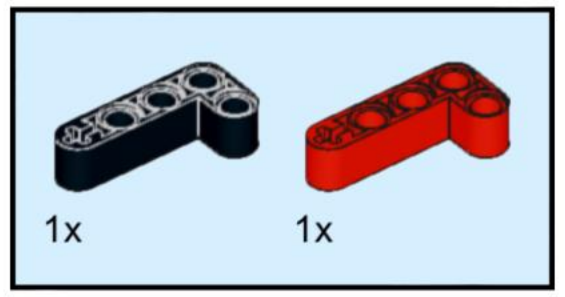


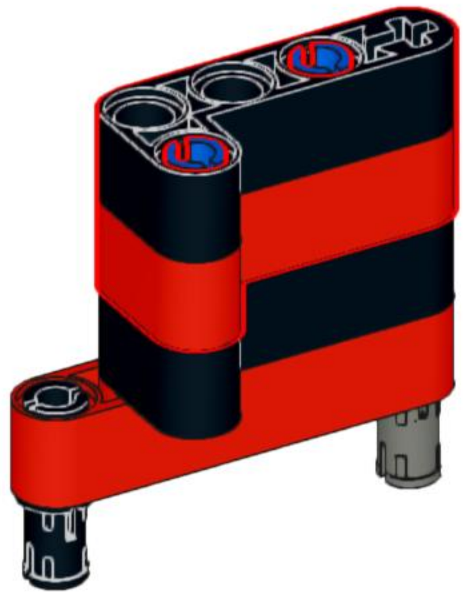

## 90

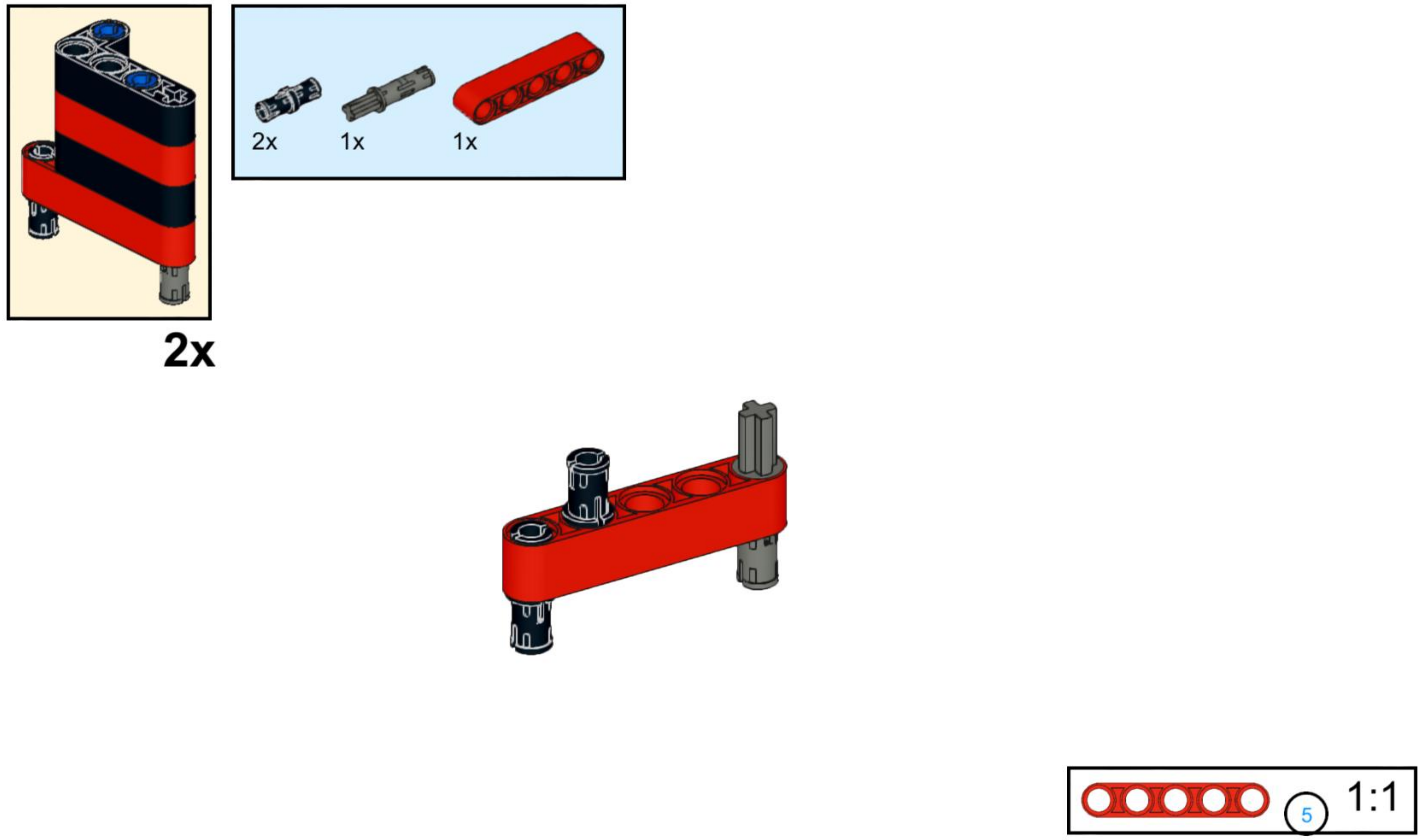


## 91

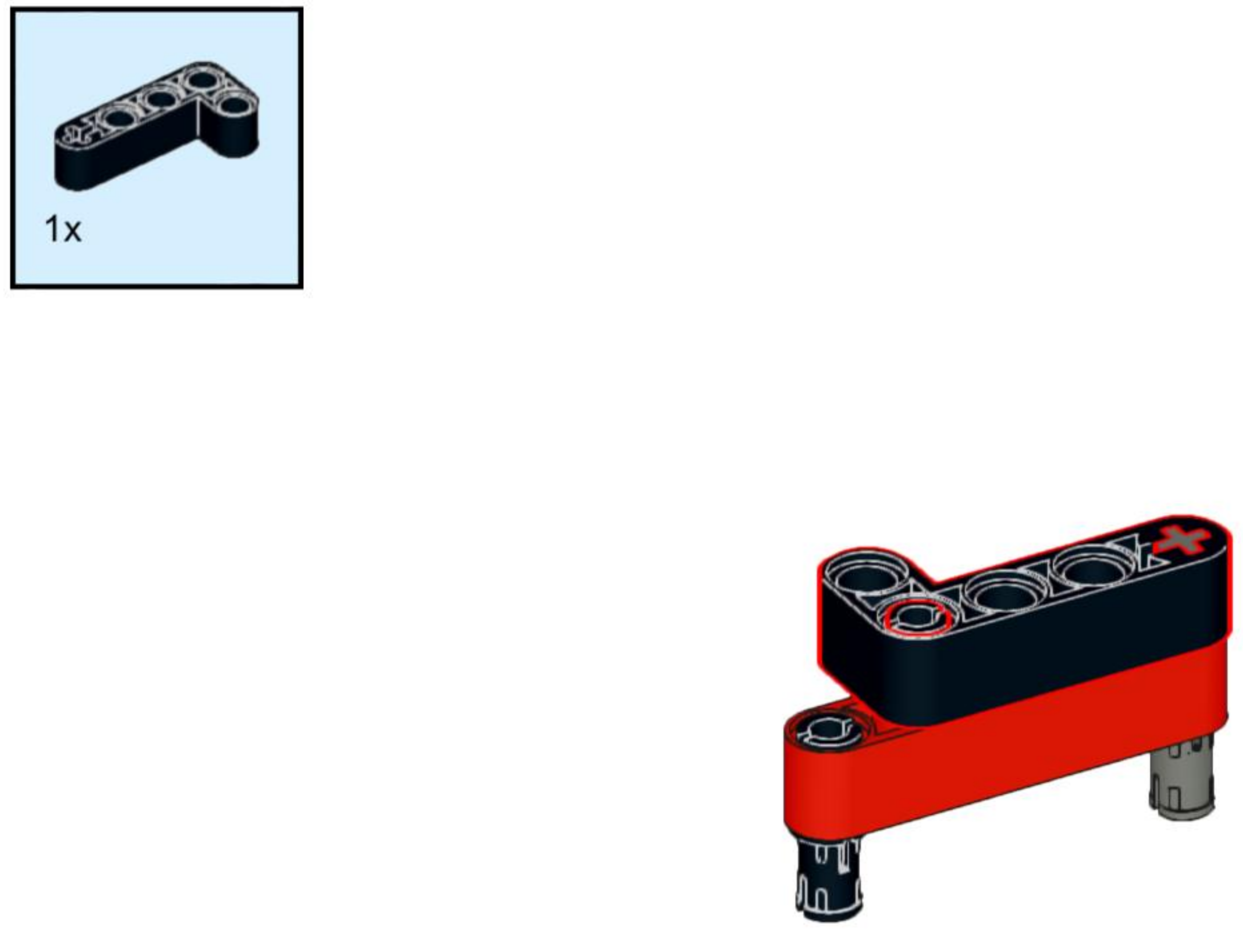


## 92

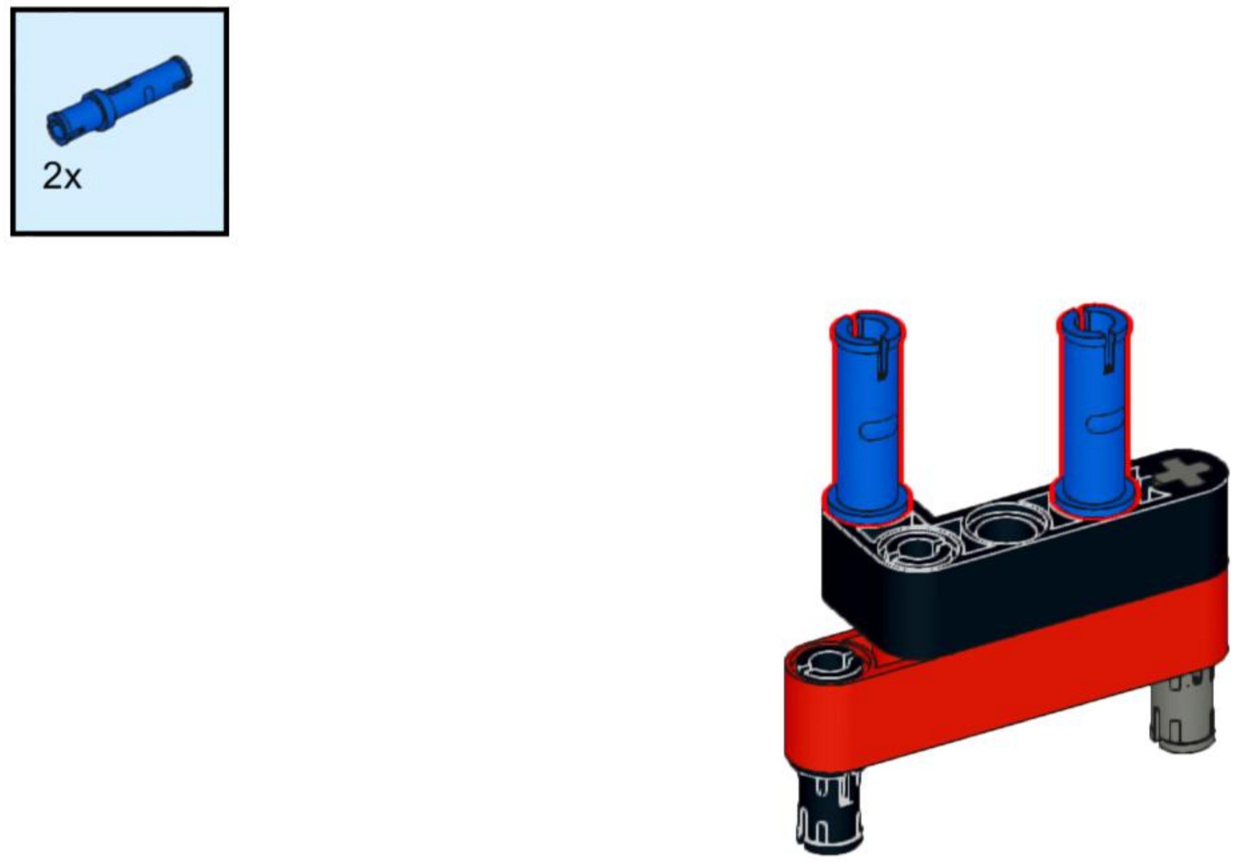


## 93

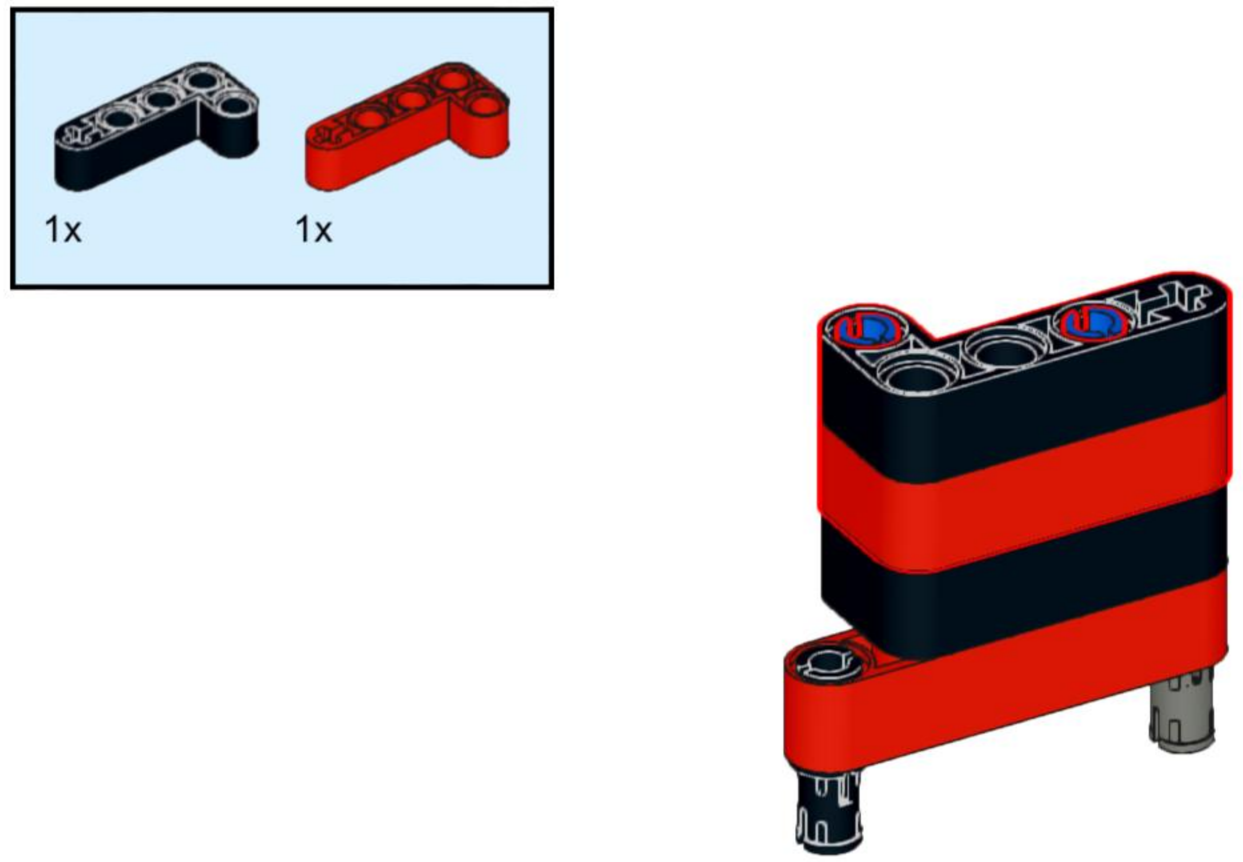

94

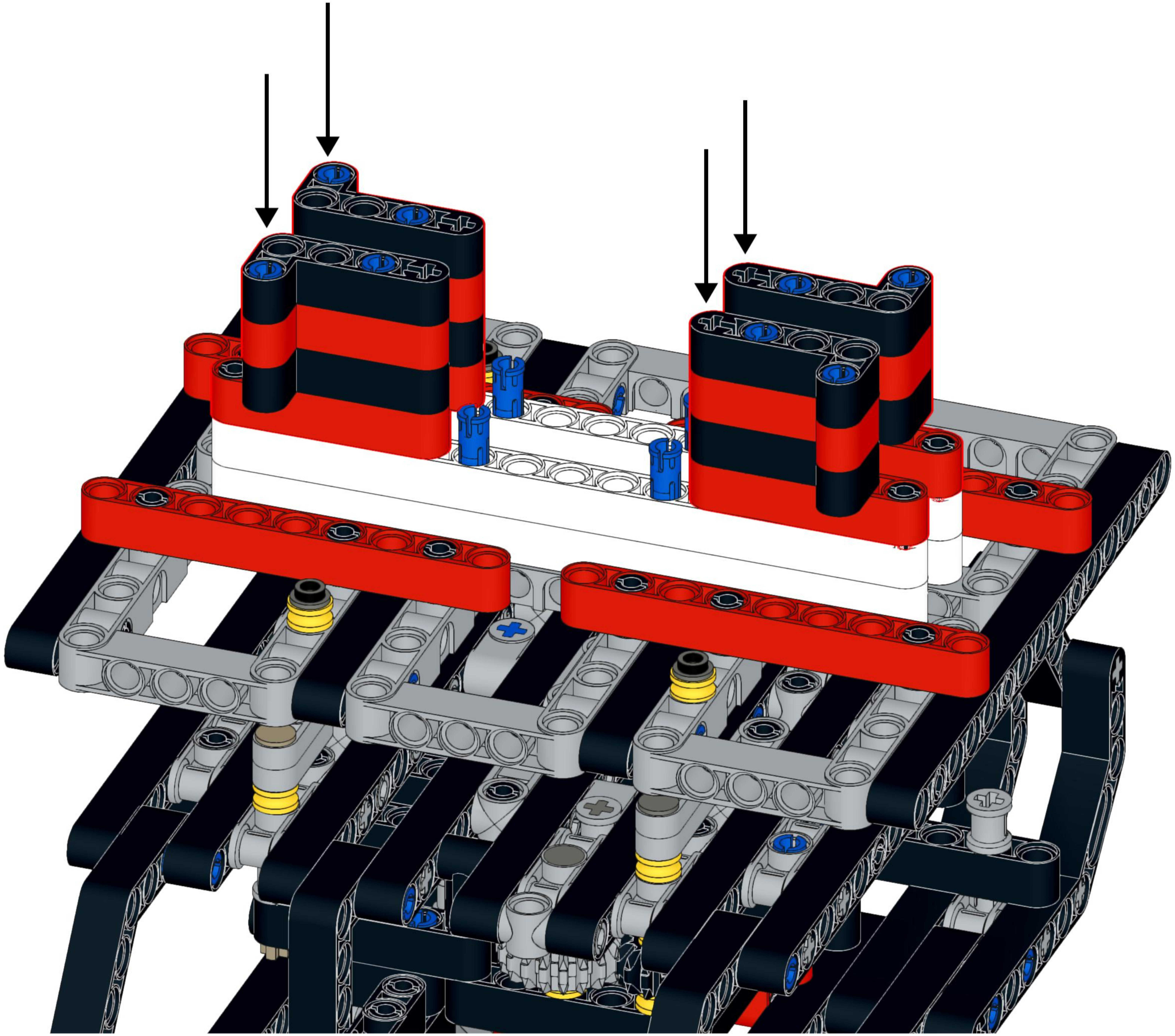

# 95

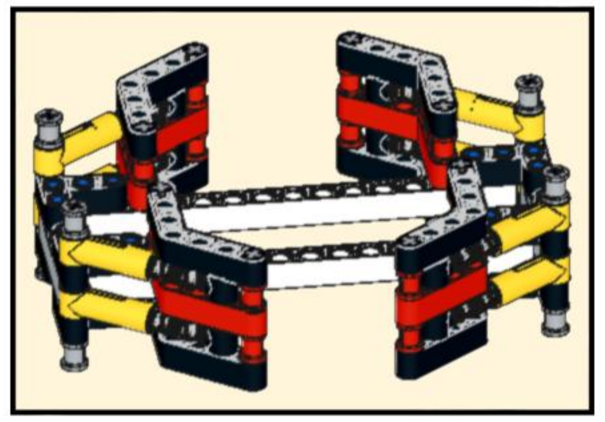

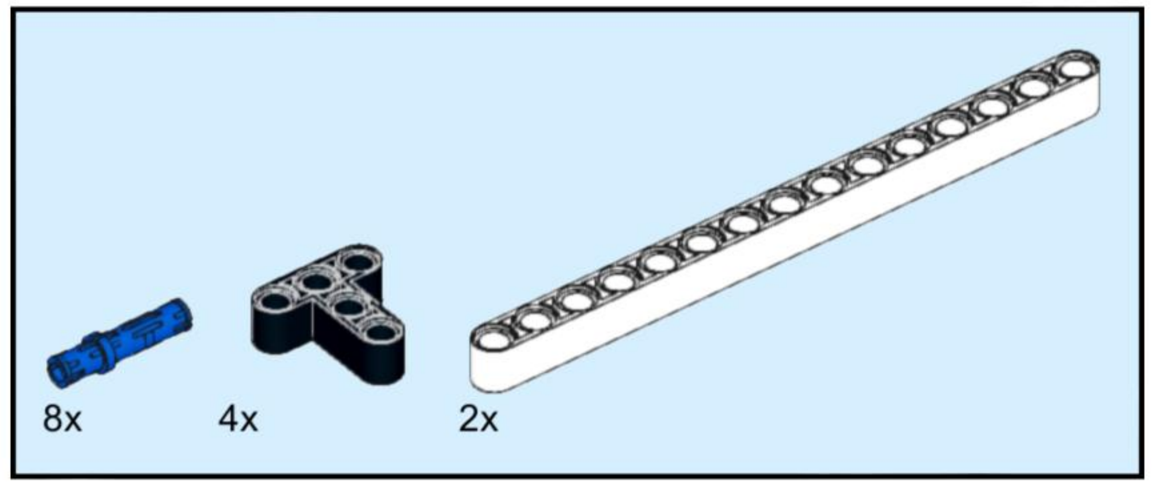


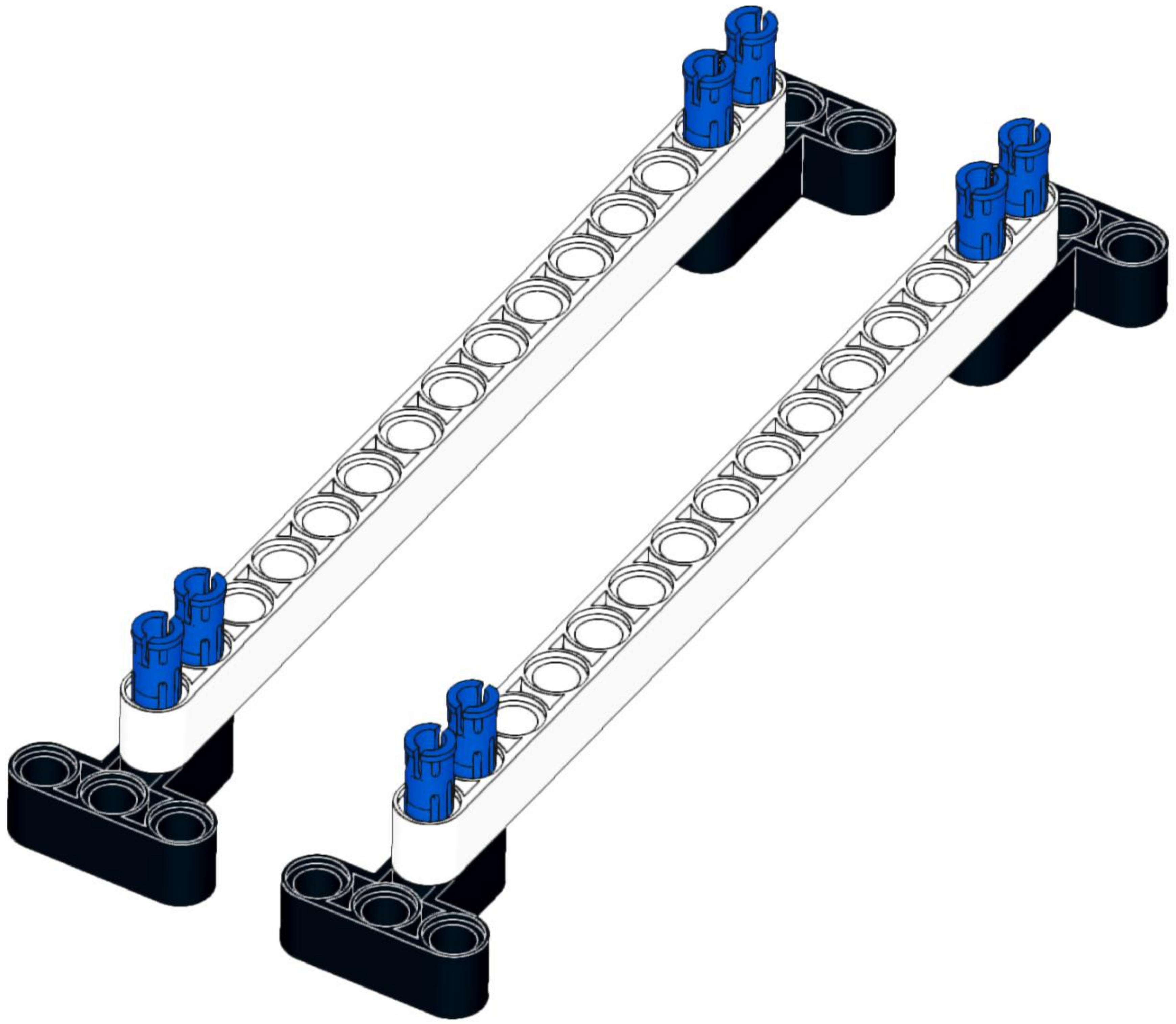

# 96

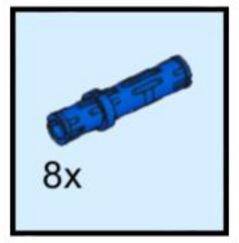


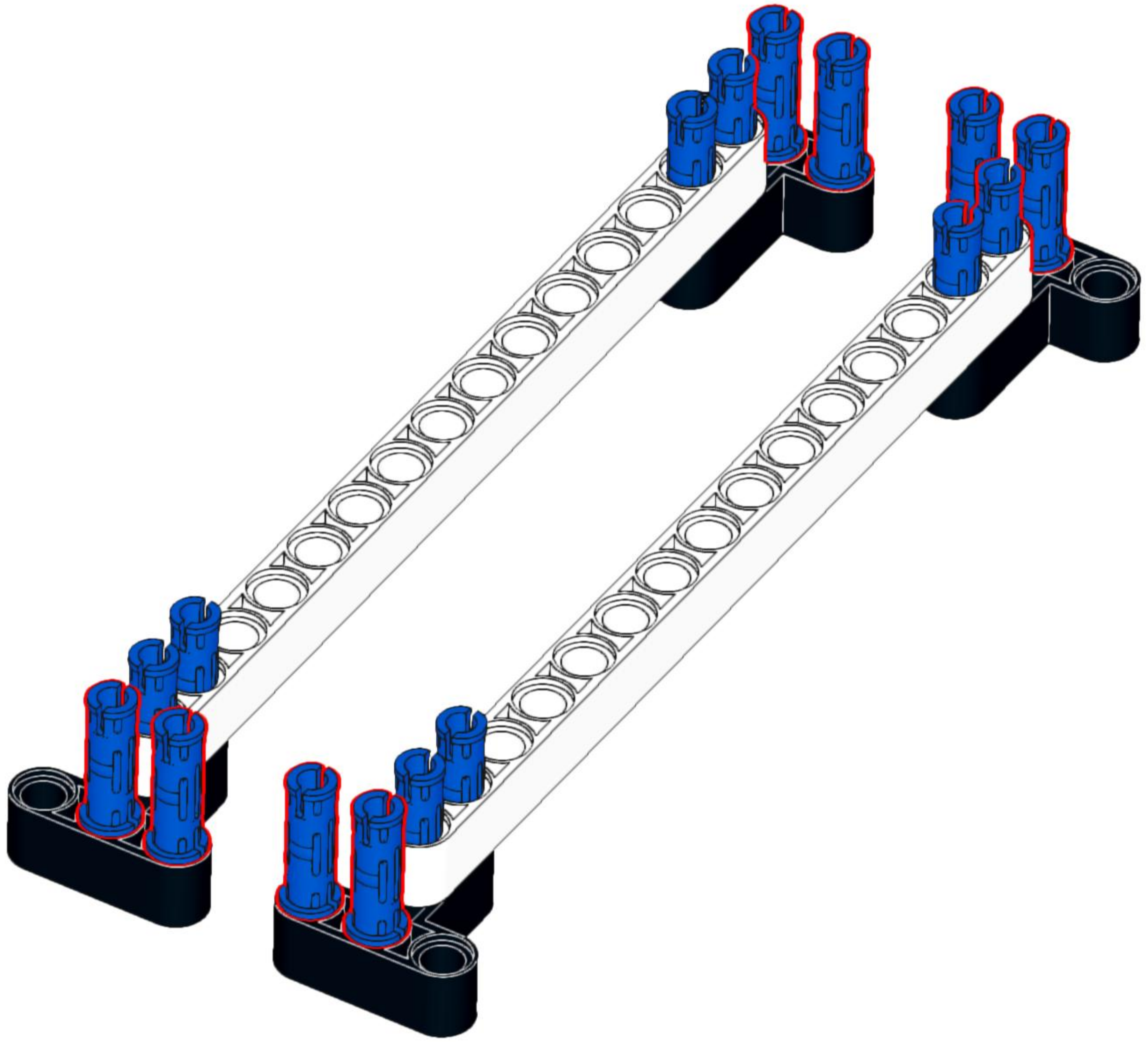

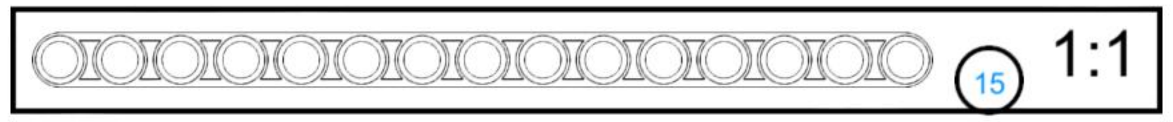

# 97

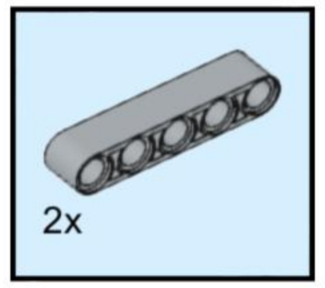


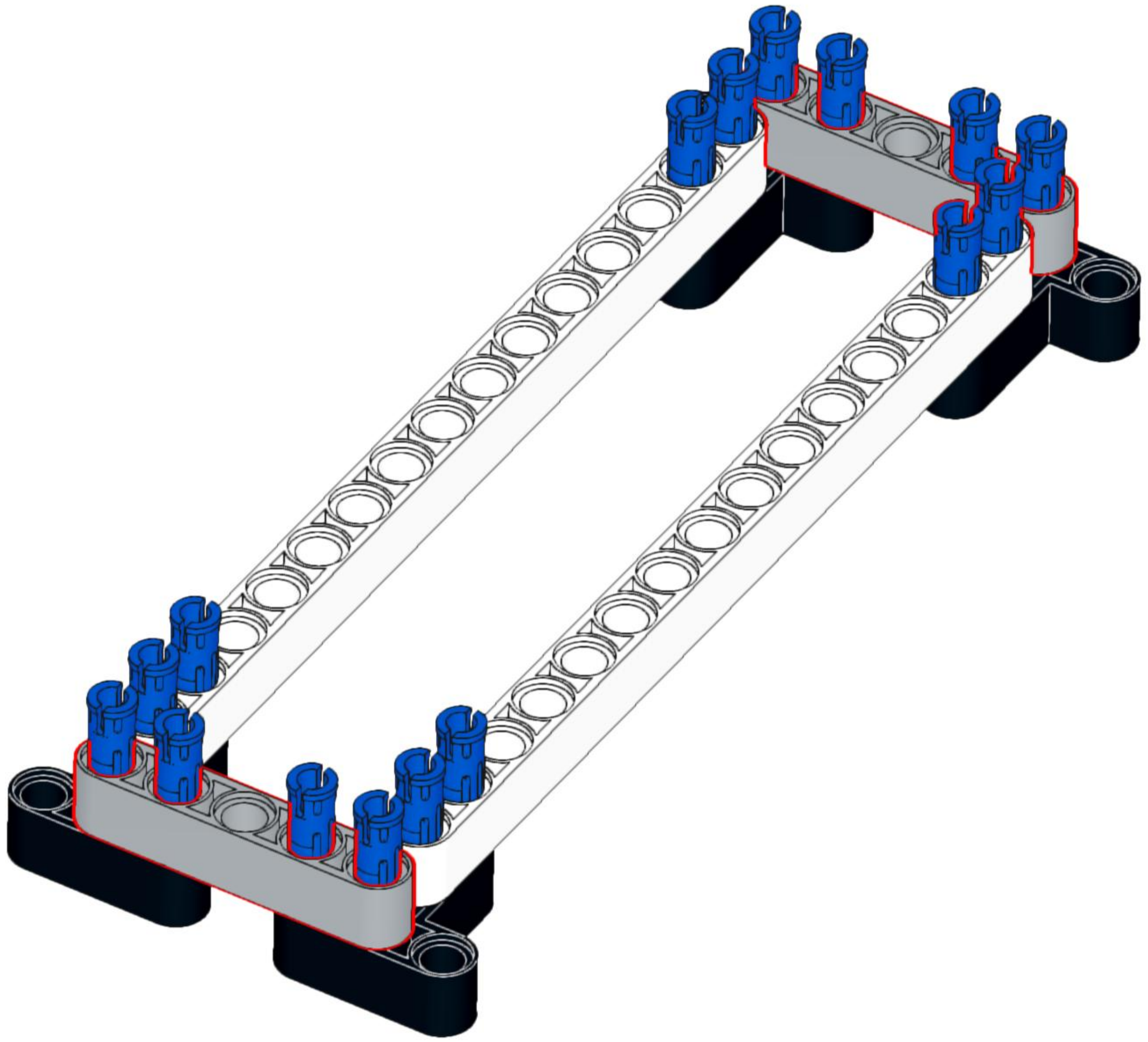

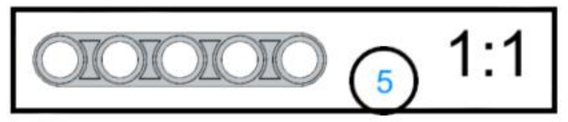


# 98

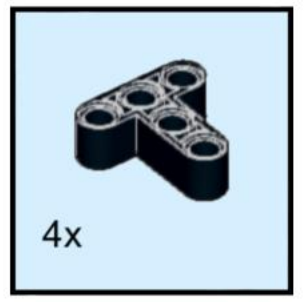


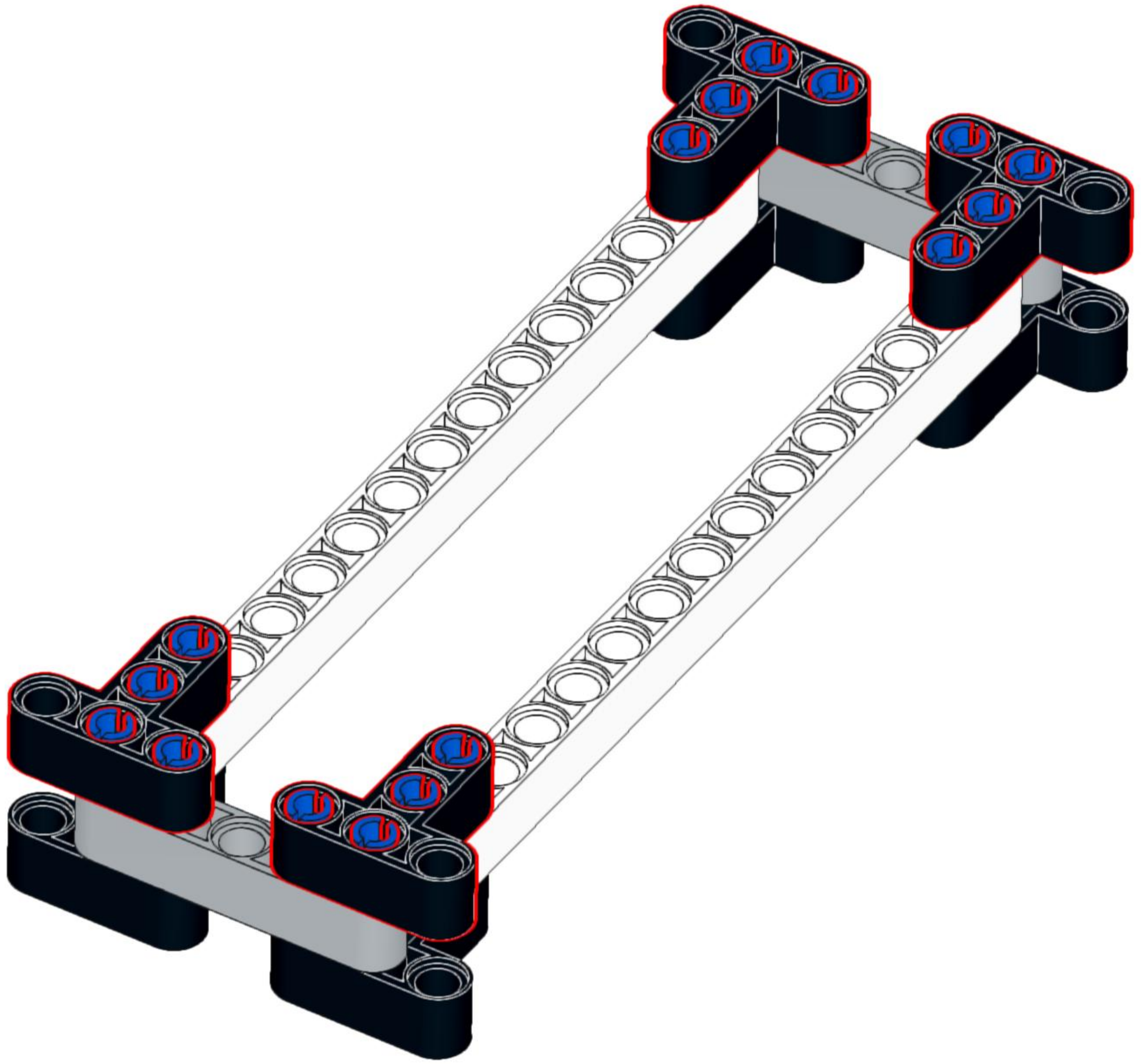

99

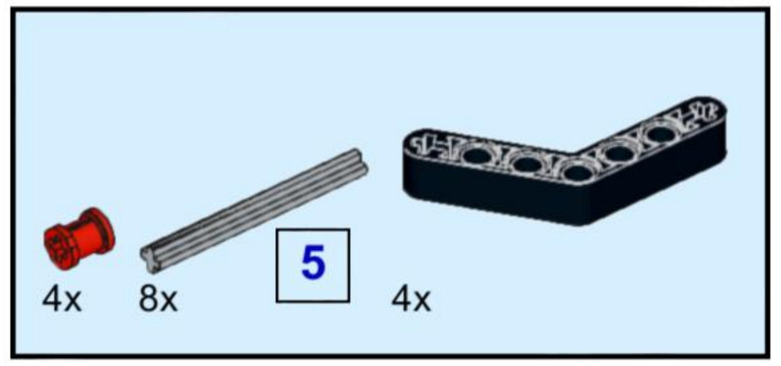


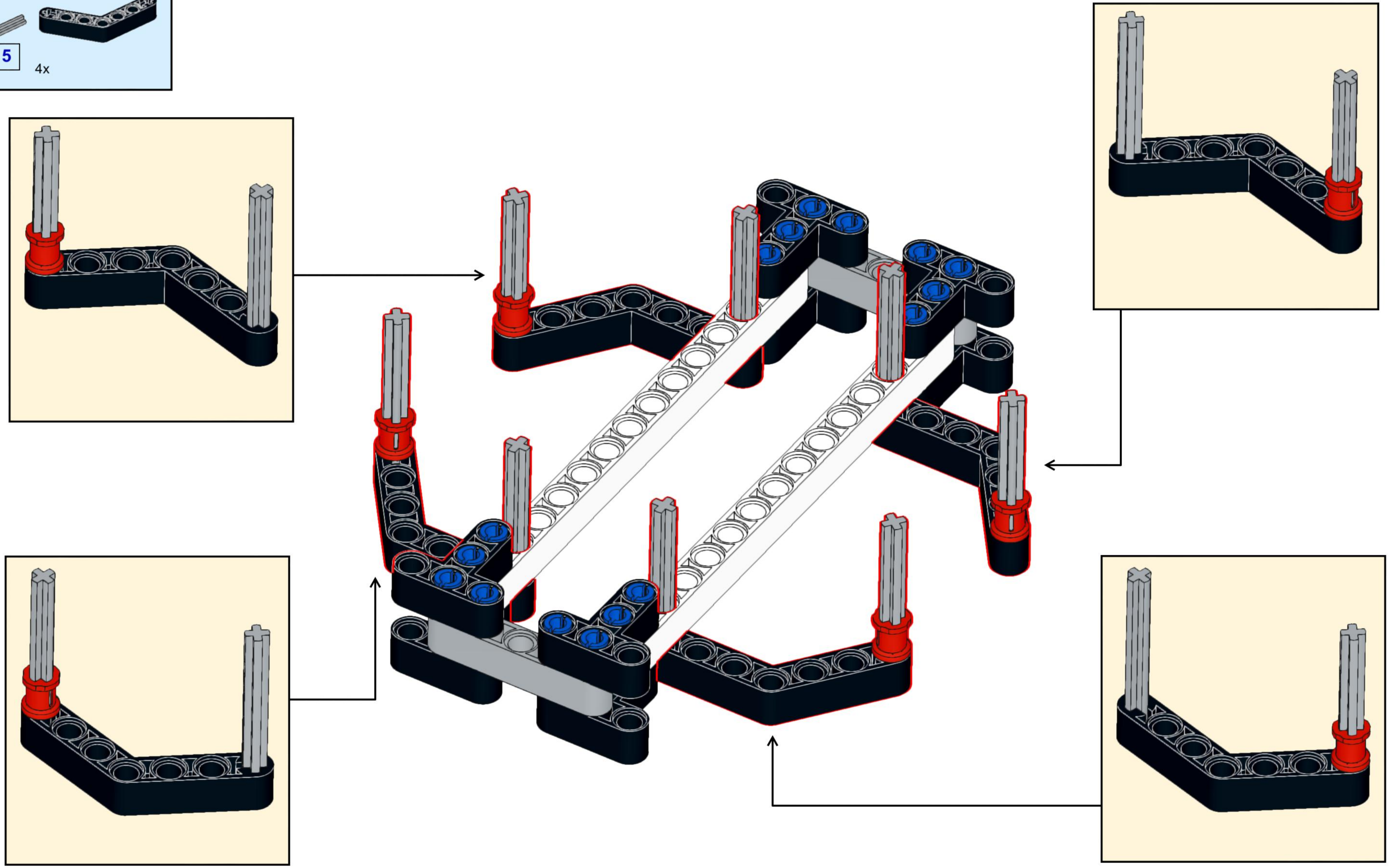

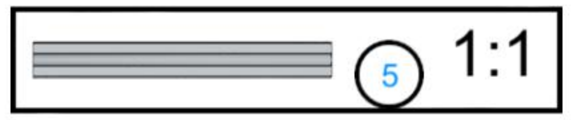

**100**

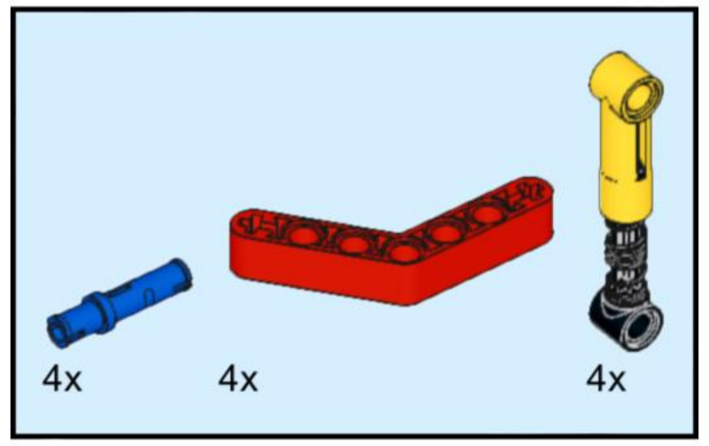


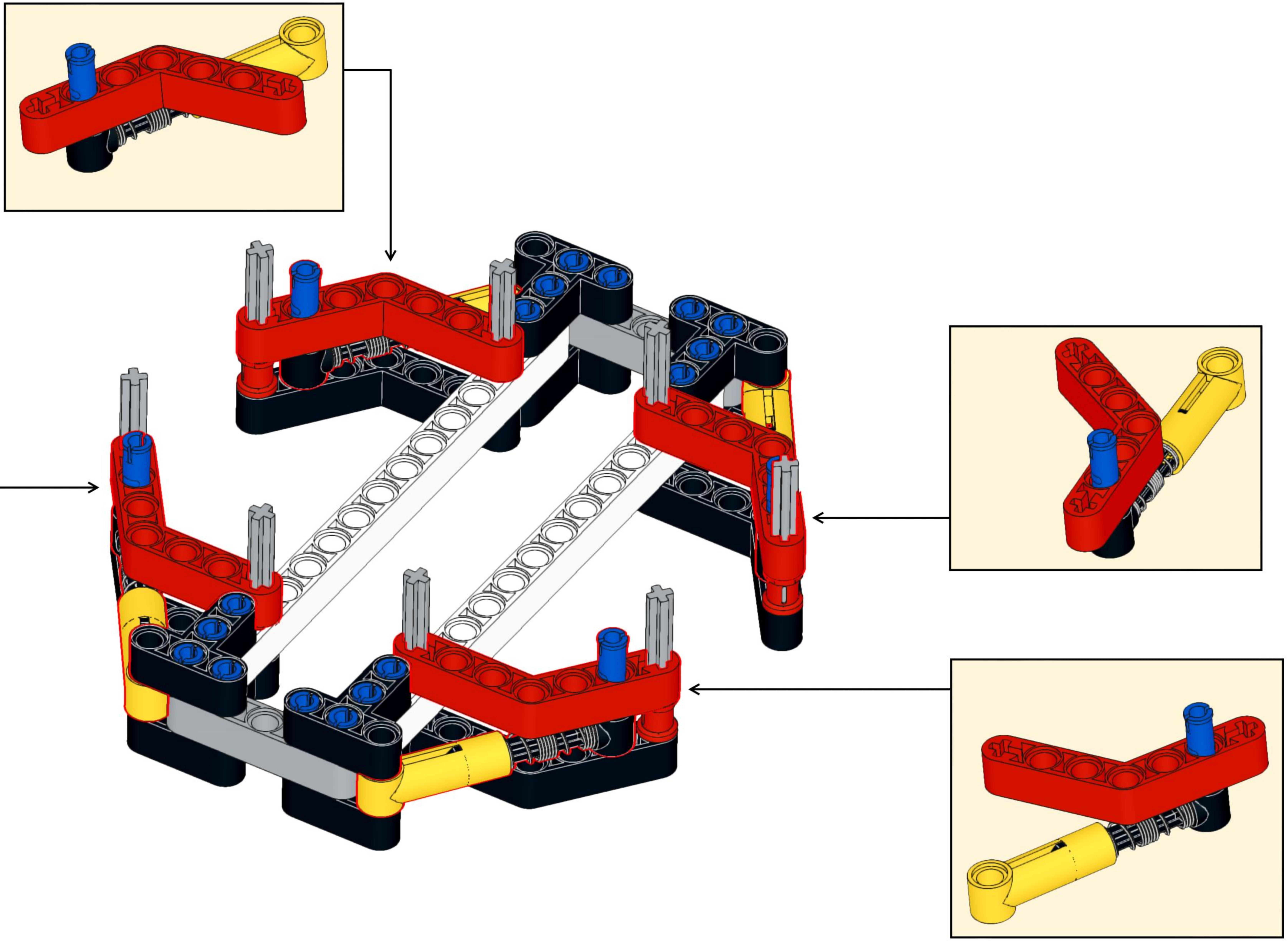

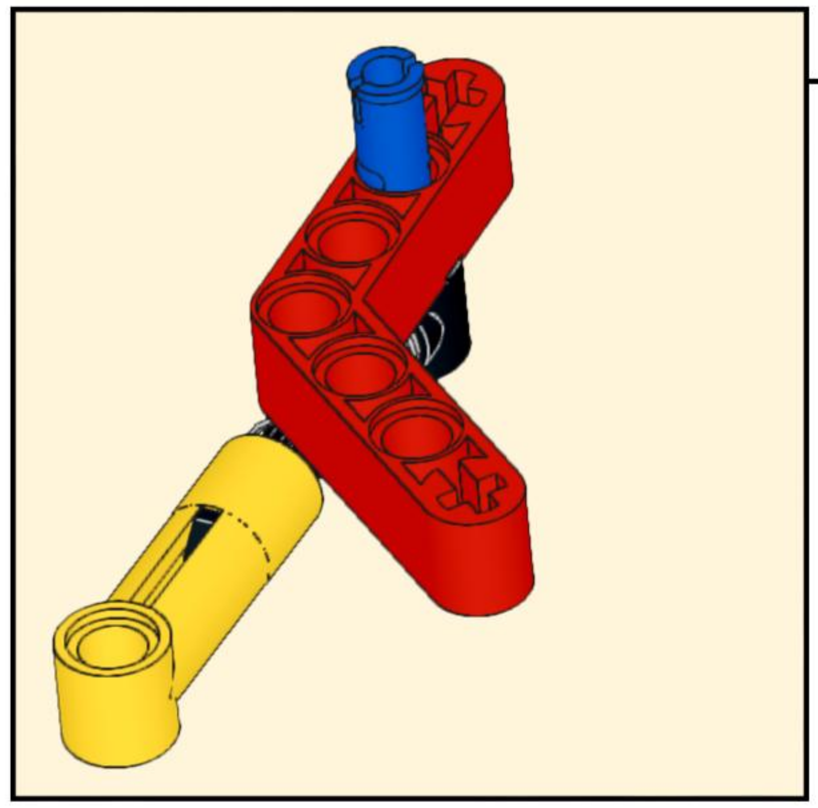

## 101

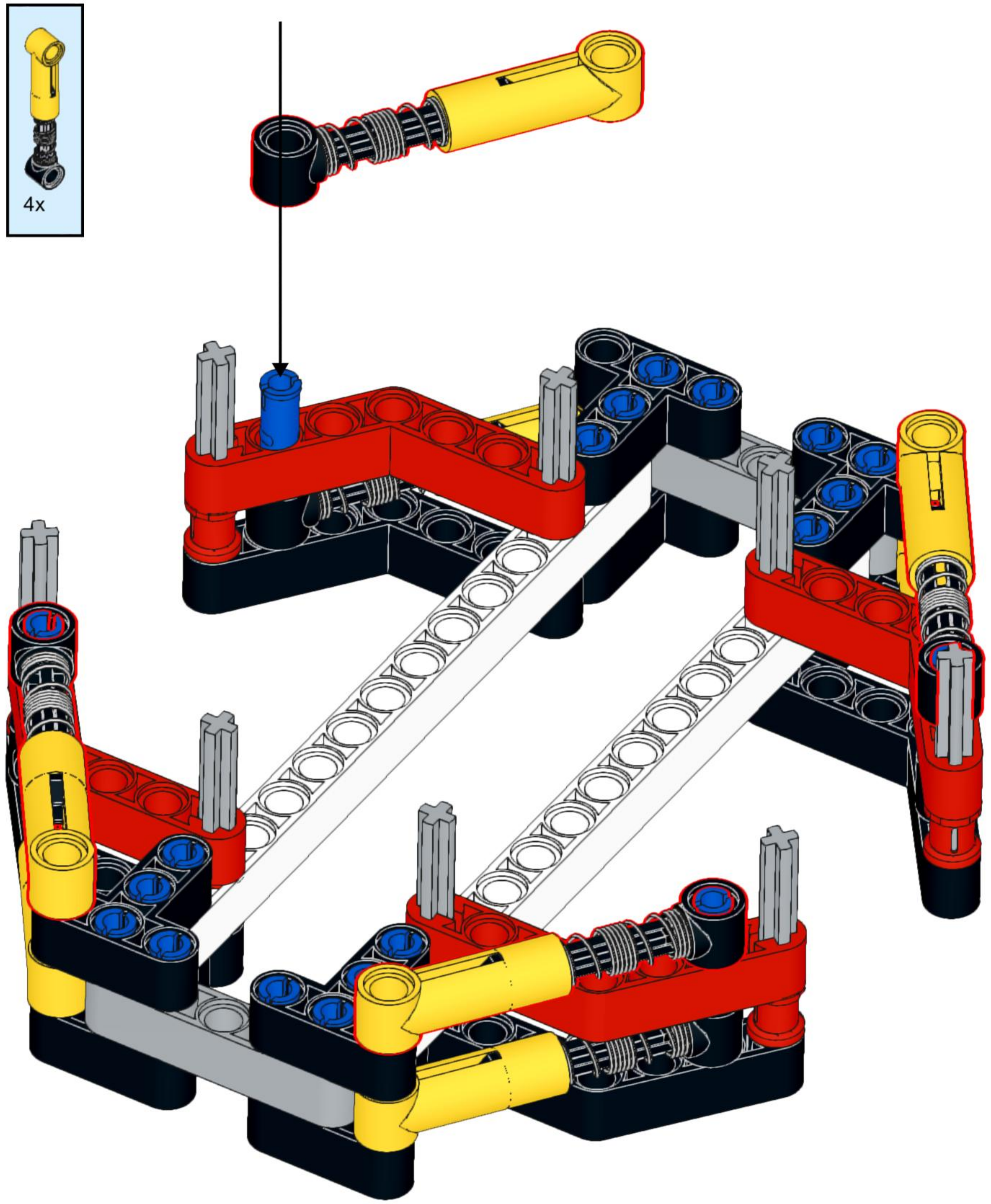


## 102

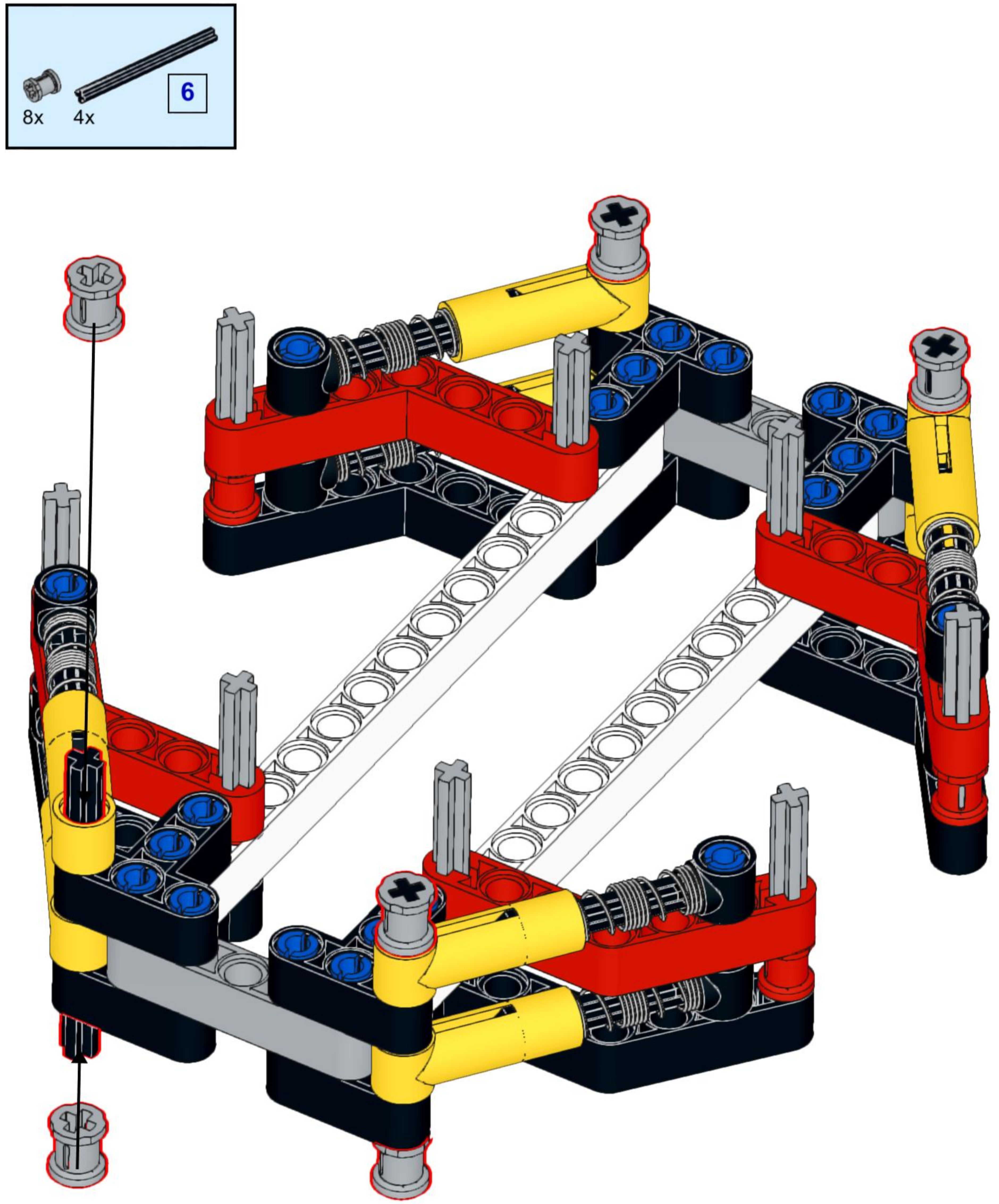


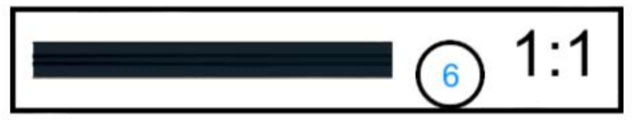

# 103

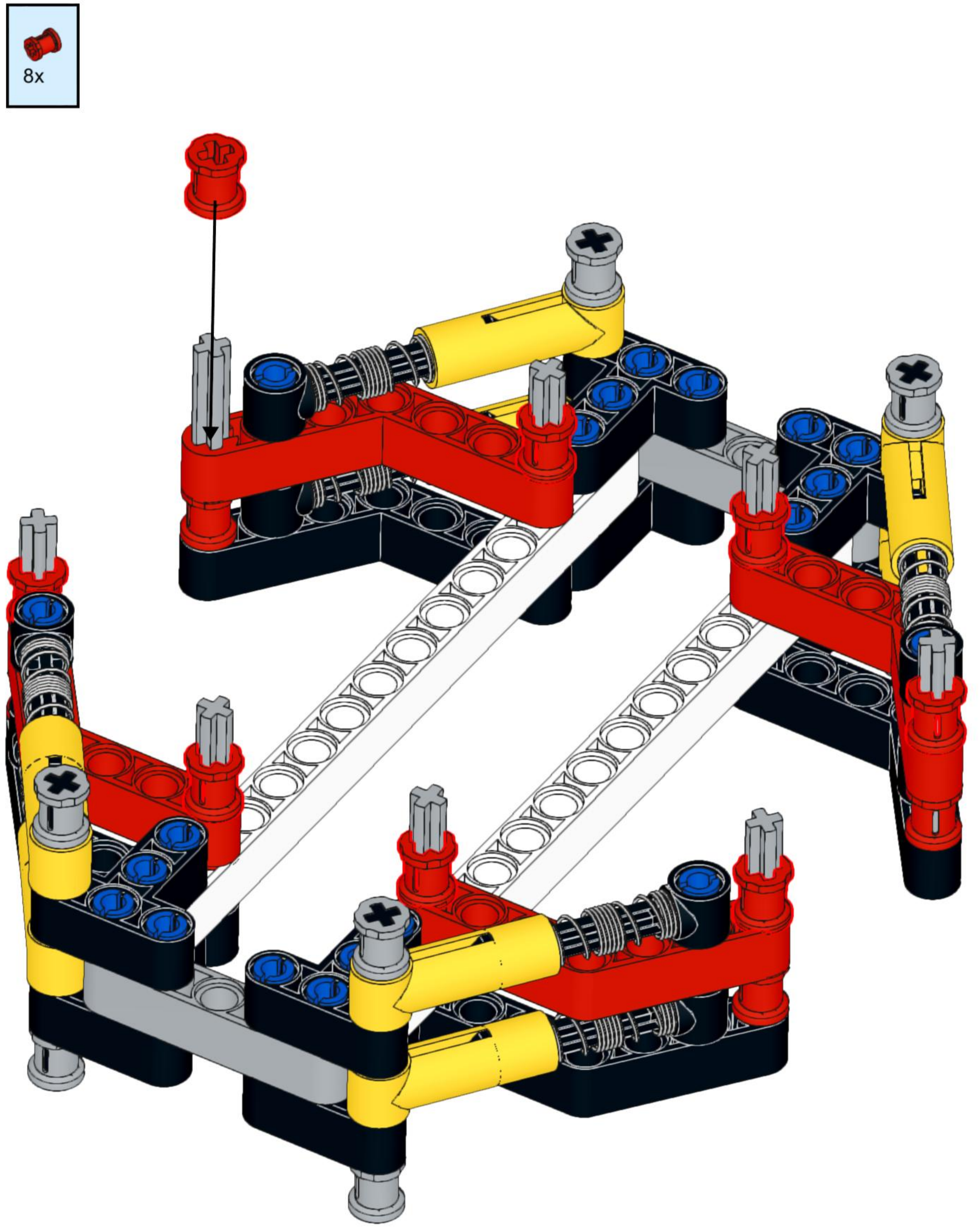


# 104

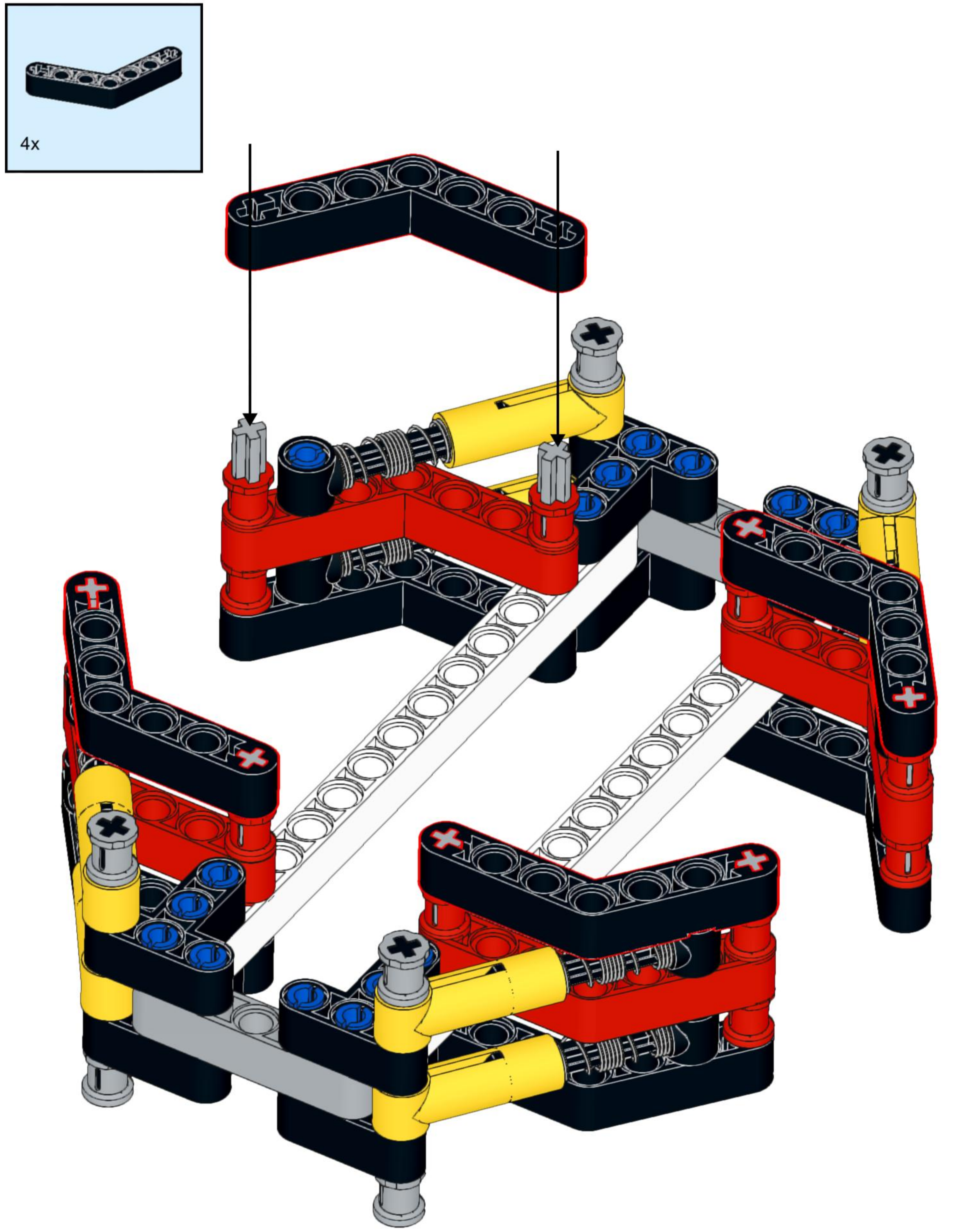

## 105

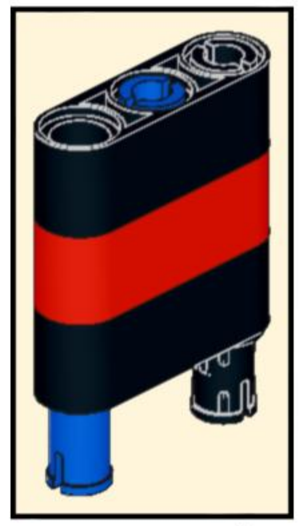

4x

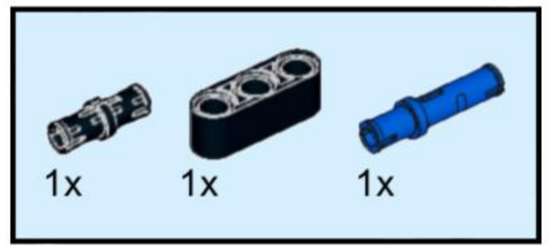


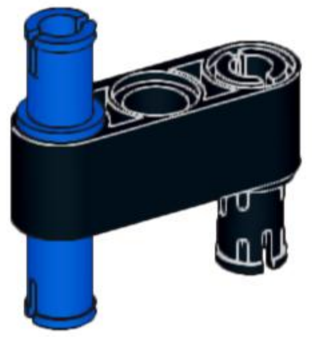

## 106

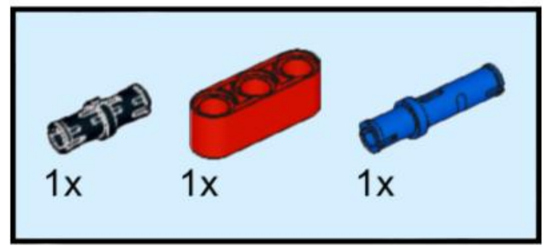


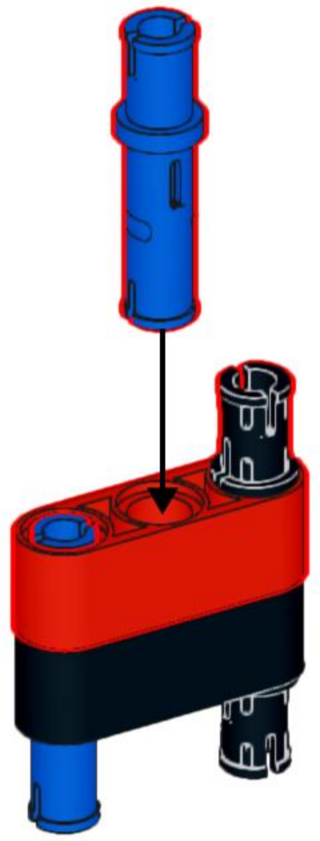

## 107

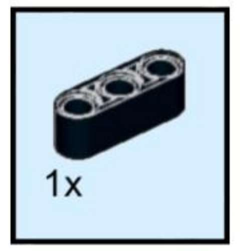


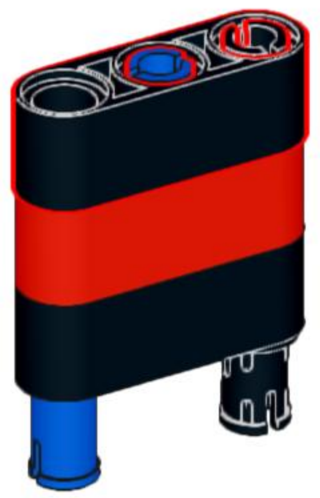

## 108

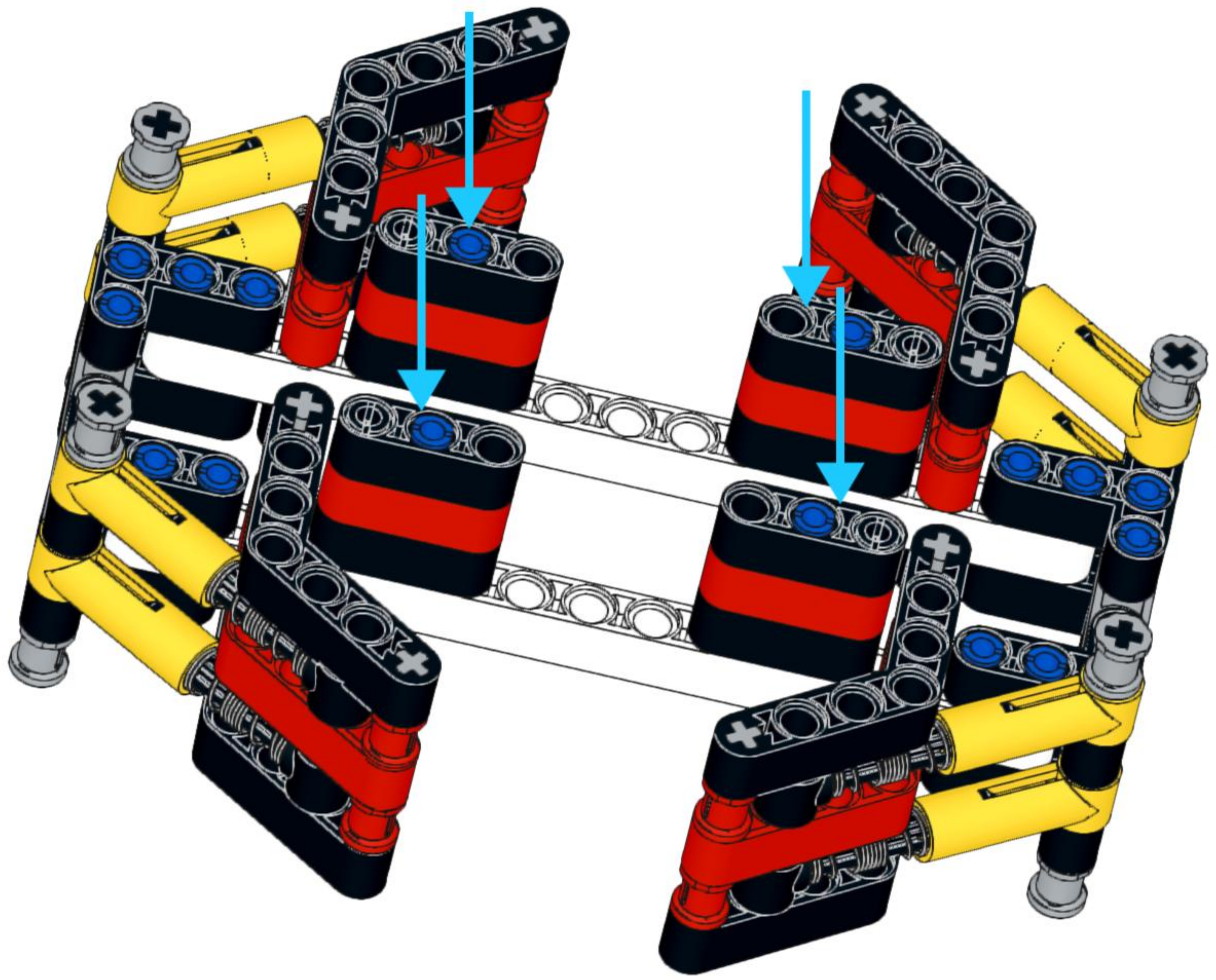

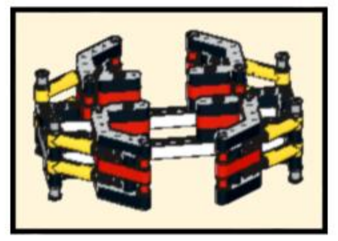

109

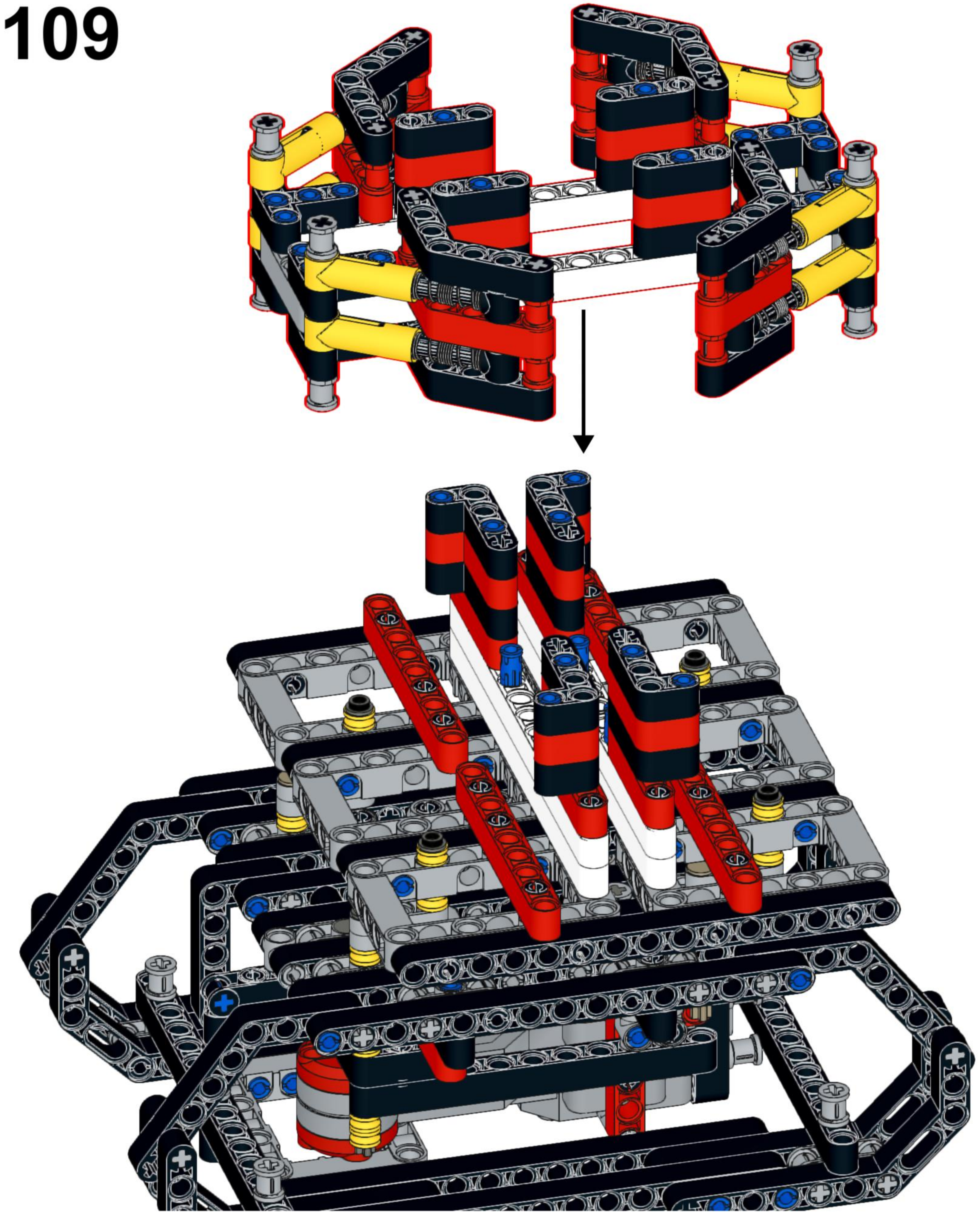

110

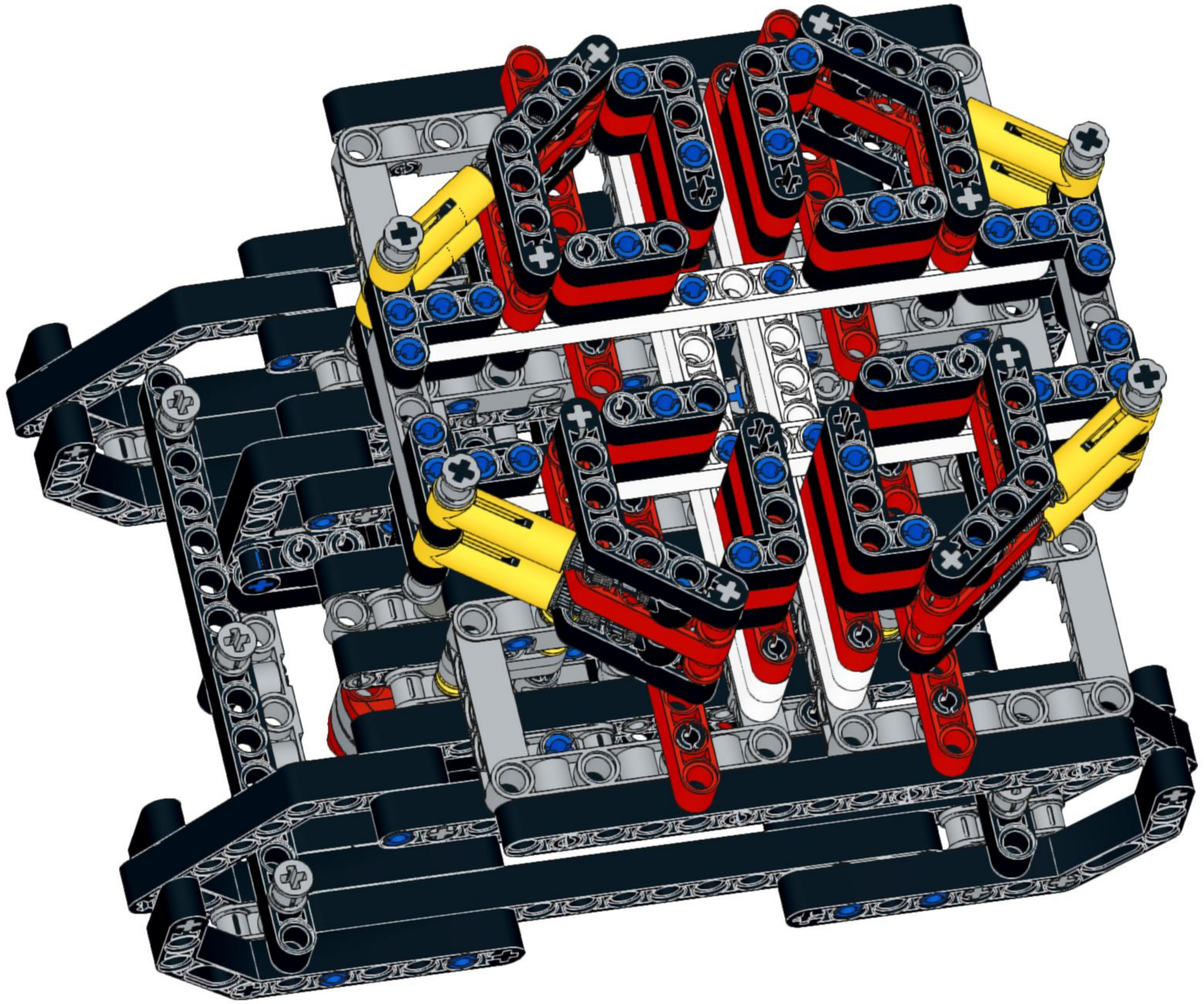

**111**

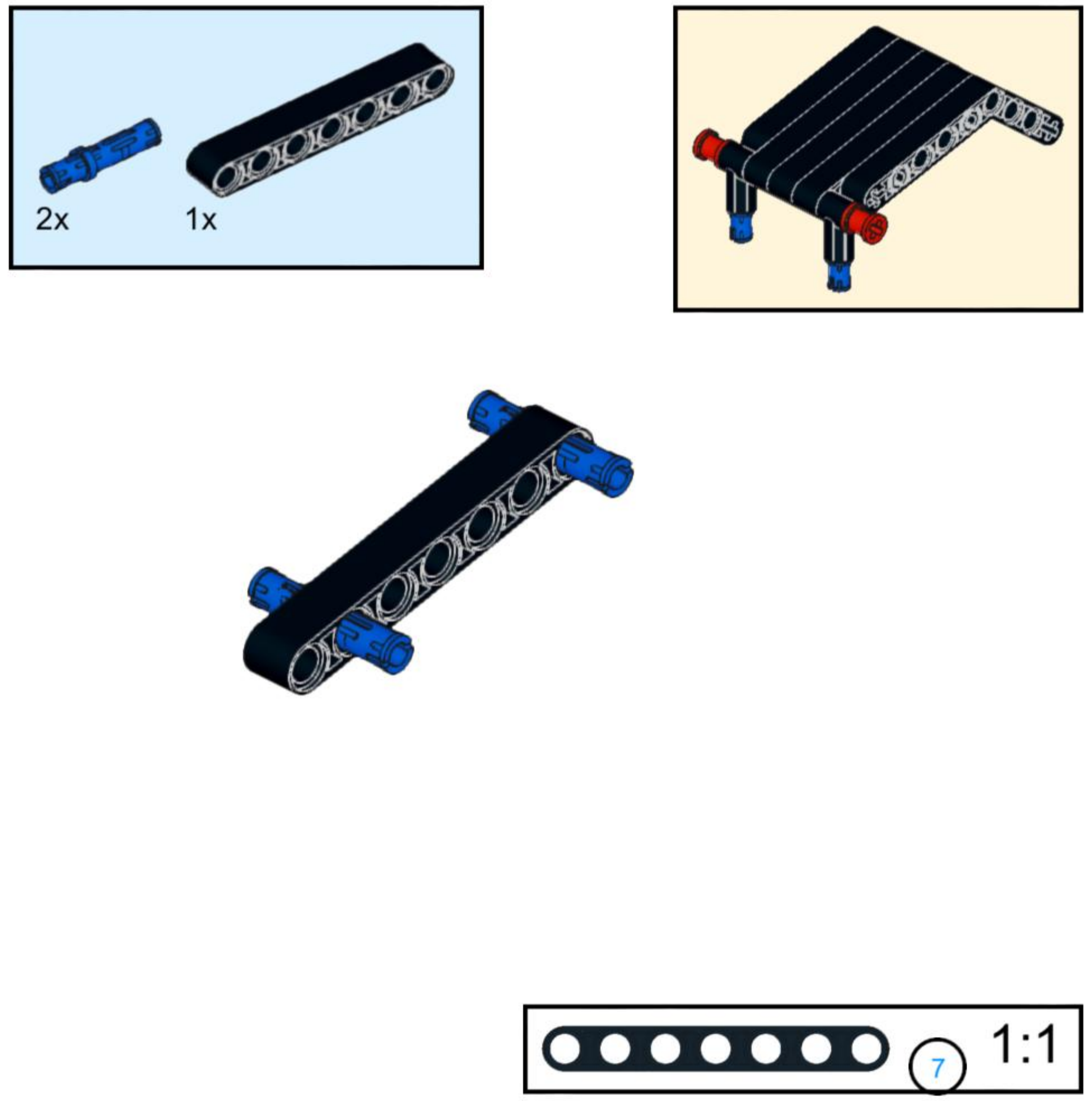


**112**

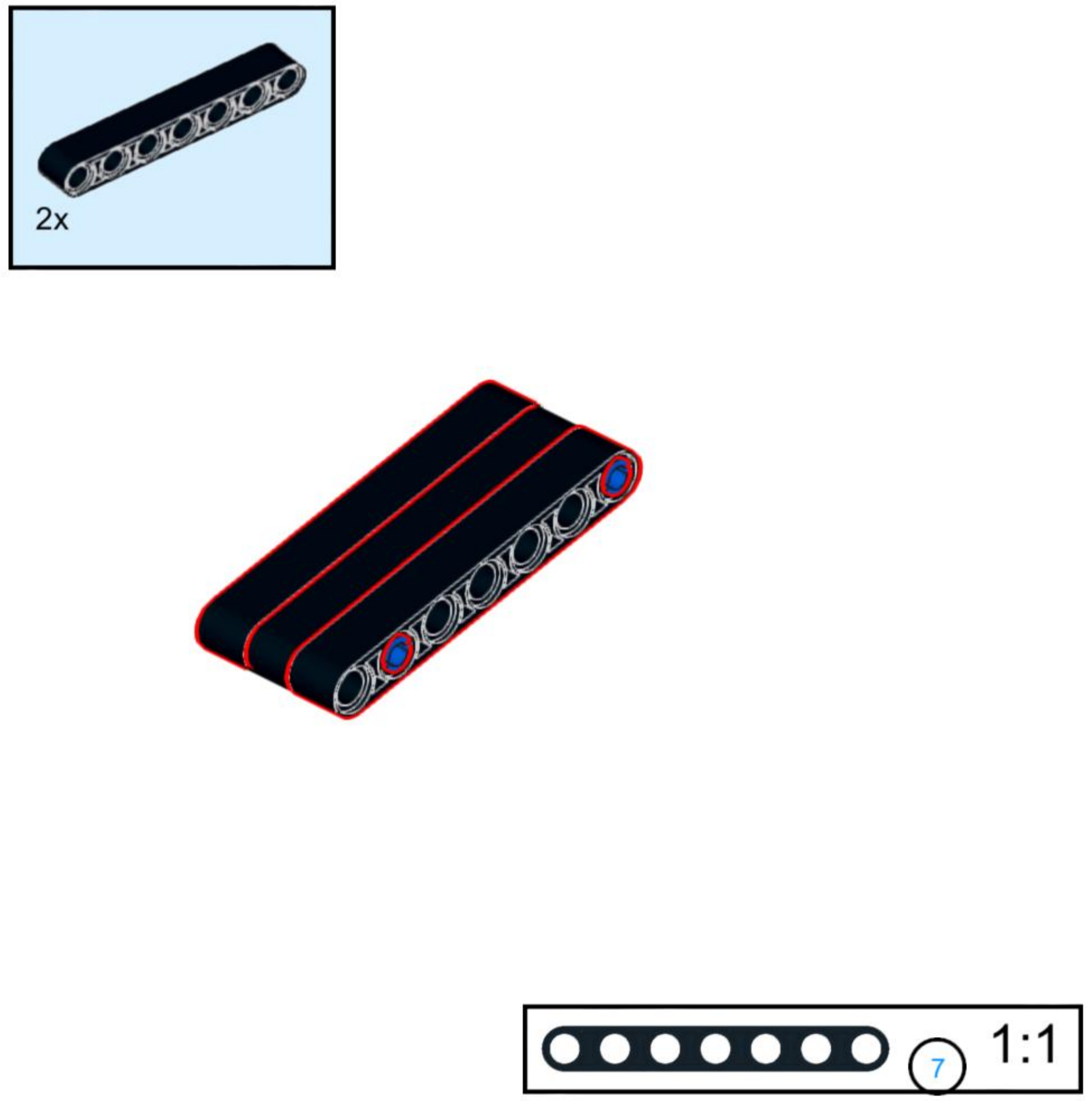


**113**

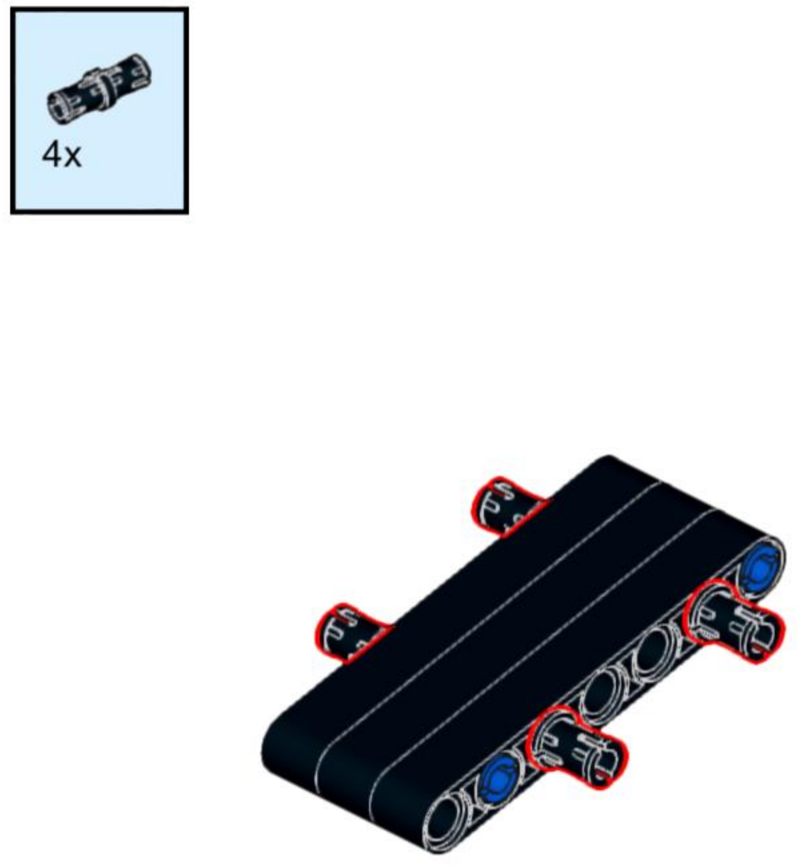


**114**

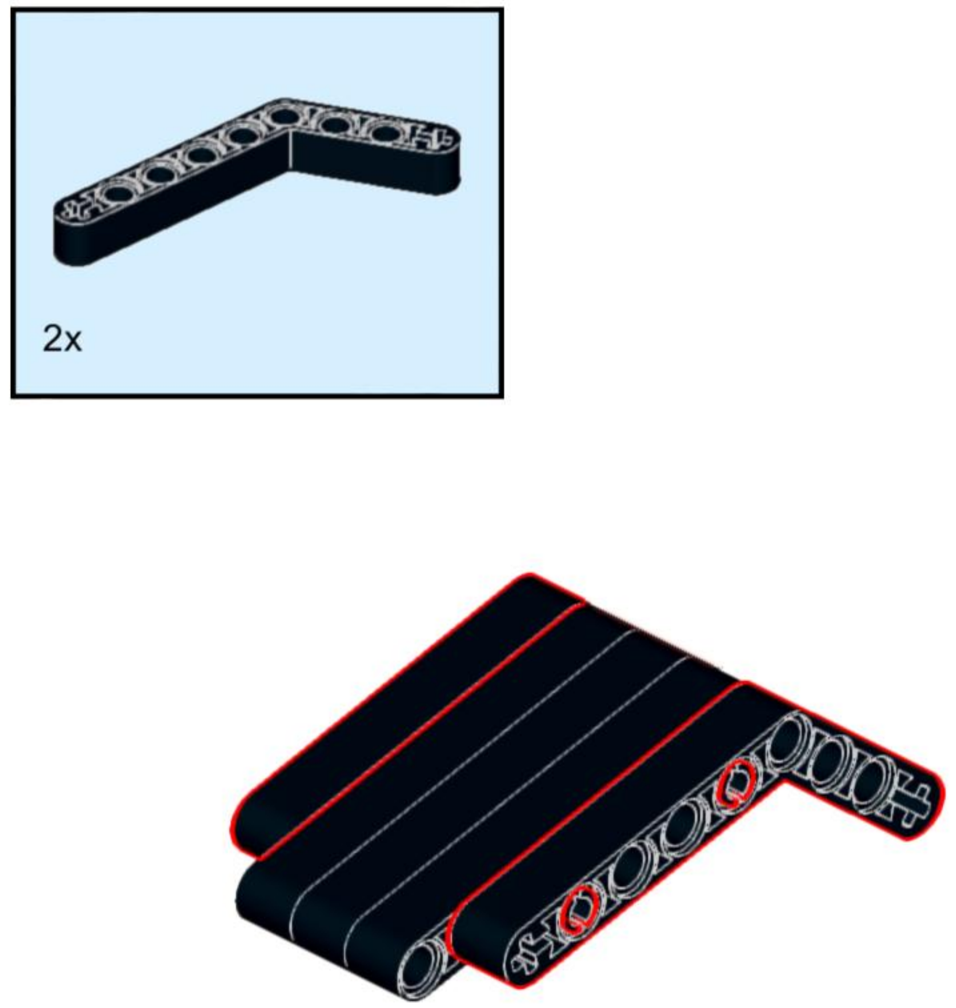


**115**

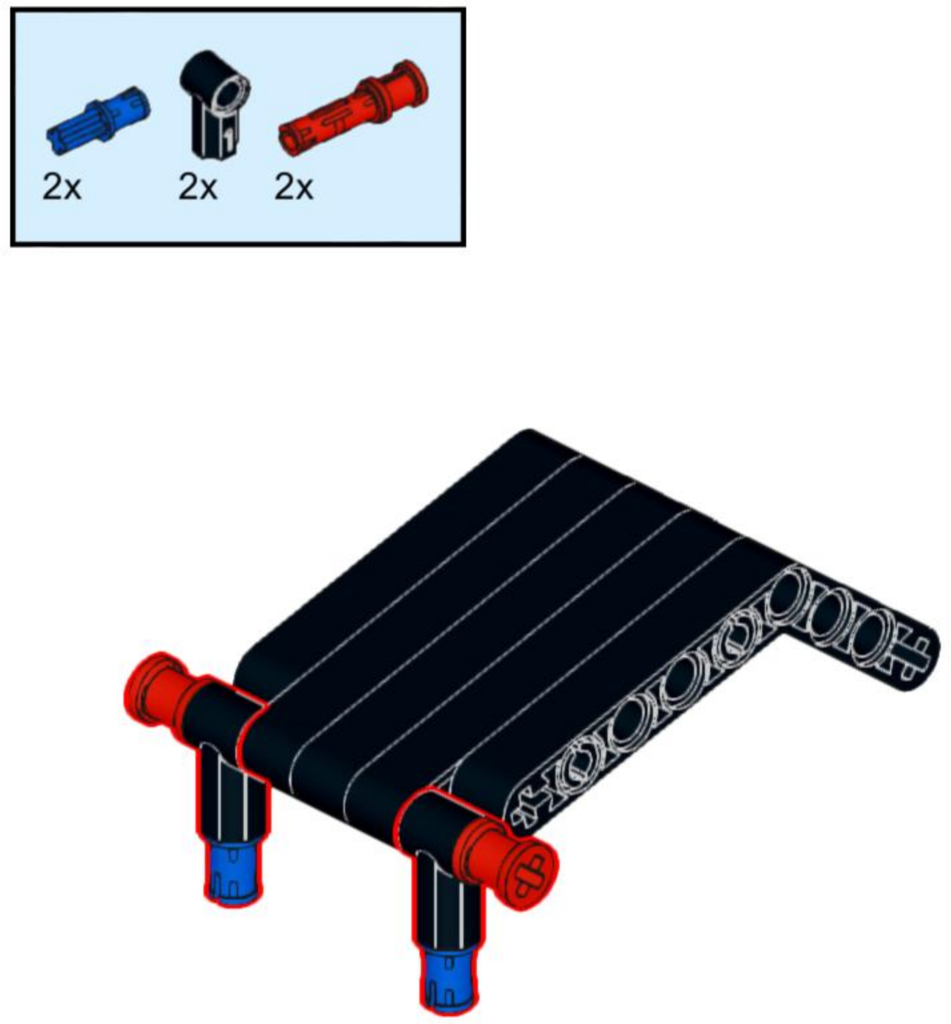


**116**

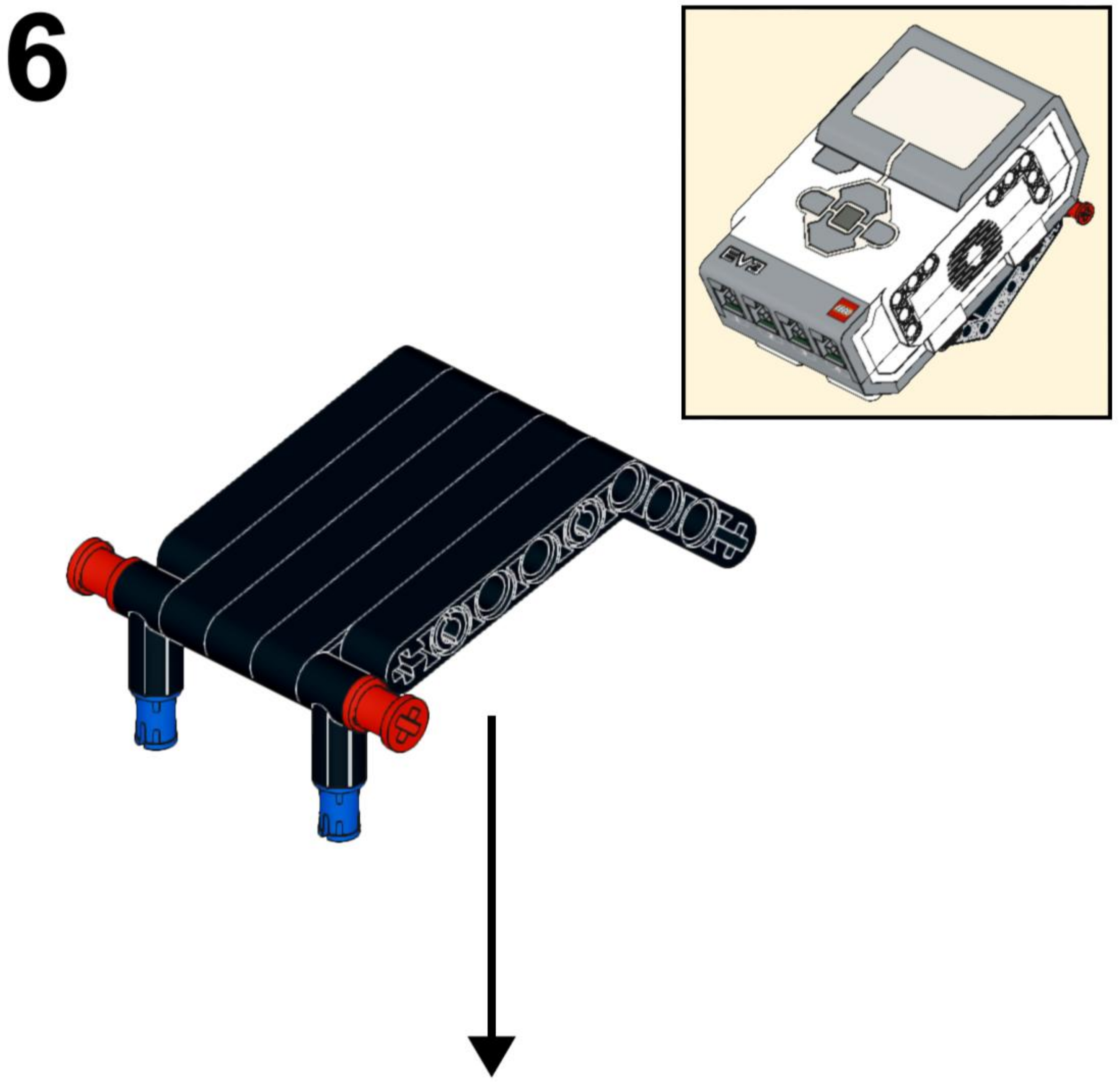

117

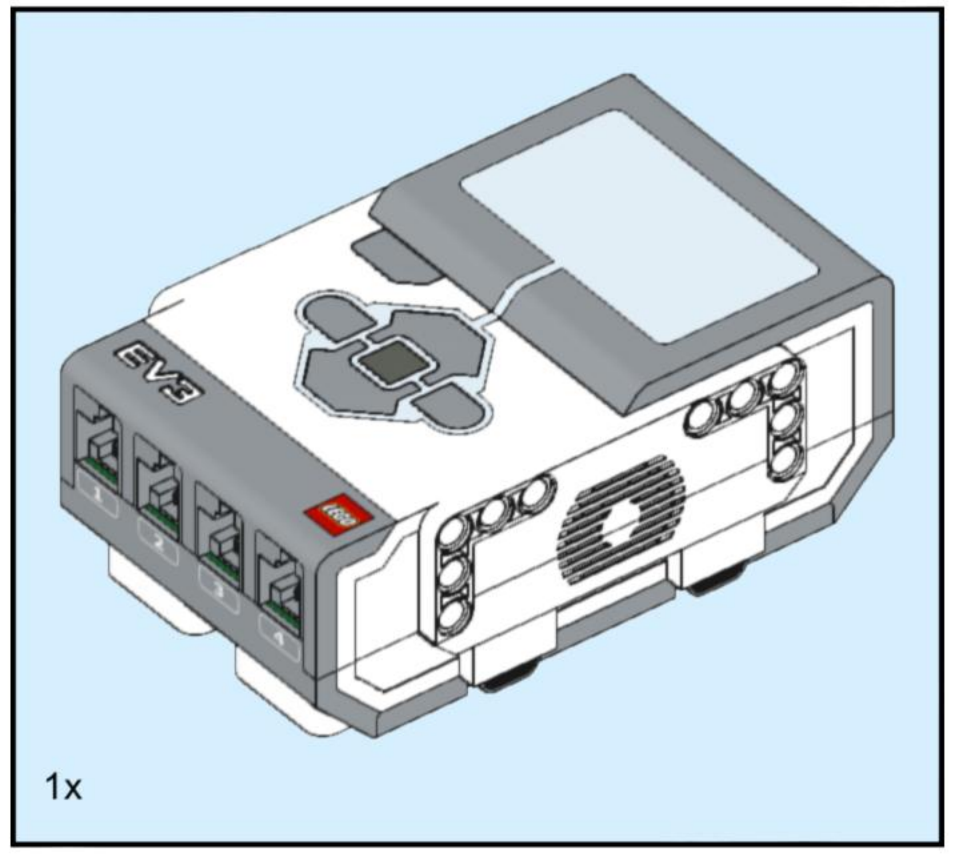


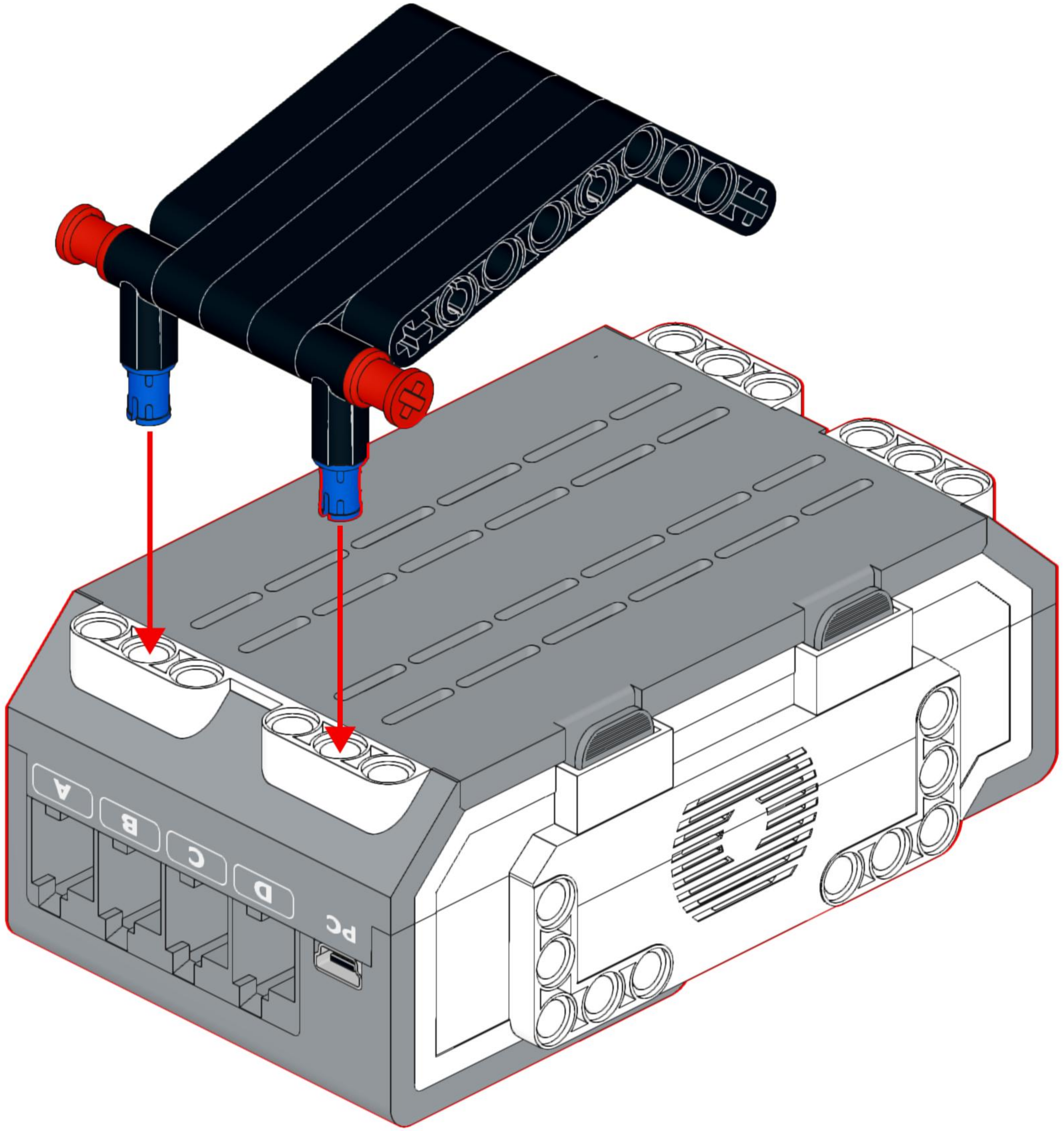

118

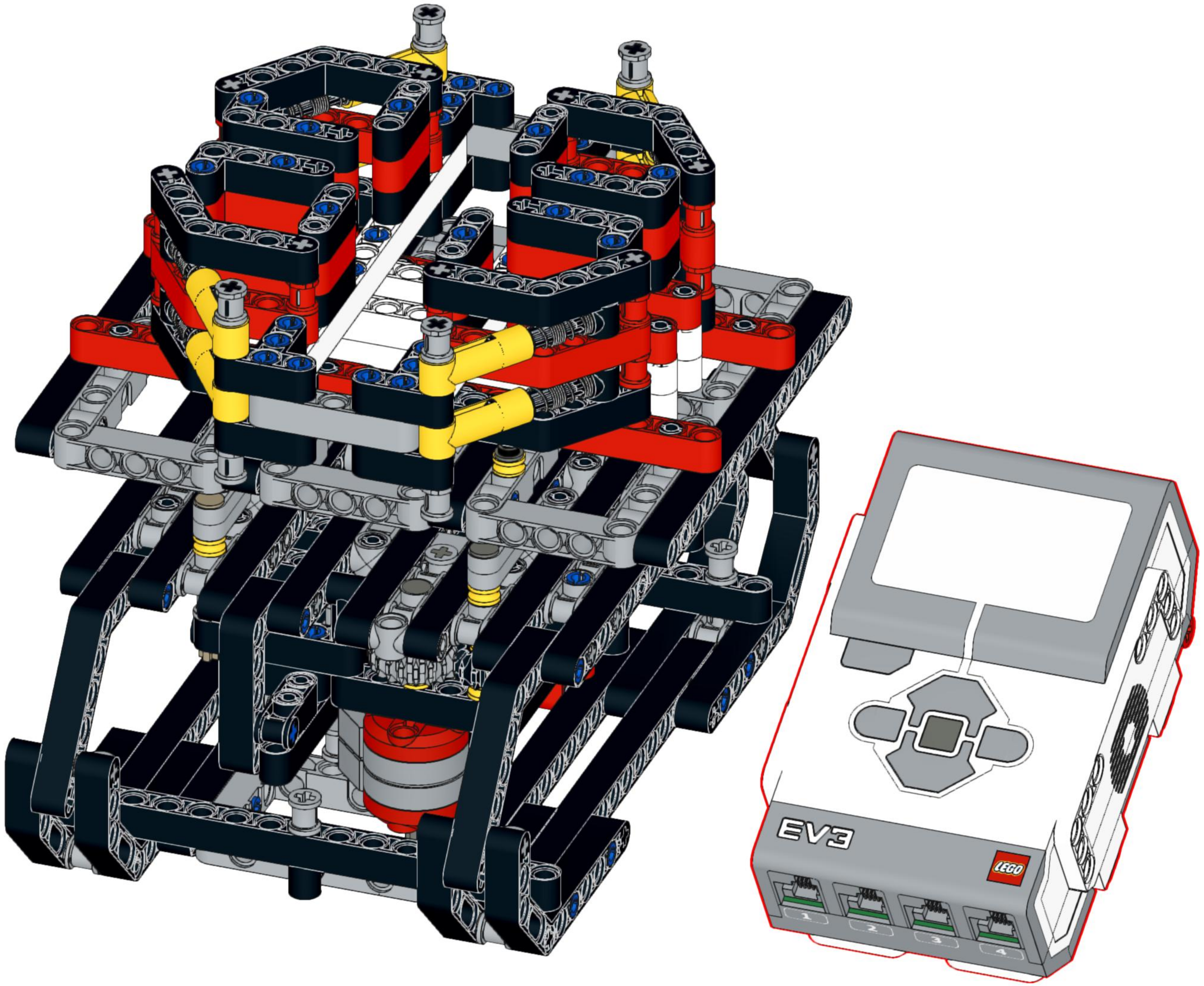

# Assembly Instructions
## Microcentrifuge

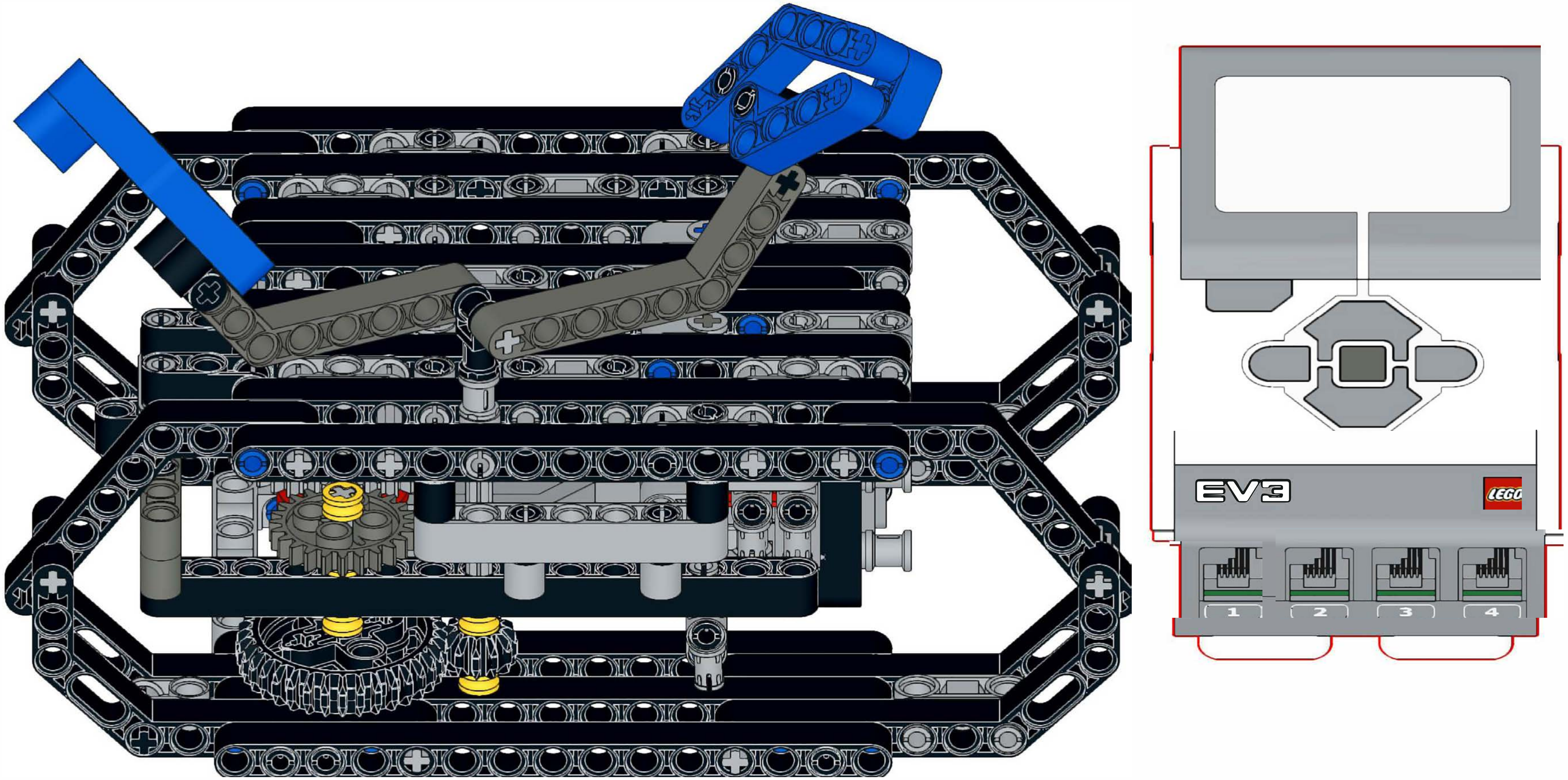

2x
3704
Black

49x
2780
Black

3x
32013
Black

1x
32039
Black

1x
32270
Black

2x
32291
Black

2x
32523
Black

5x
18651
Black

2x
42003
Black

1x
32316
Black

1x
32498
Black

5x
32524
Black

6x
6629
Black

2x
40490
Black

8x
32009
Black

1x
32525
Black

17x
32278
Black

1x
11214
Dark Bluish Gray

1x
3648
Dark Bluish Gray

2x
6629
Dark Bluish Gray

2x
40490
Dark Bluish Gray

1x
3713
Light Bluish Gray

9x
6536
Light Bluish Gray

2x
43857
Light Bluish Gray

2x
22961
Light Bluish Gray

9x
4519
Light Bluish Gray

12x
32054
Light Bluish Gray

11x
32184
Light Bluish Gray

1x
32523
Light Bluish Gray

7x
87082
Light Bluish Gray

9x
48989
Light Bluish Gray

2x
32140
Light Bluish Gray

13x
32073
Light Bluish Gray

1x
32526
Light Bluish Gray

3x
44294
Light Bluish Gray

1x
32524
Light Bluish Gray

1x
3649
Light Bluish Gray

1x
64179
Light Bluish Gray

1x
60485
Light Bluish Gray

1x
95658
White

1x
95646c01
White

1x
32526
Red

1x
15462
Medium Brown

8x
4265c
Yellow

2x
43857
Blue

8x
43093
Blue

26x
32556
Blue

2x
32140
Blue

2x
32348
Blue

1

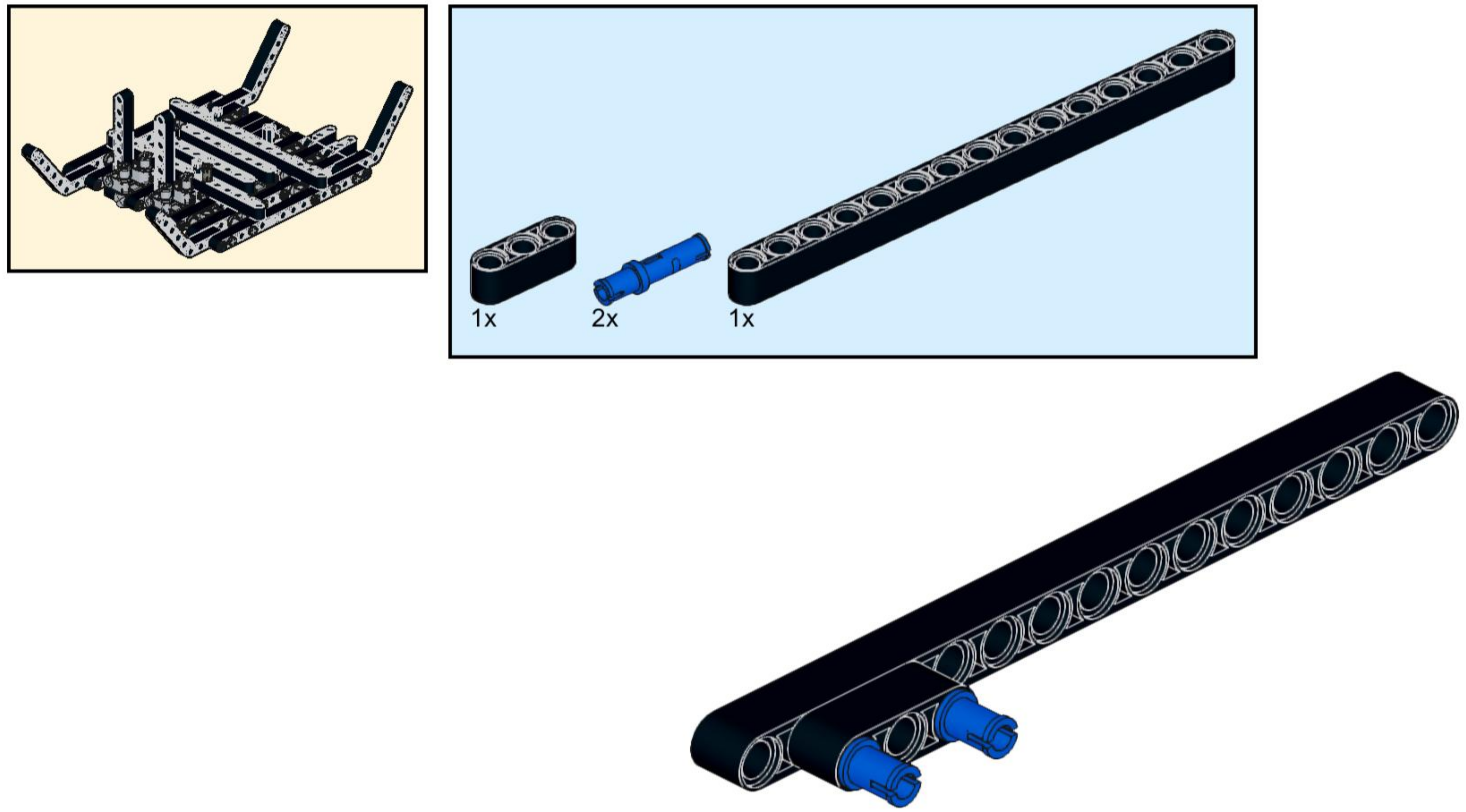


2

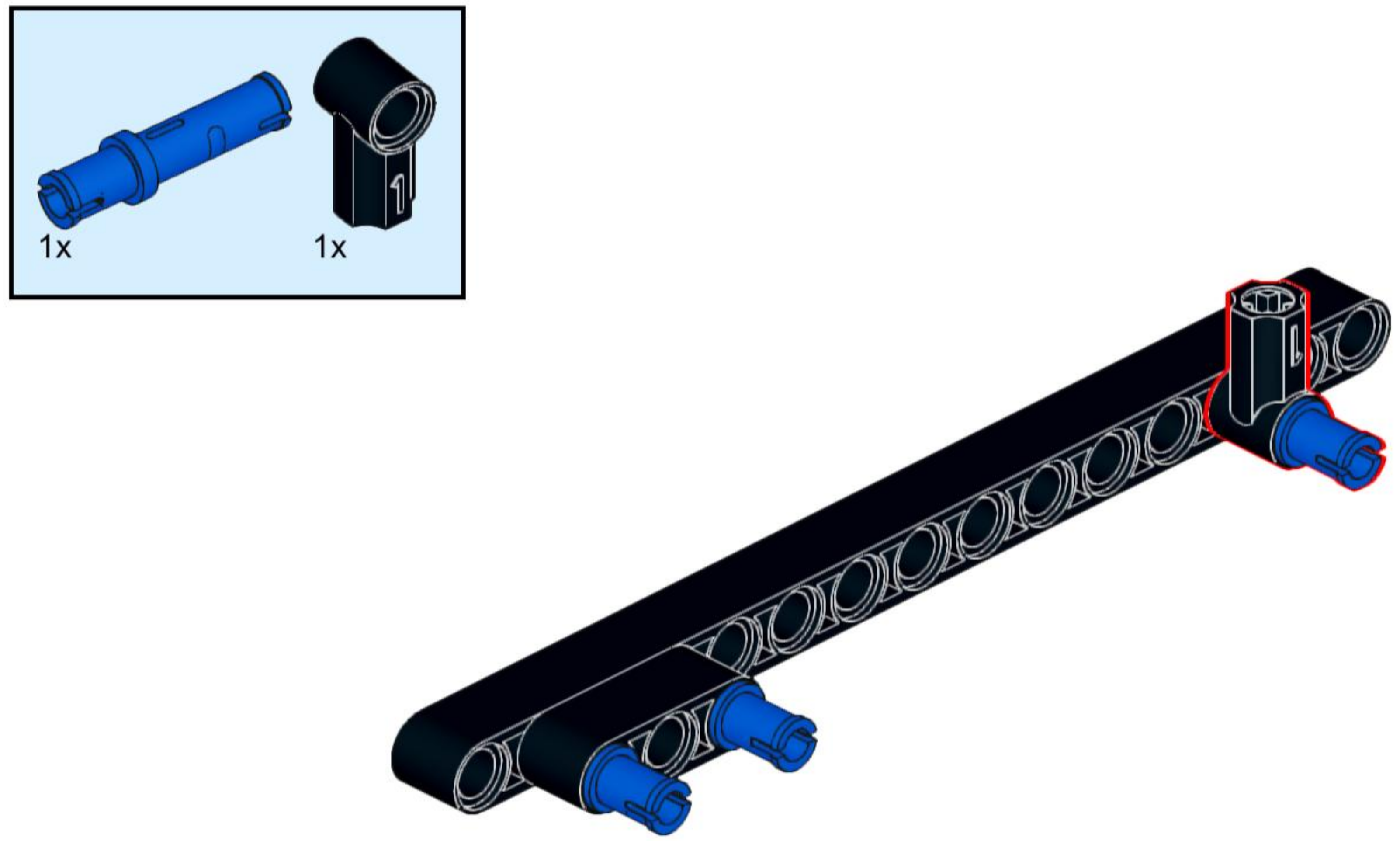


3

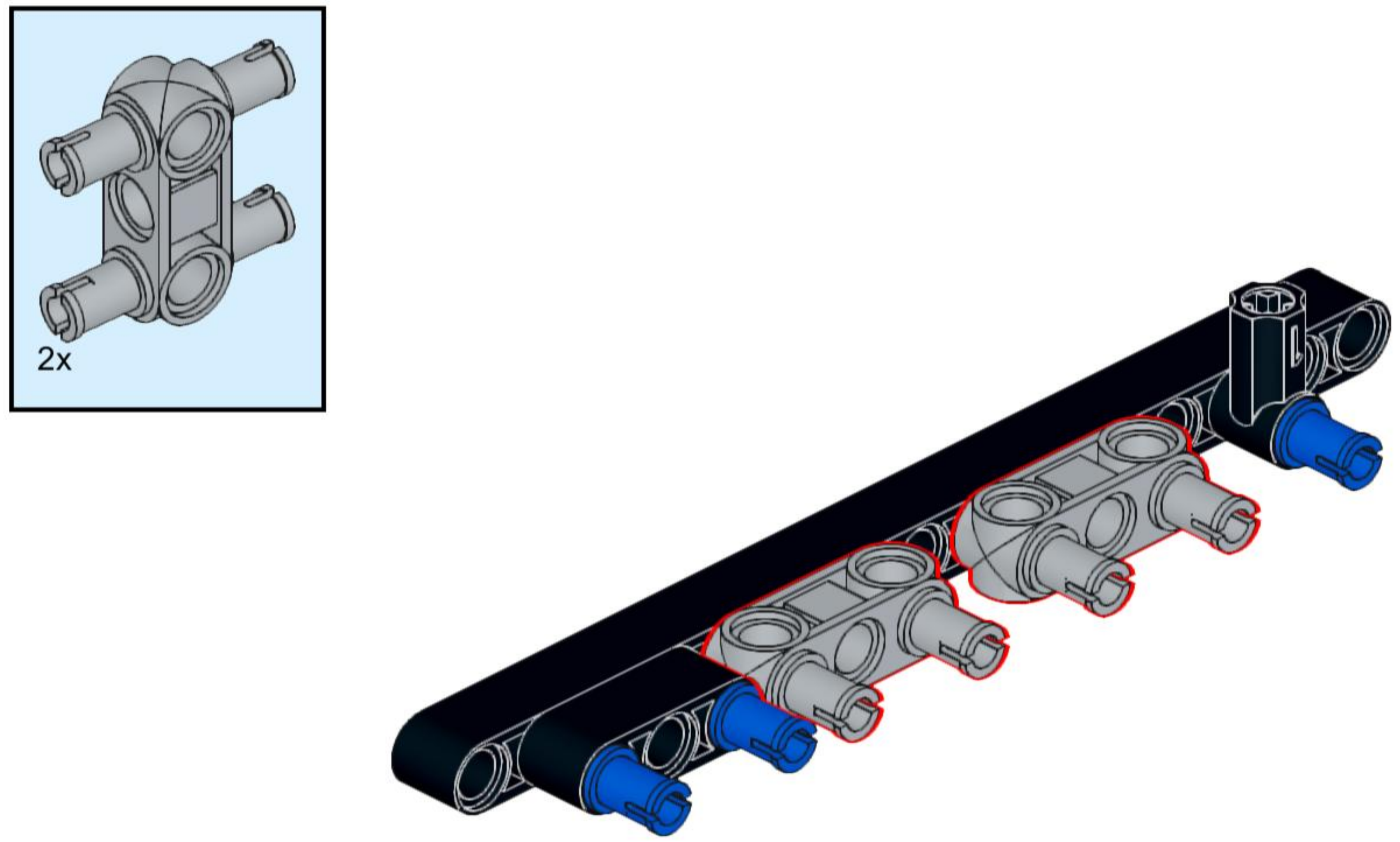


4

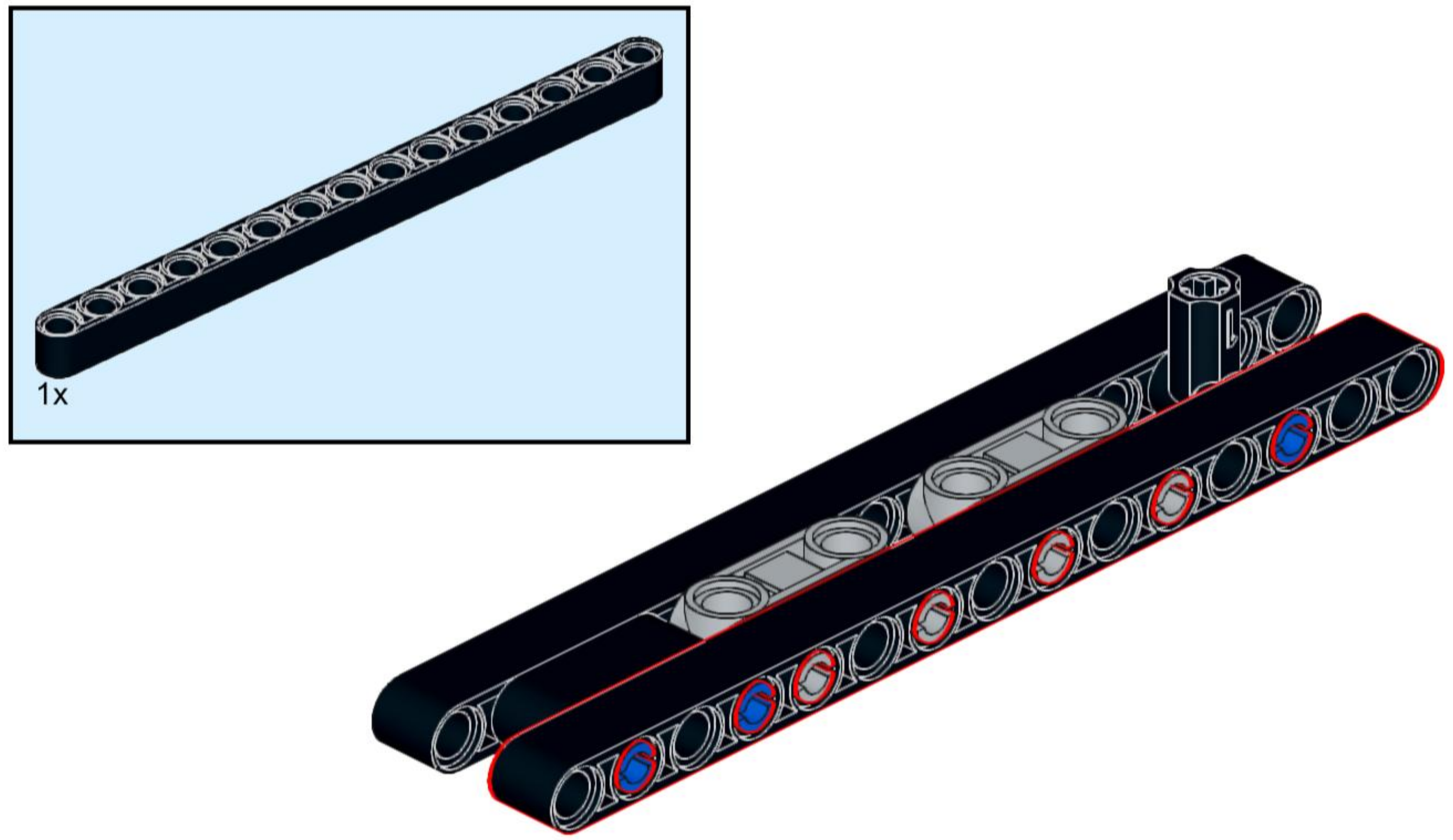


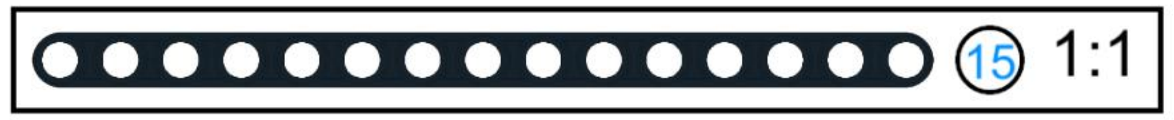

5

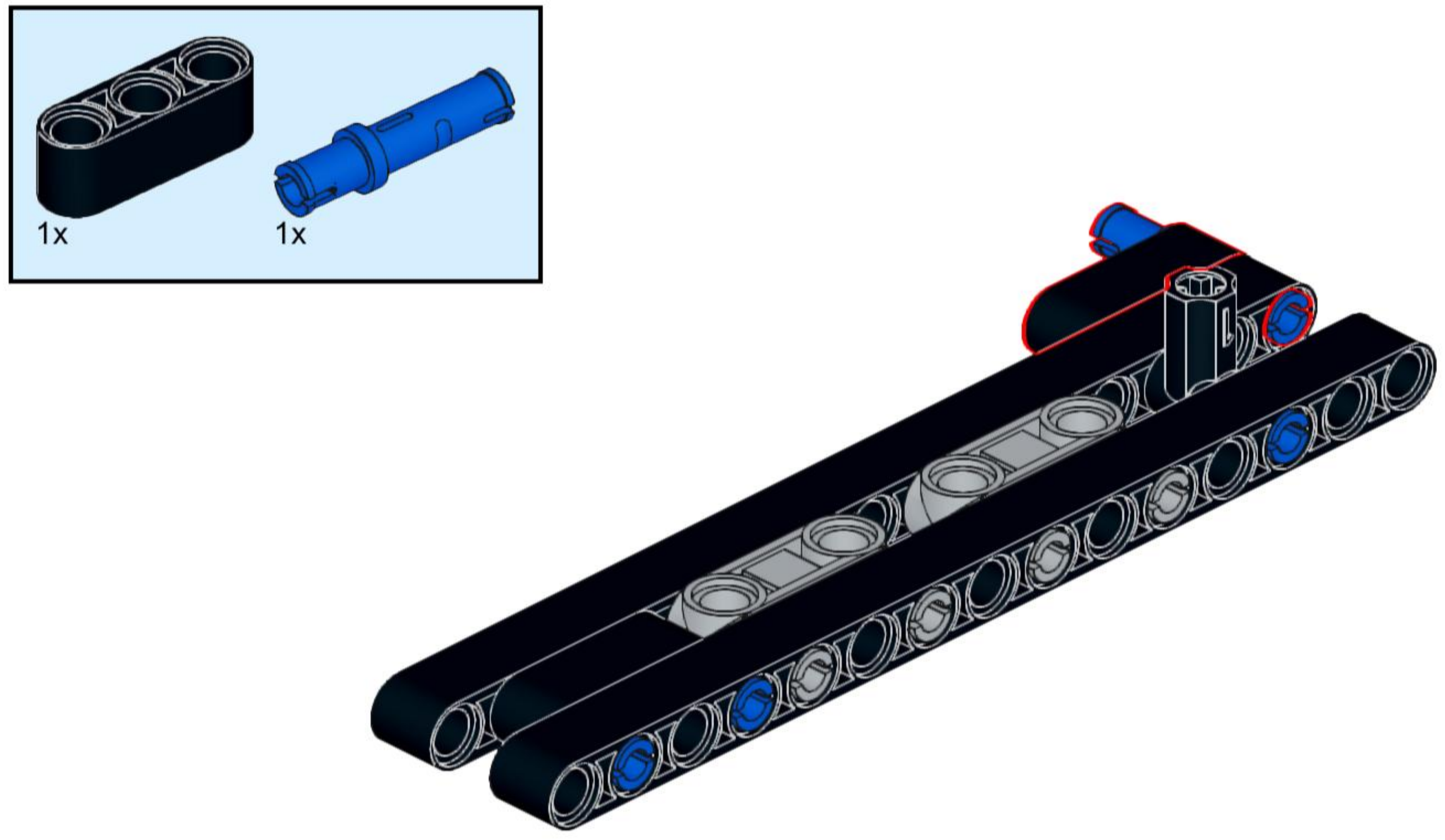


6

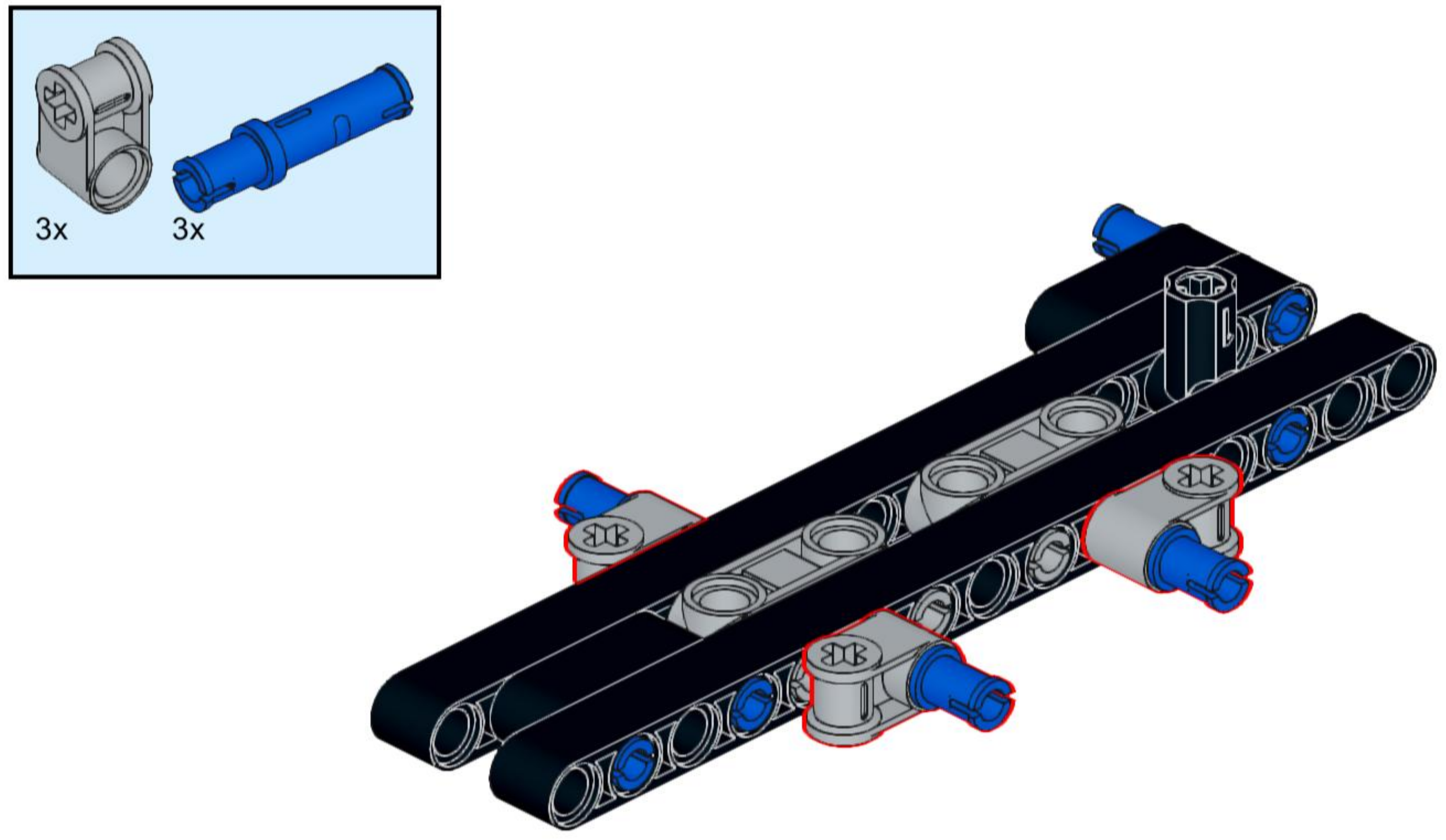


7

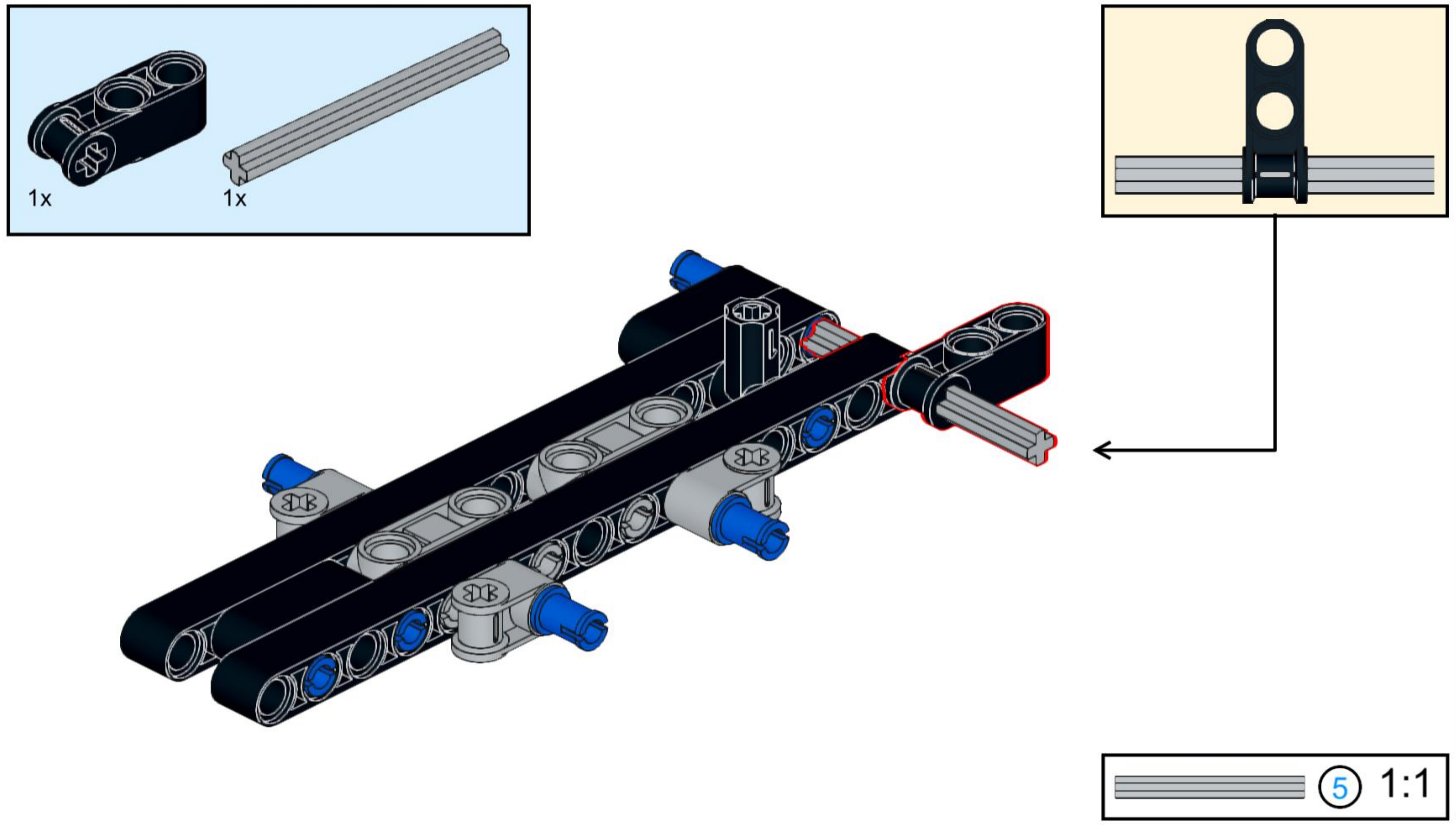


8

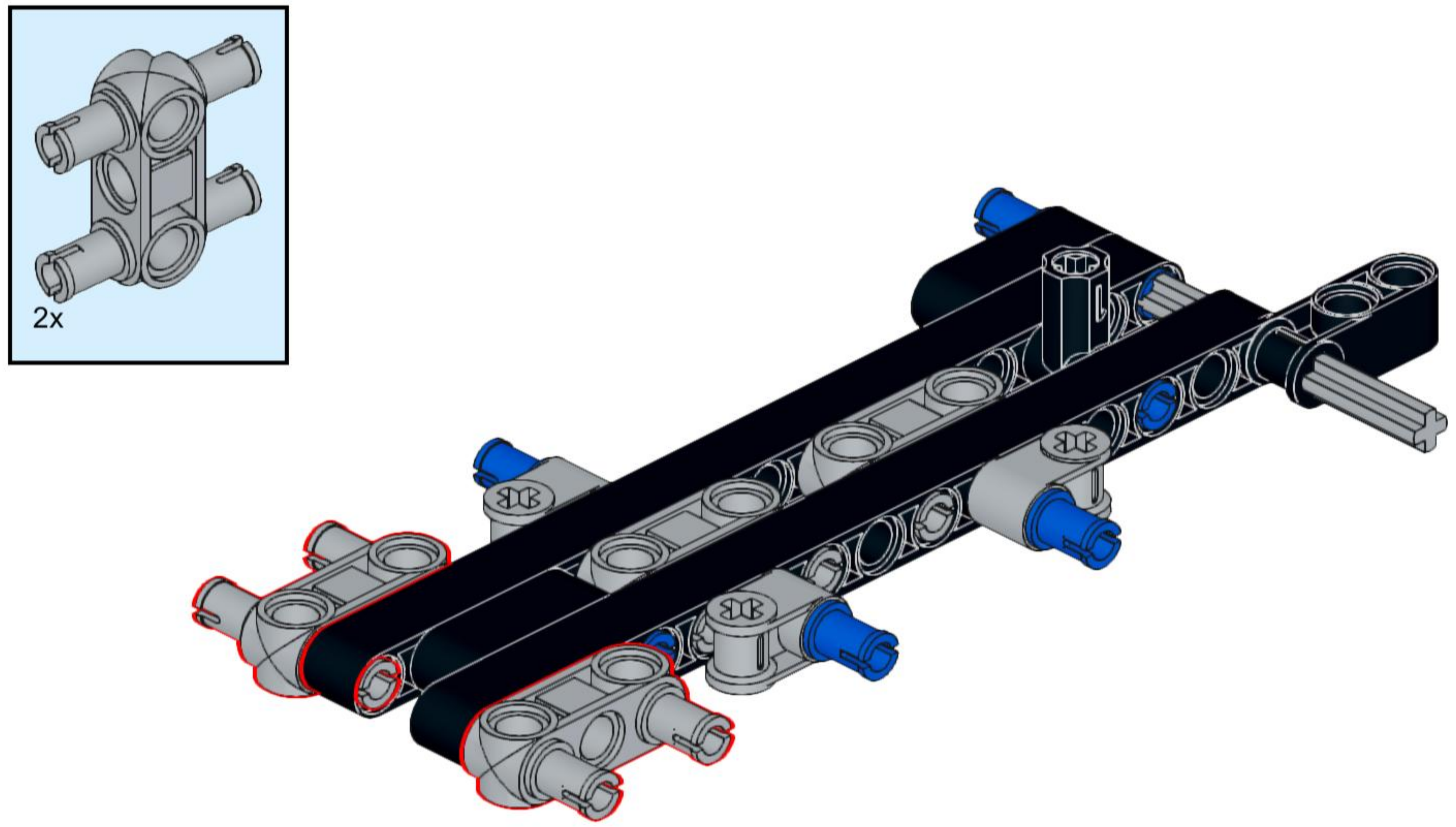

9

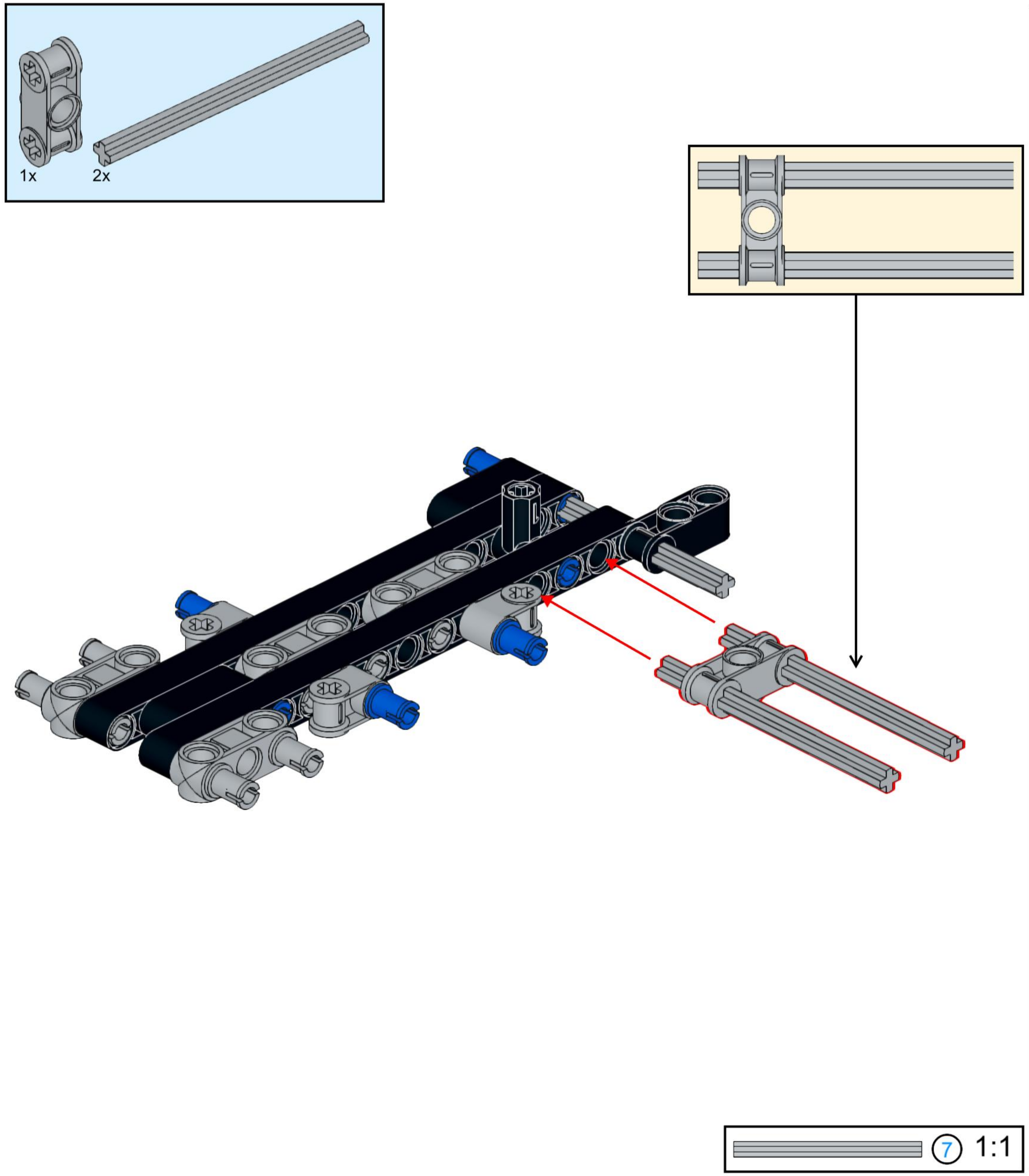


10

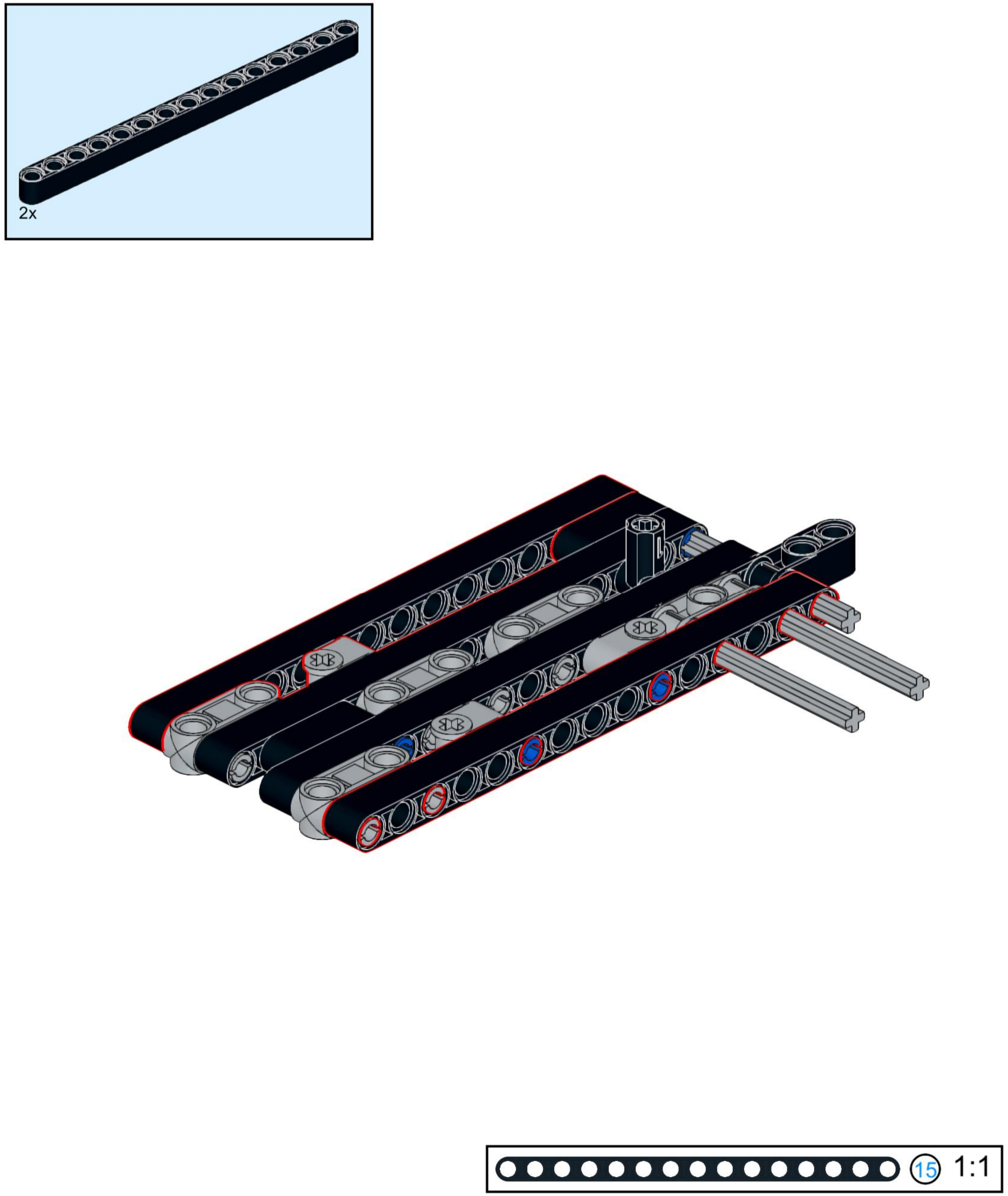

## 11

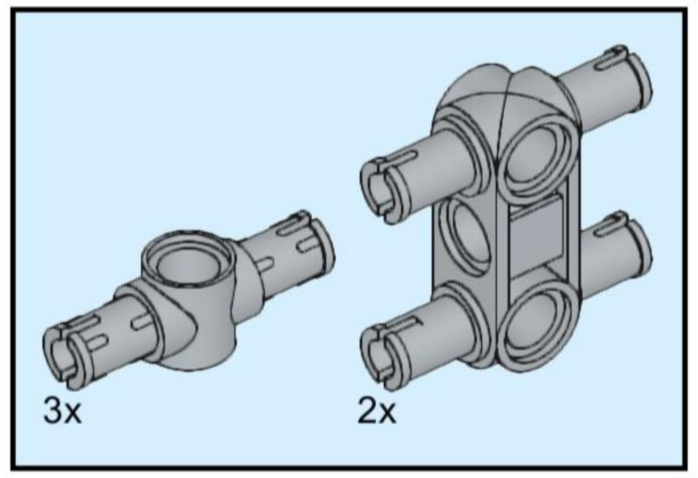


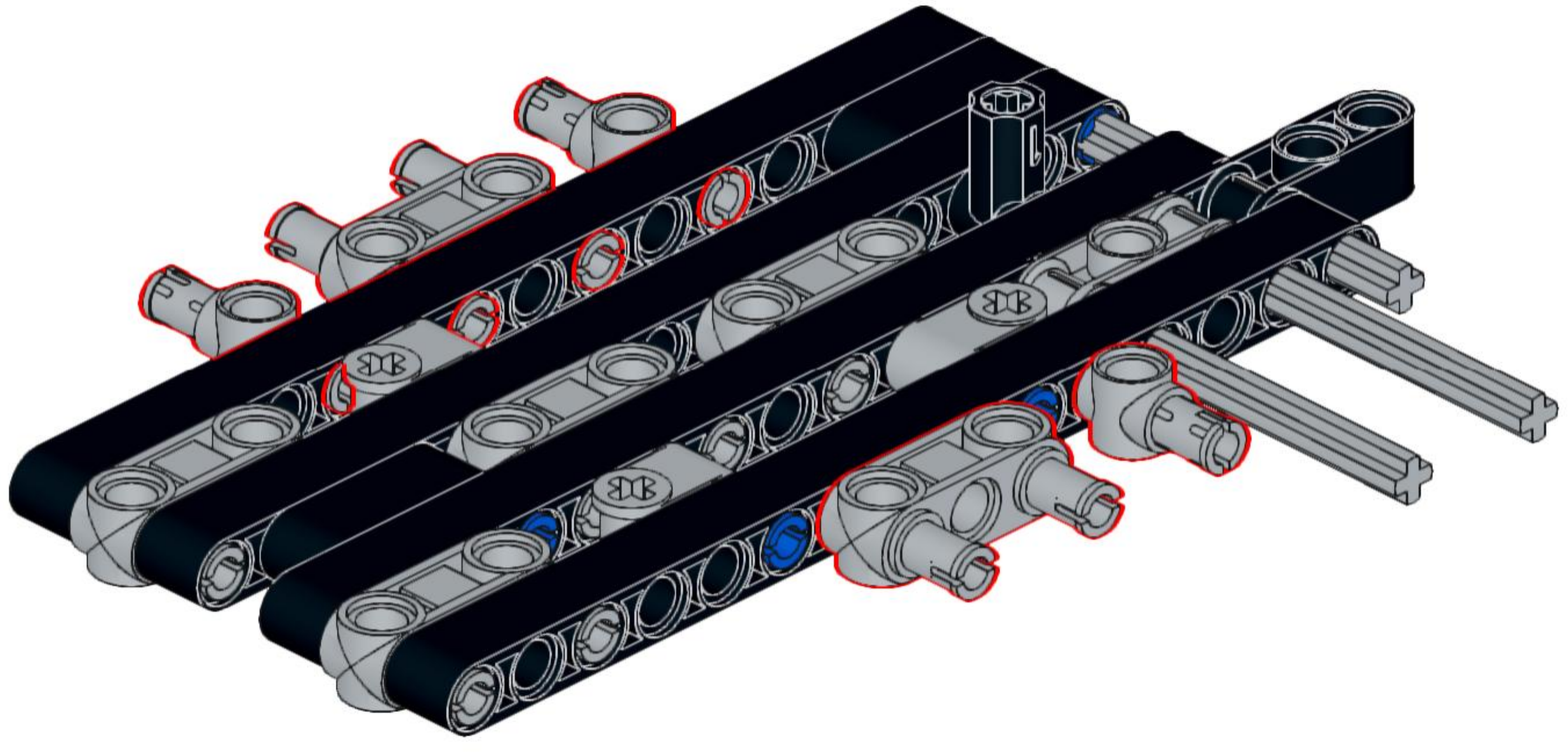

## 12

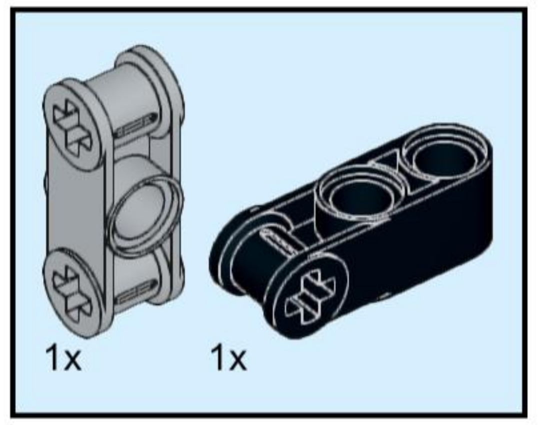


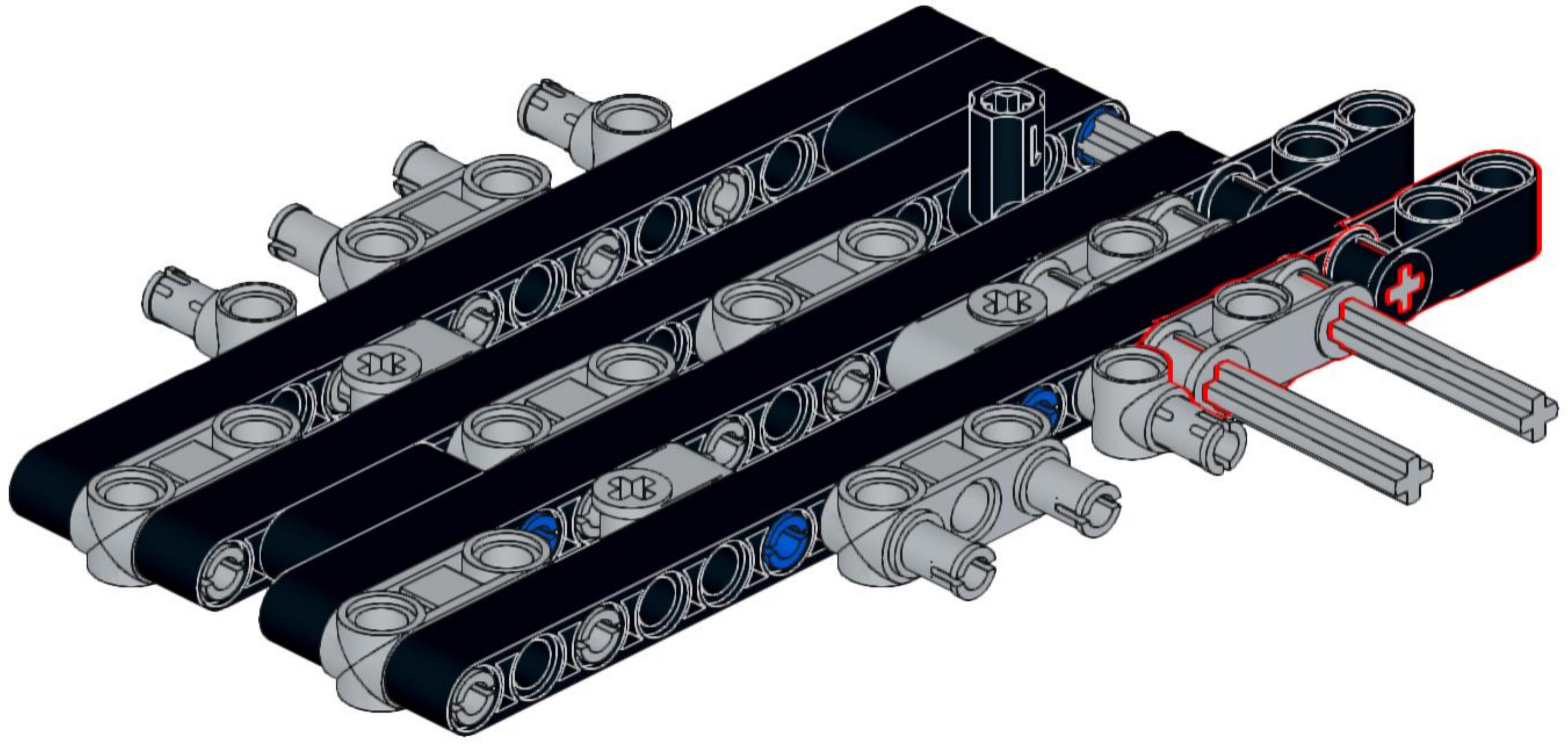

# 13

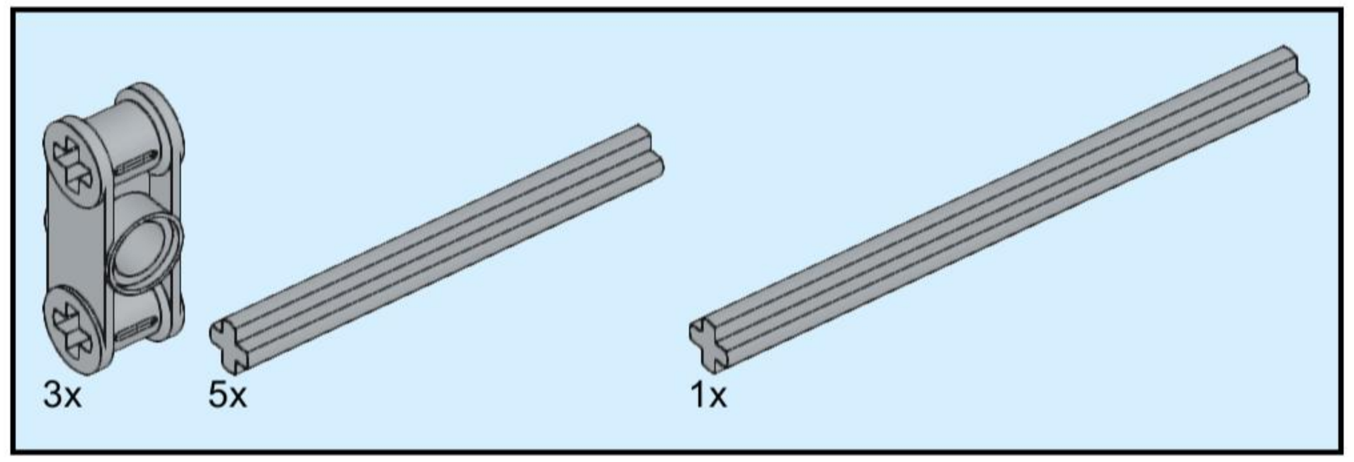


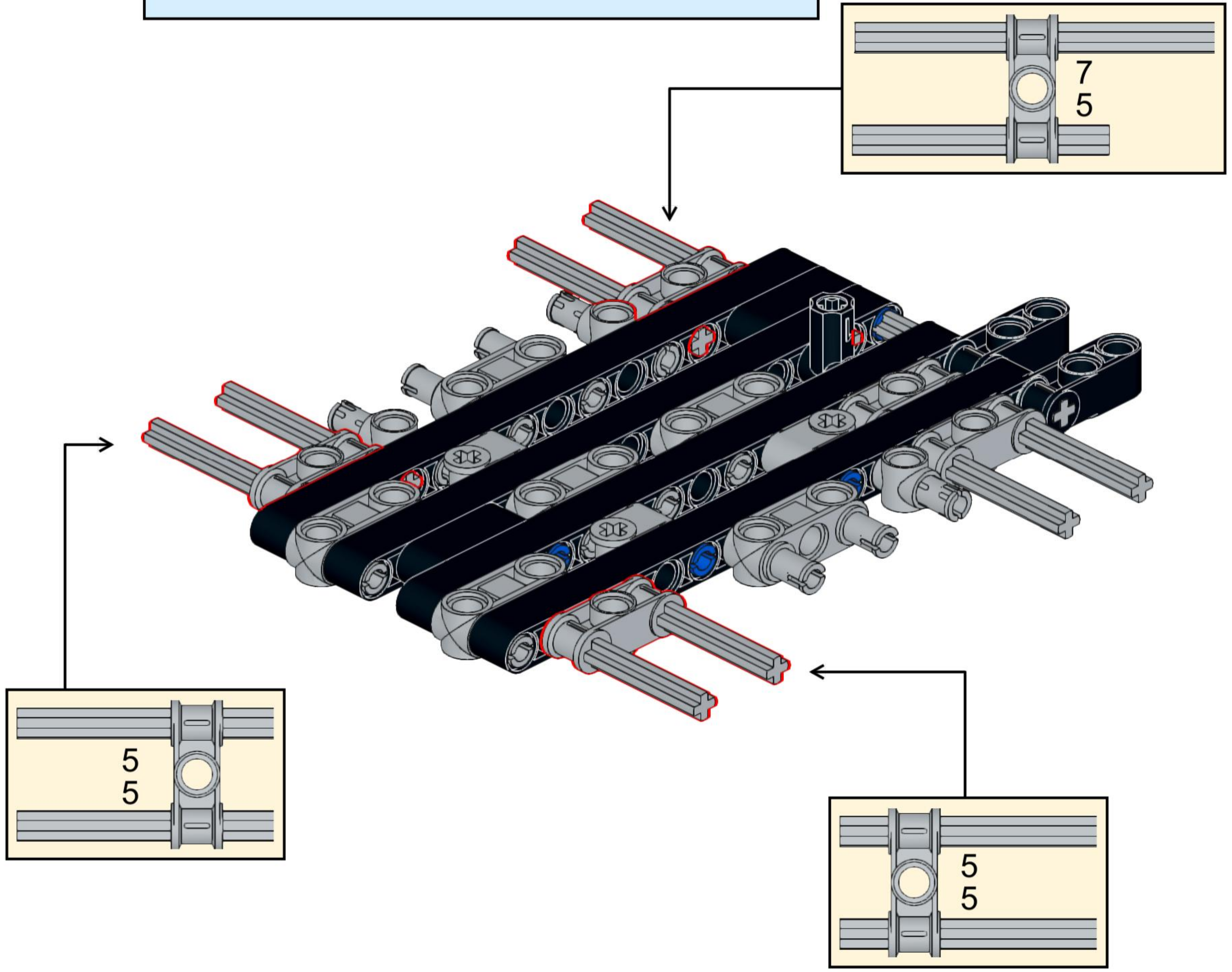


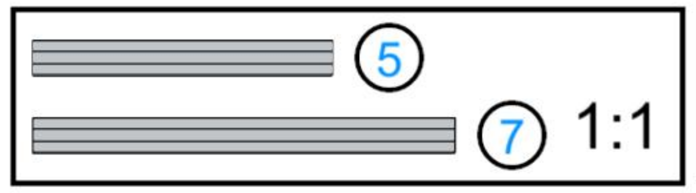


# 14

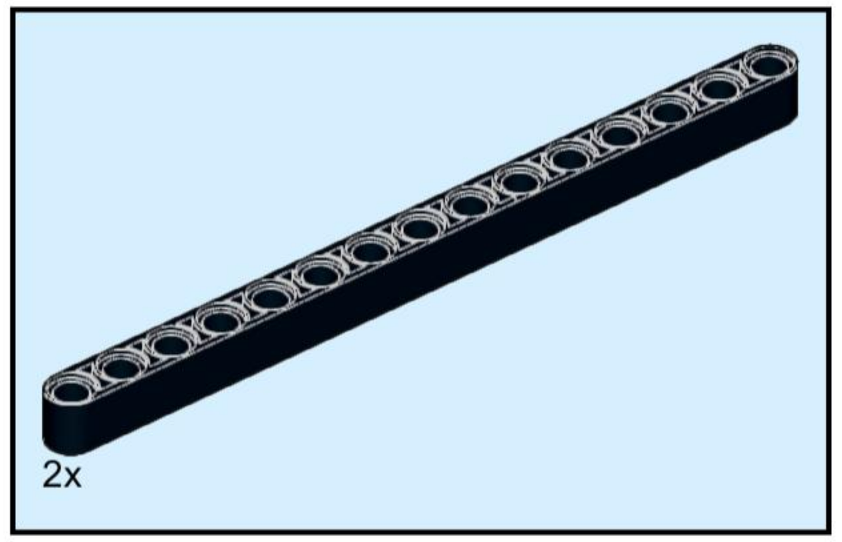


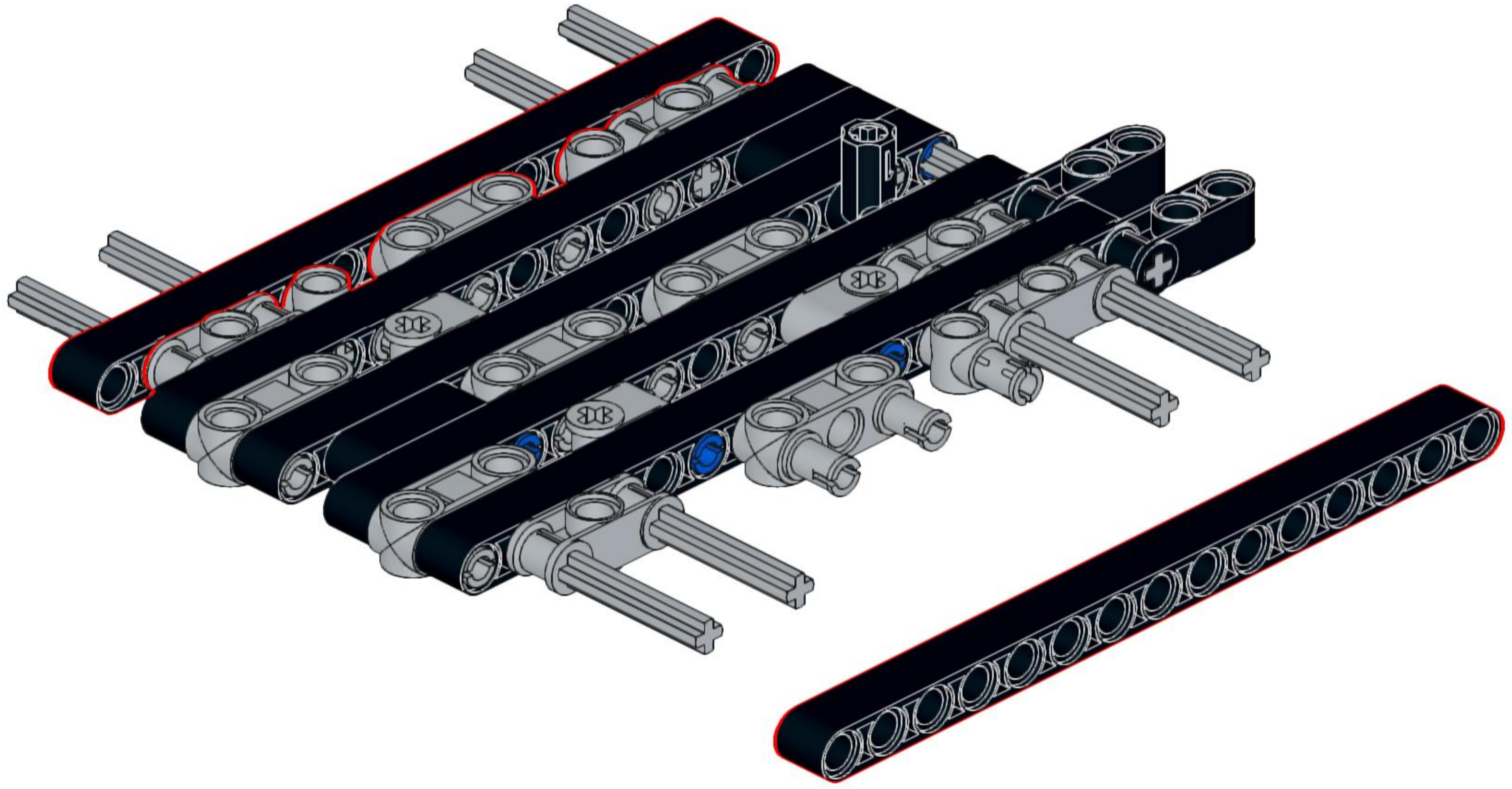

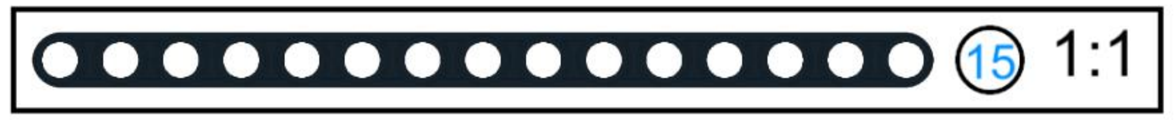

# 15

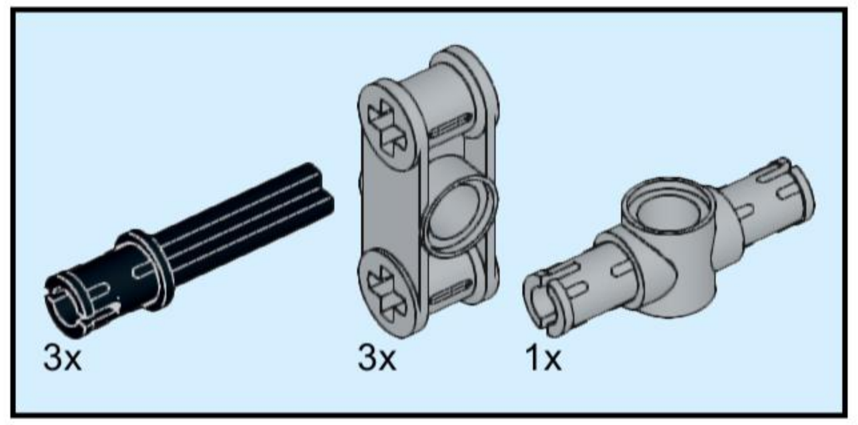


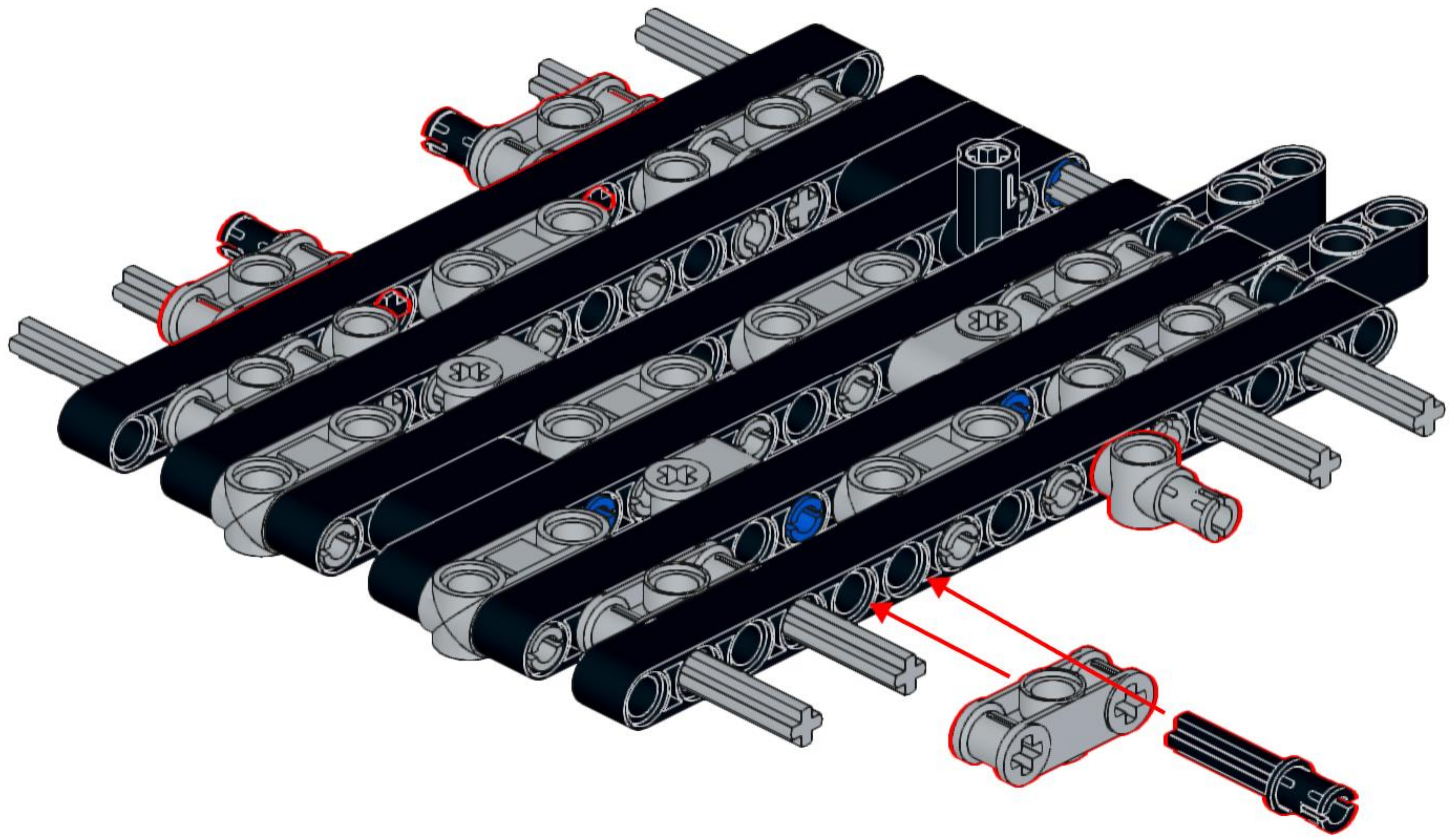

# 16

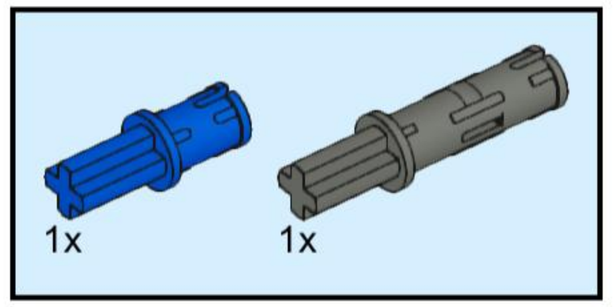


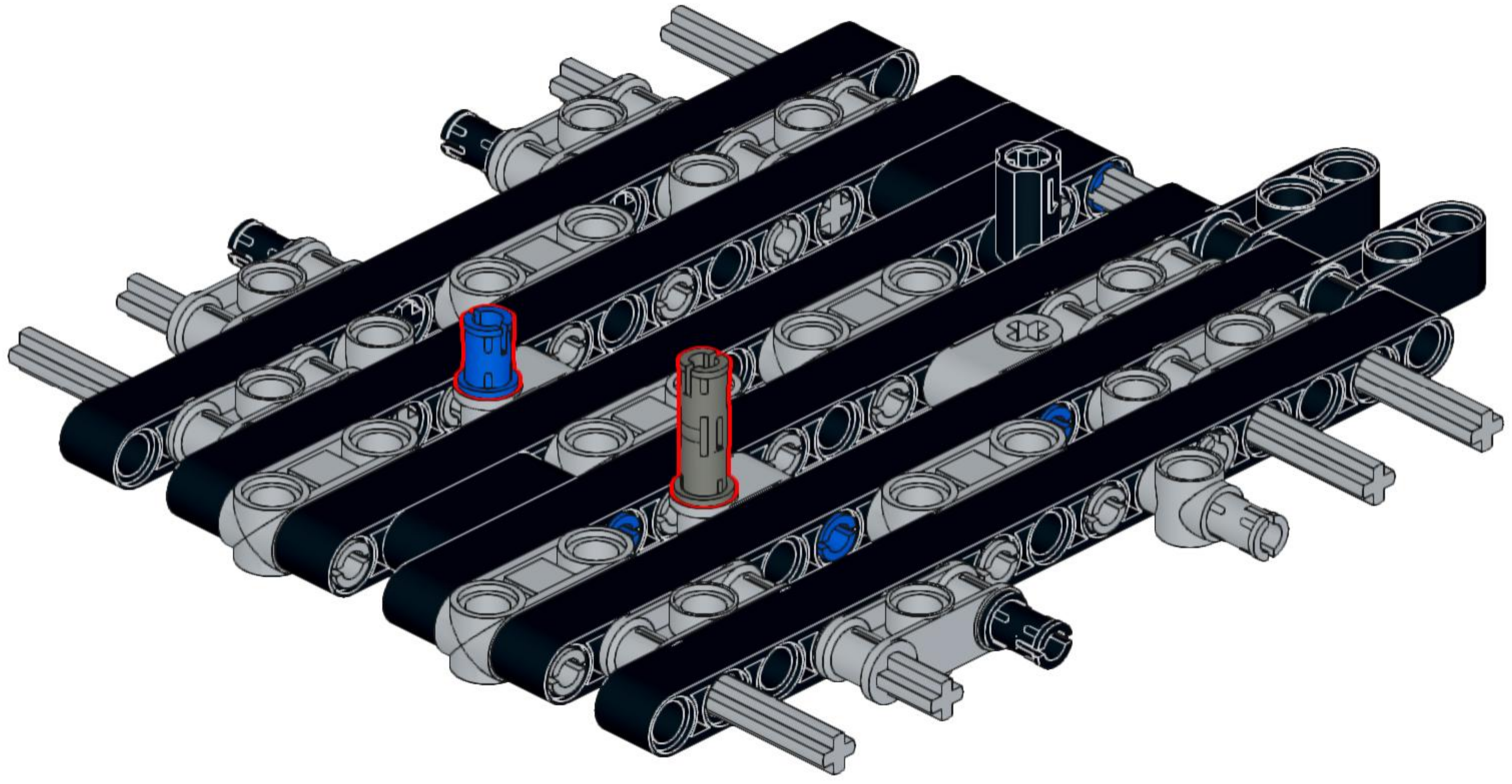

17

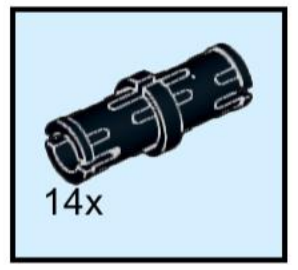


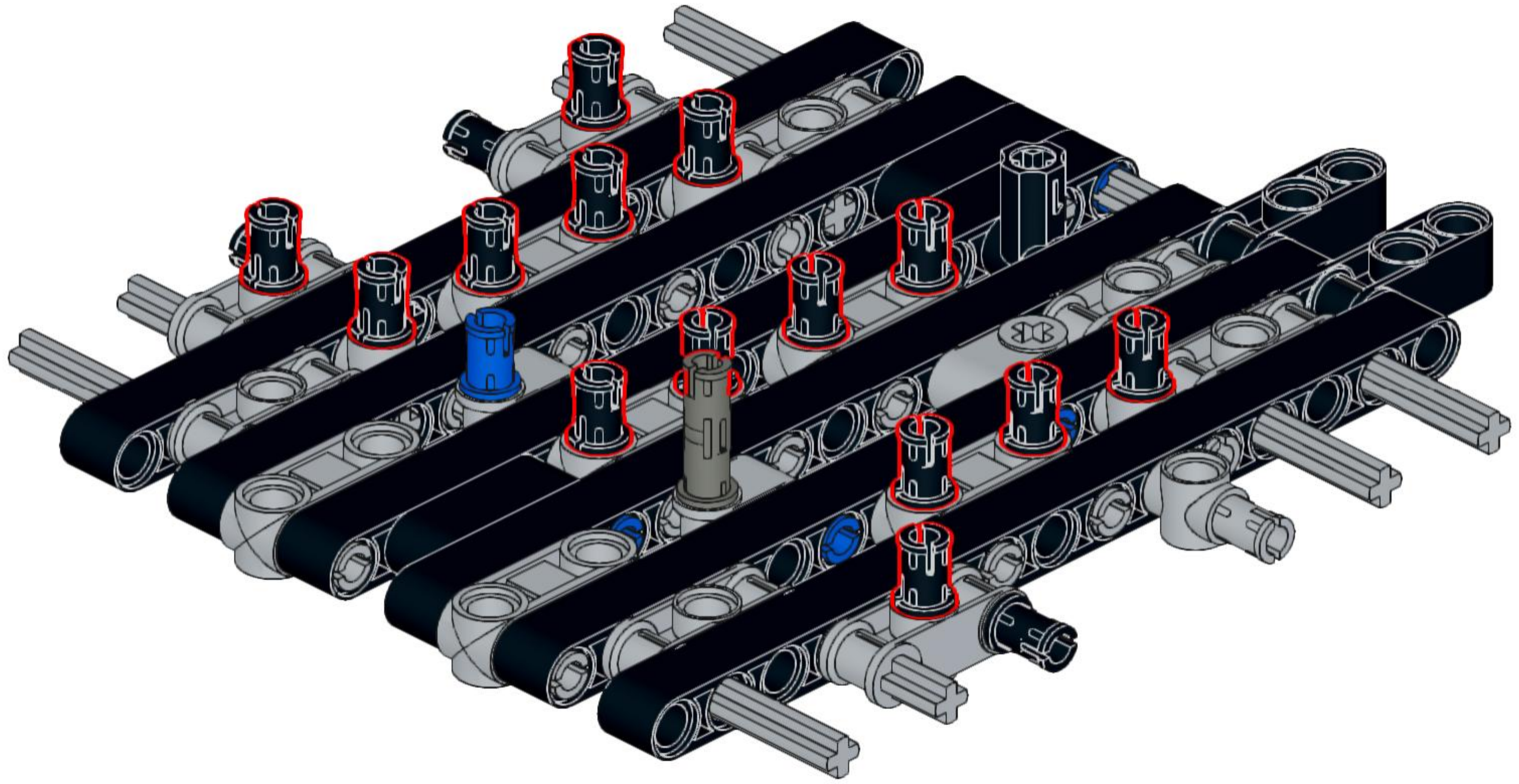

18

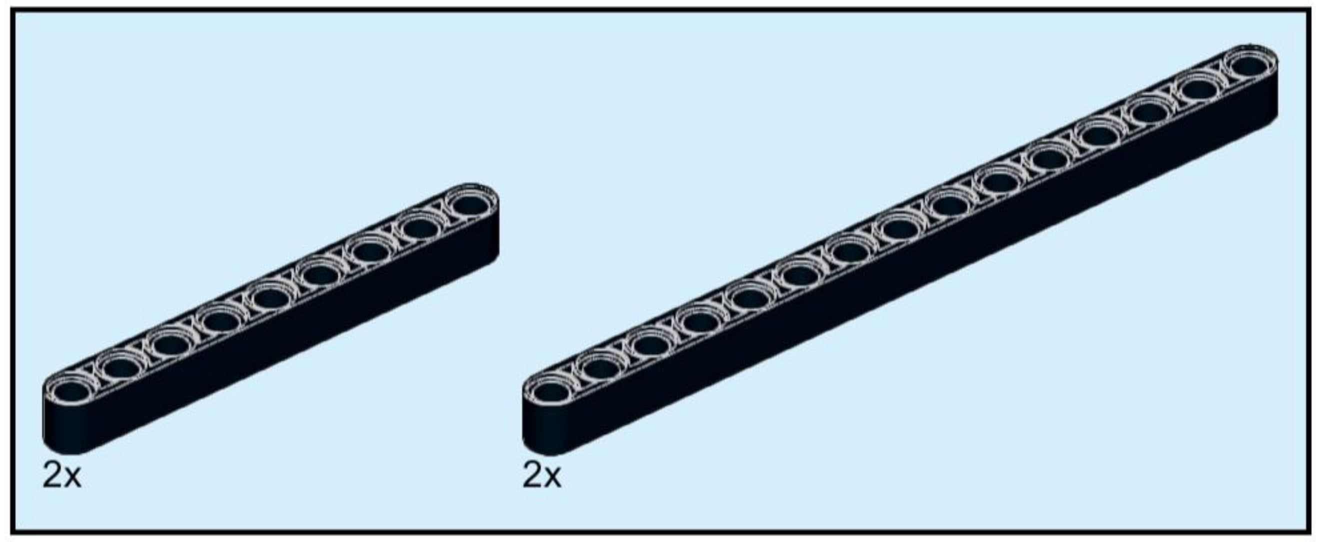


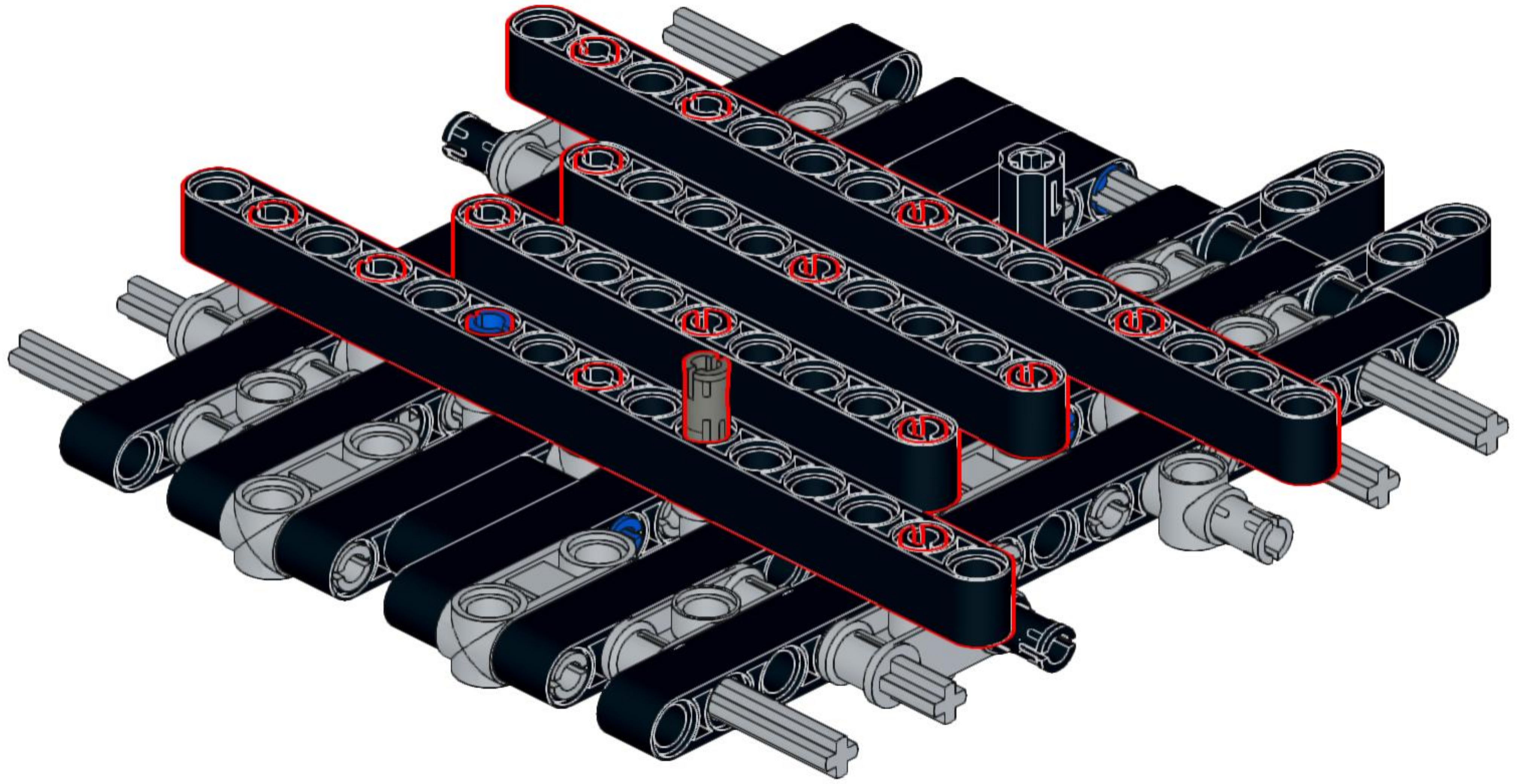

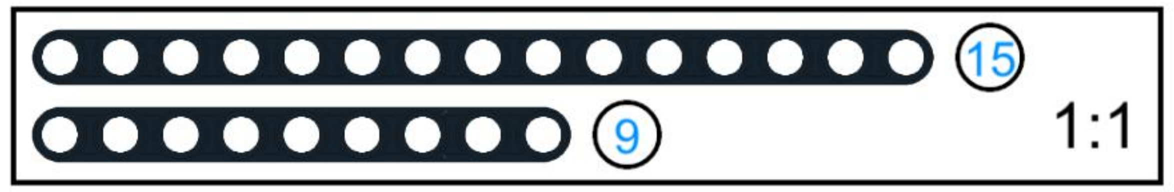

# 19

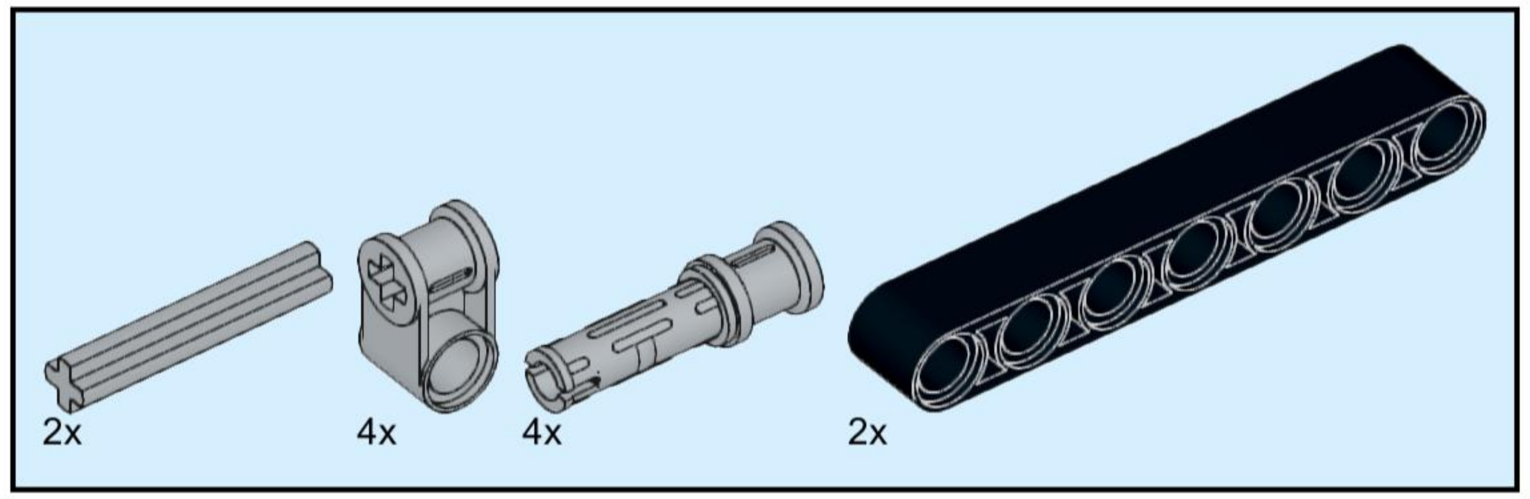


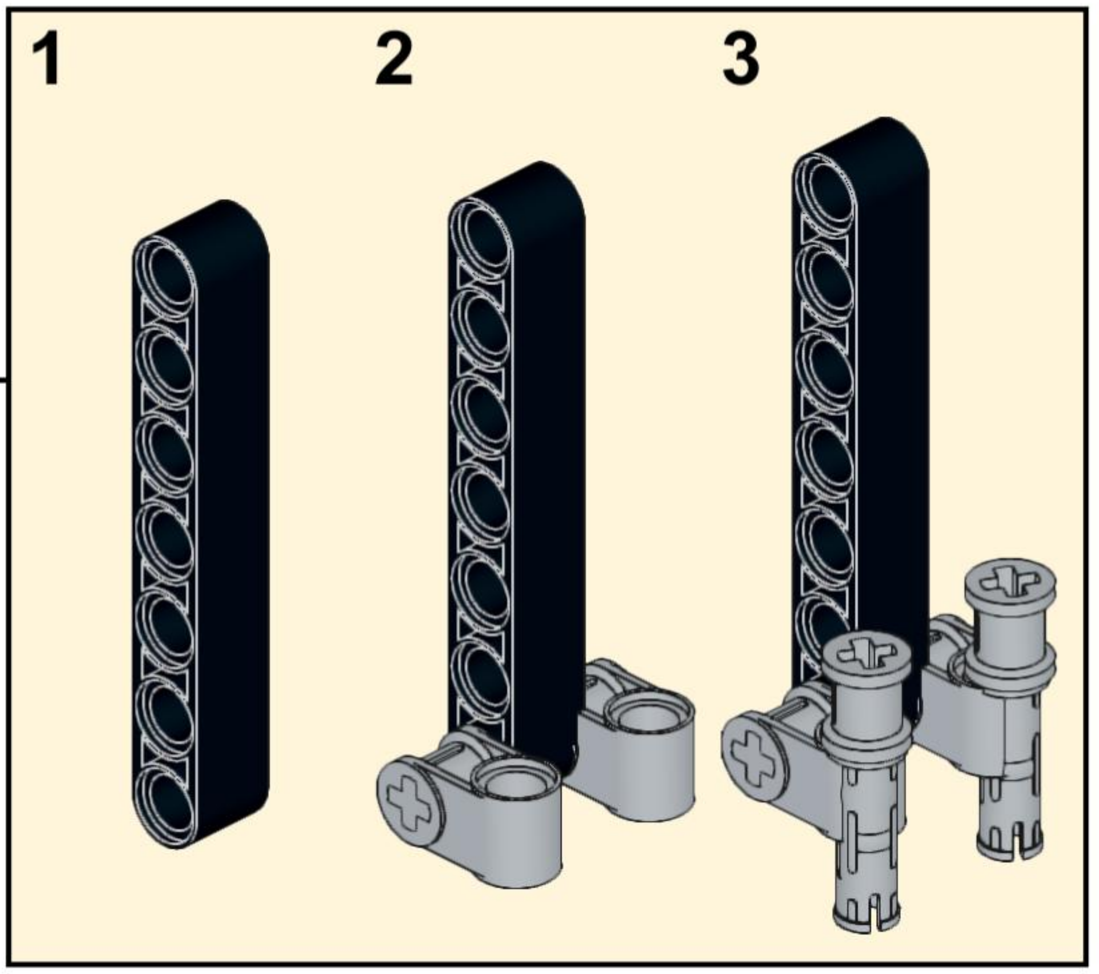


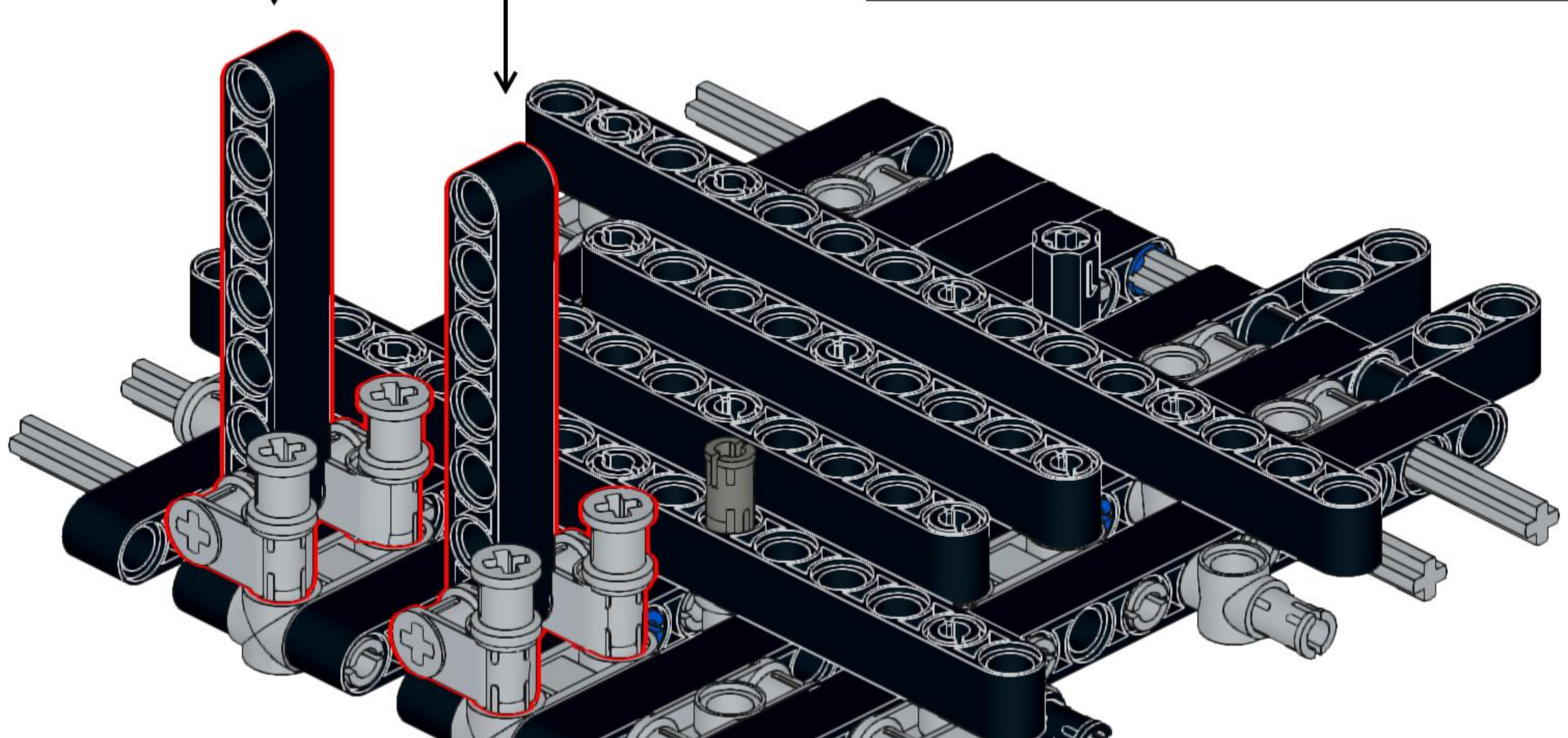

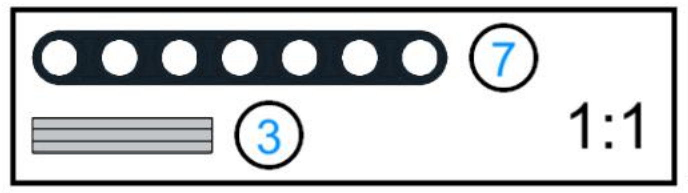


# 20

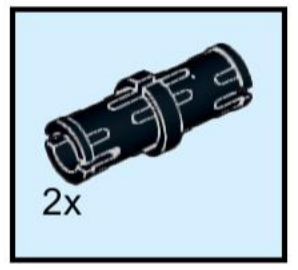


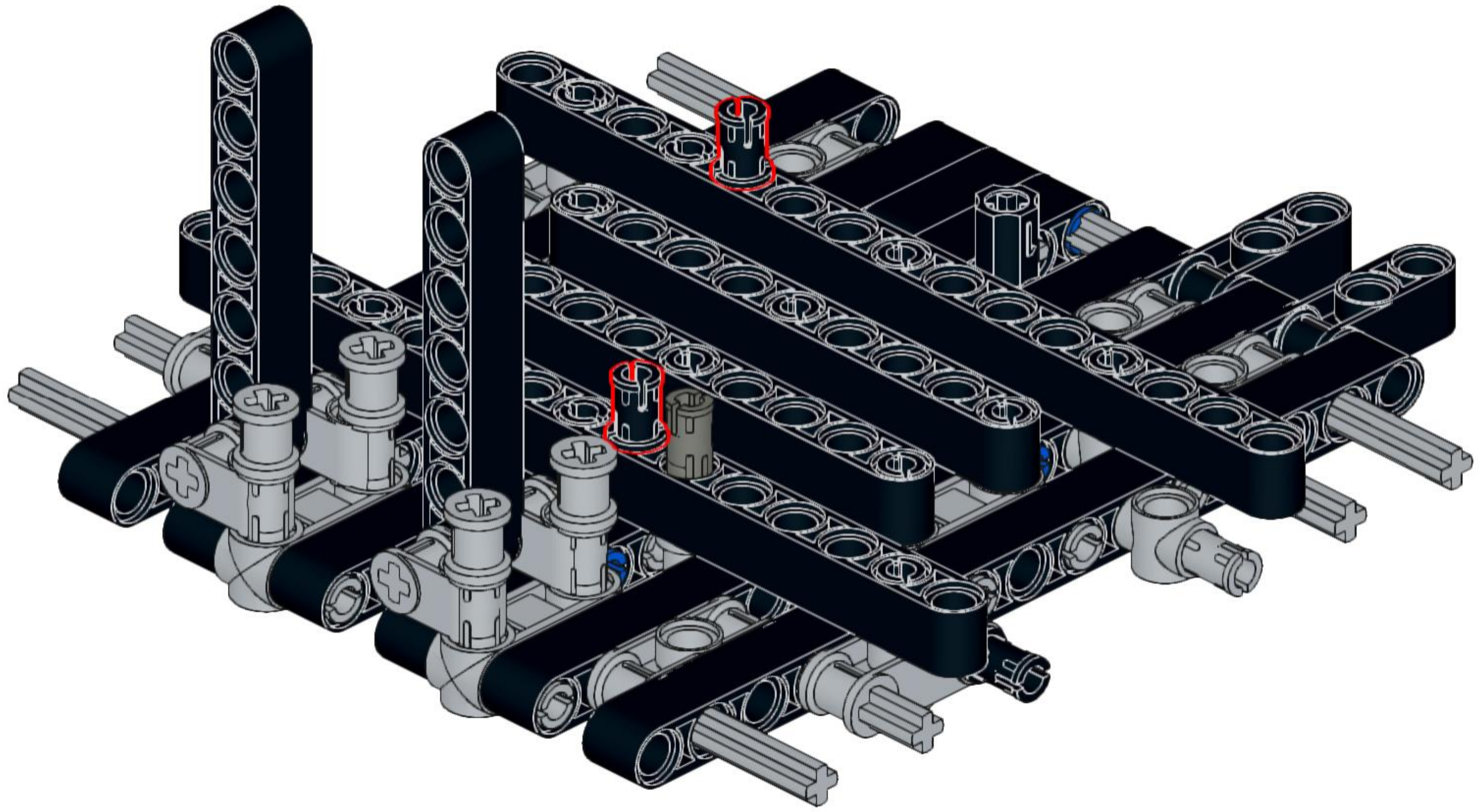

# 21

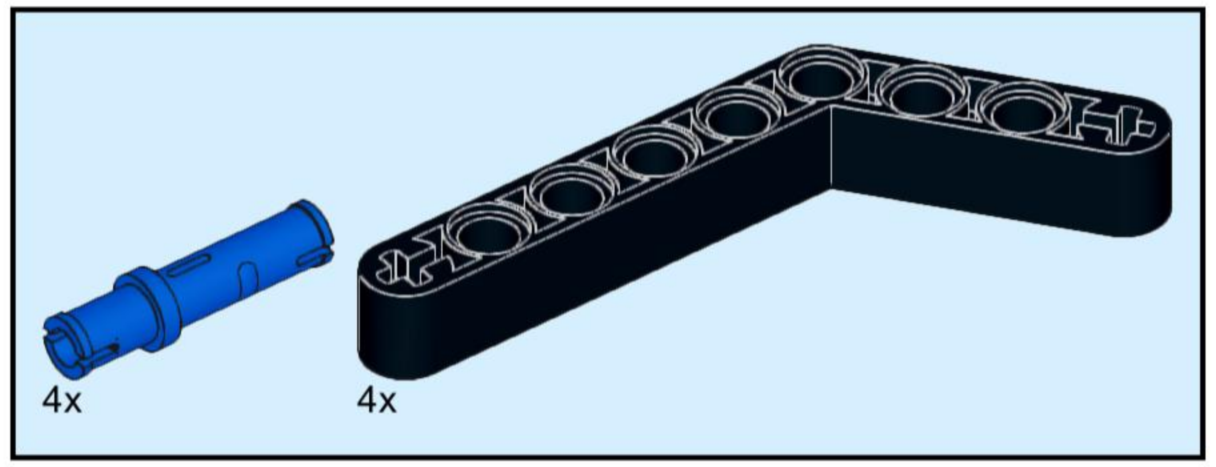


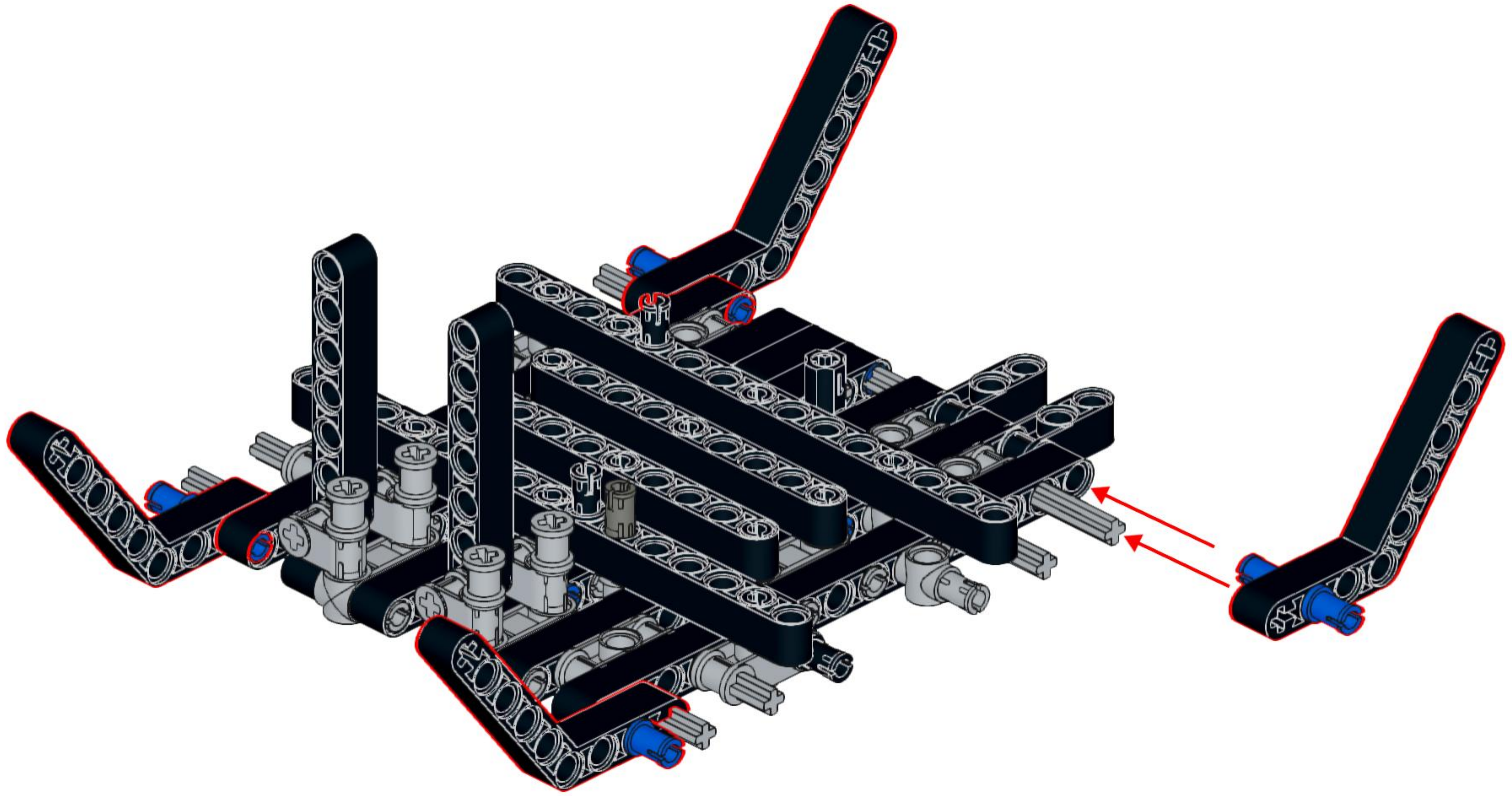

# 22

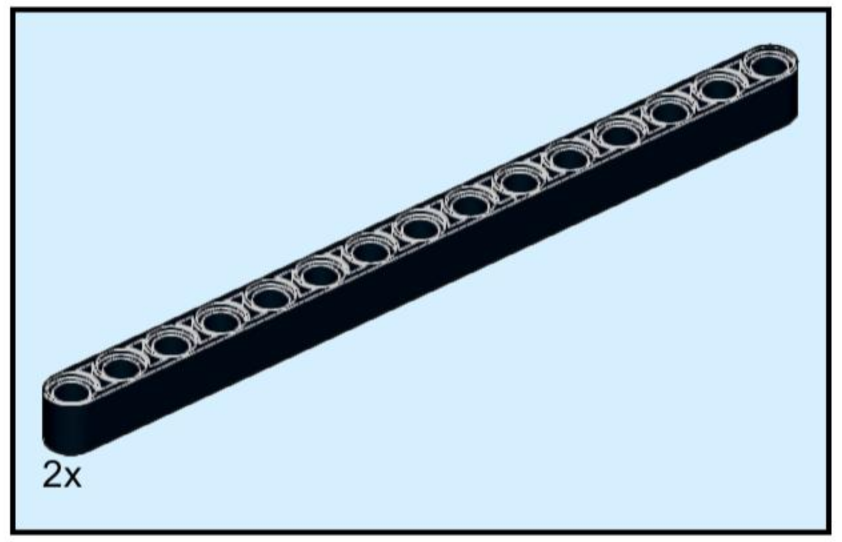


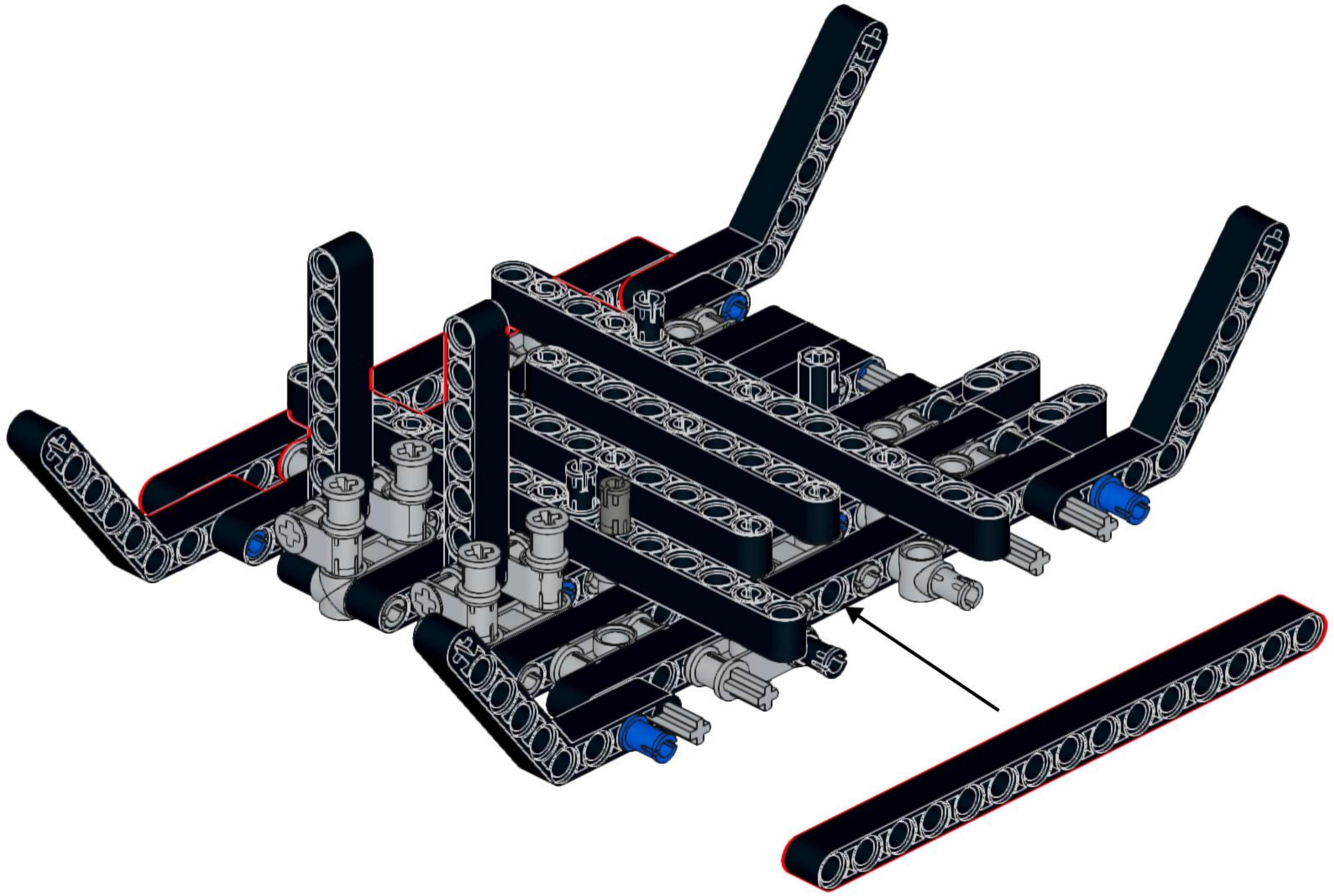

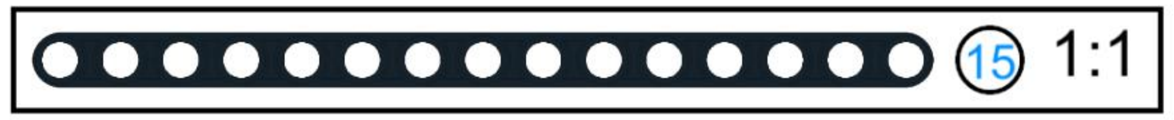

23

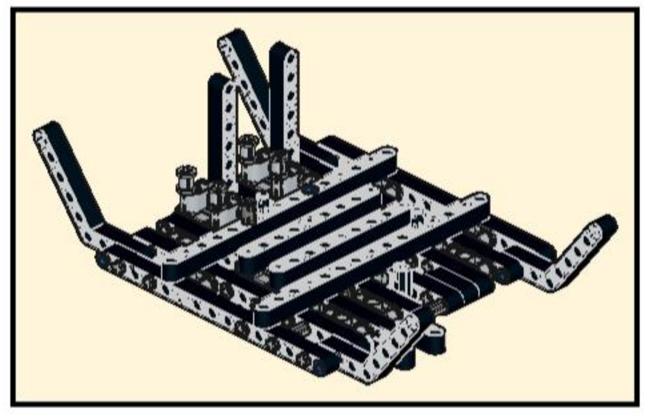

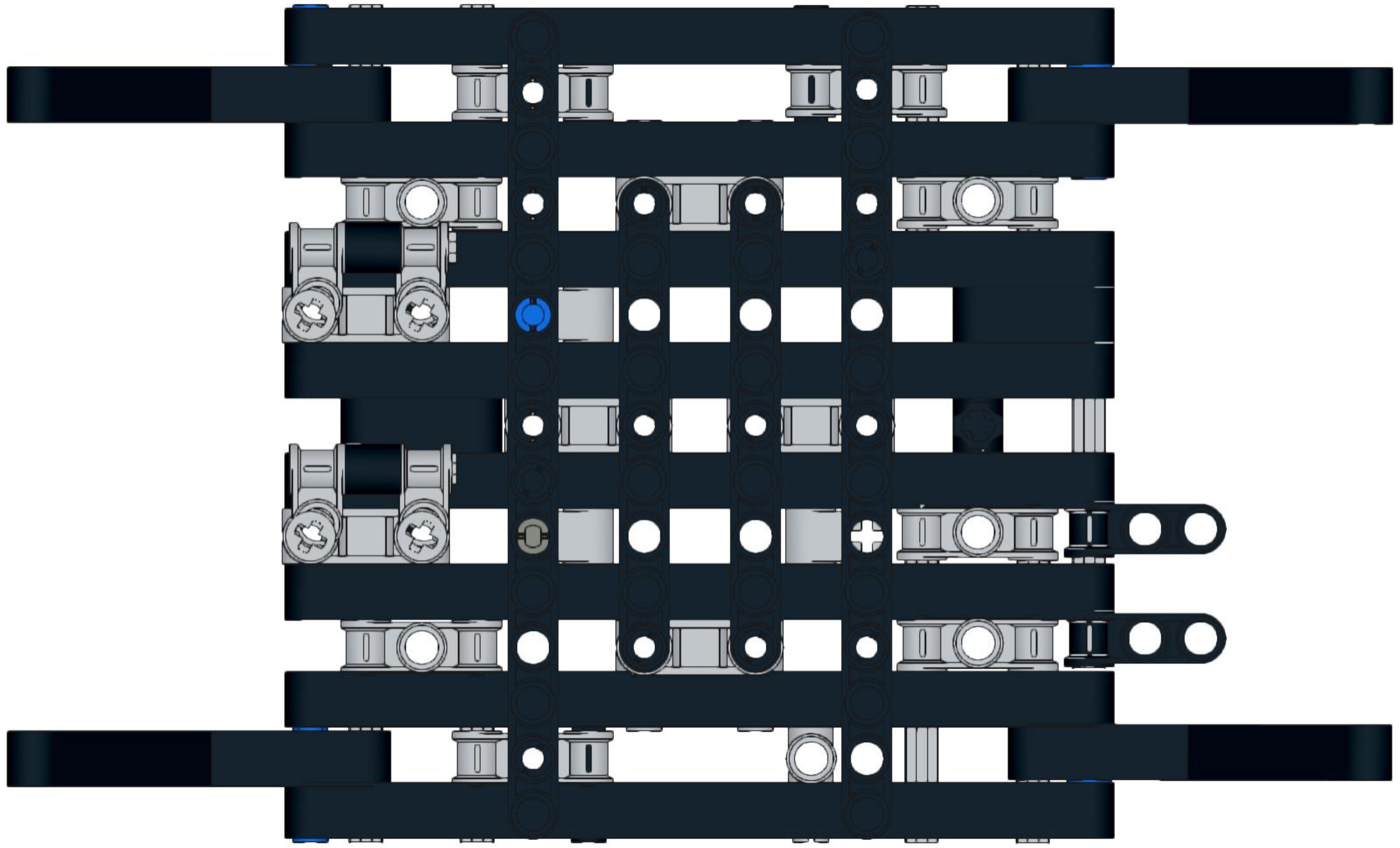

24

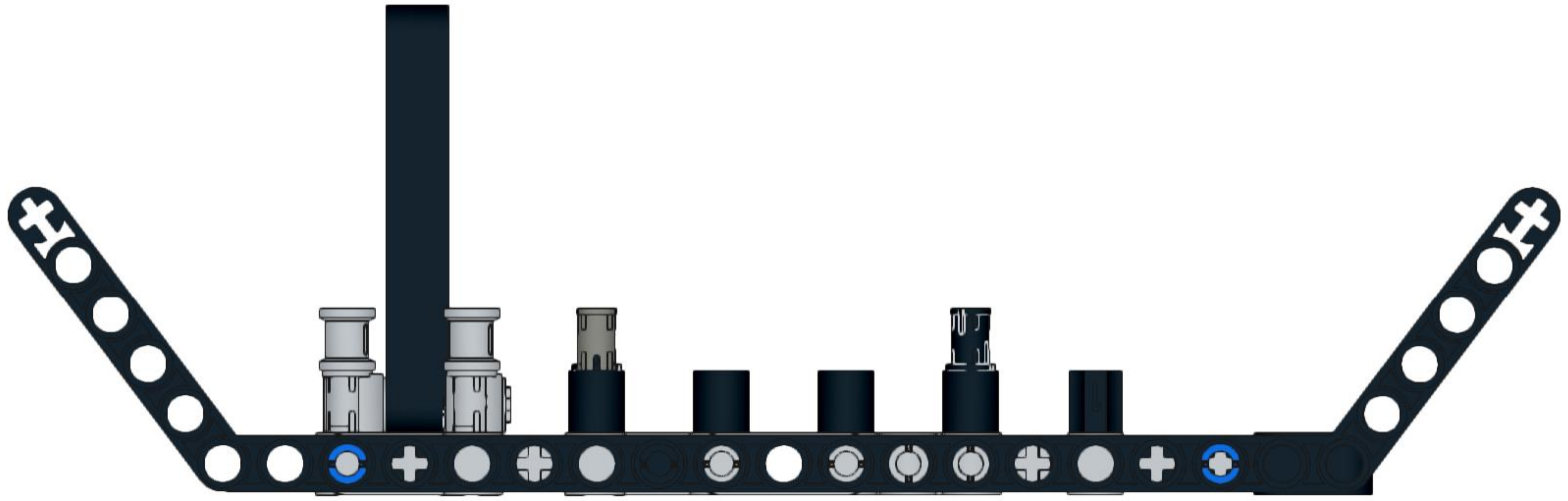

# 25

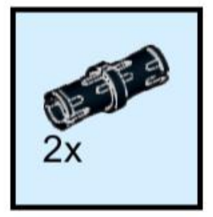


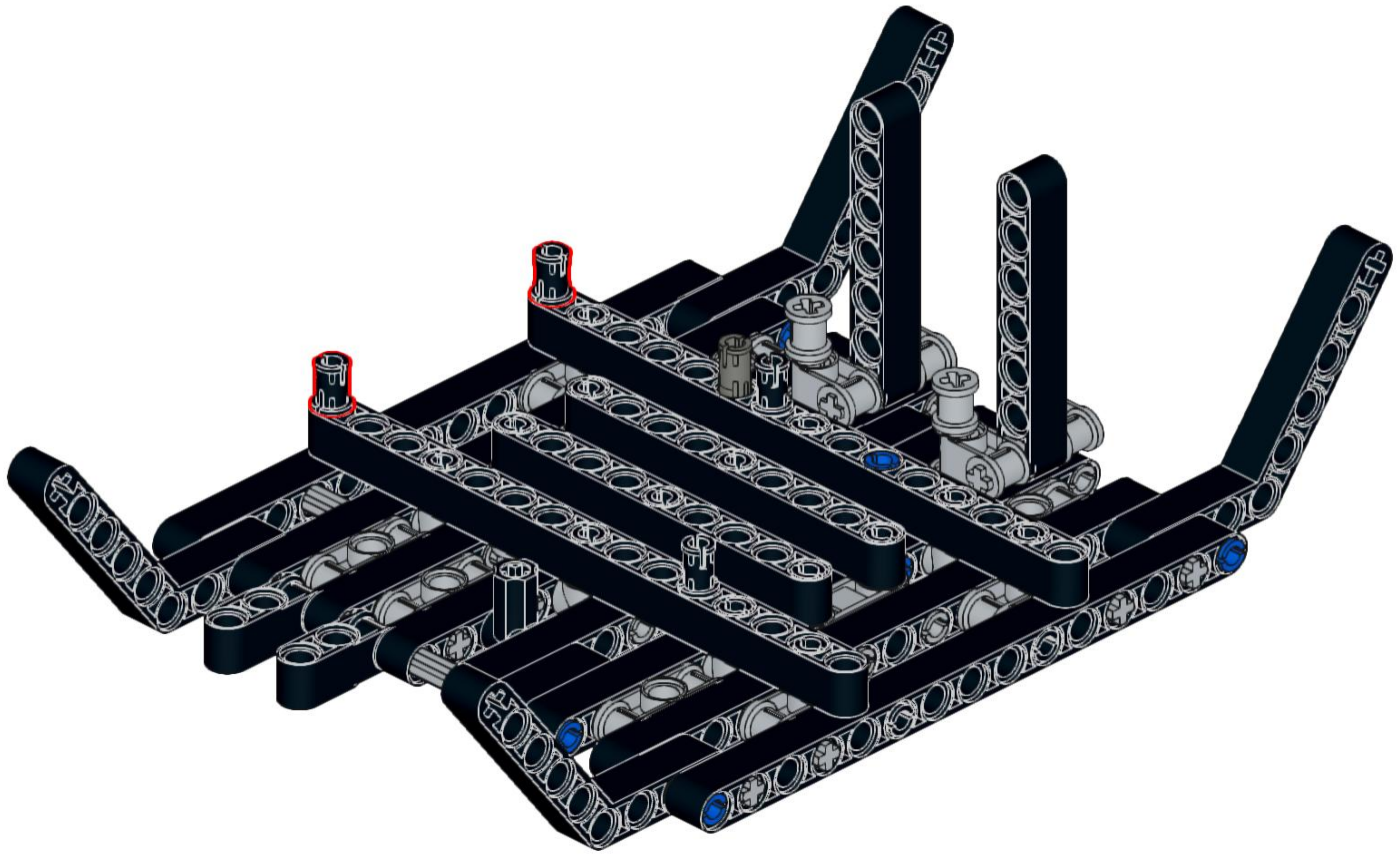

# 26

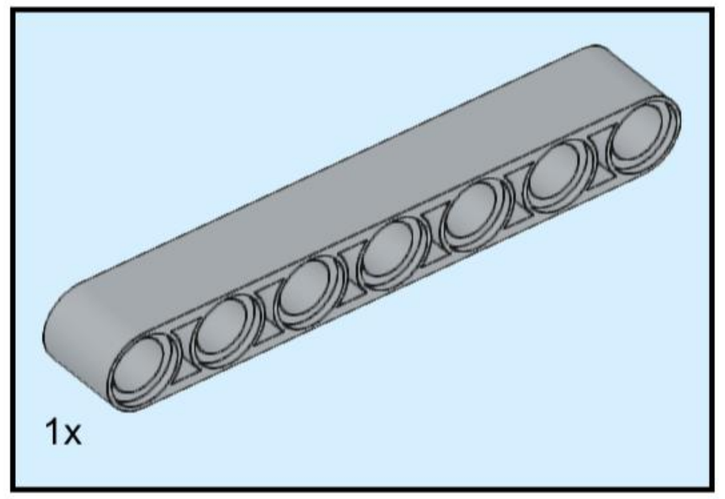


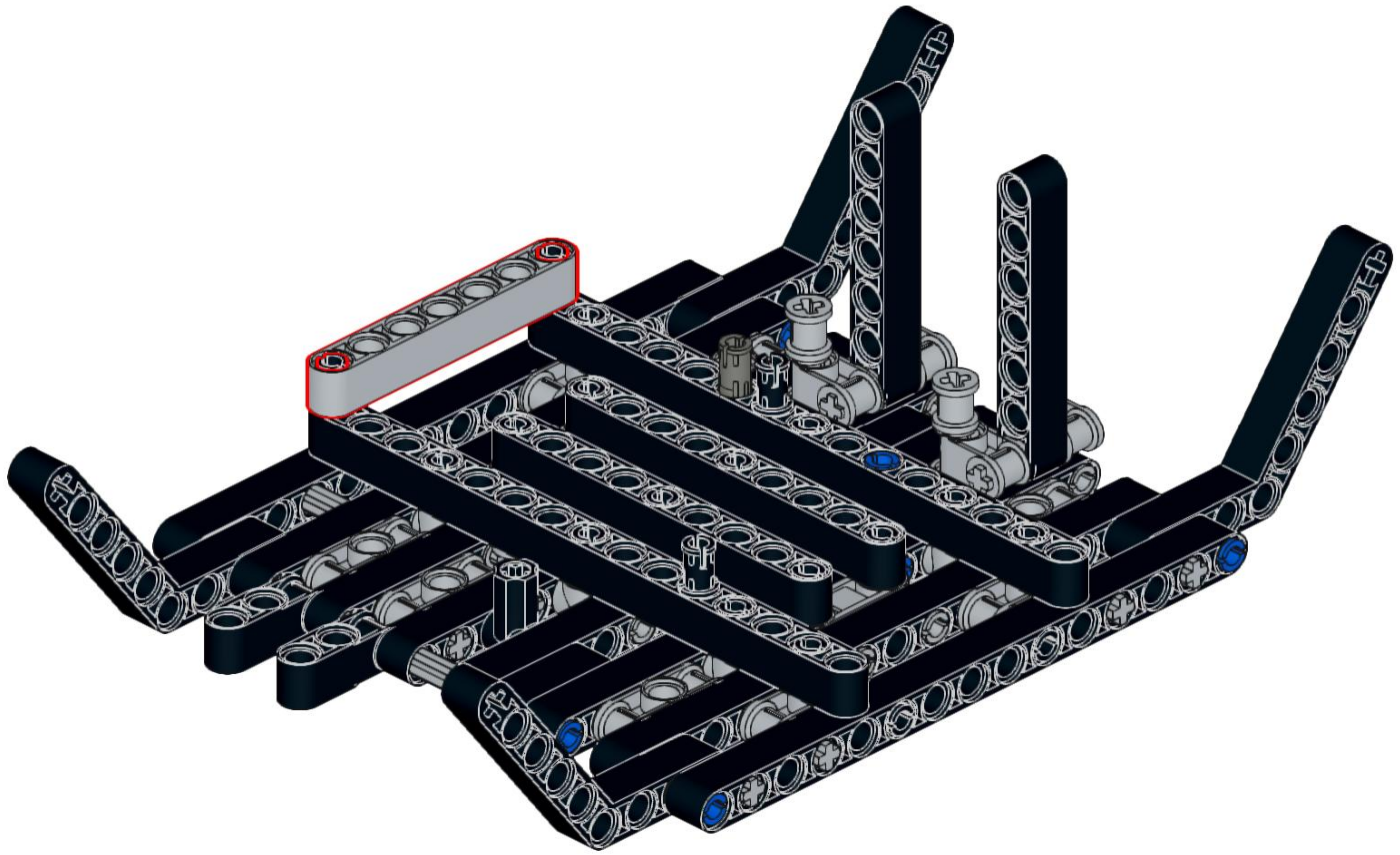

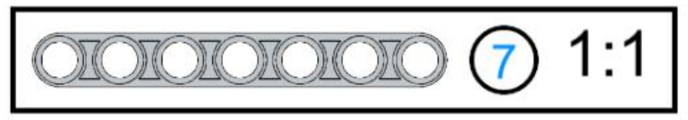

## 27

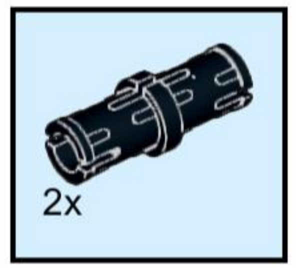


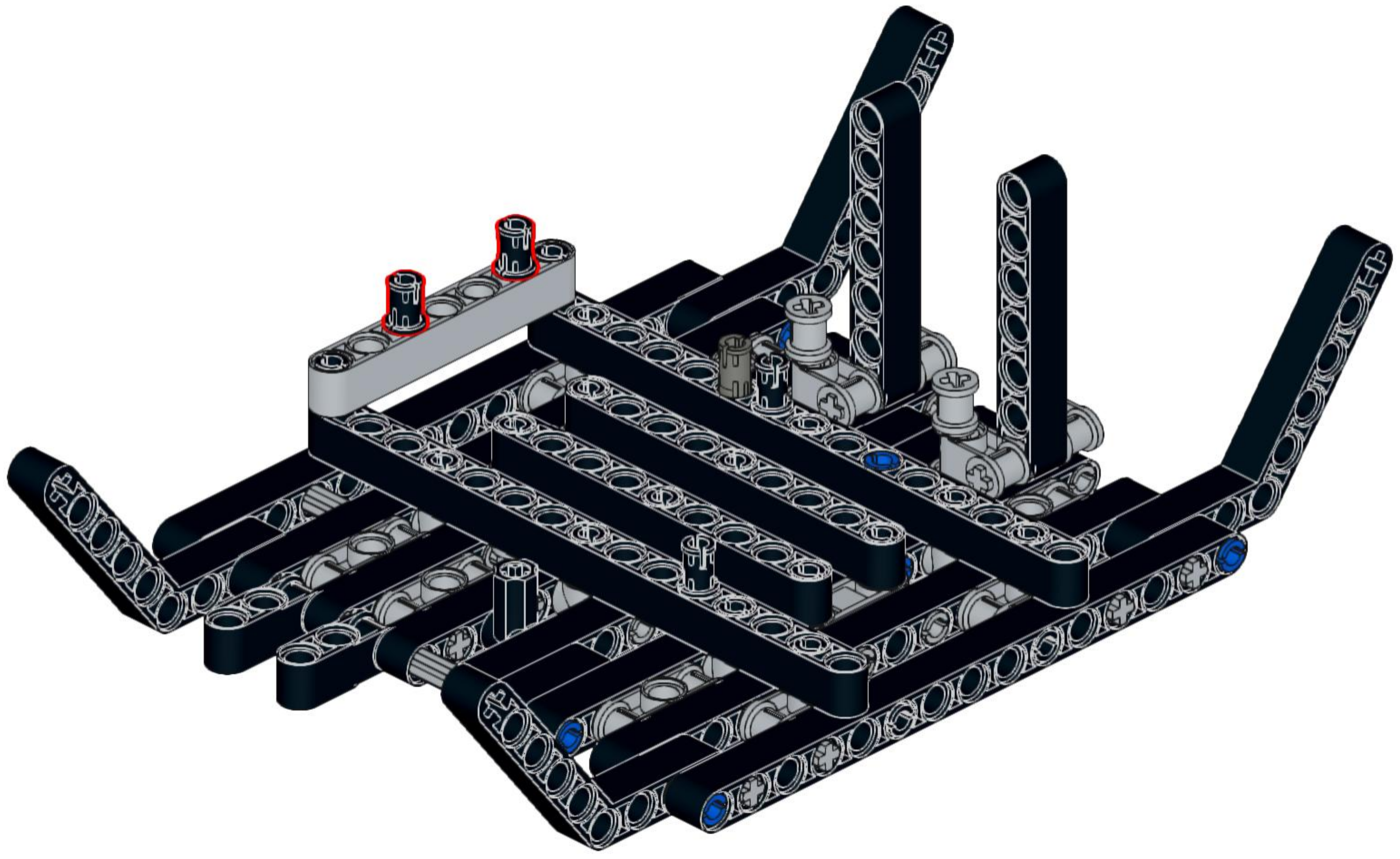

## 28

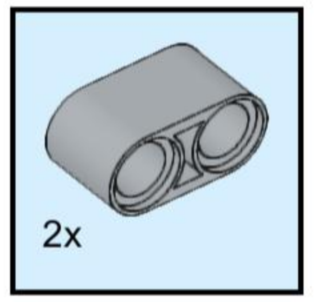


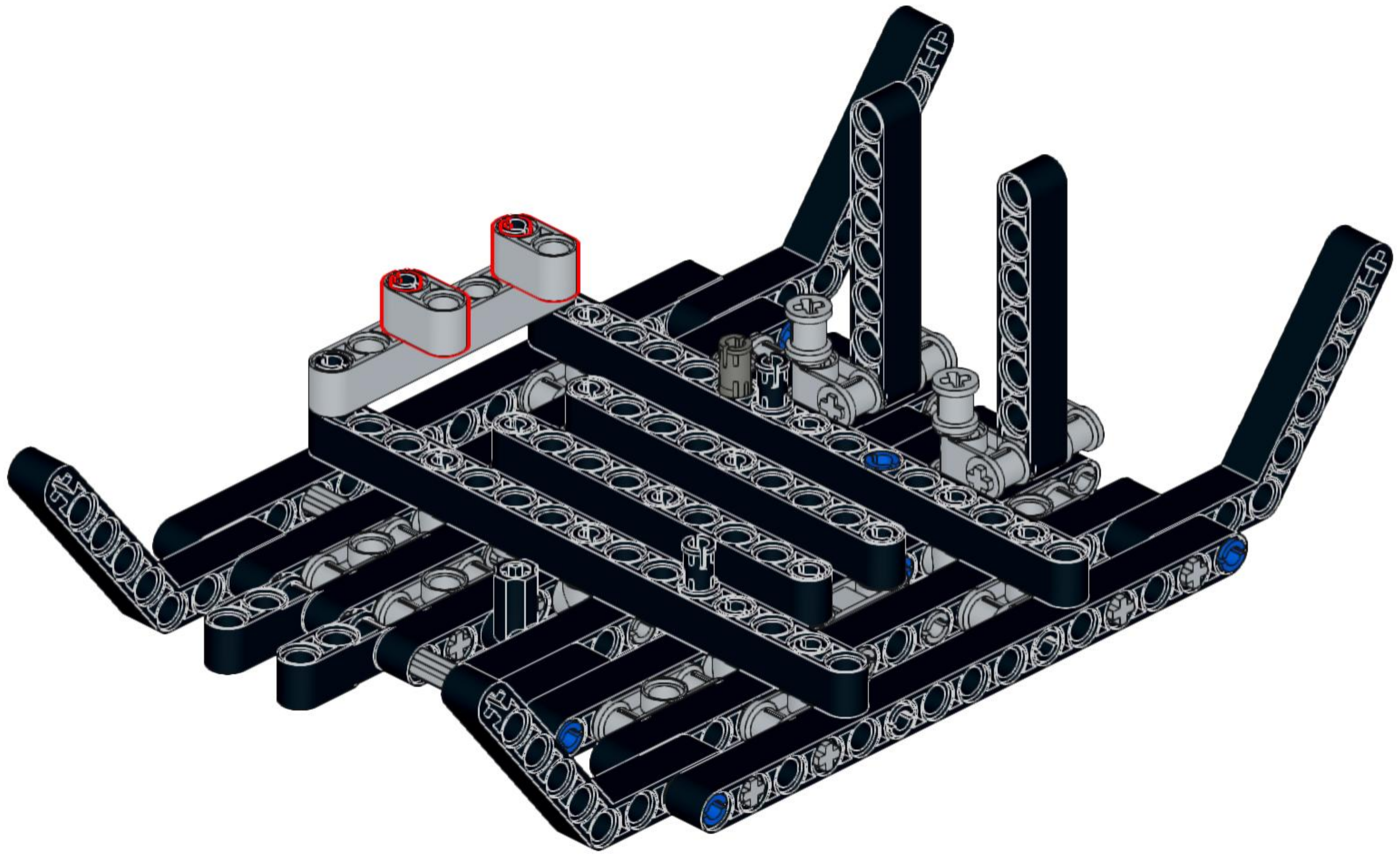

29

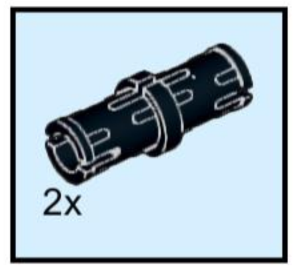


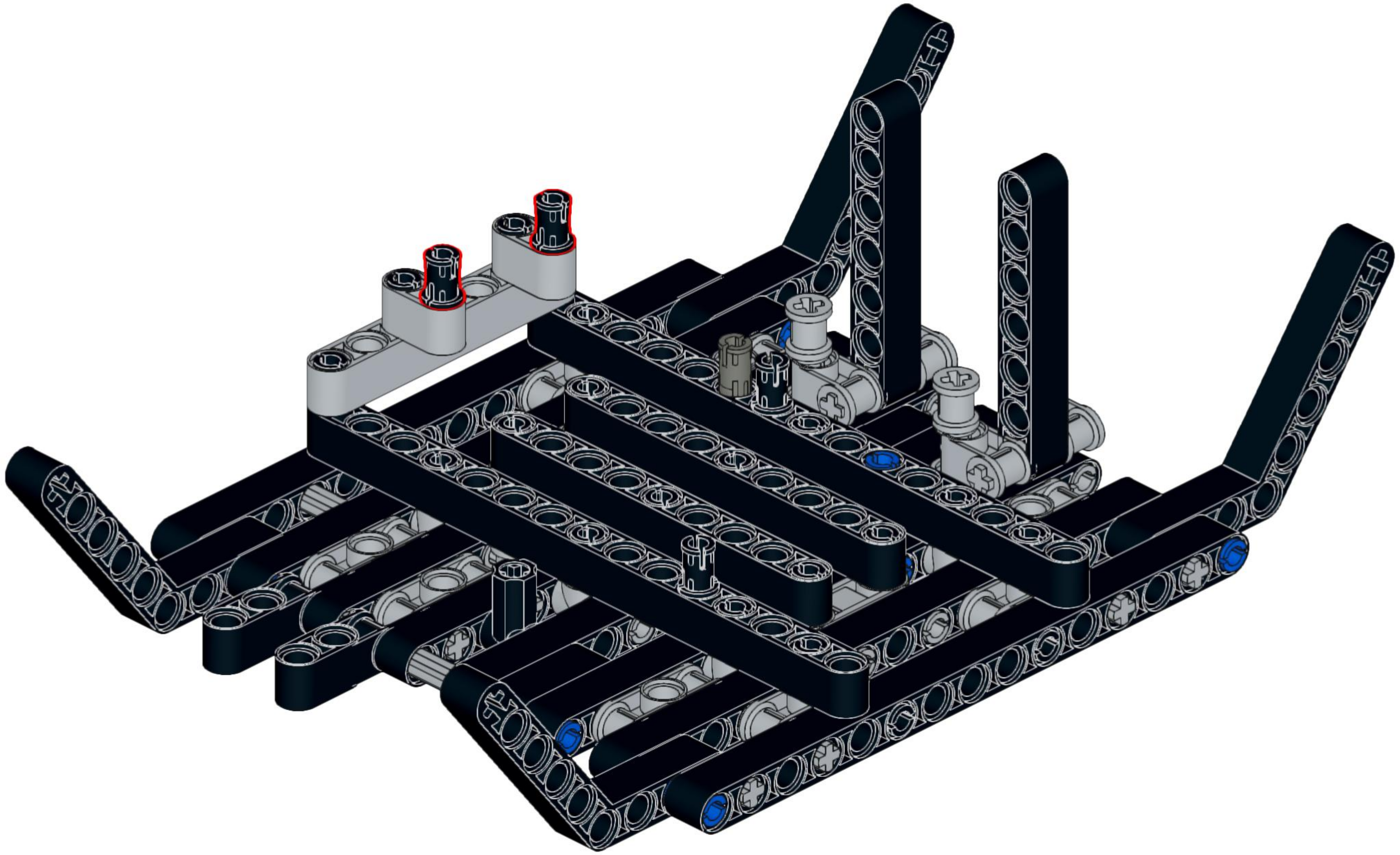

30

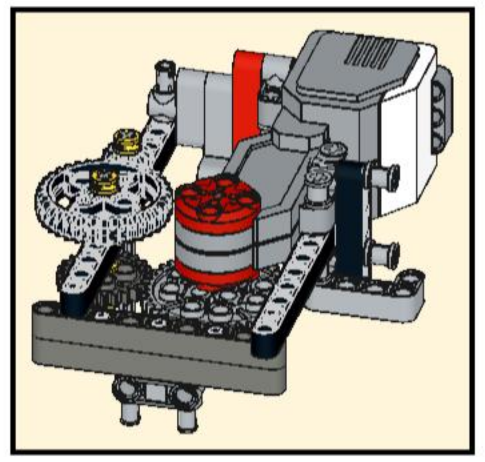

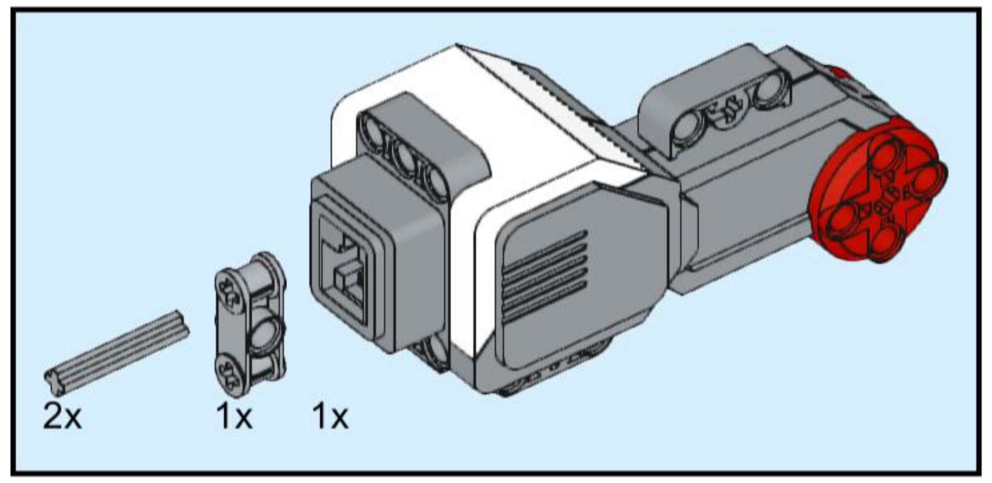


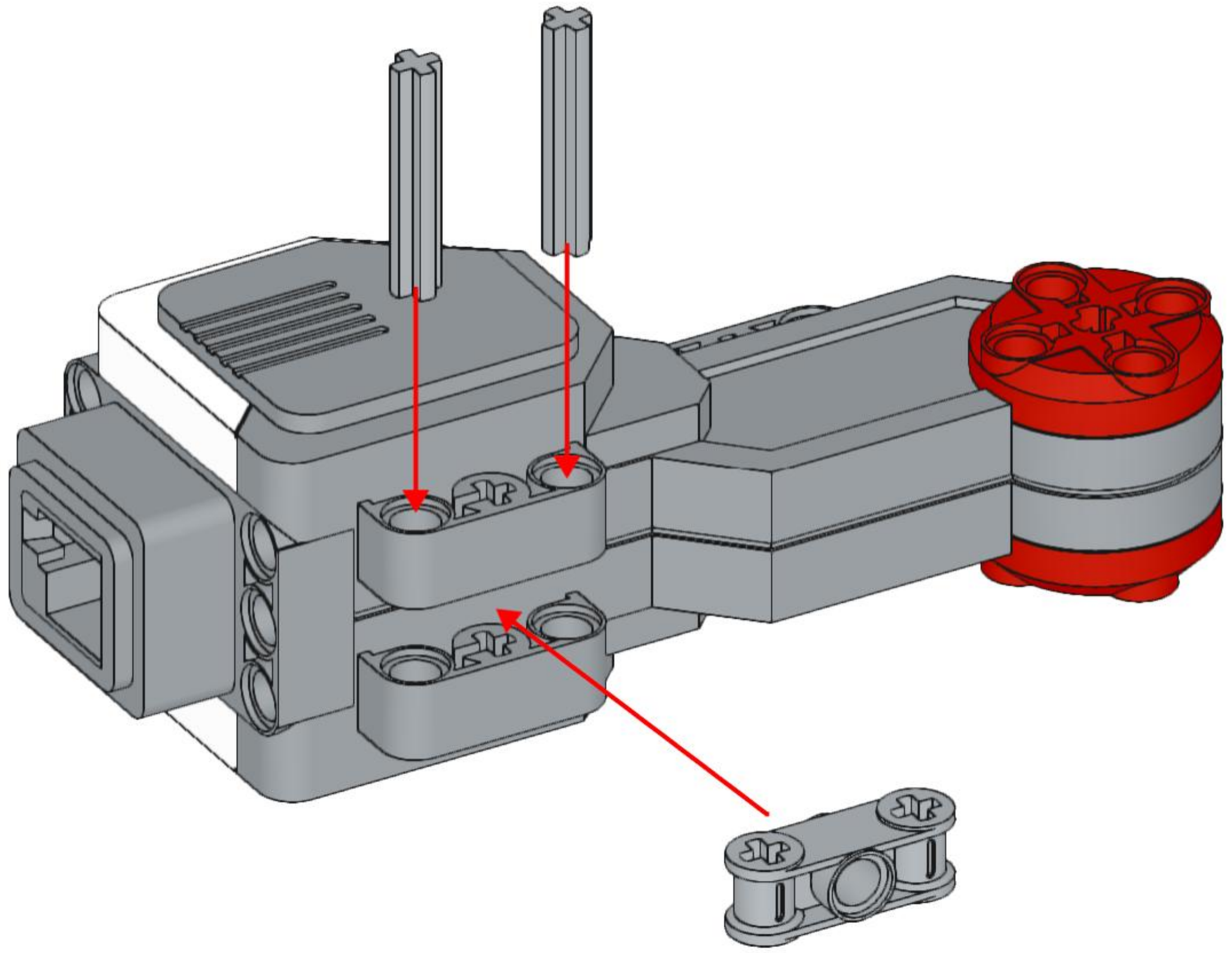

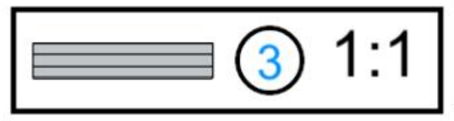


31

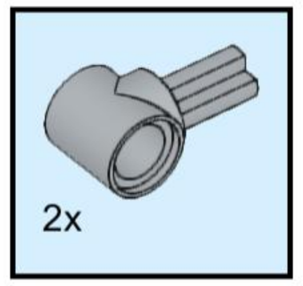


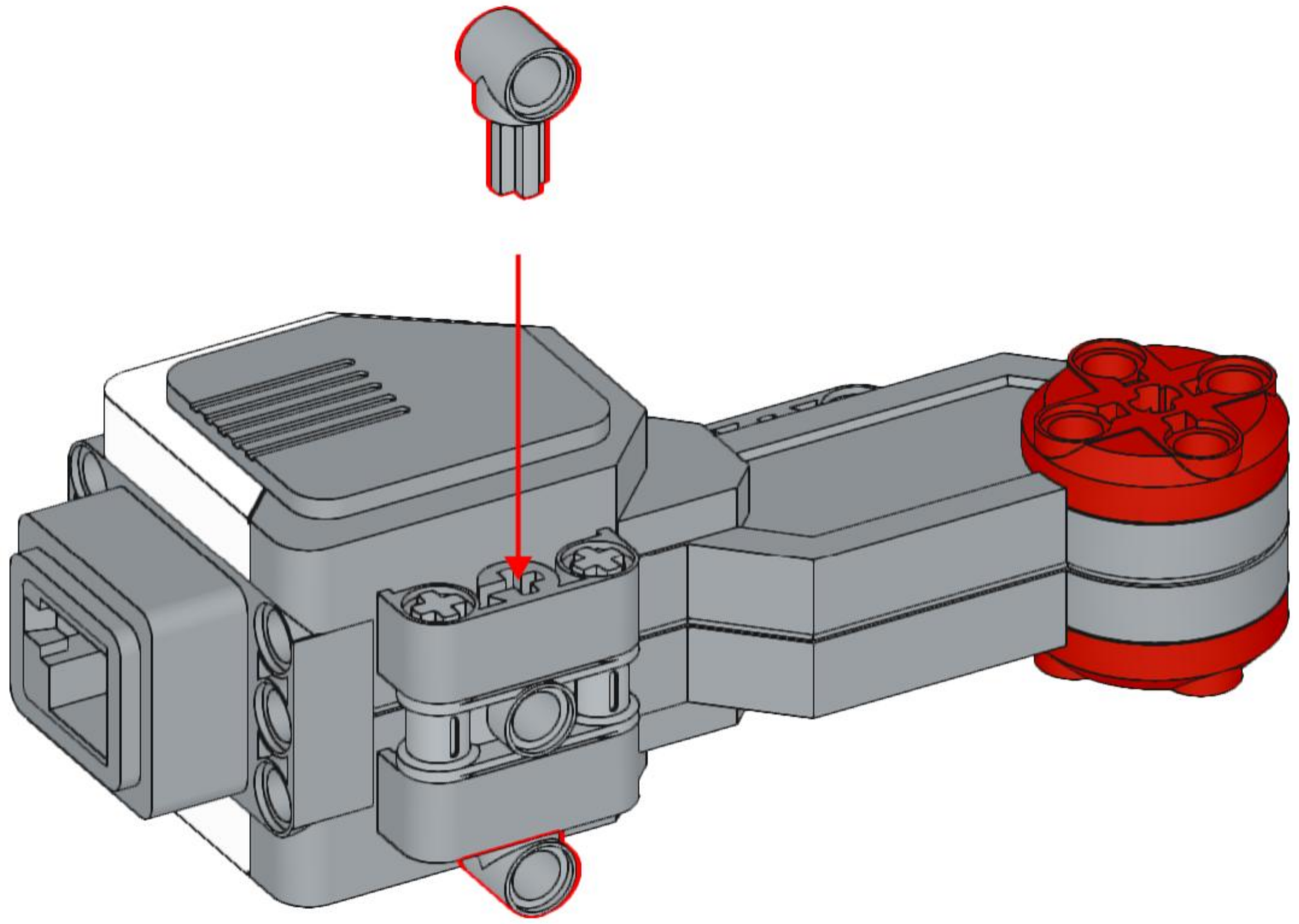

# 32

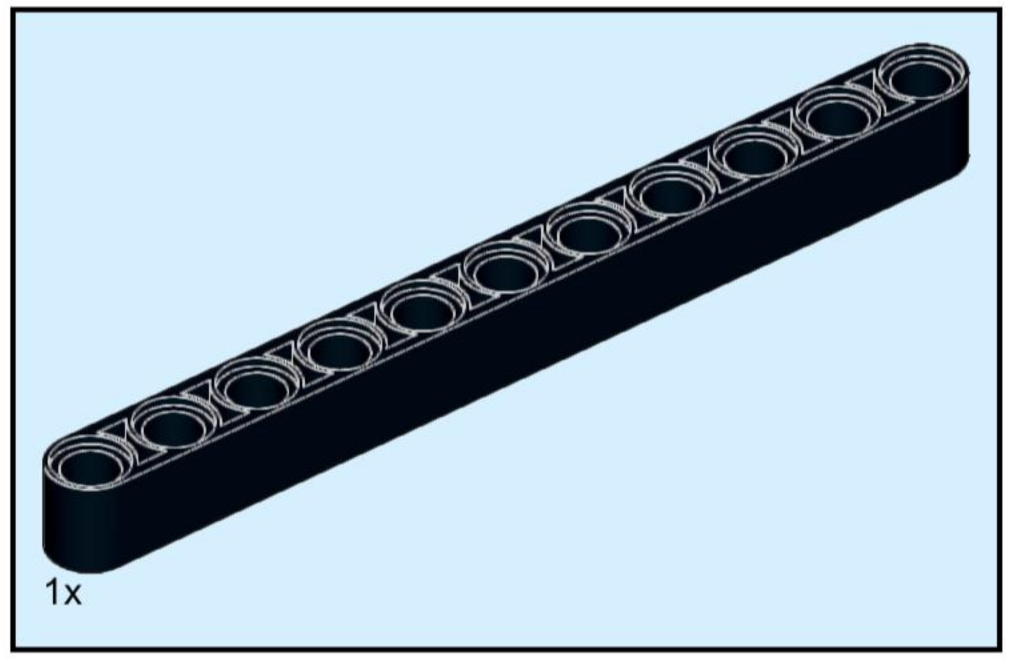


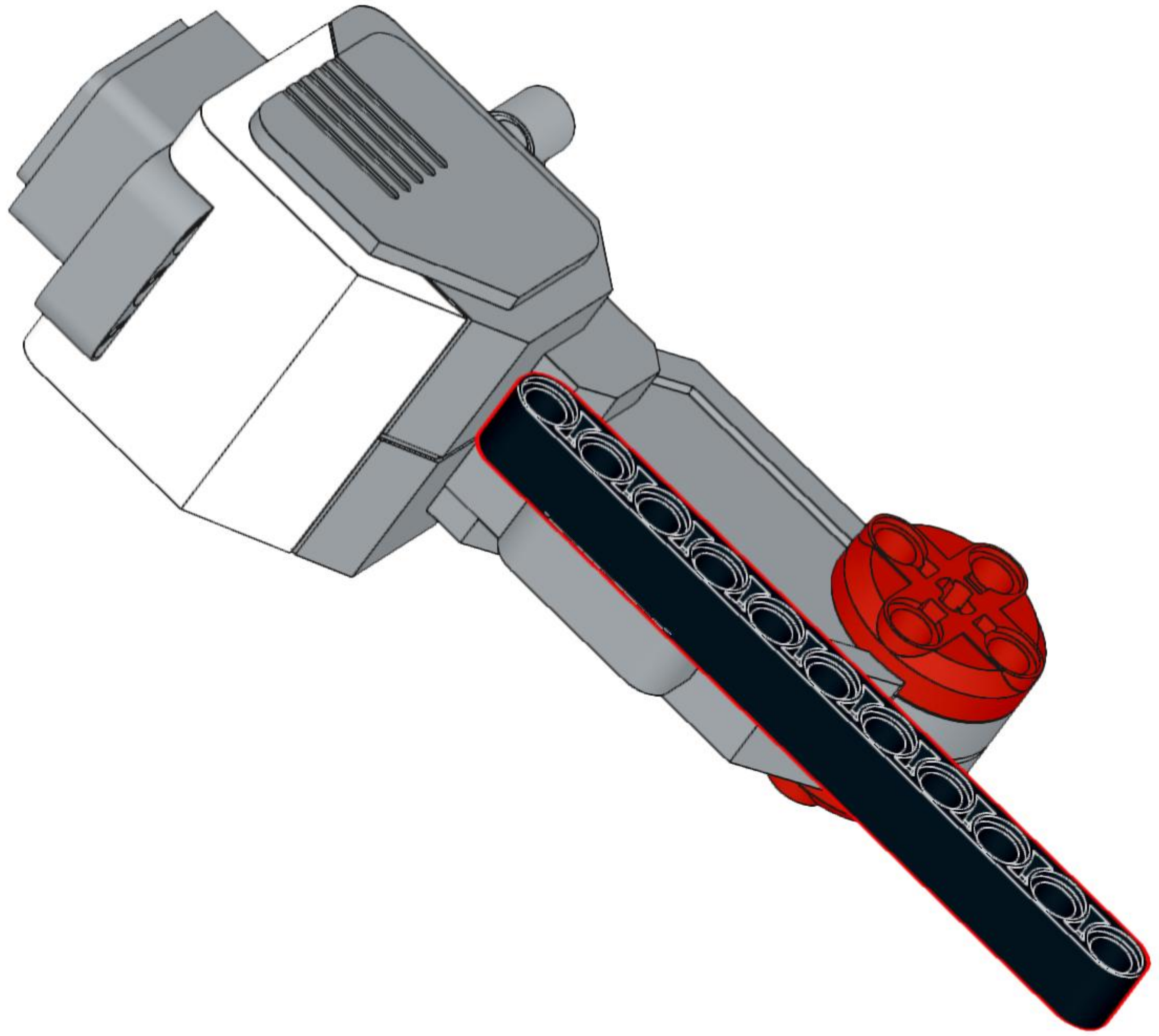

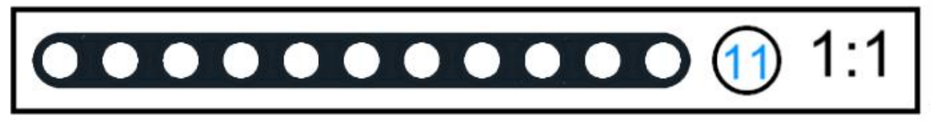


# 33

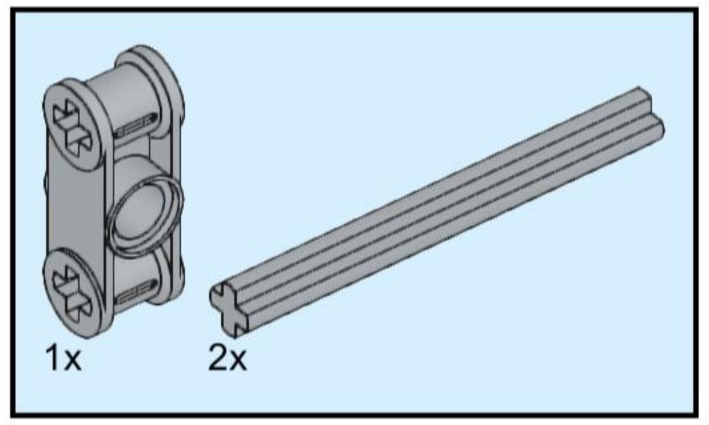


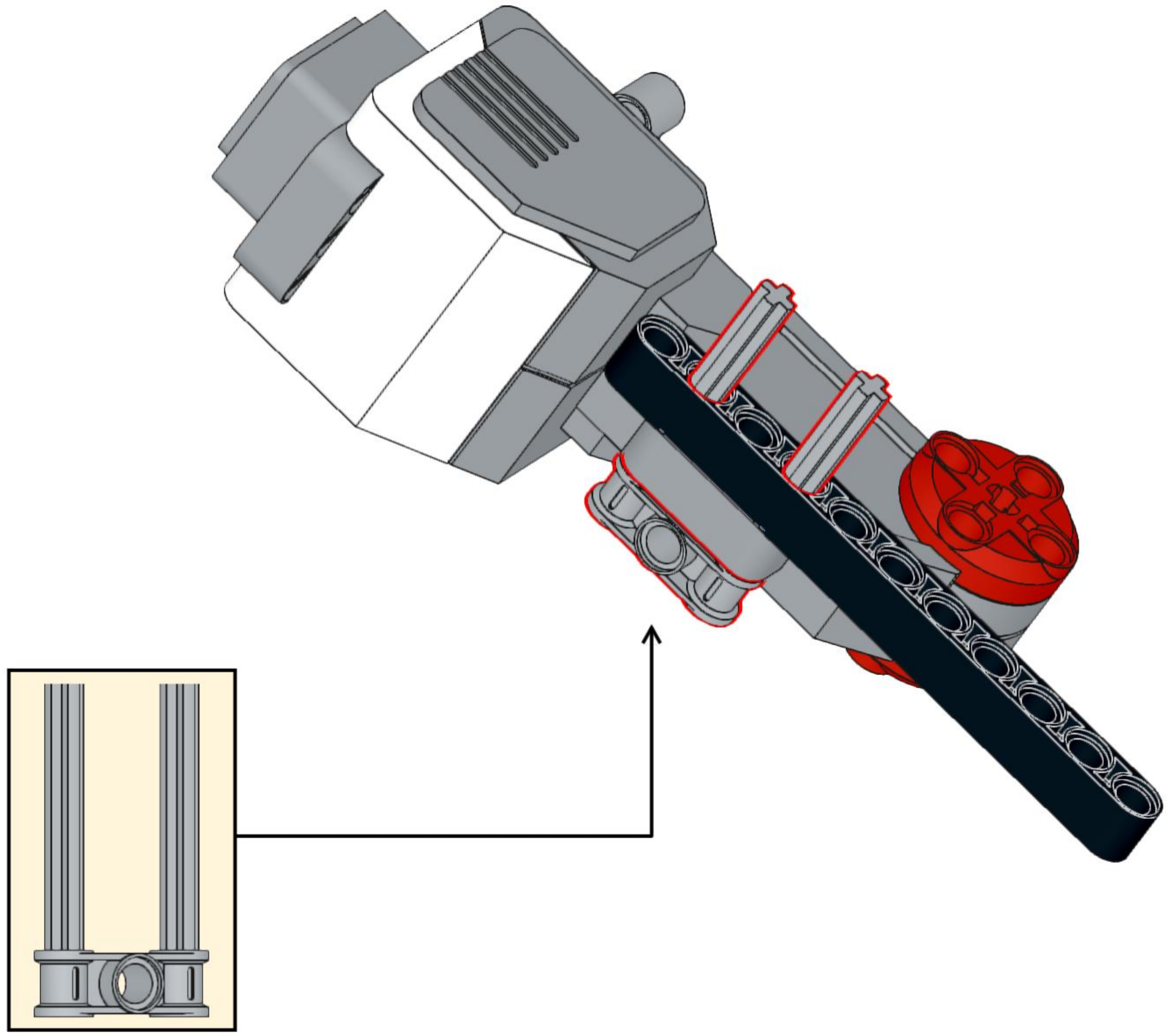

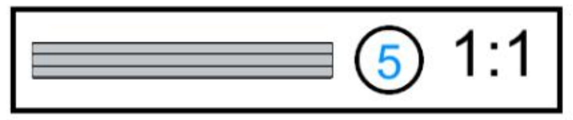

# 34

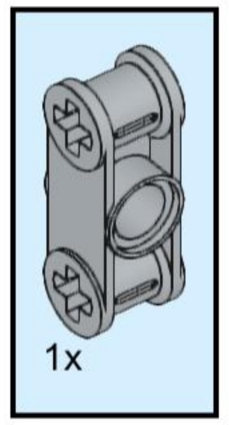


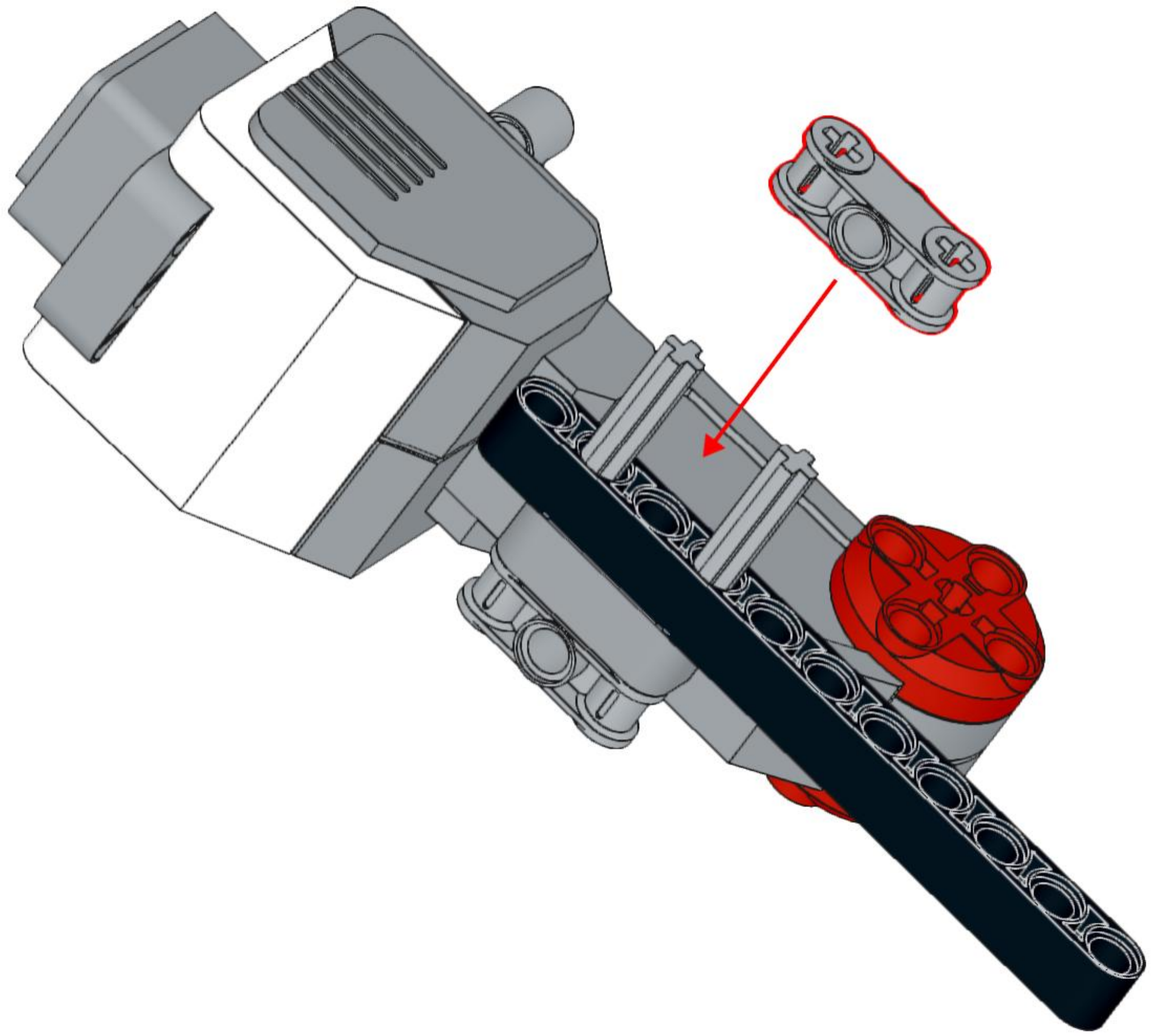

# 35

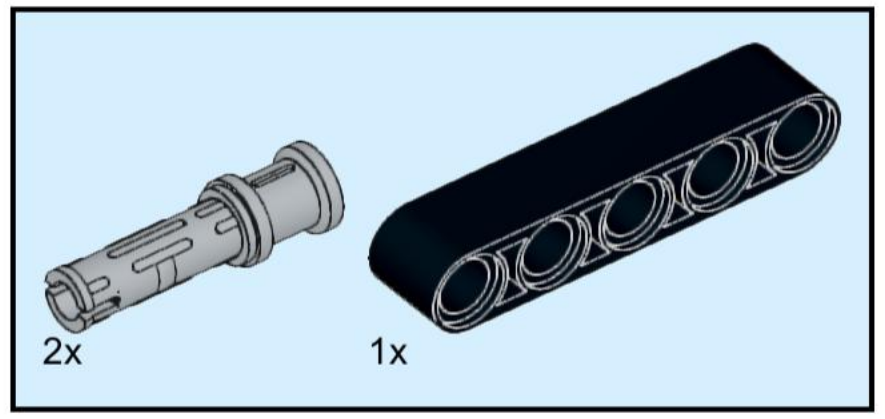


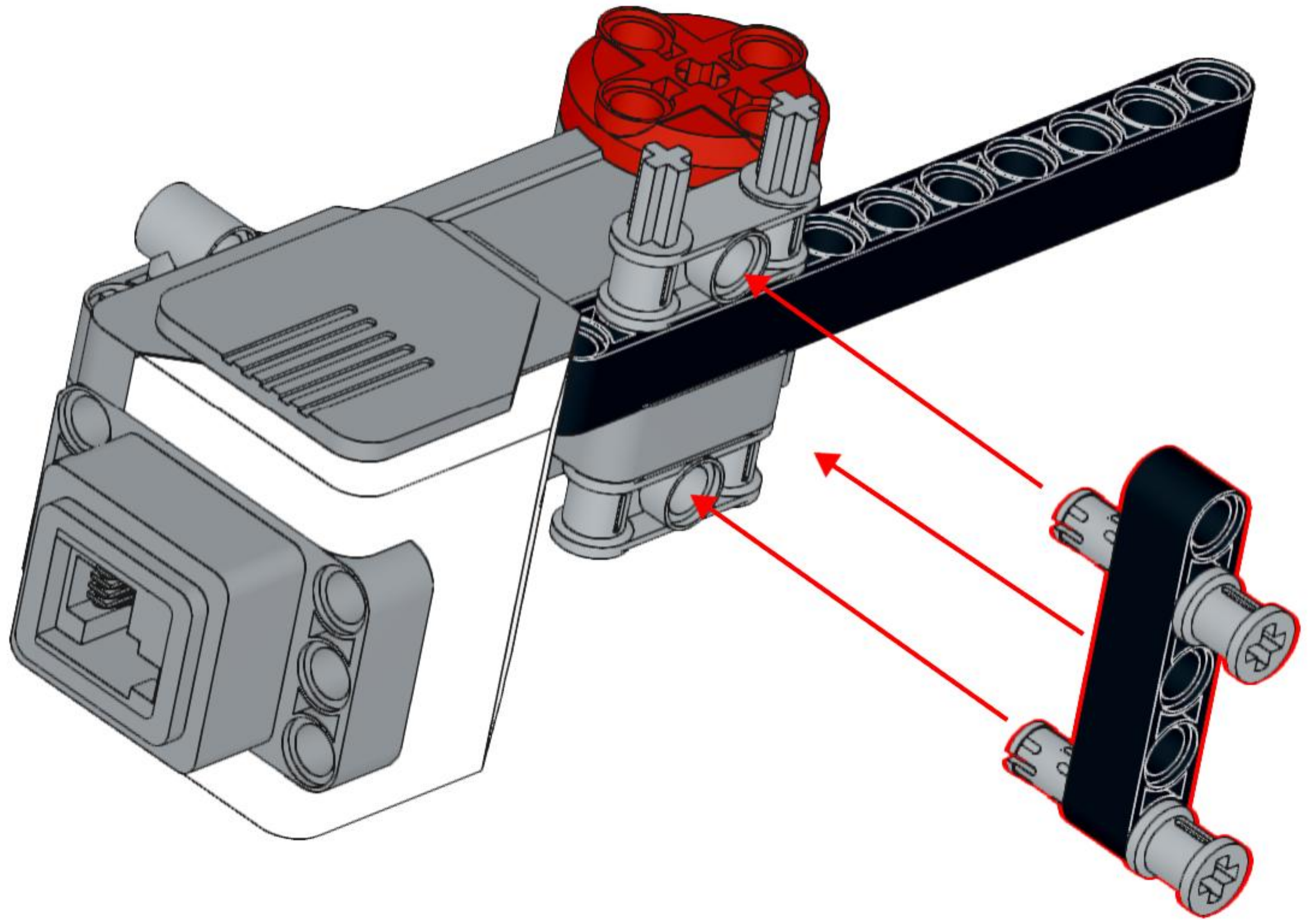

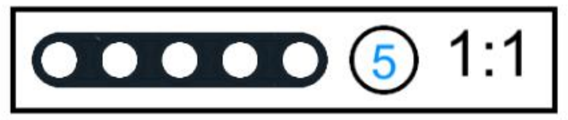

## 36

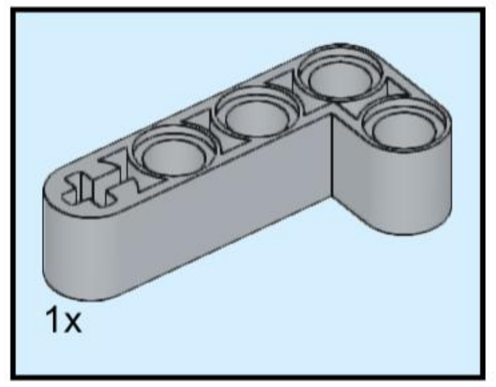


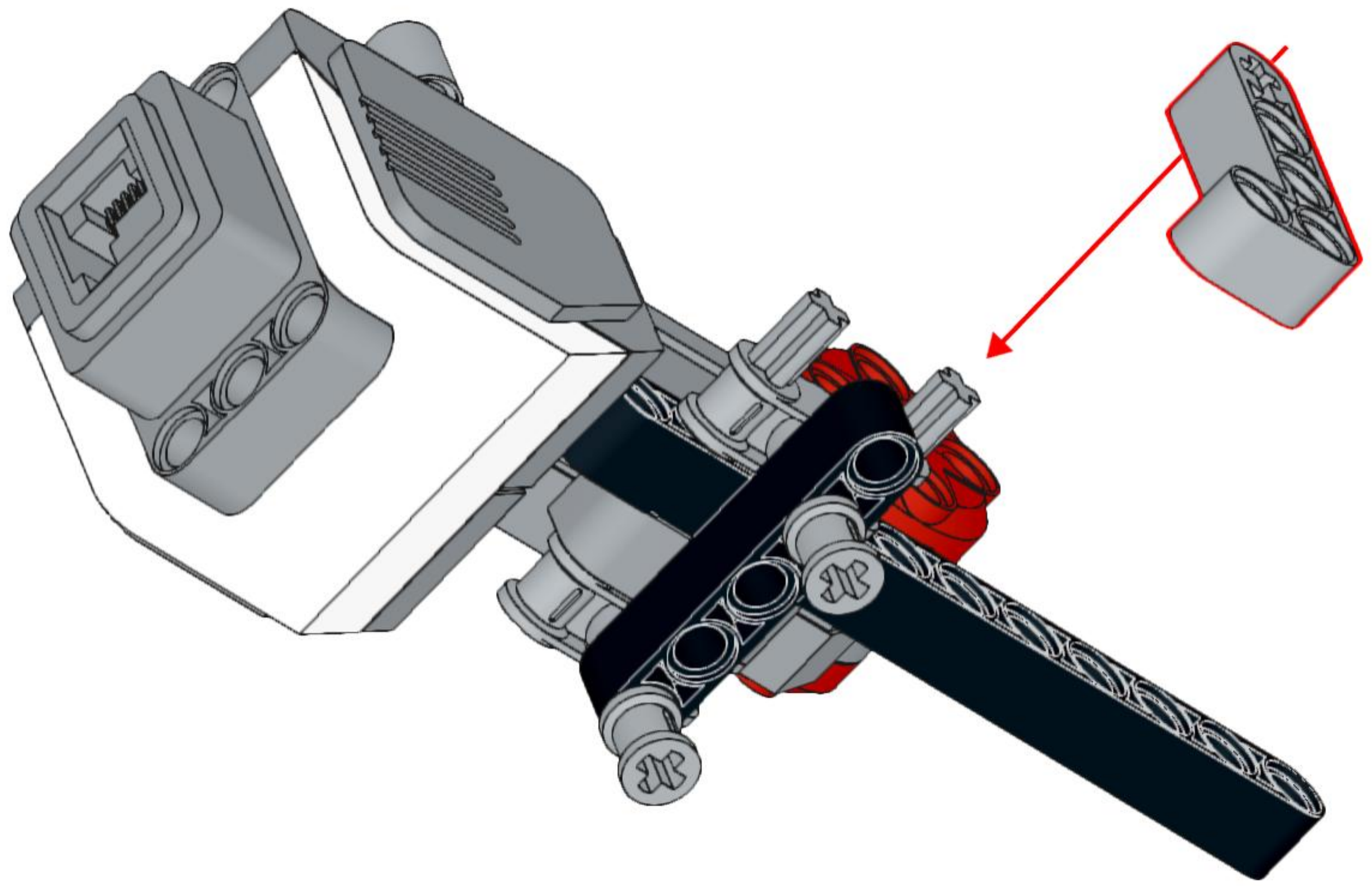

## 37

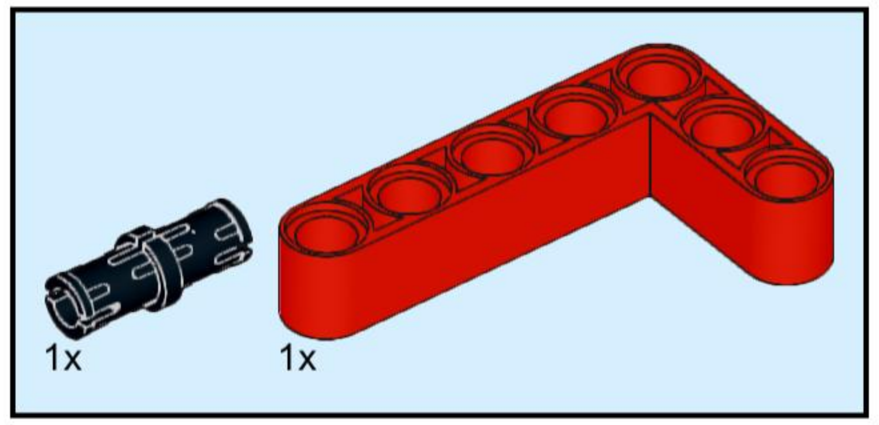


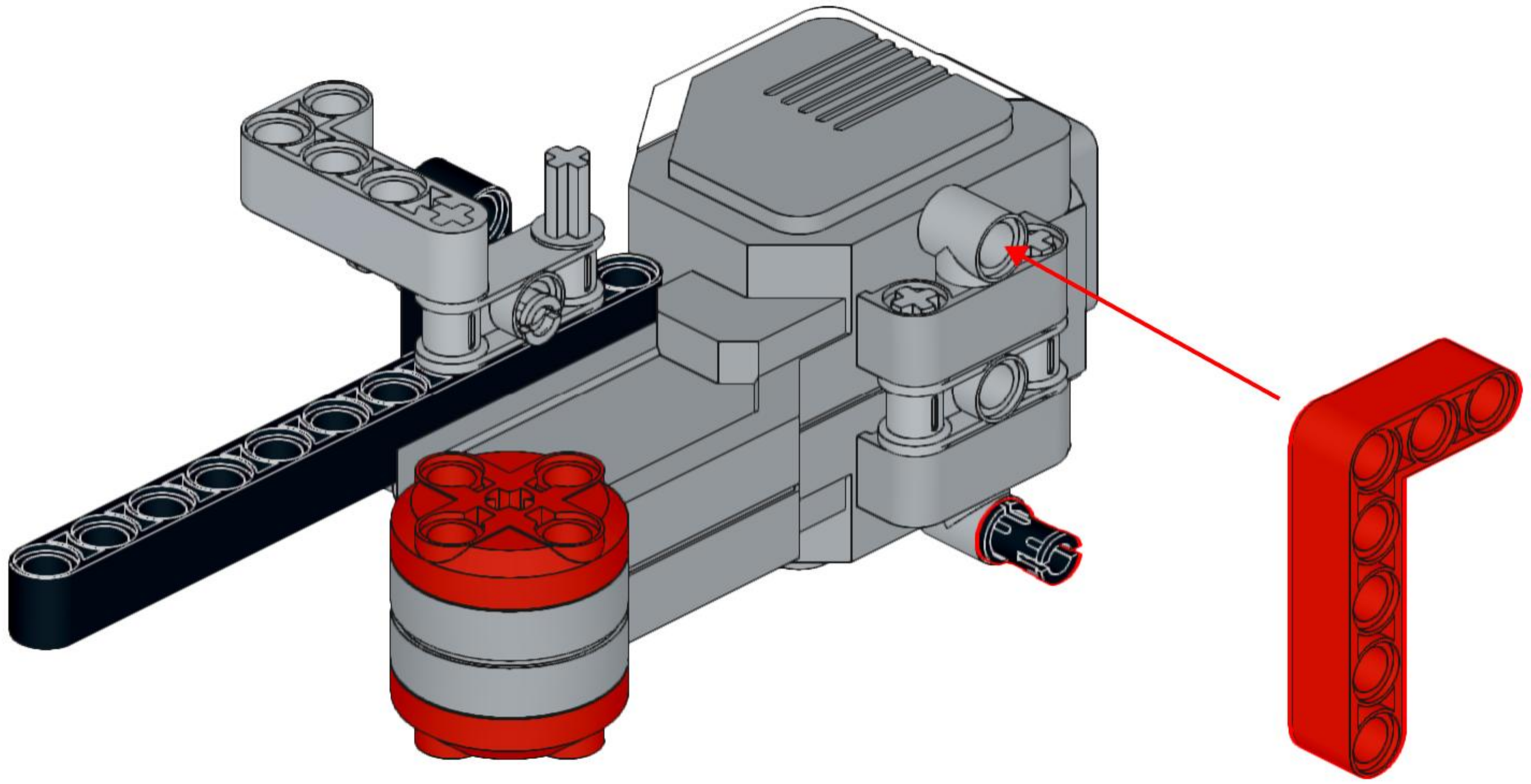

## 38

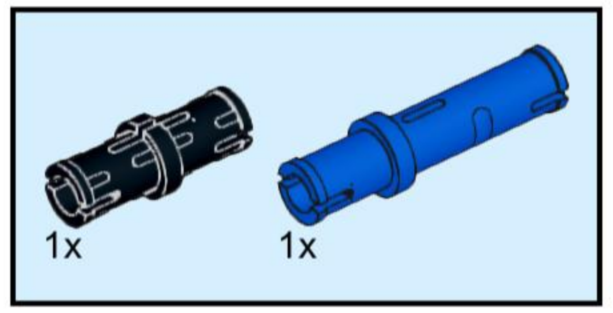


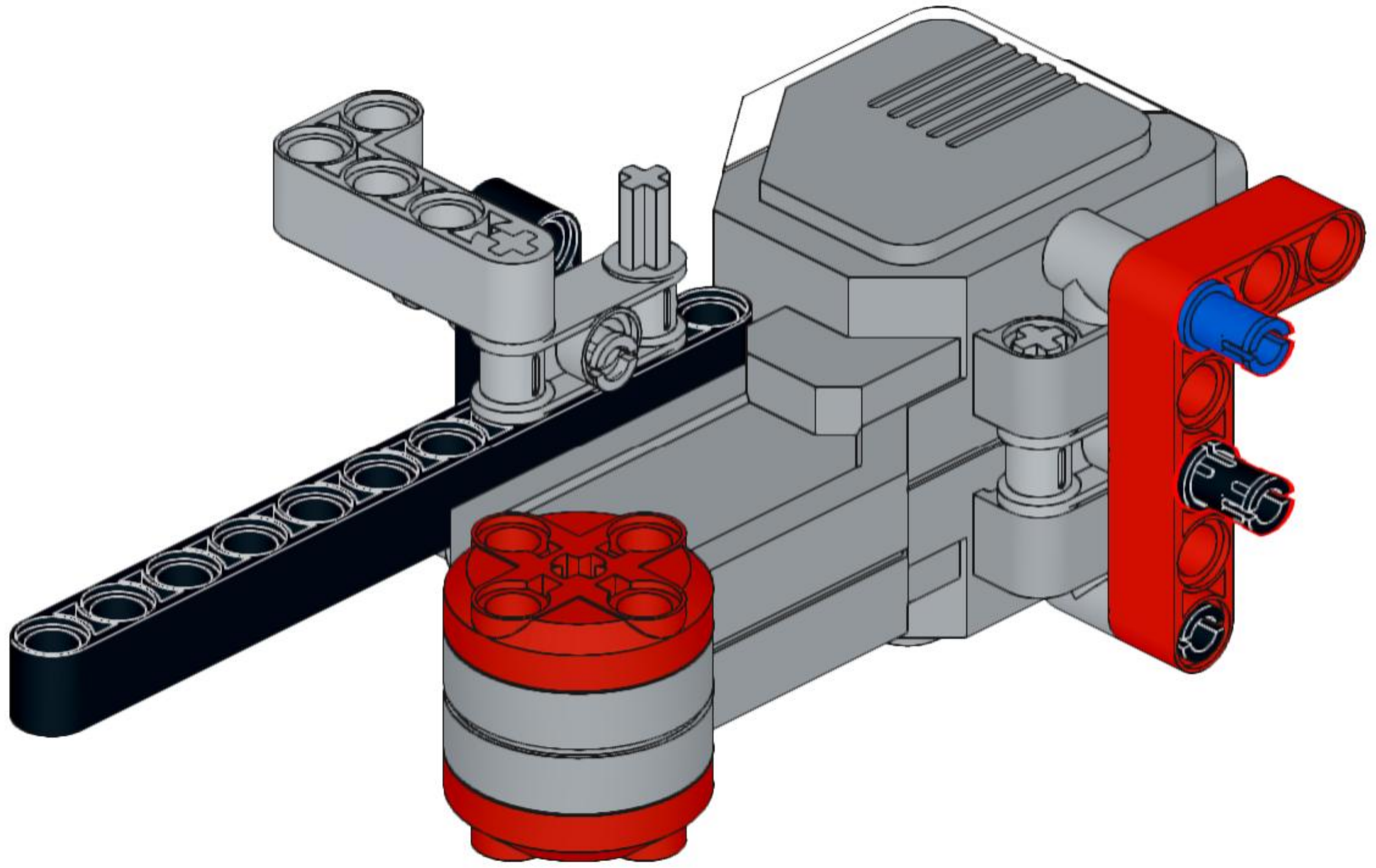

## 39

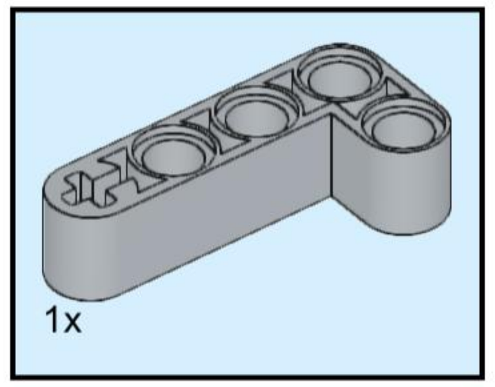


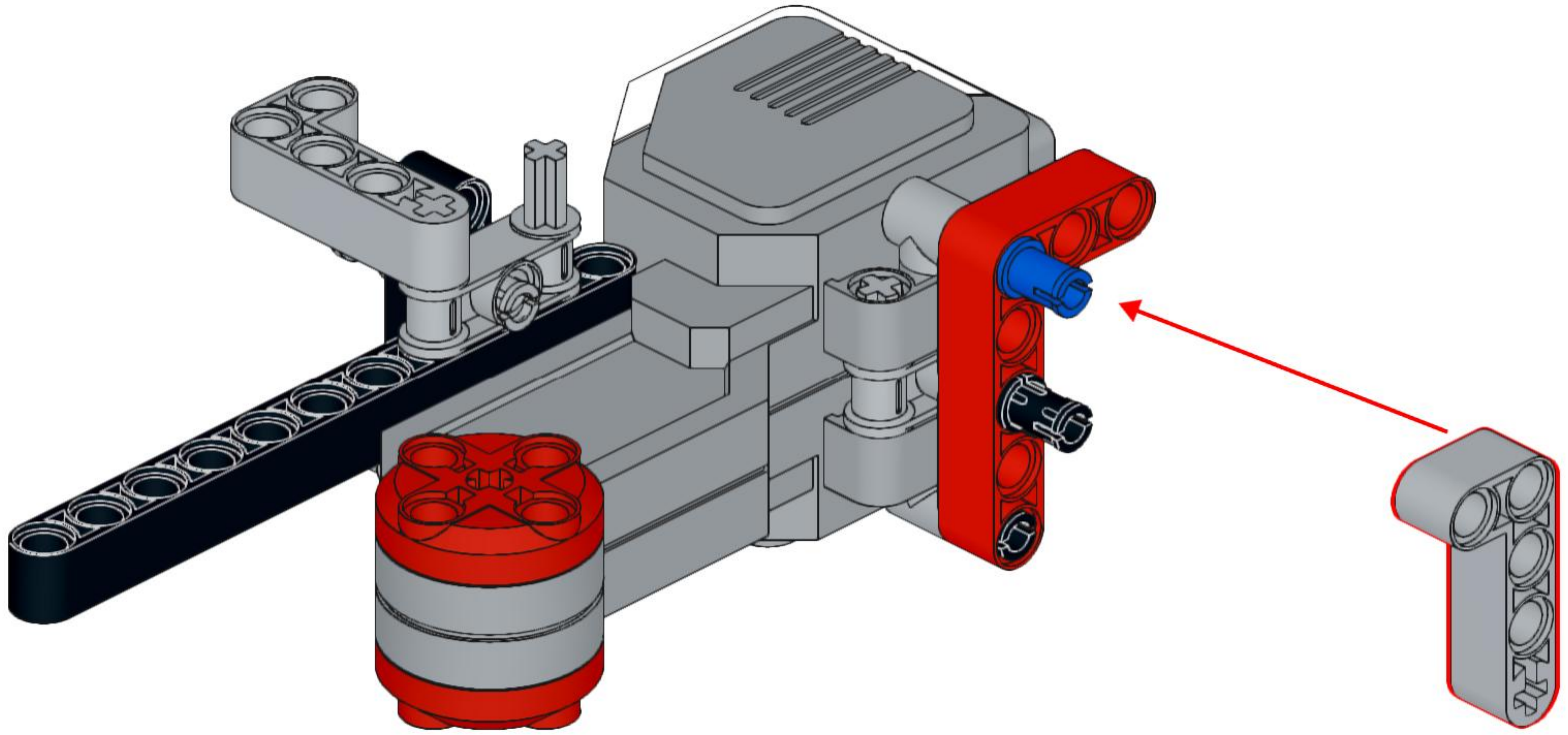

**40**

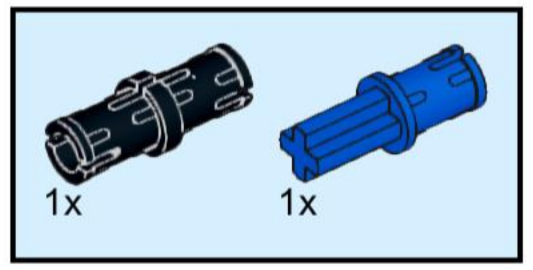


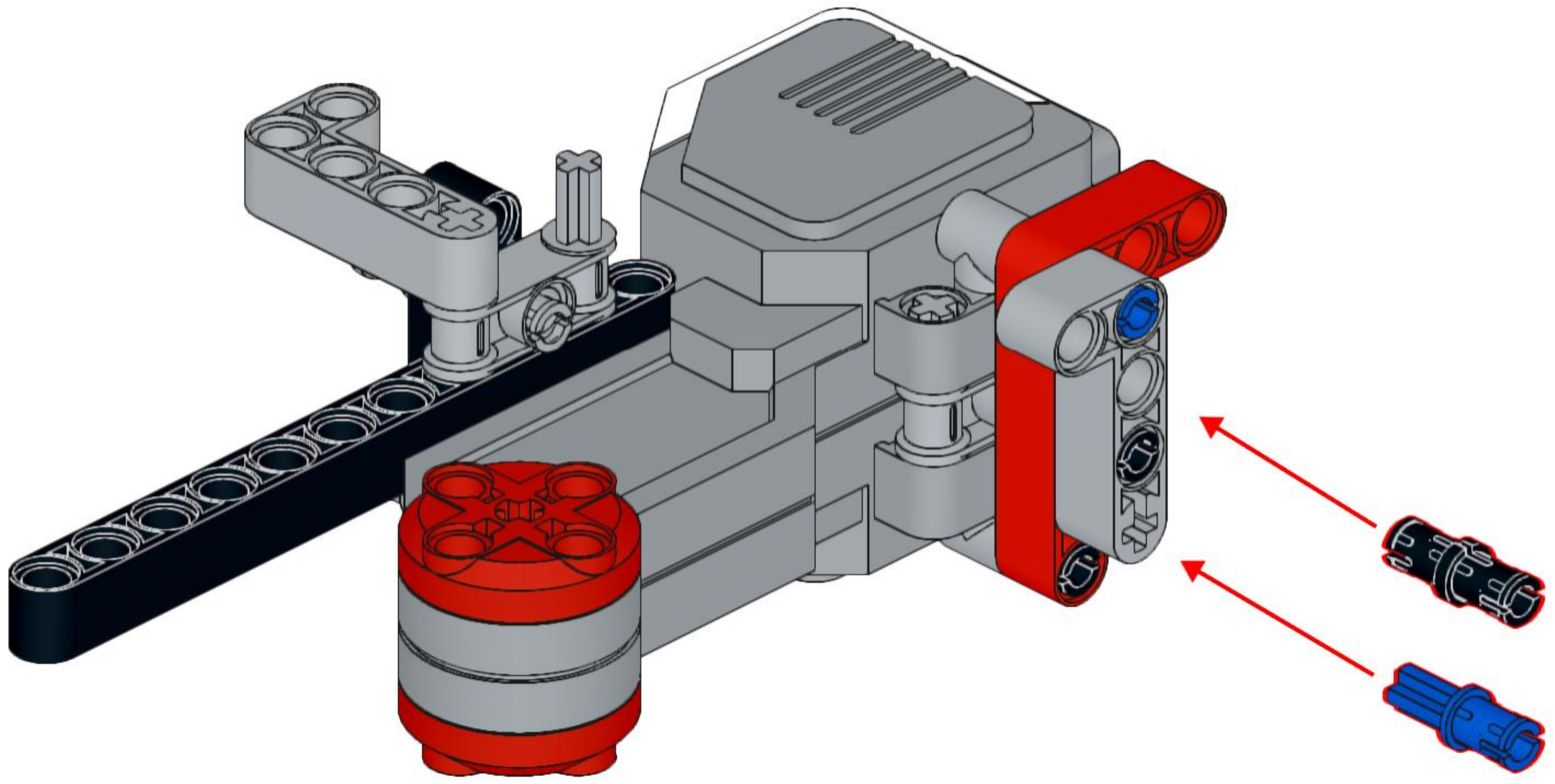

**41**

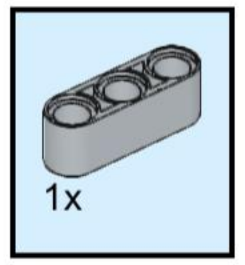


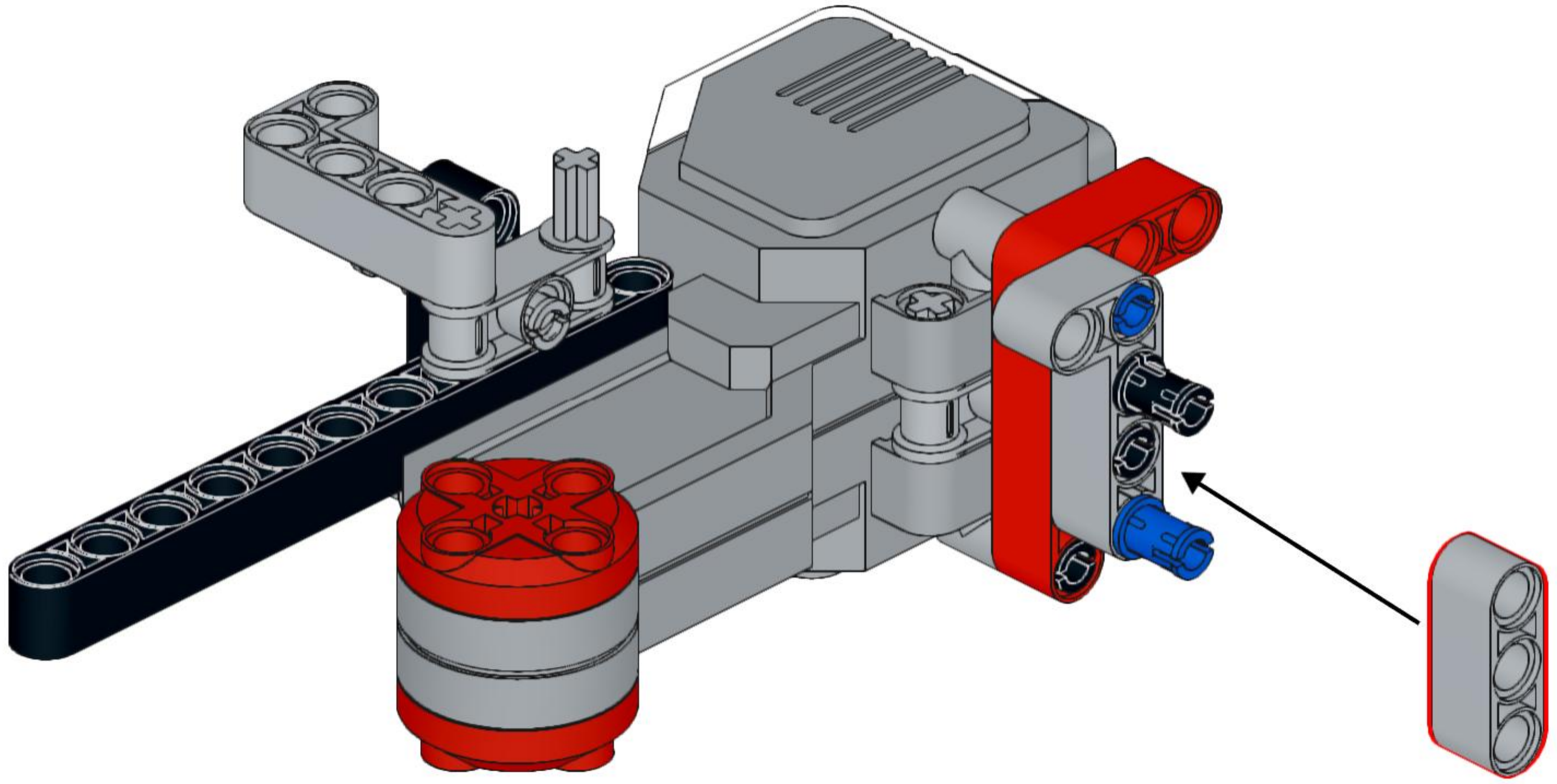

42

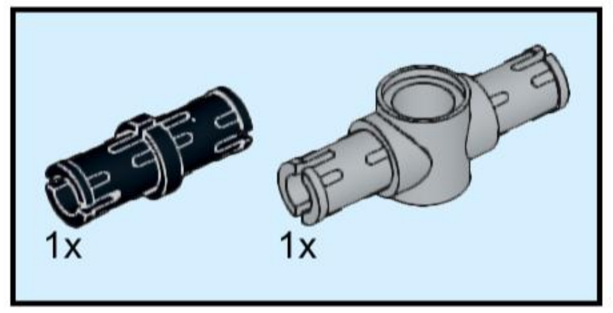


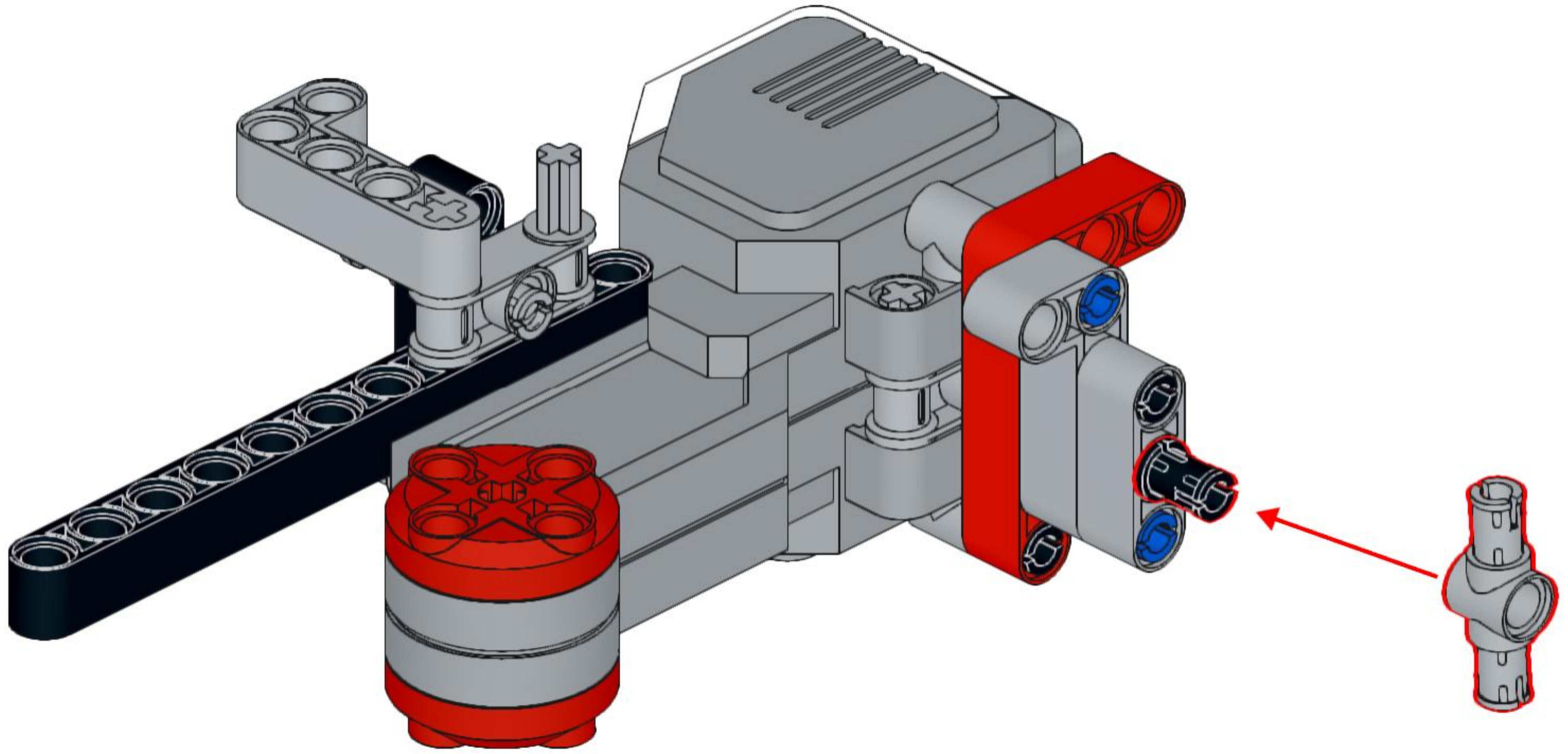

43

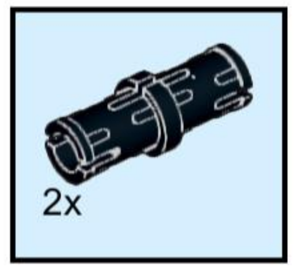


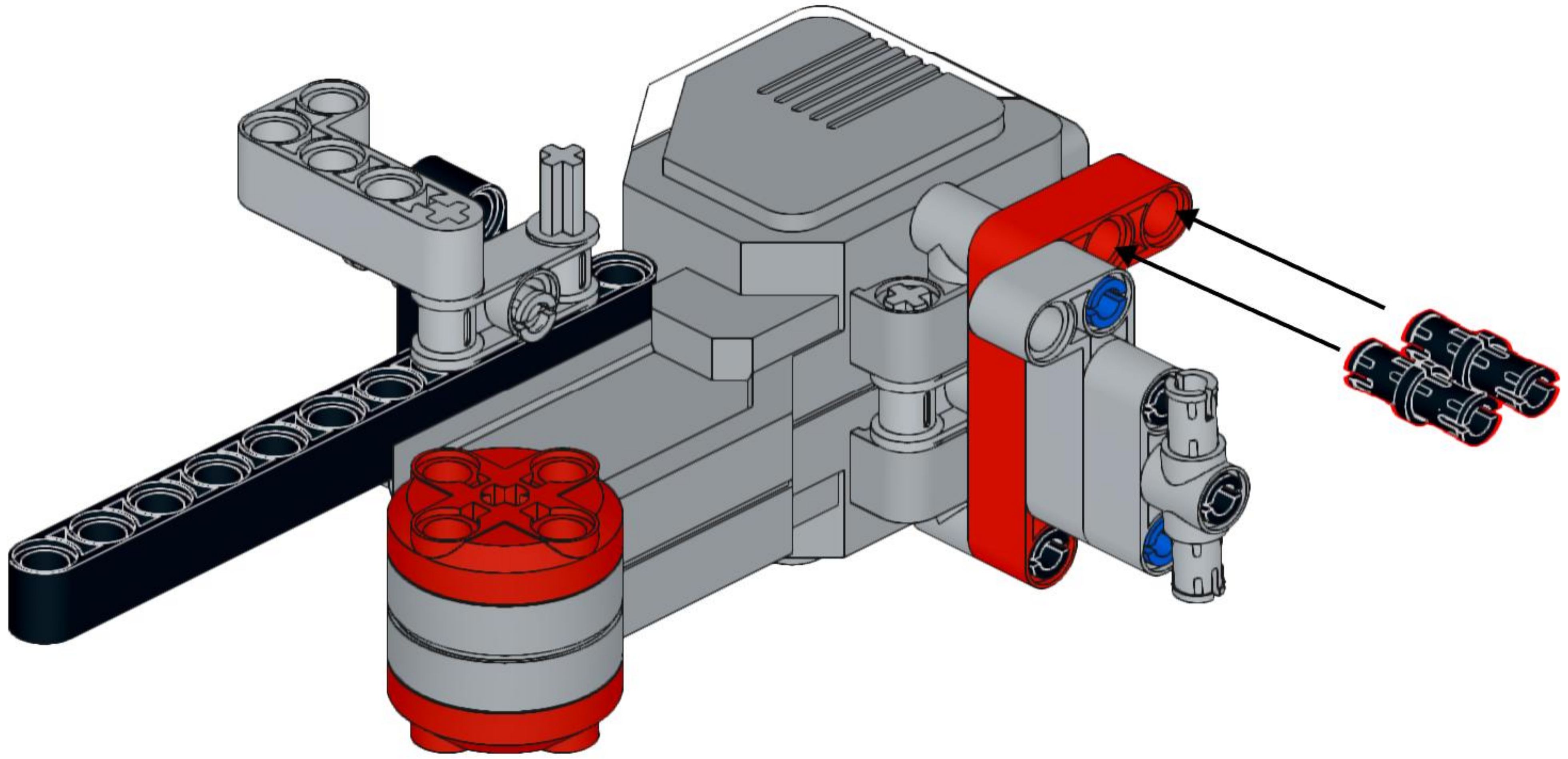

## 44

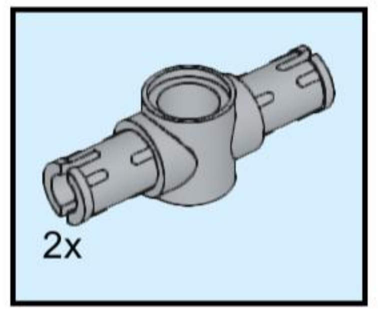


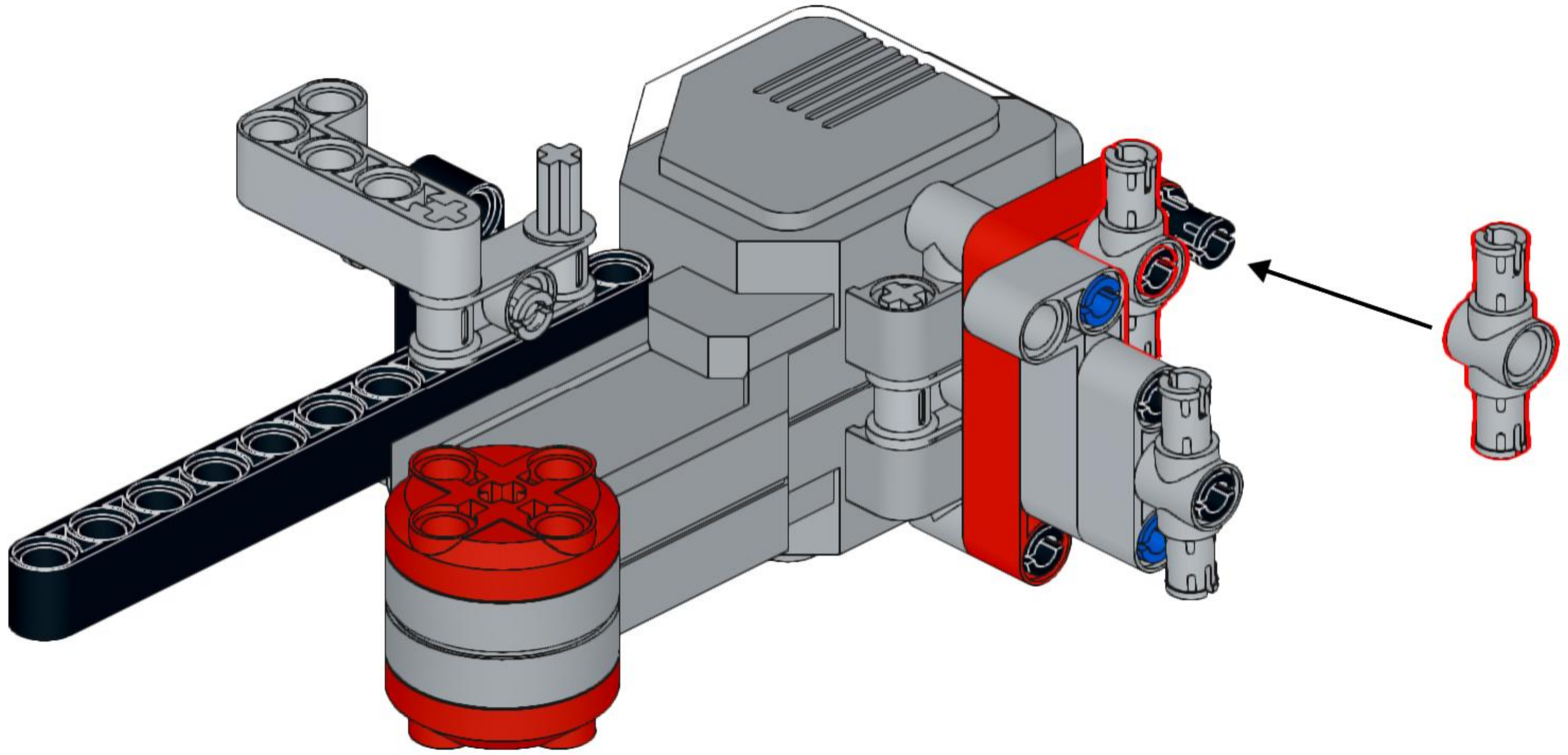

## 45

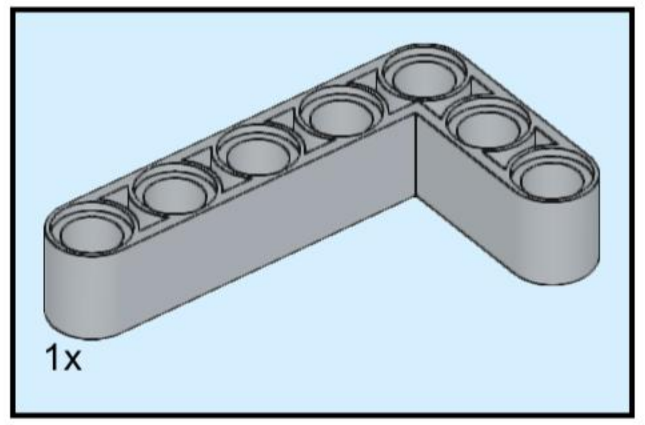


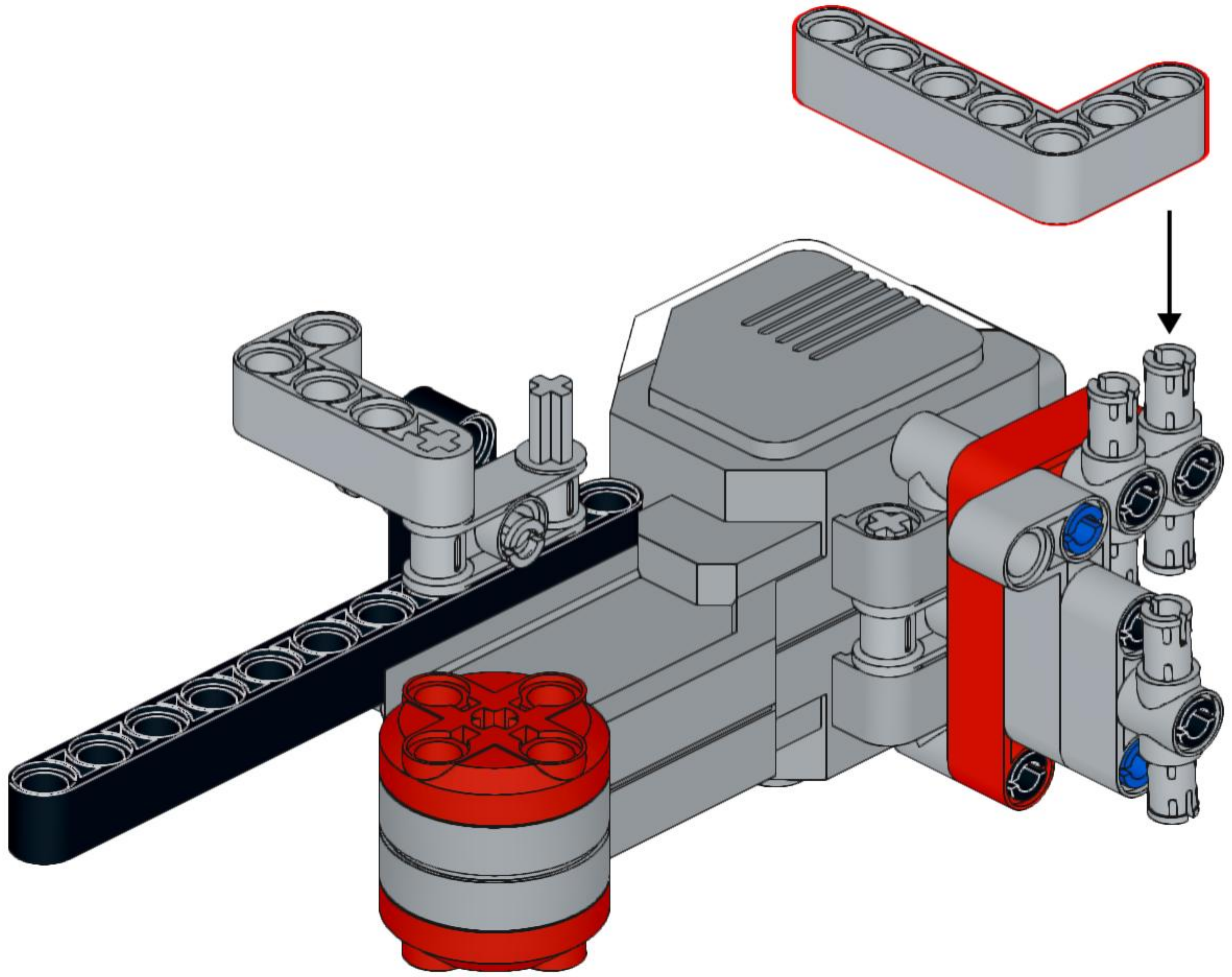

46

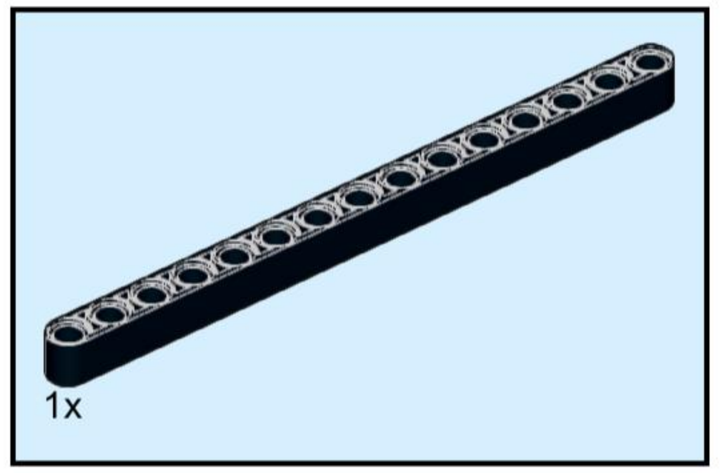


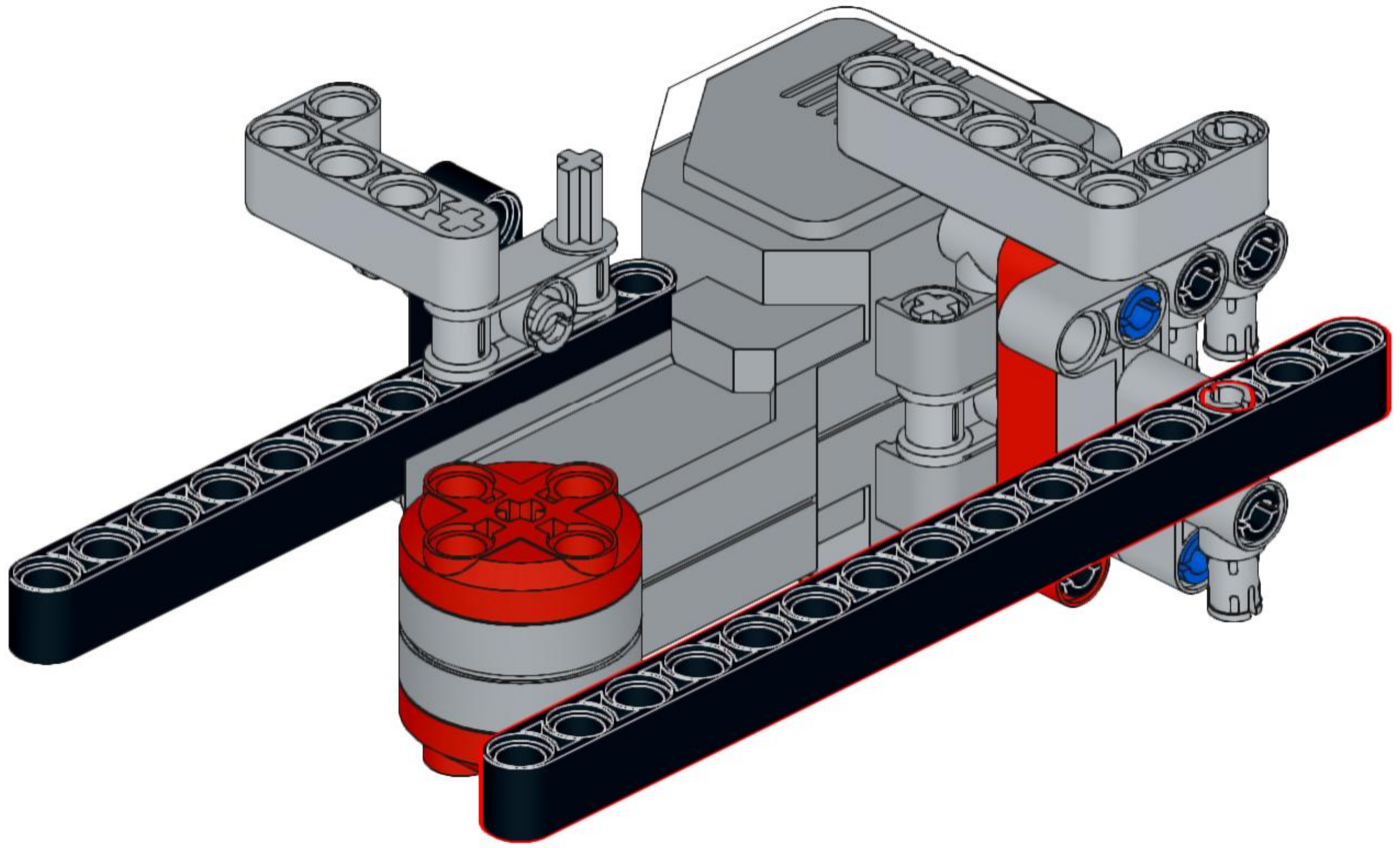

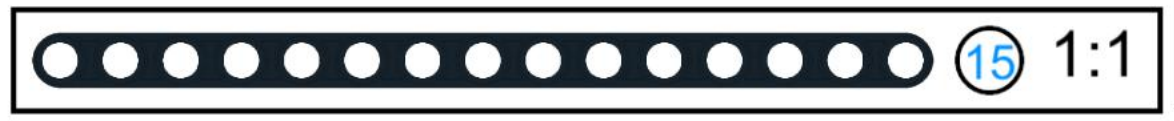


47

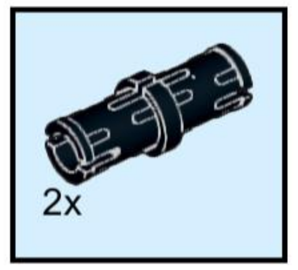


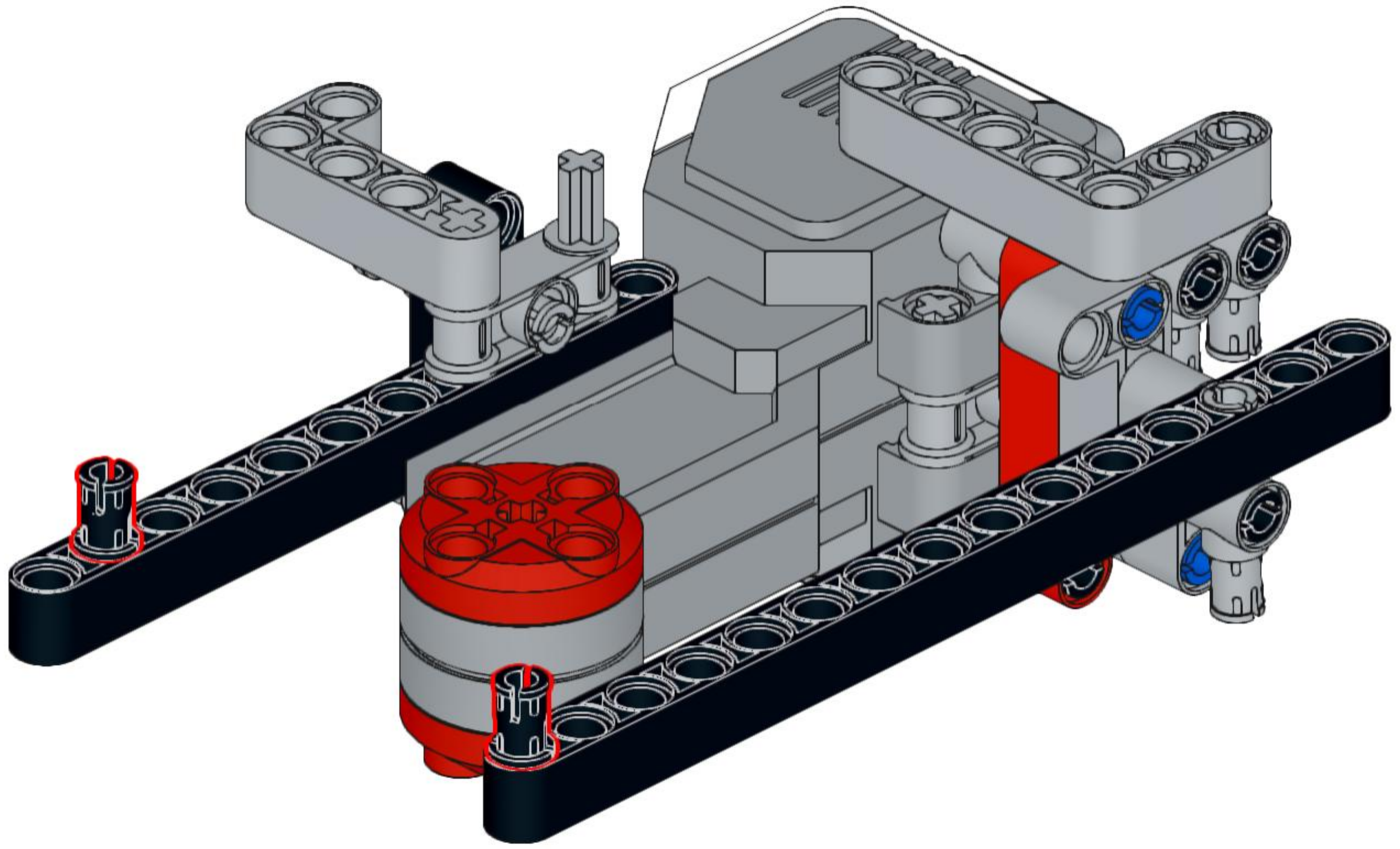

# 48

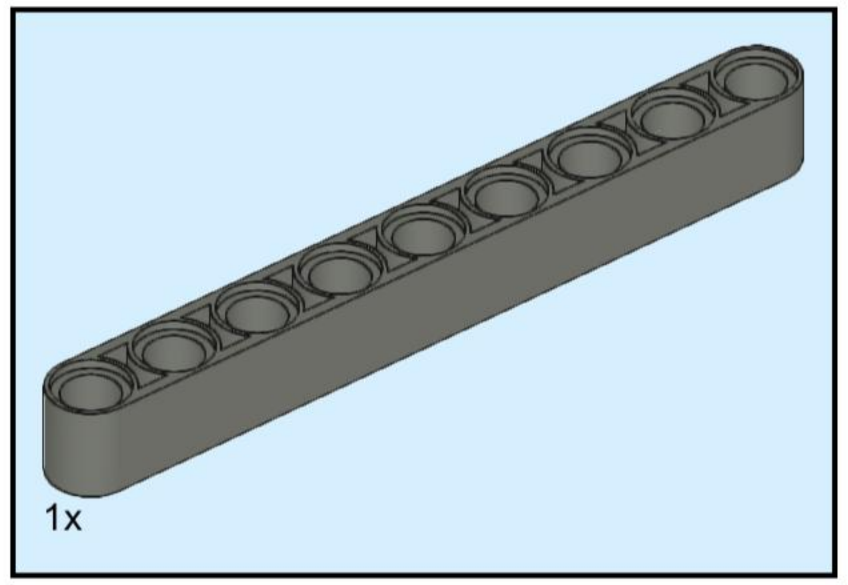


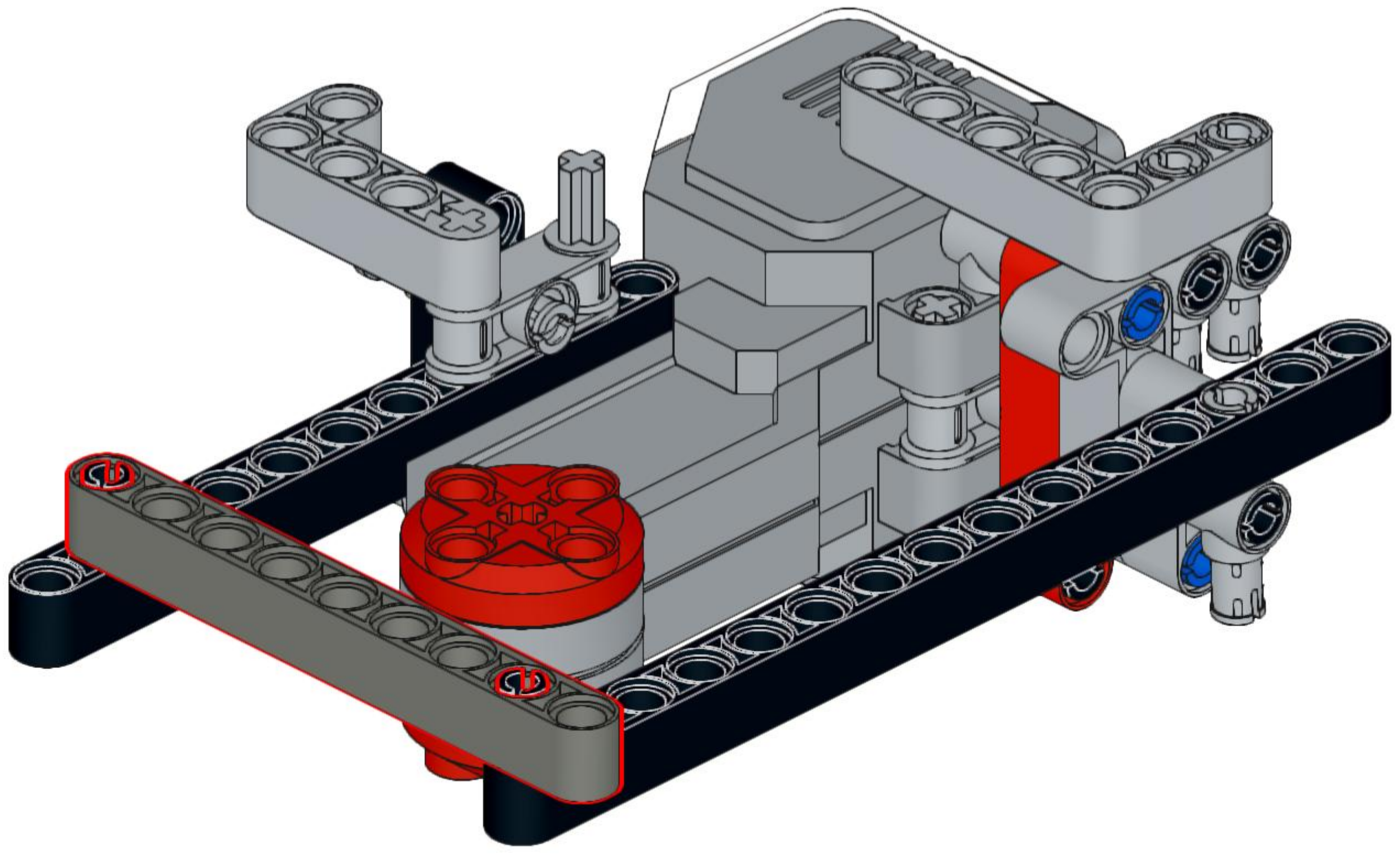

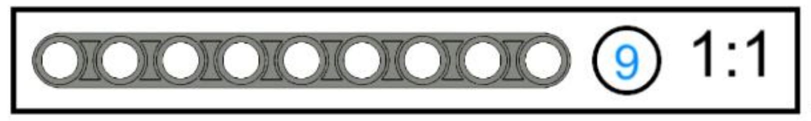


# 49

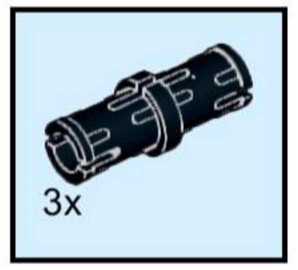


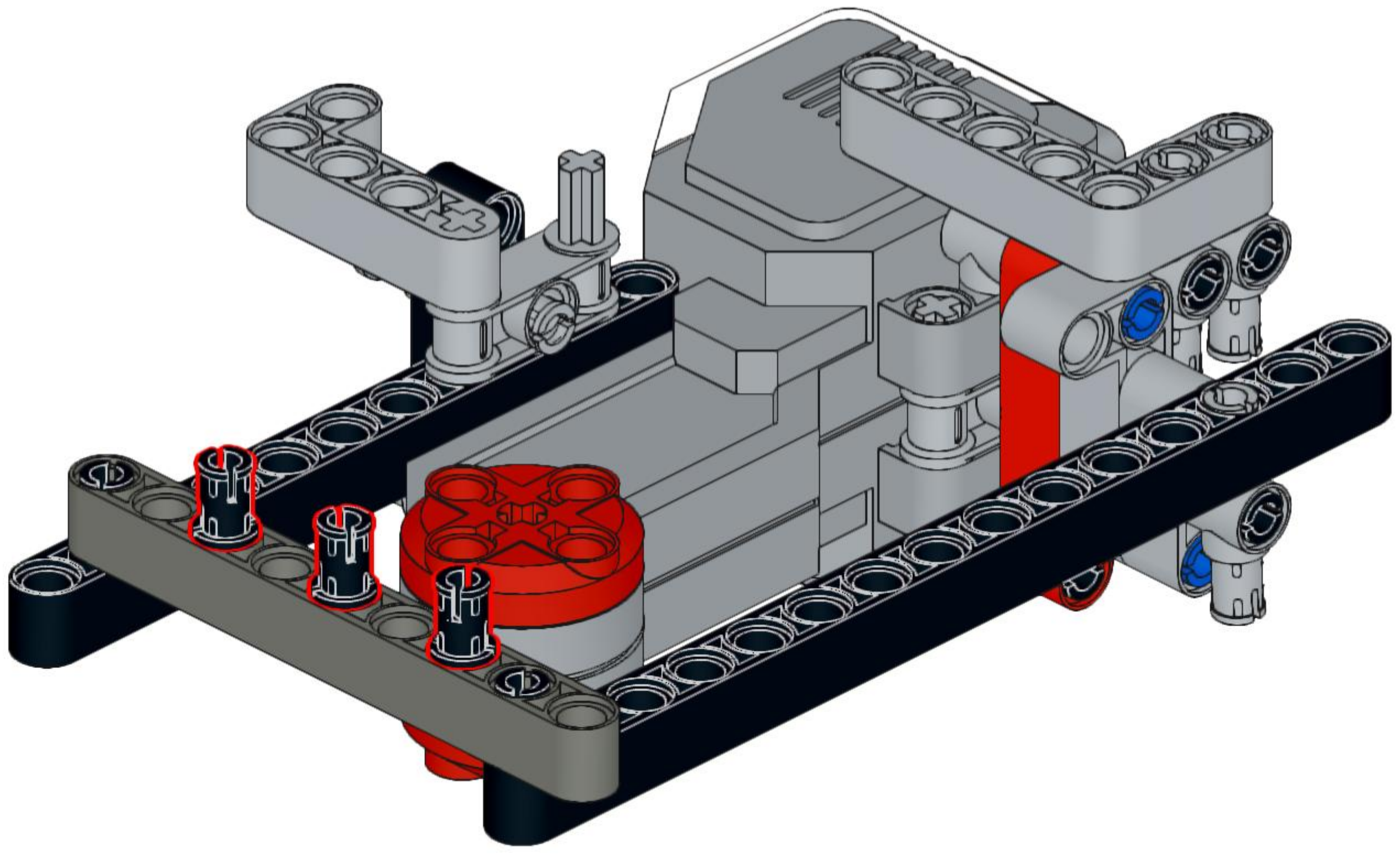

**50**

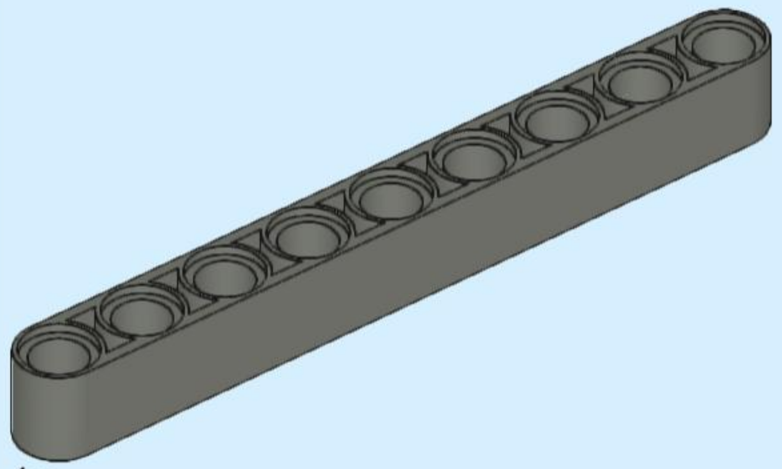

1x

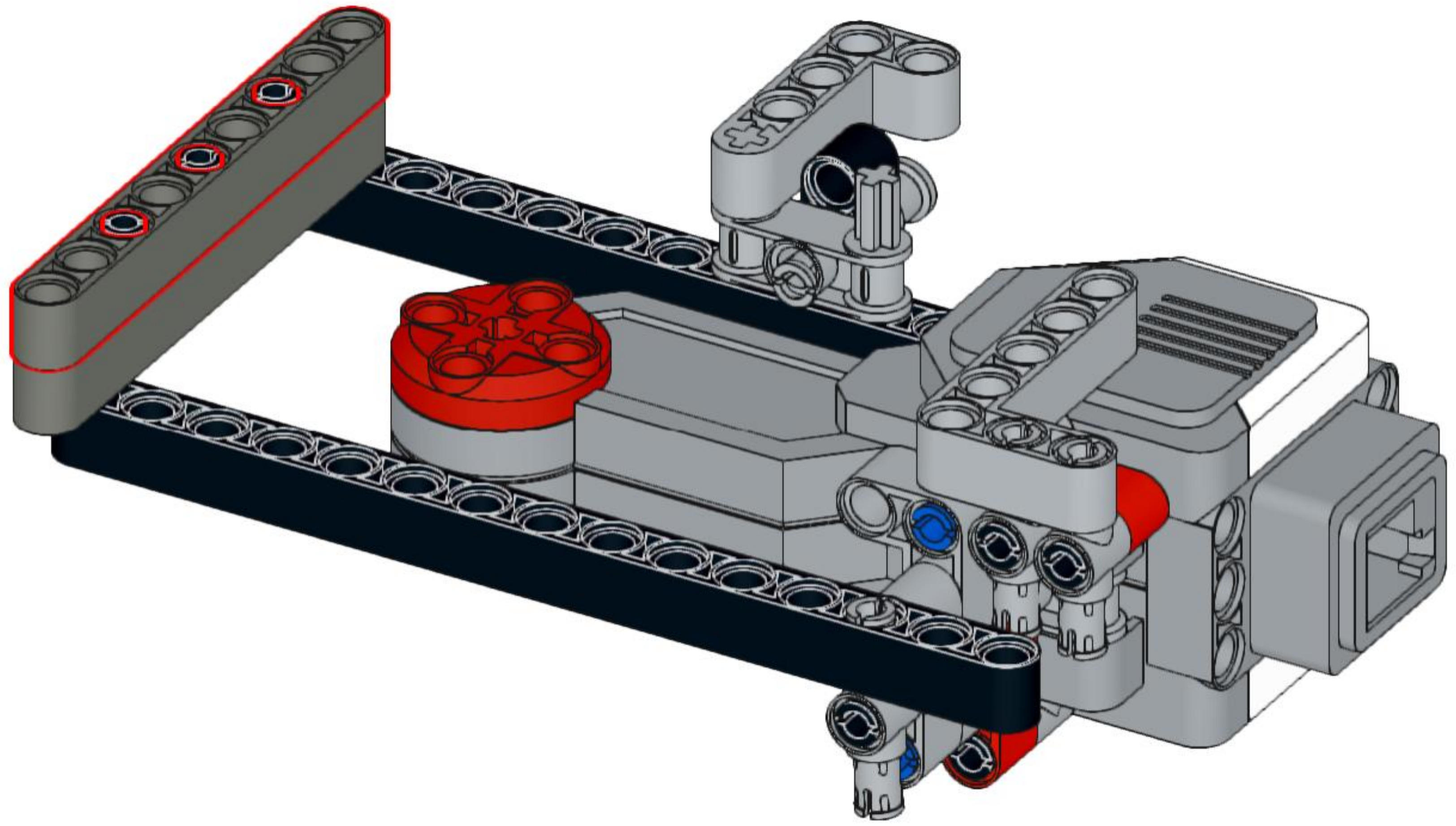

**51**

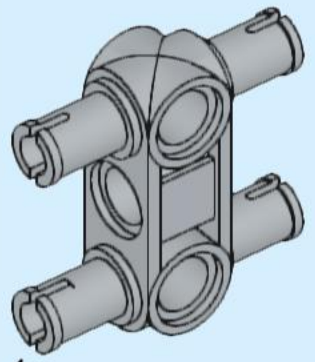

1x

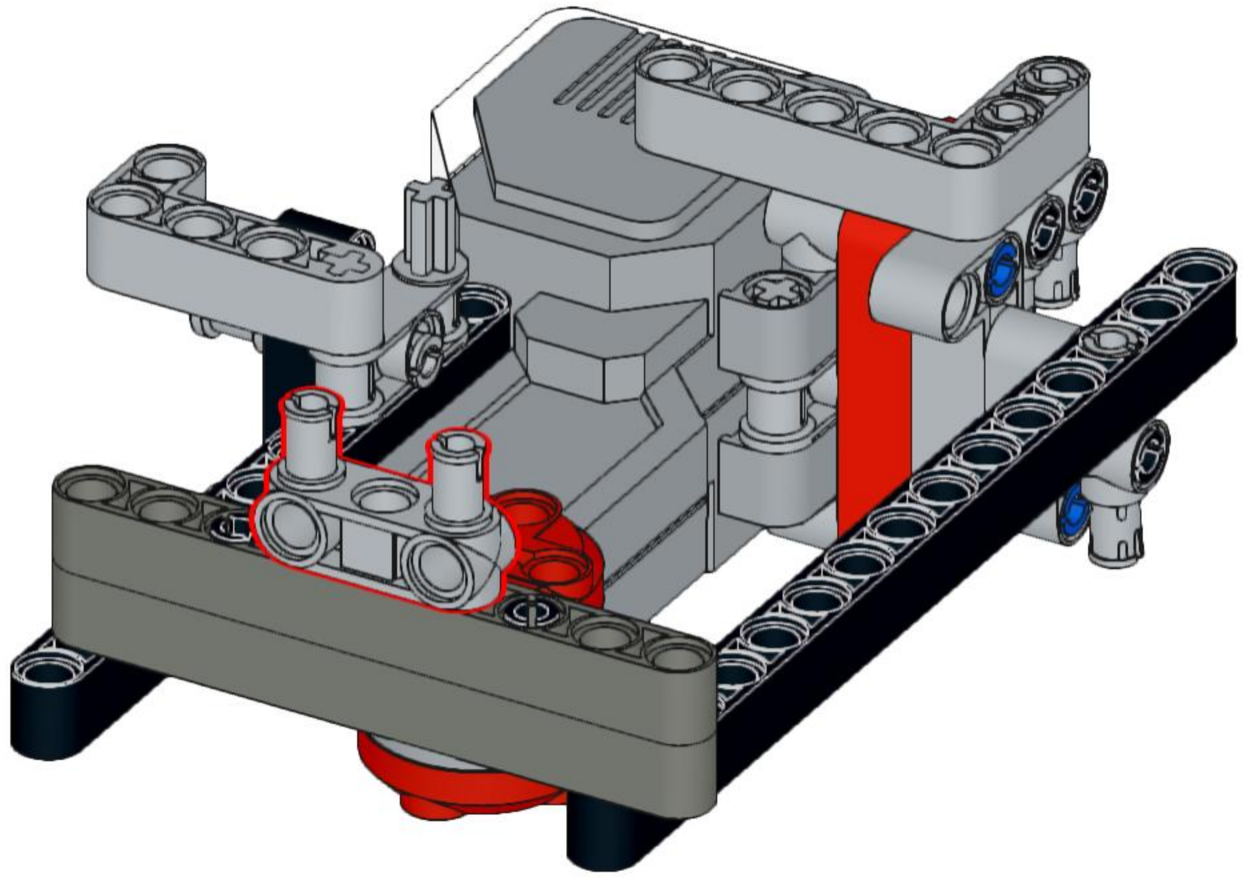

## 52

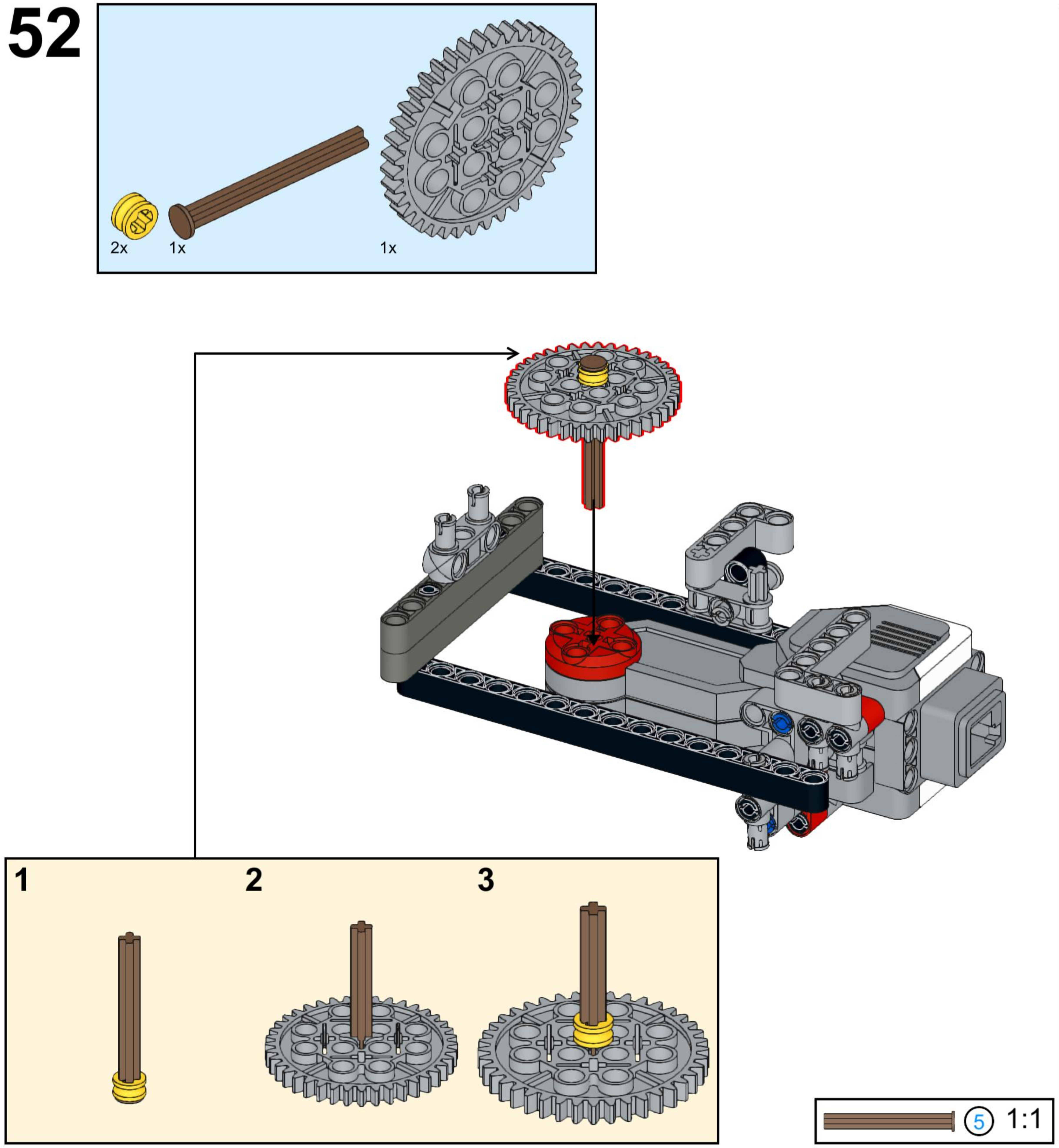


## 53

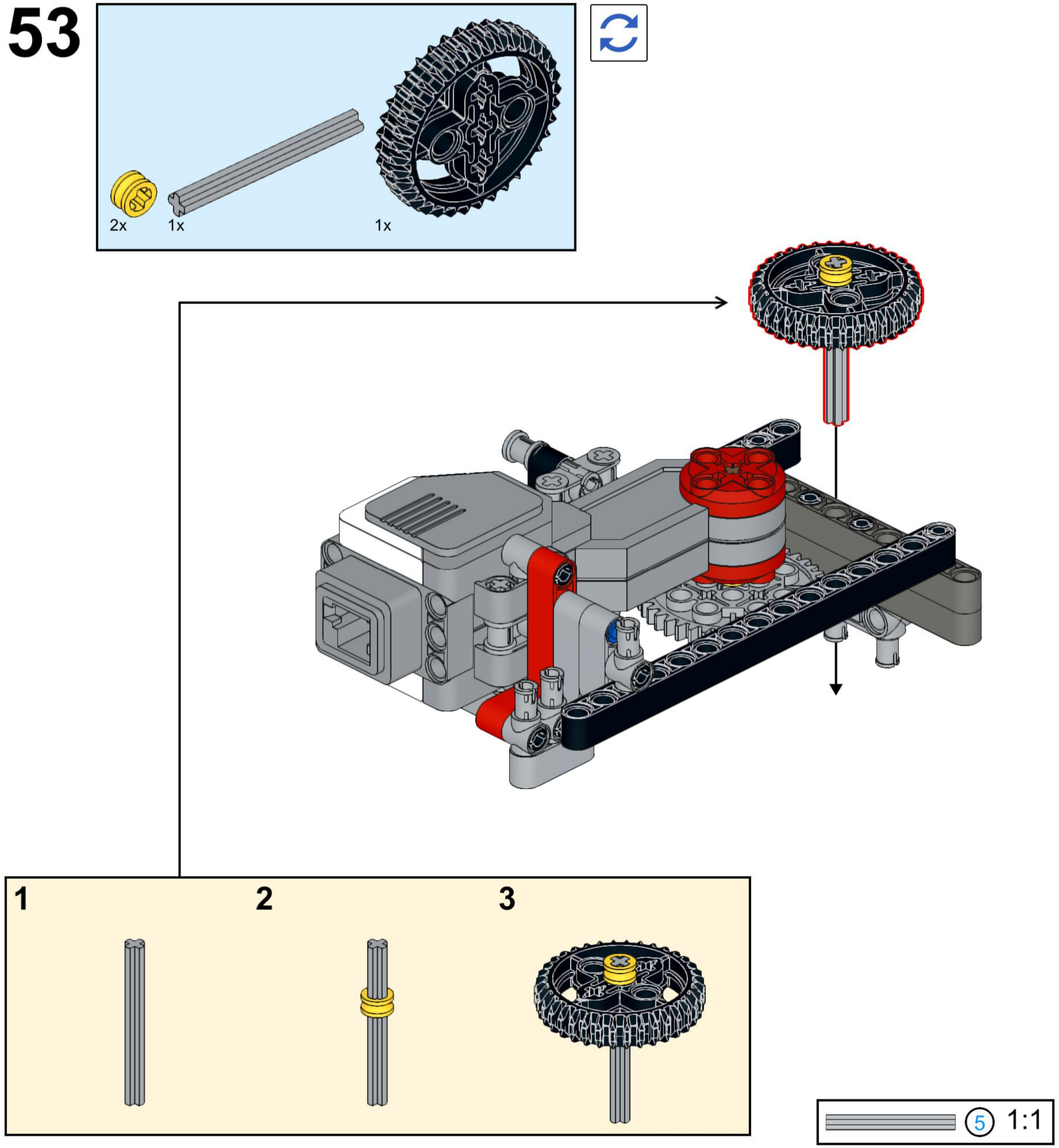

# 54

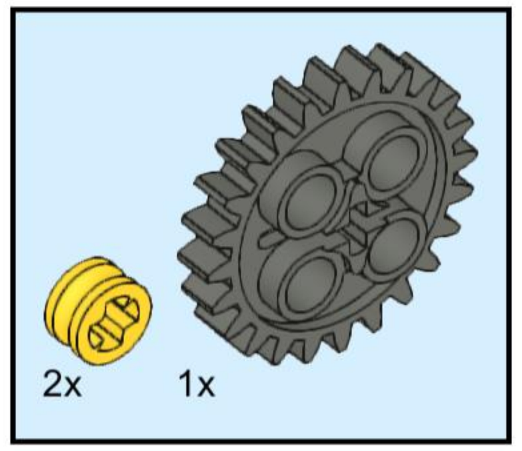


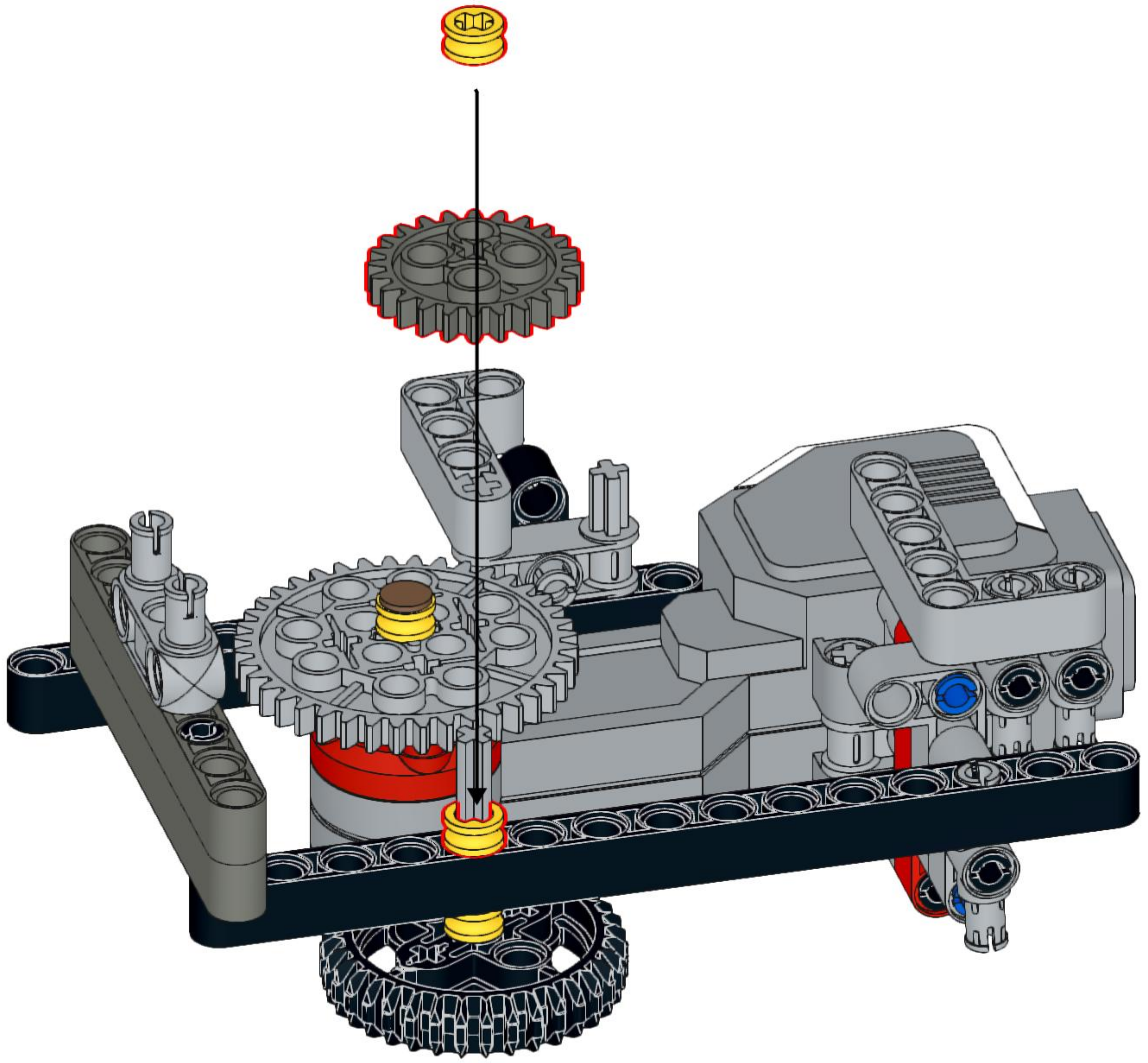

# 55

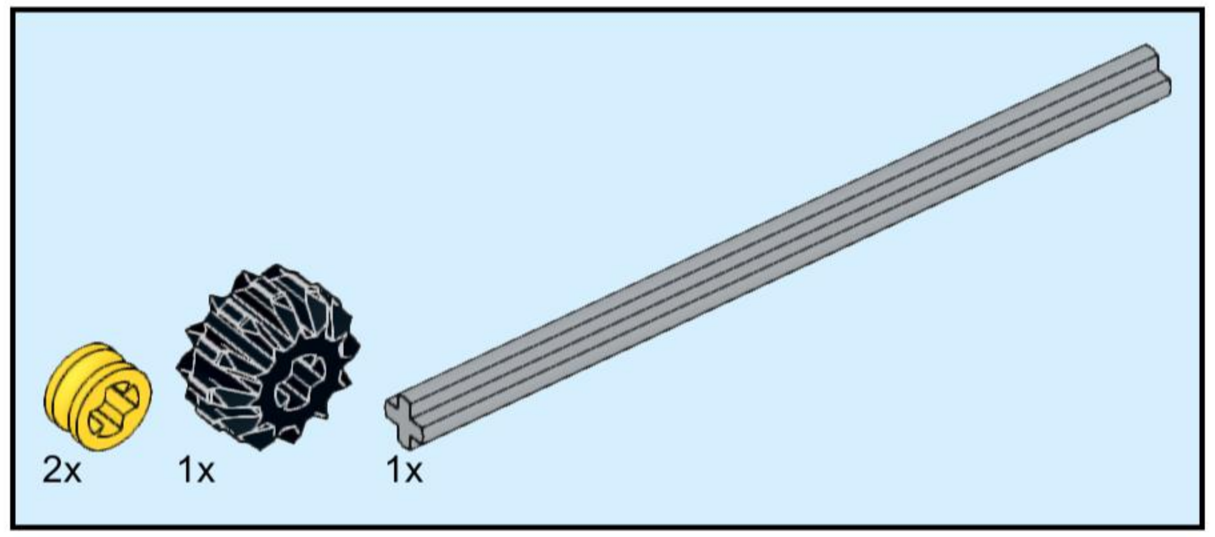


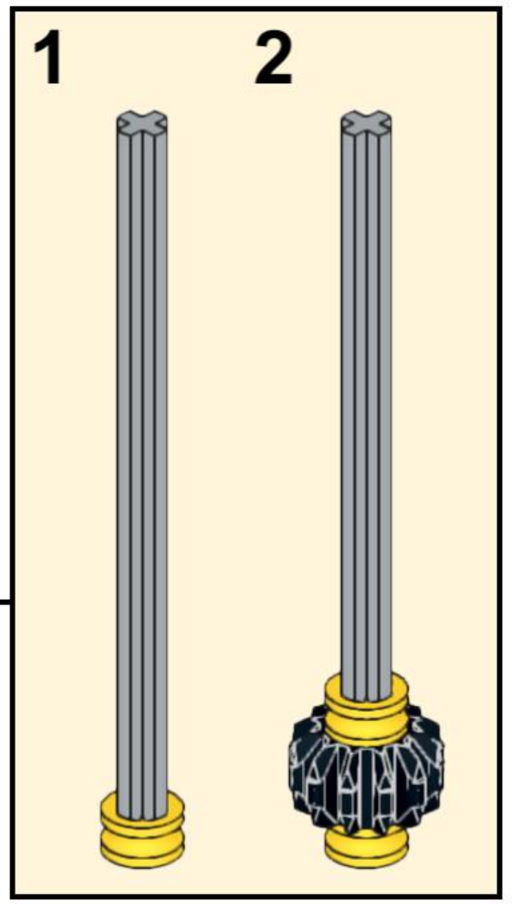


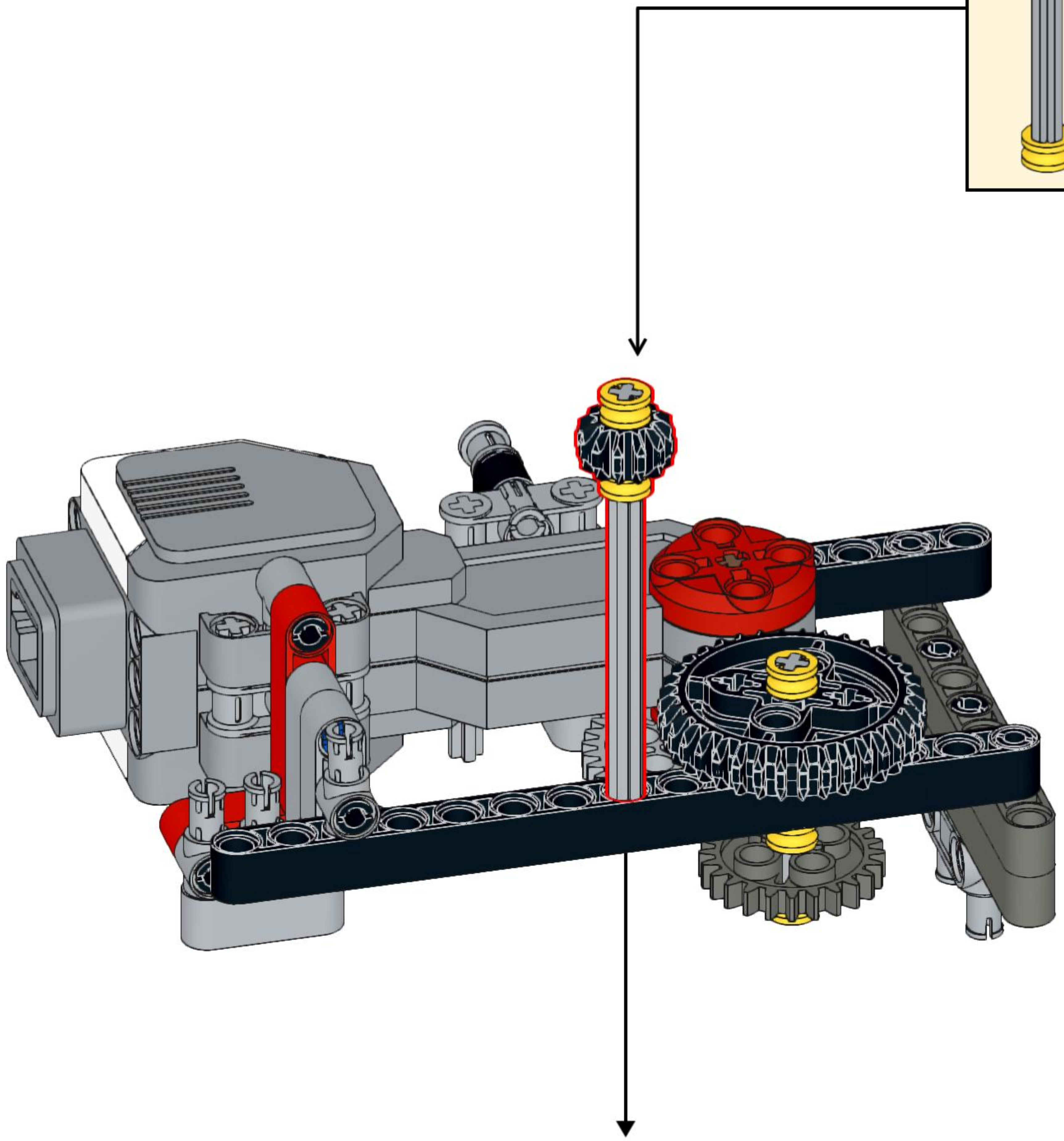

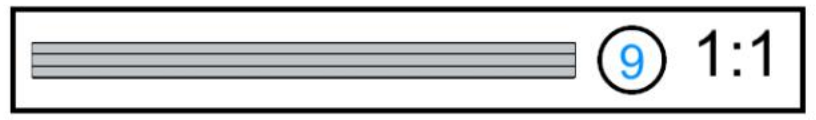

# 56

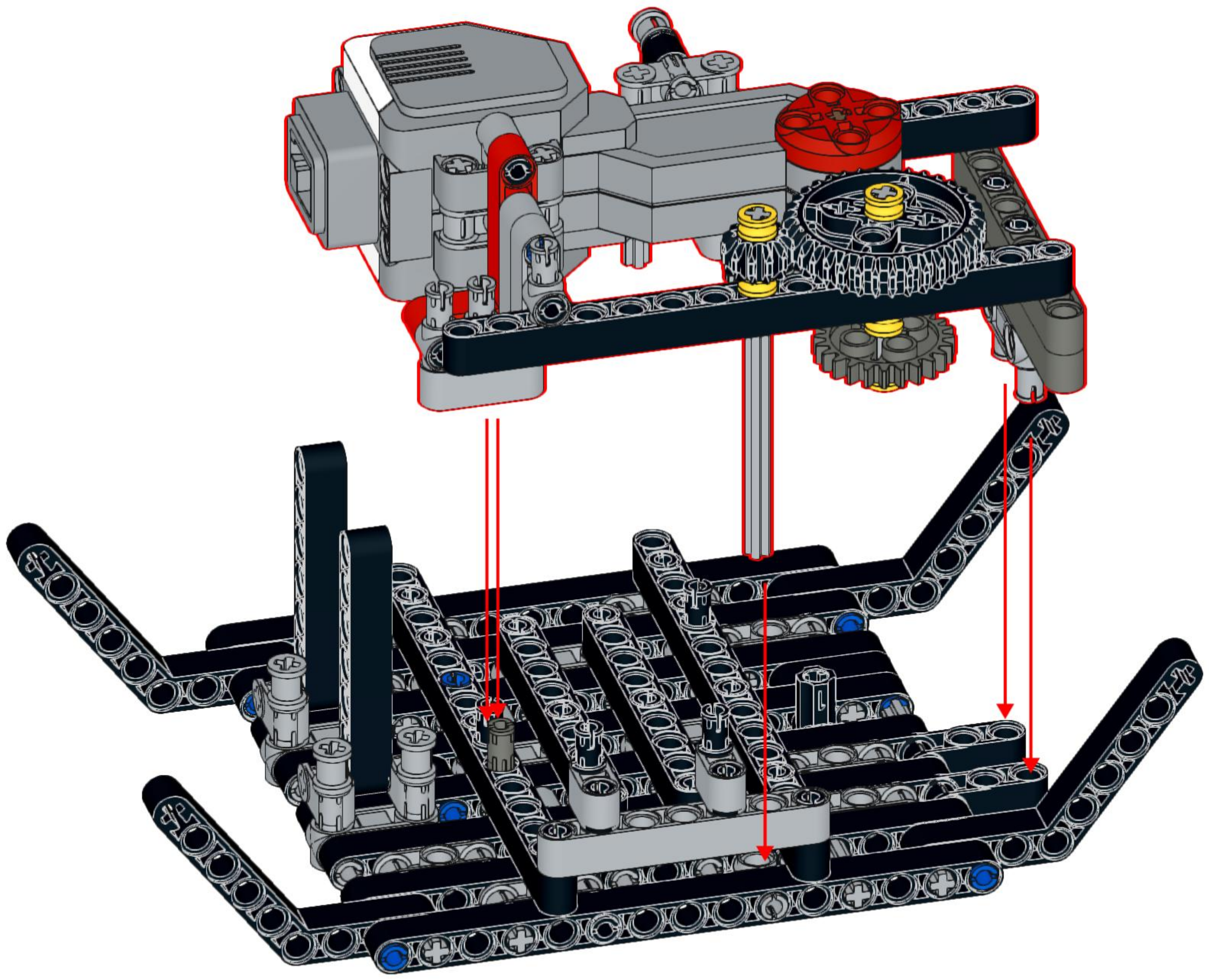

# 57

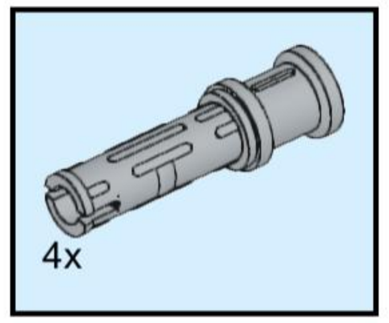


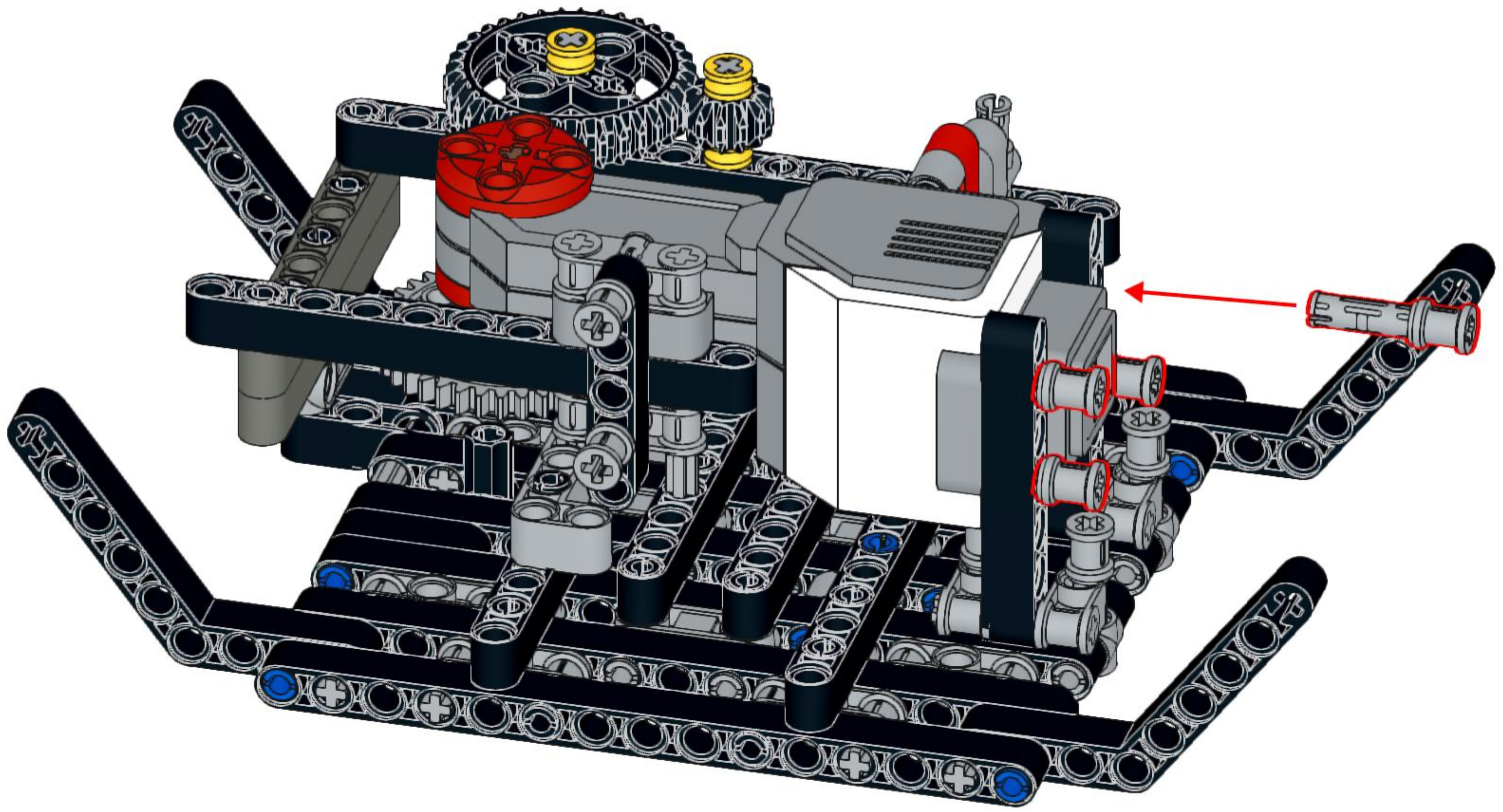

## 58

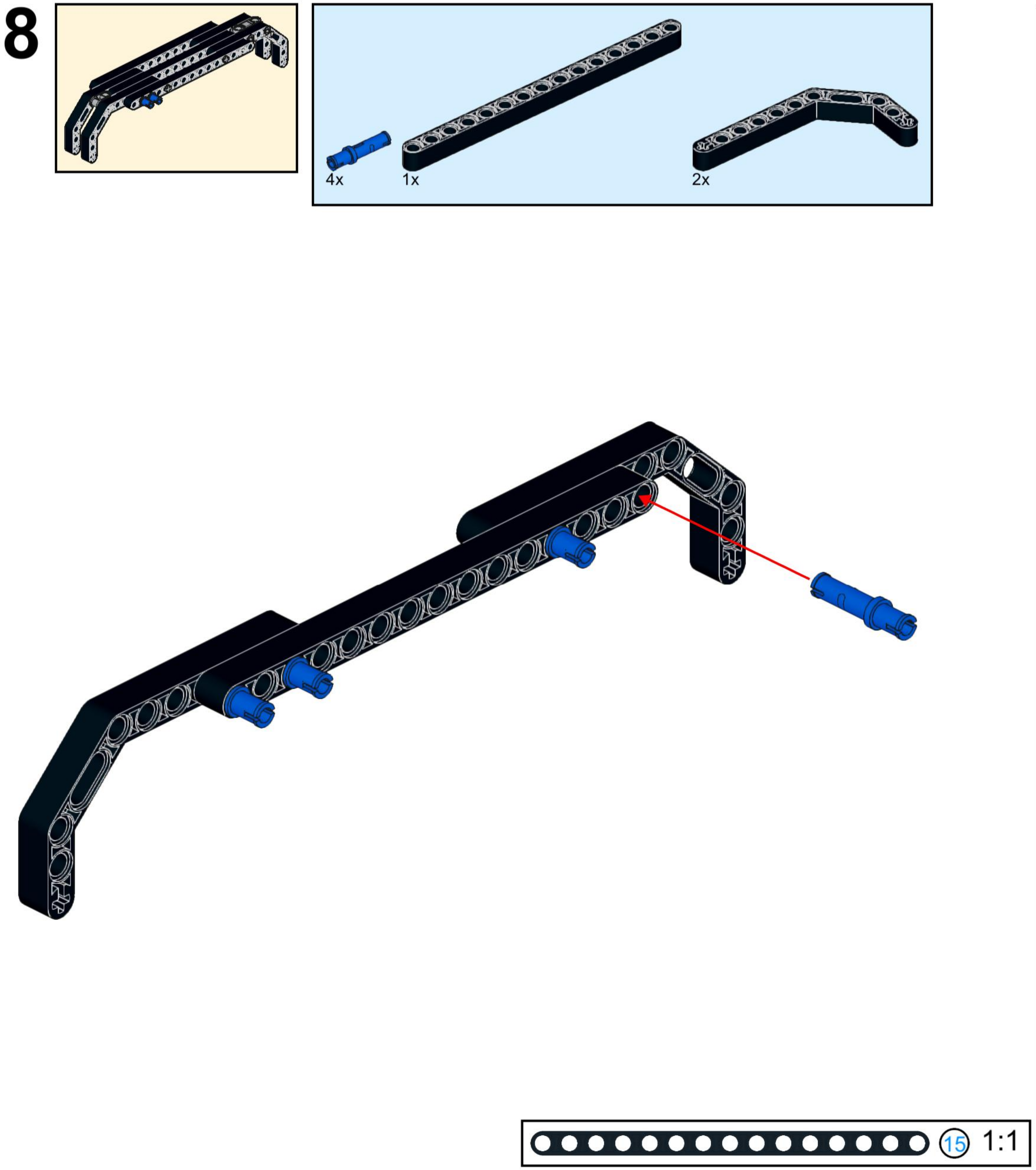


## 59

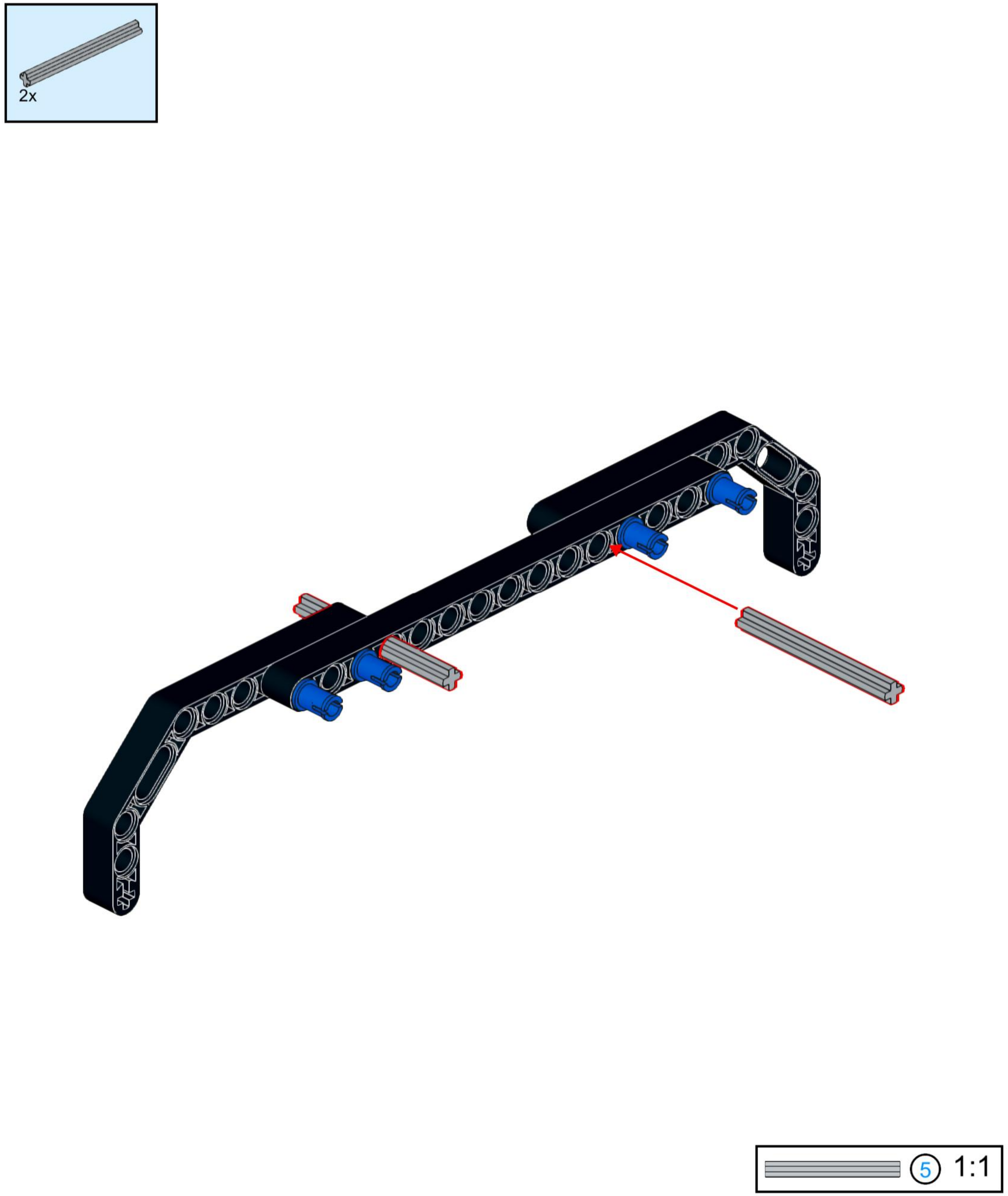

## 60

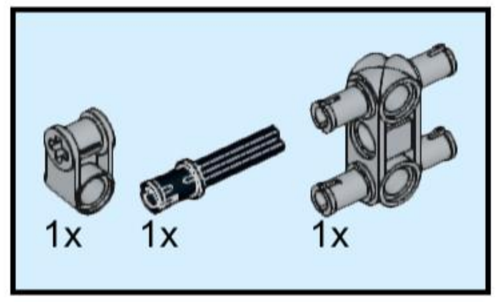


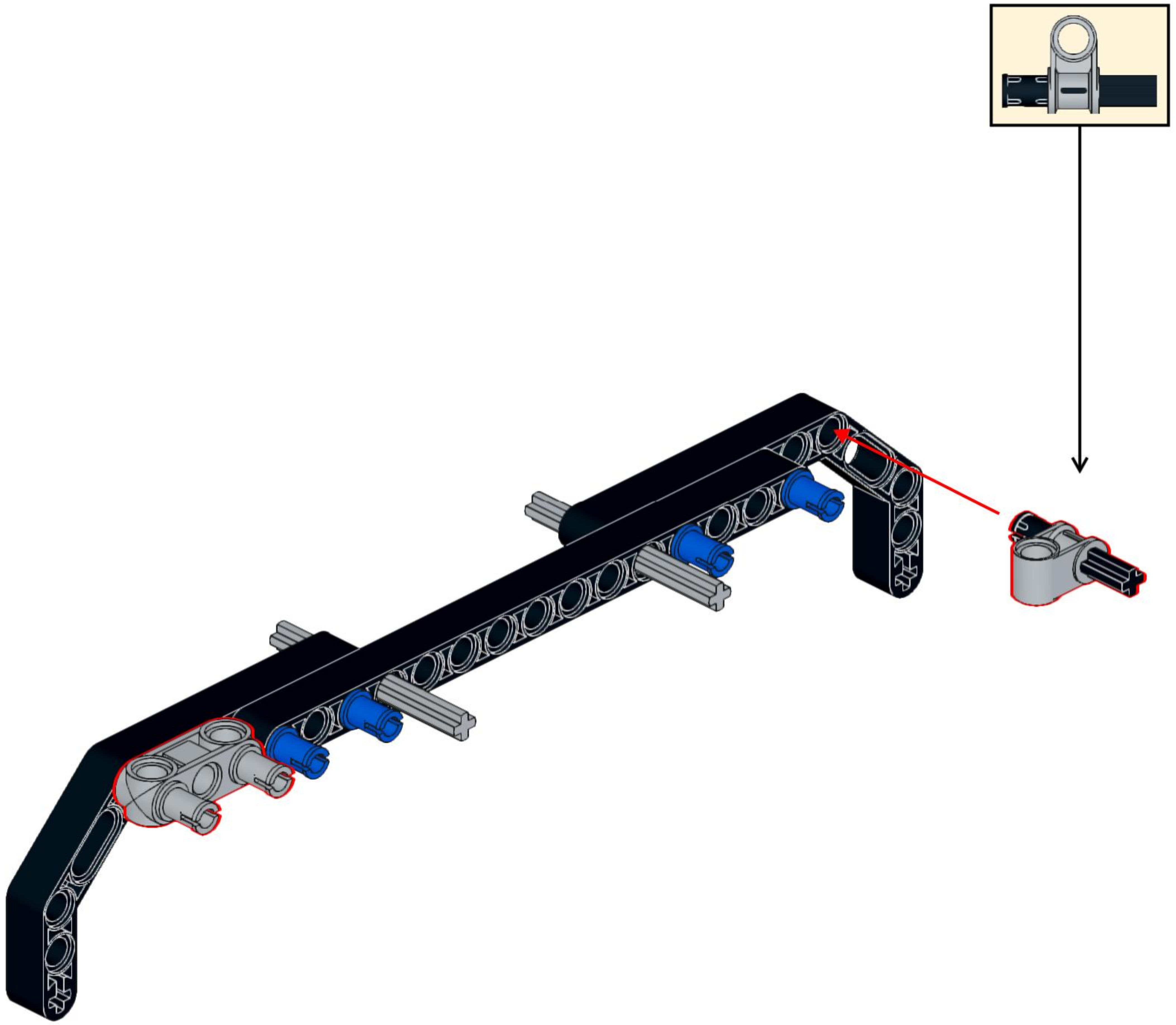

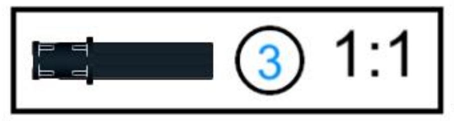


## 61

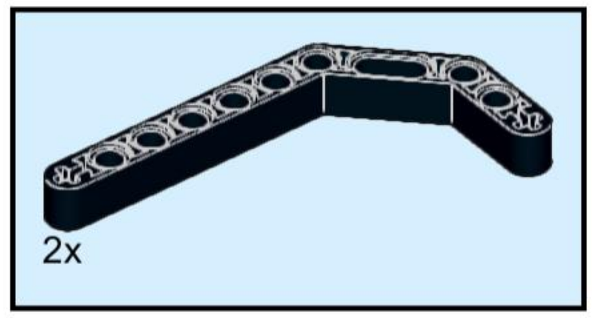


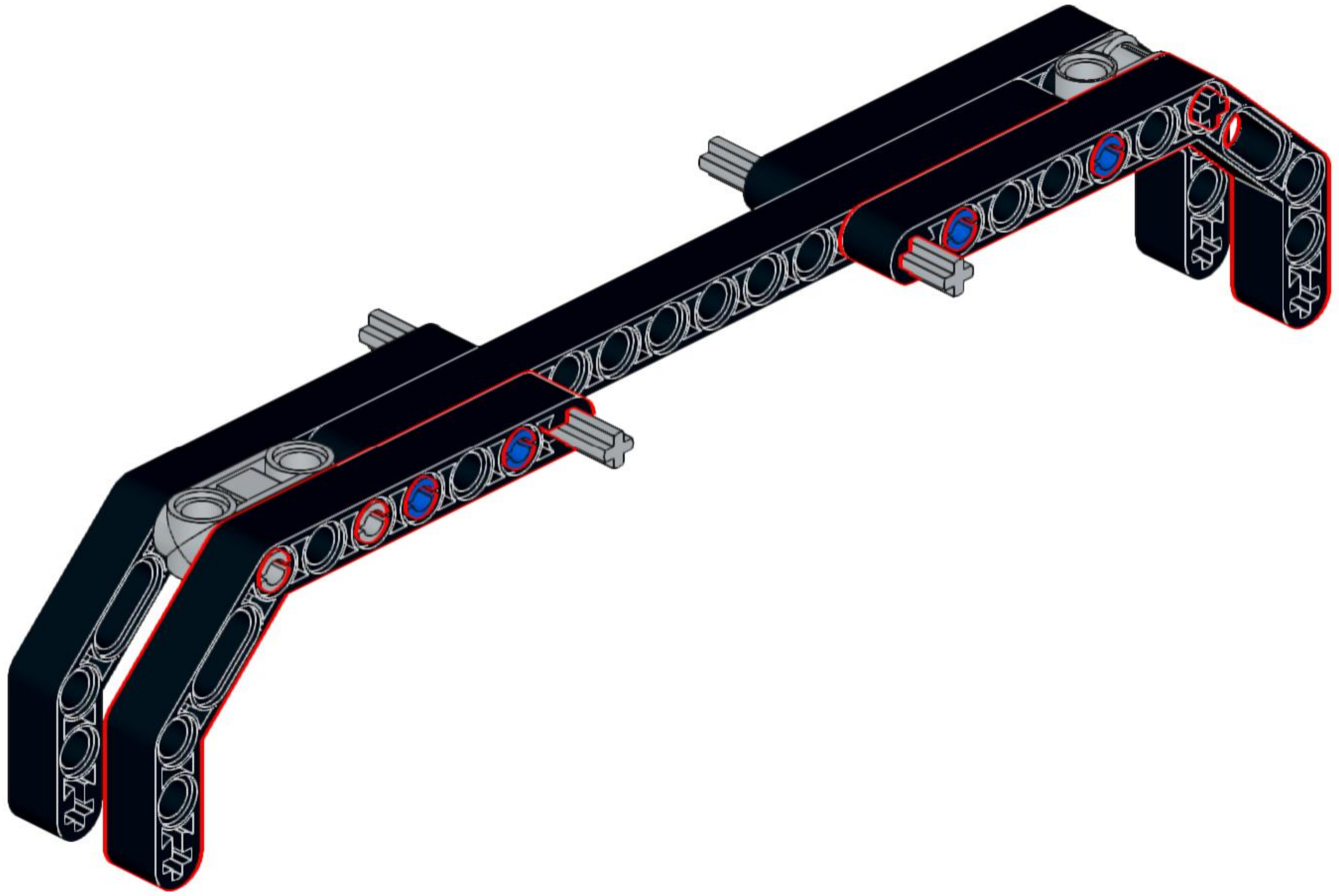

62

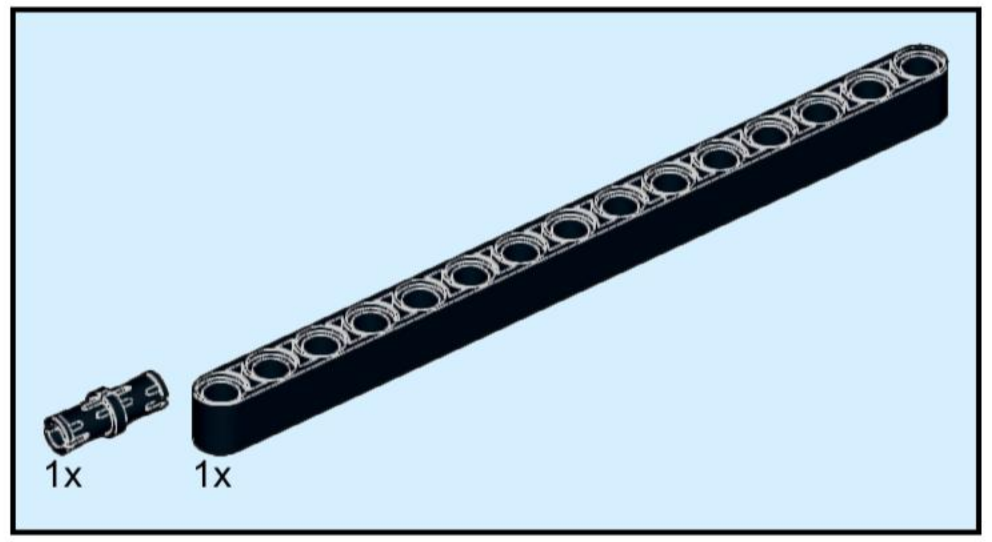


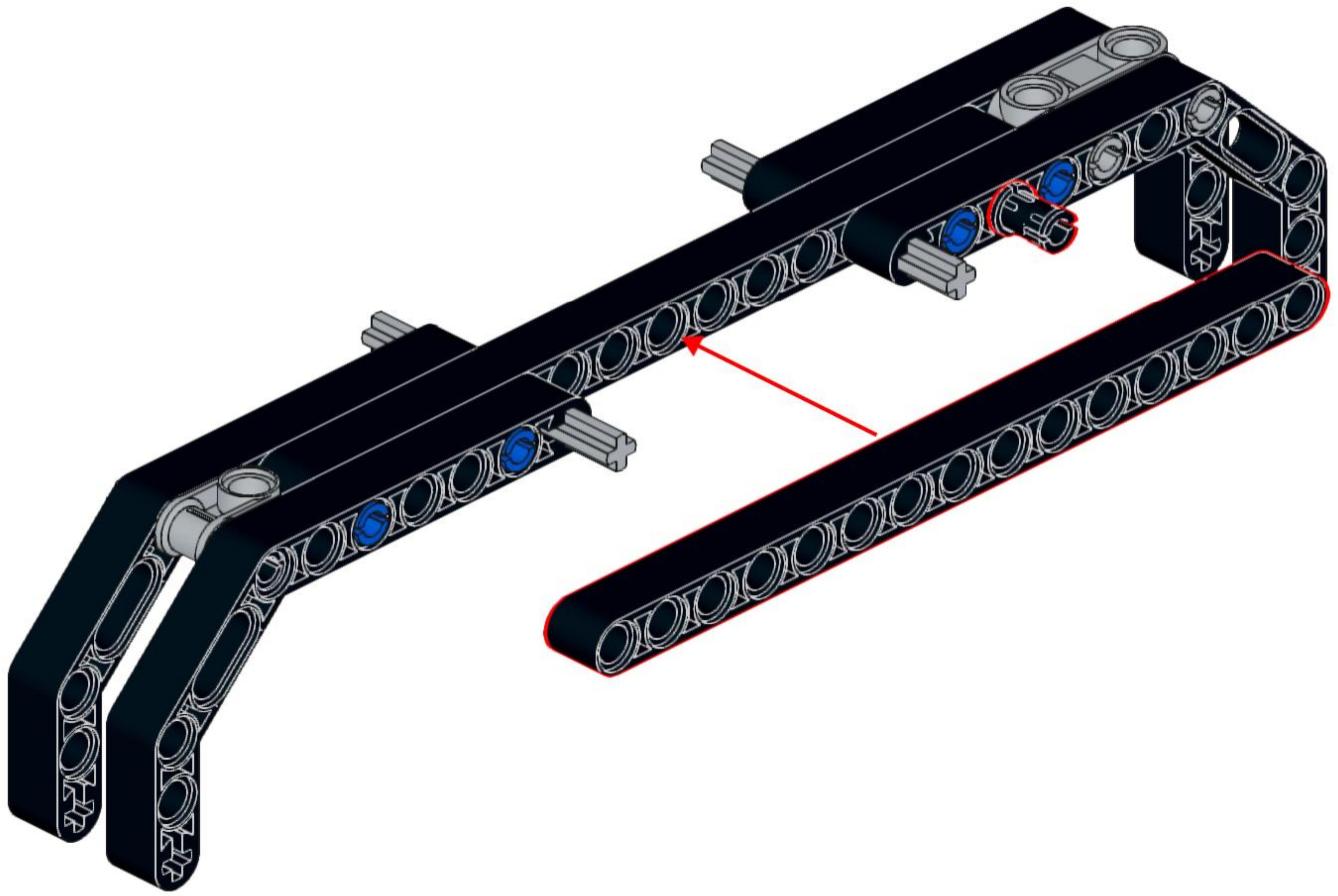

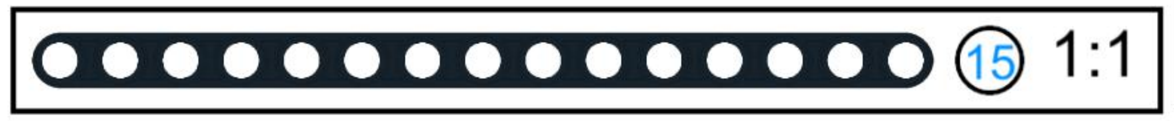


63

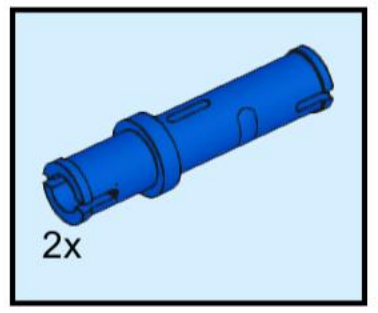


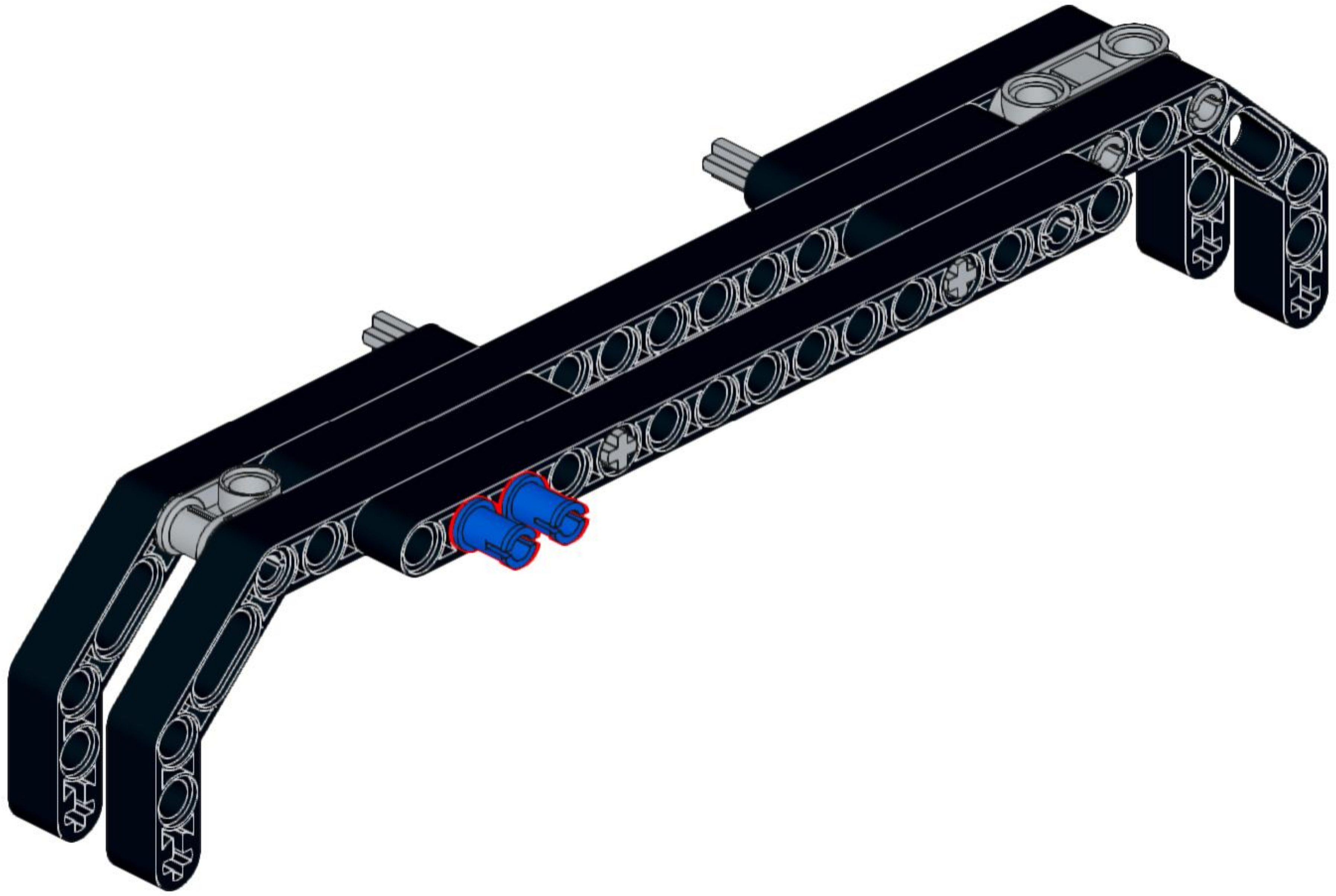

## 64

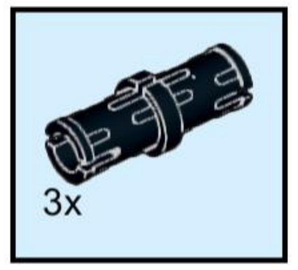


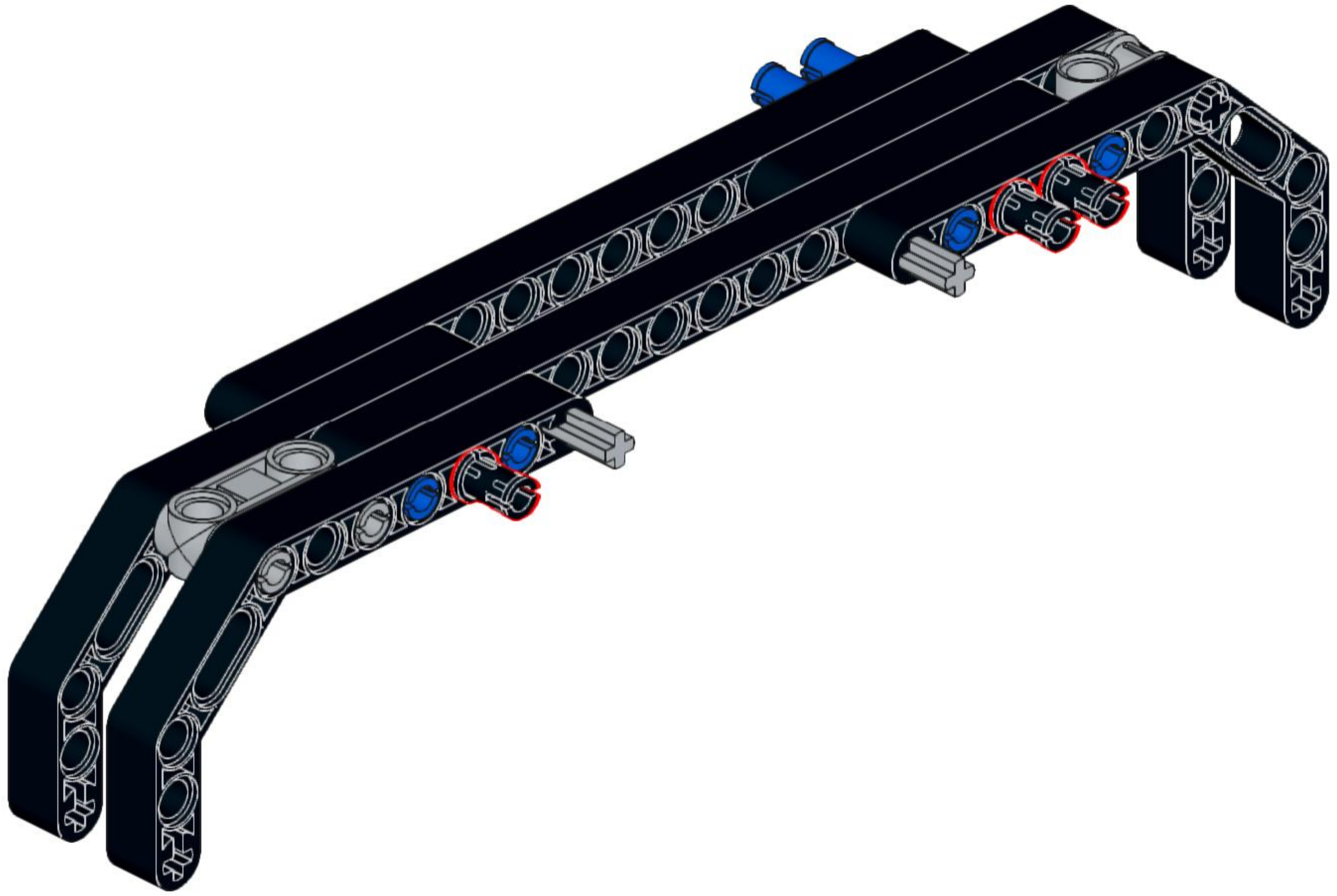

## 65

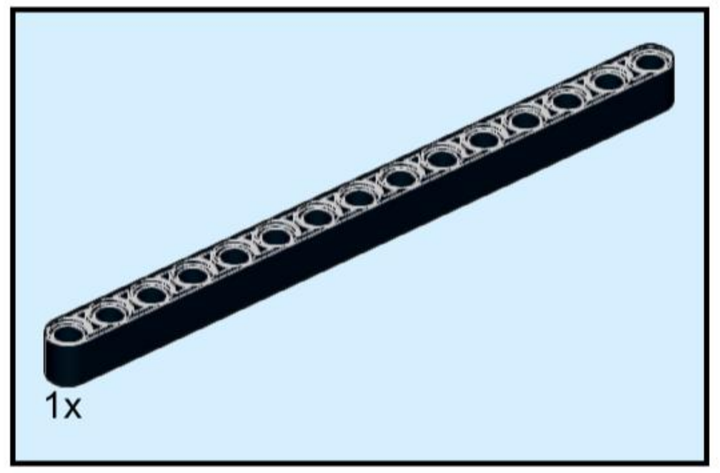


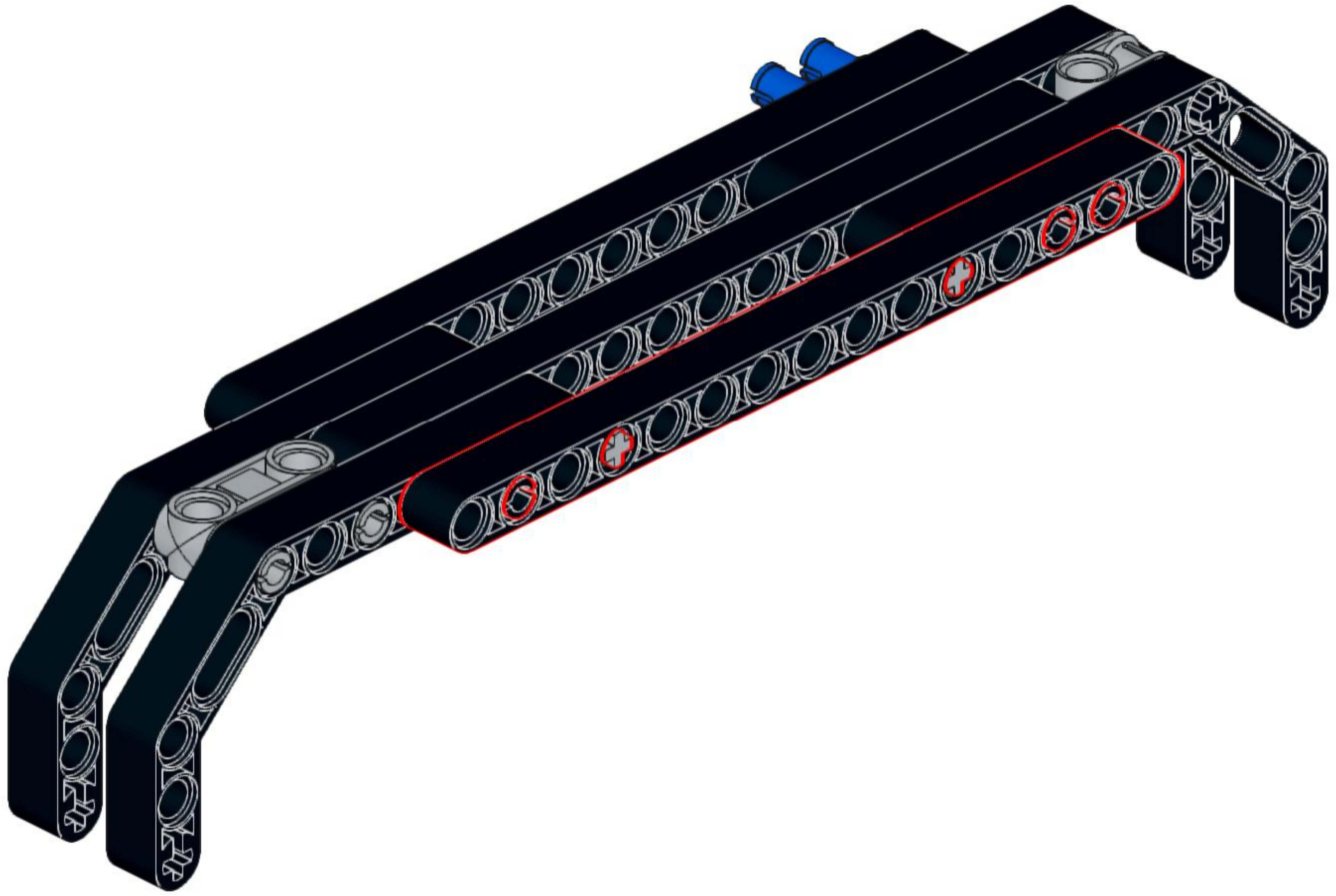

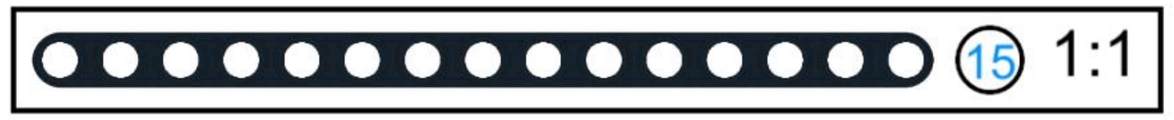

# 66

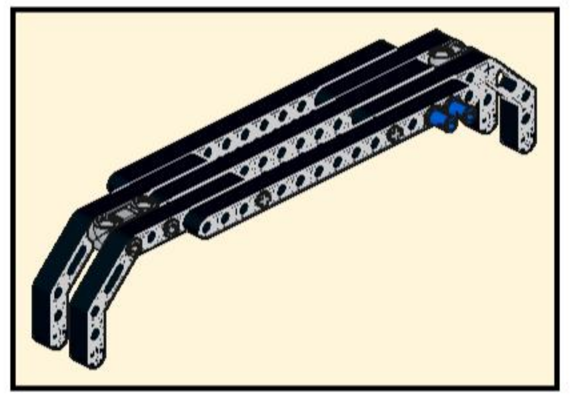

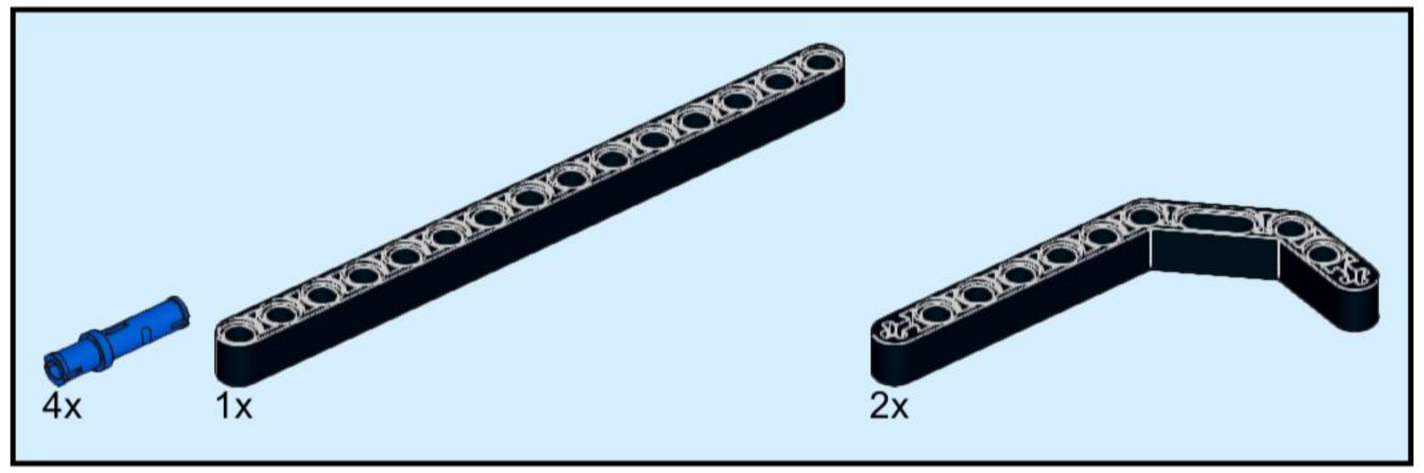


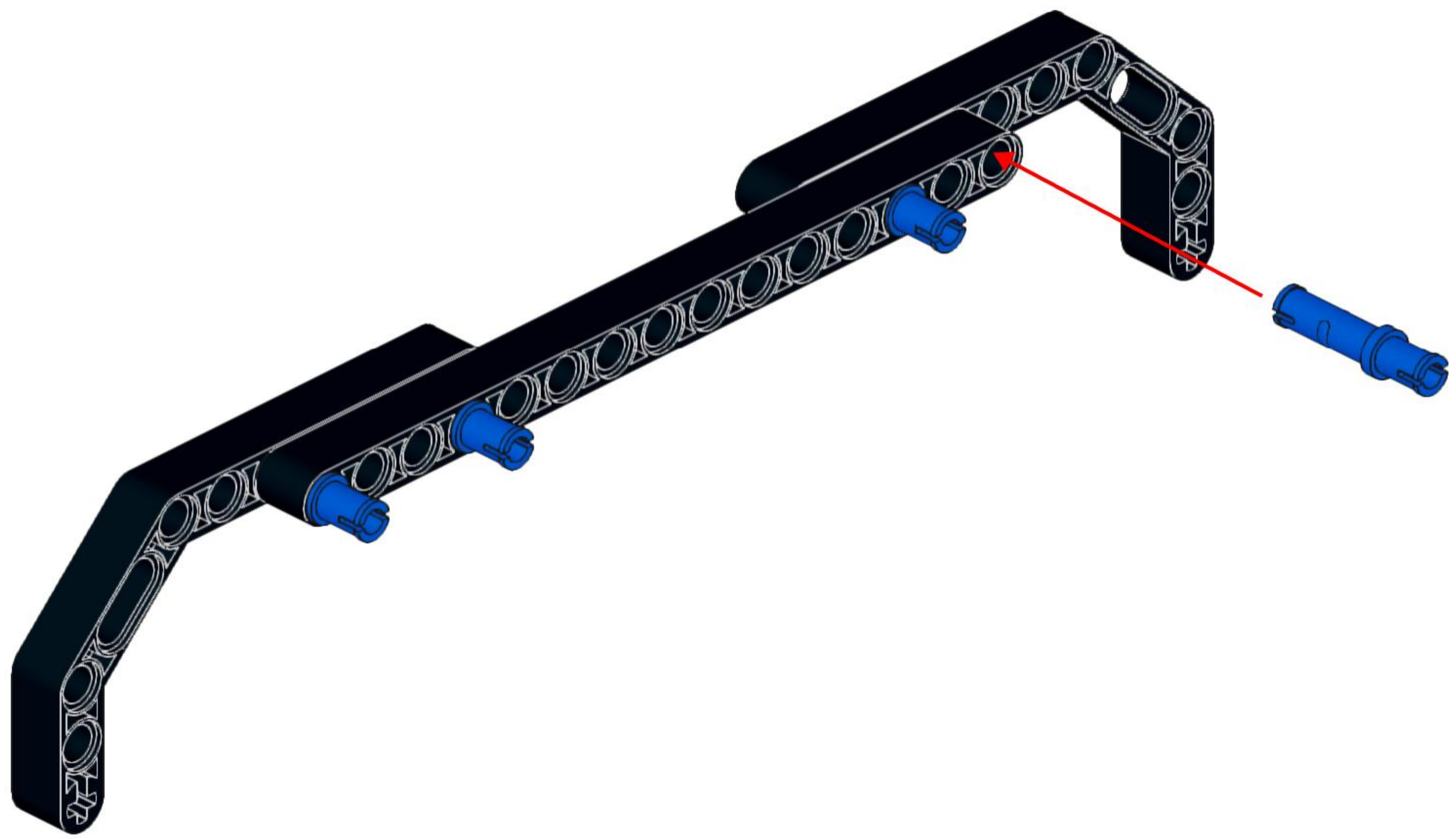

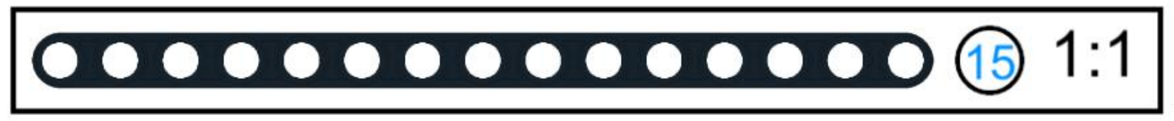


# 67

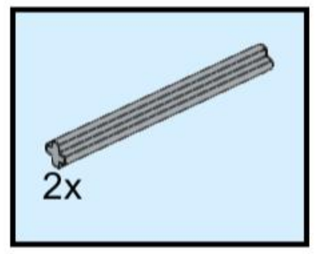


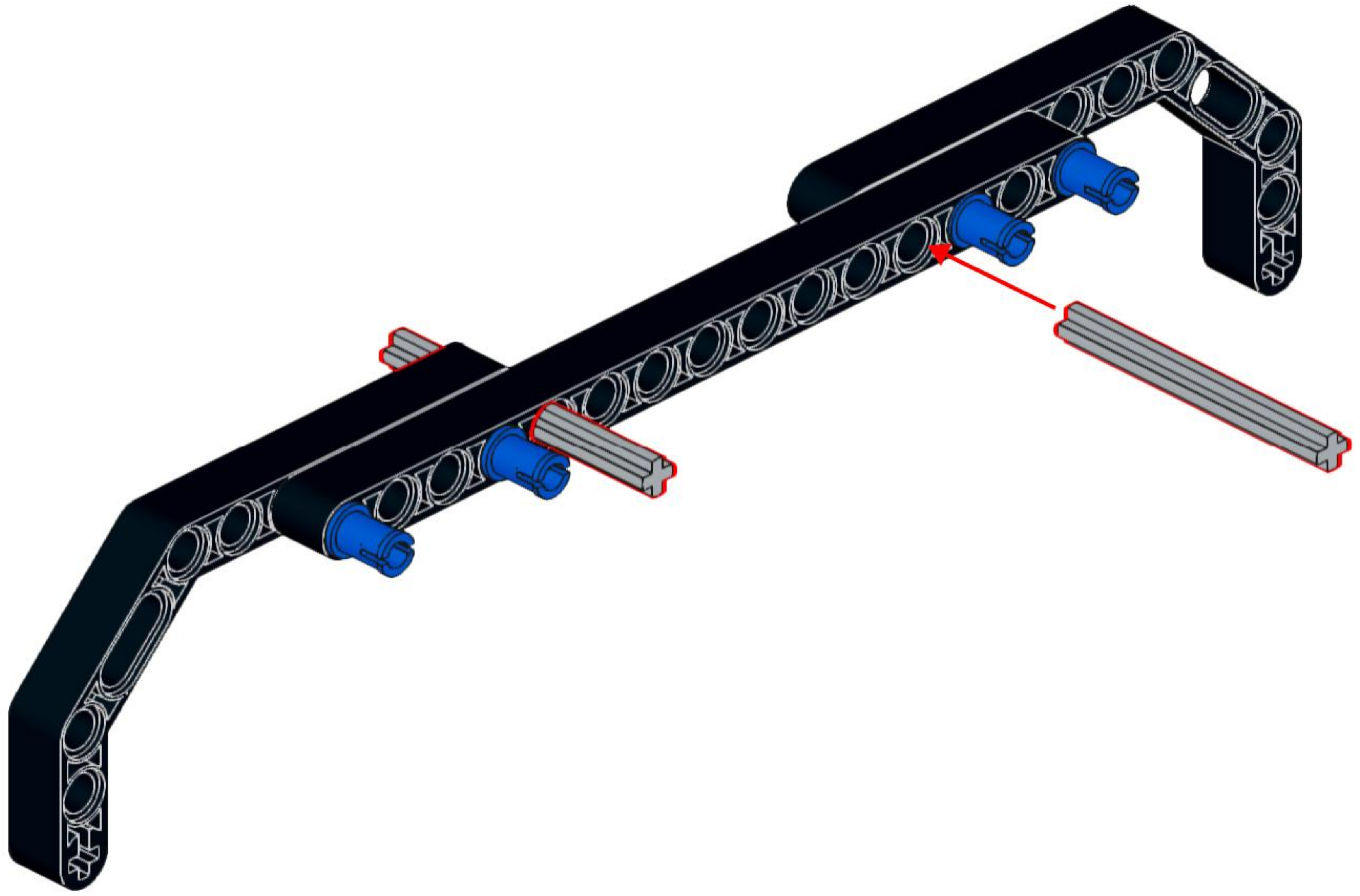

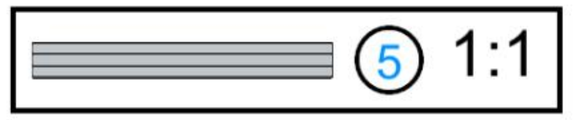

## 68

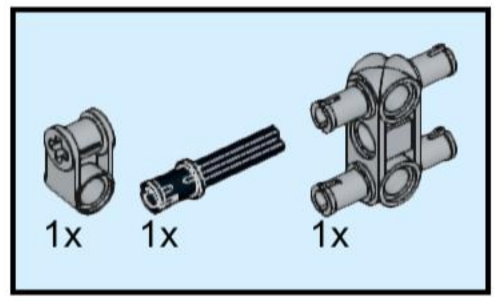


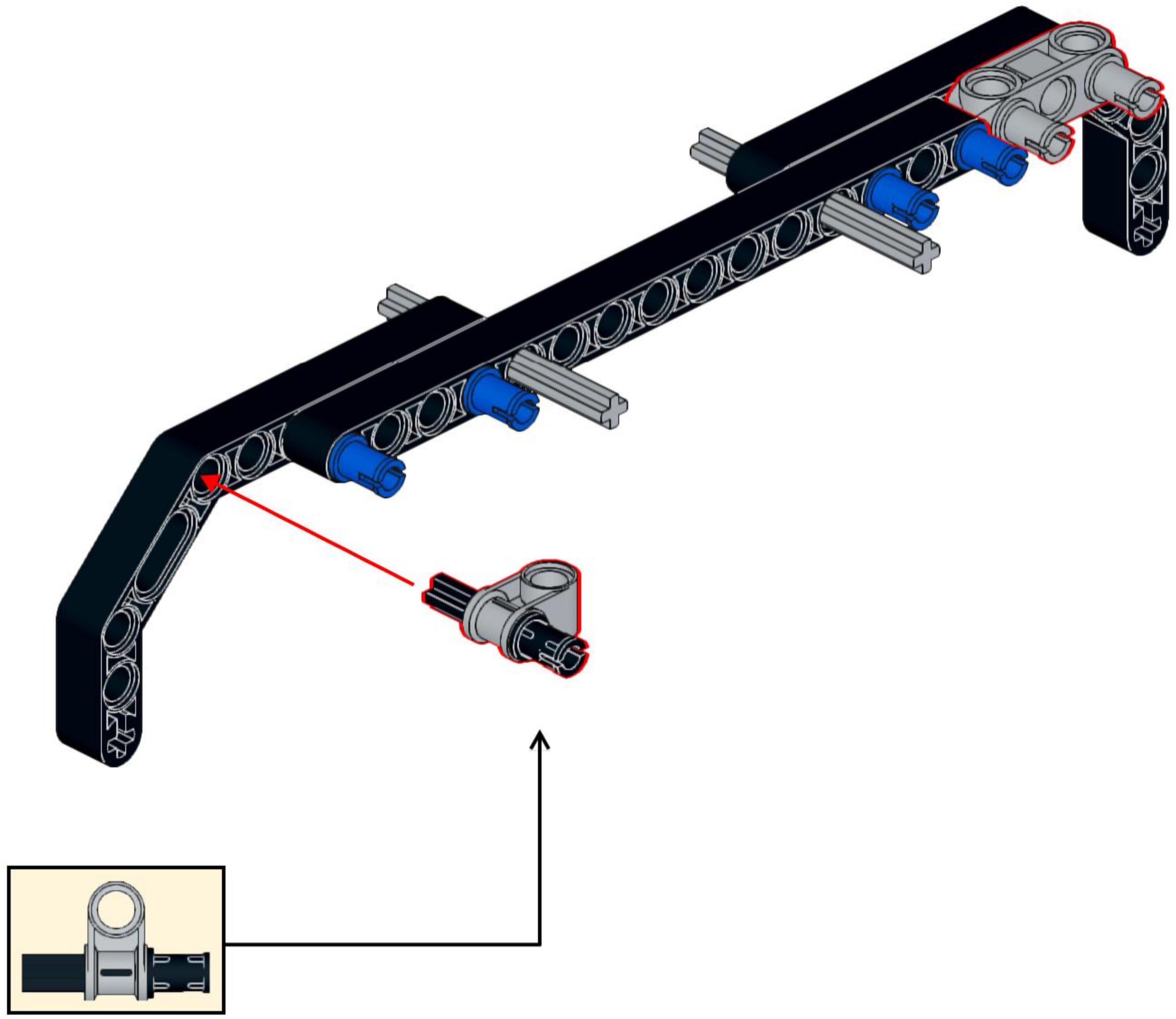

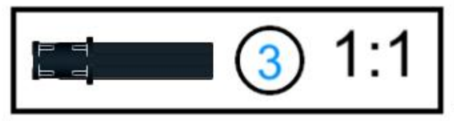


## 69

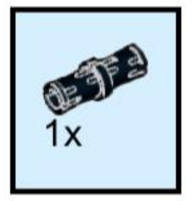


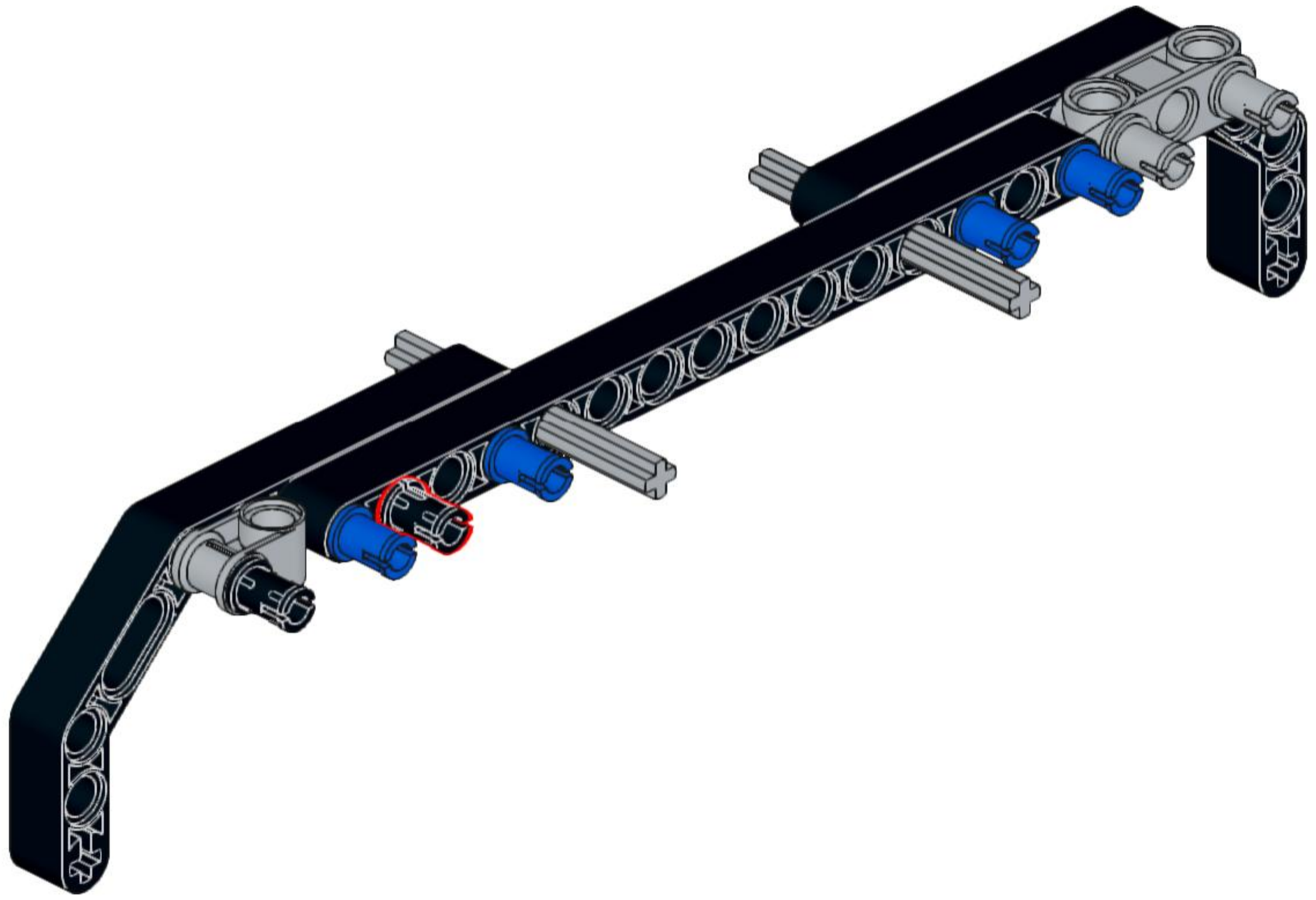

**70**

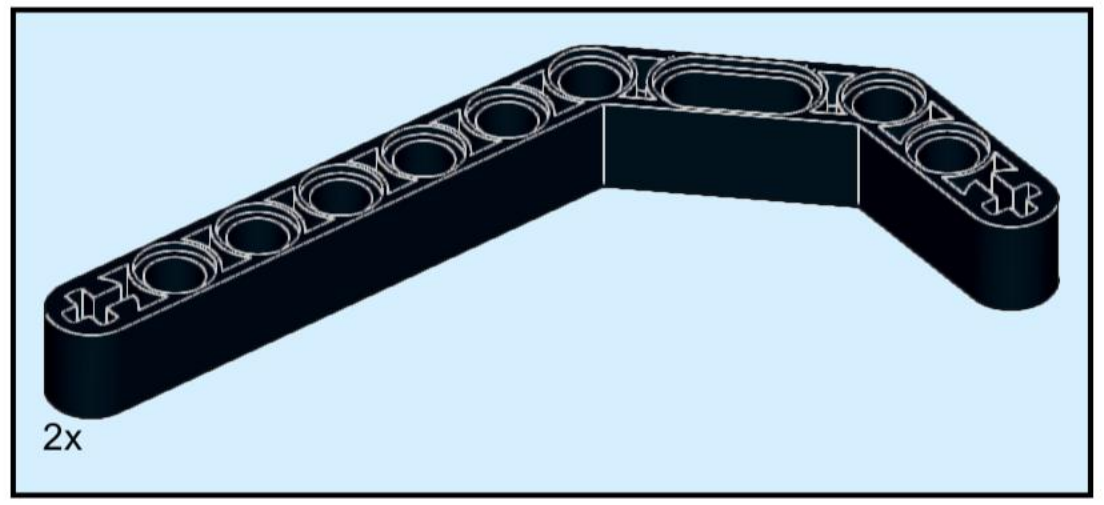


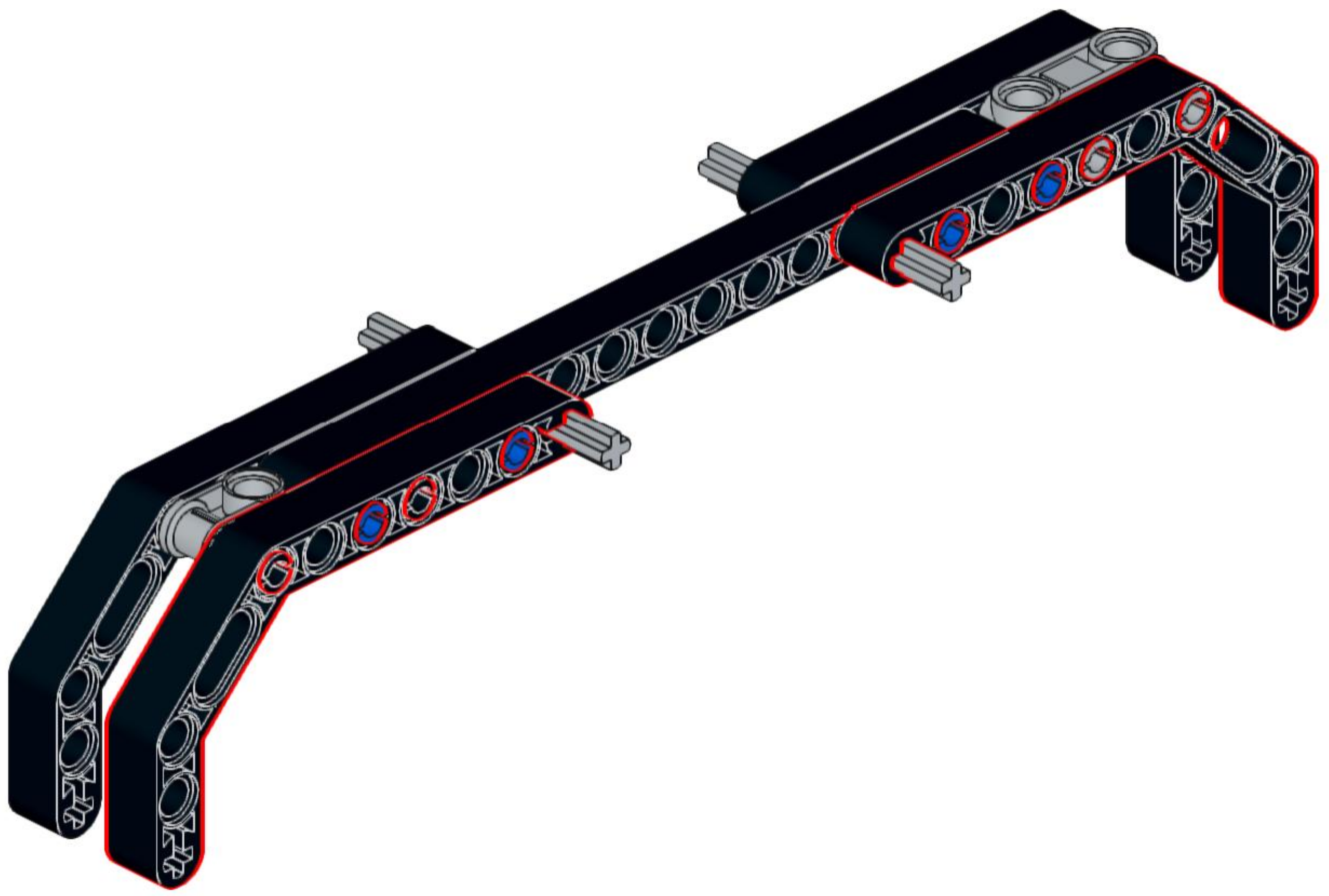

**71**

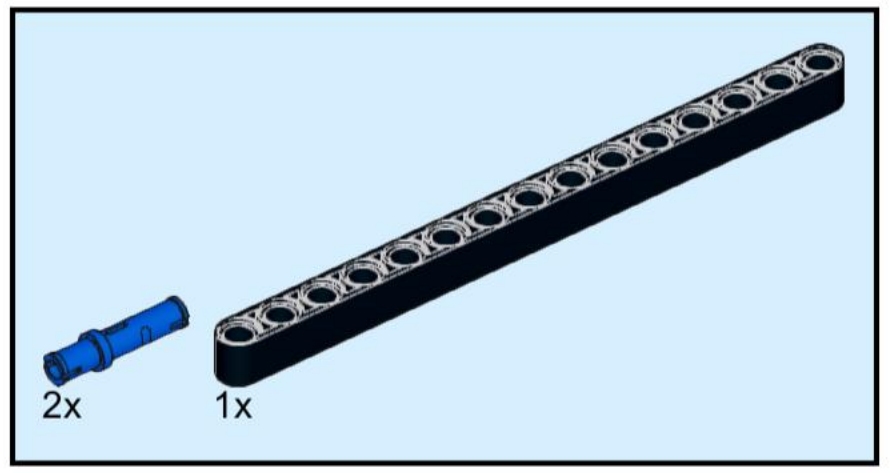


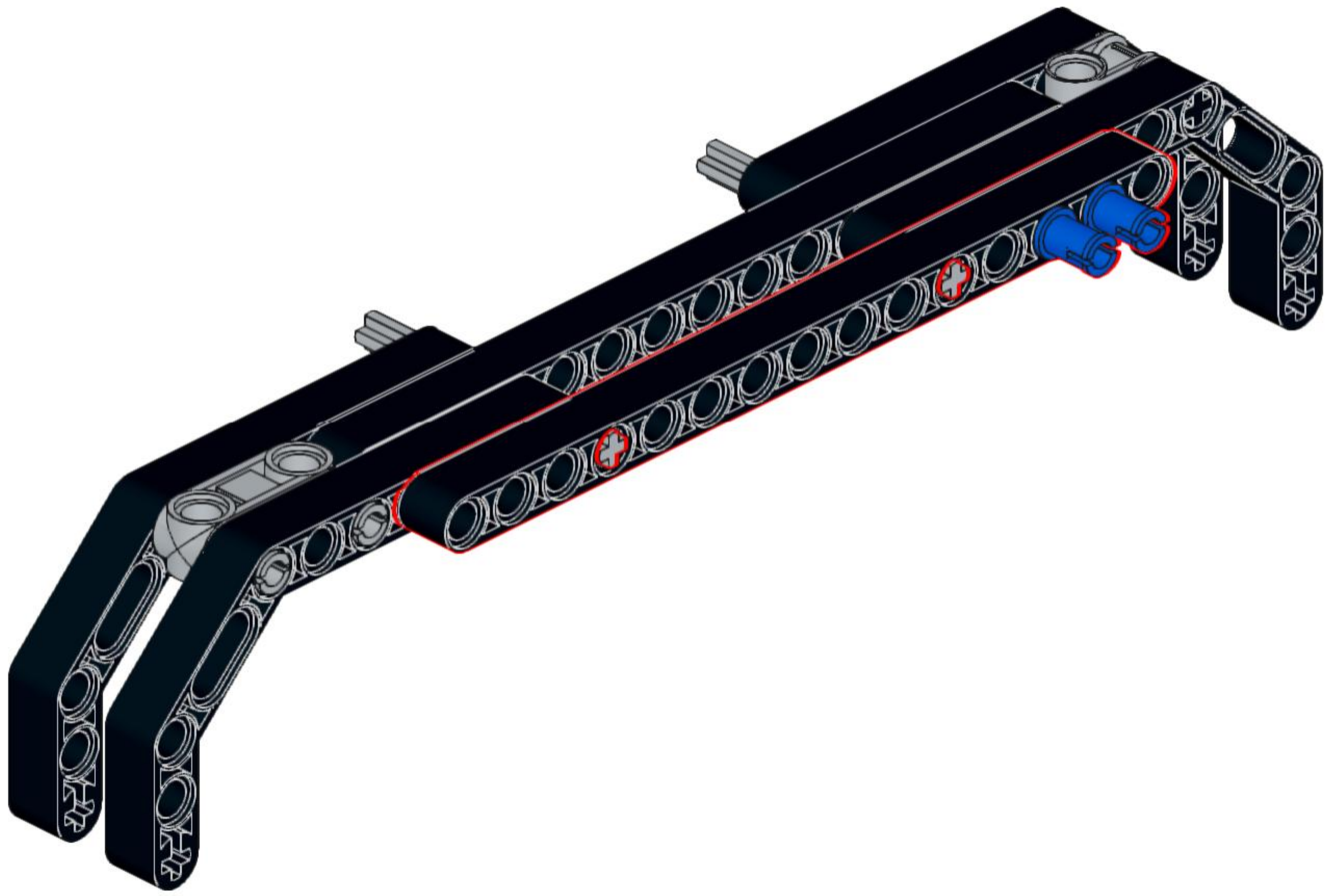

72

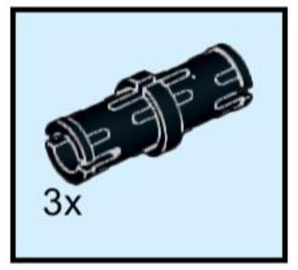


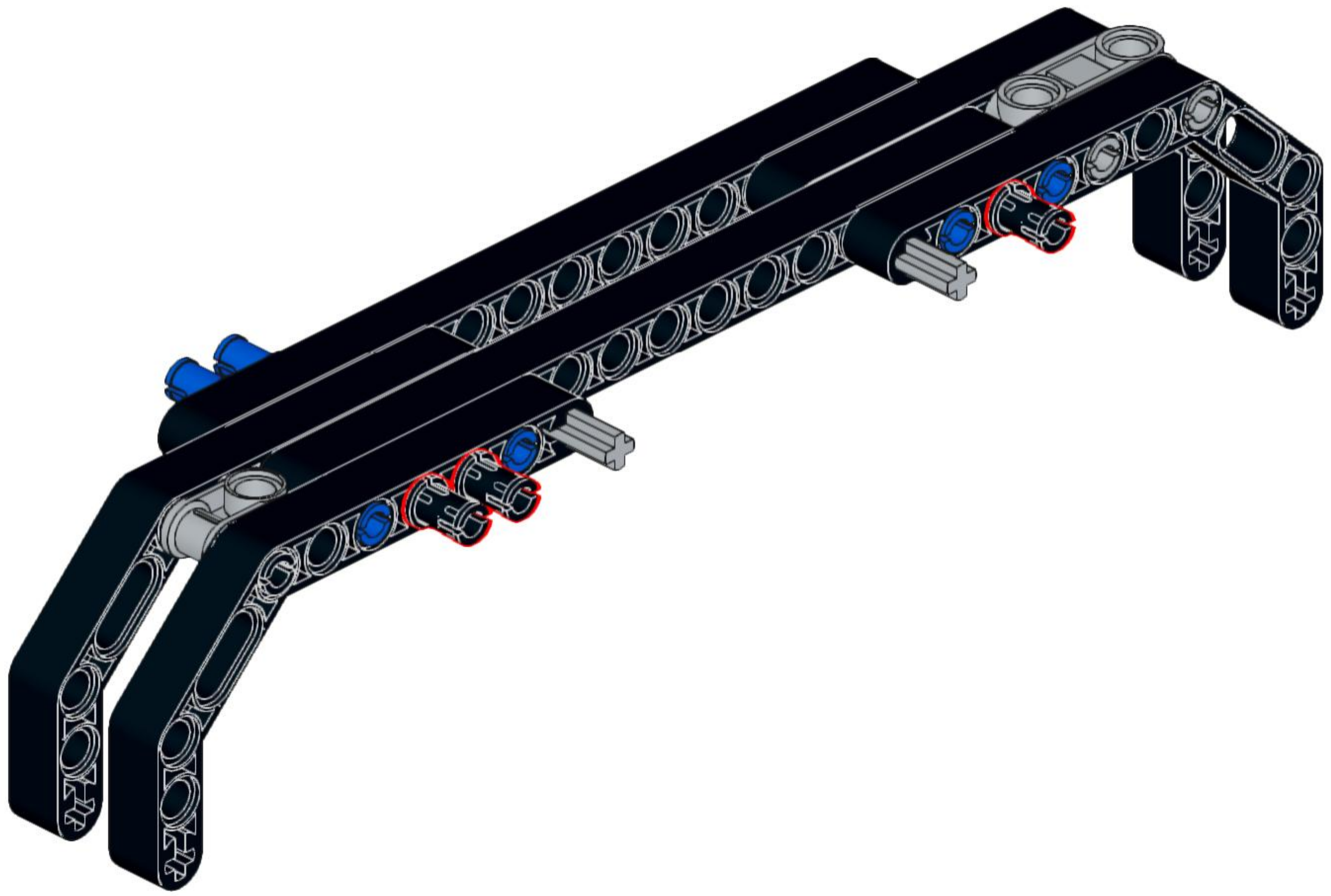

73

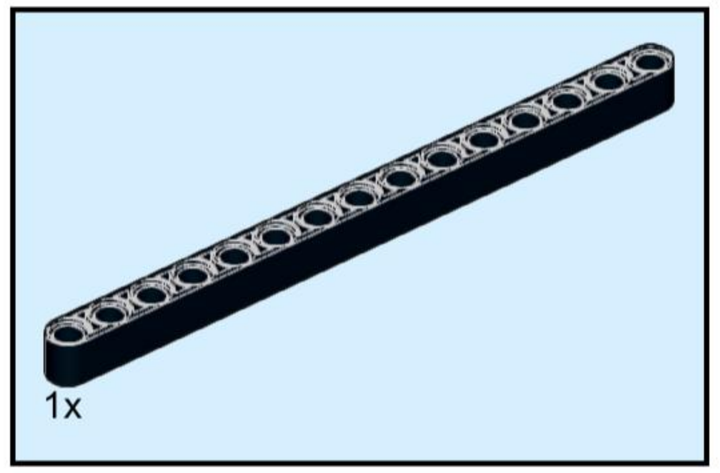


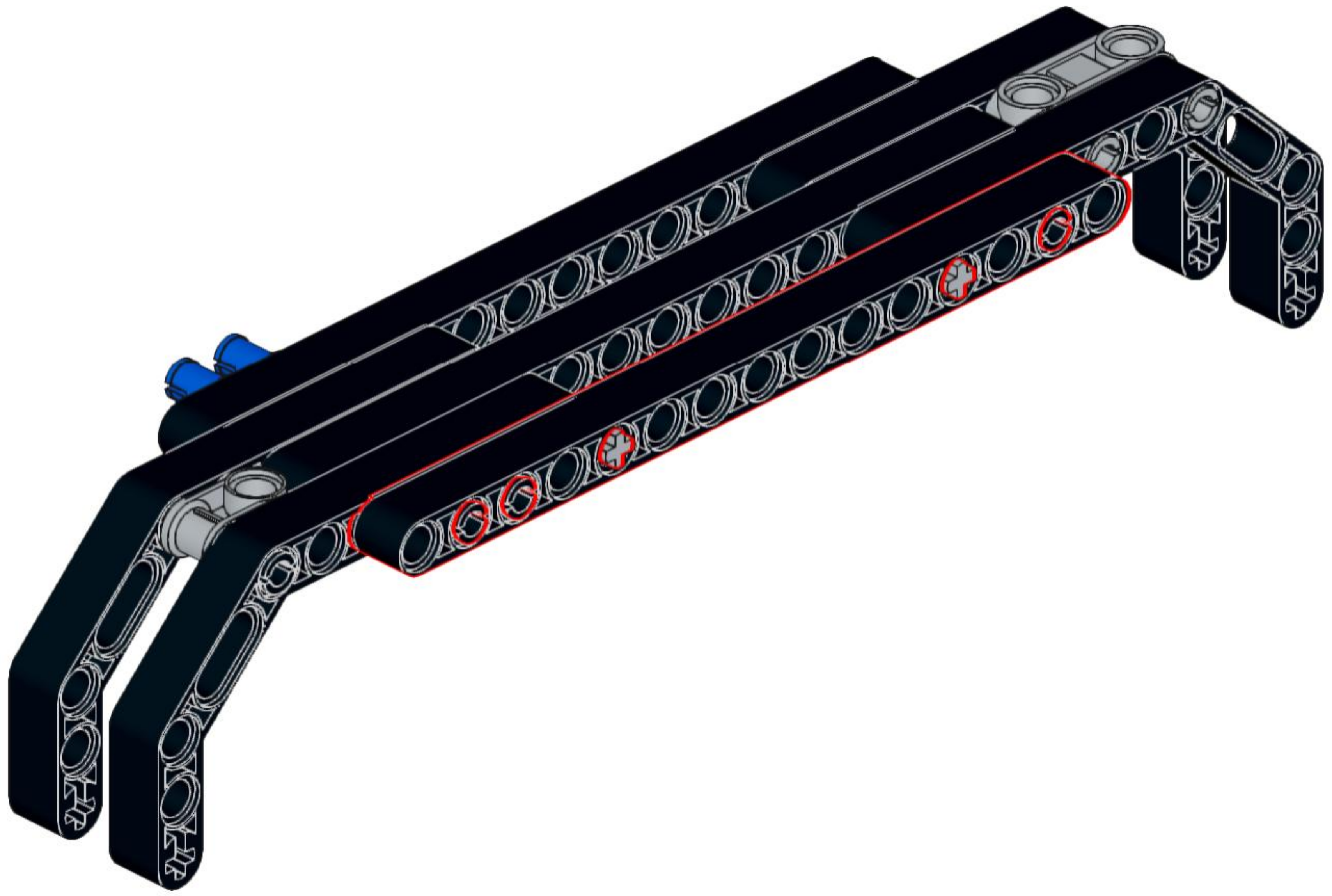

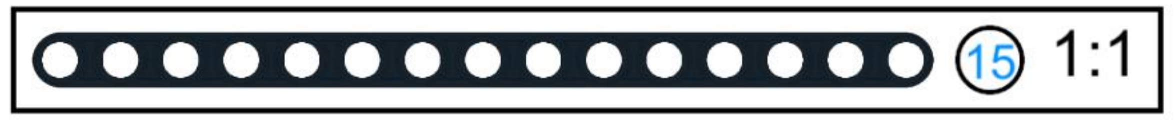

74

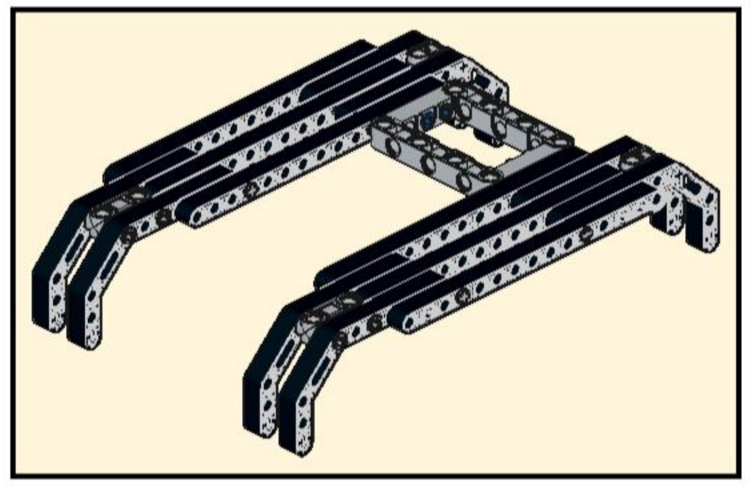

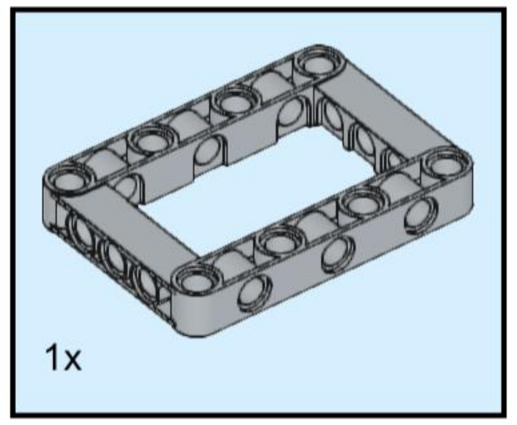


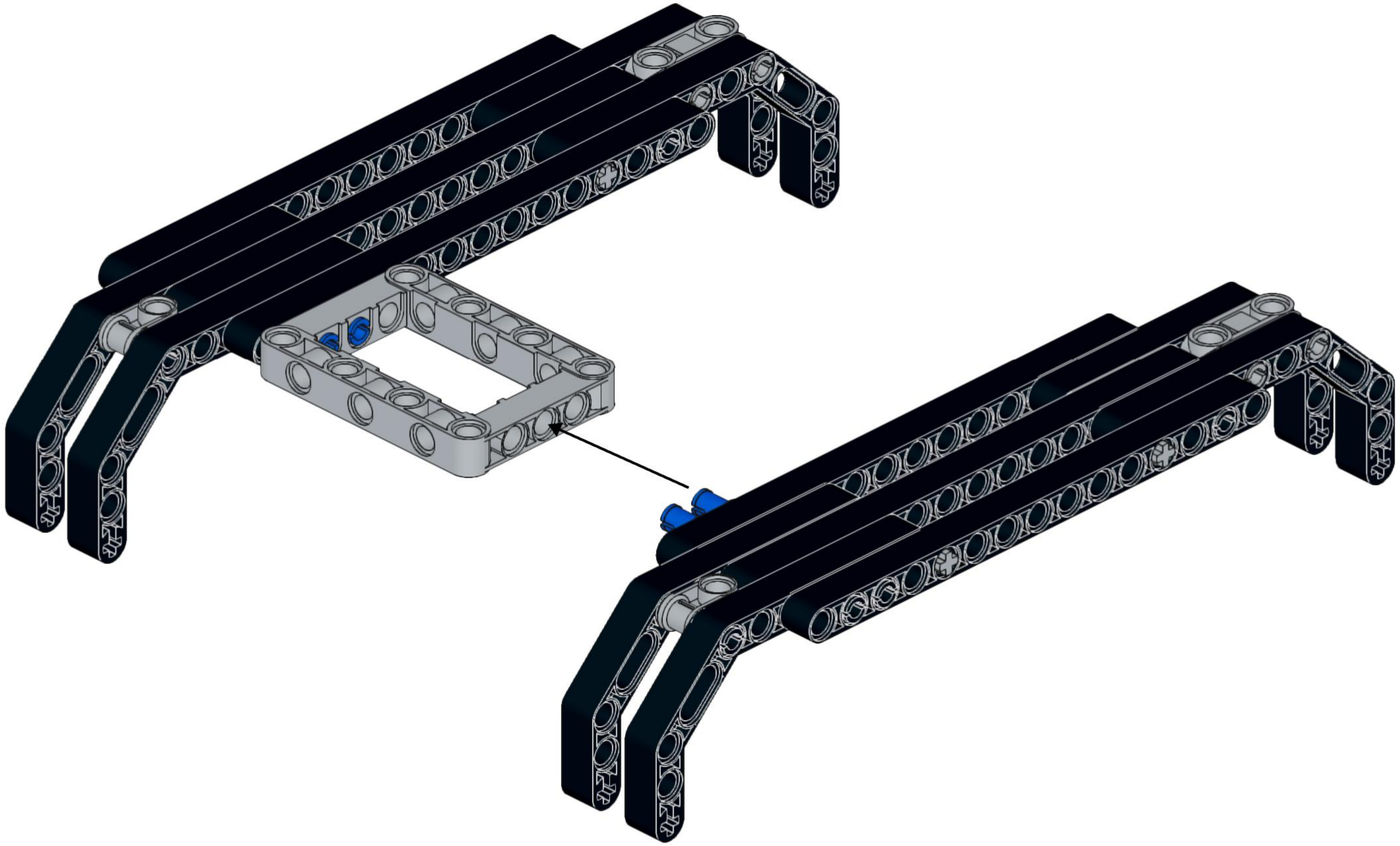

# 75

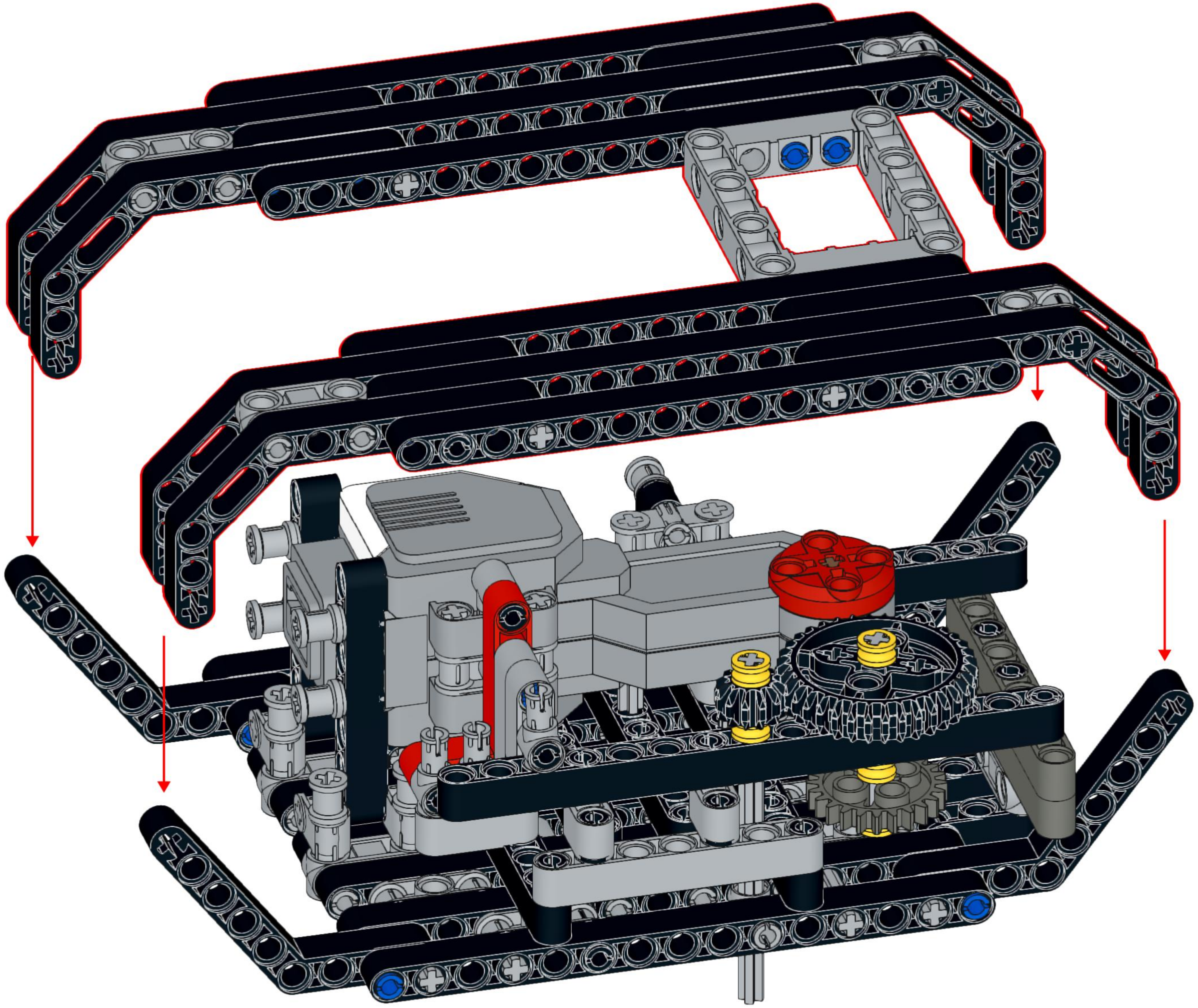

76

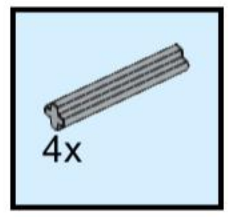


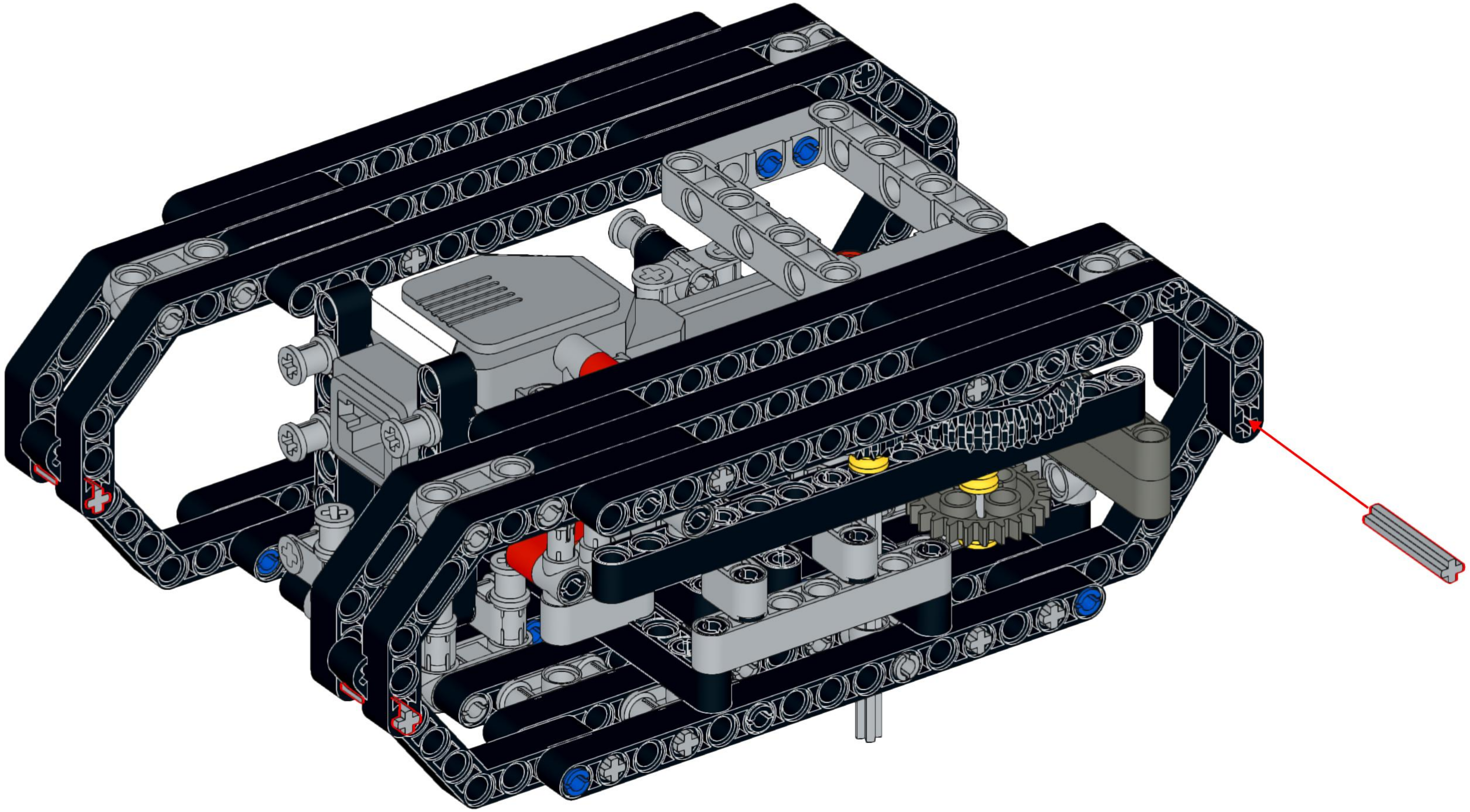

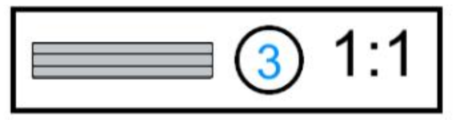

**77**

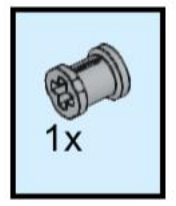


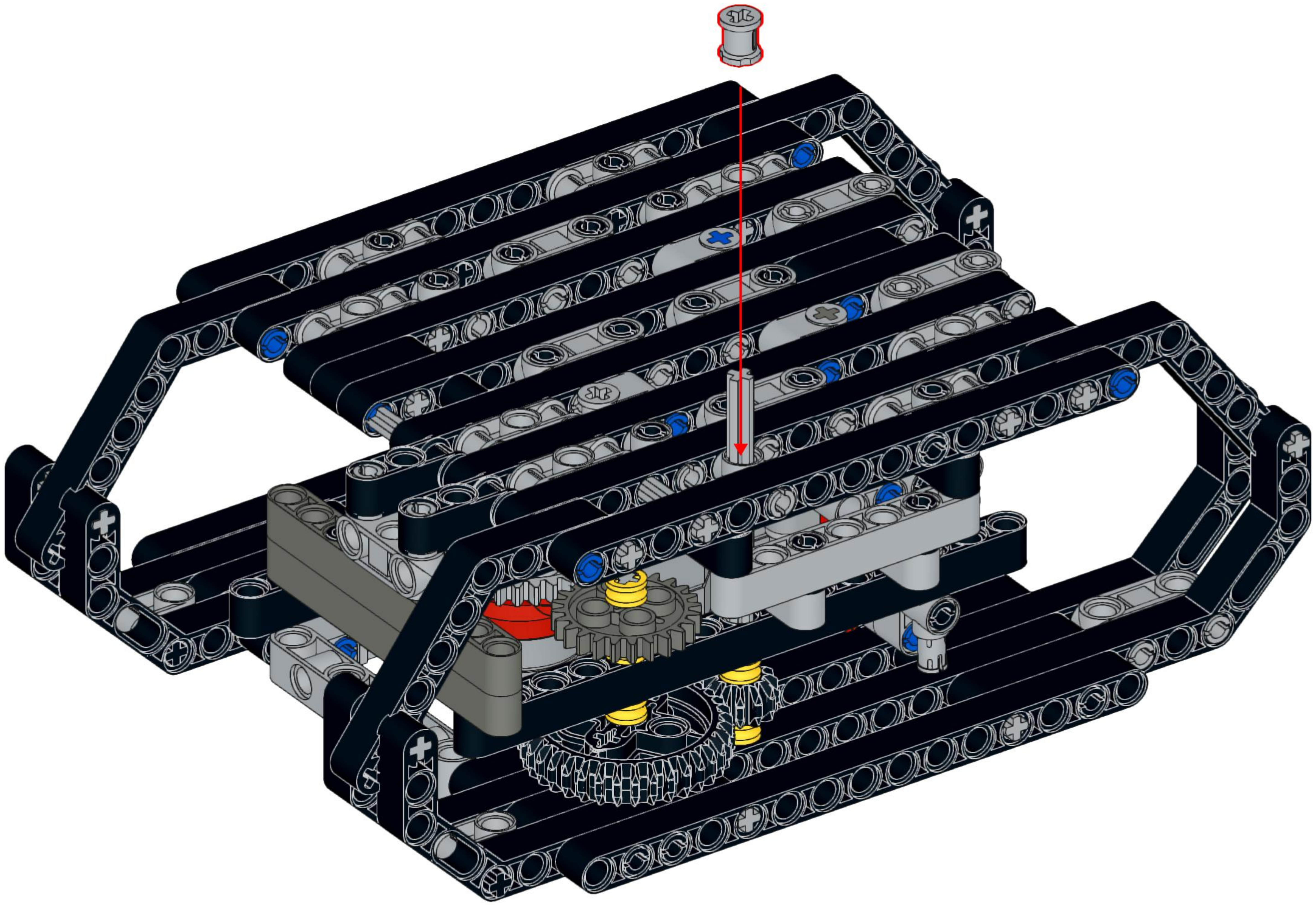

78

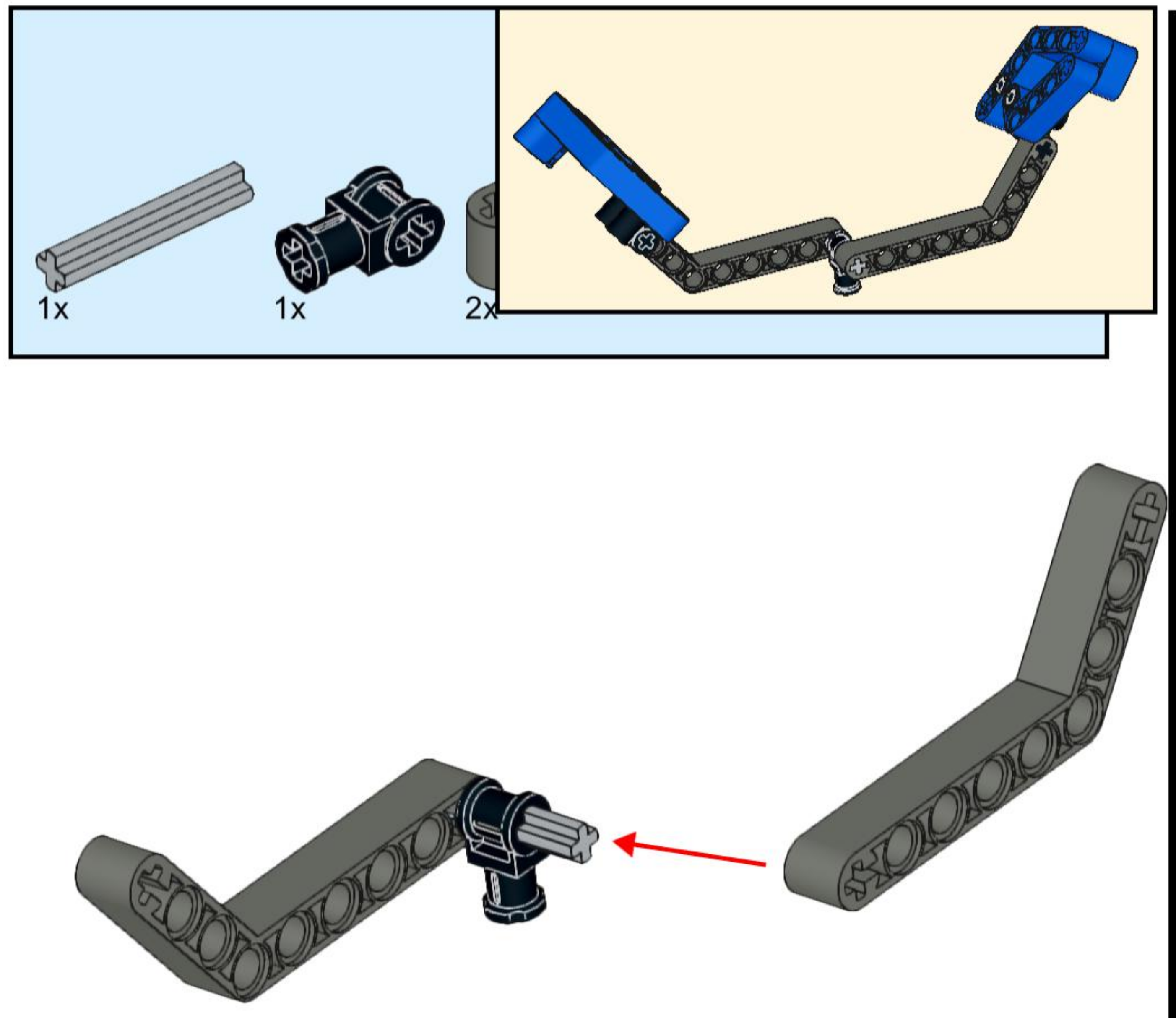


79

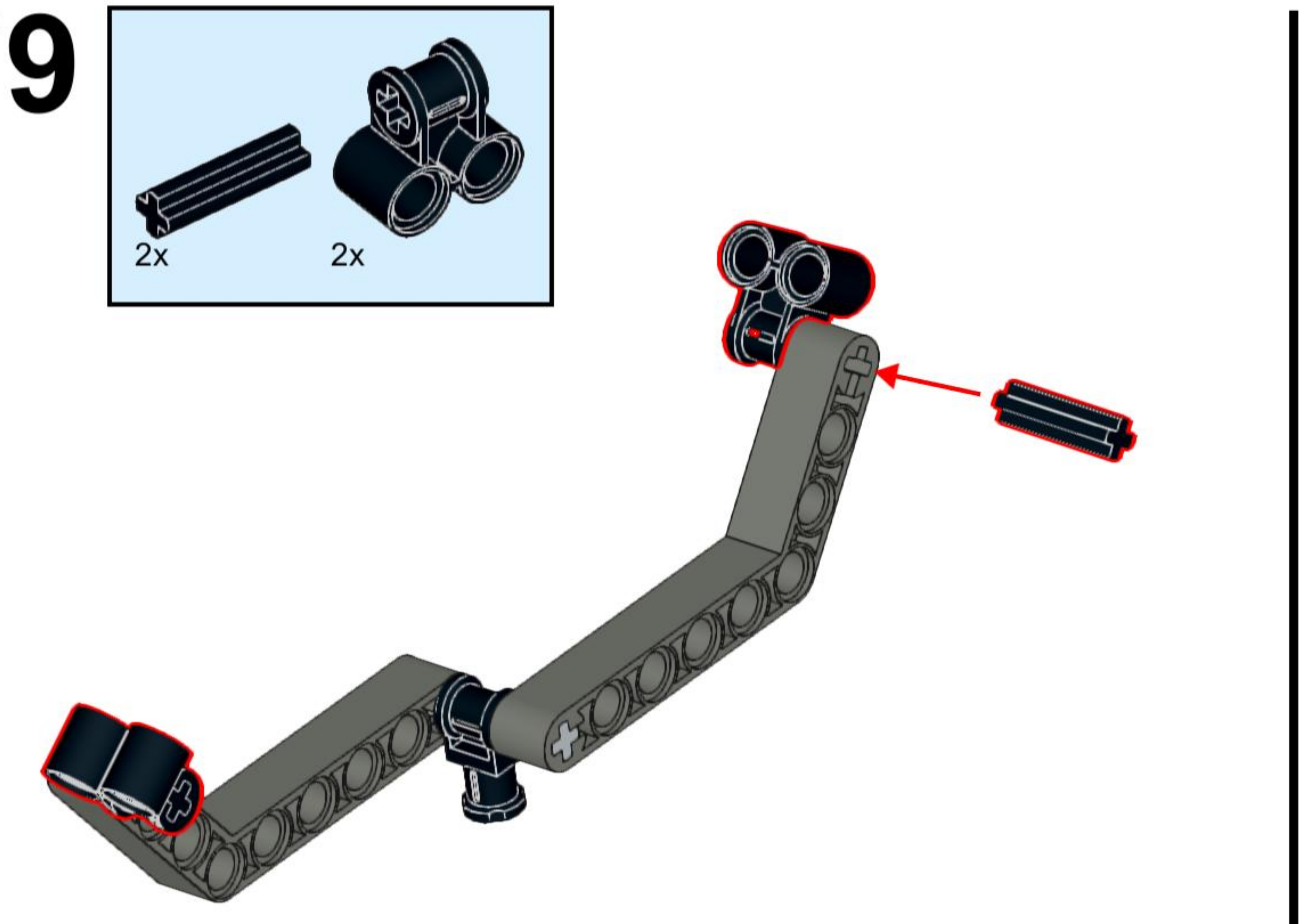


80

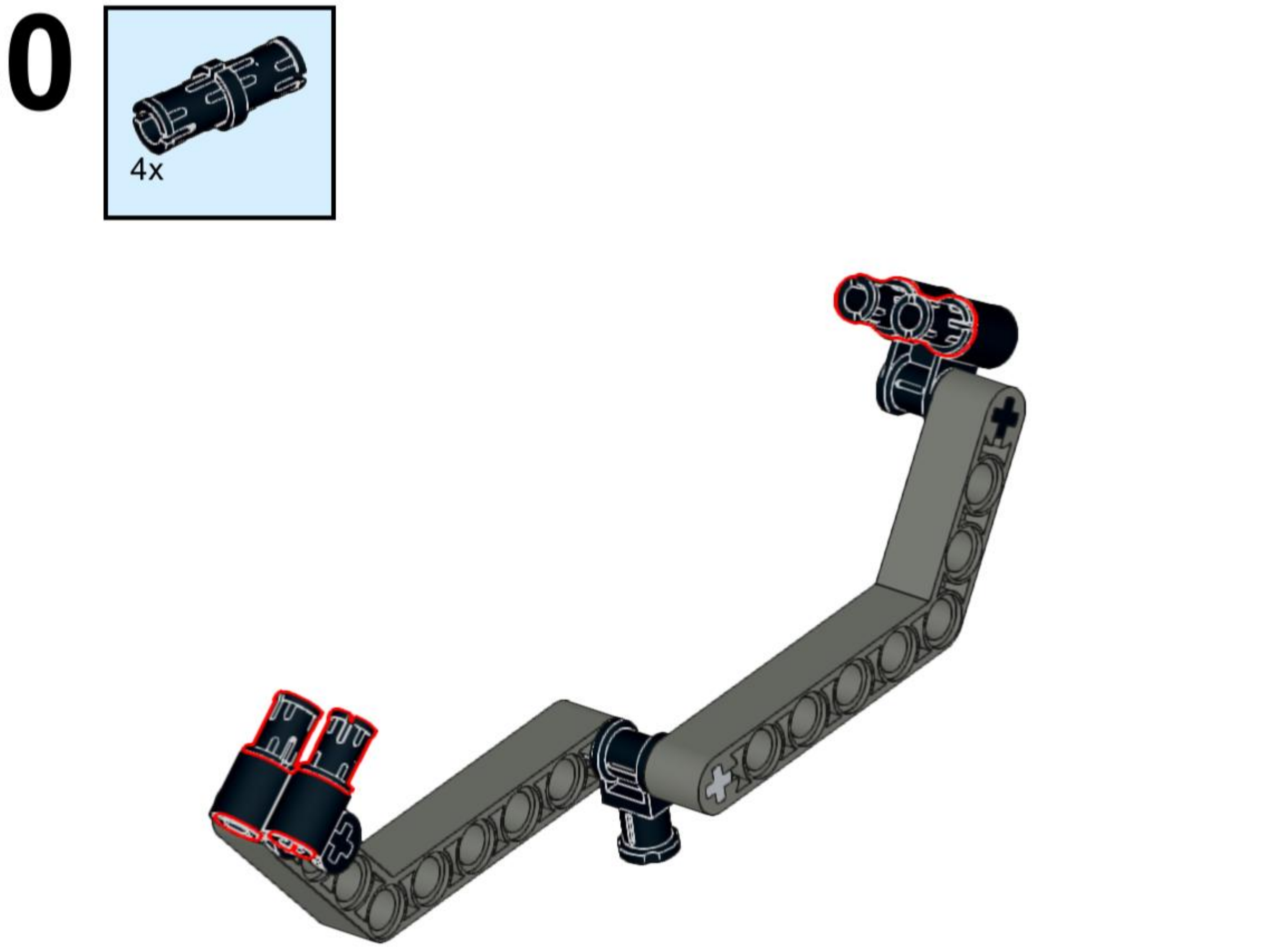


81

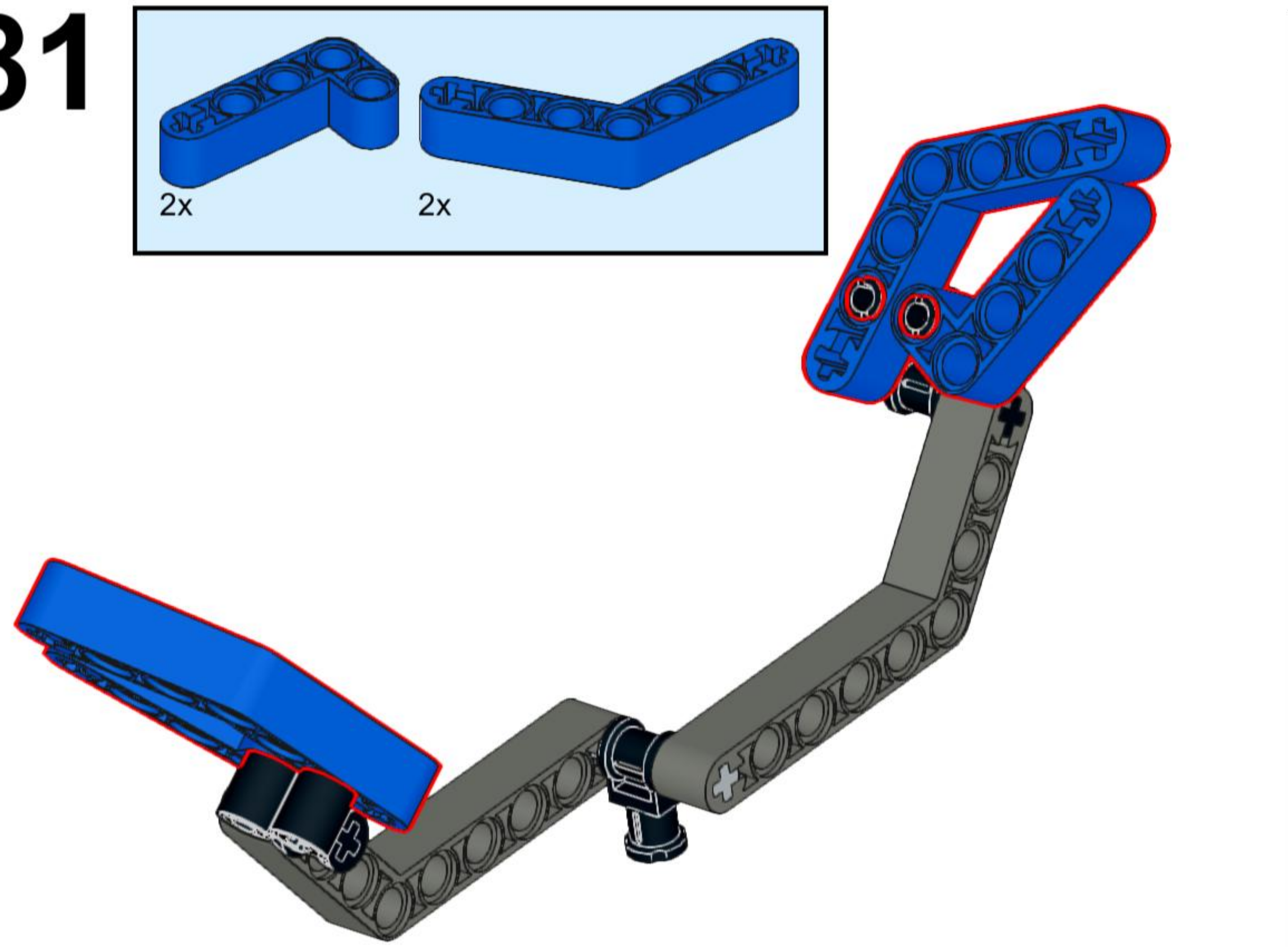


82

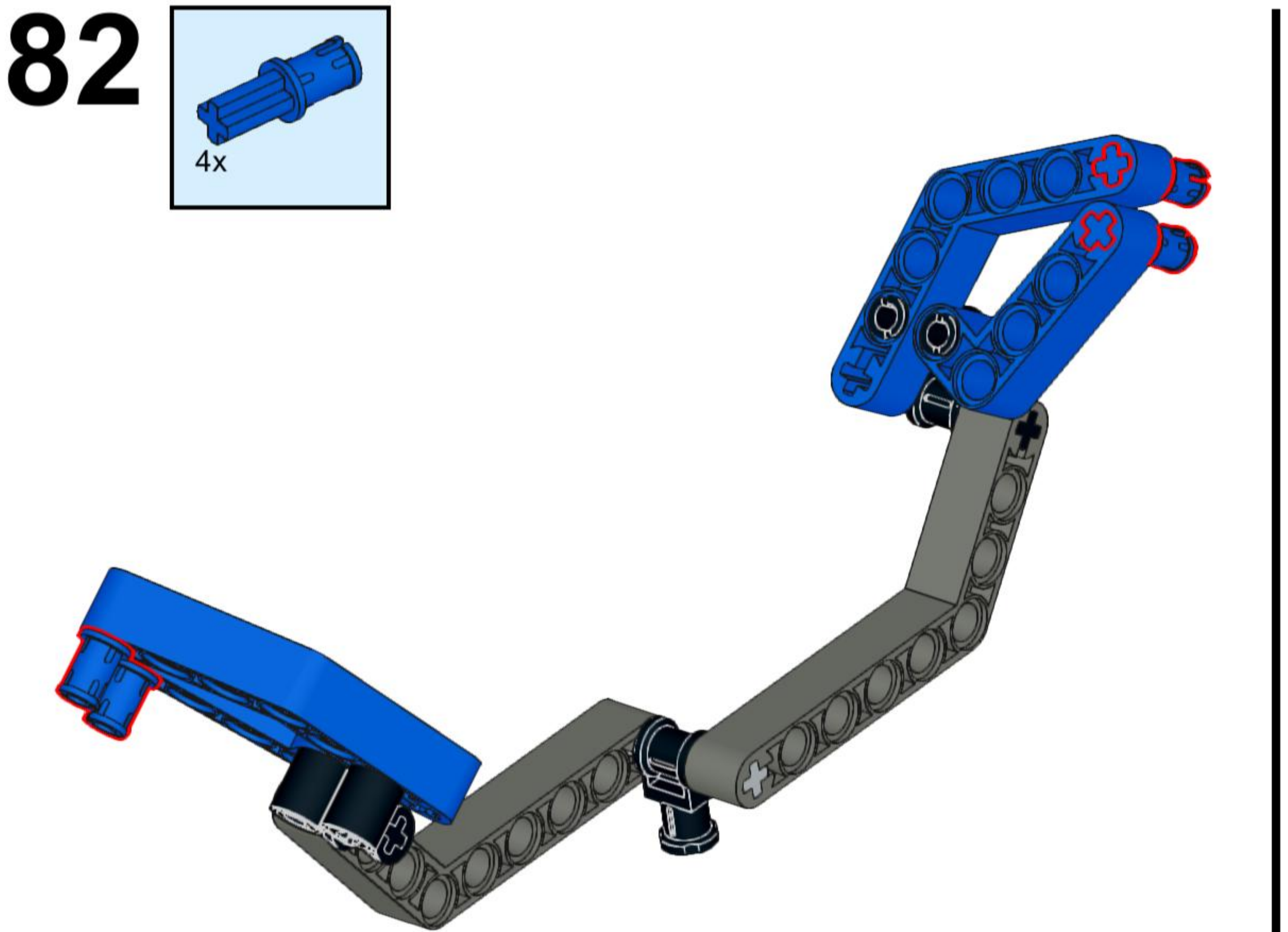


83

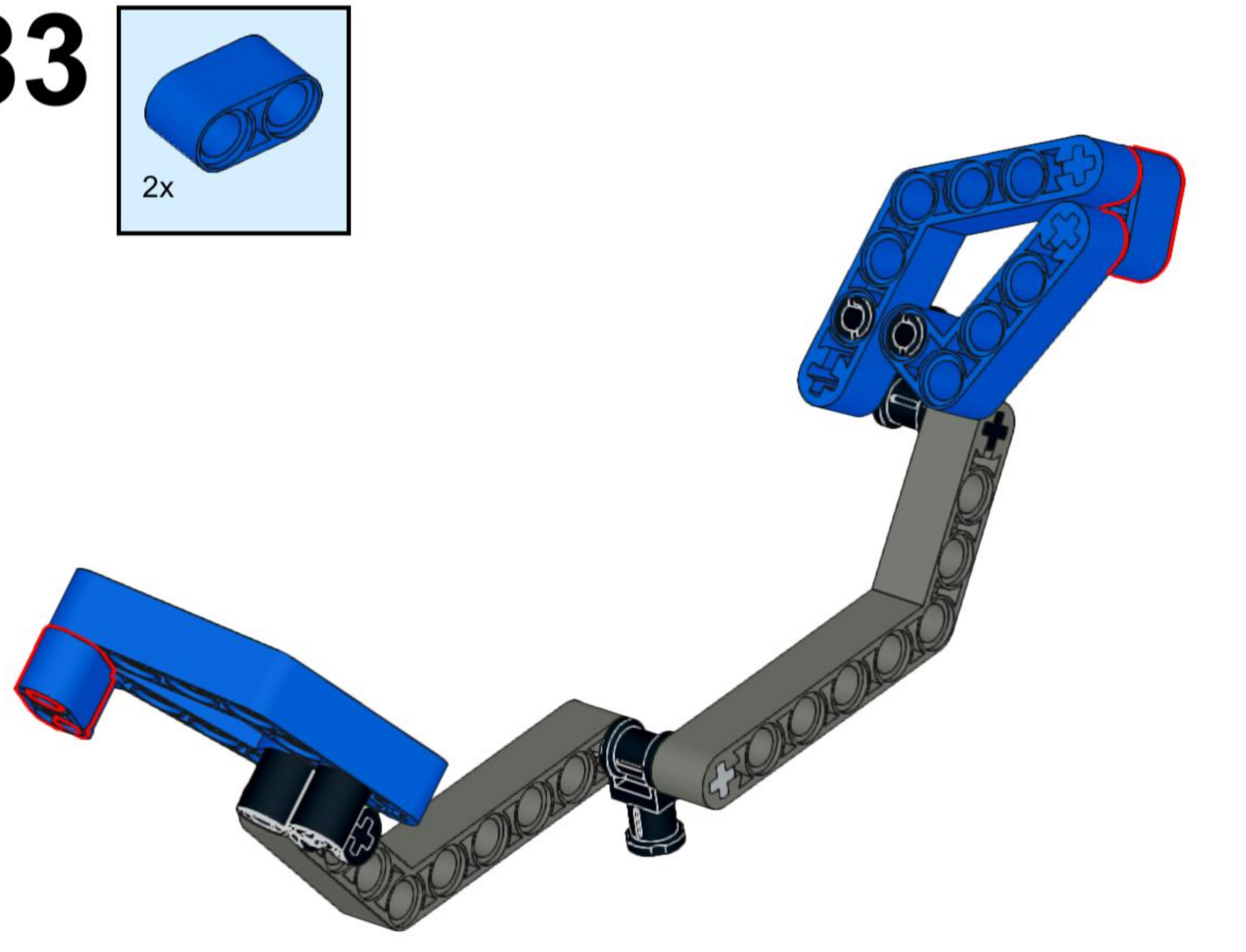

# 84

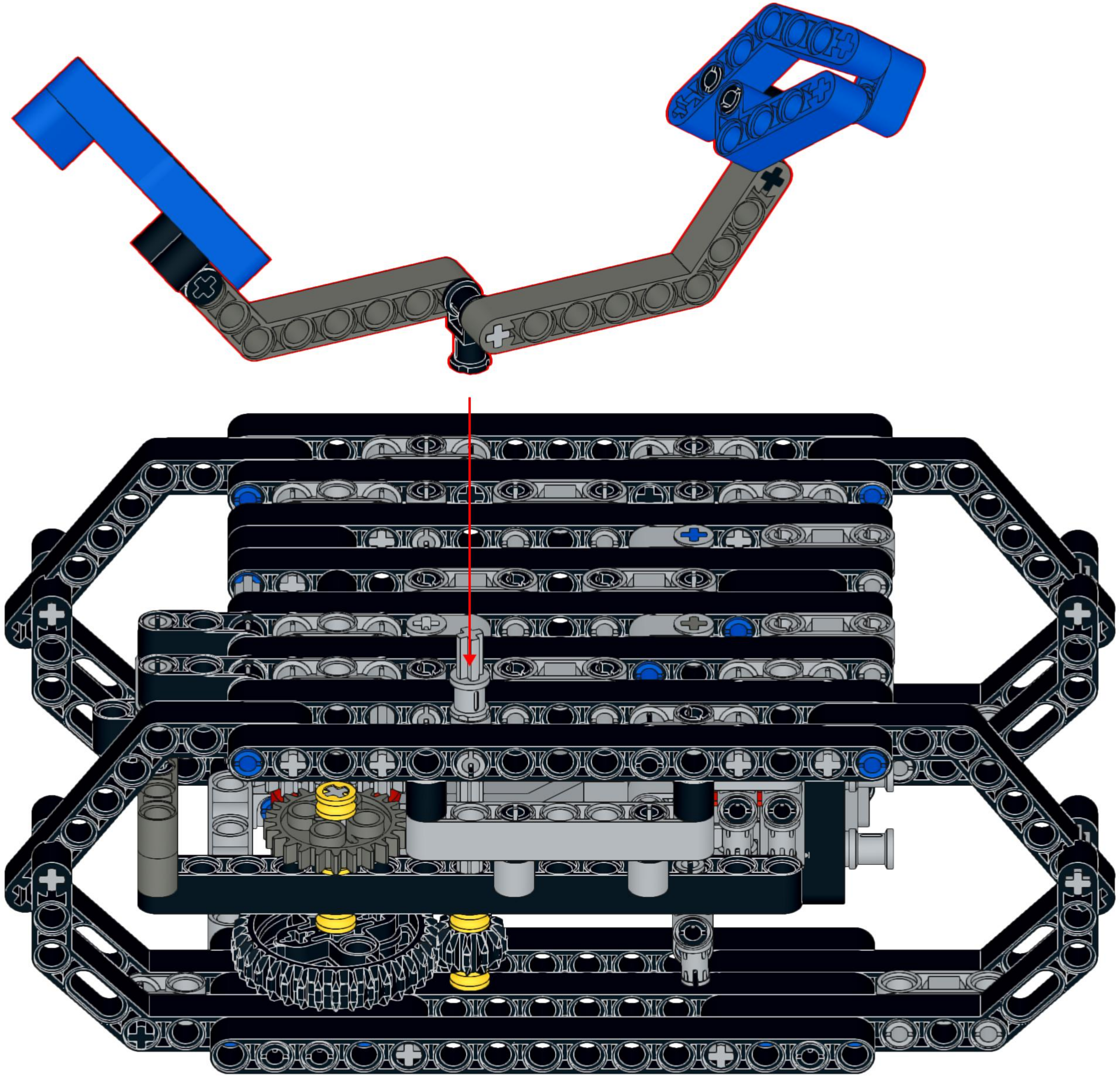

## 85

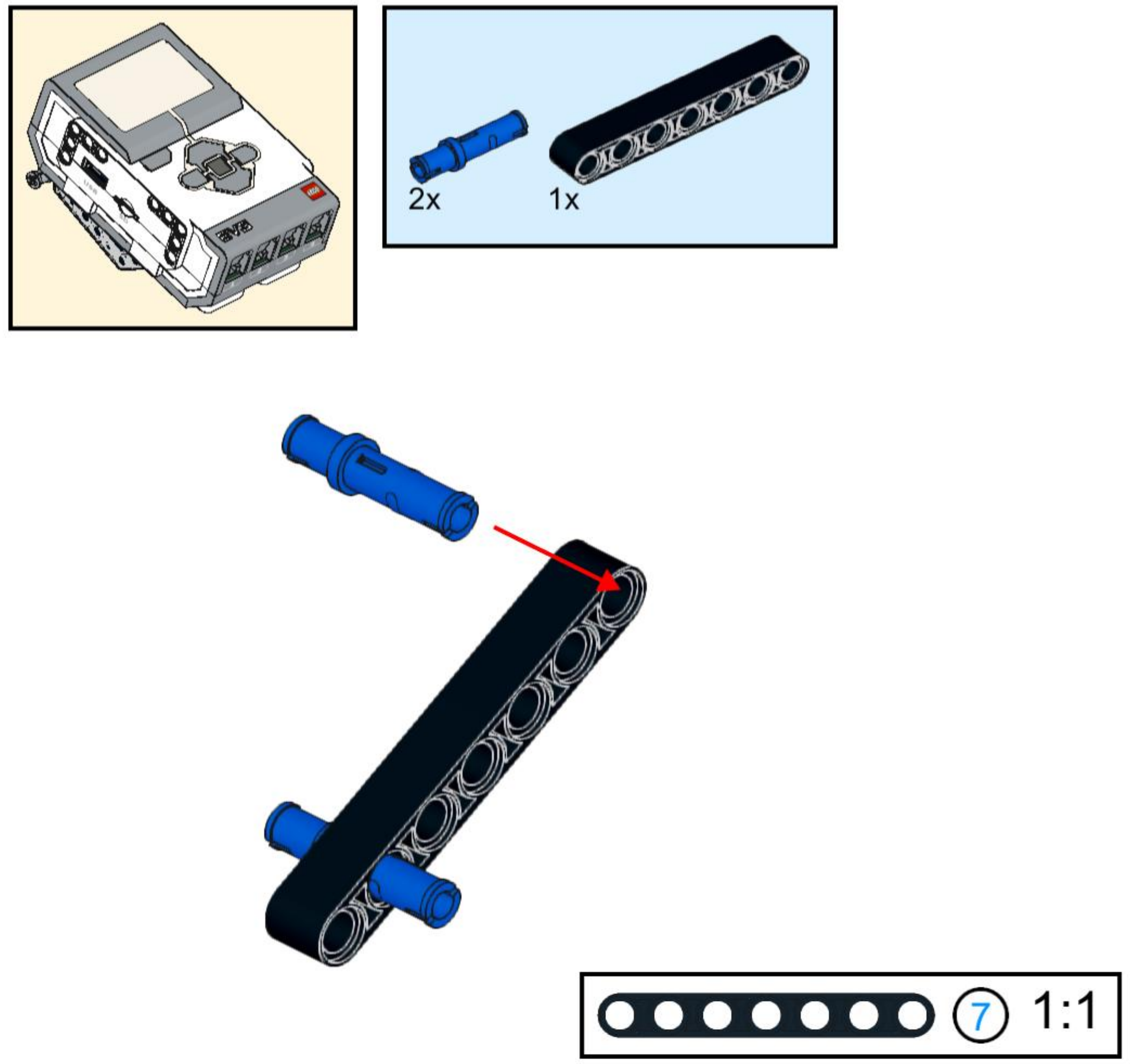


## 86

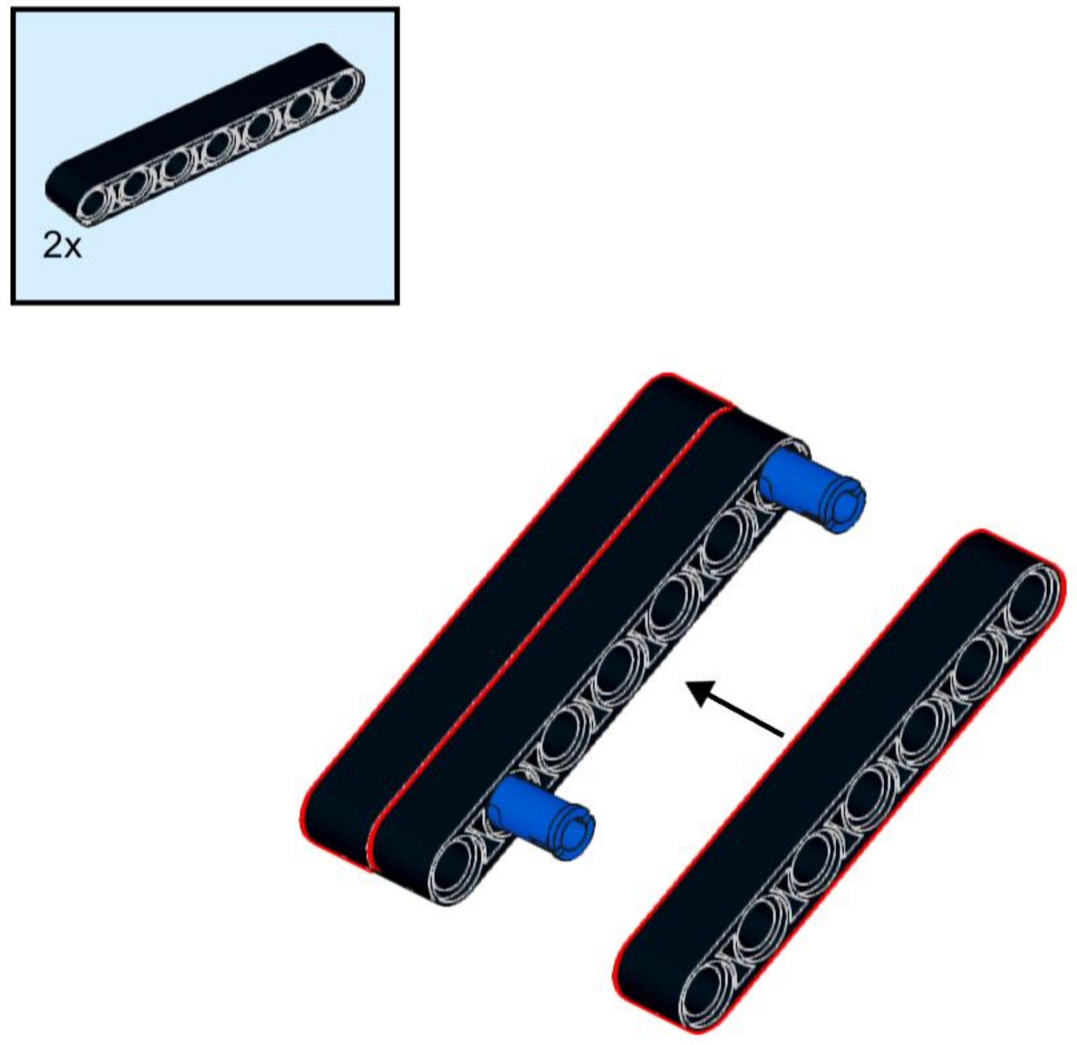


## 87

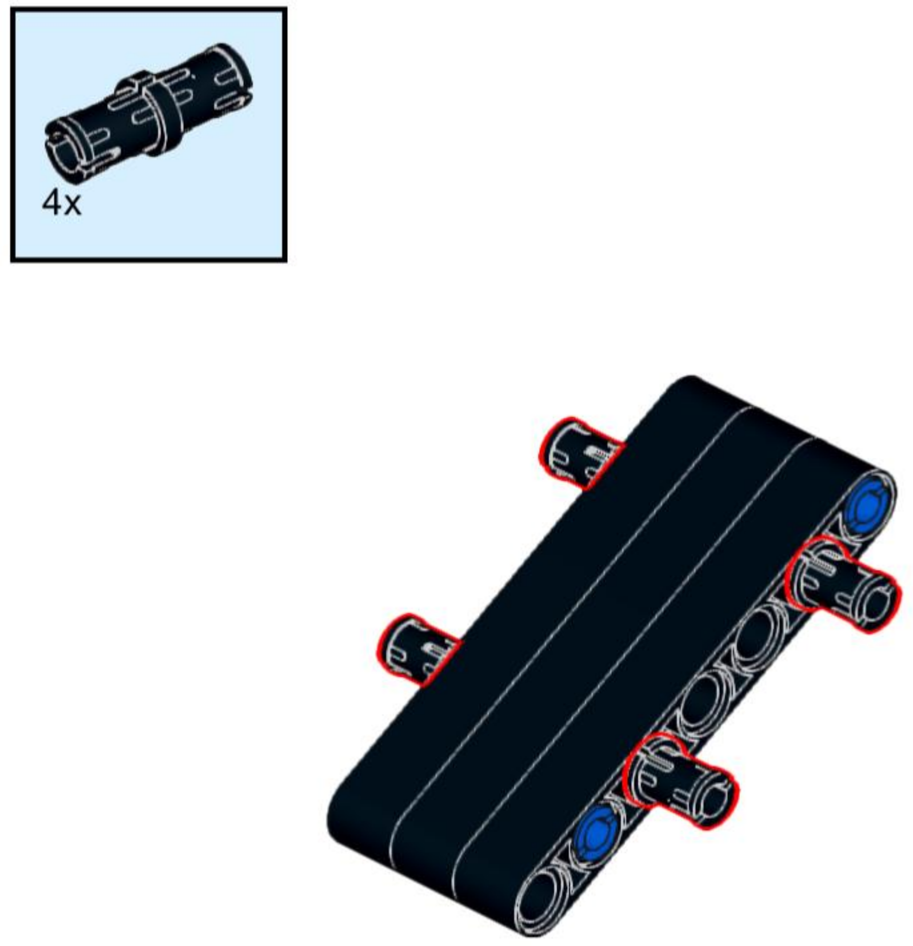


## 88

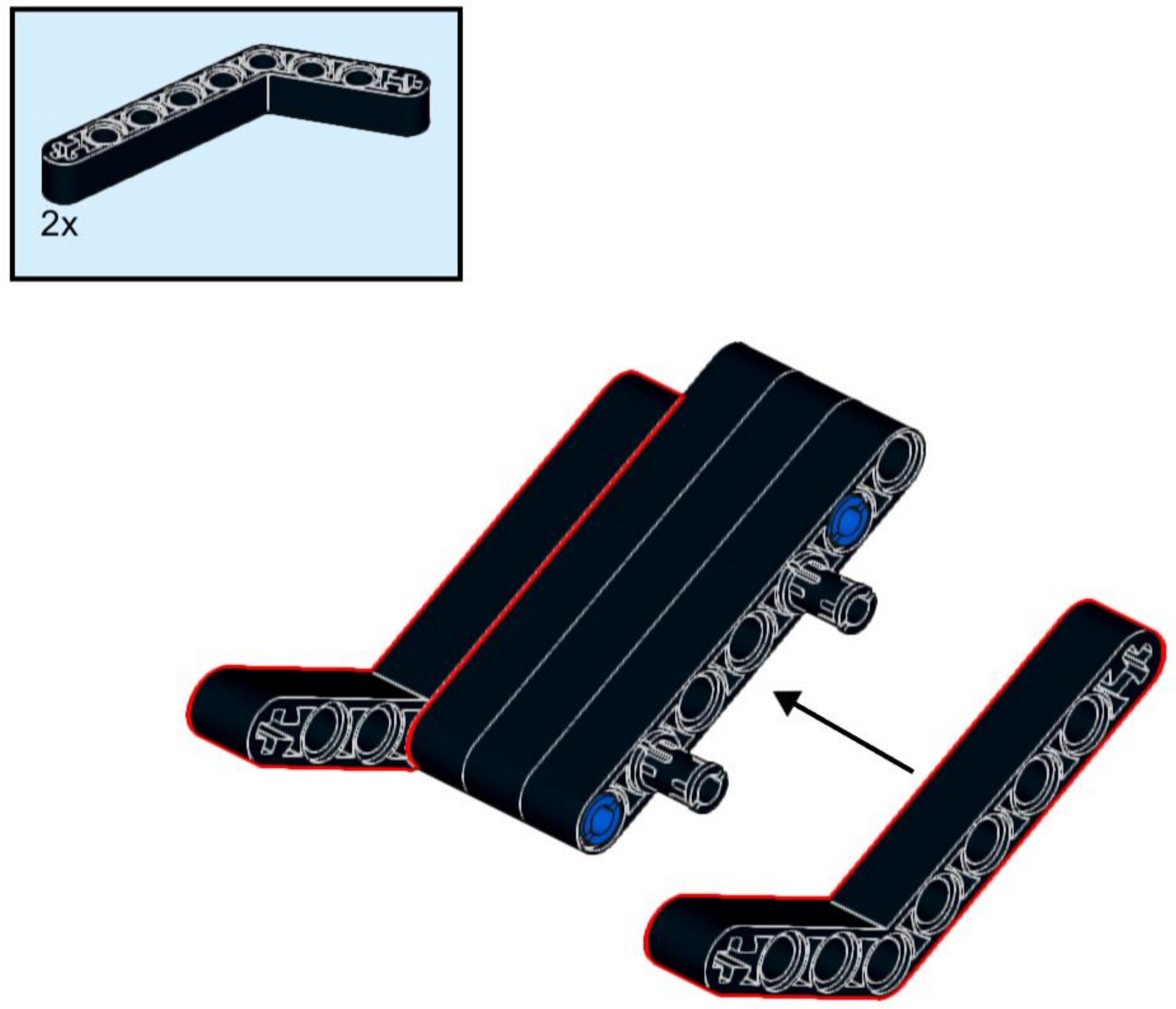


## 89

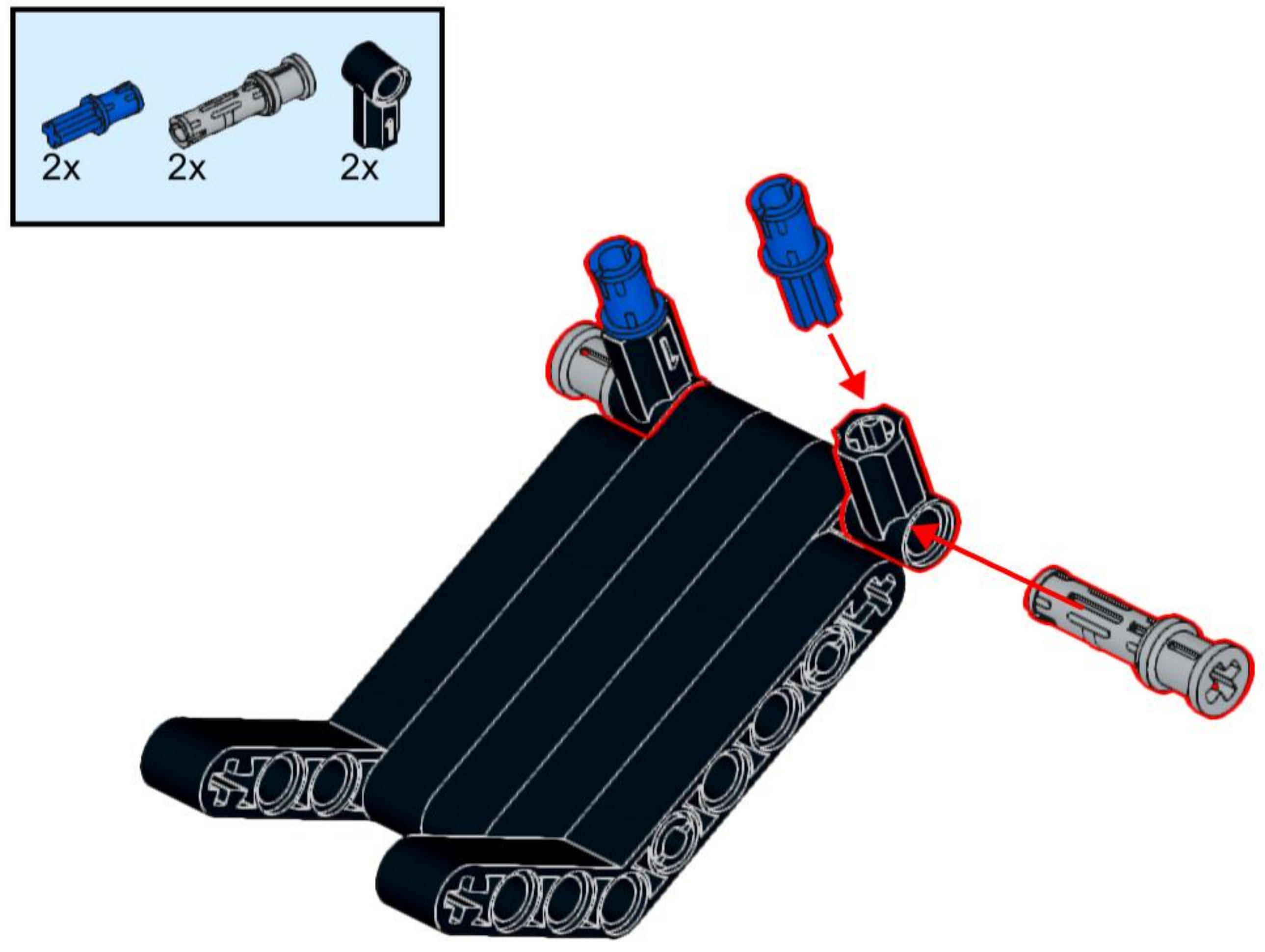


## 90

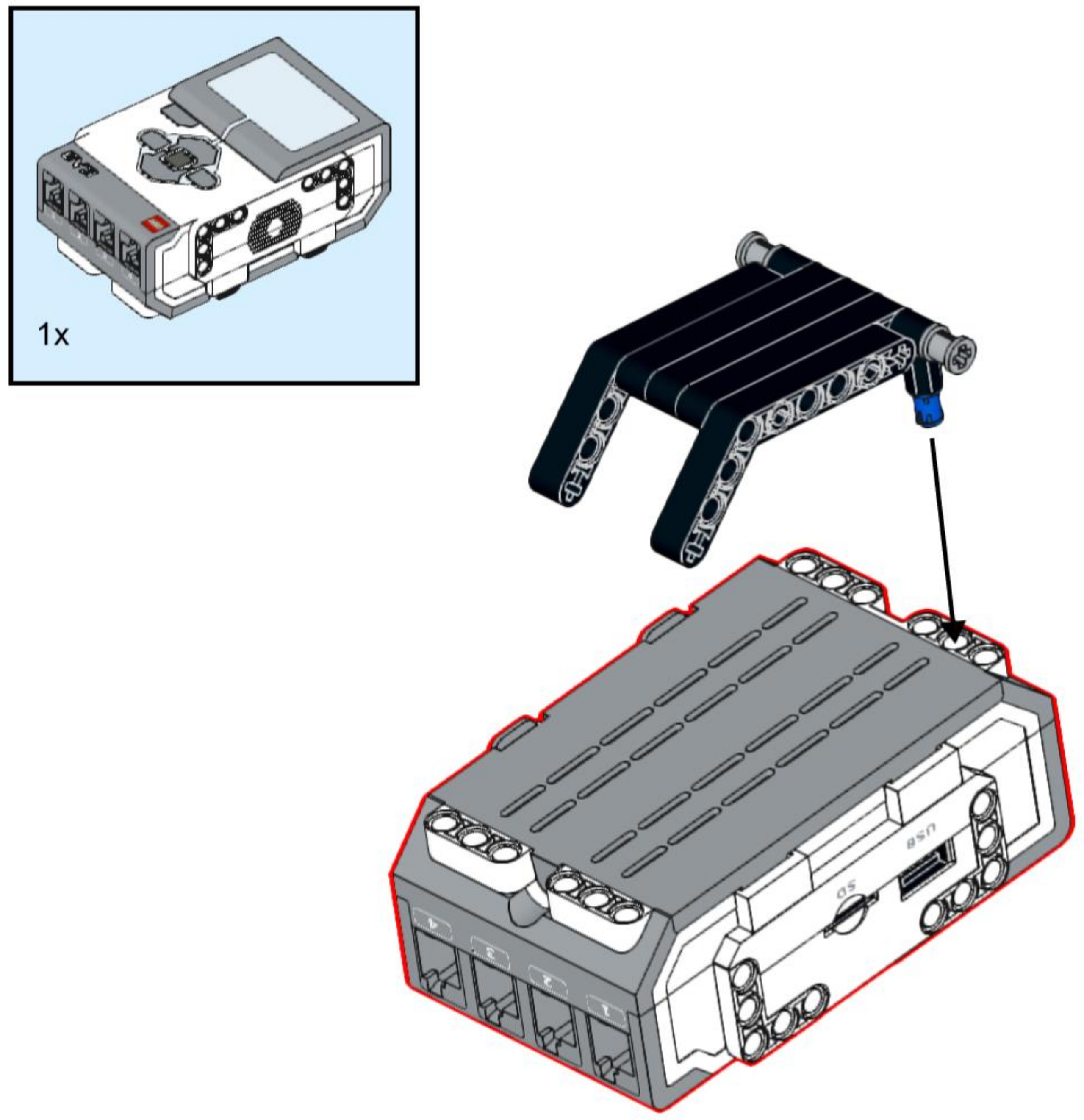

# 91

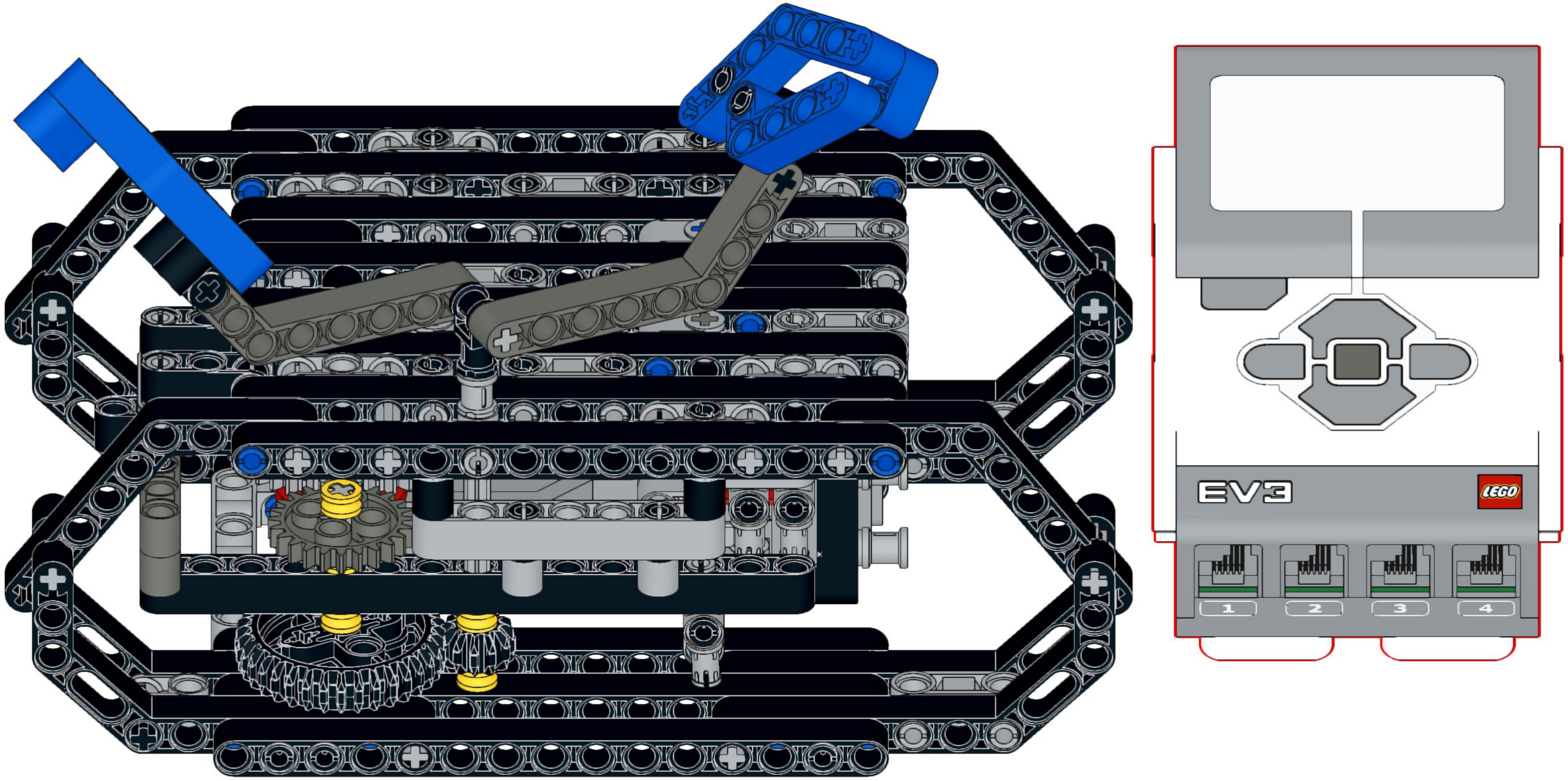

Supplement: S2 File — (PDF) [file pone.0326938.s002.pdf]
